# A combined experimental and theoretical study on the reactivity of nitrenes and nitrene radical anions

**DOI:** 10.1038/s41467-021-27687-6

**Published:** 2022-01-10

**Authors:** Yujing Guo, Chao Pei, Rene M. Koenigs

**Affiliations:** Institute of Organic Chemistry, RWTH Aachen University, Landoltweg 1, D-52074 Aachen, Germany

**Keywords:** Photocatalysis, Catalytic mechanisms, Photocatalysis

## Abstract

Nitrene transfer reactions represent one of the key reactions to rapidly construct new carbon-nitrogen bonds and typically require transition metal catalysts to control the reactivity of the pivotal nitrene intermediate. Herein, we report on the application of iminoiodinanes in amination reactions under visible light photochemical conditions. While a triplet nitrene can be accessed under catalyst-free conditions, the use of a suitable photosensitizer allows the access of a nitrene radical anion. Computational and mechanistic studies rationalize the access and reactivity of triplet nitrene and nitrene radical anion and allow the direct comparison of both amination reagents. We conclude with applications of both reagents in organic synthesis and showcase their reactivity in the reaction with olefins, which underline their markedly distinct reactivity. Both reagents can be accessed under mild reaction conditions at room temperature without the necessity to exclude moisture or air, which renders these metal-free, photochemical amination reactions highly practical.

## Abstract (web summary)

Gaining in-depth understanding of photochemical processes is key for developing more sustainable and efficient chemical transformations. Here the authors show that under visible light photochemical conditions, iminoiodinanes undergo formation of triplet nitrenes or nitrene radical anions, depending on the use of a photosensitizer; These reagents are studied in amination reactions with olefins.

## Introduction

Photochemistry is a classic discipline in chemistry and early reports date back even to the beginning of the 20^th^ century forecasting the potential of photochemical synthesis on the challenges of modern societies^1^. It features light as the main source of energy to conduct chemical transformations and can thus be regarded as a key pillar in the development of sustainable approaches and reduced ecological footprint. Today, photochemistry is one of the most rapidly developing fields in chemistry and the past decades witnessed the development of major milestones and concepts, such as dye-sensitized solar cells^2^, polymerization chemistry^3^, protein labeling^4^, photoredox catalysis^5–8^, and classic photochemical applications^9–11^ that leverage photochemistry as one of the key technologies in the advancement of all chemical disciplines.

**Fig. 1:**
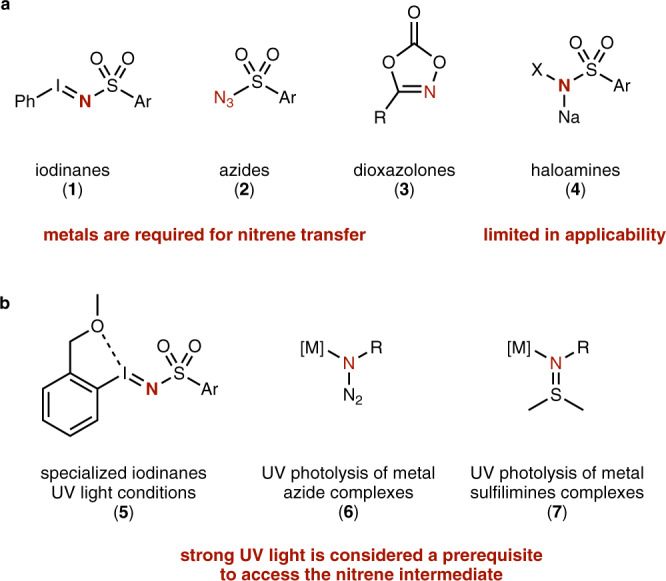
Photochemical amination reactions with nitrenes. **a** Classic precursors of nitrenes. **b** Previous applications of UV light-mediated or assisted nitrene transfer reactions.

**Fig. 2:**
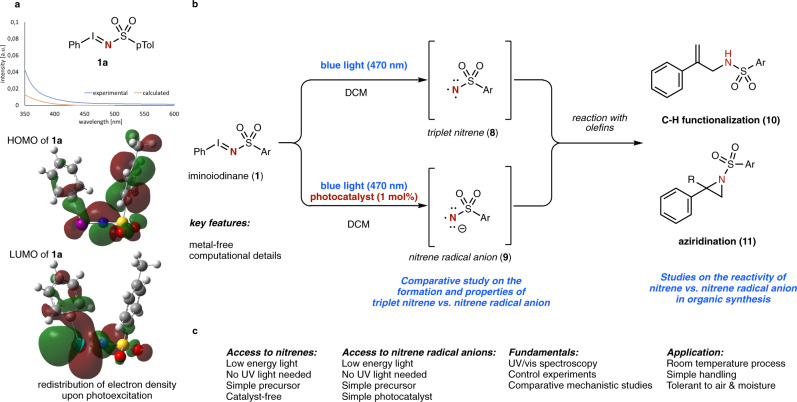
Photochemical amination reactions with nitrenes. **a** Photochemical properties of iminoiodinane **1a** and computed HOMO and LUMO. **b** Comparative study of nitrene vs. nitrene radical anions. **c** Key features of this work.

In organic synthesis, photochemistry constitutes an important strategy to access reactive intermediates via excited state chemistry of catalysts^5–8^ or reagents^11–14^. Significant advances have been made in the context of organic synthesis methodology, for example in the utilization of radicals in the presence of photoredox catalysts^5–8^ or carbenes via the photolysis reaction of appropriate reagents^11–14^. The access and application of nitrenes^15–22^ or analogs therof^23^ under visible light photochemical conditions however remains largely underestimated although it would allow for direct amination reactions with vast potential in modern drug synthesis^21,22^. Such photochemical nitrene transfer reactions would significantly expand currently available concepts in classic metal-catalyzed nitrene transfer reactions (Fig. 1a) and provide pathways under mild conditions to the pivotal nitrene intermediate. Today, essentially two strategies have been developed to access nitrenes under photochemical conditions. One concept harnesses the use of metal-catalyzed nitrene transfer reactions under UV light photochemical conditions to facilitate the formation of metal-nitrene intermediates (Fig. 1b)^24–28^. Another concept employs highly specialized, tailor-made nitrene transfer reagents (**7**), relying on strong UV-light for their photolysis^29^. UV-light can however significantly reduce reaction efficiency and increase by-product formation. Yet, UV light still remains a prerequisite to access pivotal (metal-)nitrene intermediate under photochemical conditions (Fig. 1b). The use of visible light is thus in high demand to overcome these limitations, to enable sustainable nitrene transfer reactions and to develop strategies for amination reactions (Fig. 2).

In this context, our group recently reported an initial application of nitrene radical anions in organic synthesis^30^. Their initial discovery dates back to 1980, when MacDonald described the synthesis of the phenyl nitrene radical anion^23^. Applications in organic synthesis however remained scarce and detailed, comparative studies on the properties of nitrene and nitrene radical anion remain elusive. Such studies would allow for fundamental understanding of similarities and differences of chemical properties and reactivity of these monovalent, nitrogen-based reactive intermediates and can provide the basis for further development of metal-free amination reactions (Fig. 2).

In this work, we show that iminoiodinanes are suitable, bench-stable reagents that can be used to access either a triplet nitrene or a nitrene radical anion intermediate under photochemical or photocatalytic conditions, respectively. We showcase the reactivity of these monovalent nitrogen-based reactive intermediates in the reaction with olefins that can lead either to aziridination or C-H amination reactions. Experimental and computational studies are discussed to rationalize for the observed experimental data.

## Results

### Reaction optimization

The utilization of simple, readily available nitrene transfer reagents can be regarded as a prerequisite for the realization of photochemical amination reactions. We therefore decided to initially study the photochemical properties of iodinane **1** and phenyl azide, which are commonly applied reagents in metal-catalyzed nitrene transfer reactions. While phenyl azide did not show absorbance in the visible light region, a range of different, readily available iminoiodinanes **1** revealed a weak absorbance in the visible light region as determined by both experiment and theory (Fig. 2a and Supplementary Fig. 1). An important observation was made for the electron distribution of the HOMO and LUMO. While the HOMO of **1a** is predominantly associated to electron density at the nitrogen atom (Fig. 2a), the electron distribution in the LUMO is shifted to the iodine atom (Fig. 2a and Supplementary Fig. 6), which could facilitate photolysis reactions and formation of nitrene intermediates.

Based on this observation, we investigated the reaction of α-methyl styrene **12a** with iminoiodinane **1a** under photochemical conditions (Table 1). Indeed, a very efficient amination reaction was observed in the presence of blue light (470 nm) that lead to the selective formation of the C-H functionalization product **10a** without accompanying by-products from aziridination (**11a**). Other light sources proved by far less efficient, which might be related to weaker absorption or side-reactions due to the high-energy UV light. A surprising observation was made when switching to photocatalytic reaction conditions. Using simple [Ru(bpy)_3_]Cl_2_ as photocatalyst, a complete reversal of reactivity was observed and selective aziridination reaction to yield **11a** occurred. Other photocatalysts, such as [Ru(bpz)_3_](PF_6_)_2_, iridium-based photocatalysts, or organic dyes did not alter the reactivity and the C-H functionalization product **10a** was formed selectively. Only in the case of Eosin Y as photocatalyst, a mixture of C-H amination (**10a**) and aziridination (**11a**) was observed. Further optimization steps included investigations on the solvent, concentration, reaction stoichiometry, yet no further improvements were observed (Supplementary Table 1).

**Table 1:** Investigations on the photochemical and photocatalytic reaction iminoiodinane **1a** with α-methyl styrene **12a**.


Entry^*a*^	Light source	Photocatalyst	%Yield (10a)	%Yield (11a)
1	385 nm	–	22	–
2	470 nm	–	70	–
3	530 nm	–	40	–
4	White light	–	29	–
5	In the dark	–	n.r.	n.r.
6	470 nm	Ru(bpy)_3_Cl_2_	–	80
7	470 nm	Ru(bpz)_3_(PF_6_)_2_	35	–
8	470 nm	Ir(ppy)_3_	27	–
9	470 nm	(Ir[(dF(CF_3_)ppy]_2_(dtbpy)PF_6_	30	–
10	470 nm	4-CzlPN	51	–
11	470 nm	Eosin Y	34	45

*n.r.* no reaction.

^a^Reaction conditions: **12a** (1 mmol, 5 equiv.), **1a** (0.2 mmol) and the respective photocatalyst (1 mol%) were dissolved in 2.0 mL DCM under air atmosphere. The mixture was irradiated with the light source (3 W) indicated for 4 h at room temperature.

### Comparative study on the reaction mechanism

To further explore this divergent reactivity, we examined control experiments to provide an understanding of the underlying reaction mechanism. No notable association of α-methyl styrene **12a** or phenyl iodide with iminoiodinane **1a** were observed by ^1^H-NMR studies, suggesting no association of reagents. Similarly, no notable decomposition of phenyl iodide under 470 nm blue light irradiation was observed within 4 h, which suggests that iodine species do not participate as hidden catalysts in the reaction.

**Fig. 3:**
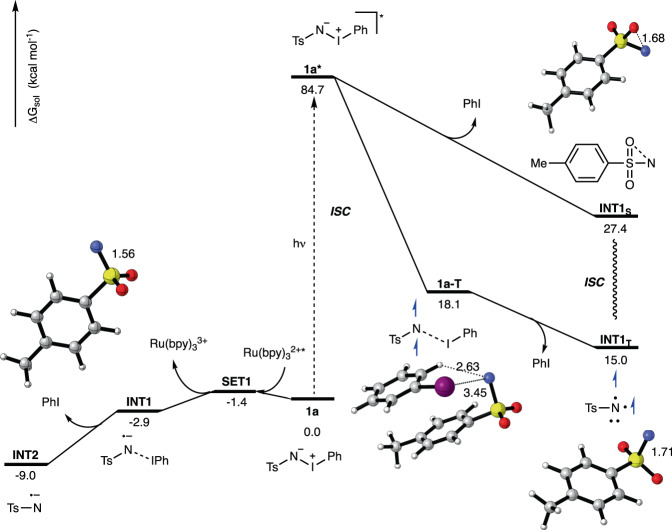
theoretical calculations on the formation of the nitrene intermediate; calculations were performed at the SMD(DCM)-(U)M06-2X-D3/def2-TZVP//(U)M06-2X-D3/def2-SVP level of theory. Gibbs energies are given in kcal mol^−1^.

Further studies on the reaction mechanism involved theoretical calculations to better rationalize this photochemical nitrene transfer reaction (Fig. 3). We therefore conducted calculations using (TD)-DFT methods to rationalize the photochemical reaction of iminoiodinane **1a** and DFT methods for the catalytic reaction involving photosensitizers. These calculations reveal that photochemical excitation of **1a** leads to formation of an excited state 1a* that remains in singlet state. This excited state can now undergo two different pathways: A) relaxation on the singlet spin surface leads to the direct formation of a singlet nitrene that features a very short N-O distance, which can be interpreted as a stabilization of the low-valent nitrene with the lone pair of an oxygen atom of the pendant sulfonyl group. B) relaxation involving intersystem crossing (ISC) results in the formation of a triplet intermediate **1a-T**, which features a very long N-I bond, close to a non-bonding situation. Scanning of different N-I bond lengths indicated that further elongation of the N-I bond proceeds in a barrier-free fashion to directly lead to a triplet nitrene intermediate. This triplet nitrene intermediate is the energetically favored intermediate and can alternatively be accessed from the high-lying singlet nitrene via intersystem crossing. This theoretical analysis of the photochemistry of iminioiodinane **1a** now rationalizes for the formation of a triplet nitrene intermediate under photochemical conditions and shows a marked difference to the ground state reactivity^31^. Under photocatalytic conditions, calculations are in line with our previous report^30^ and a very facile reduction of the iodinane, leading to iodinane radical anion **INT1**. This anion features a very long N-I bond that is close to non-existent and therefore a rapid cleavage of the N-I bond can occur in a barrierless process to give a nitrene radical anion.

For further analysis and understanding of the reactivity of triplet nitrene and nitrene radical anion, we examined their structural and electronic properties. The calculations show that the nitrene radical anion possesses a higher electron density at the nitrogen atom compared to the triplet nitrene, which in turn leads to higher nucleophilicty of the nitrene radical anion (Supplementary Figs. 4, 7, and 8). For stabilization of the negative charge, the sulfonyl group plays an important role as an electron acceptor leading to mesomeric stabilization of the nitrene radical anion. The aromatic ring plays only a minor role in stabilization of the nitrene radical anion. The analysis of bond lengths further supports this mesomeric effect within the nitrene radical anion. Despite of the additional negative charge, the S-N bond shortens upon formal reduction from triplet nitrene to nitrene radical anion from 1.71 Å to 1.56 Å and thus resembles more an S-N double bond in the case of the nitrene radical anion, which is also reflected by bond order analysis (Supplementary Fig. 5).

**Fig. 4:**
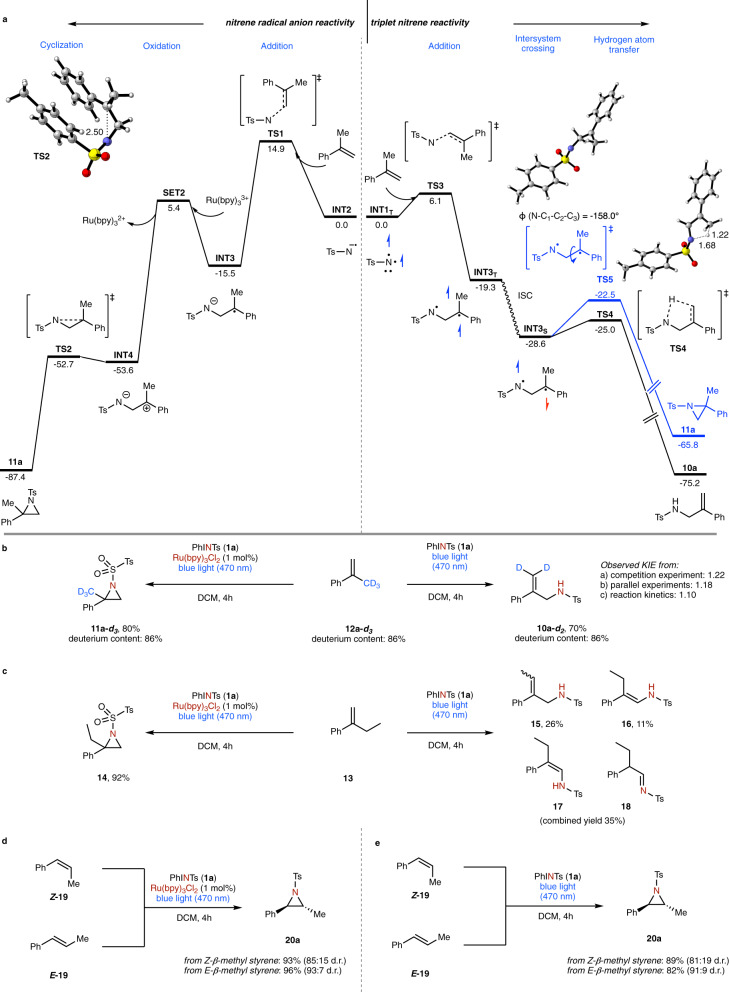
Control experiments and theoretical calculations on the formation of the nitrene intermediate; calculations were performed at the SMD(DCM)-(U)M06-2X-D3/def2-TZVP//(U)M06-2X-D3/def2-SVP level of theory. **a** Energy surface of the reaction of triplet nitrene and nitrene radical anion. Gibbs energies are given in kcal mol^−1^. **b** Reaction with deuterated α-methyl styrene. **c** Reaction with α-ethyl styrene. **d** Photocatalytic reaction with β-methyl styrene. **e** Photochemical reaction with β-methyl styrene.

Further exploration concerned the evaluation of the reaction mechanism and an understanding of the distinct reactivity of triplet nitrene and nitrene radical anion by theory and experiment (Fig. 4). In the case of the triplet nitrene, we examined two different reaction pathways using DFT calculcations (Fig. 4a, right). A first pathway involves hydrogen atom transfer from α-methyl styrene to the triplet nitrene intermediate, yet an activation free energy of 14.7 kcal mol^−1^ and the formation of two separate radicals that need intermolecular radical recombination renders this pathway not feasible (Supplementary Fig. 11, **TS6**). A second pathway involves the addition of the triplet nitrene to α-methyl styrene via a **TS3** with an activation free energy of only 6.1 kcal mol^−1^, which is significantly favored over the hydrogen transfer pathway. This addition product can undergo intersystem crossing to give an open shell singlet species that can either cyclize to give the aziridine **11a**, or undergo hydrogen atom transfer to give the C-H functionalization product **10a**. Analysis of the respective transition states reveals significant steric hindrance of rotation around the central C-C bond, which renders cyclization energetically unfavorable over hydrogen atom transfer. The latter is favored by 2.5 kcal mol^−1^ and now reasons the reactivity of triplet nitrene intermediates with α-methyl styrene to yield the C-H functionalization product **10a**. Further studies concerned the reaction of the nitrene radical anion (Fig. 4a, left). In this case, the radical addition to the olefin occurs with a significantly higher activation free energy as compared to the triplet nitrene, which is reasoned by higher electron density. Subsequent reduction by the oxidized state of the photoredox catalyst furnishes a zwitterionic intermediate **INT4** that quickly undergoes cyclization to give the aziridine product **11a** (Supplementary Fig. 10).

To examine the above pathways experimentally, we first probed the reaction mechanism with α-trideuteriomethyl styrene **12a-*d***_***3***_ under the optimized reaction conditions (Fig. 4b). The deuterium content was fully preserved under both photochemical and photocatalytic conditions. Furthermore the aziridine was selectively formed and the trideuteriomethyl-substituted aziridine **11a-*d***_***3***_ was obtained as the sole reaction product. In the case of the photochemical reaction, the deuterium label was found exclusively in the olefinic position of the reaction product, thus supporting the addition mechanism. Studies on the reaction kinetics of **12a** and **12a-*d***_***3***_ revealed only a very small kinetic isotope effect, which suggests that the hydrogen atom transfer does not occur in the rate determining step, as suggested by the theoretical calculations (Fig. 4b, and Supplementary Fig. 3). Further control experiments involved the reaction of α-ethyl styrene (Fig. 4c). While selective aziridination was observed under photocatalytic reaction conditions, a mixture of different products (**15**–**18**) that can arise from an addition – hydrogen atom transfer mechanism were observed under photochemical conditions. The theoretical analysis of reaction pathways for the hydrogen atom transfer revealed that pathways involving different hydrogen atom transfer can occur due to the presence of the ethyl group. These are very similar in energy and thus reason the unselective reaction and represent a limitation of the present C-H amination method in the case different hydrogen atom transfer reactions are possible (Supplementary Fig. 14). When studying both individual isomers of β-methyl styrene (***Z*-19** and ***E*-19**), we could observe a selective reaction towards the *trans*-aziridine **20**, which is indicative of a stepwise mechanism under both photochemical and photocatalytic reaction conditions. Further control experiments involved the use of spin trapping reagents, such as 2,2,6,6-Tetramethylpiperidinyloxyl (TEMPO), 5,5-dimethyl-1-pyrroline-1-oxide (DMPO), or DNP, and in all cases a complete suppression of the photochemical and photocatalytic reactions were observed, which is indicative of radical intermediates or participating in the reaction mechanism (Supplementary Tables 2 and 3).

### Application in synthesis

**Fig. 5:**
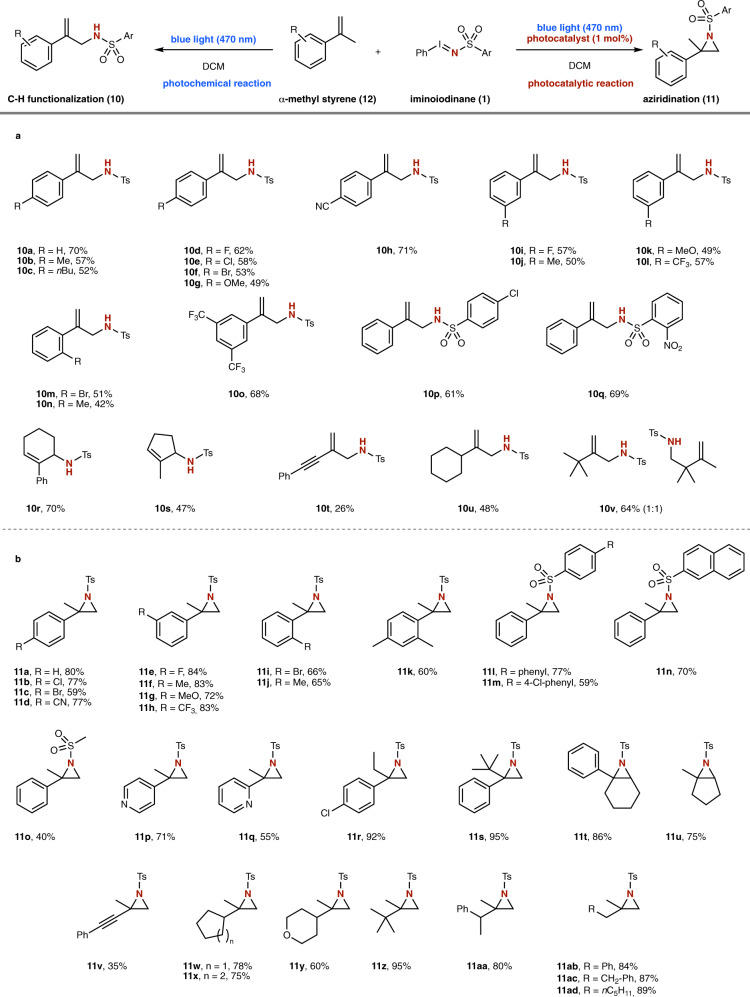
Scope of the nitrene transfer reactions in the photochemical C-H functionalization and photocatalytic aziridination reaction via nitrene radical anions. **a** Photochemical reaction with α-methyl styrenes. **b** Photocatalytic reaction with α-methyl styrenes.

We next turned our attention towards applications of the above protocols in amination reactions (Fig. 5). The photochemical C-H functionalization reaction of α-methyl styrenes **12** proceeded smoothly and the allylamine products **10a–q** were obtained in good isolated yield, without formation of by-products from aziridination (Fig. 5a). Halogens, electron-withdrawing or -donating groups in all positions of the aromatic ring were compatible and the corresponding products were obtained in high isolated yields (**10a**–**o**). Importantly, *ortho*-substitution (**10** **m, n**) had a slightly detrimental effect on the product yield. Similarly, different sulfonyl groups were tolerated under the present photochemical reaction conditions and the allyl amines products were obtained in high isolated yield (**10p, q**). When applying this photochemical protocol to cyclic, trisubstitued, cyclic olefins, 1-alkynyl-1-methyl or aliphatic 1,2-disubstituted olefins, the C-H amination products **10r**–**v** were selectively obtained in good isolated yield. Then, the photocatalytic protocol was employed in the aziridination of α-methyl styrenes (Fig. 5b). We examined a similar range of substituents, including halogen, alkyl, cyano, ether or alkoxy substituents, at both the sulfonyl group and the benzene ring of the α-methyl styrene, which proved compatible under photocatalytic conditions. Only in the case of *ortho*-substitution slightly reduced reaction yields were obtained (**11i**–**k**). Most notably, in this case also different α-alkyl-substituted styrene derivatives, such as a very bulky *tert*.-butyl group (**11s**), and nucleophilic *N*-heterocycles (**11p**, **q**) were well tolerated to yield the corresponding aziridines in high yield, which are commonly very challenging substrates in aziridination reactions. Furthermore, a range of trisubstitued, cyclic olefins, and further examples of 1,1-disusbtituted olefins were studied under the present photocatalytic conditions to yield the aziridine products **11t**–**11ad**.

**Fig. 6:**
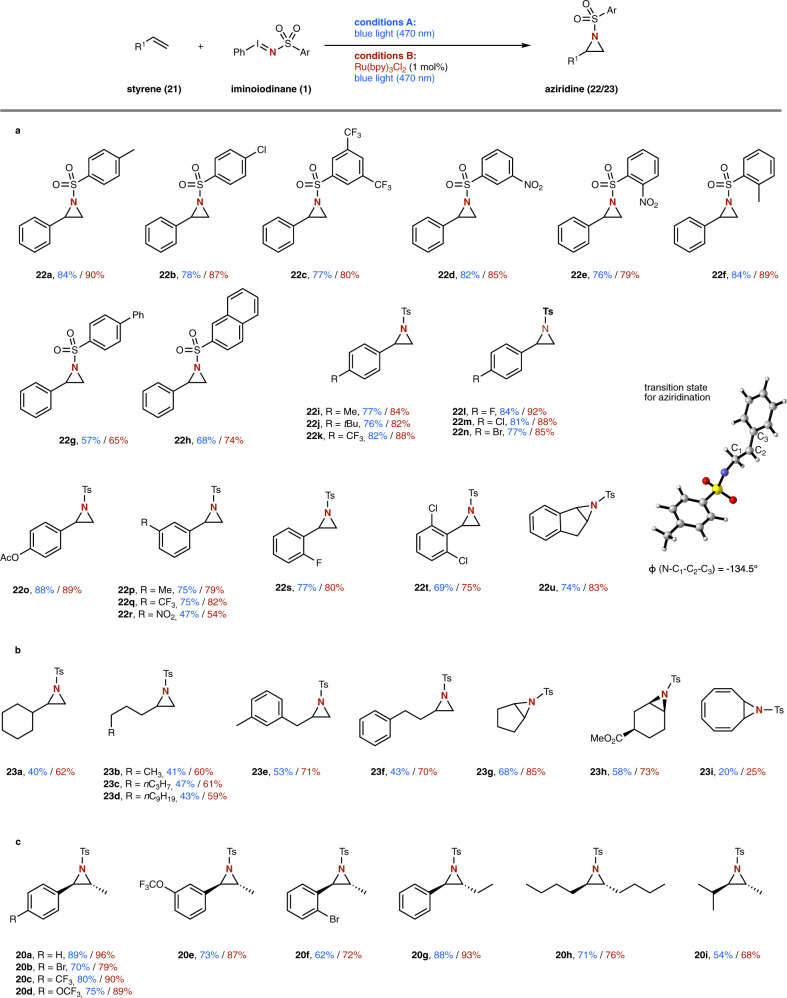
Scope of the photochemical and photocatalytic nitrene transfer reactions with styrenes and aliphatic olefins. **a** Evaluation of styrene derivatives. **b** Evaluation of aliphatic olefins **c** evaluation of 1,2-disubstituted olefins.

Finally, we embarked on the reaction of simple styrene derivatives under both photochemical and photocatalytic conditions (Fig. 6). In both cases, aziridination reaction was observed in high yields, with the photocatalytic reaction being slightly superior over the photochemical reaction. Different sulfonyl groups as well as a range of electronically and sterically distinct substituents at the aromatic ring of the styrene component proved compatible (**22a**–**t**). Most notably, even in the presence of two sterically demanding *ortho*-chloro substituents (**22t**) the desired aziridine product was obtained in good isolated yield. Further studies involved the reaction of cyclic olefins, such as indene, which smoothly reacted to the aziridine product (**22u**) in high isolated yield. An important difference in the reactivity of nitrene and nitrene radical anion was however observed in the reaction with aliphatic olefins (Fig. 6b). While only a poor yield was obtained under photochemical conditions, the photocatalysis approach via the nitrene radical anion proved superior and high yield in the aziridination reaction of aliphatic olefins was observed (**23a**–**i**). Further examples under investigation concerned the utilization of 1,2-disubstituted olefins. In this case, different aryl-alkyl and alkyl-alkyl disubstituted olefins smoothly underwent selective aziridination reaction to afford the *trans*-azridines **20a–i** in high yield.

The aziridination was then examined by DFT calculations, which suggest a similar pathways as in the aziridination reaction of α-methyl styrene for both photochemical and -catalytic conditions (cf. Fig. 3 and Supplementary Figs. 12, 13). However, a distinct difference in the conformational flexibility of the diradical intermediate **INT3**_**S**_ (cf. Fig. 4a, right) was observed. The absence of the α-methyl group renders the cyclization step to the aziridine much more favorable and it can proceed with a very low activation free energy of only 1.4 kcal mol^−1^. The direct comparison of the respective transition states show that the α-methyl group results in a conformational twist and that prevents efficient aziridination as it can be seen in the torsional angle around the N-C-C-C_Ar_ bond (Supplementary Fig. 11).

In summary, we herein report on amination reactions of olefins with iminoiodinane reagents under purely photochemical and photocatalytic reaction conditions. Depending on the reaction conditions, these amination reactions proceed via chemically distinct monovalent, nitrogen-based reactive intermediates that in turn lead to different reaction products in the reaction with α-methyl styrenes. While triplet nitrenes can be accessed via direct photolysis of iminioiodinanes and lead to C-H functionalization, a nitrene radical anion is formed under photocatalytic conditions and leads to aziridination reaction. We studied the formation, properties, and reactivity of triplet nitrene and nitrene radical anion using computational calculations to rationalize the observed reactivity in the reaction with olefins, which was further validated in control experiments. We conclude with studies on the application of these amination reactions in the reaction with a diverse set of olefins, ranging from α-substituted styrenes, styrenes, towards aliphatic olefins.

## Methods

### General method for the photochemical reactions

In an oven-dried tube (10 mL), equipped with a magnetic stirring bar, iminoiodinane (0.2 mmol, 1.0 equiv) and alkene (5.0 equiv) are dissolved in 2 mL DCM under air atmosphere. The reaction is stirred and irradiated with a 3 W LED lamp (5 cm distance) for 4 h. A cooling fan is used to maintain room temperature (25–28 °C). The product was obtained after column chromatography using *n*-hexane/EtOAc as eluent.

### General method for the photocatalytic reactions

In an oven-dried tube (10 mL), equipped with a magnetic stirring bar, iminoiodinane (0.2 mmol, 1.0 equiv), alkene (5.0 equiv), and catalyst (1 mol%) are dissolved in 2 mL DCM under air atmosphere. The reaction is stirred and irradiated with a 3 W LED lamp (5 cm distance) for 4 h. A cooling fan is used to maintain room temperature (25–28 °C). The product was obtained after column chromatography using *n*-hexane/EtOAc as eluent.

### Computational details

All calculations were performed using the Gaussian 16 series of programs^32^. All structures were optimized at the (U)M06-2X level^33^ of theory in combination with D3 dispersion corrections^34^, in which all atoms were described with the def2-SVP basis set^35^. Analytical frequency calculations were carried out at the same level of theory in order to confirm each stationary point as either an intermediate (no imaginary frequencies) or a transition state (only one imaginary frequency). Key transition-state structures were confirmed to connect corresponding reactants and products by intrinsic reaction coordinate (IRC) calculations^36,37^. The electronic energy was then refined using def2-TZVP basis set^35^ at the (U)M06-2X level on the optimized geometries in combination with D3 dispersion corrections. Solvation energies in dichloromethane (ε = 8.93; for acetonitrile: ε = 35.688) were evaluated by IEFPCM calculations with radii and non-electrostatic terms for SMD solvation model^38^ based on the optimized structures. Time-dependent (TD)-DFT calculations were carried out on the optimized structures of the PhINTs to obtain the absorption wavelength.

## Supplementary information

Supplementary Information

## Data Availability

The authors declare that the data supporting the findings of this study, including computational details, experimental details and compound characterization, are available within its Supplementary Information. All data available on request from the corresponding author.

## References

[1] Ciamician G (1912). The photochemistry of the future. Science.

[2] Gong J, Sumathy K, Qiao Q, Zhou Z (2017). Review on dye-sensitized solar cells (DSSCs): advanced techniques and research trends. Renew. Sustain. Energy Rev..

[3] Chatani S, Kloxin CJ, Bowman CN (2014). The power of light in polymer science: photochemical processes to manipulate polymer formation, structure, and properties. Polym. Chem..

[4] Chen Y, Topp EM (2019). Photolytic labeling and its applications in protein drug discovery and development. J. Pharm. Sci..

[5] Kärkäs MD, Porco Jr JA, Stephenson CRJ (2016). Photochemical approaches to complex chemotypes: applications in natural product synthesis. Chem. Rev..

[6] Nicholls TP, Leonori D, Bissember AC (2016). Applications of visible light photoredox catalysis to the synthesis of natural products and related compounds. Nat. Prod. Rep..

[7] Romero NA, Nicewicz DA (2016). Organic photoredox catalysis. Chem. Rev..

[8] Marzo L, Pagire SK, Reiser O, König B (2018). Visible-light photocatalysis: does it make a difference in organic synthesis?. Angew. Chem. Int. Ed..

[9] Poplata S, Tröster A, Zou Y-Q, Bach T (2016). Recent advances in the synthesis of cyclobutanes by olefin [2+2] photocycloaddition reactions. Chem. Rev..

[10] Cambié D, Bottecchia C, Straathof NJW, Hessel V, Noel T (2016). Applications of continuous-flow photochemistry in organic synthesis, material science, and water treatment. Chem. Rev..

[11] Yang Z, Stivanin ML, Jurberg ID, Koenigs RM (2020). Visible light-promoted reactions with diazo compounds: a mild and practical strategy towards free carbene intermediates. Chem. Soc. Rev..

[12] Jurberg ID, Davies HML (2018). Blue light-promoted photolysis of aryldiazoacetates. Chem. Sci..

[13] Hommelsheim R, Guo Y, Yang Z, Empel C, Koenigs RM (2019). Blue-light-induced carbene-transfer reactions of diazoalkanes. Angew. Chem. Int. Ed..

[14] Xiao T, Mei M, He Y, Zhou L (2018). Blue light-promoted cross-coupling of aryldiazoacetates and diazocarbonyl compounds. Chem. Commun..

[15] Dequierez G, Pons V, Dauban P (2012). Nitrene chemistry in organic synthesis: still in its infancy?. Angew. Chem. Int. Ed..

[16] Plietker B, Röske A (2019). Recent advances in Fe-catalyzed C–H aminations using azides as nitrene precursors. Catal. Sci. Technol..

[17] Kuijpers PF, van der Vlugt JI, Schneider S, de Bruin B (2017). Nitrene radical intermediates in catalytic synthesis. Chem. Eur. J..

[18] Lu H, Zhang XP (2011). Catalytic C–H functionalization by metalloporphyrins: recent developments and future directions. Chem. Soc. Rev..

[19] Singh R, Mukherjee A (2019). Metalloporphyrin catalyzed C–H amination. ACS Catal..

[20] Ochiai M, Miyamoto K, Kaneaki T, Hayashi S, Nakanashi W (2011). Highly regioselective amination of unactivated alkanes by hypervalent sulfonylimino-λ^3^-bromane. Science.

[21] Platz, M. S. in *Reactive Intermediate Chemistry* (eds. Moss, R. A., Platz, M. S. & Jones, M.Jr), 501–559 (Wiley-VCH, 2005).

[22] Doyle, M. P. in *Reactive Intermediate Chemistry* (eds. Moss, R. A., Platz, M. S. & Jones, M.Jr), 561–592 (Wiley-VCH, 2005).

[23] McDonald RN, Chowdhury AK (1980). Hypovalent radicals. 8. Identification of nucleophilic 1,2- and 1,4-addition processes with alpha-beta-unsaturated molecules in the gas phase. J. Am. Chem. Soc..

[24] Das A, Chen Y-S, Reibenspies JH, Powers DC (2019). Characterization of a reactive Rh_2_ nitrenoid by crystalline matrix isolation. J. Am. Chem. Soc..

[25] Du Y-D, Zhou C-Y, To W-P, Wang H-X, Che C-M (2020). Iron porphyrin catalysed light driven C–H bond amination and alkene aziridination with organic azides. Chem. Sci..

[26] Hernandez-Guerra D (2019). Photochemical C−H amination of ethers and geminal difunctionalization reactions in one pot. Angew. Chem. Int. Ed..

[27] Lebel H, Piras H, Borduy M (2016). Iron-catalyzed amination of sulfides and sulfoxides with azides in photochemical continuous flow synthesis. ACS Catal..

[28] Tian X, Song L, Hashmi ASK (2020). Synthesis of carbazoles and related heterocycles from sulfilimines by intramolecular C−H aminations. Angew. Chem. Int. Ed..

[29] Kobayashi Y, Masakado S, Takemoto Y (2018). Photoactivated N-acyliminoiodinanes applied to amination: an ortho-methoxymethyl group stabilizes reactive precursors. Angew. Chem. Int. Ed..

[30] Guo Y, Pei C, Jana S, Koenigs RM (2021). Synthesis of trifluoromethylated aziridines via photocatalytic amination reactions. ACS Catal..

[31] Shainyan BA, Kuzmin AV (2014). Sulfonyl nitrenes from different sources: computational study of formation and transformations. J. Phys. Org. Chem..

[32] Frisch, M. J. et al. *Gaussian 16, Revision C.01*, (Gaussian, Inc., Wallingford CT, 2019).

[33] Zhao Y, Truhlar DG (2008). The M06 suite of density functionals for main group thermochemistry, thermochemical kinetics, noncovalent interactions, excited states, and transition elements: two new functionals and systematic testing of four M06-class functionals and 12 other functionals. Theor. Chem. Acc..

[34] Grimme S, Antony J, Ehrlich S, Krieg H (2010). A consistent and accurate *ab* initio parametrization of density functional dispersion correction (DFT-D) for the 94 elements H-Pu. J. Chem. Phys..

[35] Weigend F, Ahlrichs R (2005). Balanced basis sets of split valence, triple zeta valence and quadruple zeta valence quality for H to Rn: Design and assessment of accuracy. Phys. Chem. Chem. Phys..

[36] Fukui K (1970). Formulation of the reaction coordinate. J. Phys. Chem..

[37] Fukui K (1981). The path of chemical reactions-the IRC approach. Acc. Chem. Res..

[38] Marenich AV, Cramer CJ, Truhlar DG (2009). Universal solvation model based on solute electron density and on a continuum model of the solvent defined by the bulk dielectric constant and atomic surface tensions. J. Phys. Chem. B.

