# Supplementary material for: A combined experimental and theoretical study on the reactivity of nitrenes and nitrene radical anions
Source: Nat Commun. 2022 Jan 10;13:86. doi: 10.1038/s41467-021-27687-6 (PMC8748618; doi:10.1038/s41467-021-27687-6)
Supplement: Supplementary file 1 — Supplementary Information [file 41467_2021_27687_MOESM1_ESM.pdf]

Supplementary Information

A combined experimental and theoretical study on the reactivity of nitrenes and nitrene radical anions

Yujing Guo, Chao Pei and Rene M. Koenigs*

Table of Contents

General Information	S1
UV-Vis Experiments	S2
Reaction Optimization	S3
Control Experiments	S4
General Procedures	S7
Physical Data	S8
DFT Calculations	S50
Spectra	S78
Supplementary References	S180

General Information

Unless otherwise noted, all commercially available compounds were used as provided without further purification. Chemicals used in this manuscript were purchased from Sigma Aldrich, Alfa Aesar, Fluorochem and Carl Roth. Solvents used in reactions were p.A. grade. Solvents for chromatography were technical grade and distilled prior to use. Analytical thin-layer chromatography (TLC) was performed on Macherey-Nagel silica gel aluminium plates with F-254 indicator, visualised by irradiation with UV light. Column chromatography was performed using silica gel Merck 60 (particle size 0.063 – 0.2 mm). Solvent mixtures are understood as volume/volume.

^{1}H-NMR, ^{19}F-NMR and ^{13}C-NMR were recorded on a Varian AV600/AV400 or an Agilent DD2 400 NMR spectrometer in $CDCl_3$. Data are reported in the following order: chemical shift (δ) in ppm; multiplicities are indicated br (broadened singlet), s (singlet), d (doublet), t (triplet), q (quartet), m (multiplet); coupling constants (*J*) are in Hertz (Hz).

HRMS data were recorded on a ThermoFisher Scientific LTQ Orbitrap XL using ESI ionization or on a Finnigan MAT 95 using EI ionization at 70 eV.

IR spectra were recorded on a Perkin Elmer-100 spectrometer and are reported in terms of frequency of absorption (cm^{-1}).

UV-Vis spectra were recorded on a Shimadzu UV-2600 UV-VIS spectrophotometer.

Elemental analysis was performed on an Elementar VarioEL instrument.

Iodinanes used in this manuscript were synthesized according to the literature procedure.[1,2]

LEDs used in this manuscript were purchased from Conrad Electronics.

High Power LED-Module, 3 W, 30 lm, 30 °, 470 nm, art.nr. 180745 – 62.

Reactions were irradiated from 5 cm, temperature was set to ambient and cooling was realized with a fan.

UV-Vis Experiments

Compounds **A**-**C** and **E** were measured in a 0.02 M solution in methanol at room temperature, compound **D** was measured in a 0.01 M solution in methanol at room temperature. All spectra were directly recorded after setting up the solutions.

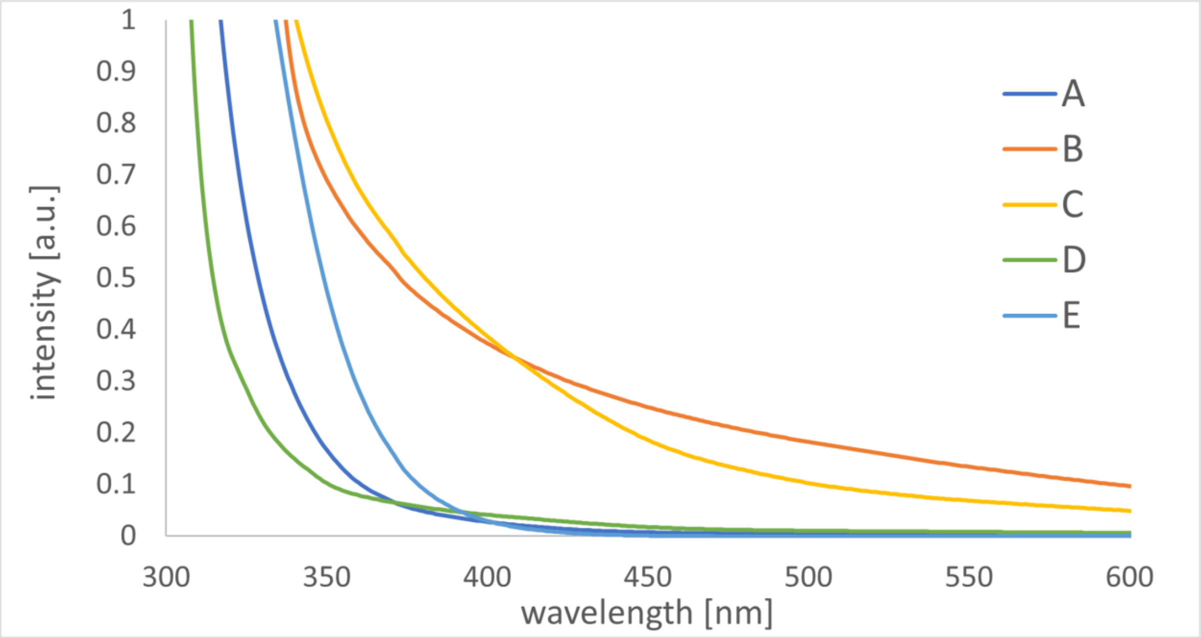

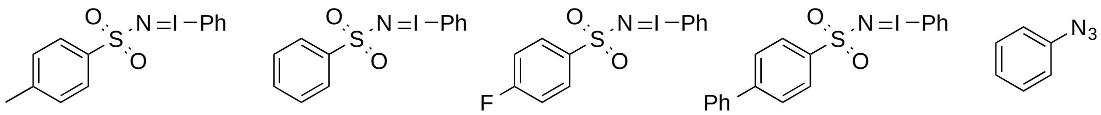

A **B** **C** **D** **E**

Supplementary Figure 1. UV-Vis spectra of different iodonanes and phenylazide as reference.

Reaction Optimization

Supplementary Table 1. Reaction Optimization.[a]

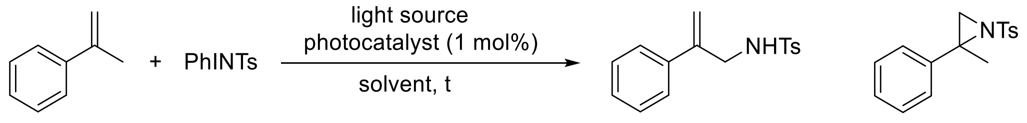

12a **1a** **10a** **11a**

entry	light	Photocatalyst	solvent	12a:1a	%Yield (10a) / (11a)
1	385 nm	-	DCM	5 : 1	22 / -
2	470 nm	-	DCM	5 : 1	70 / -
3	530 nm	-	DCM	5 : 1	40 / -
4	white light	-	DCM	5 : 1	29 / -
5	in the dark	-	DCM	5 : 1	n.r. / -
6	470 nm	$Ru(bpy)_3Cl_2{\bullet}6H_2O$	DCM	5 : 1	- / 80
7	470 nm	4-CzIPN	DCM	5 : 1	51 / -
8	470 nm	$Ir(ppy)_3$	DCM	5 : 1	27 / -
9	470 nm	$Ru(bpz)_3(PF_6)_2$	DCM	5 : 1	35 / -
10	470 nm	$(Ir[(dF(CF_3)ppy]_2(dtbpy)PF_6$	DCM	5 : 1	30 / -
11	470 nm	Eosin Y	DCM	5 : 1	34 / 45
12	470 nm	$Ru(bpy)_3Cl_2{\bullet}6H_2O$	DCM	10 :1	- / 81
13	470 nm	$Ru(bpy)_3Cl_2{\bullet}6H_2O$	DCM	3 :1	- / 71
14	470 nm	$Ru(bpy)_3Cl_2{\bullet}6H_2O$	DCE	5 : 1	- / 69
15	470 nm	$Ru(bpy)_3Cl_2{\bullet}6H_2O$	$CHCl_3$	5 : 1	- / 66
16	470 nm	$Ru(bpy)_3Cl_2{\bullet}6H_2O$	EA	5 : 1	- / 20
17	470 nm	$Ru(bpy)_3Cl_2{\bullet}6H_2O$	THF	5 : 1	- / 30
18	470 nm	$Ru(bpy)_3Cl_2{\bullet}6H_2O$	CH_3NO_2	5 : 1	- / n.r.
19	470 nm	$Ru(bpy)_3Cl_2{\bullet}6H_2O$	MeCN	5 : 1	- / 61
20[b]	470 nm	$Ru(bpy)_3Cl_2{\bullet}6H_2O$	DCM	5 : 1	- / 80
21[c]	470 nm	$Ru(bpy)_3Cl_2{\bullet}6H_2O$	DCM	5 : 1	- / 80
22[d]	470 nm	$Ru(bpy)_3Cl_2{\bullet}6H_2O$	DCM	5 : 1	- / 72

[a]Reaction conditions: α-methyl styrene **12a**, iminoiodinane **1a** and the respective photocatalyst (1 mol%) were dissolved in 2.0 mL Solvent under air atmosphere. The mixture was irradiated with the light source indicated at room temperature. n.r. = no reaction. [b]8 h; [c]4 h; [d]2 h.

Control experiments

Supplementary Table 2. Control experiments of photochemical reactions[a]

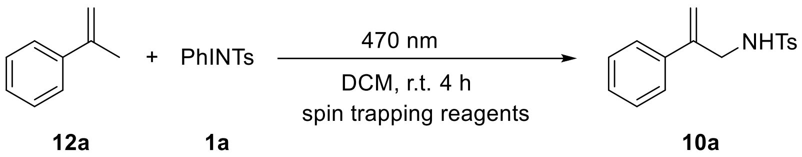

spin trapping reagents	%yield (10a)
1 equiv of 4-nitrotoluene	59
1 equiv of 4-methyl anisole	43
1 equiv of triethylamine / DABCO	n.r. / n.r.
1 equiv of dihydropyridine	15
1 equiv of MeOH / H_2O	66 / 45
0.1 / 0.5 / 1.0 equiv of TEMPO	40 / n.r. / n.r.
1.0 equiv of DMPO / DNP	n.r. / n.r.

[a]Reaction conditions: **12a** (1 mmol, 5 eq.), **1a** (0.2 mmol) and spin trapping reagents in 2.0 mL DCM under air atmosphere. The mixture was irradiated with the light source indicated for 4 h at room temperature. n.r. = no reaction. Dihydropyridine = Diethyl 1,4-dihydro-2,6-dimethyl-3,5-pyridinedi-carboxylate. TEMPO = (2,2,6,6-Tetramethylpiperidin-1-yl)oxyl. DMPO = 5,5-Dimethyl-1-pyrrolin-N-oxid. DNP = 2,4-Dinitrophenol.

Supplementary Table 3. Control experiments of photocatalytic reactions[a]

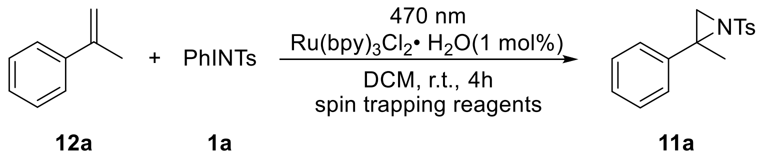

spin trapping reagents	%yield (11a)
1 equiv of 4-nitrotoluene	71
1 equiv of 4-methyl anisole	65
1 equiv of triethylamine / DABCO	18 / n.r.
1 equiv of dihydropyridine	10
1 equiv of MeOH / H_2O	64 / 67
0.1 / 0.5 / 1.0 equiv of TEMPO	51 / 25 / n.r.
1.0 equiv of DMPO / DNP	n.r. / n.r.

[a]Reaction conditions: **12a** (1 mmol, 5 eq.), **1a** (0.2 mmol), $Ru(bpy)_3Cl_2•H_2O$ (1 mol%) and spin trapping reagents in 2.0 mL DCM under air atmosphere. The mixture was irradiated with the light source indicated for 4 h at room temperature. n.r. = no reaction. Dihydropyridine = Diethyl 1,4-dihydro-2,6-dimethyl-3,5-pyridinedi-carboxylate. TEMPO = (2,2,6,6-Tetramethylpiperidin-1-yl)oxyl. DMPO = 5,5-Dimethyl-1-pyrrolin-N-oxid. DNP = 2,4-Dinitrophenol.

KIE Experiments

KIE experiment (intermolecular competition)

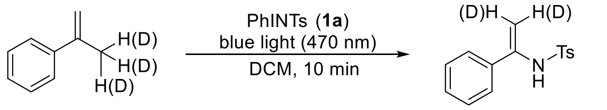

12a / 12a-*d*$_3$ (86%D) **10a / 10a-*d*$_2$ = 1.08**

Intermolecular competition experiment: α-Methylstyrene **12a-*d*$_3$** (86% D) (0.588 mmol, 71.1 mg), **12a** (0.412 mmol, 48.6 mg) and **1a** were added to an oven-dried tube (10 mL), equipped with a magnetic stirring bar, DCM (2.0 mL) was added. The reaction is stirred and irradiated with a 3 W LED lamp (5 cm distance) for 10min. A cooling fan is used to maintain room temperature (25-28 °C). The crude product was obtained after short column chromatography using *n*-hexane / EtOAc as eluent. The ratio of **10a**/**10a-*d*$_2$** = 1.08 was determined obtained.

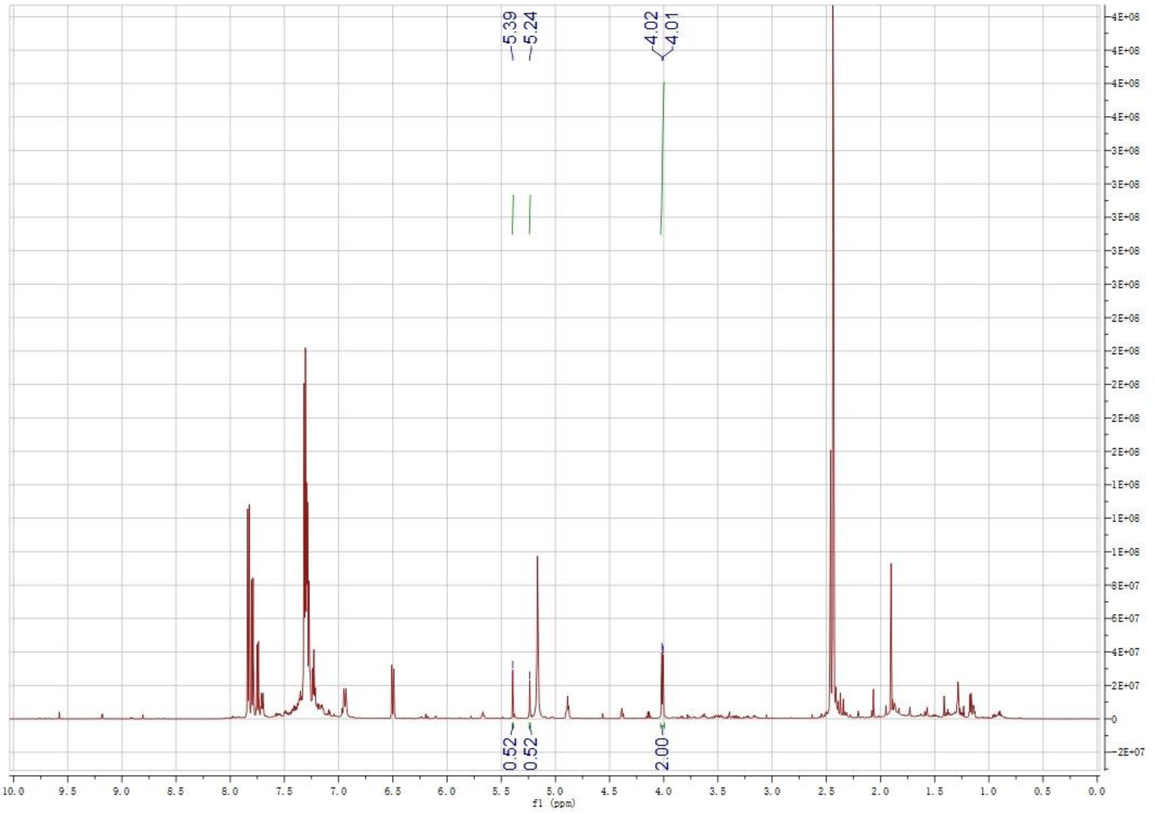

Supplementary Figure 2. The crude NMR of intermolecular competition experiment

KIE experiment (parallel experiments)

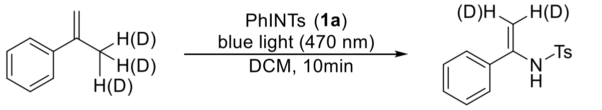

12a / 12a-*d*$_3$ (86%D) **10a / 10a-*d*$_2$ = 1.23**

Parallel experiments: α-Methylstyrene **12a** (1.0 mmol, 118.1 mg) and **1a** were added to an oven-dried tube (10 mL), equipped with a magnetic stirring bar, DCM (2.0 mL) was added. In another reaction vessel, **12a-*d*3** (86% D) (1.0 mmol, 121.1 mg) was used instead of **12a**. The two reactions are stirred and irradiated with a 3 W LED lamp (5 cm distance) for 10min. A cooling fan is used to maintain room temperature (25-28 °C). The solvent was removed in vacuo to leave a crude mixture. The ratio of **10a**/**10a-*d*2** = 1.23 was determined by ^{1}H NMR using mesitylene (14 μL) as the internal standard and going through mathematical conversion.

KIE experiment (reaction kinetics)

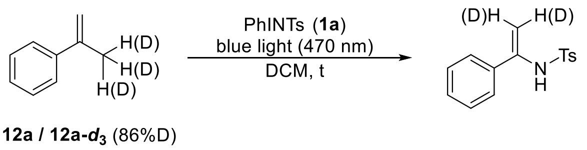

α-Methylstyrene **12a** (1.0 mmol, 118.1 mg) and **1a** were added to an oven-dried tube (10 mL), equipped with a magnetic stirring bar, DCM (2.0 mL) was added. In another reaction vessel, **12a-*d*3** (86% D) (1.0 mmol, 121.1 mg) was used instead of **12a**. The reactions are stirred and irradiated with a 3 W LED lamp (5 cm distance) for 2 min, 4min, 6 min, 8min, 10min. A cooling fan is used to maintain room temperature (25-28 °C). The solvent was removed in vacuo to leave a crude mixture. The yield was determined by ^{1}H NMR using mesitylene (14 μL) as the internal standard and going through mathematical conversion.

t (min) / Yield(%)	2	4	6	8	10
**10a**	10	16	21	27	32
**10a-*d*2**	5	10	15	22	26

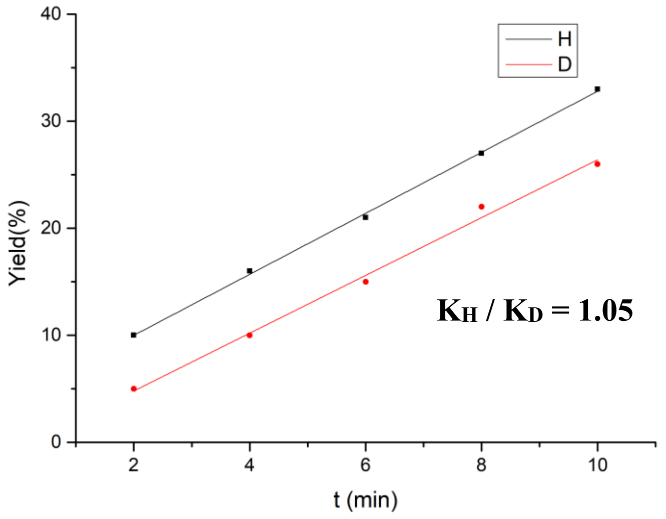

Supplementary Figure 3. KIE experiment (reaction kinetics)

General Procedures

Procedure for synthesis of iminoiodinane (1a)

Compound **1a** was prepared according to the literature procedure.[1,2] *p*-Toluenesulphonamide (10.26 g), potassium hydroxide (8.40 g) and methanol (240 ml) were stirred in a flask in an ice bath. Iodobenzene diacetate (19.20 g) was added to the stirred mixture and the resulting solution was stirred at room temperature overnight. Pour into a large excess of iced water to the reaction mixture and stirred for 1 h. A light yellow coloured solid precipitated on standing overnight. The solid was isolated by filtration. Several portions of ether were used to wash the solid. The solid was recrystallization in methanol. Yield = 65%.

General Procedure for Photochemical reactions – GP1

In an oven-dried tube (10 mL), equipped with a magnetic stirring bar, iminoiodinane (0.2 mmol, 1.0 eq.) and alkene (5.0 eq.) are dissolved in 2 mL DCM under air atmosphere. The reaction is stirred and irradiated with a 3 W LED lamp (5 cm distance) for 4 h. A cooling fan is used to maintain room temperature (25-28 °C). The product was obtained after column chromatography using *n*-hexane / EtOAc as eluent.

General Procedure for Photocatalytic reactions – GP2

In an oven-dried tube (10 mL), equipped with a magnetic stirring bar, iminoiodinane (0.2 mmol, 1.0 eq.), alkene (5.0 eq.), and catalyst (1 mol%) are dissolved in 2 mL DCM under air atmosphere. The reaction is stirred and irradiated with a 3 W LED lamp (5 cm distance) for 4 h. A cooling fan is used to maintain room temperature (25-28 °C). The product was obtained after column chromatography using *n*-hexane / EtOAc as eluent.

Physical Data

N-(4-Methylphenylsulfonyl)imino-phenyl-λ3-iodane (1a)

^{1}H NMR (400 MHz, DMSO-d_6): δ = 7.67 – 7.61 (m, 2H), 7.48 – 7.38 (m, 3H), 7.26 (t, *J* = 7.8 Hz, 2H), 7.02 (d, *J* = 7.9 Hz, 2H), 2.23 (s, 3H) ppm.
^{13}C NMR (101 MHz, DMSO-d_6): δ = 142.6, 140.5, 133.6, 130.8, 130.5, 129.0, 126.5, 117.6, 21.2 ppm.
elemental analysis: calcd (%) for $C_{13}H_{12}INO_2S$: C 41.84, H 3.24, N 3.75; found: C 41.71, H 3.25, N 3.69.

4-Methyl-*N*-(2-phenylallyl)benzenesulfonamide (10a)

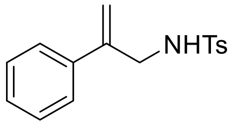

Compound **10a** was prepared according to the general procedure **GP1** and was obtained after column chromatography (*n*-hexane : ethyl acetate 4:1→1:1) as a colorless oil in 70% yield (40 mg).
^{1}H NMR (400 MHz, $CDCl_3$): δ = 7.70 (d, *J* = 8.3 Hz, 2H), 7.31 – 7.25 (m, 5H), 7.24 – 7.19 (m, 2H), 5.36 (d, *J* = 0.4 Hz, 1H), 5.19 (d, *J* = 0.6 Hz, 1H), 4.42 (t, *J* = 6.0 Hz, 1H), 4.09 – 3.85 (m, 2H), 2.43 (s, 3H) ppm.
^{13}C NMR (101 MHz, $CDCl_3$): δ = 143.4, 142.8, 137.7, 136.6, 129.6, 128.5, 128.1, 127.2, 126.0, 115.1, 47.0, 21.5 ppm.
HRMS (ESI): mass found: 288.10504, calculated mass for $C_{16}H_{18}NO_2S^+$: 288.10528.
IR (KBr): 3512, 3282, 3059, 2927, 2864, 2317, 2166, 1971, 1916, 1811, 1727, 1598, 1495, 1446, 1328, 1157, 1087, 1028, 978, 904, 814, 763, 699, 667 cm^{-1}.

4-Methyl-*N*-(2-phenylallyl-3,3-d2)benzenesulfonamide (10a-*d₂*)

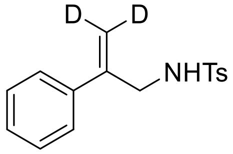

Compound **10a-*d₂*** was prepared according to the general procedure **GP1** and was obtained after column chromatography (*n*-hexane : ethyl acetate 4:1→1:1) as a colorless oil in 70% yield (40 mg), deuterium content: 86%.
^{1}H NMR (600 MHz, $CDCl_3$): δ = 7.74 (d, *J* = 8.2 Hz, 2H), 7.35 – 7.29 (m, 5H), 7.26 (dd, *J* = 6.8, 3.0 Hz, 2H), 5.39 (d, *J* = 8.7 Hz, 0.14H), 5.22 (d, *J* = 8.4 Hz, 0.14H), 4.48 (s, 1H), 4.03 (d, *J* = 6.2 Hz, 2H), 2.47 (s, 3H) ppm.
^{13}C NMR (151 MHz, $CDCl_3$): δ = 143.5, 142.7, 137.7, 136.7, 129.7, 128.5, 128.2, 127.2, 126.0, 122.9, 46.9, 21.5 ppm.
HRMS (ESI): mass found: 290.11750, calculated mass for $C_{16}H_{16}D_2NO_2S^+$: 290.11783.

IR (KBr): 3858, 3509, 3282,3058, 2921, 2867, 2682, 2328, 2194, 2164, 2078, 2028, 1911, 1731, 1597, 1495, 1444, 1326, 1157, 1091, 814, 763, 732, 697, 666 cm^{-1}.

4-Methyl-*N*-(2-(*p*-tolyl)allyl)benzenesulfonamide (10b)

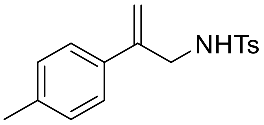

Compound **10b** was prepared according to the general procedure **GP1** and was obtained after column chromatography (*n*-hexane : ethyl acetate 9:1→4:1) as a colorless oil in 57% yield (34 mg).
^{1}H NMR (600 MHz, $CDCl_3$): δ = 7.73 (d, *J* = 8.0 Hz, 2H), 7.31 (d, *J* = 8.0 Hz, 2H), 7.14 (d, *J* = 7.8 Hz, 2H), 7.10 (d, *J* = 8.0 Hz, 2H), 5.35 (s, 1H), 5.16 (s, 1H), 4.41 (t, *J* = 6.2 Hz, 1H), 3.99 (d, *J* = 6.1 Hz, 2H), 2.46 (s, 3H), 2.35 (s, 3H) ppm.
^{13}C NMR (151 MHz, $CDCl_3$): δ = 143.5, 142.6, 138.1, 136.7, 134.8, 129.6, 129.2, 127.2, 125.9, 114.3, 47.1, 21.5, 21.1 ppm.
HRMS (ESI): Mass found: 302.12135, calculated mass for $C_{17}H_{20}NO_2S^+$: 302.12093.
IR (KBr): 3510, 3283, 2925, 2866, 2732, 2161, 2032, 1912, 1726, 1679, 1600, 1513, 1449, 1407, 1329, 1158, 1088, 976, 901, 814, 762, 707, 665 cm^{-1}.

***N*-(2-(4-Butylphenyl)allyl)-4-methylbenzenesulfonamide (10c)**

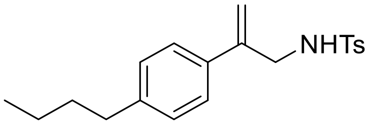

Compound **10c** was prepared according to the general procedure **GP1** and was obtained after column chromatography (*n*-hexane : ethyl acetate 9:1→4:1) as a colorless oil in 52% yield (36 mg).
^{1}H NMR (600 MHz, $CDCl_3$): δ =7.84 – 7.69 (m, 2H), 7.32 (d, *J* = 7.9 Hz, 2H), 7.19 – 7.16 (m, 2H), 7.12 (d, *J* = 8.3 Hz, 2H), 5.37 (s, 1H), 5.17 (d, J = 0.7 Hz, 1H), 4.48 (t, *J* = 6.0 Hz, 1H), 4.01 (dd, *J* = 6.2, 0.4 Hz, 2H), 2.61 (dd, *J* = 9.5, 6.0 Hz, 2H), 2.47 (s, 3H), 1.69 – 1.44 (m, 2H), 1.44 – 1.28 (m, 2H), 0.96 (t, *J* = 7.4 Hz, 3H) ppm.
^{13}C NMR (151 MHz, $CDCl_3$): δ = 143.4, 143.1, 142.6, 136.7, 135.0, 129.6, 128.6, 127.2, 125.9, 114.3, 47.0, 35.2, 33.5, 22.3, 21.5, 13.9 ppm.
HRMS (ESI): mass found: 344.16822, calculated mass for $C_{20}H_{26}NO_2S^+$: 344.16788.
IR (KBr): 3866, 3524, 3272, 3029, 2927, 2861, 2678, 2478, 2321, 2083, 1996, 1910, 1805, 1731, 1678, 1598, 1512, 1456, 1405, 1314, 1239, 1137, 1085, 1046, 1019, 896, 814, 774, 708, 671 cm^{-1}.

***N*-(2-(4-Fluorophenyl)allyl)-4-methylbenzenesulfonamide (10d)**

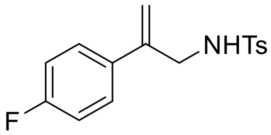

Compound **10d** was prepared according to the general procedure **GP1** and was obtained after column chromatography (*n*-hexane : ethyl acetate 9:1→4:1) as a white solid in 62% yield (38 mg).

^{1}H NMR (600 MHz, Chloroform-*d*): δ = 7.64 (d, *J* = 8.0 Hz, 2H), 7.23 (d, *J* = 8.0 Hz, 2H), 7.16 (dd, *J* = 8.5, 5.4 Hz, 2H), 6.90 (t, *J* = 8.5 Hz, 2H), 5.26 (s, 1H), 5.12 (s, 1H), 4.37 (t, *J* = 6.3 Hz, 1H), 3.90 (d, *J* = 6.3 Hz, 2H), 2.38 (s, 3H) ppm.

^{13}C NMR (151 MHz, $CDCl_3$): δ = 162.6 (d, *J* = 247.2 Hz), 143.6, 141.9, 136.7, 133.8 (d, *J* = 3.3 Hz), 129.7, 127.8 (d, *J* = 7.8 Hz), 127.2, 115.4 (d, *J* = 21.8 Hz), 115.2, 47.2, 21.5 ppm.

^{19}F NMR (565 MHz, $CDCl_3$): δ = -113.61 – -113.76 ppm.

HRMS (ESI): mass found: 306.09707, calculated mass for $C_{16}H_{17}NFO_2S^+$: 306.09586.

IR (KBr): 3514, 3284, 3069, 2928, 2869, 2733, 2161, 2029, 1978, 1898, 1726, 1600, 1509, 1452, 1327, 1229, 1157, 1090, 1014, 976, 910, 836, 816, 766, 665 cm^{-1}.

***N*-(2-(4-Chlorophenyl)allyl)-4-methylbenzenesulfonamide (10e)**

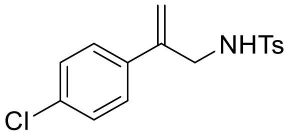

Compound **10e** was prepared according to the general procedure **GP1** and was obtained after column chromatography (*n*-hexane : ethyl acetate 9:1→4:1) as a colorless oil in 53% yield (34 mg).

^{1}H NMR (600 MHz, $CDCl_3$): δ = 7.72 (d, *J* = 7.9 Hz, 2H), 7.31 (d, *J* = 8.0 Hz, 2H), 7.27 – 7.23 (m, 2H), 7.19 (d, *J* = 8.3 Hz, 2H), 5.39 (s, 1H), 5.24 (s, 1H), 4.44 (t, *J* = 6.4 Hz, 1H), 3.98 (d, *J* = 6.3 Hz, 2H), 2.46 (s, 3H) ppm.

^{13}C NMR (151 MHz, $CDCl_3$): δ = 143.6, 141.9, 136.7, 136.2, 134.1, 129.7, 128.7, 127.4, 127.2, 115.8, 47.0, 21.5 ppm.

HRMS (ESI): Mass found: 344.04806, calculated mass for $C_{16}H_{16}ClNNaO_2S^+$: 344.04825.

IR (KBr): 3852, 3507, 3254, 3063, 2865, 2672, 2324, 2159, 1981, 1924, 1843, 1732, 1630, 1595, 1492, 1427, 1398, 1323, 1224, 1158, 1089, 1011, 889, 832, 729, 765, 729, 706, 664 cm^{-1}.

***N*-(2-(4-Bromophenyl)allyl)-4-methylbenzenesulfonamide (10f)**

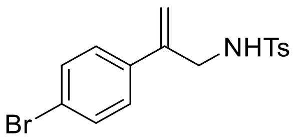

Compound **10f** was prepared according to the general procedure **GP1** and was obtained after column chromatography (*n*-hexane : ethyl acetate 9:1→4:1) as a white solid in 53% yield (39 mg).

^{1}H NMR (400 MHz, $CDCl_3$): δ = 7.68 (d, *J* = 8.2 Hz, 2H), 7.38 (d, *J* = 8.5 Hz, 2H), 7.28 (d, *J* = 8.0 Hz, 2H), 7.10 (d, *J* = 8.6 Hz, 2H), 5.36 (s, 1H), 5.21 (s, 1H), 4.45 (t, *J* = 6.1 Hz, 1H), 3.95 (d, *J* = 6.3 Hz, 2H), 2.43 (s, 3H) ppm.

^{13}C NMR (101 MHz, $CDCl_3$): δ = 143.6, 141.9, 136.6, 131.5, 129.6, 127.6, 127.1, 122.2, 115.9, 114.9, 46.9, 21.5 ppm.

HRMS (ESI): mass found: 366.01623, calculated mass for $C_{16}H_{17}BrNO_2S^+$: 366.01579.

IR (KBr): 3851, 3511, 3259, 3060, 2924, 2863, 2663, 2321, 2161, 2013, 1911, 1849, 1722, 1683, 1633, 1590, 1489, 1427, 1396, 1324, 1156, 1073, 1006, 919, 891, 819, 736, 705, 662 cm^{-1}.

***N*-(2-(4-Methoxyphenyl)allyl)-4-methylbenzenesulfonamide (10g)**

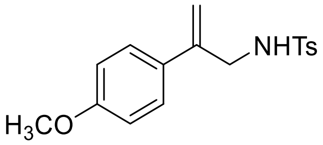

Compound **10g** was prepared according to the general procedure **GP1** and was obtained after column chromatography (*n*-hexane : ethyl acetate 9:1→4:1) as a colorless oil in 49% yield (31 mg).

^{1}H NMR (600 MHz, $CDCl_3$): δ = 7.76 – 7.69 (m, 2H), 7.31 (d, *J* = 7.8 Hz, 2H), 7.21 – 7.18 (m, 2H), 6.85 – 6.80 (m, 2H), 5.30 (s, 1H), 5.11 (s, 1H), 4.40 (t, *J* = 6.1 Hz, 1H), 3.98 (d, *J* = 6.2 Hz, 2H), 3.81 (s, 3H), 2.45 (s, 3H) ppm.

^{13}C NMR (151 MHz, $CDCl_3$): δ = 159.6, 143.5, 142.1, 136.7, 130.1, 129.6, 127.2, 113.9, 113.6, 55.3, 47.2, 21.5 ppm. (*one carbon signal is missing, due to the overlap)

HRMS (ESI): mass found: 340.09736, calculated mass for $C_{17}H_{19}NO_3SNa^+$: 340.09779.

IR (KBr): 3509, 3276, 2932, 2838, 2321, 2159, 2039, 1991, 1918, 1718, 1673, 1604, 1510, 1456, 1321, 1247, 1156, 1091, 1030, 899, 830, 746, 664 cm^{-1}.

***N*-(2-(4-Cyanophenyl)allyl)-4-methylbenzenesulfonamide (10h)**

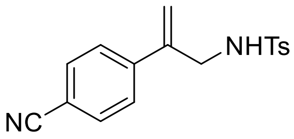

Compound **10h** was prepared according to the general procedure **GP1** and was obtained after column chromatography (*n*-hexane : ethyl acetate 9:1→4:1) as a colorless oil in 71% yield (44 mg).

^{1}H NMR (600 MHz, $CDCl_3$): δ 7.71 (d, *J* = 8.0 Hz, 2H), 7.57 (d, *J* = 8.2 Hz, 2H), 7.40 – 7.37 (m, 2H), 7.30 (d, *J* = 8.0 Hz, 2H), 5.51 (s, 1H), 5.38 (s, 1H), 4.53 (t, *J* = 6.4 Hz, 1H), 4.00 (d, *J* = 6.5 Hz, 2H), 2.45 (s, 3H) ppm.

^{13}C NMR (151 MHz, $CDCl_3$): δ = 143.8, 142.3, 141.7, 136.6, 132.3, 129.8, 127.1, 126.8, 118.5, 118.3, 111.7, 46.8, 21.5 ppm.

HRMS (ESI): Mass found: 335.08276, calculated mass for $C_{17}H_{16}N_2NaO_2S^+$: 335.08247.

IR (KBr): 3357, 3262, 3051, 2923, 2856, 2593, 2316, 2228, 2088, 1995, 1921, 1733, 1598, 1527, 1426, 1392, 1300, 1154, 1094, 1050, 903, 867, 844, 814, 679 cm^{-1}.

***N*-(2-(3-Fluorophenyl)allyl)-4-methylbenzenesulfonamide (10i)**

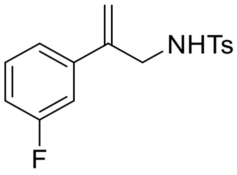

Compound **10i** was prepared according to the general procedure **GP1** and was obtained after column chromatography (*n*-hexane : ethyl acetate 9:1→4:1) as a colorless oil in 57% yield (35 mg).

^{1}H NMR (600 MHz, $CDCl_3$): δ = 7.64 (d, *J* = 8.2 Hz, 2H), 7.22 (d, *J* = 8.1 Hz, 2H), 7.20 – 7.13 (m, 1H), 6.96 (d, *J* = 7.8 Hz, 1H), 6.90 (td, *J* = 8.4, 2.5 Hz, 1H), 6.84 – 6.76 (m, 1H), 5.32 (s, 1H), 5.19 (s, 1H), 4.44 (t, *J* = 6.1 Hz, 1H), 3.91 (d, *J* = 6.2 Hz, 2H), 2.37 (s, 3H) ppm.

^{13}C NMR (151 MHz, $CDCl_3$): δ = 162.8 (d, *J* = 246.3 Hz), 143.69, 141.9, 140.1 (d, *J* = 7.6 Hz), 136.6, 130.0 (d, *J* = 8.2 Hz), 129.7, 127.2, 121.7 (d, *J* = 3.2 Hz), 116.3, 114.9, 113.1 (d, *J* = 22.3 Hz), 46.9, 21.5 ppm.

^{19}F NMR (565 MHz, $CDCl_3$): δ = -112.66 – -112.77 ppm.

HRMS (ESI): mass found: 306.09602, calculated mass for $C_{16}H_{17}FNO_2S^+$: 306.09586.

IR (KBr): 3512, 3279, 3071, 2926, 2161, 1923, 1722, 1584, 1489, 1439, 1324, 1268, 1194, 1156, 1089, 870, 813, 788, 726, 666 cm^{-1}.

4-Methyl-*N*-(2-(*m*-tolyl)allyl)benzenesulfonamide (10j)

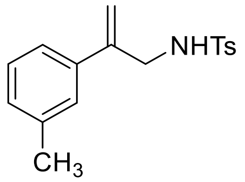

Compound **10j** was prepared according to the general procedure **GP1** and was obtained after column chromatography (*n*-hexane : ethyl acetate 9:1→4:1) as a colorless oil in 50% yield (30 mg).

^{1}H NMR (600 MHz, $CDCl_3$): δ = 7.64 (d, *J* = 8.1 Hz, 2H), 7.22 (d, *J* = 8.0 Hz, 2H), 7.10 (t, *J* = 7.6 Hz, 1H), 7.02 (d, *J* = 7.5 Hz, 1H), 6.97 – 6.89 (m, 2H), 5.27 (s, 1H), 5.11 (s, 1H), 4.35 (t, *J* = 5.9 Hz, 1H), 3.92 (d, *J* = 6.1 Hz, 2H), 2.37 (s, 3H), 2.23 (s, 3H) ppm.

^{13}C NMR (151 MHz, $CDCl_3$): δ = 143.4, 142.9, 138.2, 137.8, 136.8, 129.6, 128.9, 128.4, 127.2, 126.8, 123.1, 115.0, 47.1, 21.5, 21.4 ppm.

HRMS (ESI): mass found: 324.10239, calculated mass for $C_{17}H_{19}NNaO_2S^+$: 324.10287.

IR (KBr): 3521, 3281, 3033, 2923, 2163, 1916, 1721, 1683, 1599, 1490, 1446, 1326, 1156, 1089, 904, 863, 811, 790, 704, 666 cm^{-1}.

***N*-(2-(3-Methoxyphenyl)allyl)-4-methylbenzenesulfonamide (10k)**

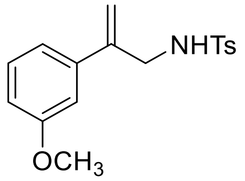

Compound **10k** was prepared according to the general procedure **GP1** and was obtained after column chromatography (*n*-hexane : ethyl acetate 9:1→4:1) as a colorless oil in 49% yield (31 mg).

^{1}H NMR (600 MHz, $CDCl_3$): δ = 7.73 (d, *J* = 8.3 Hz, 2H), 7.32 (d, *J* = 8.0 Hz, 2H), 7.22 (t, *J* = 8.0 Hz, 1H), 6.85 (dd, *J* = 10.8, 5.1 Hz, 2H), 6.81 – 6.77 (m, 1H), 5.39 (s, 1H), 5.23 (s, 1H), 4.47 (t, *J* = 6.0 Hz, 1H), 4.01 (d, *J* = 6.2 Hz, 2H), 3.81 (s, 3H), 2.46 (s, 3H) ppm.

^{13}C NMR (151 MHz, $CDCl_3$): δ = 159.7, 143.5, 142.8, 139.3, 136.8, 129.6, 129.5, 127.2, 118.4, 115.3, 113.5, 112.0, 55.2, 47.1, 21.5 ppm.
HRMS (ESI): mass found: 340.09775, calculated mass for $C_{17}H_{19}NNaO_3S^+$: 340.09779.
IR (KBr): 3870, 3522, 3279, 2935, 2838, 2165, 1923, 1721, 1681, 1597, 1488, 1455, 1430, 1323, 1288, 1227, 1157, 1090, 1041, 861, 813, 785, 700, 667 cm^{-1}.

4-Methyl-*N*-(2-(3-(trifluoromethyl)phenyl)allyl)benzenesulfonamide (10l)

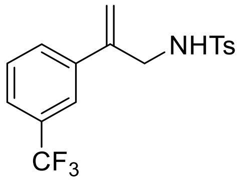

Compound **10l** was prepared according to the general procedure **GP1** and was obtained after column chromatography (*n*-hexane : ethyl acetate 9:1→4:1) as a colorless oil in 57% yield (41 mg).
^{1}H NMR (600 MHz, $CDCl_3$): δ = 7.73 (d, *J* = 8.2 Hz, 2H), 7.56 (d, *J* = 7.5 Hz, 1H), 7.46 (td, *J* = 15.5, 7.8 Hz, 3H), 7.31 (d, *J* = 8.1 Hz, 2H), 5.46 (s, 1H), 5.35 (s, 1H), 4.53 (t, *J* = 6.1 Hz, 1H), 4.05 (d, *J* = 6.3 Hz, 2H), 2.46 (s, 3H) ppm.
^{13}C NMR (151 MHz, $CDCl_3$): δ = 143.7, 141.9, 138.7, 136.6, 130.9 (q, *J* = 32.6 Hz), 129.7, 129.3, 129.0, 127.2, 124.8 (q, *J* = 3.4 Hz), 122.9 (q, *J* = 3.6 Hz), 122.2 (q, *J* = 273.3 Hz), 117.0, 46.9, 21.5 ppm.
^{19}F NMR (565 MHz, $CDCl_3$): δ = -62.72 ppm.
HRMS (ESI): mass found: 356.09307, calculated mass for $C_{17}H_{17}NO_2F_3S^+$: 356.09266.
IR (KBr): 3515, 3279, 3069, 2927, 2160, 1913, 1720, 1598, 1492, 1439, 1328, 1157, 1120, 1072, 905, 808, 726, 698, 664 cm^{-1}.

***N*-(2-(2-Bromophenyl)allyl)-4-methylbenzenesulfonamide (10m)**

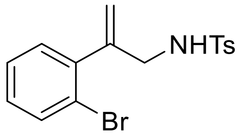

Compound **10m** was prepared according to the general procedure **GP1** and was obtained after column chromatography (*n*-hexane : ethyl acetate 9:1→4:1) as a colorless oil in 51% yield (38 mg).
^{1}H NMR (600 MHz, $CDCl_3$): δ = 7.70 (d, *J* = 8.2 Hz, 2H), 7.51 (d, *J* = 7.3 Hz, 1H), 7.29 – 7.21 (m, 3H), 7.15 (td, *J* = 7.7, 1.7 Hz, 1H), 7.05 (dd, *J* = 7.5, 1.7 Hz, 1H), 5.47 (s, 1H), 5.13 (s, 1H), 4.54 (t, *J* = 6.2 Hz, 1H), 3.95 (d, *J* = 6.4 Hz, 2H), 2.44 (s, 3H) ppm.
^{13}C NMR (151 MHz, $CDCl_3$): δ = 144.7, 143.3, 140.5, 136.8, 132.7, 130.8, 129.6, 129.2, 127.4, 127.1, 122.0, 117.9, 47.6, 21.5 ppm.
HRMS (ESI): mass found: 366.01628, calculated mass for $C_{16}H_{17}NO_2BrS^+$: 366.01579.
IR (KBr): 3282, 3060, 2923, 2162, 1920, 1810, 1724, 1643, 1596, 1426, 1325, 1156, 1086, 1024, 914, 853, 813, 759, 733, 663cm^{-1}.

4-Methyl-*N*-(2-(*o*-tolyl)allyl)benzenesulfonamide (10n)

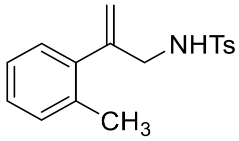

Compound **10n** was prepared according to the general procedure **GP1** and was obtained after column chromatography (*n*-hexane : ethyl acetate 9:1→4:1) as a colorless oil in 42% yield (25 mg).

^{1}H NMR (400 MHz, $CDCl_3$): δ = 7.66 (d, *J* = 8.3 Hz, 2H), 7.36 – 7.21 (m, 2H), 7.19 – 7.03 (m, 3H), 6.91 (d, *J* = 7.5 Hz, 1H), 5.37 (d, *J* = 1.2 Hz, 1H), 4.98 (d, *J* = 1.0 Hz, 1H), 4.48 (t, *J* = 6.1 Hz, 1H), 3.79 (d, *J* = 6.3 Hz, 2H), 2.40 (s, 3H), 2.16 (s, 3H) ppm.

^{13}C NMR (101 MHz, $CDCl_3$): δ = 144.4, 143.3, 139.0, 136.8, 135.1, 130.3, 129.6, 128.5, 127.6, 127.0, 125.6, 116.0, 48.2, 21.4, 19.6 ppm.

HRMS (ESI): mass found: 302.12205, calculated mass for $C_{17}H_{20}NO_2S^+$: 302.12093.

IR (KBr): 3283, 3061, 3022, 2923, 2863, 2416, 2163, 2052, 1988, 1917, 1809, 1725, 1642, 1598, 1490, 1448, 1325, 1235, 1157, 1091, 1063, 993, 907, 813, 765, 730, 704, 664 cm^{-1}.

***N*-(2-(3,5-*bis*(Trifluoromethyl)phenyl)allyl)-4-methylbenzenesulfonamide (10o)**

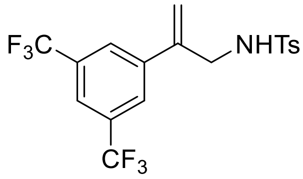

Compound **10o** was prepared according to the general procedure **GP1** and was obtained after column chromatography (*n*-hexane : ethyl acetate 9:1 - 4:1) as colorless oil in 68% (57 mg).

^{1}H NMR (600 MHz, $CDCl_3$): δ = 7.71 (s, 1H), 7.66 – 7.61 (m, 2H), 7.59 (s, 2H), 7.21 (d, *J* = 7.9 Hz, 2H), 5.44 (s, 1H), 5.39 (s, 1H), 4.52 (t, *J* = 6.4 Hz, 1H), 3.98 (d, *J* = 6.5 Hz, 2H), 2.36 (s, 3H) ppm.

^{13}C NMR (151 MHz, $CDCl_3$): δ = 143.9, 141.0, 140.2, 136.6, 131.9 (q, *J* = 33.8 Hz), 129.8, 127.1, 126.3, 123.1 (q, *J* = 273.4 Hz), 121.9 - 121.7 (m), 118.9, 46.7, 21.4 ppm.

^{19}F NMR (565 MHz, $CDCl_3$): δ = -62.92 ppm.

HRMS (ESI): Mass found: 462.03528, calculated mass for $C_{18}H_{15}F_6KNO_2S^+$: 462.03593.

IR (KBr): 3278, 3093, 2930, 2161, 1726, 1599, 1419, 1376, 1327, 1277, 897, 844, 814, 676 cm^{-1}.

4-Chloro-*N*-(2-phenylallyl)benzenesulfonamide (10p)

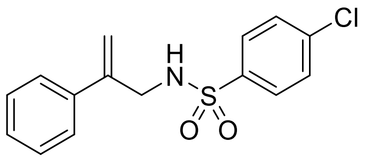

Compound **10p** was prepared according to the general procedure **GP1** and was obtained after column chromatography (*n*-hexane : ethyl acetate 9:1 - 4:1) as a as colorless oil in 61% (37 mg).

^{1}H NMR (600 MHz, $CDCl_3$): δ = 7.75 (d, *J* = 8.4 Hz, 2H), 7.47 (d, *J* = 8.3 Hz, 2H), 7.33 – 7.29 (m, 3H), 7.24 - 7.19 (m, 2H), 5.38 (s, 1H), 5.21 (s, 1H), 4.54 (t, *J* = 6.2 Hz, 1H), 4.07 (d, *J* = 6.2 Hz, 2H) ppm.
^{13}C NMR (151 MHz, $CDCl_3$): δ = 142.7, 139.2, 138.4, 137.6, 129.3, 128.6, 128.3, 126.0, 115.5, 47.1 ppm. (*one carbon signal is missing, due to the overlap)
HRMS (ESI): Mass found: 330.03235, calculated mass for $C_{15}H_{14}ClNNaO_2S^+$: 330.03260.
IR (KBr): 3852, 3524, 3284, 3089, 2929, 2668, 2325, 2163, 2100, 1808, 1725, 1632, 1580, 1475, 1397, 1327, 1279, 1159, 1087, 1014, 907, 826, 754, 703 cm^{-1}.

2-Nitro-*N*-(2-phenylallyl)benzenesulfonamide (10q)

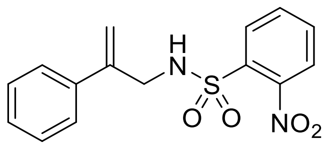

Compound **10q** was prepared according to the general procedure **GP1** and was obtained after column chromatography (*n*-hexane : ethyl acetate 9:1→4:1) as a yellow solid in 69% yield (44 mg).
^{1}H NMR (600 MHz, $CDCl_3$): δ = 8.20 – 8.02 (m, 1H), 7.92 – 7.80 (m, 1H), 7.79 – 7.67 (m, 2H), 7.36 – 7.21 (m, 3H), 7.22 – 7.07 (m, 2H), 5.60 (t, *J* = 5.8 Hz, 1H), 5.35 (s, 1H), 5.29 (s, 1H), 4.26 (d, *J* = 6.3 Hz, 2H) ppm.
^{13}C NMR (151 MHz, $CDCl_3$): δ = 147.7, 142.8, 137.6, 134.2, 133.4, 132.7, 131.1, 128.6, 128.3, 125.9, 125.4, 115.5, 47.8 ppm.
HRMS (ESI): mass found: 319.07507, calculated mass for $C_{15}H_{15}N_2O_4S^+$: 319.07471.
IR (KBr): 3869, 3363, 3099, 3051, 2974, 2936, 2866, 2679, 2321, 2158, 2006, 1889, 1801, 1725, 1627, 1572, 1527, 1442, 1405, 1334, 1234, 1153, 1112, 1076, 979, 938, 900, 860, 821, 776, 731, 706, 663 cm^{-1}.

4-Methyl-*N*-(2,3,4,5-tetrahydro-[1,1'-biphenyl]-2-yl)benzenesulfonamide (10r)

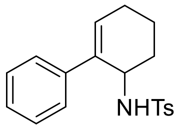

Compound **10r** was prepared according to the general procedure **GP1** and was obtained after column chromatography (*n*-hexane : ethyl acetate 20:1→9:1) as a colorless oil in 70% yield (46 mg). The NMR spectrum is consistent with the previous literature.[3]
^{1}H NMR (400 MHz, $CDCl_3$): δ = 7.50 (d, *J* = 7.9 Hz, 2H), 7.13 (t, *J* = 7.7 Hz, 3H), 7.03 (t, *J* = 7.6 Hz, 2H), 6.90 (d, *J* = 7.8 Hz, 2H), 6.06 (s, 1H), 4.30 (d, *J* = 5.4 Hz, 1H), 4.21 (d, *J* = 2.7 Hz, 1H), 2.41 (s, 3H), 2.28 – 2.01 (m, 3H), 1.93 – 1.57 (m, 3H) ppm.
^{13}C NMR (101 MHz, $CDCl_3$): δ = 142.9, 139.2, 137.1, 136.3, 131.2, 129.4, 128.2, 127.1, 127.0, 126.1, 49.4, 29.9, 25.6, 21.5, 16.5 ppm.
HRMS (ESI): mass found: 350.11816, calculated mass for $C_{19}H_{21}NO_2SNa^+$: 350.11852.
IR (KBr): 3851, 3454, 3258, 3058, 3028, 2936, 2868, 2713, 2507, 2320, 2161, 2102, 2034, 2002, 1969, 1918, 1809, 1718, 1661, 1598, 1493, 1446, 1420, 1323, 1258, 1151, 1067, 998, 945, 914, 876, 811, 757, 696, 667 cm^{-1}.

4-Methyl-*N*-(2-methylcyclopent-2-en-1-yl)benzenesulfonamide (10s)

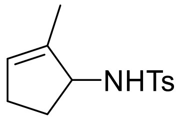

Compound **10s** was prepared according to the general procedure **GP1** and was obtained after column chromatography (*n*-hexane : ethyl acetate 20:1→9:1) as a colorless oil in 51% yield (30 mg).

^{1}H NMR (400 MHz, $CDCl_3$): δ = 7.75 (d, *J* = 8.1 Hz, 2H), 7.28 (d, *J* = 8.2 Hz, 2H), 5.45 (s, 1H), 4.30 (d, *J* = 9.7 Hz, 1H), 4.19 – 4.10 (m, 1H), 2.41 (s, 3H), 2.33 – 2.17 (m, 1H), 2.16 – 1.93 (m, 2H), 1.58 (s, 3H), 1.50 – 1.35 (m, 1H) ppm.

^{13}C NMR (101 MHz, $CDCl_3$): δ = 143.1, 138.7, 138.2, 129.6, 128.6, 127.0, 62.4, 32.4, 29.8, 21.5, 13.5 ppm.

HRMS (ESI): mass found: 252.10457, calculated mass for $C_{13}H_{18}NO_2S^+$: 252.10528.

IR (KBr): 3275, 3043, 2929, 2858, 2734, 2325, 2161, 2092, 1918, 1719, 1597, 1494, 1438, 1321, 1154, 1089, 1036, 990, 904, 813, 705, 664 cm^{-1}.

4-Methyl-*N*-(2-methylene-4-phenylbut-3-yn-1-yl)benzenesulfonamide (10t)

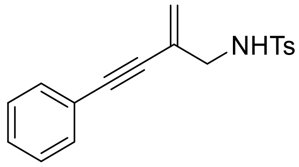

Compound **10t** was prepared according to the general procedure **GP1** and was obtained after column chromatography (*n*-hexane : ethyl acetate 20:1→9:1) as a light yellow oil in 26% yield (16 mg).

^{1}H NMR (600 MHz, $CDCl_3$): δ = 7.86 – 7.76 (m, 2H), 7.41 – 7.38 (m, 2H), 7.37 – 7.33 (m, 3H), 7.30 (d, *J* = 8.0 Hz, 2H), 5.48 (dd, *J* = 13.1, 1.1 Hz, 2H), 4.73 (t, *J* = 6.3 Hz, 1H), 3.78 (d, *J* = 6.4 Hz, 2H), 2.43 (s, 3H) ppm.

^{13}C NMR (151 MHz, $CDCl_3$): δ = 143.5, 137.1, 131.6, 129.6, 128.7, 128.3, 127.2, 126.8, 122.9, 122.3, 91.2, 86.6, 47.8, 21.5 ppm.

HRMS (ESI): mass found: 312.10486, calculated mass for $C_{18}H_{18}NO_2S^+$: 312.10528.

IR (KBr): 3520, 3286, 3059, 2926, 2864, 2201, 2162, 2030, 1986, 1906, 1729, 1676, 1597, 1492, 1446, 1333, 1157, 1086, 1025, 906, 814, 757, 689, 668 cm^{-1}.

***N*-(2-Cyclohexylallyl)-4-methylbenzenesulfonamide (10u)**

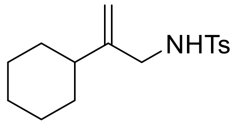

Compound **10u** was prepared according to the general procedure **GP1** and was obtained after column chromatography (*n*-hexane : ethyl acetate 20:1→9:1) as a colorless oil in 48% yield (28 mg).

^{1}H NMR (600 MHz, $CDCl_3$): δ = 7.84 – 7.73 (m, 2H), 7.34 (d, *J* = 8.0 Hz, 2H), 5.05 – 4.64 (m, 2H), 4.35 (t, *J* = 6.2 Hz, 1H), 3.55 (d, *J* = 6.4 Hz, 2H), 2.46 (s, 3H), 1.84 (tt, *J* = 11.8, 2.9 Hz, 1H), 1.78 – 1.72 (m, 2H), 1.71 – 1.65 (m, 3H), 1.34 – 0.99 (m, 5H) ppm.
^{13}C NMR (151 MHz, $CDCl_3$): δ = 149.8, 143.4, 136.9, 129.7, 127.1, 110.3, 47.0, 41.6, 32.1, 26.5, 26.1, 21.5 ppm.
HRMS (ESI): mass found: 316.13368, calculated mass for $C_{16}H_{23}NO_2SNa^+$: 316.13417.
IR (KBr): 3527, 3282, 2925, 2853, 2666, 2165, 2039, 1912, 1731, 1647, 1598, 1495, 1446, 1325, 1156, 1091, 892, 842, 813, 735, 703, 665 cm^{-1}.

_N_-(3,3-Dimethyl-2-methylenebutyl)-4-methylbenzenesulfonamide and 4-Methyl-_N_-(2,2,3-trimethylbut-3-en-1-yl)benzenesulfonamide (10v)

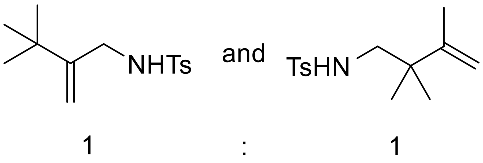

Compound **10v** was prepared according to the general procedure **GP1** and was obtained after column chromatography (*n*-hexane : ethyl acetate 20:1→9:1) as a colorless oil in 64% yield (34 mg).
^{1}H NMR (600 MHz, $CDCl_3$): δ = 7.76 (d, *J* = 8.2 Hz, 1.38H), 7.73 (d, *J* = 8.2 Hz, 1.77H), 7.32 (dd, *J* = 7.8, 5.2 Hz, 3.36H), 4.97 (s, 0.83H) and 4.87 (s, 1.00H), 4.90 (s, 0.81H) and 4.74 (s, 1.03H), 4.34 (t, *J* = 6.0 Hz, 0.70H) and 4.17 (t, *J* = 5.6 Hz, 0.90H), 3.59 (d, *J* = 6.2 Hz, 1.61H) and 2.80 (d, *J* = 6.0 Hz, 2.00H), 2.44 (s, 2.10H) and 2.43 (s, 3.08H), 1.59 (s, 2.18H), 1.56 (s, 2.94H), 1.03 (s, 13.27H) ppm.
^{13}C NMR (151 MHz, $CDCl_3$): δ = 152.7, 148.5, 143.4, 143.3, 136.8, 136.7, 129.69, 129.64, 127.1, 127.0, 112.6, 109.4, 50.9, 44.2, 39.2, 35.3, 29.1, 24.9, 21.5, 18.9 ppm.
HRMS (ESI): mass found: 290.11847, calculated mass for $C_{14}H_{21}NO_2SNa^+$: 290.11852.
IR (KBr): 3286, 2965, 2875, 2158, 2030, 1922, 1725, 1638, 1599, 1449, 1326, 1156, 1087, 896, 814, 706, 663 cm^{-1}.

2-Methyl-2-phenyl-1-tosylaziridine (11a)

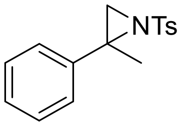

Compound **11a** was prepared according to the general procedure **GP2** and was obtained after column chromatography (*n*-hexane : ethyl acetate 20:1→9:1) as a colorless oil in 80% yield (46 mg).
^{1}H NMR (400 MHz, $CDCl_3$): δ = 7.86 (d, *J* = 8.3 Hz, 2H), 7.40 – 7.35 (m, 2H), 7.33 – 7.28 (m, 4H), 7.28 – 7.22 (m, 1H), 2.95 (s, 1H), 2.51 (s, 1H), 2.42 (s, 3H), 2.04 (s, 3H) ppm.
^{13}C NMR (101 MHz, $CDCl_3$): δ = 143.9, 140.9, 137.7, 129.5, 128.3, 127.7, 127.4, 126.5, 51.8, 41.8, 21.5, 20.9 ppm.
HRMS (ESI): mass found: 288.10533, calculated mass for $C_{16}H_{18}NO_2S^+$: 288.10528.
IR (KBr): 3518, 3061, 2978, 2932, 2173, 2028, 1894, 1813, 1728, 1683, 1597, 1553, 1494, 1444, 1380, 1313, 1156, 1131, 1093, 1057, 1013, 935, 869, 808, 767, 704, 659 cm^{-1}.

2-(Methyl-d3)-2-phenyl-1-tosylaziridine (11a-*d₃*)

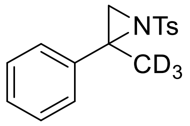

Compound **11a-*d₃*** was prepared according to the general procedure **GP2** and was obtained after column chromatography (*n*-hexane : ethyl acetate 20:1→9:1) as a colorless oil in 80% yield (46 mg), deuterium content: 86%.

^{1}H NMR (400 MHz, $CDCl_3$): δ = 7.86 (d, *J* = 8.3 Hz, 2H), 7.39 – 7.34 (m, 2H), 7.30 (ddd, *J* = 7.4, 4.6, 2.6 Hz, 4H), 7.27 – 7.21 (m, 1H), 2.94 (s, 1H), 2.50 (s, 1H), 2.42 (s, 3H), 2.05 – 1.90 (m, 0.42H) ppm.

^{13}C NMR (101 MHz, $CDCl_3$): δ = 143.9, 140.9, 137.7, 129.4, 128.3, 127.7, 127.4, 126.4, 51.5, 41.7, 21.5, 20.5 ppm.

HRMS (ESI): mass found: 291.12431, calculated mass for $C_{16}H_{15}D_3NO_2S^+$: 291.12411.

IR (KBr): 3505, 3294,3060, 2926, 2161, 2025, 1975, 1918, 1812, 1733, 1597, 1494, 1446, 1403, 1316, 1155, 1088, 1047, 974, 871, 809, 774, 699, 659 cm^{-1}.

2-(4-Chlorophenyl)-2-methyl-1-tosylaziridine (11b)

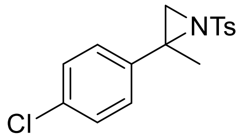

Compound **11b** was prepared according to the general procedure **GP2** and was obtained after column chromatography (*n*-hexane : ethyl acetate 20:1→9:1) as a white solid in 85% yield (55 mg).

^{1}H NMR (400 MHz, $CDCl_3$): δ = 7.84 (d, *J* = 8.3 Hz, 2H), 7.43 – 7.20 (m, 6H), 2.93 (s, 1H), 2.47 (s, 1H), 2.42 (s, 3H), 2.01 (s, 3H) ppm.

^{13}C NMR (101 MHz, $CDCl_3$): δ = 144.1, 139.4, 137.5, 133.6, 129.5, 128.5, 127.9, 127.4, 50.9, 41.8, 21.5, 20.7 ppm.

HRMS (ESI): mass found: 344.04785, calculated mass for $C_{16}H_{16}ClNO_2SNa^+$: 344.04825.

IR (KBr): 3511, 3063, 2928, 2312, 2166, 2035, 1909, 1734, 1597, 1492, 1400, 1321, 1158, 1128, 1092, 1017, 938, 873, 819, 694, 658 cm^{-1}.

2-(4-Bromophenyl)-2-methyl-1-tosylaziridine (11c)

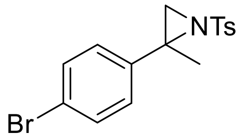

Compound **11c** was prepared according to the general procedure **GP2** and was obtained after column chromatography (*n*-hexane : ethyl acetate 20:1→9:1) as a colorless oil in 85% yield (63 mg).

^{1}H NMR (400 MHz, $CDCl_3$): δ = 7.84 (d, *J* = 8.3 Hz, 2H), 7.46 – 7.38 (m, 2H), 7.31 (d, *J* = 8.0 Hz, 2H), 7.26 – 7.20 (m, 2H), 2.93 (s, 1H), 2.46 (s, 1H), 2.42 (s, 3H), 2.00 (s, 3H) ppm.

^{13}C NMR (101 MHz, $CDCl_3$): δ = 144.1, 140.0, 137.4, 131.5, 129.5, 128.2, 127.4, 121.7, 51.0, 41.8, 21.6, 20.6 ppm.

HRMS (ESI): mass found: 387.99807, calculated mass for $C_{16}H_{16}BrNO_2SNa^+$: 387.99773.
IR (KBr): 3512, 3065, 2926, 2161, 2030, 1909, 1733, 1596, 1490, 1454, 1397, 1321, 1157, 1127, 1090, 1011, 937, 873, 816, 707, 688, 656 cm^{-1}.

4-(2-Methyl-1-tosylaziridin-2-yl)benzonitrile (11d)

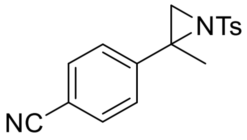

Compound **11d** was prepared according to the general procedure **GP2** and was obtained after column chromatography (*n*-hexane : ethyl acetate 20:1→9:1) as a colorless oil in 77% yield (48 mg).
^{1}H NMR (400 MHz, $CDCl_3$): δ = 7.85 (d, *J* = 8.3 Hz, 2H), 7.65 – 7.57 (m, 2H), 7.53 – 7.44 (m, 2H), 7.33 (d, *J* = 8.0 Hz, 2H), 2.98 (s, 1H), 2.45 (s, 1H), 2.43 (s, 3H), 2.04 (s, 3H) ppm.
^{13}C NMR (101 MHz, $CDCl_3$): δ = 146.0, 144.3, 137.2, 132.3, 129.6, 127.4, 127.3, 118.4, 111.6, 50.6, 41.9, 21.6, 20.1 ppm.
HRMS (ESI): mass found: 335.08225, calculated mass for $C_{17}H_{16}N_2O_2SNa^+$: 335.08247.
IR (KBr): 3065, 2984, 2930, 2229, 2175, 2083, 1924, 1808, 1734, 1691, 1601, 1501, 1452, 1403, 1321, 1262, 1158, 1129, 1093, 1063, 1022, 939, 874, 819, 766, 706, 658 cm^{-1}.

2-(3-Fluorophenyl)-2-methyl-1-tosylaziridine (11e)

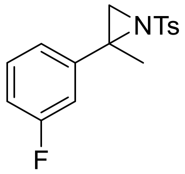

Compound **11e** was prepared according to the general procedure **GP2** and was obtained after column chromatography (*n*-hexane : ethyl acetate 20:1→9:1) as a colorless oil in 84% yield (52 mg).
^{1}H NMR (400 MHz, $CDCl_3$): δ = 7.86 (d, *J* = 8.3 Hz, 2H), 7.32 (d, *J* = 8.0 Hz, 2H), 7.30 – 7.23 (m, 1H), 7.15 (dd, *J* = 7.8, 1.0 Hz, 1H), 7.09 – 7.02 (m, 1H), 7.00 – 6.84 (m, 1H), 2.95 (s, 1H), 2.46 (s, 1H), 2.42 (s, 3H), 2.03 (s, 3H) ppm.
^{13}C NMR (101 MHz, $CDCl_3$): δ = 162.6 (d, *J* = 246.3 Hz), 144.1, 143.5 (d, *J* = 7.4 Hz), 137.5, 129.9 (d, *J* = 8.3 Hz), 129.5, 127.4, 122.0 (d, *J* = 2.8 Hz), 114.7 (d, *J* = 21.1 Hz), 113.6 (d, *J* = 22.7 Hz), 50.8, 42.0, 21.5 , 20.3 ppm.
^{19}F NMR (376 MHz, $CDCl_3$): δ = -104.78 – -129.58 ppm.
HRMS (ESI): mass found: 328.07782, calculated mass for $C_{16}H_{16}FNO_2SNa^+$: 328.07780.
IR (KBr): 3069, 2985, 2929, 2166, 2034, 1735, 1590, 1489, 1443, 1387, 1321, 1202, 1157, 1125, 1091, 1026, 938, 851, 787, 709 cm^{-1}.

2-Methyl-2-(*m*-tolyl)-1-tosylaziridine (11f)

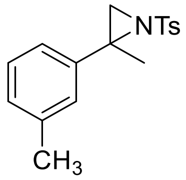

Compound **11f** was prepared according to the general procedure **GP2** and was obtained after column chromatography (*n*-hexane : ethyl acetate 20:1→9:1) as a colorless oil in 75% yield (45 mg).

^{1}H NMR (600 MHz, $CDCl_3$): δ = 7.86 (d, *J* = 8.2 Hz, 2H), 7.31 (d, *J* = 8.2 Hz, 2H), 7.23 – 7.15 (m, 3H), 7.07 (d, *J* = 7.2 Hz, 1H), 2.94 (s, 1H), 2.52 (s, 1H), 2.43 (s, 3H), 2.33 (s, 3H), 2.03 (s, 3H) ppm.

^{13}C NMR (151 MHz, $CDCl_3$): δ = 143.8, 140.9, 138.0, 137.7, 129.4, 128.4, 128.2, 127.4, 127.2, 123.5, 51.9, 41.7, 21.5, 21.4, 21.0 ppm.

HRMS (ESI): mass found: 324.10249, calculated mass for $C_{17}H_{19}NO_2SNa^+$: 324.10287.

IR (KBr): 3516, 3276, 2924, 2857, 2661, 2326, 2174, 2089, 2055, 2028, 1991, 1913, 1802, 1732, 1682, 1599, 1490, 1452, 1379, 1322, 1158, 1089, 940, 905, 844, 786, 705 cm^{-1}.

2-(3-Methoxyphenyl)-2-methyl-1-tosylaziridine (11g)

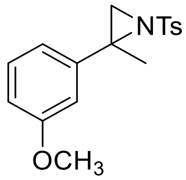

Compound **11g** was prepared according to the general procedure **GP2** and was obtained after column chromatography (*n*-hexane : ethyl acetate 20:1→9:1) as a colorless oil in 72% yield (46 mg).

^{1}H NMR (400 MHz, $CDCl_3$): δ = 7.85 (d, *J* = 8.3 Hz, 2H), 7.30 (d, *J* = 8.4 Hz, 2H), 7.22 (dd, *J* = 13.4, 5.4 Hz, 1H), 6.98 – 6.92 (m, 1H), 6.91 – 6.86 (m, 1H), 6.79 (dd, *J* = 8.2, 2.5 Hz, 1H), 3.77 (s, 3H), 2.93 (s, 1H), 2.49 (s, 1H), 2.41 (s, 3H), 2.02 (s, 3H) ppm.

^{13}C NMR (101 MHz, $CDCl_3$): δ = 159.5, 143.9, 142.6, 137.6, 129.5, 129.4, 127.4, 118.7, 113.0, 112.4, 55.2, 51.6, 41.7, 21.5, 20.7 ppm.

HRMS (ESI): mass found: 340.09772, calculated mass for $C_{17}H_{19}NO_3SNa^+$: 340.09779.

IR (KBr): 3527, 2934, 2838, 2326, 2175, 2080, 1990, 1932, 1731, 1682, 1594, 1488, 1456, 1320, 1227, 1158, 1127, 1092, 1040, 939, 901, 845, 783, 702 cm^{-1}.

2-Methyl-1-tosyl-2-(3-(trifluoromethyl)phenyl)aziridine (11h)

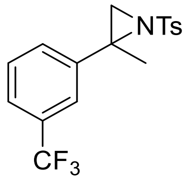

Compound **11h** was prepared according to the general procedure **GP2** and was obtained after column chromatography (*n*-hexane : ethyl acetate 20:1→9:1) as a colorless oil in 83% yield (59 mg).

^{1}H NMR (400 MHz, $CDCl_3$): δ = 7.86 (d, *J* = 8.3 Hz, 2H), 7.60 – 7.55 (m, 2H), 7.54 – 7.46 (m, 1H), 7.43 (t, *J* = 7.7 Hz, 1H), 7.32 (d, *J* = 8.0 Hz, 2H), 2.98 (s, 1H), 2.49 (s, 1H), 2.42 (s, 3H), 2.05 (s, 3H) ppm.

^{13}C NMR (101 MHz, $CDCl_3$): δ = 144.2, 142.0, 137.3, 130.9, 130.0, 129.6, 128.9, 127.5, 124.6 (q, *J* = 3.7 Hz), 123.8 (q, *J* = 272.6 Hz), 123.2 (q, *J* = 3.7 Hz), 50.8, 41.7, 21.5, 20.4 ppm.

^{19}F NMR (376 MHz, $CDCl_3$): δ = -62.66 ppm.

HRMS (ESI): mass found: 378.07446, calculated mass for $C_{17}H_{16}F_3NO_2SNa^+$: 378.07461.

IR (KBr): 3069, 2030, 2165, 2028, 1730, 1598, 1493, 1450, 1387, 1318, 1262, 1160, 1121, 1071, 1027, 939, 885, 808, 709, 663 cm^{-1}.

2-(2-Bromophenyl)-2-methyl-1-tosylaziridine (11i)

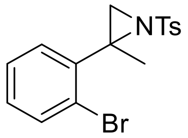

Compound **11i** was prepared according to the general procedure **GP2** and was obtained after column chromatography (*n*-hexane : ethyl acetate 20:1→9:1) as a colorless oil in 66% yield (49 mg).

^{1}H NMR (400 MHz, $CDCl_3$): δ = 7.88 (d, *J* = 8.3 Hz, 2H), 7.59 (dd, *J* = 7.7, 1.7 Hz, 1H), 7.46 (dd, *J* = 8.0, 1.0 Hz, 1H), 7.34 – 7.27 (m, 3H), 7.12 (td, *J* = 7.9, 1.7 Hz, 1H), 3.05 (s, 1H), 2.50 (s, 1H), 2.41 (s, 3H), 2.03 (s, 3H) ppm.

^{13}C NMR (101 MHz, $CDCl_3$): δ = 144.1, 140.8, 137.5, 132.5, 130.9, 129.5, 129.2, 127.6, 127.5, 120.8, 53.3, 42.5, 21.5, 20.0 ppm.

HRMS (ESI): mass found: 387.99804, calculated mass for $C_{16}H_{16}BrNO_2SNa^+$: 387.99773.

IR (KBr): 3063, 2928, 2167, 2028, 1973, 1921, 1910, 1700, 1595, 1566, 1435, 1381, 1322, 1274, 1244, 1158, 1091, 1021, 938, 876, 818, 758, 721, 691 cm^{-1}.

2-Methyl-2-(*o*-tolyl)-1-tosylaziridine (11j)

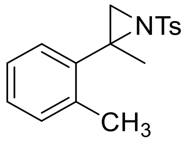

Compound **11j** was prepared according to the general procedure **GP2** and was obtained after column chromatography (*n*-hexane : ethyl acetate 20:1→9:1) as a colorless oil in 65% yield (39 mg).

^{1}H NMR (400 MHz, $CDCl_3$): δ = 7.88 (d, *J* = 8.3 Hz, 2H), 7.42 – 7.35 (m, 1H), 7.33 – 7.26 (m, 2H), 7.19 – 7.13 (m, 2H), 7.13 – 7.07 (m, 1H), 3.02 (s, 1H), 2.44 (s, 1H), 2.43 (s, 3H), 2.41 (s, 3H), 2.00 (s, 3H) ppm.

^{13}C NMR (101 MHz, $CDCl_3$): δ = 143.9, 139.7, 137.8, 134.8, 130.4, 129.5, 128.3, 127.7, 127.4, 126.0, 52.7, 41.8, 21.5, 20.7, 18.7 ppm.

HRMS (ESI): mass found: 324.10251, calculated mass for $C_{17}H_{19}NO_2SNa^+$: 324.10287.

IR (KBr): 3509, 2928, 2163, 1976, 1917, 1809, 1733, 1598, 1492, 1452, 1383, 1320, 1263, 1157, 1090, 1027, 939, 876, 821, 764, 708 cm^{-1}.

2-(2,4-Dimethylphenyl)-2-methyl-1-tosylaziridine (11k)

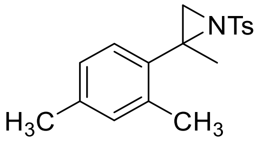

Compound **11k** was prepared according to the general procedure **GP2** and was obtained after column chromatography (*n*-hexane : ethyl acetate 20:1→9:1) as a colorless oil in 60% yield (38 mg).

^{1}H NMR (400 MHz, $CDCl_3$): δ = 7.87 (d, *J* = 8.3 Hz, 2H), 7.27 (dd, *J* = 15.9, 7.8 Hz, 3H), 7.02 – 6.85 (m, 2H), 3.00 (s, 1H), 2.42 (s, 1H), 2.41 (s, 3H), 2.39 (s, 3H), 2.27 (s, 3H), 1.98 (s, 3H) ppm.

^{13}C NMR (101 MHz, $CDCl_3$): δ = 143.8, 137.8, 137.4, 136.9, 134.6, 131.1, 129.4, 128.3, 127.4, 126.6, 52.6, 41.9, 21.5, 20.9, 20.7, 18.6 ppm.

HRMS (ESI): mass found: 338.11853, calculated mass for $C_{18}H_{21}NO_2SNa^+$: 338.11852.

IR (KBr): 3518, 3286, 2925, 2735, 2164, 2028, 1912, 1736, 1679, 1600, 1500, 1450, 1381, 1321, 1266, 1157, 1089, 1060, 1028, 940, 868, 813, 710, 660 cm^{-1}.

2-Methyl-2-phenyl-1-(phenylsulfonyl)aziridine (11l)

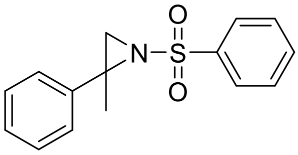

Compound **11l** was prepared according to the general procedure **GP2** and was obtained after column chromatography (*n*-hexane : ethyl acetate 20:1→9:1) as a colorless oil in 77% yield (42 mg).

^{1}H NMR (400 MHz, $CDCl_3$): δ = 7.99 – 7.96 (m, 2H), 7.64 – 7.55 (m, 1H), 7.55 – 7.48 (m, 2H), 7.38 – 7.35 (m, 2H), 7.34 – 7.22 (m, 3H), 2.97 (s, 1H), 2.54 (s, 1H), 2.05 (s, 3H) ppm.

^{13}C NMR (101 MHz, $CDCl_3$): δ = 140.8, 140.5, 133.0, 128.9, 128.4, 127.7, 127.4, 126.4, 52.0, 41.9, 21.0 ppm.

HRMS (ESI): mass found: 274.08967, calculated mass for $C_{15}H_{16}NO_2S^+$: 274.08963.

IR (KBr): 3281, 3063, 2991, 2933, 2325, 2161, 2031, 1993, 1899, 1816, 1728, 1684, 1603, 1494, 1447, 1384, 1316, 1266, 1159, 1131, 1091, 1061, 1023, 938, 871, 806, 762, 738, 690 cm^{-1}.

1-((4-Chlorophenyl)sulfonyl)-2-methyl-2-phenylaziridine (11m)

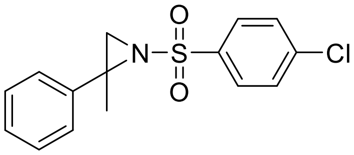

Compound **11m** was prepared according to the general procedure **GP2** and was obtained after column chromatography (*n*-hexane : ethyl acetate 20:1→9:1) as a colorless oil in 59% yield (37 mg).
^{1}H NMR (400 MHz, $CDCl_3$): δ = 7.97 – 7.85 (m, 2H), 7.52 – 7.44 (m, 2H), 7.39 – 7.26 (m, 5H), 2.96 (s, 1H), 2.54 (s, 1H), 2.04 (s, 3H) ppm.
^{13}C NMR (101 MHz, $CDCl_3$): δ = 140.5, 139.6, 139.0, 129.2, 128.9, 128.4, 127.8, 126.4, 52.3, 42.1, 21.1 ppm.
HRMS (ESI): mass found: 308.05030, calculated mass for $C_{15}H_{15}ClNO_2S^+$: 308.05065.
IR (KBr): 3520, 3284, 3029, 2979, 2929, 2320, 2164, 2029, 1911, 1727, 1681, 1582, 1475, 1448, 1392, 1324, 1273, 1160, 1087, 1021, 939, 872, 824, 760, 700 cm^{-1}.

2-Methyl-1-(naphthalen-2-ylsulfonyl)-2-phenylaziridine (11n)

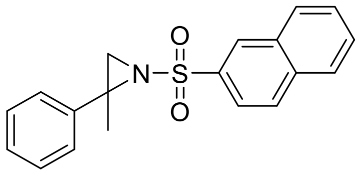

Compound **11n** was prepared according to the general procedure **GP2** and was obtained after column chromatography (*n*-hexane : ethyl acetate 20:1→9:1) as a colorless oil in 70% yield (45 mg).
^{1}H NMR (600 MHz, $CDCl_3$): δ = 8.51 (s, 1H), 8.01 – 7.97 (m, 3H), 7.92 (d, *J* = 8.1 Hz, 1H), 7.67 – 7.59 (m, 2H), 7.42 – 7.38 (m, 2H), 7.34 – 7.30 (m, 2H), 7.29 – 7.24 (m, 1H), 3.03 (s, 1H), 2.57 (s, 1H), 2.10 (s, 3H) ppm.
^{13}C NMR (151 MHz, $CDCl_3$): δ = 129.4, 129.2, 128.9, 128.7, 128.57, 128.51, 128.4, 127.89, 127.80, 127.49, 127.45, 126.9, 126.5, 122.9, 52.1, 42.1, 21.1 ppm.
HRMS (ESI): mass found: 346.08670., calculated mass for $C_{19}H_{17}NO_2SNa^+$: 346.08722.
IR (KBr): 3516, 3276, 3060, 2922, 2852, 2321, 2161, 2098, 2032, 1729, 1624, 1593, 1498, 1448, 1383, 1318, 1154, 1065, 947,868, 812, 754, 698, 658 cm^{-1}.

2-Methyl-1-(methylsulfonyl)-2-phenylaziridine (11o)

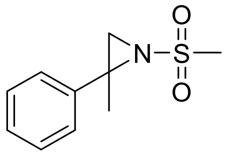

Compound **11o** was prepared according to the general procedure **GP2** and was obtained after column chromatography (*n*-hexane : ethyl acetate 9:1→4:1) as a colorless oil in 40% yield (18 mg).
^{1}H NMR (400 MHz, $CDCl_3$): δ = 7.42 – 7.38 (m, 2H), 7.37 – 7.31 (m, 2H), 7.30 – 7.26 (m, 1H), 3.12 (s, 3H), 2.93 (s, 1H), 2.60 (s, 1H), 1.97 (s, 3H) ppm.
^{13}C NMR (101 MHz, $CDCl_3$): δ = 128.4, 127.8, 126.6, 126.4, 42.3, 41.9, 26.5, 20.8 ppm.
HRMS (ESI): mass found: 212.07335, calculated mass for $C_{10}H_{14}NO_2S^+$: 212.07398.
IR (KBr): 3512, 3286, 3028, 2979, 2933, 2298, 2163, 2029, 1992, 1889, 1726, 1602, 1541, 1496, 1448, 1412, 1382, 1311, 1149, 1063, 1024, 966, 939, 875, 813, 768, 699, 661 cm^{-1}.

4-(2-Methyl-1-tosylaziridin-2-yl)pyridine (11p)

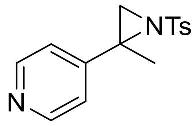

Compound **11r** was prepared according to the general procedure **GP2** and was obtained after column chromatography (*n*-hexane : ethyl acetate 20:1→9:1) as a colorless oil in 71% yield (41 mg).

^{1}H NMR (400 MHz, $CDCl_3$): δ = 8.55 (dd, *J* = 4.6, 1.4 Hz, 2H), 7.94 – 7.79 (m, 2H), 7.33 (d, *J* = 8.0 Hz, 2H), 7.27 (dd, *J* = 4.5, 1.6 Hz, 2H), 2.99 (s, 1H), 2.43 (s, 3H), 2.42 (s, 1H), 2.05 (s, 3H) ppm.

^{13}C NMR (101 MHz, $CDCl_3$): δ = 150.0, 149.5, 144.3, 137.2, 129.6, 127.4, 121.1, 49.6, 42.0, 21.6, 19.1ppm.

HRMS (ESI): mass found: 289.10044, calculated mass for $C_{15}H_{17}N_2O_2S^+$: 289.10053.

IR (KBr): 3405, 3125, 3056, 2972, 2923, 2869, 2431, 2261, 2162, 2033, 1982, 1906, 1735, 1637, 1598, 1560, 1496, 1454, 1410, 1381, 1323, 1157, 1085, 939, 815, 713, 659 cm^{-1}.

2-(2-Methyl-1-tosylaziridin-2-yl)pyridine (11q)

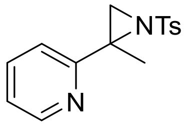

Compound **11s** was prepared according to the general procedure **GP2** and was obtained after column chromatography (*n*-hexane : ethyl acetate 20:1→9:1) as a white solid in 55% yield (32 mg).

^{1}H NMR (600 MHz, $CDCl_3$): δ = 8.45 (d, *J* = 4.7 Hz, 1H), 7.81 (d, *J* = 8.0 Hz, 2H), 7.59 (t, *J* = 7.7 Hz, 1H), 7.41 (d, *J* = 7.9 Hz, 1H), 7.26 (d, *J* = 8.0 Hz, 2H), 7.12 (dd, *J* = 7.4, 4.9 Hz, 1H), 2.93 (s, 1H), 2.61 (s, 1H), 2.37 (s, 3H), 2.07 (s, 3H) ppm.

^{13}C NMR (151 MHz, $CDCl_3$): δ = 159.1, 148.8, 144.1, 137.6, 136.8, 129.6, 127.5, 122.7, 121.2, 51.9, 41.7, 21.6, 18.4 ppm.

HRMS (ESI): mass found: 289.10007, calculated mass for $C_{15}H_{17}N_2O_2S^+$: 289.10053.

IR (KBr): 3356, 3262, 3062, 2926, 2163, 1914, 1728, 1591, 1442, 1386, 1319, 1157, 1091, 1017, 941, 903, 863, 799, 752, 718, 681, 661 cm^{-1}.

2-(4-Chlorophenyl)-2-ethyl-1-tosylaziridine (11r)

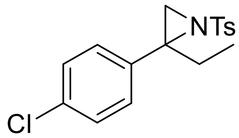

Compound **11p** was prepared according to the general procedure **GP2** and was obtained after column chromatography (*n*-hexane : ethyl acetate 20:1→9:1) as a colorless oil in 92% yield (62 mg).

^{1}H NMR (400 MHz, $CDCl_3$): δ = 7.79 (d, *J* = 8.3 Hz, 2H), 7.36 – 7.21 (m, 6H), 2.78 (s, 1H), 2.60 (s, 1H), 2.40 (s, 3H), 2.39 – 2.29 (m, 1H), 2.26 – 2.12 (m, 1H), 0.92 (t, *J* = 7.4 Hz, 3H) ppm.

^{13}C NMR (101 MHz, $CDCl_3$): δ = 144.0, 137.3, 136.7, 133.7, 129.5, 129.4, 128.4, 127.4, 57.0, 39.8, 28.6, 21.5, 10.8 ppm.
HRMS (ESI): mass found: 358.06387, calculated mass for $C_{17}H_{18}ClNO_2SNa^+$: 358.06390.
IR (KBr): 3065, 2973, 2931, 2880, 2164, 2028, 1910, 1733, 1597, 1492, 1457, 1399, 1368, 1321, 1253, 1157, 1089, 1047, 1015, 941, 870, 817, 737, 705, 656 cm^{-1}.

2-(*tert*-Butyl)-2-phenyl-1-tosylaziridine (11s)

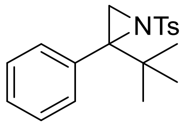

Compound **11q** was prepared according to the general procedure **GP2** and was obtained after column chromatography (*n*-hexane : ethyl acetate 20:1→9:1) as a white solid in 95% yield (63 mg).
^{1}H NMR (400 MHz, $CDCl_3$): δ = 7.74 (d, *J* = 8.3 Hz, 2H), 7.64 – 7.45 (m, 2H), 7.35 – 7.29 (m, 3H), 7.28 – 7.22 (m, 2H), 2.78 (s, 1H), 2.57 (s, 1H), 2.40 (s, 3H), 0.95 (s, 9H) ppm.
^{13}C NMR (101 MHz, $CDCl_3$): δ = 143.5, 137.4, 134.8, 131.3, 129.3, 128.1, 127.6, 127.1, 63.2, 37.3, 35.4, 26.5, 21.5 ppm.
HRMS (ESI): mass found: 352.13386, calculated mass for $C_{19}H_{23}NO_2SNa^+$: 352.13417.
IR (KBr): 3270, 3033, 2964, 2923, 2867, 2607, 2292, 2195, 2161, 2087, 2033, 1988, 1913, 1721, 1598, 1488, 1447, 1391, 1363, 1318, 1193, 1157, 1093, 1024, 950, 857, 812, 753, 704, 669 cm^{-1}.

1-Phenyl-7-tosyl-7-azabicyclo[4.1.0]heptane (11t)

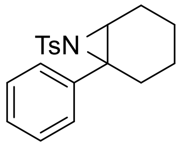

Compound **11t** was prepared according to the general procedure **GP2** and was obtained after column chromatography (*n*-hexane : ethyl acetate 20:1→9:1) as a colorless oil in 86% yield (60 mg).
^{1}H NMR (400 MHz, $CDCl_3$): δ = 7.61 (d, *J* = 8.2 Hz, 2H), 7.40 – 7.35 (m, 2H), 7.31 – 7.28 (m, 3H), 7.21 (d, *J* = 8.3 Hz, 2H), 3.54 (d, *J* = 5.4 Hz, 1H), 2.53 (dt, *J* = 14.3, 4.7 Hz, 1H), 2.39 (s, 3H), 2.10 – 1.91 (m, 1H), 1.81 – 1.62 (m, 2H), 1.62 – 1.55 (m, 1H), 1.54 – 1.43 (m, 1H), 1.43 – 1.32 (m, 1H), 1.30 – 1.13 (m, 1H) ppm.
^{13}C NMR (101 MHz, $CDCl_3$): δ = 143.3, 137.8, 137.5, 129.2, 128.4, 128.2, 128.1, 127.4, 56.4, 43.9, 33.4, 22.8, 21.5, 19.8, 19.7 ppm.
HRMS (ESI): mass found: 350.11801, calculated mass for $C_{19}H_{21}NO_2SNa^+$: 350.11852.
IR (KBr): 3518, 3261, 3058, 3030, 2935, 2863, 2725, 2306, 2162, 2022, 1918, 1720, 1657, 1597, 1494, 1445, 1414, 1321, 1153, 1088, 997, 918, 879, 811, 757, 695, 665 cm^{-1}.

1-Methyl-6-tosyl-6-azabicyclo[3.1.0]hexane (11u)

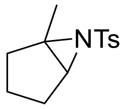

Compound **11u** was prepared according to the general procedure **GP2** and was obtained after column chromatography (*n*-hexane : ethyl acetate 20:1→9:1) as a colorless oil in 75% yield (38 mg).

^{1}H NMR (400 MHz, $CDCl_3$): δ = 7.79 (d, *J* = 8.1 Hz, 2H), 7.27 (d, *J* = 8.0 Hz, 2H), 3.29 (s, 1H), 2.40 (s, 3H), 2.09 – 1.97 (m, 1H), 1.83 (s, 3H), 1.76 (dd, *J* = 13.4, 7.8 Hz, 1H), 1.69 – 1.56 (m, 1H), 1.56 – 1.45 (m, 2H), 1.45 – 1.28 (m, 1H) ppm.

^{13}C NMR (101 MHz, $CDCl_3$): δ = 143.3, 138.7, 129.3, 126.9, 58.5, 53.1, 34.8, 27.7, 21.5, 20.4, 14.9 ppm.

HRMS (ESI): mass found: 274.08670, calculated mass for $C_{13}H_{17}NO_2SNa^+$: 274.08722.

IR (KBr): 3626, 2957, 2162, 2032, 1916, 1722, 1598, 1495, 1452, 1395, 1312, 1230, 1152, 1083, 1007, 959, 896, 859, 814, 715, 678 cm^{-1}.

2-Methyl-2-(phenylethynyl)-1-tosylaziridine (11v)

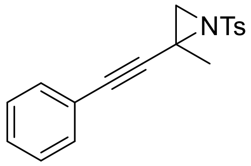

Compound **11v** was prepared according to the general procedure **GP2** and was obtained after column chromatography (*n*-hexane : ethyl acetate 20:1→9:1) as a light yellow oil in 35% yield (22 mg).

^{1}H NMR (600 MHz, $CDCl_3$): δ = 7.99 – 7.82 (m, 2H), 7.55 – 7.44 (m, 2H), 7.39 – 7.30 (m, 5H), 3.02 (s, 1H), 2.58 (s, 1H), 2.46 (s, 3H), 1.74 (s, 3H) ppm.

^{13}C NMR (151 MHz, $CDCl_3$): δ = 144.2, 136.6, 131.8, 129.5, 128.7, 128.2, 127.9, 122.1, 90.4, 88.6, 41.9, 38.8, 23.5, 21.6 ppm.

HRMS (ESI): mass found: 334.08700, calculated mass for $C_{18}H_{17}NO_2SNa^+$: 334.08722.

IR (KBr): 3510, 3283, 3059, 2982, 2927, 2324, 2179, 1928, 1810, 1727, 1673, 1596, 1570, 1491, 1445, 1326, 1271, 1158, 1089, 1027, 933, 819, 756, 737, 690 cm^{-1}.

2-Cyclopentyl-2-methyl-1-tosylaziridine (11w)

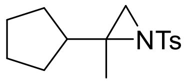

Compound **11w** was prepared according to the general procedure **GP2** and was obtained after column chromatography (*n*-hexane : ethyl acetate 20:1→9:1) as a colorless oil in 78% yield (44 mg).

^{1}H NMR (600 MHz, $CDCl_3$): δ = 7.84 (d, *J* = 8.2 Hz, 2H), 7.32 (d, *J* = 8.0 Hz, 2H), 2.60 (s, 1H), 2.45 (s, 3H), 2.27 (s, 1H), 2.13 – 1.96 (m, 1H), 1.85 – 1.75 (m, 1H), 1.70 (s, 3H), 1.65 – 1.56 (m, 3H), 1.57 – 1.48 (m, 2H), 1.43 – 1.33 (m, 1H), 1.32 – 1.21 (m, 1H) ppm.

^{13}C NMR (151 MHz, $CDCl_3$): δ = 143.5, 138.2, 129.4, 127.3, 52.5, 46.0, 40.0, 28.9, 27.5, 25.5, 25.4, 21.5, 17.5 ppm.

HRMS (ESI): mass found: 302.11826, calculated mass for $C_{15}H_{21}NO_2SNa^+$: 302.11852.

IR (KBr): 2952, 2870, 2584, 2167, 2035, 1916, 1737, 1598, 1493, 1453, 1387, 1317, 1232, 1156, 1090, 1019, 977, 938, 860, 816, 751, 708, 687, 656 cm^{-1}.

2-Cyclohexyl-2-methyl-1-tosylaziridine (11x)

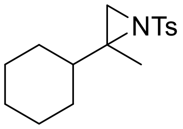

Compound **11x** was prepared according to the general procedure **GP2** and was obtained after column chromatography (*n*-hexane : ethyl acetate 20:1→9:1) as a colorless oil in 75% yield (44 mg).

^{1}H NMR (600 MHz, $CDCl_3$): δ = 7.87 – 7.83 (m, 2H), 7.33 (d, *J* = 8.0 Hz, 2H), 2.61 (s, 1H), 2.45 (s, 3H), 2.23 (s, 1H), 1.85 – 1.74 (m, 3H), 1.71 – 1.65 (m, 2H), 1.63 (s, 3H), 1.31 – 1.06 (m, 6H) ppm.

^{13}C NMR (151 MHz, $CDCl_3$): δ = 143.6, 138.2, 129.4, 127.3, 54.4, 46.1, 41.0, 28.7, 28.6, 26.24, 26.22, 26.0, 21.5, 14.7 ppm.

HRMS (ESI): mass found: 294.15167, calculated mass for $C_{16}H_{24}NO_2S^+$: 294.15223.

IR (KBr): 3046, 2982, 2927, 2853, 2664, 2324, 2178, 2047, 2000, 1911, 1800, 1725, 1597, 1537, 1491, 1446, 1388, 1306, 1225, 1150, 1111, 1086, 1001, 948, 883, 834, 741, 692 cm^{-1}.

2-Methyl-2-(tetrahydro-2H-pyran-4-yl)-1-tosylaziridine (11y)

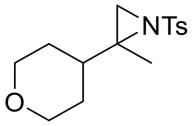

Compound **11y** was prepared according to the general procedure **GP2** and was obtained after column chromatography (*n*-hexane : ethyl acetate 4:1→2:1) as a colorless oil in 60% yield (35 mg).

^{1}H NMR (400 MHz, $CDCl_3$): δ = 7.81 (d, *J* = 7.9 Hz, 2H), 7.31 (d, *J* = 8.1 Hz, 2H), 4.10 – 3.89 (m, 2H), 3.60 – 3.14 (m, 2H), 2.60 (s, 1H), 2.43 (s, 3H), 2.25 (s, 1H), 1.65 (s, 3H), 1.63 – 1.42 (m, 5H) ppm.

^{13}C NMR (101 MHz, $CDCl_3$): δ = 143.8, 137.9, 129.4, 127.3, 67.7, 67.6, 52.9, 42.6, 40.0, 28.5, 28.2, 21.5, 15.3 ppm.

HRMS (ESI): mass found: 318.11313, calculated mass for $C_{15}H_{21}NO_3SNa^+$: 318.11344.

IR (KBr): 3356, 3260, 2949, 2845, 2756, 2691, 2589, 2165, 2032, 1918, 1735, 1657, 1598, 1527, 1496, 1449, 1388, 1298, 1241, 1150, 1086, 1014, 956, 905, 867, 836, 813, 754, 694, 657 cm^{-1}.

2-(*tert*-Butyl)-2-methyl-1-tosylaziridine (11z)

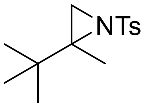

Compound **11z** was prepared according to the general procedure **GP2** and was obtained after column chromatography (*n*-hexane : ethyl acetate 20:1→9:1) as a colorless oil in 95% yield (51 mg).

^{1}H NMR (600 MHz, $CDCl_3$): δ = 7.85 (d, *J* = 8.2 Hz, 2H), 7.32 (d, *J* = 8.1 Hz, 2H), 2.54 (s, 1H), 2.45 (s, 3H), 2.35 (s, 1H), 1.74 (s, 3H), 0.95 (s, 9H) ppm.

^{13}C NMR (151 MHz, $CDCl_3$): δ = 143.5, 138.4, 129.3, 127.3, 56.1, 38.6, 34.6, 25.9, 21.5, 14.9 ppm.
HRMS (ESI): mass found: 290.11834, calculated mass for $C_{14}H_{21}NO_2SNa^+$: 290.11852.
IR (KBr): 2966, 2875, 2177, 1919, 1735, 1599, 1460, 1386, 1316, 1210, 1156, 1113, 1069, 1042, 1005, 945, 901, 830, 709, 660 cm^{-1}.

2-Methyl-2-(1-phenylethyl)-1-tosylaziridine (11aa)

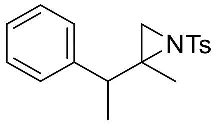

Compound **11aa** was prepared according to the general procedure **GP2** and was obtained after column chromatography (*n*-hexane : ethyl acetate 20:1→9:1) as a colorless oil in 80% yield (50 mg); d.r.56:44.
^{1}H NMR (600 MHz, $CDCl_3$): δ = 7.88 (d, *J* = 8.2 Hz, 1.53H) and 7.83 (d, *J* = 8.2 Hz, 1.96H), 7.39 – 7.30 (m, 5.25H) and 7.30 – 7.19 (m, 7.41H), 2.85 (q, *J* = 7.2 Hz, 1.01H) and 2.70 (q, *J* = 7.1 Hz, 0.81H), 2.67 (s, 0.81H), and 2.66 (s, 1.00H), 2.51 (s, 0.81H), 2.48 (s, 2.37H) and 2.46 (s, 3.00H), 2.17 (s, 1.03H), 1.64 (s, 3.09H) and 1.57 (s, 2.42H), 1.42 (d, *J* = 7.1 Hz, 2.32H) and 1.34 (d, *J* = 7.2 Hz, 3.02H) ppm.
^{13}C NMR (151 MHz, $CDCl_3$): δ = 143.8 and 143.7, 141.6 and 141.5, 138.0 and 137.9, 129.5 and 129.4, 128.4 and 128.1, 128.0 and 127.9, 127.47 and 127.40, 126.9 and 126.7, 54.3 and 53.8, 46.8 and 46.0, 41.3 and 39.2, 21.6 and 21.5, 16.1 and 15.7, 15.7 and 14.2 ppm.
HRMS (ESI): mass found: 338.11871, calculated mass for $C_{18}H_{21}NO_2SNa^+$: 338.11852.
IR (KBr): 3523, 3061, 3030, 2970, 2928, 2161, 2032, 1915, 1808, 1728, 1599, 1494, 1453, 1382, 1318, 1262, 1157, 1090, 1020, 986, 941, 897, 822, 773, 706, 659 cm^{-1}.

2-Benzyl-2-methyl-1-tosylaziridine (11ab)

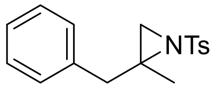

Compound **11ab** was prepared according to the general procedure **GP2** and was obtained after column chromatography (*n*-hexane : ethyl acetate 20:1→9:1) as a colorless oil in 84% yield (51 mg).
^{1}H NMR (600 MHz, $CDCl_3$): δ = 7.89 – 7.81 (m, 2H), 7.34 (d, *J* = 8.0 Hz, 2H), 7.32 – 7.28 (m, 2H), 7.28 – 7.24 (m, 1H), 7.23 – 7.19 (m, 2H), 2.97 (s, 2H), 2.63 (s, 1H), 2.47 (s, 3H), 2.44 (s, 1H), 1.60 (s, 3H) ppm.
^{13}C NMR (151 MHz, $CDCl_3$): δ = 143.8, 137.8, 136.8, 129.52, 129.51, 128.3, 127.4, 126.8, 50.8, 44.0, 40.3, 21.6, 18.4 ppm.
HRMS (ESI): mass found: 324.10217, calculated mass for $C_{17}H_{19}NO_2SNa^+$: 324.10287.
IR (KBr): 3541, 3062, 3030, 2926, 2322, 2163, 2036, 1991, 1920, 1809, 1725, 1598, 1494, 1452, 1386, 1316, 1155, 1089, 1029, 951, 821, 761, 704 cm^{-1}.

2-methyl-2-phenethyl-1-tosylaziridine (11ac)

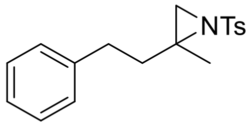

Compound **11ac** was prepared according to the general procedure **GP2** and was obtained after column chromatography (*n*-hexane : ethyl acetate 20:1→9:1) as a colorless oil in 87% yield (55 mg).

^{1}H NMR (600 MHz, $CDCl_3$): δ = 7.86 (d, *J* = 8.3 Hz, 2H), 7.34 (d, *J* = 8.0 Hz, 2H), 7.32 – 7.27 (m, 2H), 7.20 (dd, *J* = 14.5, 7.2 Hz, 3H), 2.87 – 2.69 (m, 2H), 2.58 (s, 1H), 2.46 (s, 3H), 2.28 (s, 1H), 2.03 (ddd, *J* = 13.8, 10.7, 6.5 Hz, 1H), 1.93 (ddd, *J* = 13.9, 10.5, 5.5 Hz, 1H), 1.74 (s, 3H) ppm.

^{13}C NMR (151 MHz, $CDCl_3$): δ = 143.8, 141.2, 138.0, 129.5, 128.4, 128.3, 127.3, 126.0, 50.5, 41.4, 39.6, 32.0, 21.5, 18.6 ppm.

HRMS (ESI): mass found: 338.11777, calculated mass for $C_{18}H_{21}NO_2SNa^+$: 338.11852.

IR (KBr): 3538, 3062, 3028, 2929, 2863, 2180, 2033, 1920, 1808, 1724, 1599, 1495, 1453, 1386, 1316, 1212, 1155, 1107, 1040, 946, 816, 737, 700 cm^{-1}.

2-hexyl-2-methyl-1-tosylaziridine (11ad)

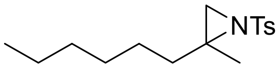

Compound **11ad** was prepared according to the general procedure **GP2** and was obtained after column chromatography (*n*-hexane : ethyl acetate 20:1→9:1) as a colorless oil in 89% yield (53 mg).

^{1}H NMR (600 MHz, $CDCl_3$): δ = 7.85 (d, *J* = 8.3 Hz, 2H), 7.33 (d, *J* = 8.1 Hz, 2H), 2.57 (s, 1H), 2.45 (s, 3H), 2.30 (s, 1H), 1.70 – 1.65 (m, 1H), 1.64 (s, 3H), 1.63 – 1.56 (m, 1H), 1.49 – 1.35 (m, 2H), 1.35 – 1.24 (m, 6H), 0.90 (t, *J* = 7.0 Hz, 3H) ppm.

^{13}C NMR (151 MHz, $CDCl_3$): δ = 143.6, 138.1, 129.4, 127.3, 51.1, 41.4, 37.8, 31.6, 29.0, 25.7, 22.5, 21.5, 18.6, 14.0 ppm.

HRMS (ESI): mass found: 318.14923, calculated mass for $C_{16}H_{25}NO_2SNa^+$: 318.14982.

IR (KBr): 2928, 2860, 2324, 2191, 2086, 1986, 1733, 1599, 1458, 1384, 1318, 1213, 1157, 1093, 1057, 1034, 946, 824, 741, 705, 677 cm^{-1}.

2-Ethyl-2-phenyl-1-tosylaziridine (14)

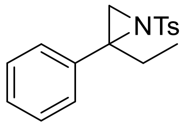

Compound **14** was prepared according to the general procedure **GP2** and was obtained after column chromatography (*n*-hexane : ethyl acetate 20:1→9:1) as a colorless oil in 92% yield (56 mg).

^{1}H NMR (400 MHz, $CDCl_3$): δ = 7.80 (d, *J* = 8.2 Hz, 2H), 7.49 – 7.19 (m, 7H), 2.80 (s, 1H), 2.63 (d, *J* = 0.7 Hz, 1H), 2.46 – 2.33 (m, 4H), 2.23 (dq, *J* = 14.8, 7.5 Hz, 1H), 0.94 (t, *J* = 7.4 Hz, 3H) ppm.

^{13}**C NMR** (101 MHz, $CDCl_3$): δ = 143.8, 138.3, 137.5, 129.4, 128.1, 128.0, 127.8, 127.5, 57.8, 39.8, 28.6, 21.5, 10.9 ppm.
HRMS (ESI): mass found: 324.10137, calculated mass for $C_{17}H_{19}NNaO_2S^+$: 324.10287.
IR (KBr): 3285, 3061, 3032, 2973, 2933, 2880, 2325, 2160, 2030, 1913, 1811, 1731, 1685, 1598, 1494, 1452, 1365, 1319, 1256, 1157, 1093, 1047, 1027, 994, 945, 868, 840, 814, 766, 720, 699, 658 cm^{-1}.

(2R,3R)-2-Methyl-3-phenyl-1-tosylaziridine (20a)

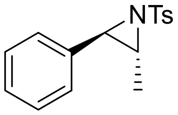

Compound **20a** was prepared from *Z*-*β*-methyl styrene according to the general procedure **GP1** and was obtained after column chromatography (*n*-hexane : ethyl acetate 20:1→9:1) as a yellow oil in 89% yield (51 mg); d.r.81:19.
Compound **20a** was prepared from *E*-*β*-methyl styrene according to the general procedure **GP1** and was obtained after column chromatography (*n*-hexane : ethyl acetate 20:1→9:1) as a yellow oil in 82% yield (47 mg); d.r. 91:9.
Compound **20a** was prepared from *Z*-*β*-methyl styrene according to the general procedure **GP2** and was obtained after column chromatography (*n*-hexane : ethyl acetate 20:1→9:1 as a yellow oil in 93% yield (53 mg); d.r.85:15.
Compound **20a** was prepared from *E*-*β*-methyl styrene according to the general procedure **GP2** and was obtained after column chromatography (*n*-hexane : ethyl acetate 20:1→9:1) as a yellow oil in 96% yield (55 mg); d.r. 93:7.
^{1}**H NMR** (600 MHz, $CDCl_3$): δ = 7.88 (d, *J* = 8.2 Hz, 0.17H) and 7.85 – 7.76 (m, 1.86H), 7.33 (d, *J* = 8.0 Hz, 0.23H) and 7.25 (dt, *J* = 9.4, 4.9 Hz, 5.31H), 7.23 – 7.18 (m, 0.20H) and 7.14 (dd, *J* = 7.6, 2.1 Hz, 1.95H), 3.92 (d, *J* = 7.3 Hz, 0.09H) and 3.79 (d, *J* = 4.3 Hz, 0.95H), 3.19 (dq, *J* = 7.4, 5.9 Hz, 0.08H) and 2.91 (qd, *J* = 6.0, 4.1 Hz, 0.96H), 2.43 (s, 0.32H) and 2.39 (s, 3.00H), 1.84 (d, *J* = 6.0 Hz, 3.14H) and1.02 (d, *J* = 5.8 Hz, 0.29H) ppm.
^{13}**C NMR** (151 MHz, $CDCl_3$): δ = 144.4 and 143.8, 137.8, 135.5, 129.7 and 129.5, 128.4 and 128.2, 128.0 and 127.8, 127.5 and127.1, 126.2, 49.18 and 41.5, 49.12 and 46.0, 21.5, 14.1 and 11.9 ppm.
HRMS (ESI): mass found: 288.10541, calculated mass for $C_{16}H_{18}NO_2S^+$: 288.10528.
IR (KBr): 3367, 3033, 2927, 2859, 2678, 2325, 2166, 2108, 1914, 1810, 1729, 1677, 1598, 1496, 1454, 1410, 1319, 1240, 1157, 1089, 1037, 970, 887, 814, 748, 684 cm^{-1}.

(2R,3R)-2-(4-Bromophenyl)-3-methyl-1-tosylaziridine (20b)

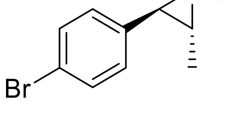

Compound **20b** was prepared according to the general procedure **GP1** and was obtained after column chromatography (*n*-hexane : ethyl acetate 20:1→9:1) as a colorless oil in 70% yield (51 mg); d.r.75:25. (from crude NMR)

Compound **20b** was prepared according to the general procedure **GP2** and was obtained after column chromatography (*n*-hexane : ethyl acetate 20:1→9:1) as a colorless oil in 79% yield (58 mg); d.r.78:22. (from crude NMR)
Main product:
^{1}H NMR (600 MHz, $CDCl_3$): δ = 7.83 (d, *J* = 8.2 Hz, 2H), 7.39 (d, *J* = 8.4 Hz, 2H), 7.29 (d, *J* = 7.3 Hz, 2H), 7.03 (d, *J* = 8.4 Hz, 2H), 3.76 (d, *J* = 4.2 Hz, 2H), 3.00 – 2.78 (m, 2H), 2.42 (s, 3H), 1.85 (d, *J* = 6.0 Hz, 3H) ppm.
^{13}C NMR (151 MHz, $CDCl_3$): δ = 144.1, 137.7, 134.7, 131.6, 129.6, 127.9, 127.2, 122.0, 49.2, 48.3, 21.6, 14.0 ppm.
HRMS (ESI): mass found: 387.99776, calculated mass for $C_{16}H_{16}BrNO_2SNa^+$: 387.99773.
IR (KBr): 3551, 2977, 2930, 2174, 2007, 1913, 1596, 1489, 1449, 1421, 1398, 1236, 1156, 1089, 1040, 1010, 976, 885, 815, 775, 736, 709, 680 cm^{-1}.

(2R,3R)-2-Methyl-1-tosyl-3-(4-(trifluoromethyl)phenyl)aziridine (20c)

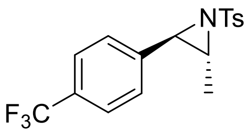

Compound **20c** was prepared according to the general procedure **GP1** and was obtained after column chromatography (*n*-hexane : ethyl acetate 20:1→9:1) as a colorless oil in 80% yield (57 mg); d.r.86:14. (from crude NMR)
Compound **20c** was prepared according to the general procedure **GP2** and was obtained after column chromatography (*n*-hexane : ethyl acetate 20:1→9:1) as a colorless oil in 90% yield (64 mg); d.r.88:12. (from crude NMR)
Main product:
^{1}H NMR (600 MHz, $CDCl_3$): δ = 7.93 – 7.65 (m, 2H), 7.62 – 7.39 (m, 2H), 7.29 (dd, *J* = 14.1, 7.6 Hz, 4H), 3.85 (d, *J* = 4.2 Hz, 1H), 3.13 – 2.68 (m, 1H), 2.43 (s, 3H), 1.88 (d, *J* = 6.0 Hz, 3H) ppm.
^{13}C NMR (151 MHz, $CDCl_3$): δ = 144.2, 139.7, 137.6, 130.2 (q, *J* = 32.6 Hz), 129.6, 127.2, 126.4, 125.5 (q, *J* = 3.6 Hz), 123.9 (q, *J* = 272.3 Hz), 49.5, 48.1, 21.59, 14.0 ppm.
^{19}F NMR (565 MHz, $CDCl_3$): δ = -62.65 ppm.
HRMS (ESI): mass found: 394.04830, calculated mass for $C_{17}H_{16}F_3NO_2SK^+$: 394.04854.
IR (KBr): 3338, 2927, 2174, 1921, 1619, 1598, 1495, 1431, 1405, 1320, 1238, 1158, 1120, 1064, 977, 890, 852, 819, 737, 707, 682 cm^{-1}.

(2R,3R)-2-Methyl-1-tosyl-3-(4-(trifluoromethoxy)phenyl)aziridine (20d)

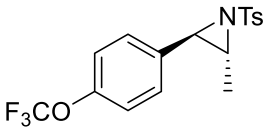

Compound **20d** was prepared according to the general procedure **GP1** and was obtained after column chromatography (*n*-hexane : ethyl acetate 20:1→9:1) as a colorless oil in 75% yield (54 mg); d.r.82:18. (from crude NMR)

Compound **20d** was prepared according to the general procedure **GP2** and was obtained after column chromatography (*n*-hexane : ethyl acetate 20:1→9:1) as a colorless oil in 89% yield (64 mg); d.r.86:14. (from crude NMR)
Main product:
^{1}H NMR (600 MHz, $CDCl_3$): δ = 7.84 (d, *J* = 8.2 Hz, 2H), 7.30 (d, *J* = 8.1 Hz, 2H), 7.19 (d, *J* = 8.6 Hz, 2H), 7.12 (d, *J* = 8.4 Hz, 2H), 3.81 (d, *J* = 4.2 Hz, 1H), 3.02 – 2.75 (m, 1H), 2.43 (s, 3H), 1.86 (d, *J* = 6.0 Hz, 3H) ppm.
^{13}C NMR (151 MHz, $CDCl_3$): δ = 148.9, 144.1, 137.7, 134.3, 129.6, 127.7, 127.2, 121.0, 120.3 (q, *J* = 257.6 Hz), 49.2, 48.1, 21.5, 14.0 ppm.
^{19}F NMR (565 MHz, $CDCl_3$): δ = -57.91 ppm.
HRMS (ESI): mass found: 394.06938, calculated mass for $C_{17}H_{16}F_3NO_3SNa^+$: 394.06952.
IR (KBr): 2934, 2327, 2176, 1913, 1597, 1510, 1449, 1403, 1322, 1255, 1219, 1155, 1091, 1040, 976, 890, 855, 816, 734, 682 cm^{-1}.

(2R,3R)-2-Methyl-1-tosyl-3-(3-(trifluoromethoxy)phenyl)aziridine (20e)

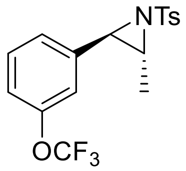

Compound **20e** was prepared according to the general procedure **GP1** and was obtained after column chromatography (*n*-hexane : ethyl acetate 20:1→9:1) as a colorless oil in 73% yield (54 mg); d.r.83:17. (from crude NMR)
Compound **20e** was prepared according to the general procedure **GP2** and was obtained after column chromatography (*n*-hexane : ethyl acetate 20:1→9:1) as a colorless oil in 87% yield (65 mg); d.r.85:15. (from crude NMR)
Main product:
^{1}H NMR (600 MHz, $CDCl_3$): δ = 7.93 – 7.76 (m, 2H), 7.36 – 7.26 (m, 3H), 7.14 – 7.08 (m, 2H), 6.96 (s, 1H), 3.80 (d, *J* = 4.2 Hz, 1H), 2.91 (qd, *J* = 6.0, 4.2 Hz, 1H), 2.43 (s, 3H), 1.87 (d, *J* = 6.0 Hz, 3H) ppm.
^{13}C NMR (151 MHz, $CDCl_3$): δ = 149.4, 144.2, 138.1, 137.6, 129.9, 129.6, 127.2, 124.7, 120.5, 120.3 (q, *J* = 257.4 Hz), 118.6, 49.4, 48.0, 21.5, 14.0 ppm.
^{19}F NMR (565 MHz, $CDCl_3$): δ = -57.88 ppm.
HRMS (ESI): mass found: 394.06960, calculated mass for $C_{17}H_{16}F_3NO_3SNa^+$: 394.06952.
IR (KBr): 2979, 2931, 2667, 2327, 2176, 2105, 1997, 1916, 1595, 1492, 1450, 1407, 1324, 1253, 1215, 1156, 1089, 1042, 997, 958, 888, 810, 768, 686 cm^{-1}.

(2R,3R)-2-(2-Bromophenyl)-3-methyl-1-tosylaziridine (20f)

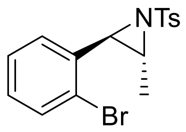

Compound **20f** was prepared according to the general procedure **GP1** and was obtained after ; column chromatography (*n*-hexane : ethyl acetate 20:1→9:1) as a colorless oil in 62% yield (46 mg); d.r.69:31.(from crude NMR)

Compound **20f** was prepared according to the general procedure **GP2** and was obtained after column chromatography (*n*-hexane : ethyl acetate 20:1→9:1) as a colorless oil in 72% yield (53 mg); d.r.74:26.(from crude NMR)

^{1}H NMR (600 MHz, $CDCl_3$): δ = 7.94 – 7.92 (m, 0.64H) and 7.92 – 7.90 (m, 2.00H), 7.55 – 7.52 (m, 0.35H) and 7.53 – 7.50 (m, 0.95H), 7.38 (d, *J* = 8.0 Hz, 0.73H) and 7.34 (d, *J* = 8.0 Hz, 2.03H), 7.25 – 7.18 (m, 0.74H) and 7.15 – 7.09 (m, 2.09H), 7.17 (dd, *J* = 7.5, 2.1 Hz, 0.30H) and 6.87 (dd, *J* = 7.0, 2.3 Hz, 1.00H), 4.02 (d, *J* = 4.3 Hz, 0.98H) and 4.00 (d, *J* = 7.3 Hz, 0.36H), 3.32 (dq, *J* = 11.6, 5.8 Hz, 0.36H) and 2.78 (qd, *J* = 6.0, 4.4 Hz, 1.01H), 2.47 (s, 1.10H) and 2.46 (s, 3.00H), 1.93 (d, *J* = 6.0 Hz, 3.06H) and 1.00 (d, *J* = 5.8 Hz, 1.06H) ppm.

^{13}C NMR (151 MHz, $CDCl_3$): δ = 144.6 and 144.2, 137.6 and 135.5, 135.1 and 132.8, 132.2 and 129.9, 129.8 and 129.6, 129.38 and 129.31, 127.9 and 127.5, 127.4 and 127.2, 123.1 and 122.9, 49.4 and 48.9, 47.0 and 41.8, 21.68 and 21.64, 14.0 and 12.0 ppm.

HRMS (ESI): mass found: 387.99766, calculated mass for $C_{16}H_{16}BrNO_2SNa^+$: 387.99773.

IR (KBr): 3062, 2976, 2929, 2326, 2177, 2086, 1921, 1810, 1596, 1472, 1441, 1409, 1323, 1236, 1157, 1089, 1030, 982, 890, 813, 752, 711, 681 cm^{-1}.

(2R,3R)-2-Ethyl-3-phenyl-1-tosylaziridine (20g)

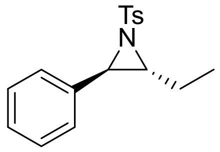

Compound **20g** was prepared according to the general procedure **GP1** and was obtained after column chromatography (*n*-hexane : ethyl acetate 20:1→9:1) as a colorless oil in 88% yield (53 mg); d.r.82:18.(from crude NMR)

Compound **20g** was prepared according to the general procedure **GP2** and was obtained after column chromatography (*n*-hexane : ethyl acetate 20:1→9:1) as a colorless oil in 93% yield (56 mg); d.r.88:12.(from crude NMR)

^{1}H NMR (600 MHz, $CDCl_3$): δ = 7.92 (d, *J* = 8.2 Hz, 0.26H) and 7.84 (d, *J* = 8.2 Hz, 1.95H), 7.32 – 7.24 (m, 5.80H), 7.36 (d, *J* = 8.0 Hz, 0.30H) and 7.17 (dd, *J* = 7.4, 1.8 Hz, 2.03H), 4.01 (d, *J* = 7.2 Hz, 0.13H) and 3.82 (d, *J* = 4.4 Hz, 0.99H), 3.04 (dd, *J* = 13.4, 7.4 Hz, 0.14H) and 2.85 (dt, *J* = 8.1, 5.2 Hz, 1.01H), 2.46 (s, 0.41H) and 2.41 (s, 3.00H), 2.36 – 2.24 (m, 1.06H) and 1.37 – 1.28 (m, 0.19H), 2.21 – 2.08 (m, 1.05H) and 1.28 – 1.22 (m, 0.17H), 1.19 (t, *J* = 7.5 Hz, 3.00H) and 0.81 (t, *J* = 7.5 Hz, 0.41H) ppm.

^{13}C NMR (151 MHz, $CDCl_3$): δ = 144.4 and 143.8, 137.8 and 135.5, 135.2 and 133.0, 129.6 and 129.5, 128.5 and 128.2, 128.06 and 128.02, 127.7 and 127.4, 127.2 and 126.4, 54.7 and 48.7, 47.7 and 46.0, 22.2 and 21.6, 21.5 and 19.8, 12.3 and 11.0 ppm.

HRMS (ESI): mass found: 324.10278, calculated mass for $C_{17}H_{19}NO_2SNa^+$: 324.10287.

IR (KBr): 3551, 3035, 2971, 2933, 2878, 2161, 2030, 1918, 1734, 1598, 1496, 1456, 1420, 1320, 1235, 1156, 1088, 1019, 903, 853, 812, 747, 691 cm^{-1}.

(2S,3S)-2,3-Dibutyl-1-tosylaziridine (20h)

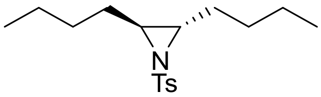

Compound **20h** was prepared from *trans*-5-Decen according to the general procedure **GP1** and was obtained after column chromatography (*n*-hexane : ethyl acetate 40:1→20:1) as a colorless oil in 71% yield (44 mg); d.r.73:27.(from crude NMR)

Compound **20h** was prepared from *trans*-5-Decen according to the general procedure **GP2** and was obtained after column chromatography (*n*-hexane : ethyl acetate 40:1→20:1) as a colorless oil in 76% yield (47 mg); d.r.75:25.(from crude NMR)

^{1}H NMR (600 MHz, $CDCl_3$): δ = 7.94 – 7.74 (m, 2.75H), 7.33 (t, *J* = 8.7 Hz, 2.81H), 2.84 – 2.73 (m, 0.70H) and 2.71 – 2.60 (m, 2.05H), 2.46 (s, 1.04H) and 2.45 (s, 3.00H), 1.83 – 1.65 (m, 4.31H), 1.54 – 1.46 (m, 0.89H), 1.46 – 1.18 (m, 12.65H), 1.10 – 0.78 (m, 8.85H) ppm.

^{13}C NMR (151 MHz, $CDCl_3$): δ = 144.1 and 143.7, 138.0 and 135.4, 129.4 and 129.3, 128.0 and 127.4, 49.8 and 45.2, 29.6 and 29.5, 29.4 and 26.4, 22.28 and 22.24, 21.58 and 21.56, 13.87 and 13.86 ppm.

HRMS (ESI): mass found: 332.16528, calculated mass for $C_{17}H_{27}NO_2SNa^+$: 332.16547.

IR (KBr): 3432, 2928, 2865, 2322, 2159, 2079, 1910, 1715, 1673, 1616, 1494, 1459, 1377, 1321, 1241, 1157, 1090, 1015, 951, 894, 813, 690 cm^{-1}.

(2S,3S)-2-Isopropyl-3-methyl-1-tosylaziridine (20i)

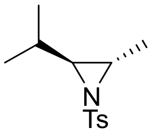

Compound **20i** was prepared according to the general procedure **GP1** and was obtained after column chromatography (*n*-hexane : ethyl acetate 20:1→9:1) as a colorless oil in 54% yield (27 mg); d.r.75:25.

Compound **20i** was prepared according to the general procedure **GP2** and was obtained after column chromatography (*n*-hexane : ethyl acetate 20:1→9:1) as a colorless oil in 68% yield (34 mg); d.r.80:20.

^{1}H NMR (400 MHz, $CDCl_3$): δ = 7.81 (t, *J* = 8.6 Hz, 2.22H), 7.29 (t, *J* = 7.2 Hz, 2.32H), 2.99 – 2.82 (m, 0.26H) and 2.73 – 2.59 (m, 0.94H), 2.51 (dd, *J* = 7.9, 4.6 Hz, 0.95H), 2.39 (d, *J* = 11.7 Hz, 4.01H), 1.62 (d, *J* = 6.0 Hz, 3.08H), 1.55 – 1.33 (m, 1.27H), 1.20 (d, *J* = 5.9 Hz, 0.82H), 0.89 (d, *J* = 6.8 Hz, 0.84H) and 0.86 (d, *J* = 6.8 Hz, 3.06H), 0.75 (d, *J* = 6.6 Hz, 0.81H) and 0.71 (d, *J* = 6.7 Hz, 3.00H) ppm.

^{13}C NMR (101 MHz, $CDCl_3$): δ = 144.1 and 143.7, 137.9 and 135.4, 129.8 and 129.3, 127.9 and 127.5, 55.0 and 51.4, 45.4 and 40.3, 30.1 and 26.3, 21.5 and 21.5, 20.7 and 19.8, 19.4 and 19.1, 14.3 and 11.9 ppm.

HRMS (ESI): mass found: 276.10229, calculated mass for $C_{13}H_{19}NO_2SNa^+$: 276.10287.

IR (KBr): 3854, 3632, 2963, 2874, 2730, 2325, 2179, 2092, 1992, 1808, 1599, 1460, 1387, 1317, 1246, 1155, 1089, 1057, 1024, 992, 947, 876, 814, 739, 708, 681 cm^{-1}.

2-Phenyl-1-tosylaziridine (22a)

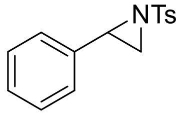

Compound **22a** was prepared according to the general procedure **GP1** and was obtained after column chromatography (*n*-hexane : ethyl acetate 20:1→9:1) as a white solid in 84% yield (46 mg).
Compound **22a** was prepared according to the general procedure **GP2** and was obtained after column chromatography (*n*-hexane : ethyl acetate 20:1→9:1) as a white solid in 90% yield (49 mg).
^{1}H NMR (400 MHz, $CDCl_3$): δ = 7.91 – 7.84 (m, 2H), 7.37 – 7.26 (m, 5H), 7.25 – 7.19 (m, 2H), 3.78 (dd, *J* = 7.2, 4.5 Hz, 1H), 2.99 (d, *J* = 7.2 Hz, 1H), 2.43 (s, 3H), 2.39 (d, *J* = 4.5 Hz, 1H) ppm.
^{13}C NMR (101 MHz, $CDCl_3$): δ = 144.6, 135.0, 134.9, 129.7, 128.5, 128.2, 127.9, 126.5, 41.0, 35.9, 21.6 ppm.
HRMS (ESI): mass found: 296.07123, calculated mass for $C_{15}H_{15}NO_2SNa^+$: 296.07157.
IR (KBr): 3482, 3037, 2926, 2859, 2586, 2318, 2040, 1896, 1815, 1727, 1687, 1595, 1494, 1456, 1387, 1319, 1232, 1190, 1156, 1117, 1091, 1028, 970, 908, 804, 780, 753, 691, 663 cm^{-1}.

1-((4-Chlorophenyl)sulfonyl)-2-phenylaziridine (22b)

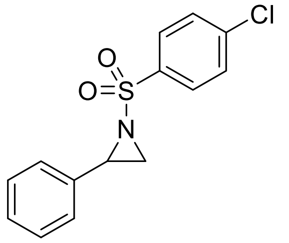

Compound **22b** was prepared according to the general procedure **GP1** and was obtained after column chromatography (*n*-hexane : ethyl acetate 20:1→9:1) as a white solid in 78% yield (46 mg).
Compound **22b** was prepared according to the general procedure **GP2** and was obtained after column chromatography (*n*-hexane : ethyl acetate 20:1→9:1) as a white solid in 87% yield (51 mg).
^{1}H NMR (400 MHz, $CDCl_3$): δ = 7.95 – 7.83 (m, 2H), 7.54 – 7.45 (m, 2H), 7.33 – 7.26 (m, 3H), 7.23 – 7.16 (m, 2H), 3.80 (dd, *J* = 7.2, 4.5 Hz, 1H), 3.01 (d, *J* = 7.2 Hz, 1H), 2.41 (d, *J* = 4.5 Hz, 1H) ppm.
^{13}C NMR (101 MHz, $CDCl_3$): δ = 140.3, 136.5, 134.6, 129.4, 129.3, 128.6, 128.4, 126.4, 41.3, 36.1 ppm.
HRMS (ESI): mass found: 316.01542, calculated mass for $C_{14}H_{12}NClO_2SNa^+$: 316.01695.
IR (KBr): 3095, 3038, 2926, 2858, 2323, 2165, 2023, 1918, 1729, 1582, 1477, 1393, 1327, 1280, 1237, 1160, 1085, 1014, 937, 910, 830, 803, 761, 699 cm^{-1}.

1-((3,5-*bis*(Trifluoromethyl)phenyl)sulfonyl)-2-phenylaziridine (22c)

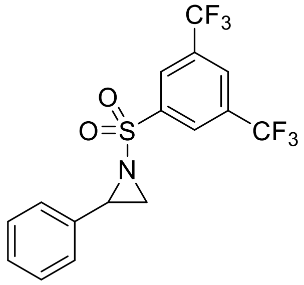

Compound **22c** was prepared according to the general procedure **GP1** and was obtained after column chromatography (*n*-hexane : ethyl acetate 20:1→9:1) as a white solid in 77% yield (61 mg).
Compound **22c** was prepared according to the general procedure **GP2** and was obtained after column chromatography (*n*-hexane : ethyl acetate 20:1→9:1) as a white solid in 80% yield (63 mg).
^{1}H NMR (600 MHz, $CDCl_3$): δ = 8.47 (s, 2H), 8.15 (s, 1H), 7.40 – 7.33 (m, 3H), 7.31 – 7.18 (m, 2H), 4.00 – 3.97 (m, 1H), 3.16 (d, *J* = 7.3 Hz, 1H), 2.59 (d, *J* = 4.6 Hz, 1H) ppm.
^{13}C NMR (151 MHz, $CDCl_3$): δ = 141.2, 133.9, 133.0 (q, *J* = 34.9 Hz), 128.89, 128.81, 128.1 (q, *J* = 3.2 Hz), 127.2 (m), 126.5, 122.3 (q, *J* = 273.4 Hz, 1H), 42.0, 36.8 ppm.
^{19}F NMR (565 MHz, $CDCl_3$): δ = -62.98 ppm.
HRMS (ESI): mass found: 396.04849, calculated mass for $C_{16}H_{12}NF_6O_2S^+$: 396.04875.
IR (KBr): 3085, 2927, 2859, 2165, 1835, 1734, 1607, 1498, 1459, 1331, 1273, 1116, 1029, 976, 906, 838, 770, 726, 692 cm^{-1}.

1-((3-Nitrophenyl)sulfonyl)-2-phenylaziridine (22d)

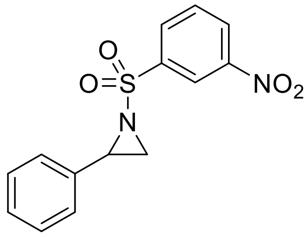

Compound **22d** was prepared according to the general procedure **GP1** and was obtained after column chromatography (*n*-hexane : ethyl acetate 9:1→4:1) as a white oil in 82% yield (50 mg).
Compound **22d** was prepared according to the general procedure **GP2** and was obtained after column chromatography (*n*-hexane : ethyl acetate 9:1→4:1) as a white oil in 85% yield (52 mg).
^{1}H NMR (600 MHz, $CDCl_3$): δ = 8.86 (s, 1H), 8.51 (d, *J* = 8.2 Hz, 1H), 8.35 (dd, *J* = 7.8, 0.8 Hz, 1H), 7.80 (t, *J* = 8.0 Hz, 1H), 7.53 – 7.29 (m, 3H), 7.28 – 7.08 (m, 2H), 3.95 (dd, *J* = 7.0, 4.8 Hz, 1H), 3.14 (d, *J* = 7.2 Hz, 1H), 2.54 (d, *J* = 4.6 Hz, 1H) ppm.
^{13}C NMR (151 MHz, $CDCl_3$): δ = 148.2, 140.5, 134.1, 133.3, 130.6, 128.77, 128.75, 128.1, 126.5, 123.1, 41.8, 36.6 ppm.
HRMS (ESI): mass found: 305.05882, calculated mass for $C_{14}H_{13}N_2O_4S^+$: 305.05905.

IR (KBr): 3464, 3090, 2932, 2872, 2663, 2332, 2160, 2079, 1985, 1887, 1811, 1726, 1605, 1533, 1498, 1459, 1347, 1234, 1169, 1124, 1081, 1029, 978, 908, 812, 775, 736, 695, 664 cm^{-1}.

1-((2-Nitrophenyl)sulfonyl)-2-phenylaziridine (22e)

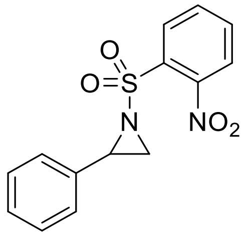

Compound **22e** was prepared according to the general procedure **GP1** and was obtained after column chromatography (*n*-hexane : ethyl acetate 9:1→4:1) as a white oil in 76% yield (40 mg).
Compound **22e** was prepared according to the general procedure **GP2** and was obtained after column chromatography (*n*-hexane : ethyl acetate 9:1→4:1) as a white oil in 79% yield (48 mg).
^{1}H NMR (600 MHz, $CDCl_3$): δ = 8.86 (s, 1H), 8.59 – 8.42 (m, 1H), 8.38 – 8.23 (m, 1H), 7.79 (t, *J* = 8.0 Hz, 1H), 7.36 – 7.29 (m, 3H), 7.26 (d, *J* = 7.8 Hz, 2H), 3.95 (dd, *J* = 7.0, 4.8 Hz, 1H), 3.14 (d, *J* = 7.2 Hz, 1H), 2.54 (d, *J* = 4.6 Hz, 1H) ppm.
^{13}C NMR (151 MHz, $CDCl_3$): δ = 148.2, 140.4, 134.1, 133.3, 130.6, 128.77, 128.75, 128.1, 126.5, 123.1, 41.8, 36.6 ppm.
HRMS (ESI): mass found: 305.05879, calculated mass for $C_{14}H_{13}N_2O_4S^+$: 305.05905.
IR (KBr): 3548, 3089, 2928, 2872, 2333, 2162, 1972, 1888, 1812, 1727, 1605, 1532, 1498, 1460, 1384, 1347, 1276, 1233, 1168, 1123, 1079, 979, 908, 812, 775, 736, 695, 664 cm^{-1}.

2-Phenyl-1-(*o*-tolylsulfonyl)aziridine (22f)

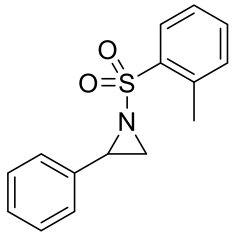

Compound **22f** was prepared according to the general procedure **GP1** and was obtained after column chromatography (*n*-hexane : ethyl acetate 20:1→9:1) as a colorless oil in 84% yield (46 mg).
Compound **22f** was prepared according to the general procedure **GP2** and was obtained after column chromatography (*n*-hexane : ethyl acetate 20:1→9:1) as a colorless oil in 89% yield (49 mg).
^{1}H NMR (600 MHz, $CDCl_3$): δ = 8.00 (d, *J* = 7.9 Hz, 1H), 7.52 (t, *J* = 7.5 Hz, 1H), 7.39 – 7.29 (m, 5H), 7.23 (d, *J* = 7.7 Hz, 2H), 3.84 (dd, *J* = 7.0, 4.6 Hz, 1H), 3.06 (d, *J* = 7.2 Hz, 1H), 2.83 (s, 3H), 2.41 (d, *J* = 4.4 Hz, 1H) ppm.
^{13}C NMR (151 MHz, $CDCl_3$): δ = 139.1, 136.4, 135.3, 133.60, 132.61, 129.2, 128.6, 128.3, 126.4, 126.0, 40.9, 36.2, 20.6 ppm.

HRMS (ESI): mass found: 274.08942, calculated mass for $C_{15}H_{16}NO_2S^+$: 274.08963.
IR (KBr): 3437, 3064, 3033, 2960, 2933, 2870, 2332, 2161, 2035, 1973, 1699, 1634, 1599, 1496, 1458, 1379, 1319, 1231, 1190, 1160, 1063, 978 cm^{-1}.

1-([1,1'-Biphenyl]-4-ylsulfonyl)-2-phenylaziridine (22g)

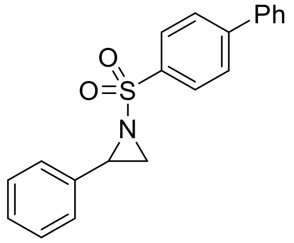

Compound **22g** was prepared according to the general procedure **GP1** and was obtained after column chromatography (*n*-hexane : ethyl acetate 20:1→9:1) as a white solid in 57% yield (38 mg).
Compound **22g** was prepared according to the general procedure **GP2** and was obtained after column chromatography (*n*-hexane : ethyl acetate 20:1→9:1) as a white solid in 65% yield (44 mg).
^{1}H NMR (600 MHz, $CDCl_3$): δ = 8.08 (d, *J* = 8.1 Hz, 2H), 7.77 (d, *J* = 8.1 Hz, 2H), 7.63 (d, *J* = 8.0 Hz, 2H), 7.51 (t, *J* = 7.4 Hz, 2H), 7.45 (t, *J* = 7.3 Hz, 1H), 7.36 – 7.30 (m, 3H), 7.29 – 7.20 (m, 2H), 3.87 (dd, *J* = 6.9, 4.7 Hz, 1H), 3.07 (d, *J* = 7.2 Hz, 1H), 2.47 (d, *J* = 4.5 Hz, 1H) ppm.
^{13}C NMR (151 MHz, $CDCl_3$): δ = 146.5, 139.2, 136.5, 134.9, 129.0, 128.65, 128.62, 128.44, 128.40, 127.7, 127.3, 126.6, 41.2, 36.0 ppm.
HRMS (ESI): mass found: 336.10491, calculated mass for $C_{20}H_{18}NO_2S^+$: 336.10528.
IR (KBr): 3842, 3451, 3064, 3034, 2925, 2858, 2668, 2326, 2201, 2087, 2028, 1996, 1943, 1729, 1595, 1456, 1385, 1327, 1162, 1126, 1095, 1030, 877, 909, 842, 761, 696 cm^{-1}.

1-(Naphthalen-2-ylsulfonyl)-2-phenylaziridine (22h)

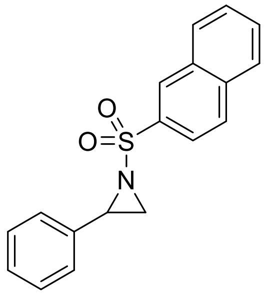

Compound **22h** was prepared according to the general procedure **GP1** and was obtained after column chromatography (*n*-hexane : ethyl acetate 20:1→9:1) as a white solid in 68% yield (42 mg).
Compound **22h** was prepared according to the general procedure **GP2** and was obtained after column chromatography (*n*-hexane : ethyl acetate 20:1→9:1) as a white solid in 74% yield (46 mg).
^{1}H NMR (600 MHz, $CDCl_3$): δ = 8.57 (s, 1H), 8.01 (d, *J* = 6.8 Hz, 3H), 7.95 (d, *J* = 8.2 Hz, 1H), 7.69 (t, *J* = 7.5 Hz, 1H), 7.65 (t, *J* = 7.5 Hz, 1H), 7.30 (t, *J* = 6.9 Hz, 3H), 7.26 (d, *J* = 7.8 Hz, 2H), 3.90 (dd, *J* = 6.9, 4.6 Hz, 1H), 3.09 (d, *J* = 7.2 Hz, 1H), 2.46 (d, *J* = 4.4 Hz, 1H) ppm.

^{13}C NMR (151 MHz, $CDCl_3$): δ = 135.3, 134.9, 134.9, 132.0, 129.4, 129.4, 129.2, 128.6, 128.3, 127.9, 127.6, 126.5, 122.9, 41.2, 36.1 ppm.
HRMS (ESI): mass found: 310.08916, calculated mass for $C_{18}H_{16}NO_2S^+$: 310.08963.
IR (KBr): 3525, 3060, 2928, 2861, 2159, 2027, 1939, 1814, 1727, 1591, 1499, 1458, 1384, 1320, 1235, 1189, 1156, 1077, 1029, 976, 906, 860, 816, 750, 698, 661 cm^{-1}.

2-(*p*-Tolyl)-1-tosylaziridine (22i)

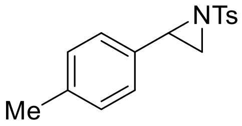

Compound **22i** was prepared according to the general procedure **GP1** and was obtained after column chromatography (*n*-hexane : ethyl acetate 20:1→9:1) as a white solid in 77% yield (44 mg).
Compound **22i** was prepared according to the general procedure **GP2** and was obtained after column chromatography (*n*-hexane : ethyl acetate 20:1→9:1) as a white solid in 84% yield (48 mg).
^{1}H NMR (400 MHz, $CDCl_3$): δ = 7.84 (d, *J* = 8.3 Hz, 2H), 7.30 (d, *J* = 8.0 Hz, 2H), 7.08 (s, 4H), 3.72 (dd, *J* = 7.2, 4.5 Hz, 1H), 2.95 (d, *J* = 7.2 Hz, 1H), 2.41 (s, 3H), 2.36 (d, *J* = 4.5 Hz, 1H), 2.29 (s, 3H) ppm.
^{13}C NMR (101 MHz, $CDCl_3$): δ = 144.5, 138.1, 135.0, 131.9, 129.6, 129.2, 127.8, 126.4, 41.0, 35.7, 21.6, 21.1 ppm.
HRMS (ESI): mass found: 288.10394, calculated mass for $C_{14}H_{19}NO_2SNa^+$: 288.10287.
IR (KBr): 3484, 2923, 2858, 2305, 2167, 2031, 1910, 1810, 1730, 1596, 1515, 1454, 1381, 1318, 1184, 1157, 1092, 1022, 974, 910, 811, 716, 661 cm^{-1}.

2-(4-(*tert*-Butyl)phenyl)-1-tosylaziridine (22j)

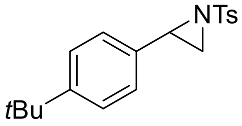

Compound **22j** was prepared according to the general procedure **GP1** and was obtained after column chromatography (*n*-hexane : ethyl acetate 20:1→9:1) as a colorless oil in 76% yield (50 mg).
Compound **22j** was prepared according to the general procedure **GP2** and was obtained after column chromatography (*n*-hexane : ethyl acetate 20:1→9:1) as a colorless oil in 82% yield (54 mg).
^{1}H NMR (400 MHz, $CDCl_3$): δ = 7.85 (d, *J* = 8.3 Hz, 2H), 7.39 – 7.27 (m, 4H), 7.19 – 7.08 (m, 2H), 3.75 (dd, *J* = 7.2, 4.5 Hz, 1H), 2.95 (d, *J* = 7.2 Hz, 1H), 2.42 (s, 3H), 2.37 (d, *J* = 4.5 Hz, 1H), 1.27 (s, 9H) ppm.
^{13}C NMR (101 MHz, $CDCl_3$): δ = 151.3, 144.5, 135.0, 131.9, 129.7, 127.9, 126.2, 125.4, 40.9, 35.7, 34.5, 31.2, 21.6 ppm.
HRMS (ESI): mass found: 330.15264, calculated mass for $C_{19}H_{24}NO_2S^+$: 330.15223.
IR (KBr): 3450, 2959, 2869, 2162, 2049, 1913, 1726, 1599, 1513, 1460, 1363, 1326, 1270, 1236, 1188, 1160, 1093, 1019, 980, 910, 816, 745, 714, 670 cm^{-1}.

1-Tosyl-2-(4-(trifluoromethyl)phenyl)aziridine (22k)

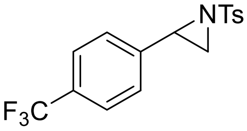

Compound **22k** was prepared according to the general procedure **GP1** and was obtained after column chromatography (*n*-hexane : ethyl acetate 20:1→9:1) as a white solid in 82% yield (56 mg).

Compound **22k** was prepared according to the general procedure **GP2** and was obtained after column chromatography (*n*-hexane : ethyl acetate 20:1→9:1) as a white solid in 88% yield (60 mg).

^{1}H NMR (600 MHz, $CDCl_3$): δ = 7.94 – 7.72 (m, 2H), 7.48 (d, *J* = 8.2 Hz, 2H), 7.27 (d, *J* = 8.4 Hz, 4H), 3.73 (dd, *J* = 7.2, 4.3 Hz, 1H), 2.95 (d, *J* = 7.2 Hz, 1H), 2.37 (s, 3H), 2.29 (d, *J* = 4.3 Hz, 1H) ppm.

^{13}C NMR (151 MHz, $CDCl_3$): δ = 144.9, 139.2, 134.7, 130.5 (q, *J* = 32.6 Hz), 129.8, 127.9, 126.9, 125.5 (q, *J* = 3.6 Hz), 123.9 (q, *J* = 272.0 Hz), 40.1, 36.2, 21.6 ppm.

^{19}F NMR (565 MHz, $CDCl_3$): δ = -62.68 ppm.

HRMS (ESI): mass found: 342.07718, calculated mass for $C_{16}H_{15}NF_3O_2S^+$: 342.07701.

IR (KBr): 2925, 2859, 2164, 2029, 1925, 1807, 1732, 1621, 1594, 1493, 1455, 1423, 1388, 1315, 1239, 1158, 1118, 1064, 1015, 977, 915, 845, 814, 750, 706, 661 cm^{-1}.

2-(4-Fluorophenyl)-1-tosylaziridine (22l)

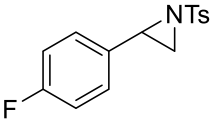

Compound **22l** was prepared according to the general procedure **GP1** and was obtained after column chromatography (*n*-hexane : ethyl acetate 20:1→9:1) as a white solid in 84% yield (49 mg).

Compound **22l** was prepared according to the general procedure **GP2** and was obtained after column chromatography (*n*-hexane : ethyl acetate 20:1→9:1) as a white solid in 92% yield (54 mg).

^{1}H NMR (400 MHz, $CDCl_3$): δ = 7.84 (d, *J* = 8.2 Hz, 2H), 7.32 (d, *J* = 8.0 Hz, 2H), 7.25 – 7.08 (m, 2H), 7.03 – 6.84 (m, 2H), 3.73 (dd, *J* = 7.1, 4.5 Hz, 1H), 2.95 (d, *J* = 7.2 Hz, 1H), 2.42 (s, 3H), 2.33 (d, *J* = 4.4 Hz, 1H) ppm.

^{13}C NMR (101 MHz, $CDCl_3$): δ = 162.6 (d, *J* = 247.3 Hz), 144.7, 134.8, 130.8 (d, *J* = 2.8 Hz), 129.7, 128.2 (d, *J* = 8.4 Hz), 127.9, 115.53 (d, *J* = 21.7 Hz), 40.2, 35.9, 21.6 ppm.

^{19}F NMR (376 MHz, $CDCl_3$): δ = -113.45 – -113.46 ppm.

HRMS (ESI): mass found: 292.08053, calculated mass for $C_{15}H_{15}NFO_2S^+$: 292.08020.

IR (KBr): 3068, 2927, 2863, 2176, 2043, 1989, 1897, 1729, 1601, 1512, 1455, 1380, 1324, 1230, 1187, 1158, 1092, 1018, 981, 909, 817, 715, 663 cm^{-1}.

2-(4-Chlorophenyl)-1-tosylaziridine (22m)

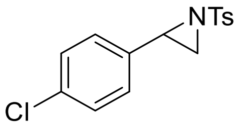

Compound **22m** was prepared according to the general procedure **GP1** and was obtained after column chromatography (*n*-hexane : ethyl acetate 20:1→9:1) as a white solid in 81% yield (50 mg).

Compound **22m** was prepared according to the general procedure **GP2** and was obtained after column chromatography (*n*-hexane : ethyl acetate 20:1→9:1) as a white solid in 88% yield (54 mg).

^{1}H NMR (400 MHz, $CDCl_3$): δ = 7.84 (d, *J* = 8.3 Hz, 2H), 7.31 (d, *J* = 8.0 Hz, 2H), 7.27 – 7.20 (m, 2H), 7.16 – 7.05 (m, 2H), 3.71 (dd, *J* = 7.1, 4.4 Hz, 1H), 2.96 (d, *J* = 7.2 Hz, 1H), 2.42 (s, 3H), 2.32 (d, *J* = 4.4 Hz, 1H) ppm.

^{13}C NMR (101 MHz, $CDCl_3$): δ = 144.7, 134.7, 134.1, 133.6, 129.7, 128.7, 127.9, 127.8, 40.2, 36.0, 21.6 ppm.

HRMS (ESI): mass found: 330.03096, calculated mass for $C_{15}H_{14}NClO_2SNa^+$: 330.03260.

IR (KBr): 3462, 2924, 2857, 2302, 2169, 1926, 1730, 1594, 1491, 1453, 1405, 1376, 1319, 1226, 1185, 1157, 1089, 1014, 977, 909, 815, 730, 696, 668 cm^{-1}.

2-(4-Bromophenyl)-1-tosylaziridine (22n)

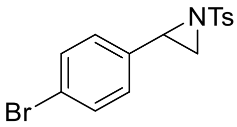

Compound **22n** was prepared according to the general procedure **GP1** and was obtained after column chromatography (*n*-hexane : ethyl acetate 20:1→9:1) as a white solid in 77% yield (54 mg).

Compound **22n** was prepared according to the general procedure **GP2** and was obtained after column chromatography (*n*-hexane : ethyl acetate 20:1→9:1) as a white solid in 85% yield (60 mg).

^{1}H NMR (400 MHz, $CDCl_3$): δ = 7.83 (d, *J* = 8.3 Hz, 2H), 7.46 – 7.36 (m, 2H), 7.32 (d, *J* = 8.0 Hz, 2H), 7.16 – 6.97 (m, 2H), 3.70 (dd, *J* = 7.1, 4.4 Hz, 1H), 2.96 (d, *J* = 7.2 Hz, 1H), 2.42 (s, 3H), 2.32 (d, *J* = 4.4 Hz, 1H) ppm.

^{13}C NMR (101 MHz, $CDCl_3$): δ = 144.7, 134.7, 134.1, 131.6, 129.7, 128.1, 127.9, 122.2, 40.2, 35.9, 21.6 ppm.

HRMS (ESI): mass found: 373.98253, calculated mass for $C_{15}H_{14}NBrO_2SNa^+$: 373.98208.

IR (KBr): 3444, 2926, 2859, 2296, 2177, 2019, 1901, 1724, 1591, 1487, 1456, 1405, 1317, 1221, 1185, 1156, 1091, 1071, 1009, 979, 904, 814, 726, 693, 663 cm^{-1}.

4-(1-Tosylaziridin-2-yl)phenyl acetate (22o)

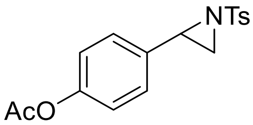

Compound **22o** was prepared according to the general procedure **GP1** and was obtained after column chromatography (*n*-hexane : ethyl acetate 20:1→9:1) as a white solid in 88% yield (58 mg).
Compound **22o** was prepared according to the general procedure **GP2** and was obtained after column chromatography (*n*-hexane : ethyl acetate 20:1→9:1) as a white solid in 89% yield (59 mg).
^{1}H NMR (600 MHz, $CDCl_3$): δ = 7.95 (d, *J* = 8.3 Hz, 2H), 7.86 (d, *J* = 8.2 Hz, 2H), 7.33 (d, *J* = 8.0 Hz, 2H), 7.28 (d, *J* = 8.2 Hz, 2H), 3.89 (s, 3H), 3.79 (dd, *J* = 7.1, 4.4 Hz, 1H), 3.01 (d, *J* = 7.2 Hz, 1H), 2.43 (s, 3H), 2.38 (d, *J* = 4.3 Hz, 1H) ppm.
^{13}C NMR (151 MHz, $CDCl_3$): δ = 166.5, 144.8, 140.1, 134.6, 130.0, 129.8, 129.8, 127.9, 126.5, 52.1, 40.4, 36.2, 21.6 ppm.
HRMS (ESI): mass found: 332.09547, calculated mass for $C_{17}H_{18}NO_4S^+$: 332.09511.
IR (KBr): 3449, 2952, 2857, 2730, 2160, 2037, 1934, 1718, 1611, 1507, 1436, 1323, 1278, 1185, 1160, 1103, 1020, 973, 908, 858, 815, 770, 718, 695, 666 cm^{-1}.

2-(*m*-Tolyl)-1-tosylaziridine (22p)

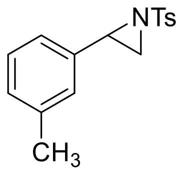

Compound **22p** was prepared according to the general procedure **GP1** and was obtained after column chromatography (*n*-hexane : ethyl acetate 20:1→9:1) as a colorless oil in 75% yield (43 mg).
Compound **22p** was prepared according to the general procedure **GP2** and was obtained after column chromatography (*n*-hexane : ethyl acetate 20:1→9:1) as a colorless oil in 79% yield (46 mg).
^{1}H NMR (400 MHz, $CDCl_3$): δ = 7.85 (d, *J* = 8.3 Hz, 2H), 7.31 (d, *J* = 8.0 Hz, 2H), 7.20 – 7.12 (m, 1H), 7.06 (d, *J* = 7.5 Hz, 1H), 7.00 (d, *J* = 6.1 Hz, 2H), 3.73 (dd, *J* = 7.2, 4.5 Hz, 1H), 2.94 (d, *J* = 7.2 Hz, 1H), 2.42 (s, 3H), 2.36 (d, *J* = 4.5 Hz, 1H), 2.28 (s, 3H) ppm.
^{13}C NMR (101 MHz, $CDCl_3$): δ = 144.5, 138.2, 134.9, 134.9, 129.7, 129.0, 128.4, 127.9, 127.1, 123.6, 41.0, 35.8, 21.6, 21.2 ppm.
HRMS (ESI): mass found: 288.10555, calculated mass for $C_{16}H_{18}NO_2S^+$: 288.10528.
IR (KBr): 3259, 3029, 2924, 2864, 2324, 2167, 2087, 1992, 1929, 1728, 1598, 1490, 1455, 1378, 1324, 1247, 1214, 1159, 1091, 1035, 978, 928, 867, 813, 786, 720, 694, 666 cm^{-1}.

1-Tosyl-2-(3-(trifluoromethyl)phenyl)aziridine (22q)

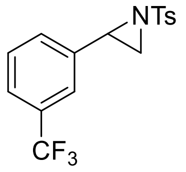

Compound **22q** was prepared according to the general procedure **GP1** and was obtained after column chromatography (*n*-hexane : ethyl acetate 20:1→9:1) as a white solid in 75% yield (51 mg).
Compound **22q** was prepared according to the general procedure **GP2** and was obtained after column chromatography (*n*-hexane : ethyl acetate 20:1→9:1) as a white solid in 82% yield (56 mg).
^{1}H NMR (600 MHz, $CDCl_3$): δ = 7.95 – 7.74 (m, 2H), 7.47 – 7.43 (m, 1H), 7.42 – 7.29 (m, 3H), 7.28 (d, *J* = 8.0 Hz, 2H), 3.73 (dd, *J* = 7.2, 4.4 Hz, 1H), 2.93 (d, *J* = 7.2 Hz, 1H), 2.37 (s, 3H), 2.31 (d, *J* = 4.4 Hz, 1H) ppm.
^{13}C NMR (151 MHz, $CDCl_3$): δ = 145.0, 136.3, 134.6, 131.0 (q, *J* = 32.6 Hz), 130.0, 129.8, 129.1, 128.0, 125.1 (q, *J* = 3.5 Hz), 123.8 (q, *J* = 272.1 Hz), 123.4 (q, *J* = 3.9 Hz), 40.1, 36.1, 21.6 ppm.
^{19}F NMR (565 MHz, $CDCl_3$): δ = -62.78 ppm.
HRMS (ESI): mass found: 342.07770, calculated mass for $C_{16}H_{15}NF_3O_2S^+$: 342.07701.
IR (KBr): 3473, 3068, 2929, 2862, 2163, 1919, 1728, 1597, 1493, 1452, 1381, 1324, 1231, 1159, 1123, 1074, 983, 920, 845, 806, 719, 700, 672 cm^{-1}.

2-(3-Nitrophenyl)-1-tosylaziridine (22r)

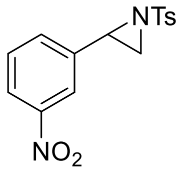

Compound **22r** was prepared according to the general procedure **GP1** and was obtained after column chromatography (*n*-hexane : ethyl acetate 20:1→9:1) as a colorless oil in 47% yield (30 mg).
Compound **22r** was prepared according to the general procedure **GP2** and was obtained after column chromatography (*n*-hexane : ethyl acetate 20:1→9:1) as a colorless oil in 54% yield (32 mg).
^{1}H NMR (600 MHz, $CDCl_3$): δ = 8.10 (d, *J* = 8.1 Hz, 1H), 7.90 (d, *J* = 8.2 Hz, 2H), 7.57 (d, *J* = 4.1 Hz, 2H), 7.48 – 7.43 (m, 1H), 7.37 (d, *J* = 8.1 Hz, 2H), 4.33 (dd, *J* = 7.4, 4.4 Hz, 1H), 3.06 (d, *J* = 7.5 Hz, 1H), 2.46 (s, 3H), 2.26 (d, *J* = 4.3 Hz, 1H) ppm.
^{13}C NMR (151 MHz, $CDCl_3$): δ = 148.0, 145.0, 134.1, 134.0, 131.3, 129.9, 129.4, 129.0, 128.1, 124.7, 39.3, 35.9, 21.7 ppm.
HRMS (ESI): mass found: 341.05660, calculated mass for $C_{15}H_{14}N_2O_4SNa^+$: 341.05665.
IR (KBr): 3290, 3070, 2926, 2890, 2723, 2163, 1727, 1602, 1525, 1451, 1340, 1231, 1160, 1044, 1091, 1044, 982, 915, 857, 815, 791, 722, 697, 670 cm^{-1}.

2-(2-Fluorophenyl)-1-tosylaziridine (22s)

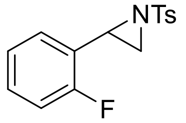

Compound **22s** was prepared according to the general procedure **GP1** and was obtained after column chromatography (*n*-hexane : ethyl acetate 20:1→9:1) as a colorless oil in 77% yield (45 mg).
Compound **22s** was prepared according to the general procedure **GP2** and was obtained after column chromatography (*n*-hexane : ethyl acetate 20:1→9:1) as a colorless oil in 80% yield (47 mg).
^{1}H NMR (400 MHz, $CDCl_3$): δ = 7.86 (d, *J* = 8.3 Hz, 2H), 7.32 (d, *J* = 8.0 Hz, 2H), 7.26 – 7.18 (m, 1H), 7.11 (td, *J* = 7.5, 1.8 Hz, 1H), 7.06 – 6.91 (m, 2H), 3.96 (dd, *J* = 7.2, 4.4 Hz, 1H), 2.99 (d, *J* = 7.2 Hz, 1H), 2.42 (s, 3H), 2.38 (d, *J* = 4.4 Hz, 1H) ppm.
^{13}C NMR (101 MHz, $CDCl_3$): δ = 161.3 (d, *J* = 248.0 Hz), 144.7, 134.6, 129.7 (d, *J* = 7.8 Hz), 129.7, 128.0, 127.4 (d, *J* = 3.2 Hz), 124.2 (d, *J* = 3.7 Hz), 122.4 (d, *J* = 13.4 Hz), 115.2 (d, *J* = 21.0 Hz), 35.57 (d, *J* = 5.4 Hz), 35.09, 21.64 ppm.
^{19}F NMR (376 MHz, $CDCl_3$): δ = -119.41 – -119.50 ppm.
HRMS (ESI): mass found: 292.07994, calculated mass for $C_{15}H_{15}NFO_2S^+$: 292.07916.
IR (KBr): 3546, 3064, 2927, 2863, 2170, 2049, 1922, 1806, 1730, 1593, 1495, 1455, 1386, 1326, 1244, 1212, 1159, 1092, 1034, 980, 910, 815, 759, 696, 663 cm^{-1}.

2-(2,6-Dichlorophenyl)-1-tosylaziridine (22t)

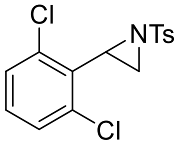

Compound **22t** was prepared according to the general procedure **GP1** and was obtained after column chromatography (n-hexane : ethyl acetate 20:1→9:1) as a colorless oil in 69% yield (47 mg).
Compound **22t** was prepared according to the general procedure **GP2** and was obtained after column chromatography (n-hexane : ethyl acetate 20:1→9:1) as a colorless oil in 75% yield (52 mg).
^{1}H NMR (600 MHz, $CDCl_3$): δ = 7.84 (d, *J* = 8.3 Hz, 2H), 7.28 (d, J = 8.0 Hz, 2H), 7.17 (d, *J* = 7.9 Hz, 2H), 7.10 – 6.99 (m, 1H), 3.81 (dd, *J* = 7.2, 4.6 Hz, 1H), 3.06 (d, *J* = 7.2 Hz, 1H), 2.47 (d, *J* = 4.6 Hz, 1H), 2.39 (s, 3H) ppm.
^{13}C NMR (151 MHz, $CDCl_3$): δ = 144.7, 136.1, 134.9, 130.5, 129.8, 129.6, 128.69, 128.62, 39.6, 34.9, 21.6 ppm.
HRMS (ESI): mass found: 342.01153, calculated mass for $C_{15}H_{14}NCl_2O_2S^+$: 342.01168.
IR (KBr): 3508, 3070, 2926, 2859, 2163, 2044, 1988, 1925, 1728, 1592, 1562, 1493, 1432, 1328, 1238, 1188, 1160, 1093, 971, 912, 852, 784, 712, 665 cm^{-1}.

1-Tosyl-1,1*a*,6,6*a*-tetrahydroindeno[1,2-*b*]azirine (22u)

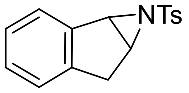

Compound **22u** was prepared according to the general procedure **GP1** and was obtained after column chromatography (*n*-hexane : ethyl acetate 9:1→4:1) as a white solid in 74% yield (42 mg).
Compound **22u** was prepared according to the general procedure **GP2** and was obtained after column chromatography (*n*-hexane : ethyl acetate 9:1→4:1) as a white solid in 83% yield (48 mg).
^{1}H NMR (600 MHz, $CDCl_3$): δ = 8.47 (s, 2H), 8.15 (s, 1H), 7.44 – 7.30 (m, 3H), 7.33 – 7.14 (m, 2H), 4.09 – 3.83 (m, 1H), 3.16 (d, *J* = 7.3 Hz, 1H), 2.59 (d, *J* = 4.6 Hz, 1H) ppm.
^{13}C NMR (151 MHz, $CDCl_3$): δ = 144.4, 143.5, 138.2, 135.4, 129.6, 128.7, 127.7, 126.6, 125.5, 125.0, 50.1, 44.9, 34.6, 21.6 ppm.
HRMS (ESI): mass found: 286.08944, calculated mass for $C_{16}H_{16}NO_2S^+$: 286.08963.
IR (KBr): 3460, 3044, 2924, 2858, 2324, 2164, 2043, 1922, 1811, 1730, 1596, 1465, 1381, 1359, 1311, 1237, 1185, 1152, 1088, 1023, 1001, 968, 927, 871, 814, 766, 719, 671 cm^{-1}.

2-Cyclohexyl-1-tosylaziridine (23a)

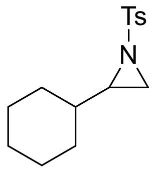

Compound **23a** was prepared according to the general procedure **GP1** and was obtained after column chromatography (*n*-hexane : ethyl acetate 20:1→9:1) as a colorless oil in 40% yield (23 mg).
Compound **23a** was prepared according to the general procedure **GP2** and was obtained after column chromatography (*n*-hexane : ethyl acetate 20:1→9:1) as a colorless oil in 62% yield (35 mg).
^{1}H NMR (600 MHz, $CDCl_3$): δ = 7.88 – 7.75 (m, 2H), 7.33 (d, *J* = 8.1 Hz, 2H), 2.59 (d, *J* = 7.0 Hz, 1H), 2.57 – 2.50 (m, 1H), 2.44 (s, 3H), 2.10 (d, *J* = 4.7 Hz, 1H), 1.76 – 1.67 (m, 1H), 1.66 – 1.58 (m, 3H), 1.53 – 1.46 (m, 1H), 1.22 – 1.03 (m, 4H), 1.03 – 0.97 (m, 1H), 0.95 – 0.86 (m, 1H) ppm.
^{13}C NMR (151 MHz, $CDCl_3$): δ = 144.3, 135.1, 129.5, 128.0, 45.1, 39.3, 32.6, 30.1, 29.6, 25.9, 25.5, 25.3, 21.63 ppm.
HRMS (ESI): mass found: 302.11820, calculated mass for $C_{15}H_{21}NNaO_2S^+$: 302.11852.
IR (KBr): 3336, 2925, 2853, 2662, 2164, 1916, 1725, 1598, 1494, 1449, 1404, 1321, 1230, 1157, 1091, 948, 885, 814, 717, 666 cm^{-1}.

2-Butyl-1-tosylaziridine (23b)

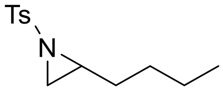

Compound **23b** was prepared according to the general procedure **GP1** and was obtained after column chromatography (*n*-hexane : ethyl acetate 20:1→9:1) as a colorless oil in 47% yield (24 mg).

Compound **23b** was prepared according to the general procedure **GP2** and was obtained after column chromatography (*n*-hexane : ethyl acetate 20:1→9:1) as a colorless oil in 61% yield (31 mg).

^{1}H NMR (400 MHz, $CDCl_3$): δ = 7.81 (d, *J* = 8.2 Hz, 2H), 7.32 (d, *J* = 8.0 Hz, 2H), 2.70 (dq, *J* = 7.3, 4.9 Hz, 1H), 2.61 (d, *J* = 7.0 Hz, 1H), 2.43 (s, 3H), 2.03 (d, *J* = 4.6 Hz, 1H), 1.60 – 1.44 (m, 1H), 1.39 – 1.27 (m, 1H), 1.27 – 1.14 (m, 4H), 0.79 (t, *J* = 7.0 Hz, 3H) ppm.

^{13}C NMR (101 MHz, $CDCl_3$): δ = 144.3, 135.2, 129.5, 127.9, 40.3, 33.7, 30.9, 28.8, 22.0, 21.5, 13.7 ppm.

HRMS (ESI): mass found: 276.10249, calculated mass for $C_{13}H_{19}NNaO_2S^+$: 276.10287.

IR (KBr): 2930, 2865, 2327, 2168, 2103, 1993, 1915, 1597, 1457, 1400, 1321, 1230, 1158, 1090, 1036, 923, 863, 814, 773, 711, 661 cm^{-1}.

2-Hexyl-1-tosylaziridine (23c)

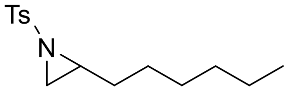

Compound **23c** was prepared according to the general procedure **GP1** and was obtained after column chromatography (*n*-hexane : ethyl acetate 20:1→9:1) as a colorless oil in 41% yield (23 mg).

Compound **23c** was prepared according to the general procedure **GP2** and was obtained after column chromatography (*n*-hexane : ethyl acetate 20:1→9:1) as a colorless oil in 60% yield (34 mg).

^{1}H NMR (400 MHz, $CDCl_3$): δ = 7.81 (d, *J* = 8.3 Hz, 2H), 7.31 (d, *J* = 8.0 Hz, 2H), 2.69 (tt, *J* = 7.3, 4.8 Hz, 1H), 2.62 (d, *J* = 7.0 Hz, 1H), 2.42 (s, 3H), 2.03 (d, *J* = 4.6 Hz, 1H), 1.56 – 1.44 (m, 1H), 1.38 – 1.25 (m, 1H), 1.25 – 1.10 (m, 8H), 0.83 (t, *J* = 7.0 Hz, 3H) ppm.

^{13}C NMR (101 MHz, $CDCl_3$): δ = 144.3, 135.2, 129.5, 127.9, 40.4, 33.7, 31.5, 31.2, 28.6, 26.6, 22.3, 21.5, 13.9 ppm.

HRMS (ESI): mass found: 304.13369, calculated mass for $C_{15}H_{23}NNaO_2S^+$: 304.13417.

IR (KBr): 2927, 2858, 2327, 2085, 2000, 1912, 1597, 1457, 1400, 1323, 1231, 1158, 1091, 1017, 930, 865, 814, 777, 712, 662 cm^{-1}.

2-Dodecyl-1-tosylaziridine (23d)

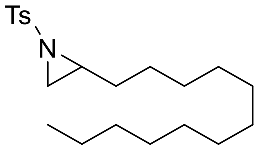

Compound **23d** was prepared according to the general procedure **GP1** and was obtained after column chromatography (*n*-hexane : ethyl acetate 20:1→9:1) as a colorless oil in 43% yield (32 mg).

Compound **23d** was prepared according to the general procedure **GP2** and was obtained after column chromatography (*n*-hexane : ethyl acetate 20:1→9:1) as a colorless oil in 59% yield (43 mg).

^{1}H NMR (400 MHz, $CDCl_3$): δ = 7.80 (d, *J* = 8.2 Hz, 2H), 7.31 (d, *J* = 8.0 Hz, 2H), 2.69 (dq, *J* = 7.3, 4.8 Hz, 1H), 2.61 (d, *J* = 7.0 Hz, 1H), 2.42 (s, 3H), 2.03 (d, *J* = 4.6 Hz, 1H), 1.50 (dt, *J* = 13.3, 5.6 Hz, 1H), 1.38 – 1.08 (m, 21H), 0.86 (t, *J* = 6.8 Hz, 3H) ppm.
^{13}C NMR (101 MHz, $CDCl_3$): δ = 144.3, 135.2, 129.5, 127.9, 40.4, 33.7, 31.8, 31.2, 29.62, 29.60, 29.5, 29.4, 29.38, 29.31, 28.9, 26.7, 22.6, 21.5, 14.0 ppm.
HRMS (ESI): mass found: 366.24557, calculated mass for $C_{21}H_{36}NO_2S^+$: 366.24613.
IR (KBr): 3882, 3525, 2923, 2853, 2327, 2191, 2107, 1988, 1912, 1730, 1597, 1495, 1459, 1401, 1325, 1231, 1160, 1092, 1018, 927, 865, 813, 713, 662 cm^{-1}.

2-(3-Methylbenzyl)-1-tosylaziridine (23e)

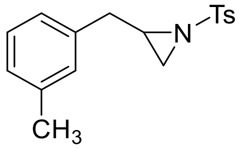

Compound **23e** was prepared according to the general procedure **GP1** and was obtained after column chromatography (*n*-hexane : ethyl acetate 20:1→9:1) as a colorless oil in 53% yield (32 mg).
Compound **23e** was prepared according to the general procedure **GP2** and was obtained after column chromatography (*n*-hexane : ethyl acetate 20:1→9:1) as a colorless oil in 71% yield (43 mg).
^{1}H NMR (400 MHz, $CDCl_3$): δ = 7.66 (d, *J* = 8.3 Hz, 2H), 7.19 (d, *J* = 8.0 Hz, 2H), 7.04 (t, *J* = 7.8 Hz, 1H), 6.95 (d, *J* = 7.6 Hz, 1H), 6.84 (d, *J* = 6.9 Hz, 2H), 3.07 – 2.83 (m, 1H), 2.80 – 2.67 (m, 2H), 2.63 (dd, *J* = 14.4, 7.1 Hz, 1H), 2.40 (s, 3H), 2.22 (s, 3H), 2.15 (d, *J* = 4.5 Hz, 1H) ppm.
^{13}C NMR (101 MHz, $CDCl_3$): δ = 144.1, 137.9, 136.9, 134.8, 129.49, 129.46, 128.3, 127.7, 127.2, 125.6, 41.3, 37.4, 32.8, 21.5, 21.2 ppm.
HRMS (ESI): mass found: 324.10260, calculated mass for $C_{17}H_{19}NNaO_2S^+$: 324.10287.
IR (KBr): 3505, 3022, 2921, 2161, 2029, 1917, 1724, 1676, 1599, 1489, 1451, 1391, 1321, 1230, 1157, 1090, 973, 945, 903, 853, 813, 774, 742, 695, 661 cm^{-1}.

2-Phenethyl-1-tosylaziridine (23f)

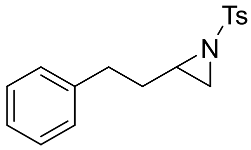

Compound **23f** was prepared according to the general procedure **GP1** and was obtained after column chromatography (*n*-hexane : ethyl acetate 20:1→9:1) as a colorless oil in 43% yield (26 mg).
Compound **23f** was prepared according to the general procedure **GP2** and was obtained after column chromatography (*n*-hexane : ethyl acetate 20:1→9:1) as a colorless oil in 70% yield (42 mg).
^{1}H NMR (600 MHz, $CDCl_3$): δ = 7.88 – 7.73 (m, 2H), 7.34 (d, *J* = 8.1 Hz, 2H), 7.26 (dq, *J* = 5.0, 1.7 Hz, 2H), 7.23 – 7.16 (m, 1H), 7.11 (dd, *J* = 9.1, 2.0 Hz, 2H), 2.81 – 2.73 (m, 1H), 2.67

– 2.53 (m, 3H), 2.44 (s, 3H), 2.05 (d, *J* = 4.6 Hz, 1H), 1.92 – 1.83 (m, 1H), 1.73 – 1.63 (m, 1H)ppm.
^{13}C NMR (151 MHz, $CDCl_3$): δ = 144.5, 140.6, 135.0, 129.6, 128.4, 128.3, 128.0, 126.1, 39.7, 33.8, 33.1, 32.9, 21.6 ppm.
HRMS (ESI): mass found: 324.10257, calculated mass for $C_{17}H_{19}NNaO_2S^+$: 324.10287.
IR (KBr): 3545, 3061, 3028, 2926, 2860, 2167, 2024, 1887, 1811, 1725, 1598, 1495, 1452, 1400, 1320, 1231, 1157, 1091, 1021, 926, 872, 814, 748, 697, 662 cm^{-1}.

6-Tosyl-6-azabicyclo[3.1.0]hexane (23g)

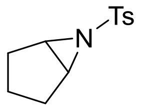

Compound **23g** was prepared according to the general procedure **GP1** and was obtained after column chromatography (*n*-hexane : ethyl acetate 20:1→9:1) as a colorless oil in 68% yield (32 mg).
Compound **23g** was prepared according to the general procedure **GP2** and was obtained after column chromatography (*n*-hexane : ethyl acetate 20:1→9:1) as a colorless oil in 85% yield (41 mg).
^{1}H NMR (400 MHz, $CDCl_3$): δ = 7.78 (d, *J* = 8.2 Hz, 2H), 7.29 (d, *J* = 8.0 Hz, 2H), 3.30 (s, 2H), 2.41 (s, 3H), 1.91 (dd, *J* = 13.3, 7.9 Hz, 2H), 1.75 – 1.46 (m, 3H), 1.45 – 1.23 (m, 1H) ppm.
^{13}C NMR (101 MHz, $CDCl_3$): δ = 144.0, 136.0, 129.5, 127.5, 46.6, 26.9, 21.5, 19.4 ppm.
HRMS (ESI): mass found: 260.07144, calculated mass for $C_{12}H_{15}NNaO_2S^+$: 260.07157.
IR (KBr): 35149, 3048, 2959, 2859, 2162, 2028, 1920, 1598, 1494, 1444, 1402, 1366, 1316, 1191, 1154, 1092, 1012, 973, 870, 814, 720, 670 cm^{-1}.

Methyl (1R,3R,6S)-7-tosyl-7-azabicyclo[4.1.0]heptane-3-carboxylate (23h)

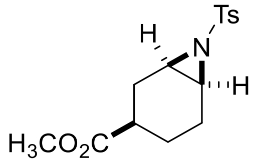

Compound **23h** was prepared according to the general procedure **GP1** and was obtained after column chromatography (*n*-hexane : ethyl acetate 20:1→9:1) as a colorless oil in 58% yield (36 mg).
Compound **23h** was prepared according to the general procedure **GP2** and was obtained after column chromatography (*n*-hexane : ethyl acetate 20:1→9:1) as a colorless oil in 73% yield (45 mg).
^{1}H NMR (400 MHz, $CDCl_3$): δ = 7.77 (d, *J* = 8.3 Hz, 2H), 7.30 (d, *J* = 8.0 Hz, 2H), 3.61 (s, 3H), 3.04 (ddd, *J* = 6.9, 3.9, 1.3 Hz, 1H), 2.96 (t, *J* = 6.2 Hz, 1H), 2.49 – 2.43 (m, 1H), 2.41 (s, 3H), 2.07 (dd, *J* = 14.7, 4.9 Hz, 1H), 2.01 – 1.81 (m, 2H), 1.79 – 1.67 (m, 2H), 1.43 – 1.27 (m, 1H) ppm.
^{13}C NMR (101 MHz, $CDCl_3$): δ = 175.2, 144.2, 135.4, 129.6, 127.6, 51.7, 39.8, 38.7, 35.8, 25.4, 22.9, 21.5, 21.3 ppm.
HRMS (ESI): mass found: 332.09306, calculated mass for $C_{15}H_{19}NNaO_4S^+$: 332.09270.

IR (KBr): 3541, 2950, 2165, 1976, 1919, 1730, 1597, 1495, 1439, 1403, 1316, 1237, 1155, 1090, 1058, 1016, 977, 919, 888, 814, 724, 668 cm^{-1}.

(2Z,4Z,6Z)-9-Tosyl-9-azabicyclo[6.1.0]nona-2,4,6-triene (23i)

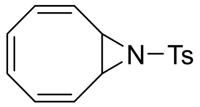

Compound **23i** was prepared according to the general procedure **GP1** and was obtained after column chromatography (*n*-hexane : ethyl acetate 20:1→9:1) as a colorless oil in 20% yield (11 mg).

Compound **23i** was prepared according to the general procedure **GP2** and was obtained after column chromatography (*n*-hexane : ethyl acetate 20:1→9:1) as a colorless oil in 25% yield (14 mg).

^{1}H NMR (600 MHz, $CDCl_3$): δ = 8.14 – 7.71 (m, 2H), 7.35 (d, *J* = 8.1 Hz, 2H), 5.99 (dd, *J* = 12.4, 1.0 Hz, 2H), 5.93 – 5.86 (m, 2H), 5.82 (d, *J* = 11.6 Hz, 2H), 3.41 (s, 2H), 2.46 (s, 3H) ppm.

^{13}C NMR (151 MHz, $CDCl_3$): δ = 144.5, 135.1, 129.5, 129.5, 127.8, 125.4, 124.2, 43.9, 21.6 ppm.

HRMS (ESI): mass found: 274.08943, calculated mass for $C_{15}H_{16}NO_2S^+$: 274.08963.

IR (KBr): 3474, 2923, 2853, 2585, 2241, 2168, 1978, 1930, 1688, 1595, 1493, 1425, 1383, 1318, 1251, 1153, 1088, 1001, 900, 863, 811, 788, 724, 693, 668 cm^{-1}.

DFT Calculations

Computational Details

All calculations were performed using the Gaussian 16, Revision C.01 package.[4] All structures were optimized at the (U)M06-2X level[5] of theory in combination with D3 dispersion corrections,[6] in which all atoms were described with the def2-SVP basis set,[7] Analytical frequency calculations were carried out at the same level of theory in order to confirm each stationary point as either an intermediate (no imaginary frequencies) or a transition state (only one imaginary frequency). Key transition-state structures were confirmed to connect corresponding reactants and products by intrinsic reaction coordinate (IRC) calculations.[8] The electronic energy was then refined using def2-TZVP basis set[7] at the (U)M06-2X level on the optimized geometries in combination with D3 dispersion corrections.[5] Solvation energies in dichloromethane (ε = 8.93) and acetonitrile (ε = 35.688) were evaluated by IEFPCM calculations with radii and non-electrostatic terms for SMD solvation model[9] based on the optimized structures. The given Gibbs free energies in dichloromethane and acetonitrile were calculated in Table S1 according to the formula: $G_{sol} = TCG + E_{sol} + 1.89$ kcal mol^{-1}. Time-dependent (TD)-DFT calculations were carried out on the optimized structures of the PhINTs to obtain the absorption wavelength. The CYLview software was employed to show the 3D structures of the studied species.[10]

Calculation of Single Electron Transfer Steps by Marcus-Hush Theory

To get more details of the singlet electron transfer (SET) process, we estimated the free energy barrier of SET process using the Marcus-Hush theory,[11] which can be calculated according to the following formula:

$$\Delta G^{\ddagger}_{MH} = \frac{(\Delta G_r + \lambda)^2}{4\lambda}$$

Where ΔG_r is the Gibbs free energy change of the SET step, λ is the reorganization energy including inner sphere energy and outer sphere energy. However, the outer sphere energy is often much larger than the inner sphere contribution. Hence, the outer sphere reorganization energy (λ_{outer}) can be regarded as the total reorganization energy, which can be calculated according to the following formula:

$$\lambda = \lambda_{outer} = 332\left(\frac{1}{2r_1} + \frac{1}{2r_2} - \frac{1}{R}\right)\left(\frac{1}{\varepsilon_{opt}} - \frac{1}{\varepsilon_s}\right)$$

Where r_1 and r_2 are the radii of electron donor and acceptor, R is the sum of r_1 and r_2, ε_{opt} and ε_s is the high frequency (optical) dielectric constant and static dielectric constant of solvent respectively (for DCM, $\varepsilon_{opt} = 2.03$, $\varepsilon_s = 8.93$).

Supplementary Table 4. Calculated free energy barriers ($\Delta G^{\ddagger}_{MH}$, kcal mol^{-1}) of single electron transfer steps.

	r_1 (Å)	r_2 (Å)	R (Å)	λ	ΔG_r	$\Delta G^{\ddagger}_{MH}$
$SET1_{DCM}$	5.40	7.15	12.55	10.47	-2.88	1.38
$SET2_{DCM}$	6.08	7.14	13.22	9.68	-38.11	20.51
$SET3_{DCM}$	5.40	7.14	12.54	10.47	-30.15	9.25
$SET1_{MeCN}$	5.40	7.15	12.55	14.45	-17.68	0.18
$SET2_{MeCN}$	6.08	7.14	13.22	13.37	-23.00	1.73
$SET3_{MeCN}$	5.40	7.14	12.54	14.46	-14.45	0.00

Natural Bond Orbital (NBO) Analysis and Orbital Analysis

Further understanding of the structural and electronic properties of triplet nitrene and nitrene radical anion, we then performed natural bond orbital (NBO) analysis and orbital analysis at SMD(DCM)-M06-2X-D3/def2-TZVP level. For both intermediates, the highest spin population is found on the nitrogen atom. And further calculations show nitrene radical anion **INT2** possesses a higher electron density compared to triplet nitrene **INT1$_T$**, which in turn leads to higher nucleophilicity of **INT2**.

Further SOMO orbital analysis and Mayer bond order analysis using Multiwfn program[12] show a π-bonding between S and N atoms in nitrene radical anion **INT2**.

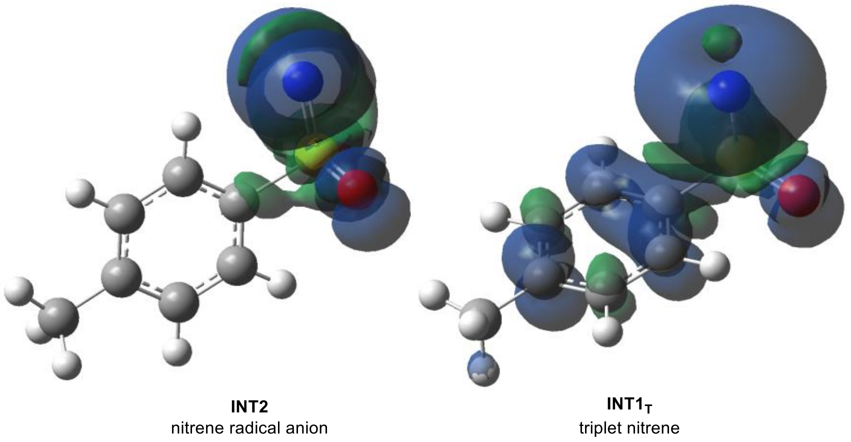

Supplementary Figure 4. Computed spin density plots of nitrene radical anion **INT2** and triplet nitrene **INT1$_T$**.

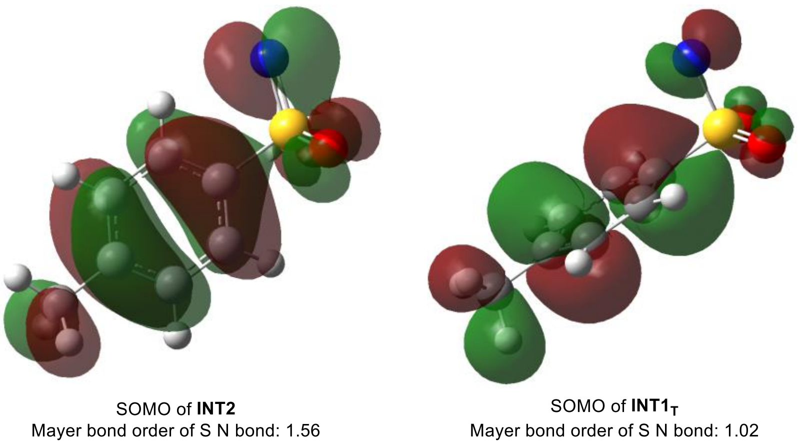

Supplementary Figure 5. SOMO orbitals analysis and Mayer bond order analysis of **INT2** and **INT1$_T$**..

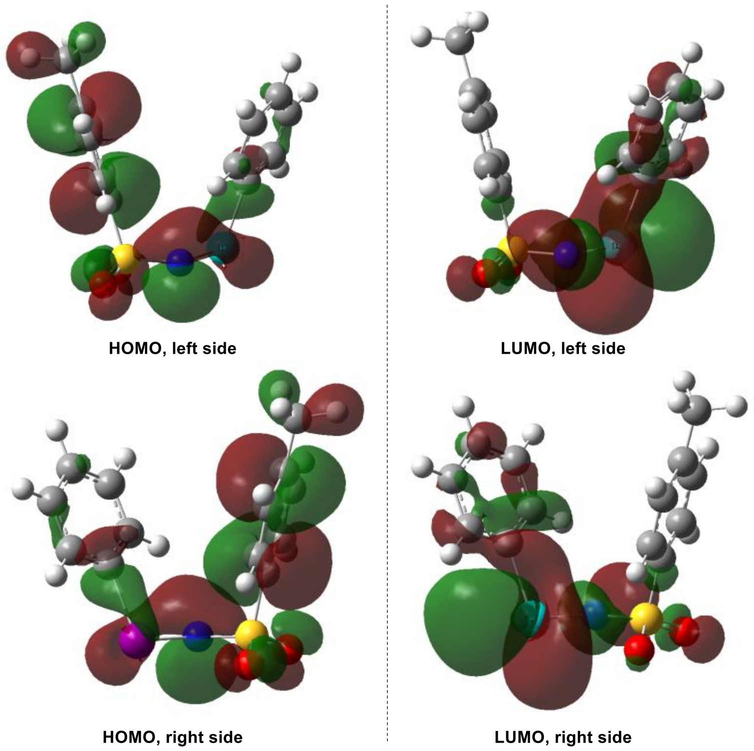

Supplementary Figure 6. HOMO (orbital #77) and LUMO (orbital #78) of **1a**.

Summary of Natural Population Analysis:

Atom	No	Natural Charge	Natural Population Core	Valence	Rydberg	Total
S	1	2.20567	9.99867	3.55646	0.23921	13.79433
O	2	-1.01224	1.99984	6.98427	0.02814	9.01224
O	3	-1.00806	1.99984	6.98005	0.02817	9.00806
C	4	-0.32920	1.99878	4.29631	0.03411	6.32920
C	5	-0.02838	1.99917	4.00962	0.01958	6.02838
C	6	-0.20952	1.99911	4.19017	0.02024	6.20952
C	7	-0.21995	1.99910	4.20046	0.02039	6.21995
C	8	-0.22724	1.99913	4.20919	0.01892	6.22724
C	9	-0.22332	1.99913	4.20579	0.01839	6.22332
H	10	0.23447	0.00000	0.76373	0.00180	0.76553
H	11	0.24153	0.00000	0.75668	0.00179	0.75847
H	12	0.22278	0.00000	0.77558	0.00163	0.77722
H	13	0.22301	0.00000	0.77537	0.00162	0.77699
C	14	-0.63326	1.99945	4.62200	0.01182	6.63326
H	15	0.21971	0.00000	0.77875	0.00154	0.78029
H	16	0.22467	0.00000	0.77359	0.00174	0.77533
H	17	0.22464	0.00000	0.77364	0.00172	0.77536
N	18	-0.90530	1.99977	5.87453	0.03100	7.90530
* Total *		-1.00000	29.99201	58.52619	0.48180	89.00000

Supplementary Figure 7. Natural bond orbital (NBO) analysis of nitrene radical anion **INT2**.

Summary of Natural Population Analysis:

Atom	No	Natural Charge	Natural Population Core	Valence	Rydberg	Total
S	1	2.16138	9.99854	3.61520	0.22489	13.83862
O	2	-0.92720	1.99983	6.89300	0.03437	8.92720
O	3	-0.92716	1.99983	6.89296	0.03437	8.92716
C	4	-0.39735	1.99882	4.36897	0.02956	6.39735
C	5	0.03294	1.99919	3.94890	0.01897	5.96706
C	6	-0.16519	1.99911	4.14655	0.01954	6.16519
C	7	-0.16744	1.99911	4.14878	0.01955	6.16744
C	8	-0.21359	1.99914	4.19620	0.01825	6.21359
C	9	-0.21659	1.99914	4.19890	0.01855	6.21659
H	10	0.25558	0.00000	0.74285	0.00156	0.74442
H	11	0.25545	0.00000	0.74298	0.00156	0.74455
H	12	0.23857	0.00000	0.75988	0.00156	0.76143
H	13	0.23879	0.00000	0.75965	0.00156	0.76121
C	14	-0.64084	1.99944	4.62919	0.01221	6.64084
H	15	0.23831	0.00000	0.75997	0.00172	0.76169
H	16	0.22780	0.00000	0.77069	0.00151	0.77220
H	17	0.23301	0.00000	0.76539	0.00160	0.76699
N	18	-0.22646	1.99990	5.20571	0.02084	7.22646
* Total *		0.00000	29.99203	57.54578	0.46219	88.00000

Supplementary Figure 8. Natural bond orbital (NBO) analysis of triplet nitrene **INT1$_T$**.

Based on the above optimized structure of **1a**, DFT-TD was performed at SMD(DCM)-M06-2X-D3/def2-TZVP level.

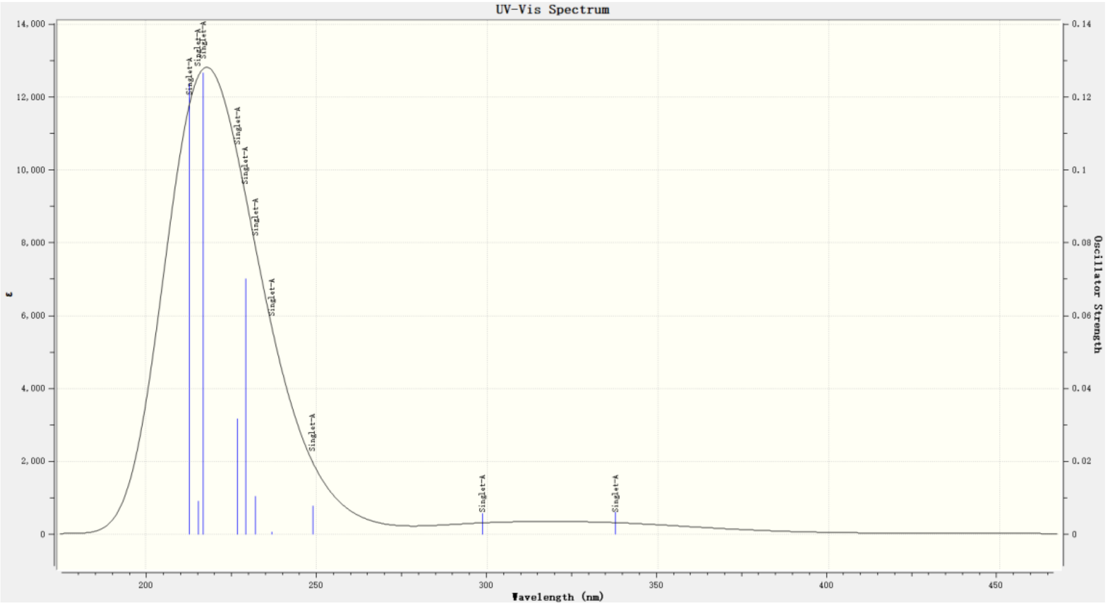

Supplementary Figure 9. Calculated UV-Vis spectrum of **1a**.

Excitation energies and oscillator strengths:

Excited State 1: Singlet-A 3.6721 eV **337.64 nm** f=0.0060 <S**2>=0.000

73 -> 78 0.39940

73 -> 82 -0.10352

76 -> 78 0.24148

77 -> 78 0.44170

Excited State 2: Singlet-A 4.1509 eV **298.69 nm** f=0.0057 <S**2>=0.000

70 -> 78 0.12250

72 -> 78 0.25132

75 -> 78 -0.15361

76 -> 78 0.54218

77 -> 78 -0.24286

Excited State 3: Singlet-A 4.9804 eV **248.94 nm** f=0.0078 <S**2>=0.000

72 -> 78 0.25363

76 -> 79 0.49253

76 -> 82 -0.27028

77 -> 79 -0.17120

Excited State 4: Singlet-A 5.2315 eV **237.00 nm** f=0.0005 <S**2>=0.000

74 -> 80 0.12266

75 -> 78 -0.13612

75 -> 79 0.12337

75 -> 80 0.31266

77 -> 79 0.27284

77 -> 80 -0.25422

77 -> 81 -0.11535
77 -> 82 0.20288
77 -> 83 -0.25140

Excited State 5: Singlet-A 5.3391 eV **232.22 nm** f=0.0104 <S**2>=0.000
72 -> 78 0.25191
74 -> 78 0.17213
74 -> 80 -0.10496
74 -> 81 0.19798
74 -> 82 0.10531
75 -> 78 -0.15263
75 -> 79 -0.12245
75 -> 81 -0.11857
75 -> 82 -0.11446
76 -> 78 -0.11736
76 -> 79 -0.20155
76 -> 81 0.23660
76 -> 83 -0.12094
77 -> 83 0.20932

Excited State 6: Singlet-A 5.4069 eV **229.31 nm** f=0.0701 <S**2>=0.000
72 -> 78 -0.10015
73 -> 79 0.16873
73 -> 82 -0.10762
76 -> 79 0.18136
77 -> 78 -0.26881
77 -> 79 0.39305
77 -> 80 0.35375

Excited State 7: Singlet-A 5.4627 eV **226.97 nm** f=0.0316 <S**2>=0.000
71 -> 78 0.14210
72 -> 78 0.38054
74 -> 78 -0.21657
75 -> 78 0.12948
76 -> 78 -0.18103
76 -> 79 -0.15050
76 -> 81 -0.18524
77 -> 79 0.16933
77 -> 81 0.15221
77 -> 82 -0.14929

Excited State 8: Singlet-A 5.7155 eV **216.93 nm** f=0.1267 <S**2>=0.000
72 -> 78 0.12428
73 -> 78 -0.24056
74 -> 79 -0.14961
75 -> 78 -0.26646

77 -> 78 0.26077
77 -> 80 0.38296
77 -> 82 0.18039
Excited State 9: Singlet-A 5.7548 eV **215.45 nm** f=0.0090 <S**2>=0.000
73 -> 78 -0.21927
74 -> 78 -0.32690
74 -> 79 0.21980
74 -> 81 0.10304
75 -> 78 0.33535
75 -> 79 -0.17903
76 -> 78 0.10597
77 -> 78 0.20777
77 -> 79 0.15100
77 -> 82 0.12092
Excited State 10: Singlet-A 5.8242 eV **212.88 nm** f=0.1236 <S**2>=0.000
72 -> 78 0.10841
73 -> 78 0.30945
73 -> 79 -0.15286
75 -> 78 0.10117
75 -> 80 0.17007
76 -> 79 -0.17587
76 -> 80 0.18061
77 -> 78 -0.16277
77 -> 79 -0.23083
77 -> 80 0.25379
77 -> 81 -0.14498
77 -> 82 0.17127

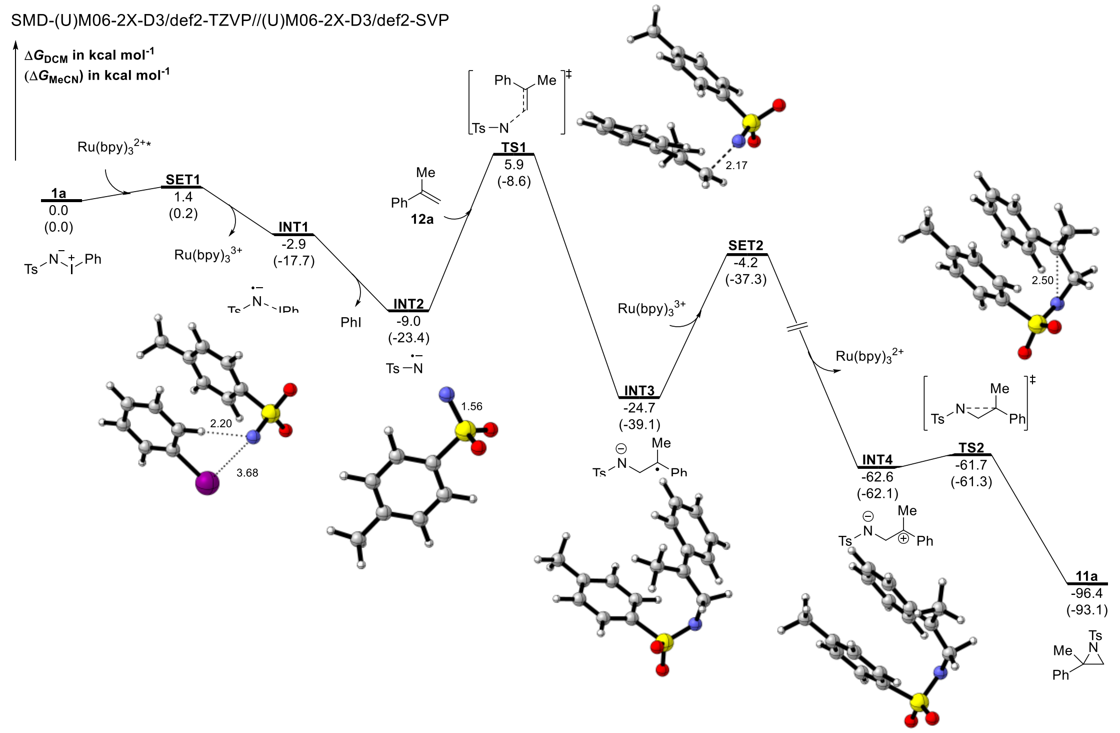

Supplementary Figure 10. Relative free-energy profile of photocatalytic reaction of iminoiodinane **1a** with **12a**.

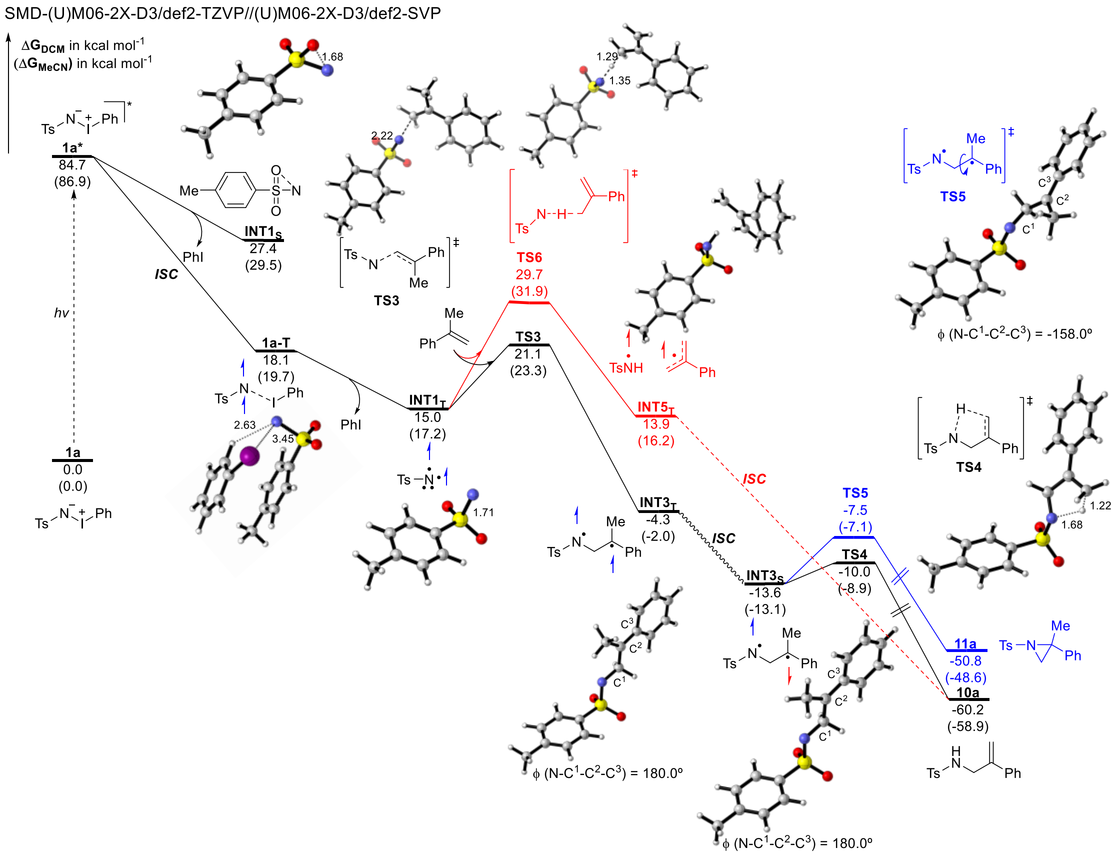

Supplementary Figure 11. Relative free-energy profile of photochemical reaction of iminoiodinane **1a** with **12a**.

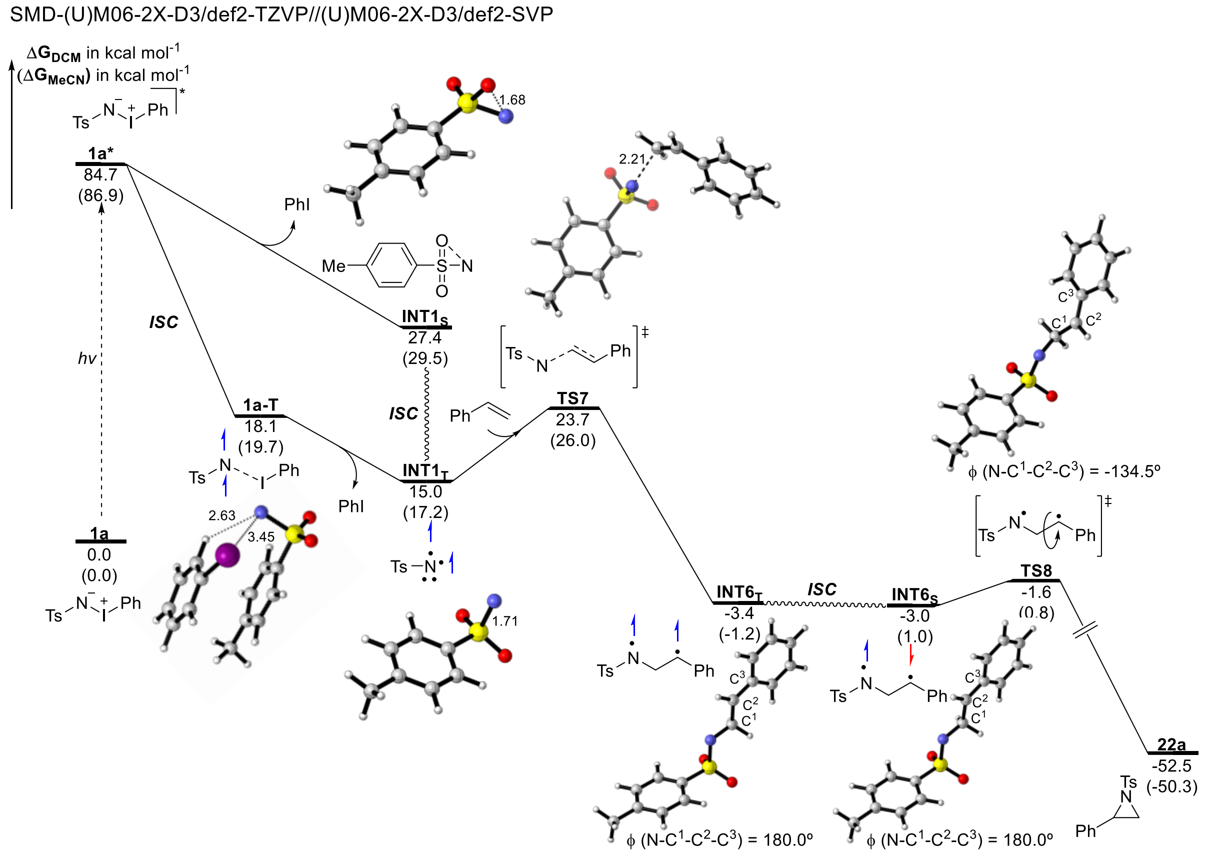

Supplementary Figure 12. Relative free-energy profile of photochemical reaction of iminoiodinane **1a** with styrene.

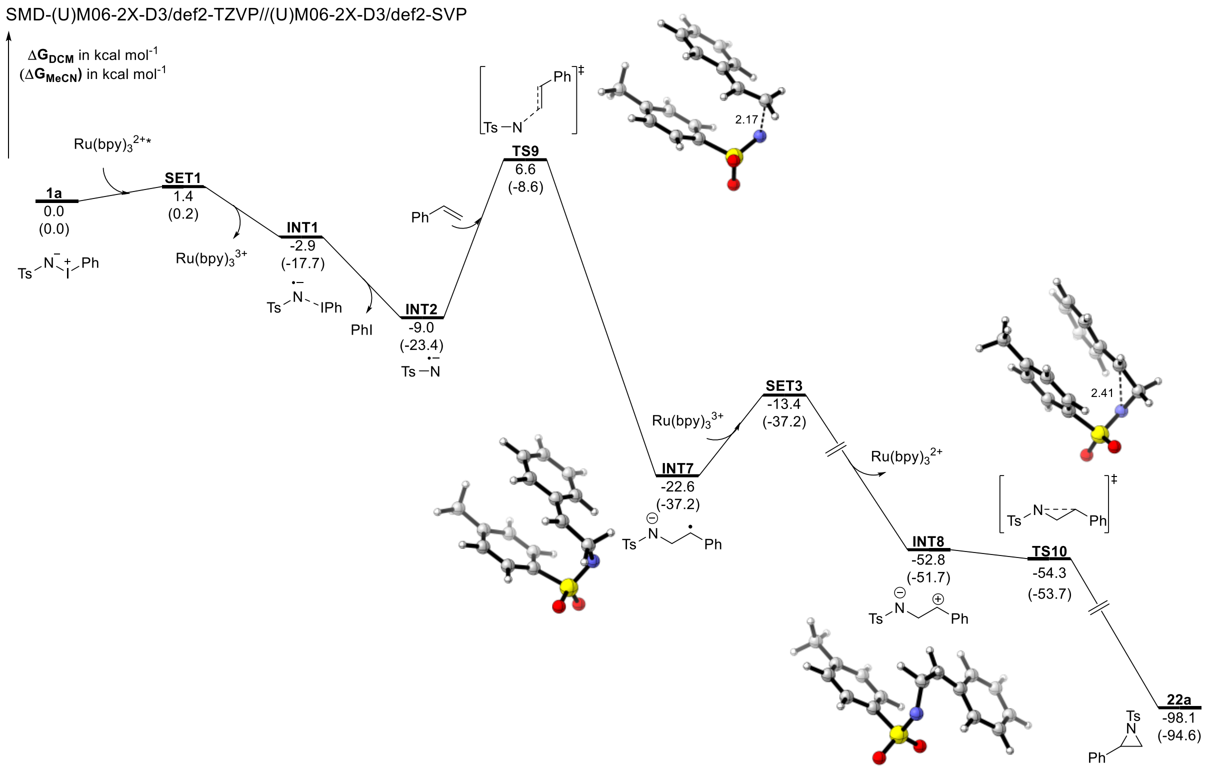

Supplementary Figure 13. Relative free-energy profile of photocatalytic reaction of iminoiodinane **1a** with styrene.

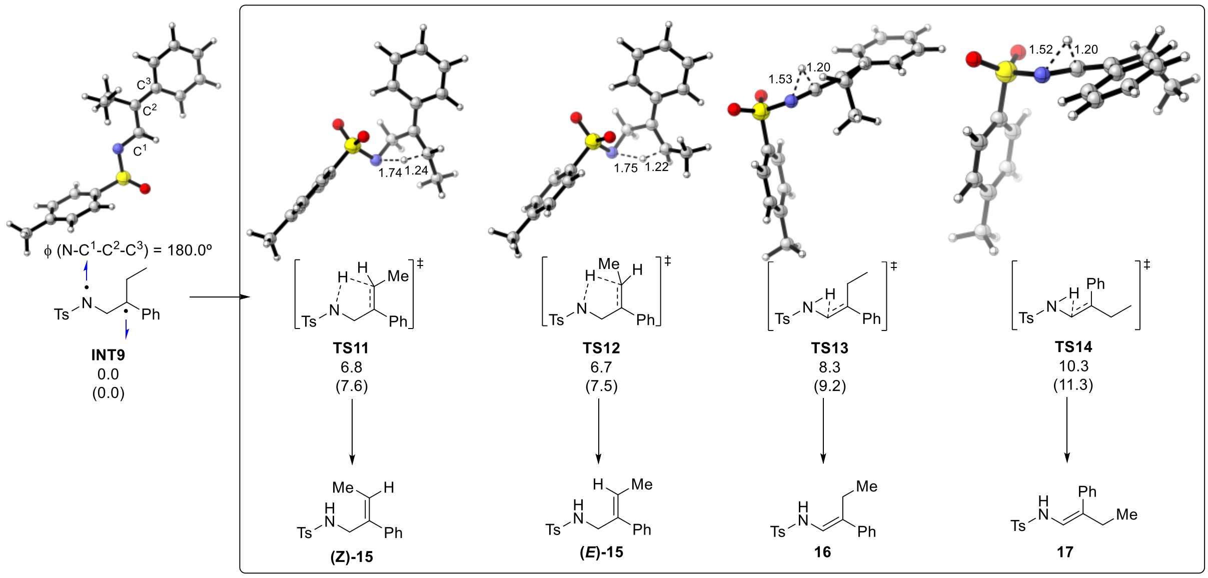

Supplementary Figure 14. Selective H-transfer step of of **INT9** from α-ethyl styrene **13**.

Computed energies of all stationary points

Supplementary Table 5. Thermal correction to Gibbs free energies (***TCG***, in Hartree), thermal correction to enthalpies (***TCH***, in Hartree), sum of electronic and thermal free energies (***G***, in Hartree), Sum of electronic and thermal enthalpies (***G***, in Hartree), single point energies in dichloromethane computed at the (U)M06-2X-D3/def2-TZVP level (E_{sol}, in Hartree), and total spin-squared operator of open-shell species S^2.

Name	*TCG*/a.u.	*TCH*/a.u.	*G*/a.u.	E_{DCM}/a.u.	E_{MeCN}/a.u.	S^2
**1a**	0.177353	0.242490	-1402.290641	-1403.514800	-1403.517404	-
**PhI**	0.059292	0.097728	-528.933922	-529.265727	-529.264630	-
**$Ru(bpy)_3^{2+}$**	0.425155	0.516648	-1578.446643	-1580.731415	-1580.743282	2.0154
**$Ru(bpy)_3^{3+}$**	0.429102	0.518877	-1578.082838	-1580.590952	-1580.622206	0.7551
**$Ru(bpy)_3^{2+}$**	0.428938	0.518655	-1578.530139	-1580.807785	-1580.818006	-
**INT1**	0.171647	0.241317	-1402.391271	-1403.658100	-1403.664898	0.7562
**INT2**	0.091769	0.141810	-873.455084	-874.384549	-874.391828	0.7554
**12a**	0.130070	0.171714	-348.412462	-348.939666	-348.938195	-
**TS1**	0.245426	0.314092	-1221.861269	-1223.321025	-1223.326921	0.7833
**INT3**	0.247321	0.316133	-1221.912959	-1223.371752	-1223.377426	0.7705
**INT4**	0.250611	0.316238	-1221.802279	-1223.218386	-1223.221399	-
**TS2**	0.251330	0.315886	-1221.798069	-1223.217738	-1223.220851	-
**11a**	0.250983	0.319436	-1221.885743	-1223.272562	-1223.271219	-

**1a-T**	0.172441	0.241693	-1402.289544	-1403.481074	-1403.481175	2.0105
**INT1$_T$**	0.094300	0.141280	-873.358573	-874.204410	-874.204644	2.0099
**INT1$_S$**	0.094261	0.142582	-873.335132	-874.184669	-874.185024	-
**TS3**	0.242420	0.314998	-1221.764543	-1223.149365	-1223.148069	2.0422
**INT3$_T$**	0.241798	0.315996	-1221.806492	-1223.189179	-1223.187874	2.0267
**INT3$_S$**	0.241760	0.314988	-1221.808396	-1223.204017	-1223.205534	0.7808
**TS4**	0.243793	0.312832	-1221.799781	-1223.200383	-1223.200870	-
**10a**	0.251686	0.31984	-1221.891522	-1223.288158	-1223.288344	-
**TS5**	0.245307	0.314771	-1221.803881	-1223.197938	-1223.199454	0.8932
**TS6**	0.236607	0.309412	-1221.752338	-1223.129951	-1223.128657	2.0284
**INT5$_T$**	0.238007	0.313213	-1221.778834	-1223.156505	-1223.154975	2.0419
**styrene**	0.102670	0.142140	-309.173011	-309.628699	-309.627427	-
**TS7**	0.216379	0.285421	-1182.521147	-1183.835609	-1183.834411	2.0477
**INT6$_T$**	0.216294	0.286095	-1182.563882	-1183.878730	-1183.877660	2.0305
**INT6$_S$**	0.217186	0.285917	-1182.562806	-1183.878956	-1183.875006	1.0058
**TS8**	0.219382	0.285469	-1182.559469	-1183.879009	-1183.877547	0.9625
**22a**	0.224321	0.290284	-1182.644777	-1183.965078	-1183.964015	-
**TS9**	0.219275	0.284222	-1182.618210	-1184.010229	-1184.016711	0.7832
**INT7**	0.222044	0.286180	-1182.670196	-1184.059547	-1184.065831	0.7740
**INT8**	0.221888	0.287355	-1182.547794	-1183.890448	-1183.892743	-
**TS10**	0.224321	0.290284	-1182.547262	-1183.896158	-1183.899277	-
**INT9**	0.270312	0.345461	-1261.043697	-1262.508135	-1262.509447	0.8609
**TS11**	0.273122	0.343914	-1261.032913	-1262.500094	-1262.500072	-
**TS12**	0.273639	0.343560	-1261.033268	-1262.500817	-1262.500872	-
**TS13**	0.270931	0.344196	-1261.027899	-1262.495560	-1262.495362	-
**TS14**	0.271056	0.344429	-1261.026378	-1262.492449	-1262.492119	-

Coordinates of all stationary points

1a

S	-1.44134200	1.90371500	0.03578200
O	-1.70391400	3.07529800	-0.78015200
O	-2.00137500	1.77076500	1.37748700
C	0.33089900	1.75304200	0.20750100
C	3.08103700	1.29213900	0.33468500
C	0.86641000	1.11013400	1.32053500
C	1.14779400	2.20211600	-0.82737600
C	2.51863900	1.97189100	-0.75448700
C	2.23874700	0.87723600	1.37345400
H	0.20303400	0.80560000	2.13252300
H	0.69779800	2.72864600	-1.67107900
H	3.16825900	2.32619100	-1.55816900
H	2.66645600	0.36762100	2.24002000
C	4.55415000	0.98692900	0.37768300
H	5.12597300	1.67539700	-0.25808900
H	4.94608000	1.05177500	1.40164100
H	4.74215600	-0.03765100	0.01751000

N	-1.87849000	0.64335300	-0.96382700
I	-1.94846700	-1.10431300	0.02932200
C	0.07070200	-1.73664100	-0.20184400
C	2.72013200	-2.40579800	-0.50776800
C	0.77390500	-1.17843100	-1.26055300
C	0.64212700	-2.61028100	0.71504400
C	1.98592200	-2.94912700	0.54593100
C	2.11804600	-1.52113800	-1.40381900
H	0.28361100	-0.46683600	-1.93006500
H	0.07123000	-3.01379600	1.55308000
H	2.45922800	-3.63411100	1.25075400
H	2.69603400	-1.08587400	-2.22069400
H	3.77101700	-2.67218900	-0.62979100

PhI

C	2.63733400	-1.20576800	-0.00000100
C	1.24236400	-1.21381300	0.00000000
C	0.55723200	-0.00000300	0.00000000
C	1.24236200	1.21381300	0.00000000
C	2.63732800	1.20577100	-0.00000100
C	3.33651400	0.00000100	-0.00000200
H	3.17755200	-2.15369600	-0.00000100
H	0.69579100	-2.15692000	0.00000000
H	0.69578100	2.15691500	0.00000000
H	3.17754900	2.15369800	-0.00000100
H	4.42711500	0.00000600	-0.00000200
I	-1.54891700	0.00000000	0.00000100

$Ru(bpy)_3^{2+*}$

C	2.30520200	-1.82747500	-0.47887000
C	0.59563700	-2.32787500	-1.98005300
C	1.33895700	-3.34811700	-2.56353900
C	2.61497700	-3.60542200	-2.06789200
C	3.10353000	-2.83937500	-1.01216600
C	2.72943500	-0.94746300	0.63810800
C	2.10021000	0.80960600	2.04181400
C	3.34036700	0.80791700	2.67231300
C	4.29860800	-0.11203800	2.25701200
C	3.99150800	-0.99867300	1.22645300
H	-0.40956100	-2.08484500	-2.33292600
H	0.92073600	-3.92358700	-3.38878200
H	4.09729000	-3.03332800	-0.61223300
H	1.31468600	1.51545400	2.32004800
H	3.54270100	1.52304900	3.46897900
H	4.73643400	-1.71433100	0.88302500
N	1.06986800	-1.58822200	-0.97117000
N	1.80626400	-0.05301400	1.06022400
C	-2.72992100	-0.94627900	-0.63805100
C	-3.99205800	-0.99689200	-1.22631100
C	-4.29880600	-0.11011400	-2.25685100
C	-3.34015800	0.80938700	-2.67221900
C	-2.09996300	0.81050000	-2.04179400
C	-2.30603600	-1.82648800	0.47890100
C	-3.10473900	-2.83812600	1.01213100
C	-2.61649500	-3.60438300	2.06784700
C	-1.34040500	-3.34754200	2.56355200
C	-0.59669900	-2.32754900	1.98012200
H	-5.28374000	-0.13601300	-2.72494400
H	-1.31412800	1.51598500	-2.32006900
H	-4.09854900	-3.03171900	0.61214500
H	-3.23036800	-4.39679000	2.49817900
H	0.40857300	-2.08488900	2.33304100
C	-0.46508400	2.78598000	0.53496900
C	-1.76594900	1.43631000	1.99890700
C	-2.33679800	2.53068500	2.59467800
C	-1.94987400	3.82511100	2.14311100
C	-1.03593400	3.94699600	1.13072200
C	0.46646900	2.78573000	-0.53505000
C	1.76655700	1.43537200	-1.99903500
C	2.33801900	2.52944300	-2.59477900
C	1.95183800	3.82407400	-2.14316600
C	1.03797300	3.94644100	-1.13076700
H	-2.02630100	0.42368200	2.31591100
H	-2.38639400	4.71553600	2.59611200
H	2.02633300	0.42260800	-2.31608000
H	2.38886300	4.71426500	-2.59613800
H	0.74986100	4.93160400	-0.76689800
N	-1.07062600	-1.58770000	0.97124100
N	0.83681100	1.53649700	-1.01582400
H	-0.92241800	-3.92318300	3.38879300
H	-4.73730600	-1.71218600	-0.88282200
H	-3.54220600	1.52461200	-3.46887500
H	-3.06294700	2.40044500	3.39469300
H	3.06407700	2.39881500	-3.39481300

H	3.22855700	-4.39802600	-2.49828000
H	5.28349600	-0.13840400	2.72517300
N	-0.83613100	1.53694500	1.01571200
N	-1.80636000	-0.05225600	-1.06022000
Ru	-0.00003200	-0.01511300	-0.00001000
H	-0.74725900	4.93200800	0.76689200

Ru(bpy)$_3$$^{3+}$

C	1.71363700	-2.31634400	0.57640600
C	2.20893200	-0.56451000	2.04111400
C	3.19798900	-1.31013300	2.67268100
C	3.44364700	-2.60630200	2.22445200
C	2.69477200	-3.11350200	1.16347800
C	0.86484300	-2.75308400	-0.55606300
C	-0.84327600	-2.13542300	-2.02623600
C	-0.81531000	-3.37575500	-2.65368000
C	0.09158200	-4.33147700	-2.20047000
C	0.93866800	-4.01706400	-1.13862900
H	1.98277900	0.45543000	2.35935800
H	3.76134500	-0.87584100	3.49881100
H	2.88113800	-4.12345100	0.80090500
H	-1.53875500	-1.35727800	-2.34782500
H	-1.49562500	-3.58154100	-3.48029600
H	1.64772500	-4.75780600	-0.77159200
N	1.48330700	-1.05758900	1.02586800
N	-0.02054900	-1.83128300	-1.01068500
C	1.16309000	2.64622200	0.55269300
C	1.37223200	3.89750900	1.12984200
C	0.55983000	4.30820500	2.18580800
C	-0.44842800	3.45987800	2.63877800
C	-0.60884900	2.22667300	2.01737500
C	1.96222600	2.11457600	-0.57543500
C	3.03284300	2.79280300	-1.15578500
C	3.72317000	2.20327200	-2.21382600
C	3.33004500	0.94546900	-2.66626800
C	2.25793200	0.31905000	-2.04096000
H	0.71396700	5.28510400	2.64768000
H	-1.38626000	1.53054800	2.33921600
H	3.33383300	3.77343300	-0.78979200
H	4.56249000	2.72417400	-2.67814200
H	1.91579300	-0.66677600	-2.36295900
C	-2.82440900	0.61762800	-0.57279400
C	-1.42960000	1.77033200	-2.05162100
C	-2.51542300	2.35630600	-2.69242000
C	-3.79845000	2.05141700	-2.24261600
C	-3.95400200	1.17478900	-1.16960800
C	-2.87293100	-0.31773700	0.57437400
C	-1.60540500	-1.59859000	2.06210600
C	-2.74656600	-2.07024800	2.70096500
C	-3.99127600	-1.64167000	2.24442700
C	-4.05464300	-0.75781300	1.16803200
H	-0.40615000	1.98061400	-2.36953700
H	-4.67486200	2.49329500	-2.72019000
H	-0.60960600	-1.90940100	2.38514900
H	-4.90909300	-1.99301200	2.71938200
H	-5.02183400	-0.41971800	0.79862300
N	1.58983800	0.89144000	-1.02782900
N	-1.66805300	-0.74058100	1.03222900
H	3.84271000	0.45008000	-3.49101300
H	2.15938000	4.55454700	0.76260100
H	-1.10625600	3.74348000	3.46053600
H	-2.35044600	3.03842100	-3.52667500
H	-2.65334000	-2.76141000	3.53886500
H	4.21385800	-3.22075300	2.69414200
H	0.14036900	-5.31724100	-2.66662700
N	-1.58189700	0.91892800	-1.02577100
N	0.18031800	1.82896600	1.00742200
Ru	-0.00485300	0.00240300	0.00044000
H	-4.95161300	0.93332300	-0.80534100

Ru(bpy)$_3$$^{2+}$

C	-1.56014600	-2.43557300	-0.58527900
C	-2.11569600	-0.76363000	-2.10026700
C	-3.04213000	-1.58011900	-2.74057100
C	-3.22283100	-2.87739400	-2.26842200
C	-2.47322100	-3.30949700	-1.17814100
C	-0.71364200	-2.80486700	0.57766300
C	0.89248500	-2.07593100	2.09039800
C	0.92444000	-3.31057800	2.73041200
C	0.09460200	-4.32468200	2.25979700
C	-0.73334600	-4.06853900	1.17062600
H	-1.93838700	0.26059800	-2.43510100
H	-3.60670900	-1.19898800	-3.59088700
H	-2.60428800	-4.31995300	-0.79531200

H	1.52363400	-1.24969400	2.42446500
H	1.58933300	-3.46586500	3.57944300
H	-1.38584900	-4.85148700	0.78852700
N	-1.39265900	-1.17833000	-1.05307700
N	0.09480200	-1.82723200	1.04482500
C	-1.33876500	2.56767200	-0.57678000
C	-1.64841000	3.79334400	-1.16886500
C	-0.90289900	4.23277400	-2.25900200
C	0.13661400	3.43603400	-2.73137700
C	0.38950700	2.22678000	-2.09206600
C	-2.07633500	2.01406500	0.58726000
C	-3.16410600	2.65548800	1.18240400
C	-3.79290600	2.06266200	2.27348100
C	-3.31921000	0.84097500	2.74418900
C	-2.23160400	0.25854100	2.10173500
H	-1.13280600	5.18814600	-2.73205300
H	1.19288900	1.56733800	-2.42715600
H	-3.52363700	3.60933700	0.80073000
H	-4.64404300	2.55155500	2.74881800
H	-1.82401200	-0.69807000	2.43540800
C	2.78911000	0.78485200	0.58247100
C	1.35233600	1.79944300	2.10095000
C	2.40388300	2.44446400	2.74374200
C	3.69744400	2.23936500	2.27129200
C	3.89169600	1.40026100	1.17785800
C	2.89443000	-0.12740100	-0.58455800
C	1.72672300	-1.44151500	-2.10406600
C	2.89782500	-1.83102000	-2.74580200
C	4.11066300	-1.33776400	-2.27227100
C	4.10842800	-0.47640600	-1.17887900
H	0.32087700	1.92815900	2.43577200
H	4.54902300	2.72730900	2.74681700
H	0.75181100	-1.80117300	-2.43994800
H	5.05112200	-1.61983000	-2.74700400
H	5.04829000	-0.08427600	-0.79460500
N	-1.62448500	0.82842700	1.05379600
N	1.72274300	-0.61140800	-1.05417600
H	-3.78009000	0.34032800	3.59506500
H	-2.46245100	4.40575600	-0.78538200
H	0.74696200	3.73981200	-3.58122200
H	2.20428400	3.09300400	3.59615600
H	2.85142700	-2.50788400	-3.59829900
H	-3.94022300	-3.54859200	-2.74196800
H	0.09042000	-5.30705000	2.73343700
N	1.53786900	0.99009500	1.05125500
N	-0.32843500	1.80145400	-1.04559600
Ru	0.00244500	0.00086300	-0.00083800
H	4.89634700	1.23166900	0.79440800

INT1

S	0.11986900	-2.82809500	-0.28738600
O	-0.67290500	-3.97339800	-0.77169700
O	0.96993000	-3.01695900	0.90087300
C	-1.15610300	-1.66613700	0.29380300
C	-2.98333500	0.37124000	0.93792700
C	-0.88932600	-0.79569000	1.34878700
C	-2.35212600	-1.54977800	-0.41375800
C	-3.25666500	-0.54107400	-0.08932400
C	-1.79474400	0.21755800	1.66032000
H	0.04058500	-0.92803400	1.90524100
H	-2.55218900	-2.26482800	-1.21466700
H	-4.19240300	-0.44935400	-0.64900800
H	-1.57153200	0.91112500	2.47647600
C	-3.92253800	1.51073900	1.23835800
H	-4.92932300	1.31546100	0.84239500
H	-4.00631100	1.68777900	2.32068800
H	-3.55494600	2.44332100	0.77919200
N	0.90220800	-2.15607600	-1.47310400
I	2.42640400	0.68350800	0.31184100
C	0.70499500	1.62567400	-0.45412400
C	-1.60488300	2.82233500	-1.41625500
C	-0.07677700	0.93466400	-1.37814200
C	0.35364300	2.89629300	0.00023400
C	-0.80963300	3.49327400	-0.48737800
C	-1.23690200	1.54966400	-1.85256200
H	0.20229900	-0.08550100	-1.68079700
H	0.97811500	3.41157800	0.73108500
H	-1.09314200	4.48710300	-0.13429200
H	-1.86599800	1.00914100	-2.56210300
H	-2.51777600	3.28861200	-1.79259700

INT2

S	2.01351600	0.01067600	-0.00041600
O	2.32324900	-0.99647000	-1.03384500

O	2.29529700	-0.36410500	1.39867600
C	0.18260600	0.01918100	-0.03058600
C	-2.62865500	0.01773900	0.00223600
C	-0.51759000	-1.18924100	-0.01052100
C	-0.51501300	1.21989800	-0.04445700
C	-1.91277900	1.21659800	-0.02651300
C	-1.90814700	-1.18677700	0.00774800
H	0.05107600	-2.12132100	-0.02053100
H	0.06904200	2.14282300	-0.08194600
H	-2.45923800	2.16434800	-0.03866400
H	-2.45463800	-2.13498200	0.02288200
C	-4.13704000	0.00375100	0.01894900
H	-4.54326600	1.02481100	0.02660200
H	-4.54275900	-0.51424100	-0.86420900
H	-4.52234500	-0.51988500	0.90774100
N	2.55103100	1.43790600	-0.33785900

12a

C	-1.85368500	-1.22748700	-0.19220900
C	-0.46104400	-1.16259100	-0.18635300
C	0.20588900	0.05838000	-0.00851900
C	-0.57242800	1.20917600	0.19541400
C	-1.96210800	1.14602100	0.19283800
C	-2.61084600	-0.07350200	-0.00573900
H	-2.34831800	-2.18849200	-0.34204800
H	0.11249300	-2.07820000	-0.33507300
H	-0.07847900	2.16357300	0.38319500
H	-2.54416800	2.05396300	0.35883800
H	-3.70062100	-0.12381300	-0.00461300
C	1.69411200	0.12435900	-0.02355300
C	2.34540300	1.24510400	-0.36112000
H	1.81845200	2.14701400	-0.67644400
H	3.43647100	1.28205600	-0.35040400
C	2.44586100	-1.13094600	0.33704200
H	2.31787500	-1.90466300	-0.43569500
H	3.51993200	-0.92830800	0.43123400
H	2.07944500	-1.55421400	1.28420100

TS1

S	-2.25029400	-1.24815200	-0.42335500
N	-1.07438600	-2.20881500	-0.84345100
O	-2.77152500	-1.42180100	0.95434700
O	-3.26052600	-1.23396700	-1.49537300
C	-1.61250600	0.45734100	-0.36507500
C	-0.45005300	0.78407700	-1.05835500
H	0.04545600	-0.00052700	-1.63352100
C	0.08199700	2.06753900	-0.95689000
H	1.01438100	2.30760000	-1.47620500
C	-0.54638400	3.04739600	-0.17776100
C	-1.72434100	2.70602000	0.49736100
H	-2.22128200	3.45784700	1.11716700
C	-2.25405400	1.41869800	0.41170300
H	-3.14529100	1.12853200	0.97111600
C	3.96422400	0.39173100	0.38850000
C	2.81114200	0.01375900	1.07359600
C	1.93493600	-0.95413200	0.54909100
C	2.24266200	-1.50017400	-0.71479700
C	3.39265700	-1.11771200	-1.39722200
C	4.26709800	-0.17487900	-0.84928600
H	4.63010100	1.14011600	0.82437300
H	2.58363700	0.47245100	2.03750500
H	1.51859500	-2.17647700	-1.17562100
H	3.59975900	-1.54477000	-2.38103800
H	5.16611400	0.12645100	-1.39050500
C	0.70993900	-1.34704700	1.26041400
C	0.08190200	-2.52436800	0.96271400
H	-0.81810800	-2.81194700	1.50569500
H	0.59400100	-3.30225900	0.39921400
C	0.11537200	-0.38453200	2.25512700
C	0.04270000	4.43186300	-0.07368800
H	-0.01958100	4.96505100	-1.03546900
H	-0.48499900	5.03393500	0.67896900
H	1.10589900	4.38900900	0.20665500
H	0.17112600	0.65373000	1.88732900
H	0.64223100	-0.42203500	3.22586400
H	-0.94395300	-0.63190500	2.41193100

INT3

S	-2.08564000	-1.35408000	-0.43340700
N	-0.78469600	-2.22033400	-0.55008500
O	-2.82525700	-1.51788600	0.84292200
O	-2.87283200	-1.44383100	-1.66503800
C	-1.57301800	0.39726200	-0.36233500
C	-0.45110000	0.81394400	-1.07836700

H	0.09882900	0.07336700	-1.66343500
C	-0.02290800	2.13514500	-0.99150000
H	0.87497300	2.44829100	-1.53204100
C	-0.71202500	3.06575100	-0.20077200
C	-1.84200200	2.63360400	0.50039600
H	-2.38543800	3.34557500	1.12833000
C	-2.27068700	1.30667000	0.42606400
H	-3.12810400	0.94550300	0.99696500
C	3.85954400	0.57983800	0.41825100
C	2.68963000	0.19584200	1.06162900
C	1.88075500	-0.85693400	0.55865400
C	2.30933500	-1.48249100	-0.64536600
C	3.47596900	-1.08518800	-1.28166800
C	4.26862700	-0.05663000	-0.75687300
H	4.46164000	1.38997300	0.83667000
H	2.38928900	0.70846200	1.97670300
H	1.66133000	-2.23600300	-1.09630700
H	3.76945800	-1.57430200	-2.21338200
H	5.18491900	0.25131200	-1.26380200
C	0.67437900	-1.26628700	1.21103500
C	-0.09321900	-2.46736700	0.70661500
H	-0.78818900	-2.79384300	1.50238600
H	0.61237200	-3.29894000	0.53230800
C	0.08233000	-0.44978500	2.31789900
C	-0.23355300	4.49328500	-0.11374000
H	-0.34158700	5.00997700	-1.08043900
H	-0.80390100	5.05954300	0.63565400
H	0.83101200	4.53805800	0.16170300
H	0.15813100	0.63113500	2.11117900
H	0.58423700	-0.63768200	3.28696100
H	-0.98349100	-0.69903200	2.42303900

INT4

S	2.58230100	0.15779000	0.49720500
N	1.98596400	-1.32553800	0.39581400
O	3.03550200	0.40635900	1.85467500
O	3.45041000	0.49599900	-0.63782100
C	1.09121300	1.14318400	0.23666000
C	0.73164000	1.52433200	-1.05361500
H	1.46575400	1.41388300	-1.85555200
C	-0.54043300	2.05747700	-1.29004800
H	-0.82121600	2.36355200	-2.30161700
C	-1.45737800	2.21139300	-0.24867900
C	-1.05984200	1.84953700	1.05225900
H	-1.76187600	1.98505500	1.87946400
C	0.19953200	1.32052500	1.29751800
H	0.51296600	1.02133500	2.30033100
C	-3.31429100	-1.00643500	0.14512500
C	-2.26727200	-1.07754500	-0.75677600
C	-0.96729100	-1.45113700	-0.32584700
C	-0.76033800	-1.76097400	1.04296900
C	-1.82943000	-1.71700700	1.93027300
C	-3.09613600	-1.33643100	1.48880900
H	-4.30580000	-0.70235500	-0.19160500
H	-2.44365100	-0.81549800	-1.80045500
H	0.24965400	-2.01623200	1.36804000
H	-1.66863800	-1.97194900	2.97804200
H	-3.92713500	-1.29461200	2.19548500
C	0.10832300	-1.49423600	-1.27132900
C	1.54373000	-1.60896100	-0.90273200
H	1.62692000	-2.71637300	-1.12046100
H	2.14583900	-1.16986700	-1.72963000
C	-0.18435300	-1.55096600	-2.73024100
H	-1.02809200	-2.20727500	-2.97758000
H	0.70688300	-1.84011900	-3.30025400
H	-0.45108100	-0.52423900	-3.03738200
C	-2.83393100	2.77183700	-0.48765300
H	-3.03423700	2.90347300	-1.55915800
H	-2.94911000	3.75121200	0.00047100
H	-3.60550600	2.10771000	-0.06910700

TS2

S	-2.49164900	0.32861600	-0.51316300
N	-2.07798900	-1.20400300	-0.64340800
O	-3.22106400	0.61143800	0.73618500
O	-3.02360200	0.79794000	-1.78192200
C	-0.91467200	1.19245800	-0.28281900
C	0.00973700	1.19729100	-1.33076200
H	-0.29577300	0.80760000	-2.30463800
C	1.29434200	1.67373100	-1.10948300
H	2.02367000	1.66824900	-1.92441100
C	1.68088200	2.16545600	0.15178900
C	0.73113000	2.18882300	1.17423500
H	1.00480900	2.59635500	2.15093700

C	-0.56511700	1.70644200	0.96031700
H	-1.32744600	1.73382900	1.74213300
C	1.87576100	-2.00812300	-1.71170000
C	0.71372000	-1.92139800	-0.96215100
C	0.77330100	-1.49743900	0.39395500
C	2.02437800	-1.10737300	0.93589200
C	3.17527800	-1.17223400	0.16662300
C	3.10101600	-1.63556900	-1.15049000
H	1.82895100	-2.34938800	-2.74593400
H	-0.26705100	-2.13188000	-1.39515300
H	2.08893600	-0.73639900	1.95801300
H	4.13293600	-0.86730100	0.58890200
H	4.01057700	-1.69547200	-1.75145100
C	-0.41022300	-1.51674300	1.19389900
C	-1.77676500	-1.78581500	0.61975600
H	-2.51481000	-1.55535400	1.41256600
H	-1.78004000	-2.89989000	0.53702100
C	3.08290300	2.66991100	0.36668400
H	3.27477600	3.56765100	-0.23989600
H	3.26019200	2.92938700	1.41867800
H	3.82391000	1.91285500	0.06652800
C	-0.38676600	-1.34840000	2.66063700
H	0.59800200	-1.33190300	3.13336700
H	-0.93716500	-0.41473900	2.87247300
H	-1.00825800	-2.14458100	3.10294400

11a

S	-0.67470300	-1.26996300	0.57987400
N	0.48114600	-0.45107800	-0.33268900
O	-0.28824000	-1.06647900	1.96472500
O	-0.89485500	-2.61280200	0.05415500
C	-2.11915900	-0.30824300	0.22814500
C	-3.02990400	-0.76595900	-0.71656000
H	-2.85519400	-1.71980800	-1.21575900
C	-4.15840200	0.00680100	-0.98768600
H	-4.88310000	-0.34081000	-1.72643800
C	-4.38022300	1.21710200	-0.32361300
C	-3.44490400	1.64377100	0.63239600
H	-3.61295500	2.58301800	1.16376800
C	-2.31502200	0.88839200	0.91683400
H	-1.59053600	1.20405400	1.66889000
C	4.99954500	1.14795000	-0.86130800
C	4.04923700	0.13651200	-0.98930200
C	2.86832700	0.18195900	-0.24366800
C	2.64819100	1.24352000	0.63588600
C	3.60080400	2.25257000	0.76713200
C	4.77660000	2.20740600	0.01836700
H	5.91633900	1.11046500	-1.45169400
H	4.22117400	-0.69375400	-1.67913800
H	1.71905100	1.26347200	1.20873900
H	3.42380800	3.07939500	1.45673500
H	5.52015700	2.99945700	0.11909300
C	1.86219300	-0.93006800	-0.34729400
C	0.99506600	-1.03396900	-1.55930000
H	0.63074500	-2.03526100	-1.81135700
H	1.11336900	-0.32754100	-2.38433700
C	2.24753700	-2.19170900	0.40324600
H	2.32311600	-1.97203200	1.47678100
H	1.50852700	-2.98954900	0.25872800
H	3.22836200	-2.53458100	0.04675400
C	-5.60174600	2.04935200	-0.60752300
H	-6.19480100	1.62407400	-1.42677100
H	-6.24446300	2.11205200	0.28304400
H	-5.32044000	3.07622500	-0.88173700

1a-T

S	-2.04469300	2.01069200	-0.10937000
O	-2.37956000	3.28795800	-0.71054600
O	-2.79773900	1.45492200	0.99597400
C	-0.31852800	1.96215100	0.24849300
C	2.41524700	1.78985600	0.71272800
C	0.13872200	1.17994600	1.30717700
C	0.55395900	2.68107700	-0.57048700
C	1.91901300	2.58604000	-0.33030600
C	1.50927200	1.10068100	1.52975500
H	-0.57836400	0.64363200	1.93082100
H	0.15617600	3.30438000	-1.37308200
H	2.61752800	3.13544200	-0.96464900
H	1.88650300	0.48308400	2.34739600
C	3.89378500	1.69455700	0.96943300
H	4.47045900	1.89627300	0.05709000
H	4.20024200	2.42973200	1.72987000
H	4.16285300	0.69609300	1.33974300
N	-2.10638200	0.88937300	-1.41104400

I	-1.34660500	-2.07084600	0.19551800
C	0.64757000	-1.68568700	-0.35359600
C	3.28030000	-1.12318200	-1.04709900
C	0.90622600	-0.85871100	-1.44593000
C	1.68657900	-2.23608000	0.39602800
C	3.00552800	-1.95028800	0.04158000
C	2.23022400	-0.57954400	-1.78611000
H	0.08312600	-0.43426000	-2.02295900
H	1.47054400	-2.88350100	1.24642600
H	3.82196800	-2.38473300	0.62128600
H	2.43619600	0.06910600	-2.63925400
H	4.31361200	-0.90503300	-1.32133100

INT1$_T$

S	1.94656300	-0.00086600	-0.07442800
O	2.42666700	-1.27151300	-0.58021500
O	2.43030300	1.27024800	-0.57550500
C	0.18215600	0.00135500	-0.05987500
C	-2.59629400	0.00517500	0.01112800
C	-0.49230800	1.22092400	-0.04351100
C	-0.49629100	-1.21821500	-0.04349200
C	-1.88488100	-1.20430000	-0.00760800
C	-1.88312600	1.21093100	-0.00789600
H	0.07372200	2.15325000	-0.07241900
H	0.06793600	-2.15162800	-0.07267900
H	-2.43271200	-2.14873000	0.00265800
H	-2.42780700	2.15675800	0.00230100
C	-4.10039700	-0.00288800	0.02475100
H	-4.49051300	-0.26916400	-0.96944200
H	-4.50415100	0.98124200	0.29320400
H	-4.48418500	-0.74495800	0.73810700
N	2.26054200	-0.00438400	1.61055600

INT1$_S$

S	1.90817800	0.15460200	-0.08987600
O	2.33327400	1.53232100	-0.13106300
O	2.43572500	-0.84394800	0.97511500
C	0.15179600	0.08980500	-0.05148900
C	-2.62084900	-0.03929700	0.00862400
C	-0.46635800	-1.16045800	-0.11957100
C	-0.57460900	1.27274900	0.03385200
C	-1.96547800	1.19530700	0.06401800
C	-1.85212300	-1.21182900	-0.08351300
H	0.14458900	-2.06104200	-0.20295000
H	-0.05271600	2.22921000	0.07551800
H	-2.55213400	2.11302000	0.13251200
H	-2.35502000	-2.17975900	-0.13224900
C	-4.12206600	-0.12620400	0.04110300
H	-4.45681500	-0.73184500	0.89570700
H	-4.50238400	-0.60844000	-0.87113900
H	-4.58039600	0.86698000	0.12208100
N	2.62430700	-1.10416800	-0.67028800

TS3

S	0.89520000	-0.78943900	-0.89099100
N	-0.00866500	-0.87845800	0.53318600
O	0.27731400	0.21604200	-1.75056200
O	1.11703800	-2.14574600	-1.36955300
C	2.43442300	-0.14011000	-0.30108400
C	3.43936800	-1.02527600	0.07845500
H	3.27889700	-2.09940900	-0.02390300
C	4.63634100	-0.50772000	0.56632300
H	5.43491700	-1.18961200	0.86487000
C	4.83419400	0.87383700	0.67694400
C	3.80115900	1.73729300	0.28340200
H	3.94623000	2.81689500	0.36107100
C	2.59937100	1.24125400	-0.20747700
H	1.79515000	1.90433400	-0.52919100
C	-5.28532500	1.43515300	0.68395700
C	-4.64458200	0.21838500	0.89931800
C	-3.43093900	-0.08229400	0.25651400
C	-2.86125000	0.89169200	-0.58608600
C	-3.50107100	2.10917200	-0.79166300
C	-4.71722400	2.38469800	-0.16432900
H	-6.23279400	1.64339200	1.18316100
H	-5.10330600	-0.51306600	1.56566100
H	-1.89297500	0.71035800	-1.05691500
H	-3.03962900	2.85329000	-1.44240800
H	-5.21632700	3.34071900	-0.32956800
C	-2.75998400	-1.37396100	0.48176700
C	-1.79416700	-1.83298600	-0.37518700
H	-1.65083200	-1.37767400	-1.35619500
H	-1.34798500	-2.81708400	-0.22469100
C	-3.09581300	-2.15213500	1.71939000

C	6.13691700	1.43216300	1.18293900
H	6.73924700	0.65881200	1.67603300
H	6.72924300	1.84257000	0.35095200
H	5.96517400	2.24841800	1.89806800
H	-2.42289200	-3.01007100	1.83610700
H	-4.12853000	-2.53342000	1.67846000
H	-3.01946500	-1.51558200	2.61387100

INT3$_T$

S	-1.12327900	-1.23744400	0.00051700
N	-0.07562900	0.09128600	0.00011800
O	-0.93920500	-1.92591800	1.26788600
O	-0.93910200	-1.92695700	-1.26627100
C	-2.70375600	-0.45019000	0.00012600
C	-3.30815300	-0.14841200	-1.21711400
H	-2.81069900	-0.41842800	-2.14956000
C	-4.54704200	0.48453900	-1.20633700
H	-5.03401600	0.72918900	-2.15241100
C	-5.18245400	0.80972500	-0.00049800
C	-4.54709100	0.48558700	1.20569000
H	-5.03413100	0.73106700	2.15152300
C	-3.30823100	-0.14733500	1.21709700
H	-2.81082300	-0.41653900	2.14980200
C	6.00974300	1.22614500	-0.00007000
C	4.65485800	1.52294200	0.00005500
C	3.67504600	0.49557400	-0.00011800
C	4.14482000	-0.84477100	-0.00042000
C	5.50316300	-1.13063800	-0.00054300
C	6.44744300	-0.10142200	-0.00037000
H	6.73742400	2.03940800	0.00007000
H	4.33922000	2.56632800	0.00029500
H	3.43879200	-1.67553600	-0.00056600
H	5.83135700	-2.17131100	-0.00077700
H	7.51351500	-0.33111000	-0.00046700
C	2.27710000	0.80978800	0.00000800
C	1.29576600	-0.32944400	-0.00012000
H	1.45934300	-1.00032200	0.87389700
H	1.45907200	-0.99990100	-0.87451800
C	1.80436500	2.22697500	0.00025000
H	2.17716400	2.77175400	-0.88366200
H	0.71080400	2.27832300	0.00017900
H	2.17701500	2.77140700	0.88444300
C	-6.53819400	1.46307400	-0.00068500
H	-6.68060400	2.08511100	-0.89395300
H	-7.33153300	0.69965500	0.00320900
H	-6.67785600	2.09118700	0.88878000

INT3$_S$

S	1.09469600	-1.21741200	-0.00089500
N	0.07887300	0.10702500	-0.00128200
O	0.92384300	-1.92886300	-1.26244000
O	0.92272700	-1.92934600	1.26023500
C	2.68550900	-0.44318100	-0.00002400
C	3.29367500	-0.14727000	1.21615100
H	2.79240400	-0.41452000	2.14737600
C	4.53855600	0.47465000	1.20655200
H	5.02794800	0.71421900	2.15285400
C	5.17724200	0.79468600	0.00132400
C	4.53889000	0.47655800	-1.20462900
H	5.02856900	0.71763600	-2.15040400
C	3.29405400	-0.14534700	-1.21558200
H	2.79309300	-0.41115300	-2.14738700
C	-6.01671700	1.23536600	0.00095200
C	-4.66070400	1.53318300	0.00026900
C	-3.68135600	0.50883100	-0.00010700
C	-4.15174400	-0.83148800	0.00022200
C	-5.50740700	-1.12015200	0.00088100
C	-6.45308000	-0.09061400	0.00126200
H	-6.74356100	2.04899500	0.00124900
H	-4.35525000	2.57829800	0.00006600
H	-3.44454600	-1.66128000	-0.00008300
H	-5.83356400	-2.16119300	0.00109900
H	-7.51891900	-0.32119800	0.00179900
C	-2.27449900	0.80487600	-0.00075400
C	-1.27831500	-0.29632900	-0.00085600
H	-1.46250800	-0.96781900	-0.87181000
H	-1.46227600	-0.96844100	0.86958200
C	-1.69328500	2.16704800	-0.00158100
H	-1.01770800	2.27256500	0.86595600
H	-1.01834800	2.27180700	-0.86970200
H	-2.42703700	2.97710600	-0.00169000
C	6.53957600	1.43520800	0.00192000
H	6.68746500	2.05644600	0.89500400
H	7.32657900	0.66521800	-0.00152500

H	6.68547100	2.06225200	-0.88744600

TS4

S	-1.17464300	1.36512000	0.76394700
N	-0.04161500	1.09724000	-0.36361300
O	-1.64089900	2.73488400	0.61472900
O	-0.72696100	0.88406200	2.07914800
C	-2.53582900	0.30563200	0.28933500
C	-2.74578700	-0.89747200	0.95211900
H	-2.10258100	-1.15970800	1.79342900
C	-3.79002700	-1.72736600	0.54071900
H	-3.96483600	-2.67204700	1.06050400
C	-4.62449000	-1.36410200	-0.51961700
C	-4.39485900	-0.13809000	-1.16345100
H	-5.04721200	0.16354600	-1.98656100
C	-3.36043100	0.69923100	-0.76499300
H	-3.18410800	1.66167100	-1.24796800
C	5.81601200	-0.62285600	-0.84617400
C	4.62554600	0.03812400	-1.12300200
C	3.48314600	-0.17674700	-0.32741000
C	3.56834800	-1.08194300	0.75066200
C	4.75852400	-1.74210400	1.02279900
C	5.88399800	-1.51213400	0.22728000
H	6.69226600	-0.45089400	-1.47184300
H	4.56652300	0.71285200	-1.97747100
H	2.70130100	-1.25519400	1.38832400
H	4.81476100	-2.43418200	1.86342300
H	6.81862600	-2.03113600	0.44540500
C	2.24312400	0.52031000	-0.62697200
C	0.94323500	0.13543800	-0.02067700
H	1.09045800	0.01717300	1.07553700
H	0.71337400	-0.89230800	-0.38908200
C	2.13327700	1.69752600	-1.41001500
H	1.40976900	1.59783100	-2.23683700
H	1.20410500	2.14589800	-0.76521700
H	3.03586300	2.25476800	-1.66644700
C	-5.76004700	-2.25009800	-0.96024700
H	-5.76381000	-3.19902300	-0.40865300
H	-6.72905100	-1.75629100	-0.79355300
H	-5.68941300	-2.47720100	-2.03399600

10a

S	2.33285700	-1.39970200	0.39611300
N	1.32685300	-2.13314600	-0.70260700
O	3.66108600	-1.51724400	-0.17320100
O	1.98087600	-1.92952200	1.69902700
C	1.86588300	0.31615900	0.39417300
C	0.84600300	0.74128800	1.24135500
H	0.43432900	0.05256700	1.98105800
C	0.36872200	2.04291600	1.11874400
H	-0.44120800	2.38093100	1.76897800
C	0.90358400	2.92170300	0.16829600
C	1.94844600	2.47161000	-0.65080900
H	2.38479800	3.15225000	-1.38496600
C	2.43537900	1.17235300	-0.54409000
H	3.24691000	0.81252700	-1.17927300
C	-3.90870100	0.97301000	0.11679700
C	-2.65983000	0.69530800	-0.43070600
C	-2.18449200	-0.62334000	-0.50822900
C	-2.98982800	-1.65136200	0.00342600
C	-4.24138200	-1.37334400	0.55061400
C	-4.70655300	-0.06131500	0.60771700
H	-4.25794500	2.00538500	0.17149300
H	-2.02300200	1.51000400	-0.78111600
H	-2.64750200	-2.68672300	-0.03791500
H	-4.85571600	-2.18911800	0.93449400
H	-5.68376200	0.15651900	1.04080800
C	-0.84822700	-0.91260900	-1.09057700
C	-0.10479800	-2.10262800	-0.49749000
H	-0.53182700	-3.03576600	-0.90087200
H	-0.27312600	-2.15014300	0.58965700
C	-0.33626400	-0.17524600	-2.08142300
H	0.66631700	-0.35841300	-2.47196200
H	1.69063100	-2.10130800	-1.65042900
H	-0.90136500	0.64043500	-2.53490800
C	0.38956600	4.33224200	0.05639400
H	0.90432600	4.98882000	0.77474300
H	0.55910300	4.74113900	-0.94822600
H	-0.68539000	4.38179300	0.27710500

TS5

S	-1.14215900	-1.07018400	0.65056200
N	-0.05163300	0.02241200	-0.00105300
O	-1.01793300	-0.96174800	2.09828400

O	-0.97119300	-2.35948400	-0.00433000
C	-2.69098800	-0.37848300	0.15383400
C	-3.27709000	-0.81203600	-1.03045000
H	-2.78626900	-1.58415600	-1.62431700
C	-4.49128800	-0.25118900	-1.41944000
H	-4.96361400	-0.58123100	-2.34670200
C	-5.11839800	0.72587900	-0.63791600
C	-4.50289200	1.13348200	0.55473700
H	-4.98664400	1.89031900	1.17591500
C	-3.29173700	0.58562000	0.96054300
H	-2.81205900	0.88337600	1.89390700
C	5.94847400	1.24991300	-0.39308900
C	4.61424000	1.46962900	-0.07874600
C	3.67149700	0.41106600	-0.08973800
C	4.15100700	-0.87720600	-0.44392400
C	5.48692500	-1.08909800	-0.75019000
C	6.39778400	-0.02899900	-0.72845600
H	6.64751100	2.08762200	-0.38095500
H	4.28752800	2.48031100	0.16481400
H	3.46907700	-1.72829900	-0.46553600
H	5.82494500	-2.09340700	-1.01007600
H	7.44658500	-0.19871500	-0.97432700
C	2.29602700	0.63342800	0.25850600
C	1.29224300	-0.46020000	0.10122400
H	1.42006400	-1.21652300	0.90569900
H	1.47791500	-1.02489600	-0.83645600
C	1.78921100	1.93712300	0.77111800
H	1.41388100	2.57711800	-0.04715000
H	0.92318900	1.75817100	1.42731000
H	2.55587400	2.49601000	1.32170600
C	-6.44144000	1.31528900	-1.04743400
H	-6.68206700	1.07261200	-2.09014100
H	-7.25131400	0.92377000	-0.41333300
H	-6.43708300	2.40854100	-0.93706400

TS6

S	-0.96687900	-0.71791000	0.72404100
O	-0.29510900	0.33538100	1.47587400
O	-1.09555400	-2.06114100	1.26584100
C	-2.56669700	-0.13917900	0.24485900
C	-5.07098500	0.76627300	-0.55771300
C	-3.56646400	-1.06897700	-0.02960800
C	-2.78657600	1.23218000	0.13185400
C	-4.04177200	1.67409100	-0.27109600
C	-4.81536200	-0.60536100	-0.43109300
H	-3.36031300	-2.13408000	0.08439500
H	-1.98298400	1.93062400	0.36911300
H	-4.23033400	2.74552300	-0.36389500
H	-5.60999500	-1.32157800	-0.64899100
C	-6.43365600	1.26027900	-0.96181200
H	-6.36234200	2.17857200	-1.55954200
H	-7.03743600	1.49080100	-0.07047200
H	-6.97543800	0.50460500	-1.54498700
N	-0.16173700	-0.84354300	-0.75419800
C	3.35968800	2.21563700	0.42141400
C	2.87281600	0.92144300	0.24278900
C	3.70783400	-0.09319600	-0.24729200
C	5.04654700	0.21913000	-0.52807100
C	5.53129300	1.51042500	-0.34584100
C	4.68728400	2.51652900	0.12612300
H	2.69125100	2.99070100	0.79905000
H	1.83004700	0.71292600	0.48856400
H	5.72360800	-0.56542500	-0.86968500
H	6.57761100	1.73068900	-0.56323800
H	5.06775700	3.52858200	0.27204900
C	3.17857600	-1.46883700	-0.45983700
C	3.70995200	-2.30382900	-1.38001600
H	4.54149600	-2.00300900	-2.01853800
H	3.30652400	-3.30855500	-1.51829900
C	2.00520700	-1.88615500	0.31452700
H	0.98857000	-1.37548600	-0.29656600
H	1.76097800	-2.95293500	0.26957500
H	1.89242100	-1.45722000	1.31735100

$INT5_T$

S	-0.72465700	-0.58230900	0.31108100
O	-0.19664300	0.14197600	1.46040900
O	-0.63776500	-2.03202500	0.22548300
C	-2.41219500	-0.10788000	0.08585900
C	-5.06075400	0.65134000	-0.29816700
C	-3.28046900	-0.98374300	-0.55893000
C	-2.83441800	1.13530900	0.55058600
C	-4.15968200	1.50641800	0.35128900
C	-4.60312300	-0.59372000	-0.74764700

H	-2.91740100	-1.95598800	-0.89463900
H	-2.12959700	1.78883700	1.06621300
H	-4.50516700	2.47861900	0.70857800
H	-5.29588000	-1.26991500	-1.25242000
C	-6.50009500	1.05259200	-0.47745500
H	-6.59420600	2.13522300	-0.63459300
H	-7.08448800	0.79561000	0.41952000
H	-6.95535700	0.53477100	-1.33145900
N	0.01573100	0.05913700	-1.05041000
C	3.62140400	2.23892000	0.36028200
C	2.97722400	1.02108100	0.57523600
C	3.49035600	-0.16059500	0.02071600
C	4.65687100	-0.09411700	-0.75249700
C	5.30079300	1.12304900	-0.96190200
C	4.78453500	2.29380000	-0.40597300
H	3.20651800	3.15141500	0.79131800
H	2.05202900	0.98207600	1.15622400
H	5.07047100	-1.01199800	-1.17513700
H	6.21422900	1.15729600	-1.55786900
H	5.28848100	3.24727400	-0.57112600
C	2.77875000	-1.45848500	0.23153600
C	2.59661800	-2.31539600	-0.84536300
H	2.97132600	-2.06426400	-1.83870600
H	2.05651000	-3.25428900	-0.71792500
C	2.26144700	-1.75090300	1.49333500
H	0.98579100	-0.30248200	-0.99218400
H	1.67215900	-2.65421200	1.65032800
H	2.42067600	-1.08061200	2.33688900

Styrene

C	1.35341600	1.33018200	0.00003500
C	-0.01560400	1.08741300	-0.00001300
C	-0.51215200	-0.22583000	-0.00003300
C	0.40865800	-1.28247200	-0.00001100
C	1.78134400	-1.04184000	0.00003800
C	2.25902200	0.26667100	0.00006200
H	1.71964400	2.35804800	0.00004900
H	-0.70832500	1.93032300	-0.00004200
H	0.03708900	-2.30967500	-0.00003000
H	2.47951900	-1.88029300	0.00005600
H	3.33260600	0.46043100	0.00009900
C	-1.95496500	-0.53550000	-0.00007700
H	-2.19338500	-1.60396300	-0.00017500
C	-2.96400100	0.33960900	-0.00000400
H	-2.80223600	1.41957800	0.00010500
H	-3.99921300	-0.00384500	-0.00004800

TS7

S	-0.74234700	-1.04968300	0.59925800
N	0.09790300	-0.90239400	-0.86192400
O	-0.03452000	-0.26379800	1.60522400
O	-1.01798000	-2.46198700	0.81427600
C	-2.26209900	-0.22477300	0.21874800
C	-3.32593900	-0.96939800	-0.28468700
H	-3.21996000	-2.04842400	-0.40503200
C	-4.50800300	-0.31008100	-0.60750300
H	-5.35149400	-0.88058400	-1.00127900
C	-4.63460600	1.07415800	-0.43300600
C	-3.54400500	1.79259000	0.07705400
H	-3.63207900	2.87158100	0.21951800
C	-2.35433300	1.15307200	0.40702400
H	-1.50448200	1.70092600	0.81635300
C	5.43861400	1.29949400	-0.76284300
C	4.73267000	0.17539300	-1.17794700
C	3.57437600	-0.23930200	-0.49735300
C	3.12040100	0.52320200	0.59653300
C	3.82652000	1.64949100	1.00335000
C	4.98860500	2.03807500	0.33214300
H	6.34049000	1.60388900	-1.29580600
H	5.08079400	-0.40283900	-2.03666300
H	2.19266400	0.25154100	1.10536200
H	3.46215500	2.23587800	1.84803000
H	5.53791600	2.92288100	0.65743600
C	2.86713700	-1.42994500	-0.94980500
C	1.87722900	-2.06831900	-0.25711800
H	1.71917300	-1.85817000	0.80247000
H	1.43496600	-2.98683200	-0.64340100
C	-5.92668500	1.77511000	-0.75506900
H	-6.50727100	1.21876100	-1.50213700
H	-6.54788300	1.86872600	0.14913100
H	-5.74465400	2.78824900	-1.13739400
H	3.13259700	-1.80020800	-1.94438400

INT6$_T$

S	-1.04796200	-1.19107500	0.00018900
N	0.03445800	0.11074100	-0.00075700
O	-0.88054700	-1.88577200	-1.26639600
O	-0.87997700	-1.88454900	1.26736700
C	-2.60914000	-0.36665400	0.00015000
C	-3.20689500	-0.05127800	1.21715700
H	-2.71552400	-0.33165400	2.14976700
C	-4.43172200	0.60846300	1.20608200
H	-4.91330000	0.86405000	2.15202000
C	-5.05981900	0.94687000	0.00010600
C	-4.43144800	0.60875100	-1.20593300
H	-4.91288600	0.86460200	-2.15188700
C	-3.20671100	-0.05094600	-1.21696700
H	-2.71519800	-0.33112300	-2.14956400
C	5.75027200	-0.78600500	-0.00028000
C	4.36854800	-0.65552900	-0.00042100
C	3.75929600	0.62698800	-0.00012200
C	4.61304800	1.76161000	0.00032700
C	5.99073600	1.61955000	0.00046700
C	6.57092800	0.34532100	0.00016500
H	6.19672100	-1.78164600	-0.00051000
H	3.74774900	-1.55310900	-0.00075800
H	4.16335900	2.75661300	0.00056400
H	6.62606700	2.50666600	0.00081700
H	7.65599700	0.23605700	0.00027900
C	2.35080800	0.79246900	-0.00025000
H	1.92134600	1.79526700	0.00007600
C	1.39719500	-0.35135100	-0.00065100
H	1.55016000	-1.02220900	-0.87680300
H	1.55008700	-1.02278200	0.87505600
C	-6.40113700	1.62937100	-0.00029200
H	-6.53243100	2.25049800	0.89529500
H	-7.21078800	0.88333500	-0.00970000
H	-6.52474400	2.26422600	-0.88736700

INT6$_S$

S	-1.04200000	-1.17231000	0.00014000
N	0.02313800	0.14091200	-0.00024700
O	-0.86916200	-1.86776400	-1.26582500
O	-0.86902200	-1.86730000	1.26634000
C	-2.61180700	-0.36385200	0.00008600
C	-3.21253200	-0.05405100	1.21699000
H	-2.71818600	-0.32938300	2.14952600
C	-4.44377300	0.59371300	1.20596700
H	-4.92767500	0.84489100	2.15191200
C	-5.07530200	0.92570700	0.00002300
C	-4.44386800	0.59328200	-1.20596500
H	-4.92790700	0.84415300	-2.15193800
C	-3.21270200	-0.05442900	-1.21692700
H	-2.71843000	-0.33007300	-2.14941100
C	5.74131100	-0.80945000	-0.00030100
C	4.36183200	-0.66063700	-0.00017300
C	3.76938400	0.62978600	0.00001500
C	4.63835500	1.75238800	0.00006800
C	6.01444700	1.59219600	-0.00006400
C	6.57758900	0.31088100	-0.00025000
H	6.17417800	-1.81106800	-0.00043900
H	3.72980900	-1.55039300	-0.00020700
H	4.20226500	2.75342300	0.00021500
H	6.66113900	2.47105000	-0.00002000
H	7.66105400	0.18691800	-0.00035100
C	2.36312500	0.81945800	0.00018300
H	1.95606400	1.83198000	0.00043900
C	1.38752500	-0.30363900	0.00022400
H	1.53993300	-0.97337100	-0.87524200
H	1.53951200	-0.97289900	0.87613100
C	-6.42314300	1.59531400	-0.00039400
H	-6.56052900	2.21492100	0.89533800
H	-7.22570100	0.84165200	-0.01019800
H	-6.55265400	2.22927500	-0.88727900

TS8

S	-1.11346000	1.34226700	-0.24348300
N	0.10948100	0.18613400	-0.13125400
O	-0.98053300	2.23482900	0.89836300
O	-1.08476300	1.86559800	-1.60139800
C	-2.56611600	0.35877600	-0.03684900
C	-3.15033200	-0.22810600	-1.15697800
H	-2.71808000	-0.05731000	-2.14381000
C	-4.28538100	-1.01158700	-0.98243100
H	-4.75491700	-1.47944900	-1.85025300
C	-4.84073900	-1.20762900	0.29016900
C	-4.23112600	-0.59694100	1.39271000
H	-4.65654600	-0.73908500	2.38803200

C	-3.09349800	0.19108700	1.23936900
H	-2.61774000	0.68204100	2.08927400
C	5.51943200	-0.01565200	1.34351700
C	4.22734400	0.24350200	0.91056500
C	3.71607500	-0.36742000	-0.26443000
C	4.57390200	-1.25659000	-0.96573700
C	5.86131500	-1.51174700	-0.52321900
C	6.34580300	-0.89242800	0.63428100
H	5.88952400	0.46777400	2.24892500
H	3.60225100	0.92546900	1.48821800
H	4.19979700	-1.73982000	-1.87054500
H	6.49886200	-2.19920900	-1.08118900
H	7.35931200	-1.09411800	0.98270300
C	2.41281600	-0.11862300	-0.77006200
H	2.09988800	-0.64756100	-1.67228100
C	1.40997100	0.80978700	-0.18551000
H	1.66361900	1.10943700	0.84852300
H	1.38182500	1.75736600	-0.76363800
C	-6.08694400	-2.03501900	0.45631800
H	-6.06209300	-2.92255700	-0.19033700
H	-6.97706400	-1.44955100	0.17934100
H	-6.21239900	-2.36451200	1.49554800

22a

S	-0.93678000	1.98532800	0.01826600
N	0.59725100	1.49006400	0.54678600
O	-1.48181800	2.80245700	1.08091100
O	-0.83405400	2.50795400	-1.33931100
C	-1.76953600	0.42634200	-0.03142900
C	-1.96323700	-0.20731300	-1.25313700
H	-1.62413900	0.27460500	-2.17110000
C	-2.60548300	-1.44467500	-1.26951500
H	-2.76599100	-1.95445800	-2.22130700
C	-3.05040200	-2.04159100	-0.08595400
C	-2.84614800	-1.36852900	1.12917400
H	-3.19724500	-1.82261100	2.05829200
C	-2.21107800	-0.13443400	1.16703600
H	-2.05835200	0.40339600	2.10391300
C	3.30413300	-1.73424200	1.33475700
C	2.46810200	-0.64765400	1.08641300
C	2.42806300	-0.06967400	-0.18602900
C	3.22495700	-0.59818300	-1.20564900
C	4.06109000	-1.68443100	-0.95528700
C	4.10381300	-2.25404300	0.31678600
H	3.32769200	-2.18207800	2.32949200
H	1.82535400	-0.24266400	1.86996000
H	3.18844900	-0.15442000	-2.20308700
H	4.67938500	-2.08952400	-1.75779300
H	4.75641100	-3.10587400	0.51348200
C	1.56504700	1.11334900	-0.47013700
H	1.23427100	1.22823000	-1.50855600
C	1.68823100	2.40636900	0.26524500
H	2.41639300	2.50459600	1.07353000
H	1.44976700	3.31261700	-0.30004000
C	-3.74770300	-3.37519400	-0.10042100
H	-3.77508700	-3.80214600	-1.11065200
H	-4.78301500	-3.27595400	0.25774100
H	-3.23828700	-4.08927300	0.56245100

TS9

S	-2.59443500	0.22645700	-0.24186100
N	-2.35847200	-1.20447200	-0.86680600
O	-3.04608700	0.24755700	1.16604800
O	-3.37511600	1.02417400	-1.20456700
C	-0.98063900	1.06521000	-0.16168200
C	-0.11868900	0.96678500	-1.25378300
H	-0.45017300	0.40047900	-2.12699200
C	1.14946500	1.53315200	-1.19071900
H	1.83377800	1.43008500	-2.03782900
C	1.58314800	2.20141000	-0.03703700
C	0.70092600	2.30507200	1.04150400
H	1.02524200	2.82294800	1.94896800
C	-0.57666200	1.74252100	0.98287900
H	-1.27033800	1.79161100	1.82417900
C	3.14718200	-1.02997900	0.68554100
C	1.89668900	-1.11962700	1.29116900
C	0.79690900	-1.67759600	0.61507300
C	0.99193500	-2.11227300	-0.71192300
C	2.24219200	-2.02355000	-1.31381700
C	3.33053000	-1.48602900	-0.62079300
H	3.98514800	-0.59446300	1.23533200
H	1.75226400	-0.74349300	2.30684500
H	0.12445900	-2.45242700	-1.28110900
H	2.36596700	-2.35437000	-2.34759100

H	4.30858000	-1.41055700	-1.10036200
C	-0.51501700	-1.73242100	1.25081400
C	-1.58443900	-2.41427000	0.75558400
H	-2.53887500	-2.37003800	1.27735200
H	-1.46132600	-3.19432800	0.00517000
C	2.98815000	2.73969200	0.04339300
H	3.24382100	3.33189800	-0.84818900
H	3.12360200	3.37632300	0.92900400
H	3.71247500	1.91126700	0.10577100
H	-0.67482900	-1.05937200	2.09781300

INT7

S	-2.54777300	0.15204200	-0.30938500
N	-2.14130600	-1.36311900	-0.29006200
O	-3.28605000	0.60200900	0.89134300
O	-3.10849100	0.50190500	-1.61795900
C	-0.99112900	1.09033000	-0.22008100
C	-0.12032000	1.05141600	-1.30942100
H	-0.46090600	0.57586900	-2.23175500
C	1.15917500	1.58401000	-1.19409700
H	1.84982100	1.52858500	-2.04052000
C	1.59267500	2.16465100	0.00620400
C	0.69565500	2.22669900	1.07613100
H	1.01538300	2.68809000	2.01520300
C	-0.59012400	1.69237700	0.96747200
H	-1.29952500	1.71448400	1.79709000
C	3.21620700	-1.16517800	0.49196000
C	2.04897000	-1.13419200	1.24143000
C	0.80881700	-1.56253800	0.69856000
C	0.80710100	-2.02008200	-0.64858600
C	1.98261700	-2.05048700	-1.38529700
C	3.19465100	-1.62323800	-0.83022900
H	4.15519100	-0.82729500	0.93757100
H	2.06838100	-0.76188100	2.26893800
H	-0.15301800	-2.28640400	-1.09383900
H	1.95472400	-2.39778100	-2.42077900
H	4.11190300	-1.64374100	-1.42201300
C	-0.37528000	-1.51448600	1.48635600
C	-1.76171700	-1.87920200	1.00806700
H	-2.47566600	-1.58487800	1.80062200
H	-1.81490700	-2.98627700	0.94395900
C	3.00948300	2.65804800	0.14250900
H	3.31893400	3.24344400	-0.73618300
H	3.13151500	3.28672500	1.03583600
H	3.70262600	1.80526200	0.23029300
H	-0.25530800	-1.18512400	2.52322500

INT8

S	-0.16419200	-1.80299700	-0.38729400
N	0.34227000	-1.89646800	1.12973300
O	-0.76555900	-3.07626100	-0.74854300
O	0.85077000	-1.25860200	-1.30914300
C	-1.47637400	-0.57191000	-0.36822900
C	-1.25359000	0.70727500	-0.86847100
H	-0.29974400	0.92108900	-1.35543300
C	-2.26230500	1.66939600	-0.76204700
H	-2.09757100	2.67285600	-1.16149600
C	-3.48690500	1.36332500	-0.16371800
C	-3.68897100	0.06075800	0.32423800
H	-4.64766100	-0.19385900	0.78263800
C	-2.69686100	-0.90661800	0.22153200
H	-2.84874100	-1.92501200	0.58561100
C	4.03625100	-0.09249500	-0.37446500
C	2.82722700	-0.36346100	0.25011900
C	1.99941300	0.71693400	0.67048500
C	2.39314900	2.05421500	0.37191300
C	3.57832200	2.30464500	-0.28584600
C	4.39953200	1.22525500	-0.65491500
H	4.68056300	-0.91479000	-0.68458100
H	2.49038700	-1.38329700	0.42544100
H	1.74557800	2.87680500	0.68319200
H	3.87958300	3.32621000	-0.51718400
H	5.33481500	1.42307700	-1.18230300
C	0.85795500	0.52945500	1.46656100
H	0.34107200	1.44704100	1.77531800
C	0.28126100	-0.74360700	1.95141200
H	-0.74837000	-0.50177000	2.29771000
H	0.82016200	-0.94837000	2.90573500
C	-4.58543600	2.38816700	-0.06135000
H	-4.23086200	3.38578400	-0.35068800
H	-5.42589600	2.12369500	-0.72057900
H	-4.97982700	2.44467300	0.96335500

TS10

S	2.55446500	0.03149900	0.33885500
N	2.03203000	-1.45472900	0.05857000
O	3.34581100	0.57018600	-0.77617500
O	3.07194500	0.09492500	1.69624100
C	1.04566900	1.03611500	0.32274300
C	0.14282900	0.91584700	1.37937300
H	0.44573500	0.36354200	2.27184900
C	-1.12427900	1.47514600	1.26072900
H	-1.84348700	1.36735500	2.07751600
C	-1.50406300	2.18248500	0.10416100
C	-0.56895400	2.32618500	-0.92261500
H	-0.83634500	2.89737000	-1.81535400
C	0.70375700	1.75347100	-0.82016200
H	1.45088400	1.86028100	-1.61032200
C	-1.99894200	-2.30947200	1.17996800
C	-0.82503700	-2.05095700	0.49571300
C	-0.87297300	-1.32253400	-0.72681800
C	-2.11420000	-0.81020400	-1.18585600
C	-3.28166400	-1.06756700	-0.48408600
C	-3.21954900	-1.82151200	0.69166200
H	-1.97276300	-2.87372700	2.11233900
H	0.15983700	-2.34503900	0.86959700
H	-2.13813000	-0.22209900	-2.10574000
H	-4.23741800	-0.68522600	-0.84276800
H	-4.13725500	-2.02061100	1.24875100
C	0.26194200	-1.13924000	-1.54571700
H	0.07939500	-0.55737600	-2.45873100
C	1.63754400	-1.66153400	-1.30578200
H	2.32068600	-1.21481900	-2.05180300
H	1.60403400	-2.74952800	-1.53060800
C	-2.88189400	2.78088300	0.00420600
H	-3.65478100	2.02866800	0.22456500
H	-3.00838200	3.59755900	0.73067000
H	-3.07083300	3.18921900	-0.99726600

INT9

S	-1.19513700	-1.36365100	-0.02631600
N	-0.14516900	-0.05902100	-0.10142900
O	-0.99075800	-2.03721900	1.24923800
O	-1.07559200	-2.10636400	-1.27412900
C	-2.76556000	-0.55218800	0.00445400
C	-3.39852700	-0.25757800	-1.19980500

H	-2.92978300	-0.54917700	-2.14061800
C	-4.62647400	0.39540300	-1.16556900
H	-5.13500200	0.63473800	-2.10176600
C	-5.22434500	0.74764400	0.05198500
C	-4.56203700	0.42992900	1.24477700
H	-5.01975900	0.69599300	2.19971100
C	-3.33285200	-0.22272900	1.23162300
H	-2.81391800	-0.48774200	2.15377200
C	5.94836700	1.02200000	-0.03044700
C	4.60003800	1.30489000	-0.20237900
C	3.61928600	0.28285300	-0.13891400
C	4.07809000	-1.03724000	0.11239100
C	5.42715100	-1.31128300	0.27784100
C	6.37489200	-0.28590200	0.20808300
H	6.67690900	1.83268300	-0.08041200
H	4.29514500	2.33684400	-0.37501300
H	3.36798300	-1.86294600	0.16891400
H	5.74688900	-2.33778300	0.46343100
H	7.43479500	-0.50528900	0.34100300
C	2.22421200	0.57620300	-0.31770500
C	1.20853300	-0.48925300	-0.09101900
H	1.39743100	-1.01396800	0.87231500
H	1.34633000	-1.29820600	-0.84649400
C	1.71334500	1.93771800	-0.64977200
H	0.81561600	1.81804700	-1.27715400
H	2.45334000	2.50363900	-1.23141800
C	-6.56943500	1.42281900	0.07683700
H	-6.70703500	2.06851000	-0.80050900
H	-7.37582300	0.67325600	0.06704300
H	-6.69309900	2.03209400	0.98159900
C	1.31461000	2.71963700	0.61240600
H	0.54531700	2.16916500	1.17137400
H	2.18348500	2.86910000	1.26950900
H	0.90460000	3.70374100	0.34618000

TS11

S	-0.53754900	-0.42664400	0.32745600
N	-0.16417900	1.09669400	0.71401700
O	-0.30055500	-1.34364400	1.45759800
O	0.08262000	-0.84435400	-0.94615200
C	-2.28941400	-0.31250600	0.04599600
C	-2.80999000	-0.74815200	-1.16455100

H	-2.12632000	-1.10814500	-1.93440500
C	-4.19154100	-0.70734000	-1.36028400
H	-4.61134800	-1.04501200	-2.31026300
C	-5.04703600	-0.24046500	-0.35953700
C	-4.48939300	0.19234700	0.85444000
H	-5.14816800	0.56231000	1.64366600
C	-3.11747700	0.15786000	1.06504400
H	-2.67329100	0.49962500	2.00084900
C	5.25931900	-0.56573000	-0.87597800
C	4.30859600	0.42493500	-0.67477300
C	3.13418500	0.14724500	0.05398800
C	2.91625600	-1.15327500	0.55685500
C	3.85992500	-2.14545500	0.32129000
C	5.03007100	-1.85247800	-0.38168200
H	6.17722100	-0.34143300	-1.41992200
H	4.48160900	1.43401300	-1.05202200
H	1.98376400	-1.40108800	1.07062100
H	3.67782600	-3.15676600	0.68510600
H	5.77110000	-2.63582100	-0.55012200
C	2.15700000	1.19649500	0.26576600
C	1.13982500	1.15091100	1.38460500
H	1.20500400	2.08679700	1.96326900
H	1.32426500	0.31803600	2.07802400
C	1.85956100	2.17567800	-0.71101200
H	0.77413800	1.57223600	-0.67731800
H	2.39614300	2.07727200	-1.65853200
C	-6.53889800	-0.20155300	-0.56210300
H	-6.81192900	-0.50423000	-1.58110900
H	-7.04639400	-0.87810900	0.14167300
H	-6.93390000	0.80965300	-0.38633400
C	1.37136800	3.57692000	-0.36775900
H	0.58846600	3.54441300	0.39889800
H	2.20687100	4.20180400	-0.02010600
H	0.94431500	4.04525000	-1.26279000

TS12

S	-0.59506100	-0.47795000	0.24564600
N	-0.26631100	0.65332000	1.34716400
O	-0.36644400	-1.83700900	0.76894100
O	0.07703300	-0.20277100	-1.04475300
C	-2.33874600	-0.24606600	-0.00312100
C	-2.81783800	-0.05004600	-1.29097800

H	-2.10714200	0.00610400	-2.11651100
C	-4.19409600	0.07376600	-1.48825000
H	-4.58189900	0.23053600	-2.49707400
C	-5.08474900	0.00105400	-0.41445600
C	-4.56832900	-0.19906300	0.87590300
H	-5.25500600	-0.25486800	1.72387200
C	-3.20219200	-0.32611300	1.08930500
H	-2.78932100	-0.47324700	2.08810400
C	5.26807400	-0.15431000	-0.74047300
C	4.28004200	0.66164800	-0.20678900
C	3.06754300	0.10528700	0.24992600
C	2.85050200	-1.28573800	0.13272800
C	3.82915000	-2.08530500	-0.44318200
C	5.03676900	-1.52614900	-0.86699100
H	6.21526500	0.27577800	-1.06691000
H	4.45390600	1.73242000	-0.10292200
H	1.89347000	-1.72235600	0.43118600
H	3.64823600	-3.15368000	-0.56220100
H	5.80579100	-2.16555200	-1.30397900
C	2.06329100	0.95692700	0.84777300
C	1.07888600	0.43419400	1.88172300
H	1.18912400	1.04099900	2.79602100
H	1.29552000	-0.61133200	2.14858100
C	1.70434100	2.25683700	0.40005500
H	0.56983100	1.81548300	0.34105300
H	1.43321400	2.93256400	1.22675100
C	-6.57087600	0.13014100	-0.62168600
H	-6.81242300	0.32225500	-1.67489600
H	-7.08991800	-0.79005700	-0.31486300
H	-6.98164400	0.95378300	-0.01954600
C	2.22730200	2.91622900	-0.85249000
H	3.16562500	3.46428200	-0.68627100
H	2.38653900	2.16887700	-1.64152500
H	1.48681600	3.64044700	-1.21684200

TS13

S	-1.39857700	-1.82874500	0.37660200
N	-0.17998400	-1.37636600	-0.64274100
O	-0.89272300	-1.92960700	1.74784200
O	-2.08323400	-2.94615400	-0.23991300
C	-2.48691000	-0.41913600	0.31785900
C	-3.29065800	-0.23723600	-0.80874600

H	-3.26583400	-0.97667800	-1.61085900
C	-4.11354000	0.87973300	-0.87289500
H	-4.74770600	1.03252100	-1.74917100
C	-4.15126500	1.81421500	0.17428900
C	-3.34305100	1.59806100	1.29403500
H	-3.37072300	2.31064400	2.12099900
C	-2.50859900	0.48284900	1.37519500
H	-1.89010000	0.29402900	2.25384100
C	5.22456000	1.54299500	-0.15299300
C	4.12056300	0.97412400	-0.78287200
C	3.21953200	0.16758100	-0.06674400
C	3.47852500	-0.07223100	1.29571900
C	4.58598500	0.49085300	1.91879600
C	5.46246700	1.30482400	1.19908200
H	5.90355700	2.17633500	-0.72547400
H	3.94376300	1.17980800	-1.83927600
H	2.82494800	-0.73109000	1.86981500
H	4.77286800	0.28327600	2.97324200
H	6.33170200	1.74419300	1.69000300
C	2.03848300	-0.38965700	-0.74374100
C	0.90063400	-0.72794300	-0.01028600
H	0.83449100	-0.49462000	1.05916200
H	1.19849800	-1.87338000	-0.22008700
C	1.99453200	-0.55504700	-2.23542300
H	1.49951300	-1.51144500	-2.46345600
H	3.01159800	-0.59720000	-2.64666600
C	-5.06404700	3.00851000	0.08962400
H	-6.11813600	2.69601900	0.13515700
H	-4.88279400	3.70995300	0.91385000
H	-4.92256500	3.54587300	-0.85894300
C	1.17263400	0.56722600	-2.88319900
H	0.14809900	0.55294900	-2.48552100
H	1.61402000	1.55368900	-2.68138500
H	1.12049600	0.42737400	-3.97129600

TS14

S	1.26317700	-1.19124000	1.41057800
N	-0.17955500	-0.52610900	0.94608100
O	1.34214000	-2.58415600	0.96515200
O	1.46406100	-0.85221900	2.80442900
C	2.43746600	-0.25967000	0.44884600
C	2.79080100	1.02094200	0.87602500
H	2.37239100	1.41046000	1.80544600
C	3.68341100	1.76099800	0.11155500
H	3.96994000	2.76358000	0.43730200
C	4.23318200	1.23892100	-1.06990700
C	3.86979800	-0.05211400	-1.46410600
H	4.30160200	-0.47671800	-2.37273200
C	2.97399600	-0.80985600	-0.70958900
H	2.70416800	-1.82747000	-0.99573800
C	-5.11209600	1.33604300	0.01551600
C	-4.33563800	0.24294400	-0.34689500
C	-2.94918500	0.21313800	-0.08592300
C	-2.37266300	1.33185900	0.54956500
C	-3.15653400	2.42763700	0.90243900
C	-4.52510300	2.43578100	0.64354800
H	-6.18267900	1.33018300	-0.19347400
H	-4.82003200	-0.59923500	-0.84050200
H	-1.30818200	1.32859200	0.77110900
H	-2.68770900	3.28297900	1.39083700
H	-5.13469600	3.29438300	0.92901000
C	-2.14982700	-0.94675000	-0.51013000
C	-0.85181400	-1.22920200	-0.07041200
H	-0.34206300	-2.09162200	-0.52146000
H	-1.33800500	-1.50747700	0.99506500
C	-2.72969500	-1.92574600	-1.50263400
H	-3.71926000	-2.27060600	-1.16748700
H	-2.09158300	-2.82003700	-1.53334500
C	5.20385900	2.05836800	-1.87793100
H	6.10194800	2.29333600	-1.28786700
H	5.52047400	1.52586300	-2.78363300
H	4.75222800	3.01412100	-2.18132200
C	-2.83273900	-1.32583000	-2.90932000
H	-3.48231000	-0.43982000	-2.91972700
H	-1.84105700	-1.01887800	-3.27013300
H	-3.24205300	-2.06282000	-3.61369900

Spectra

Supplementary Figure 15. *4-Methyl-N-(2-phenylallyl)benzenesulfonamide (**10a**)*

^{1}H NMR (400 MHz, 293 K, $CDCl_3$)

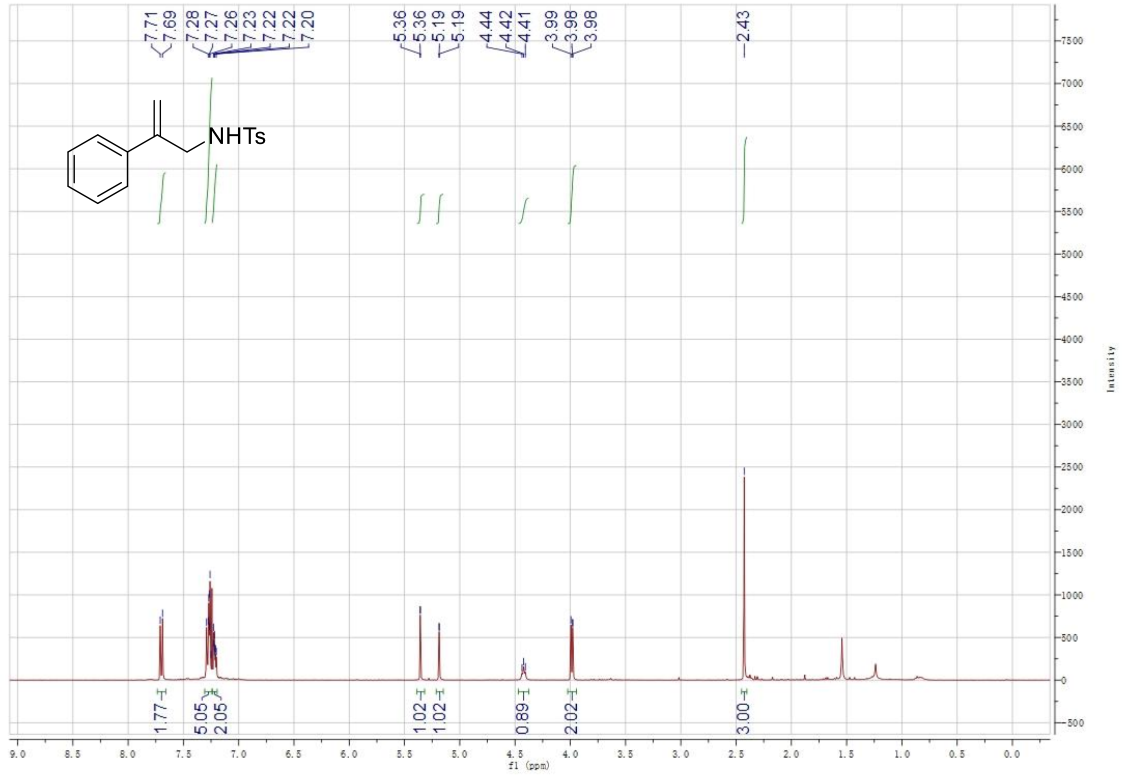

^{13}C NMR (101 MHz, 293 K, $CDCl_3$)

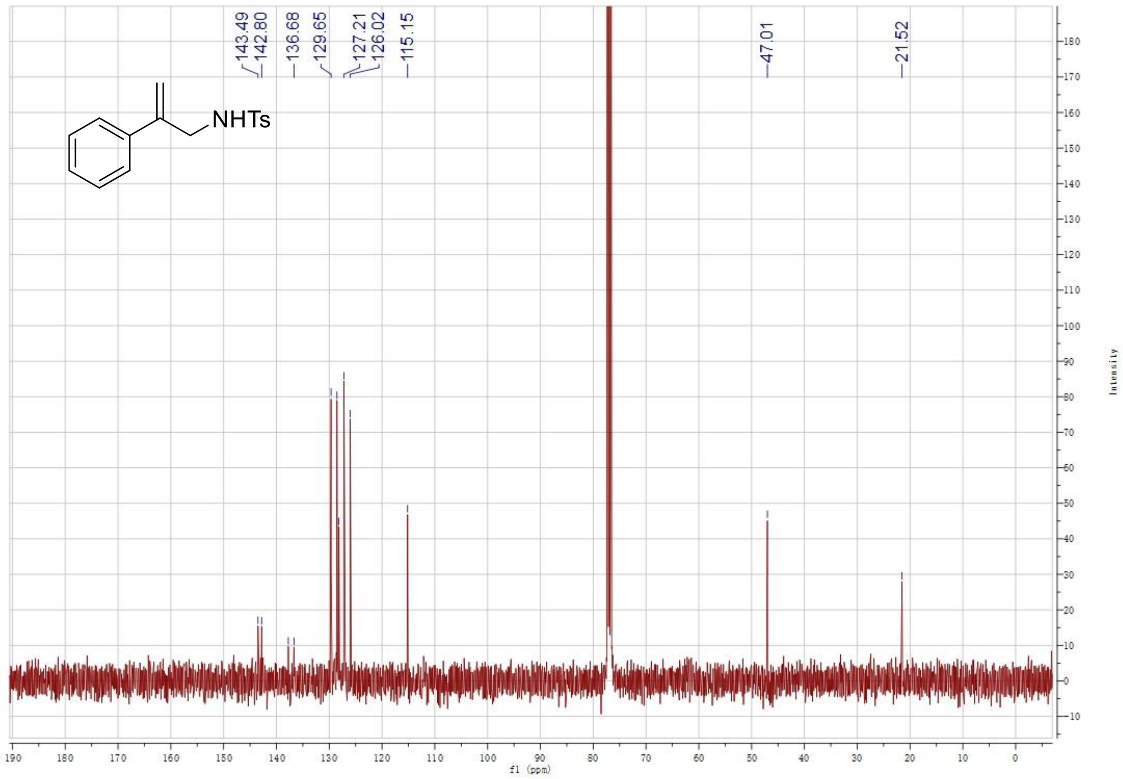

Supplementary Figure 16. *4-Methyl-N-(2-phenylallyl-3,3-d2)benzenesulfonamide* (***10a-d$_2$***)

^{1}H NMR (400 MHz, 293 K, $CDCl_3$)

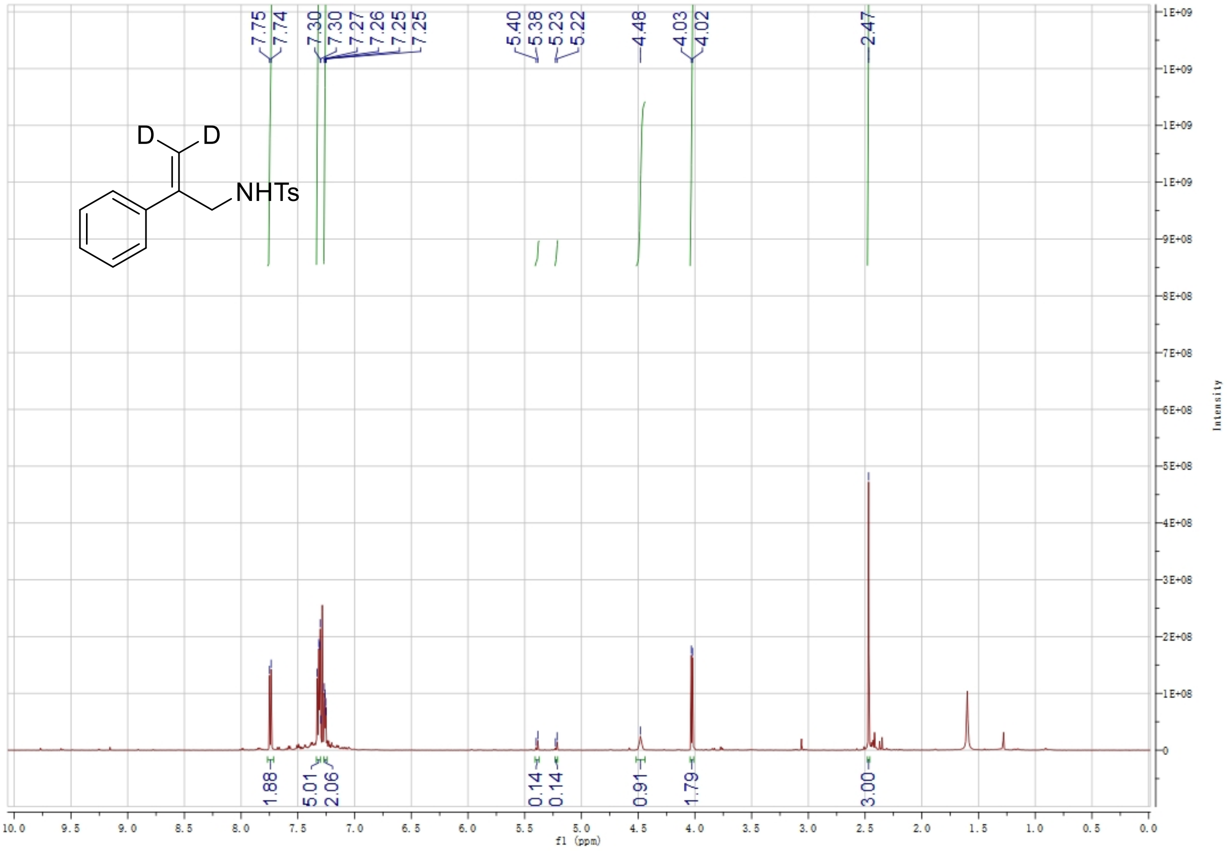

^{13}C NMR (101 MHz, 293 K, $CDCl_3$)

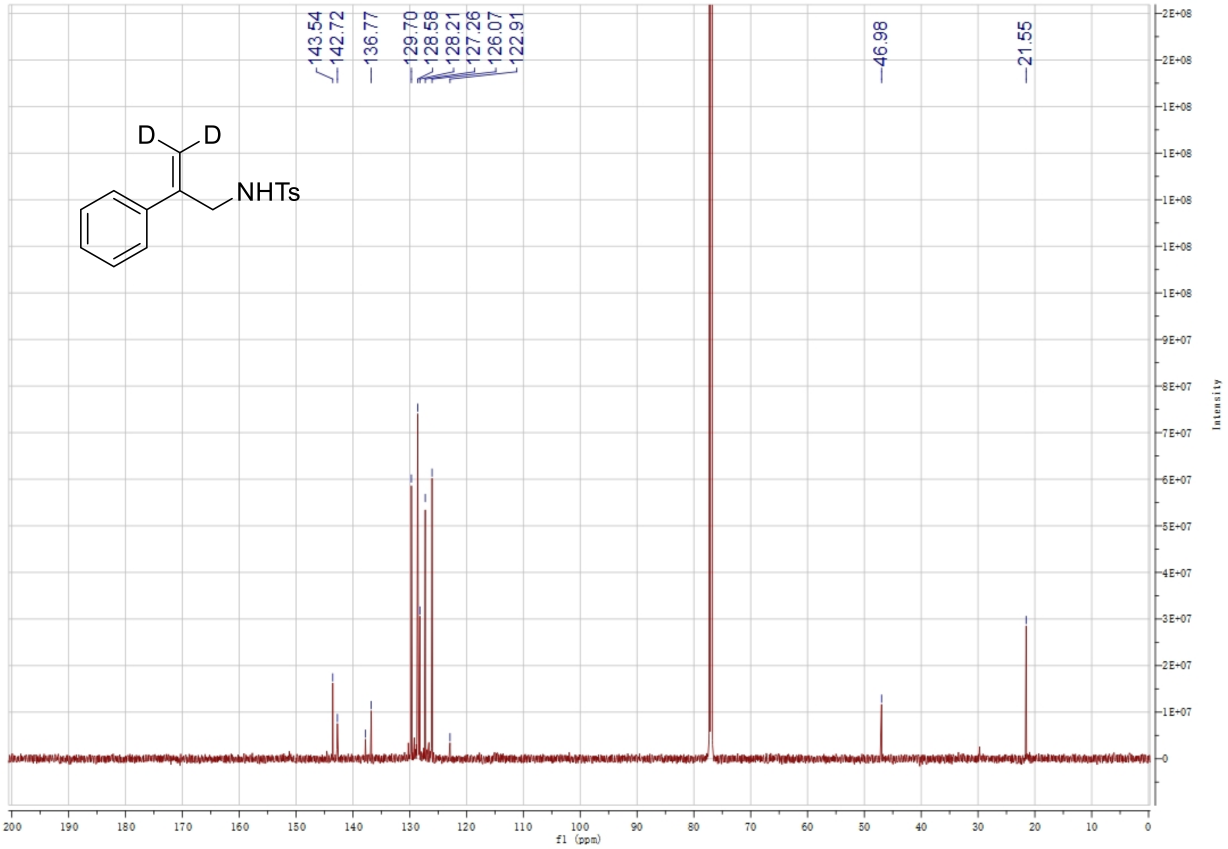

Supplementary Figure 17. *4-Methyl-N-(2-(p-tolyl)allyl)benzenesulfonamide* (***10b***)

^{1}H NMR (600 MHz, 293 K, $CDCl_3$)

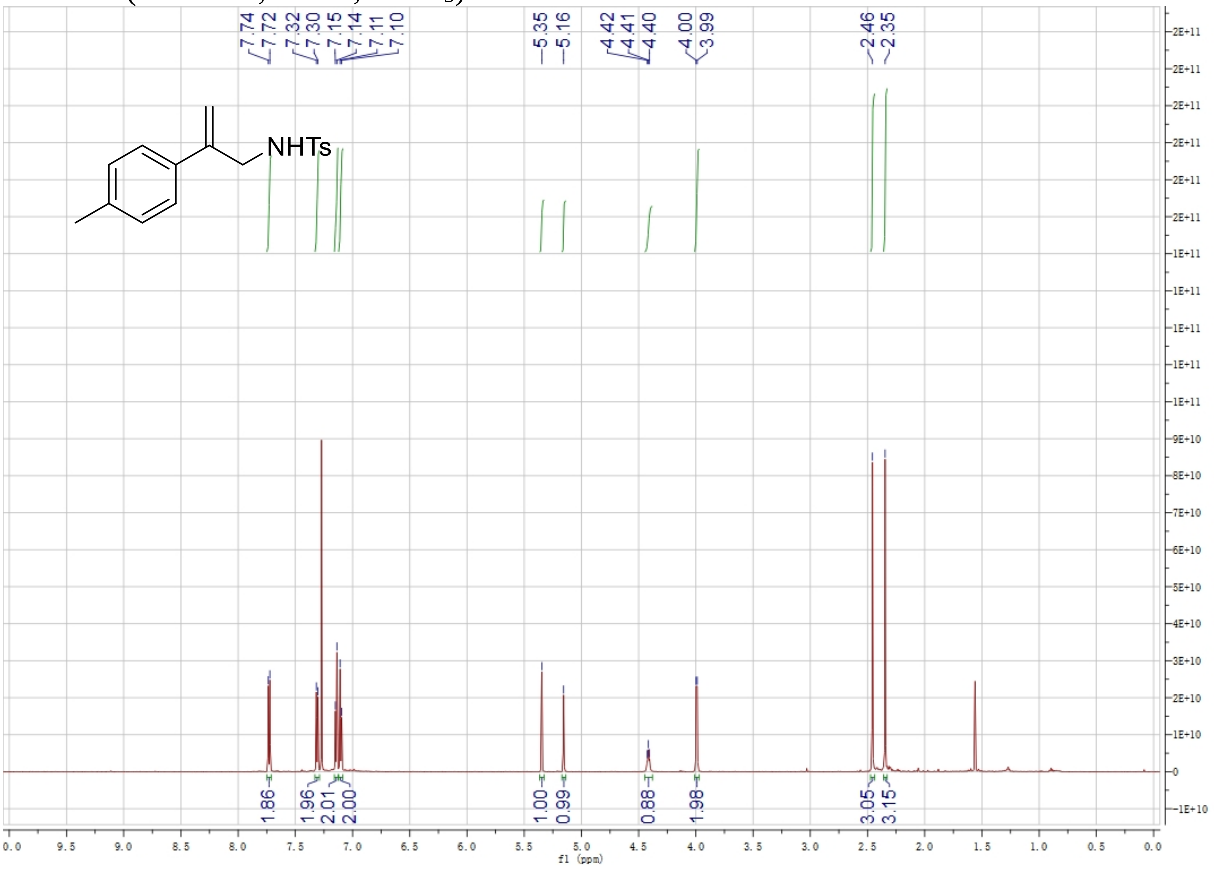

^{13}C NMR (151 MHz, 293 K, $CDCl_3$)

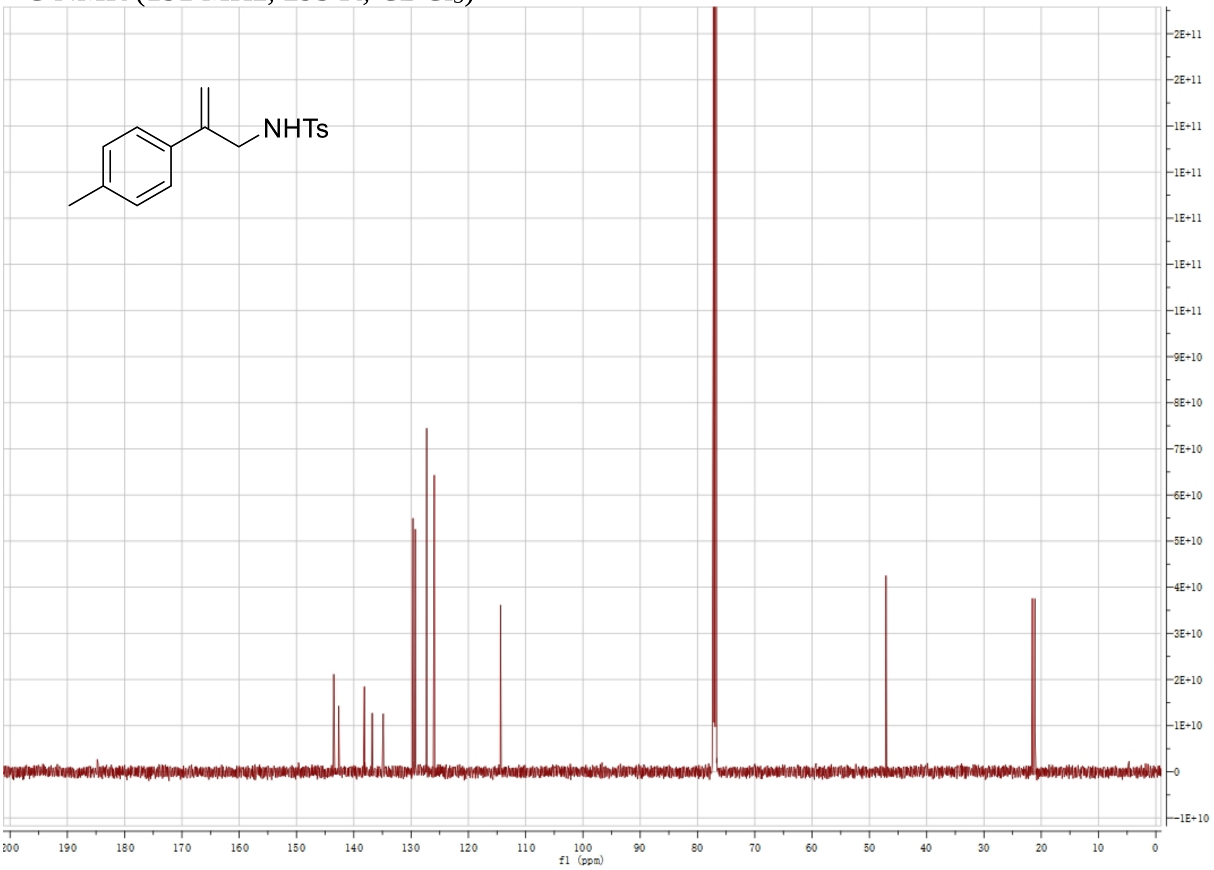

Supplementary Figure 18. *N-(2-(4-Butylphenyl)allyl)-4-methylbenzenesulfonamide (**10c**)*

^{1}H NMR (600 MHz, 293 K, $CDCl_3$)

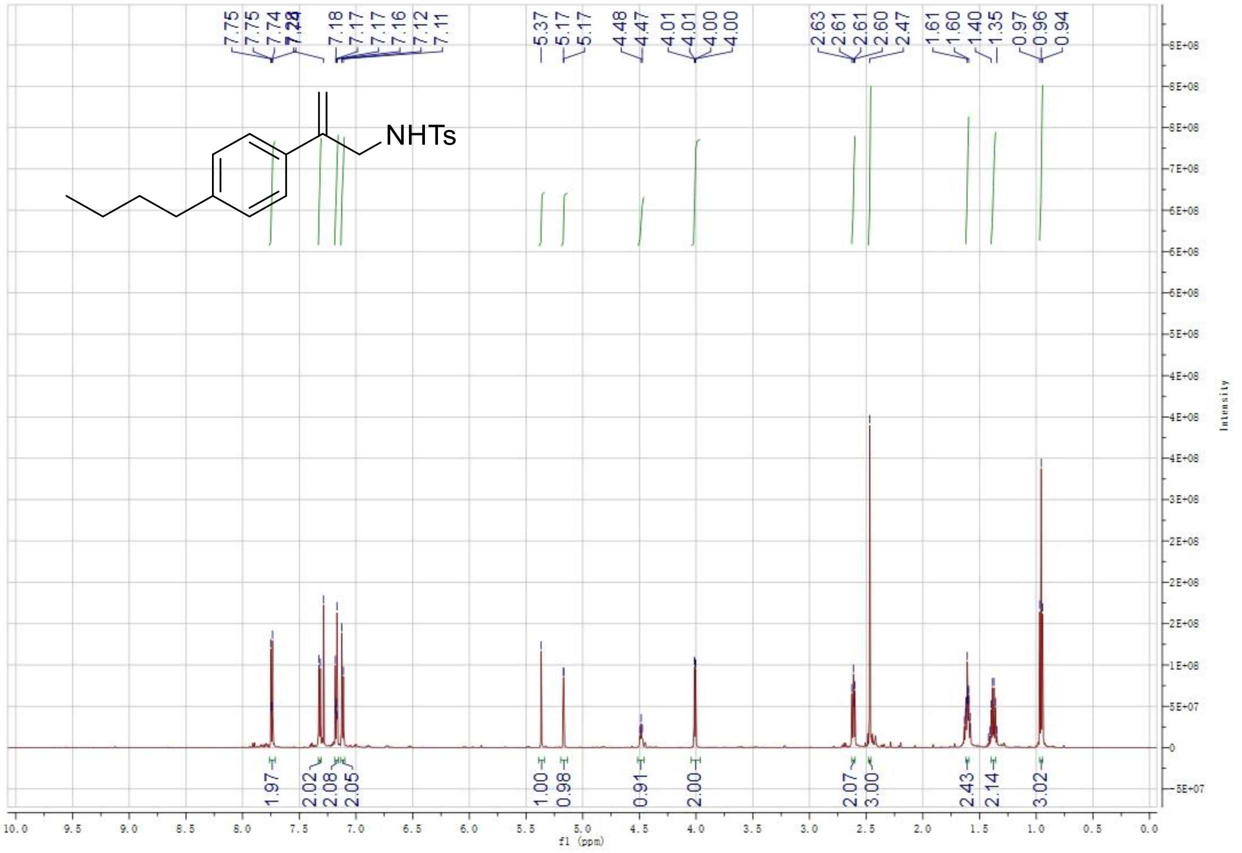

^{13}C NMR (151 MHz, 293 K, $CDCl_3$)

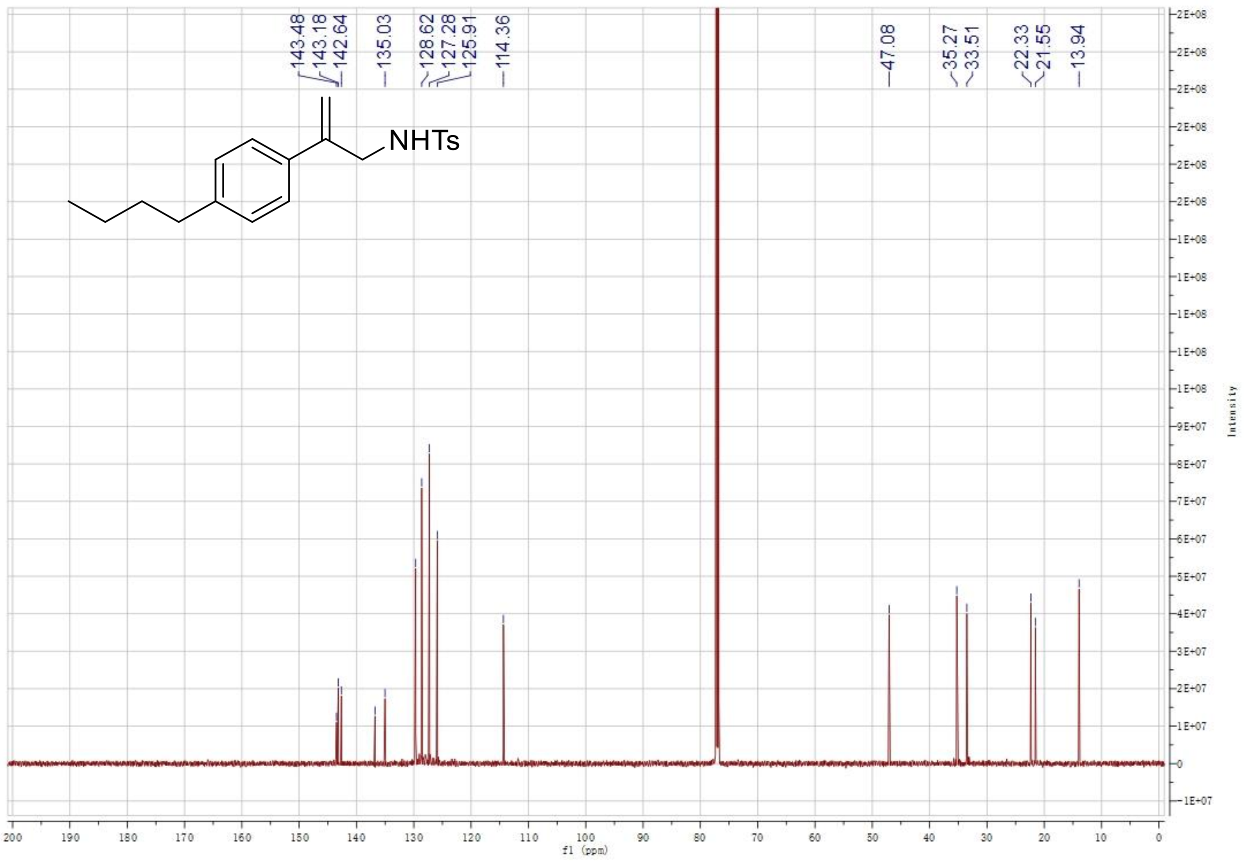

Supplementary Figure 19. *N-(2-(4-Fluorophenyl)allyl)-4-methylbenzenesulfonamide* (**10d**)

^{1}H NMR (600 MHz, 293 K, $CDCl_3$)

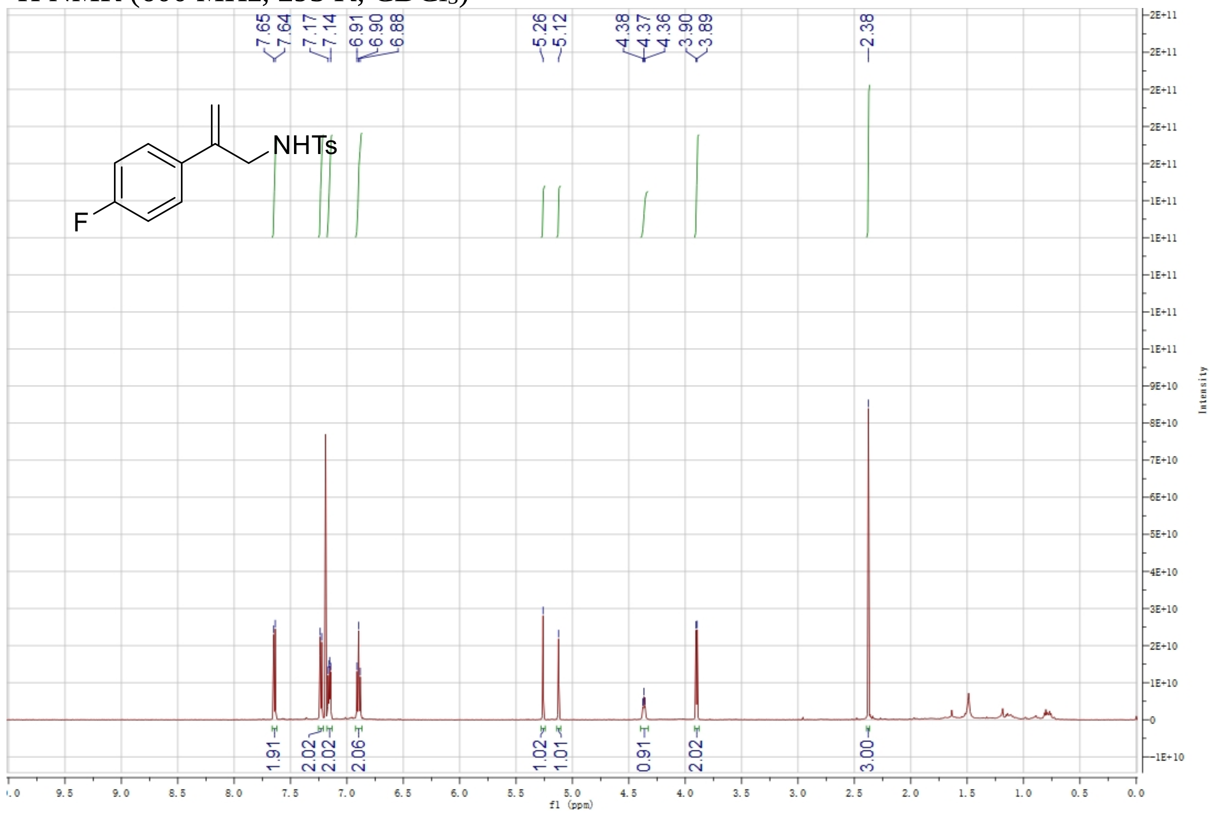

^{13}C NMR (151 MHz, 293 K, $CDCl_3$)

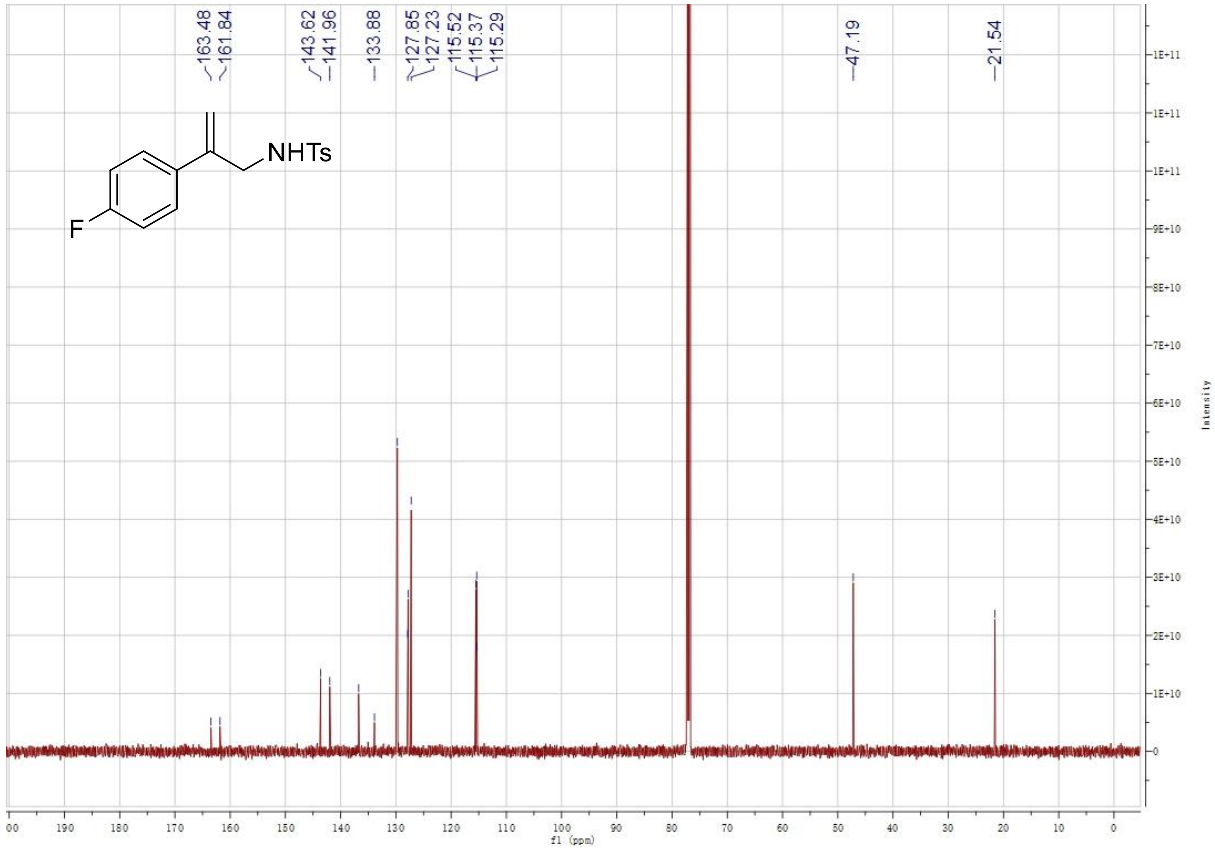

^{19}FNMR (565 MHz, 293 K, $CDCl_3$)

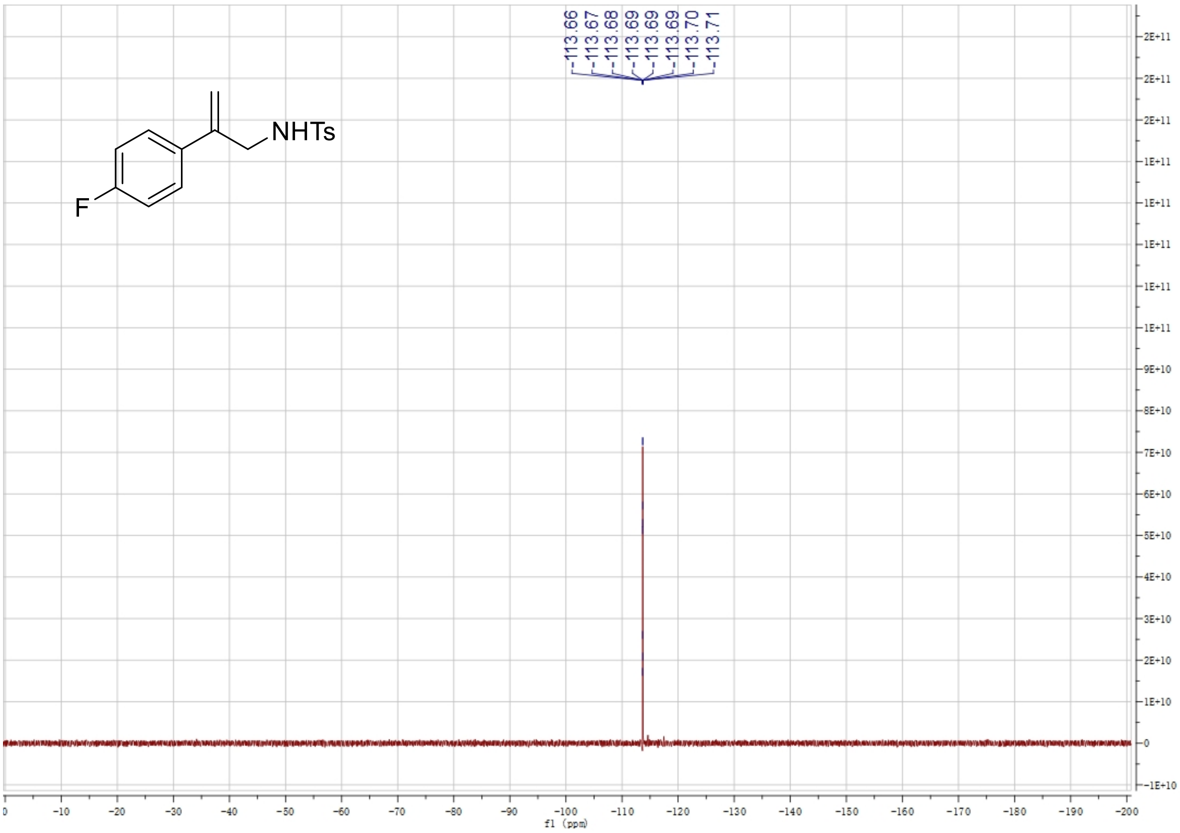

Supplementary Figure 20. *N-(2-(4-Chlorophenyl)allyl)-4-methylbenzenesulfonamide (**10e**)*

^{1}H NMR (600 MHz, 293 K, $CDCl_3$)

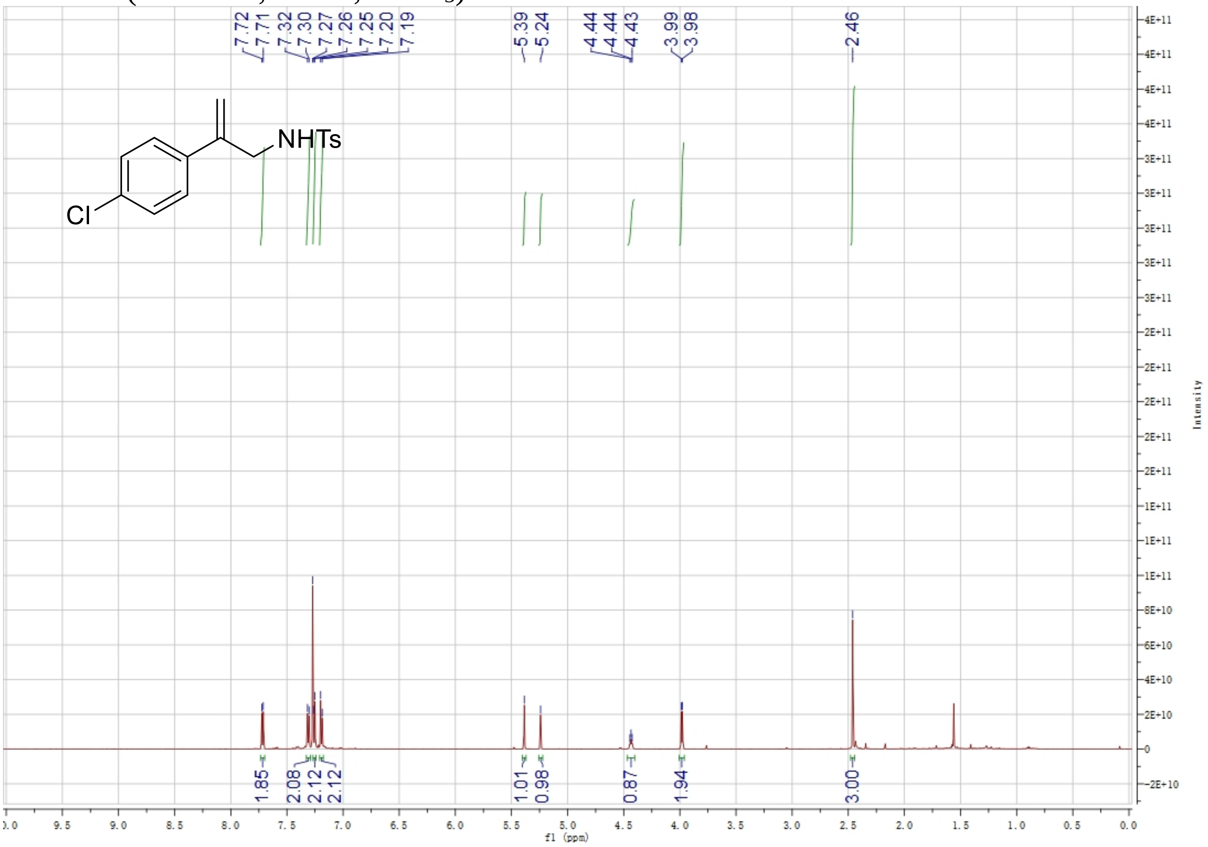

^{13}C NMR (151 MHz, 293 K, $CDCl_3$)

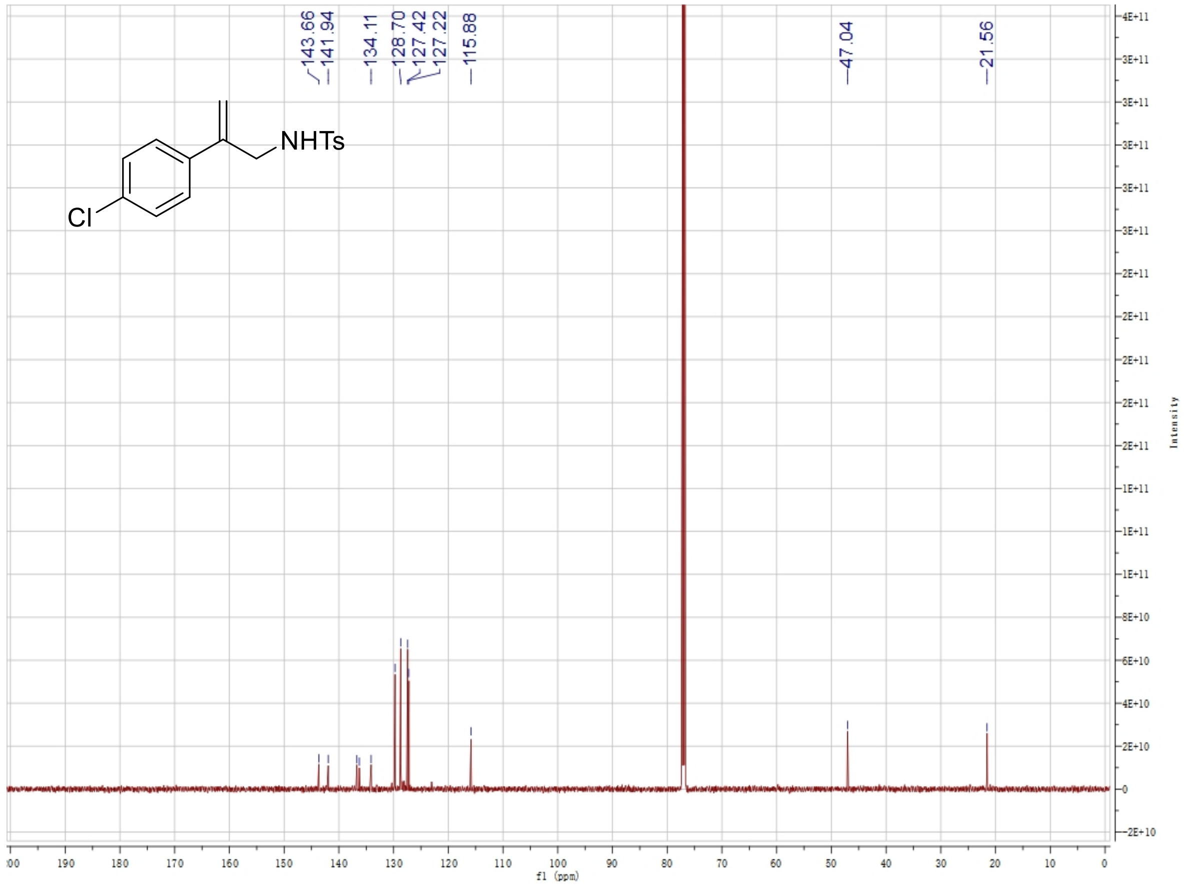

Supplementary Figure 21. *N-(2-(4-Bromophenyl)allyl)-4-methylbenzenesulfonamide (**10f**)*

^{1}H NMR (400 MHz, 293 K, $CDCl_3$)

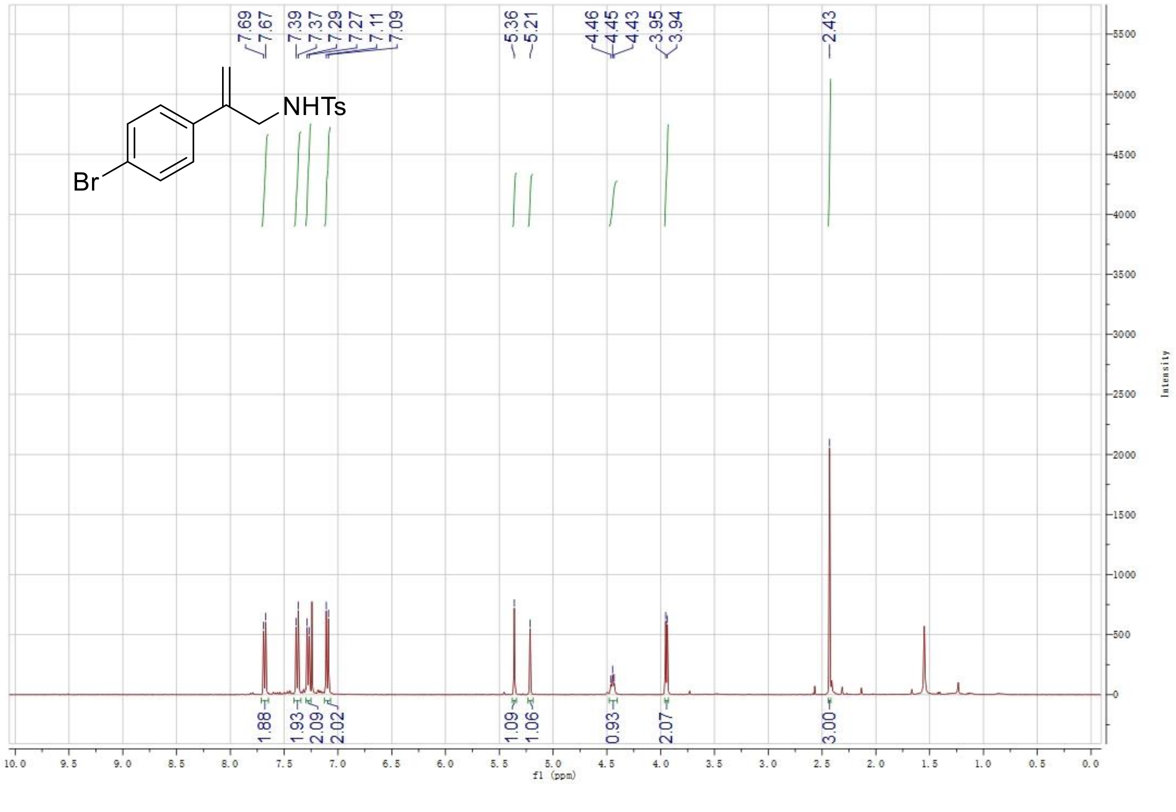

^{13}C NMR (101 MHz, 293 K, $CDCl_3$)

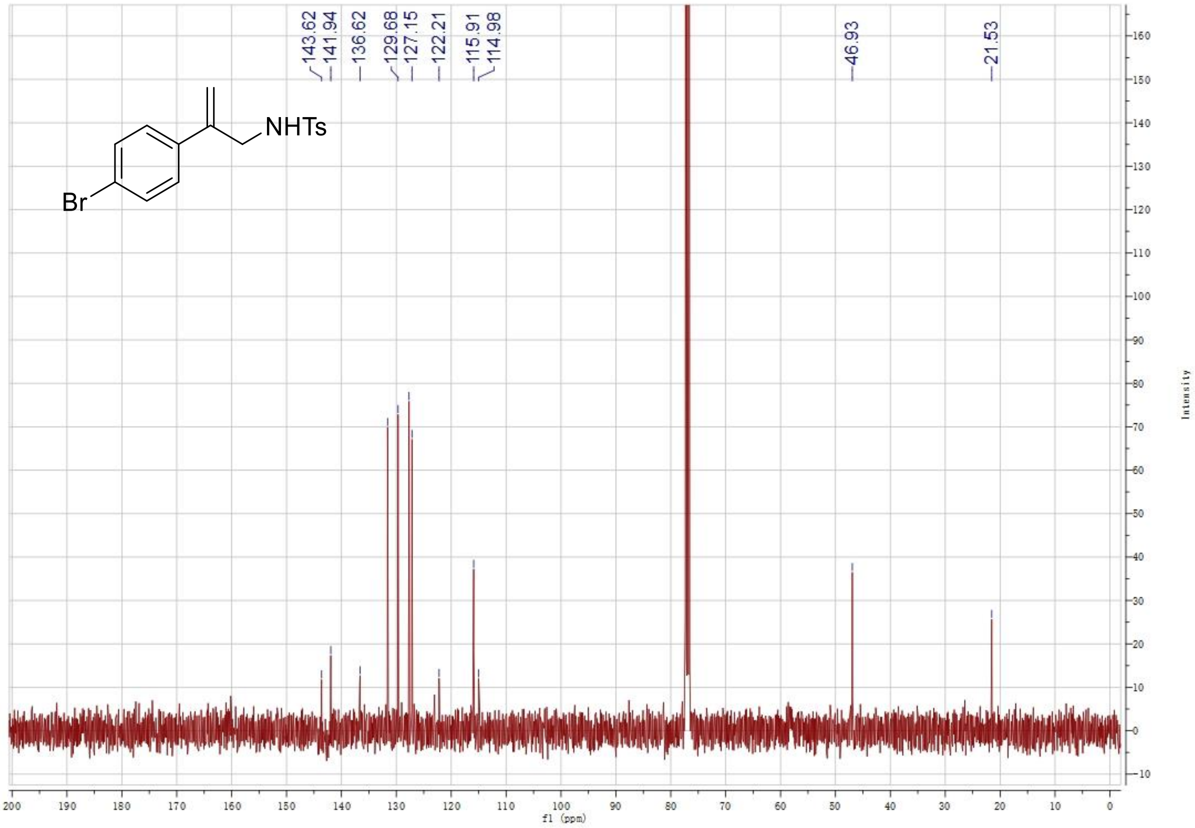

Supplementary Figure 22. *N-(2-(4-Methoxyphenyl)allyl)-4-methylbenzenesulfonamide (**10g**)*

^{1}H NMR (600 MHz, 293 K, $CDCl_3$)

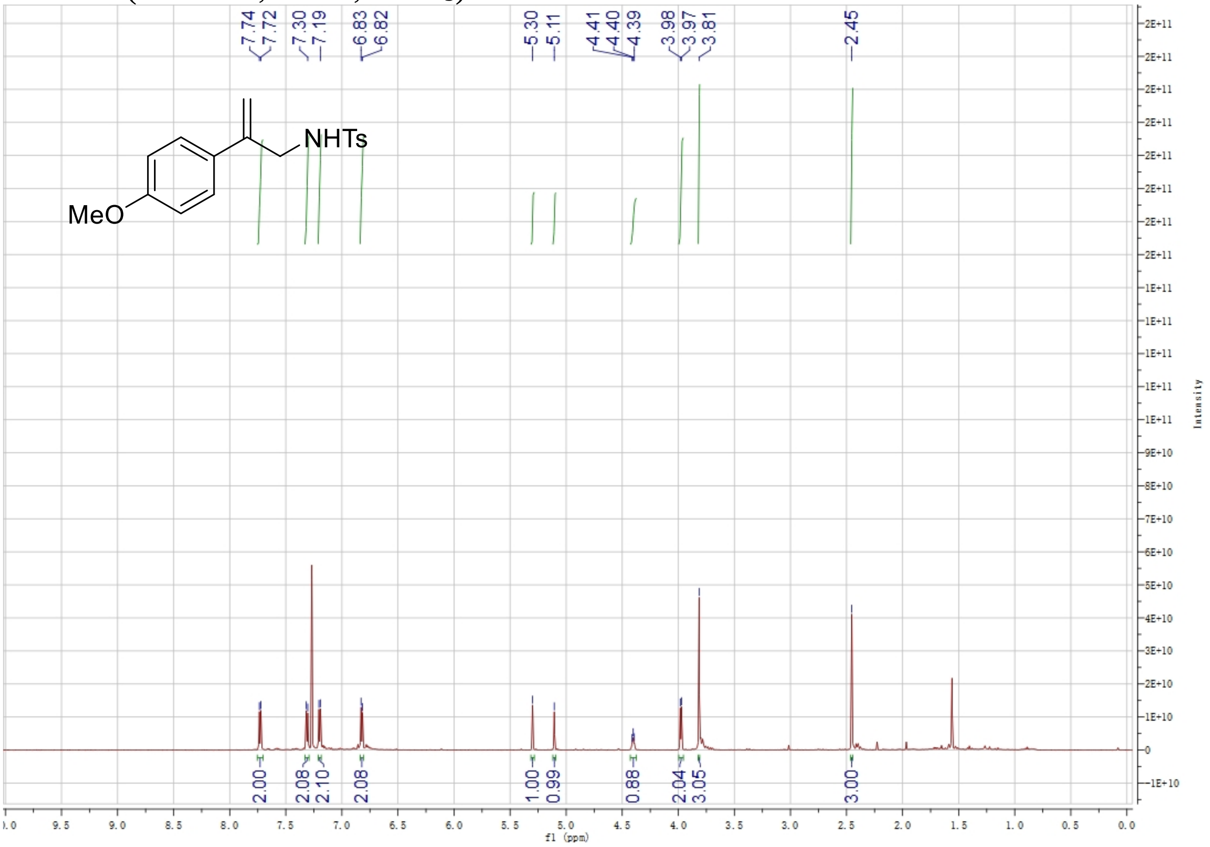

^{13}C NMR (151 MHz, 293 K, $CDCl_3$)

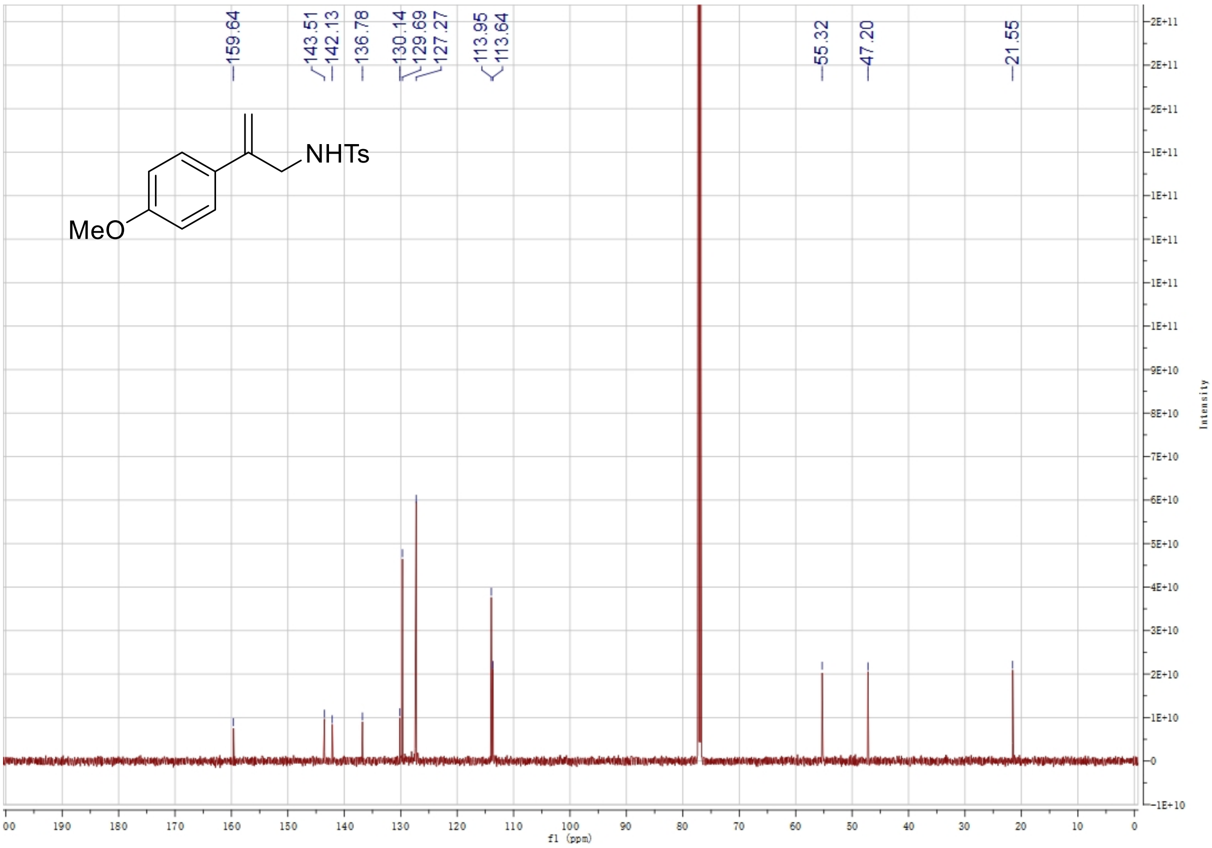

Supplementary Figure 23. *N-(2-(4-Cyanophenyl)allyl)-4-methylbenzenesulfonamide (**10h**)*

^{1}H NMR (600 MHz, 293 K, $CDCl_3$)

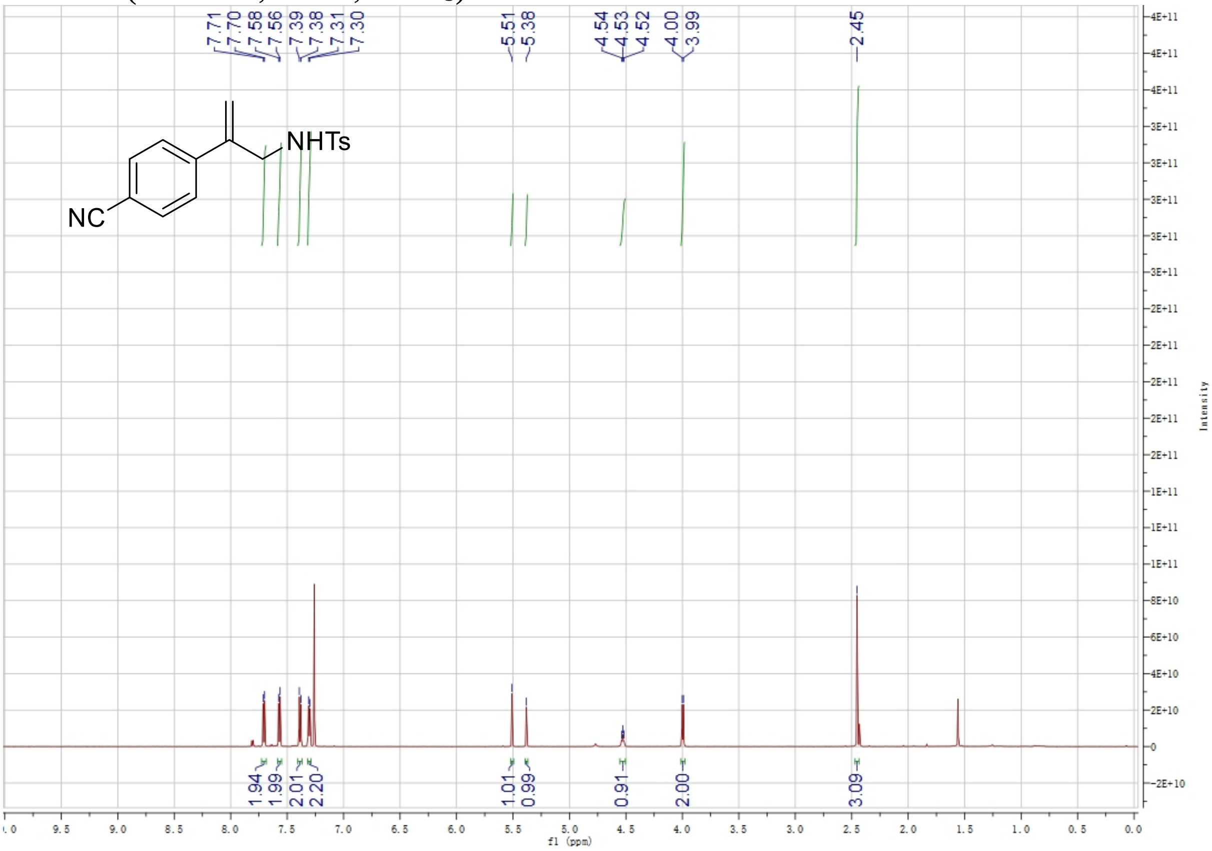

^{13}C NMR (151 MHz, 293 K, $CDCl_3$)

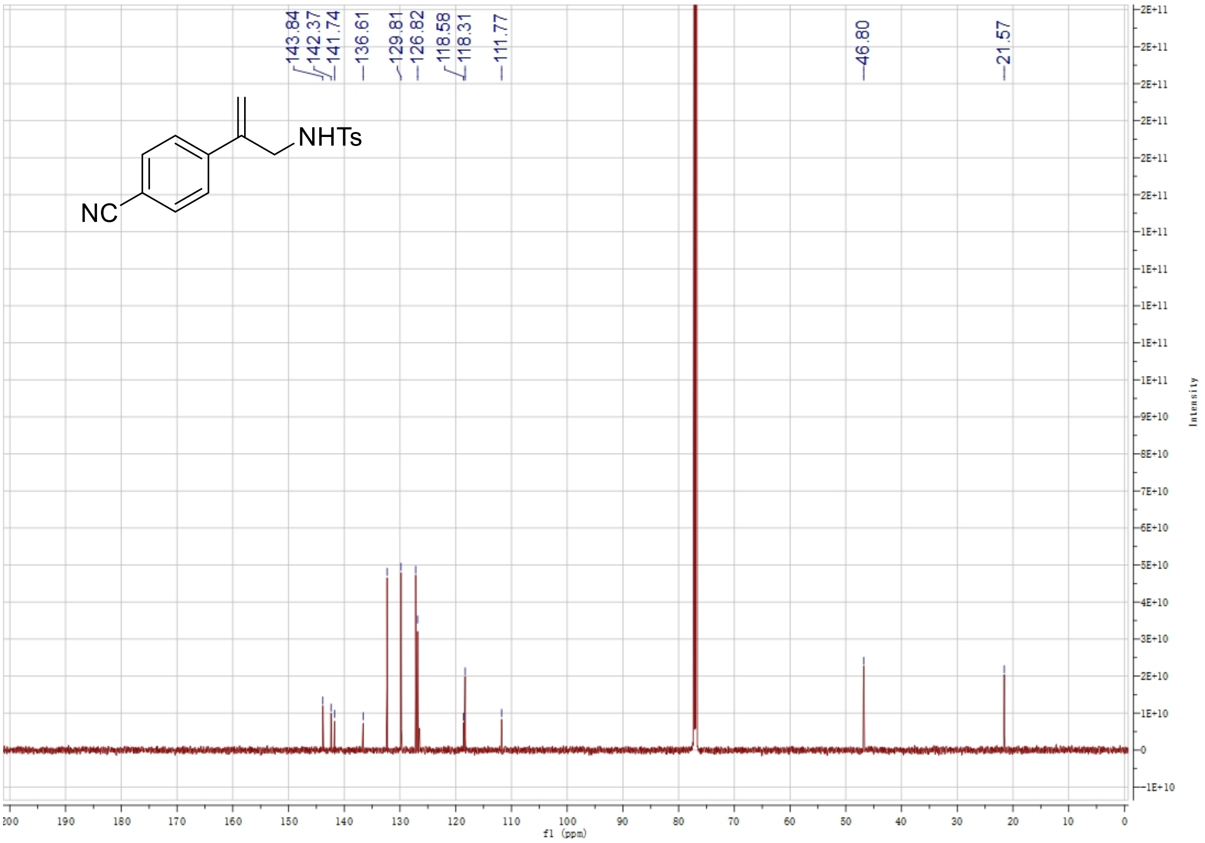

Supplementary Figure 24. *N-(2-(3-Fluorophenyl)allyl)-4-methylbenzenesulfonamide* (***10i***)

^{1}H NMR (600 MHz, 293 K, $CDCl_3$)

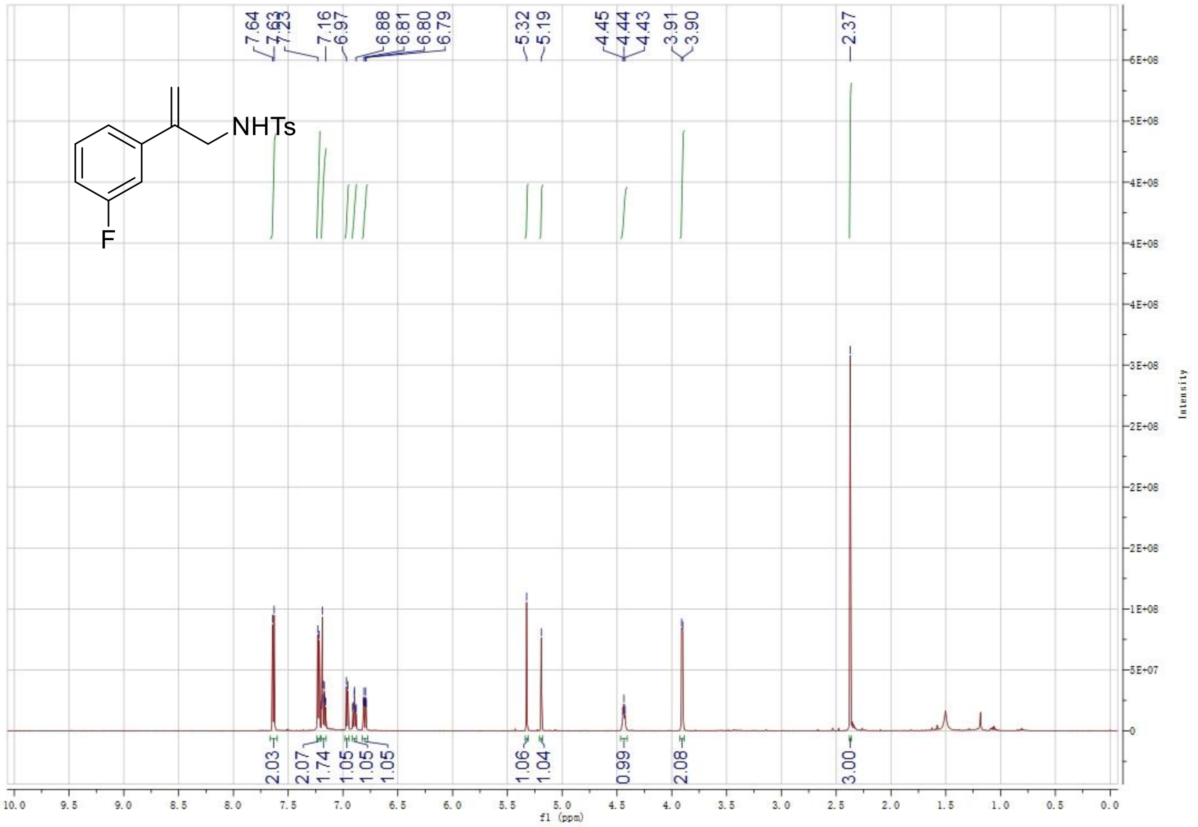

^{13}C NMR (151 MHz, 293 K, $CDCl_3$)

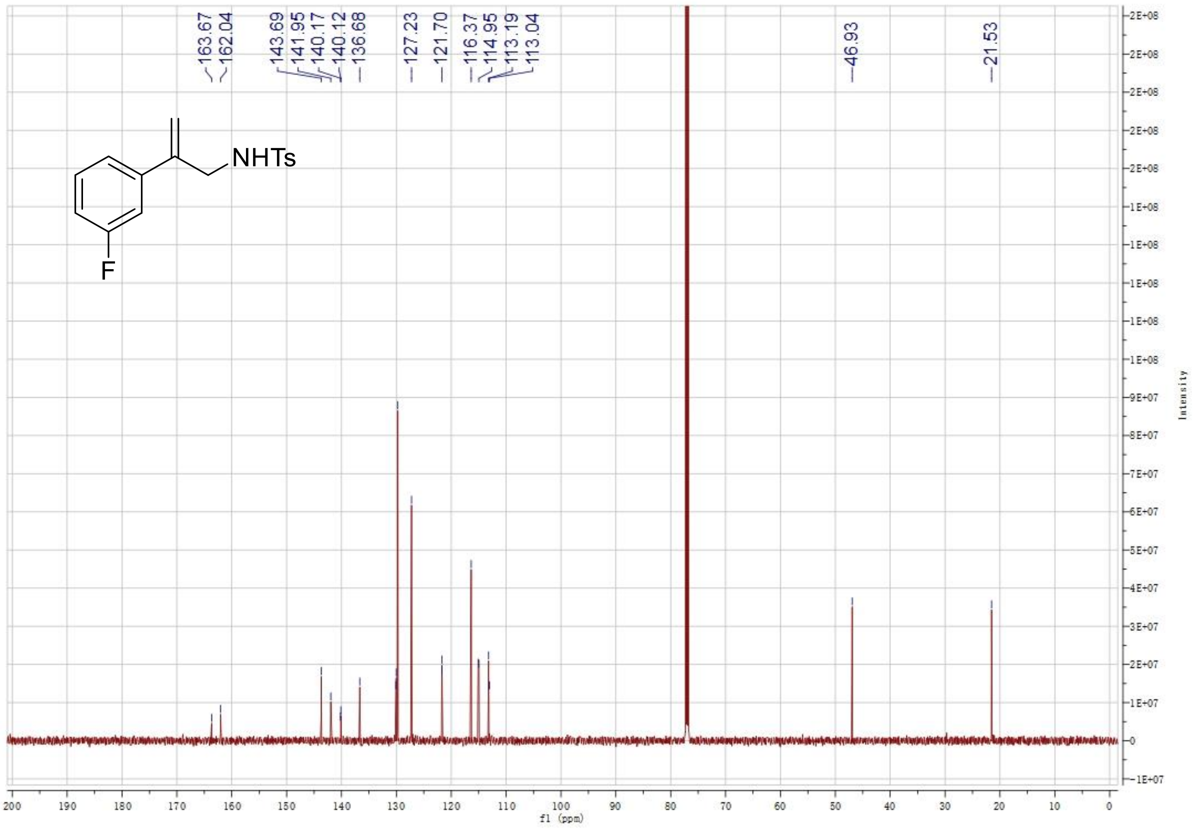

^{19}F NMR (565 MHz, 293 K, $CDCl_3$)

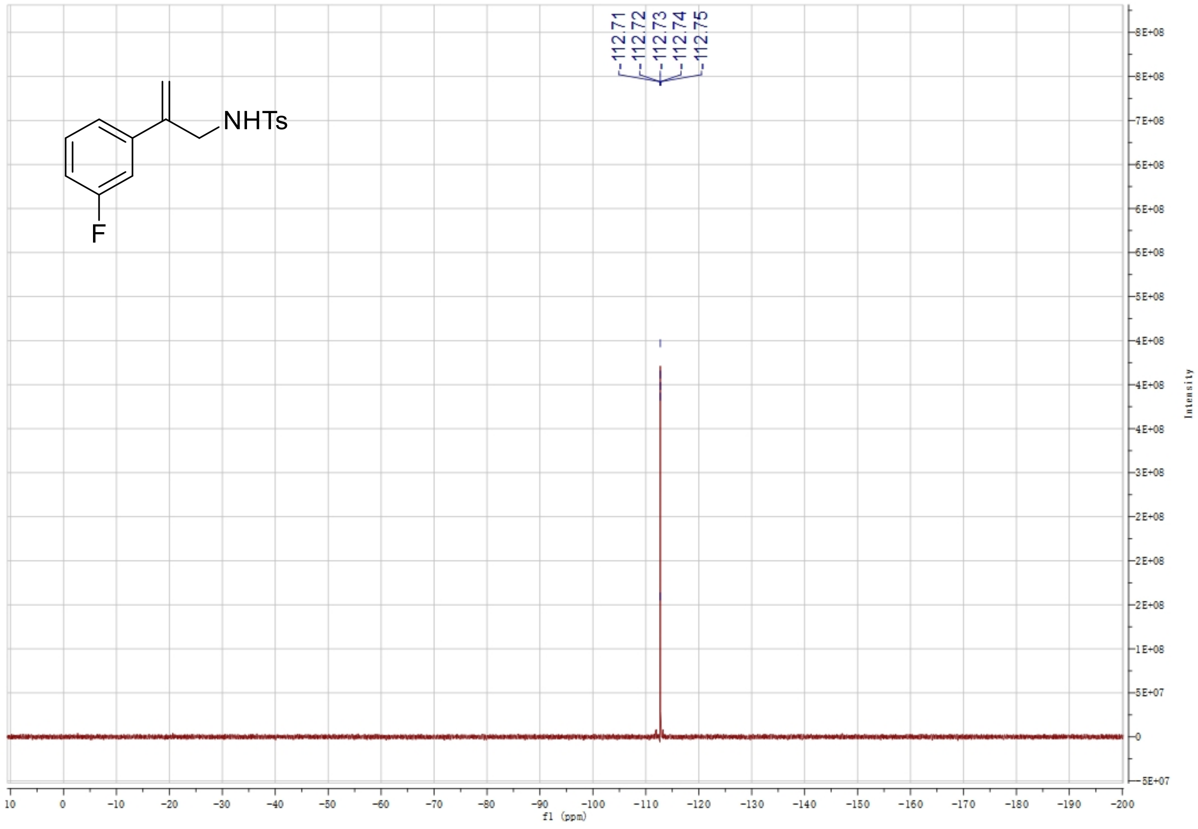

Supplementary Figure 25. *4-Methyl-N-(2-(m-tolyl)allyl)benzenesulfonamide* (***10j***)

^{1}H NMR (600 MHz, 293 K, $CDCl_3$)

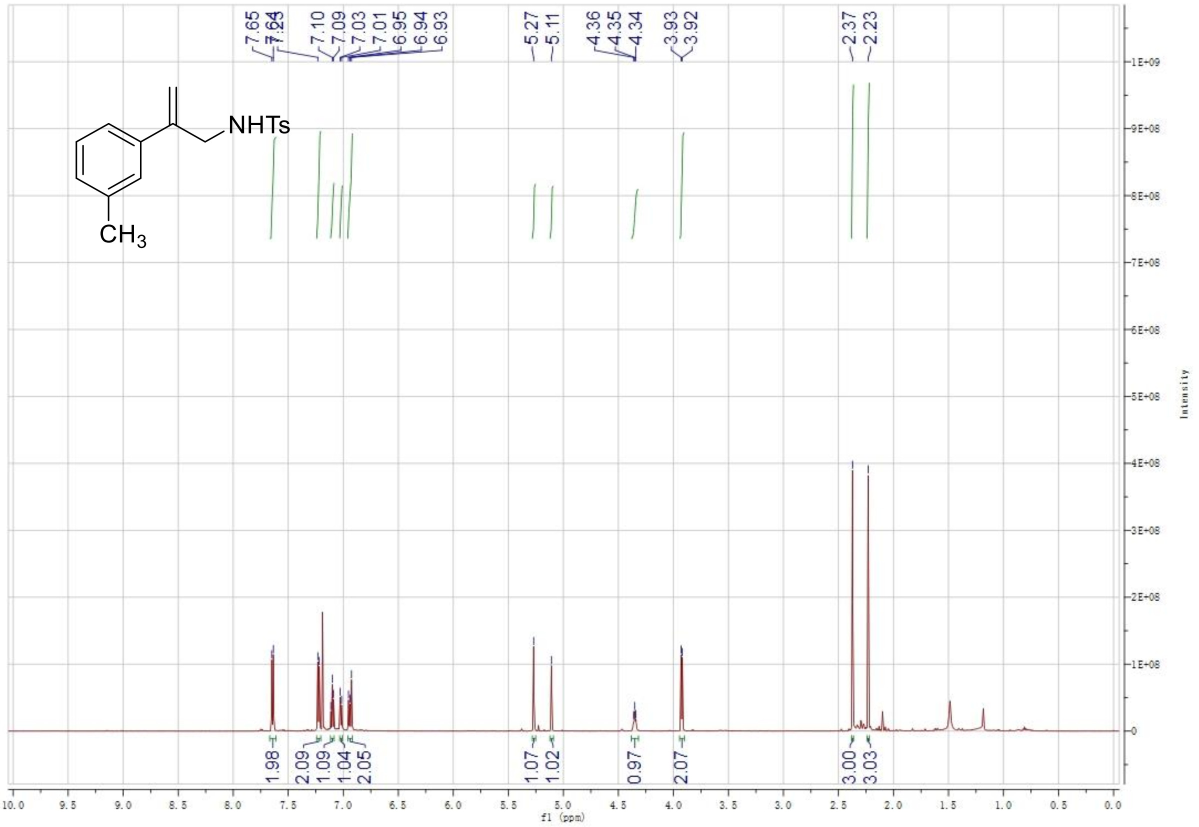

^{13}C NMR (151 MHz, 293 K, $CDCl_3$)

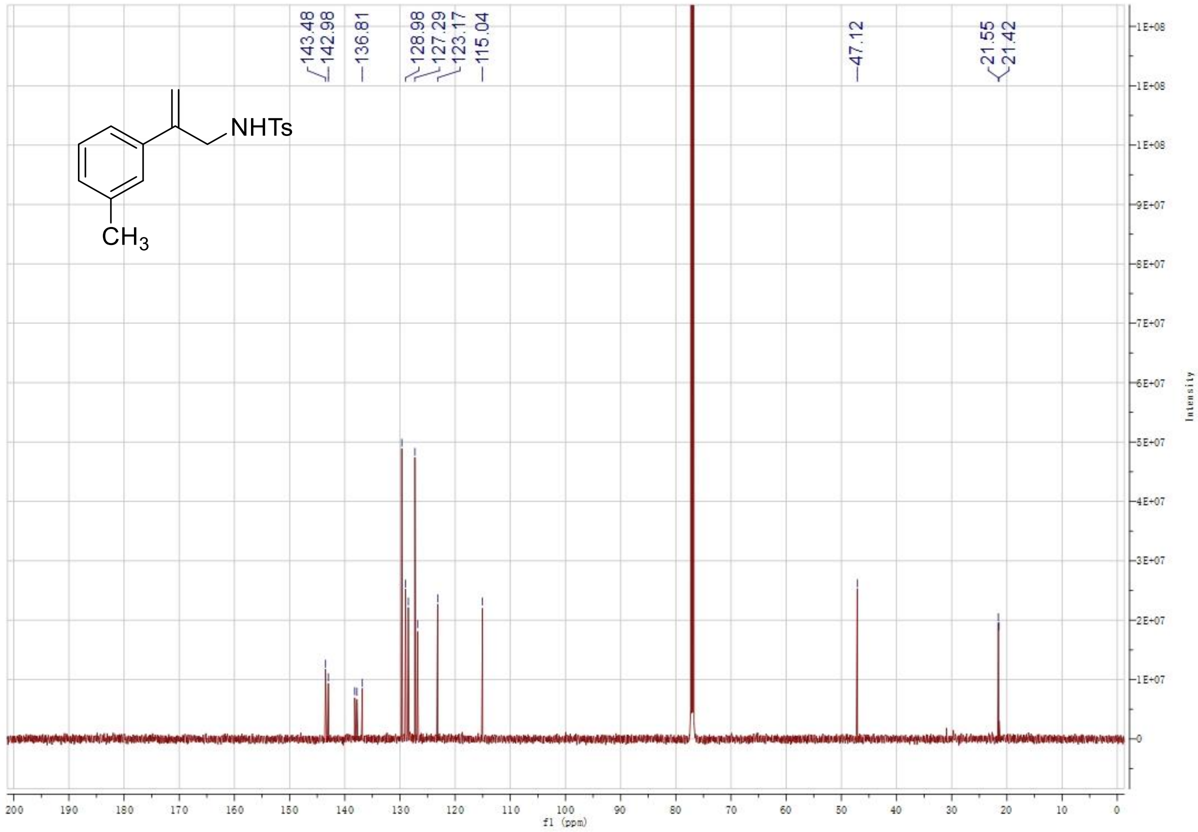

Supplementary Figure 26. *N-(2-(3-Methoxyphenyl)allyl)-4-methylbenzenesulfonamide* (***10k***)

^{1}H NMR (600 MHz, 293 K, $CDCl_3$)

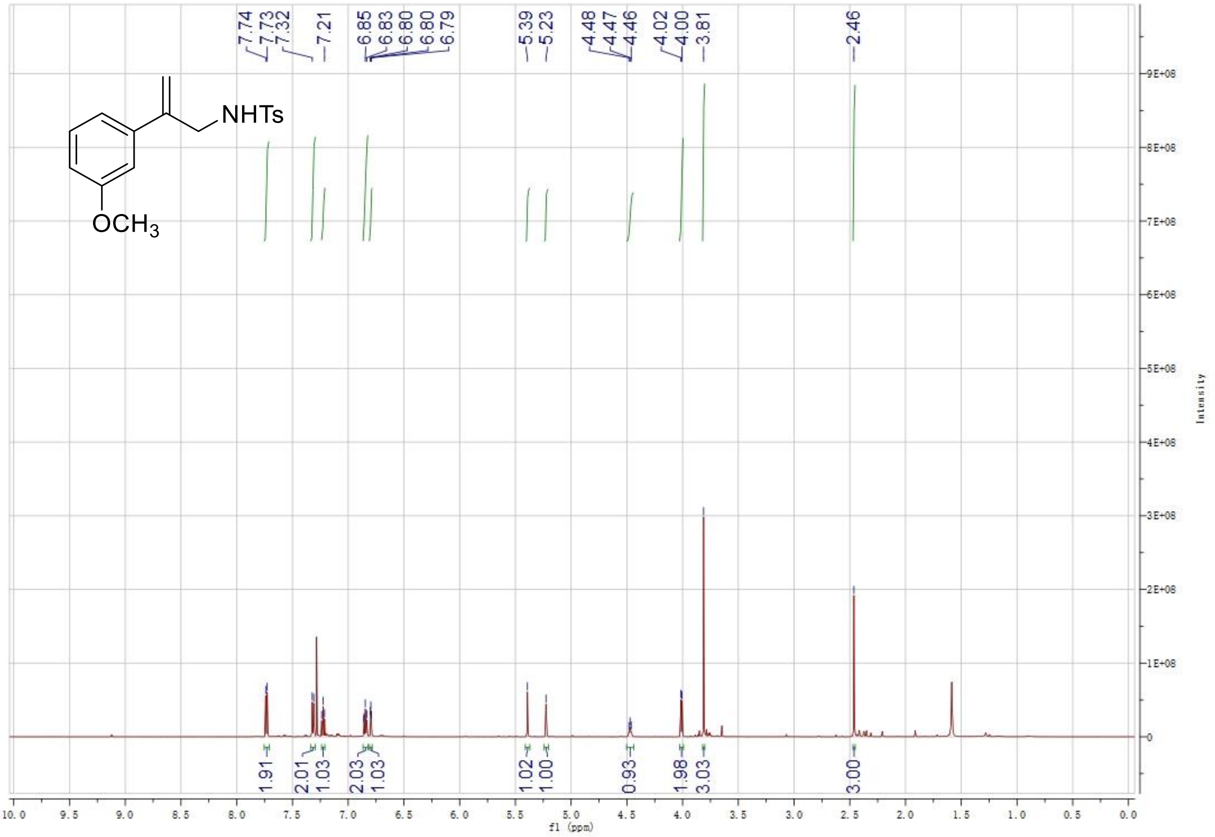

^{13}C NMR (151 MHz, 293 K, $CDCl_3$)

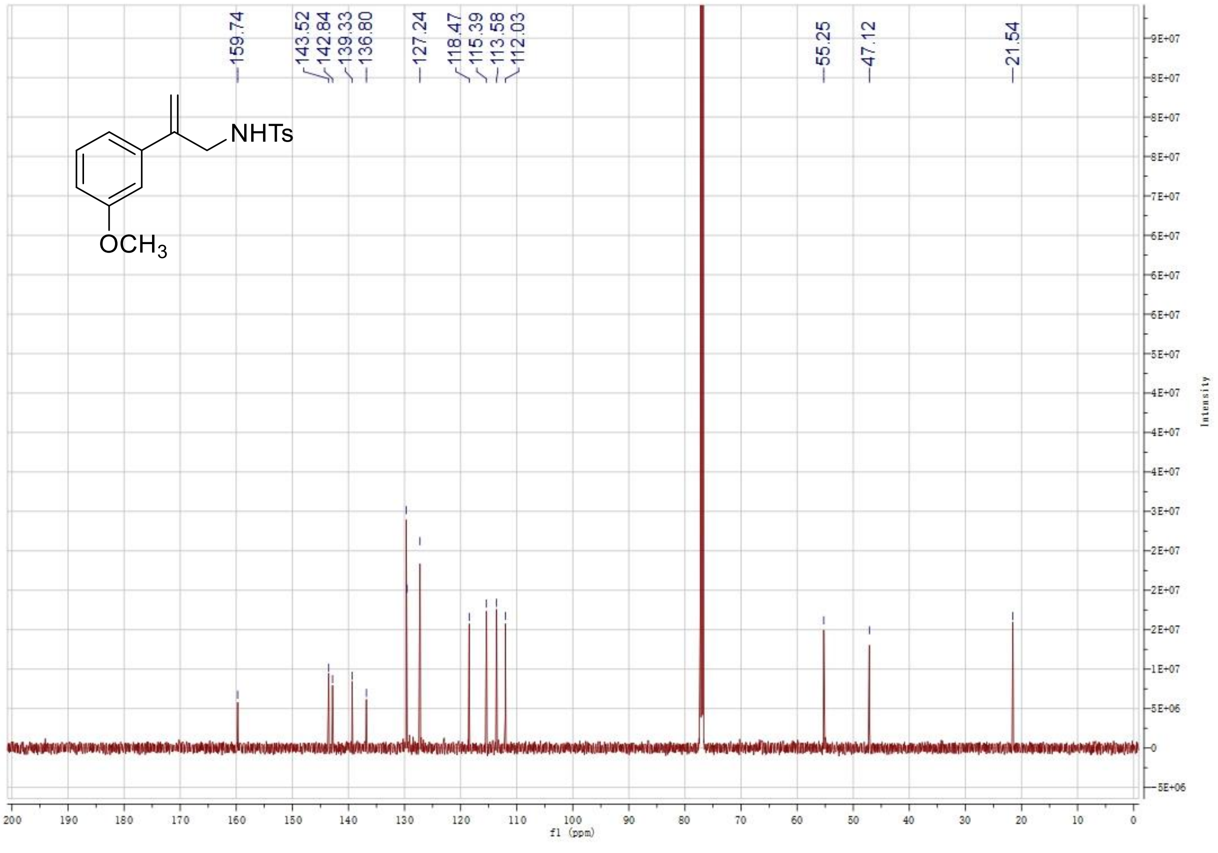

Supplementary Figure 27.
*4-Methyl-N-(2-(3-(trifluoromethyl)phenyl)allyl)benzenesulfonamide (**10l**)*
^{1}H NMR (600 MHz, 293 K, $CDCl_3$)

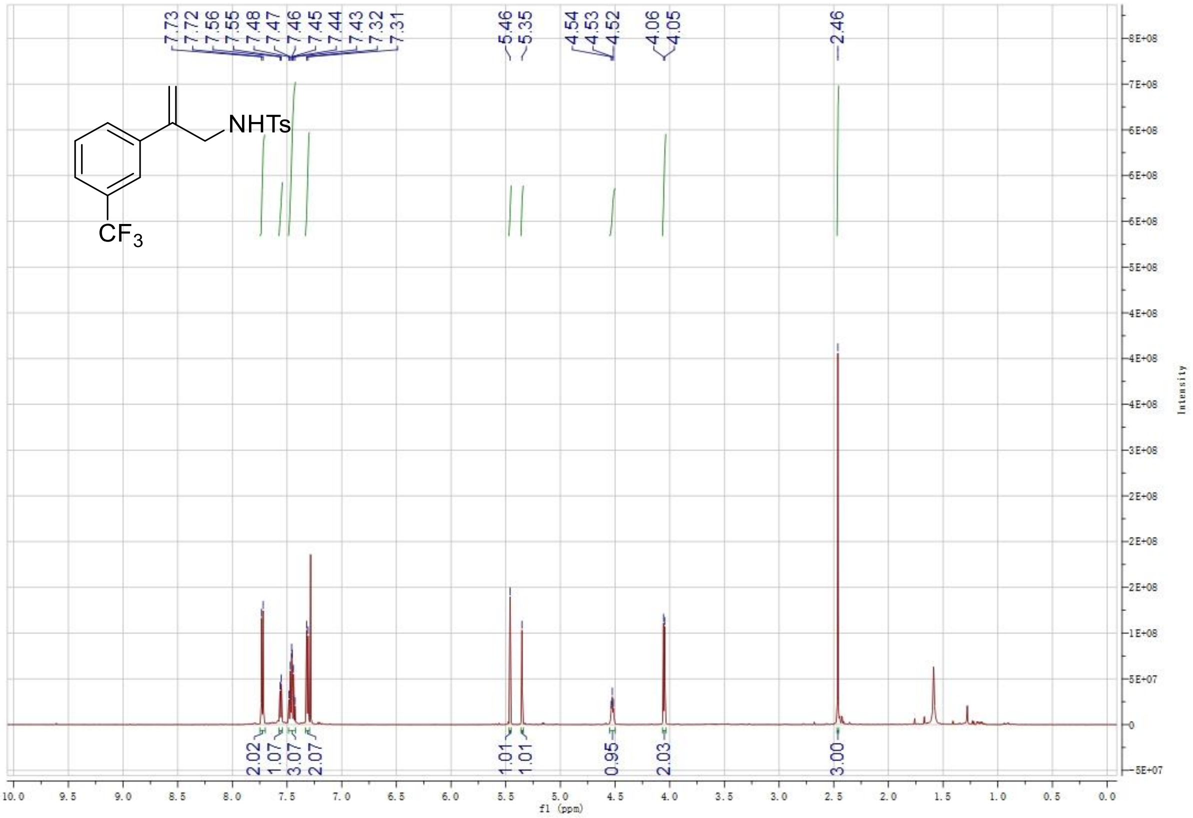

^{13}C NMR (151 MHz, 293 K, $CDCl_3$)

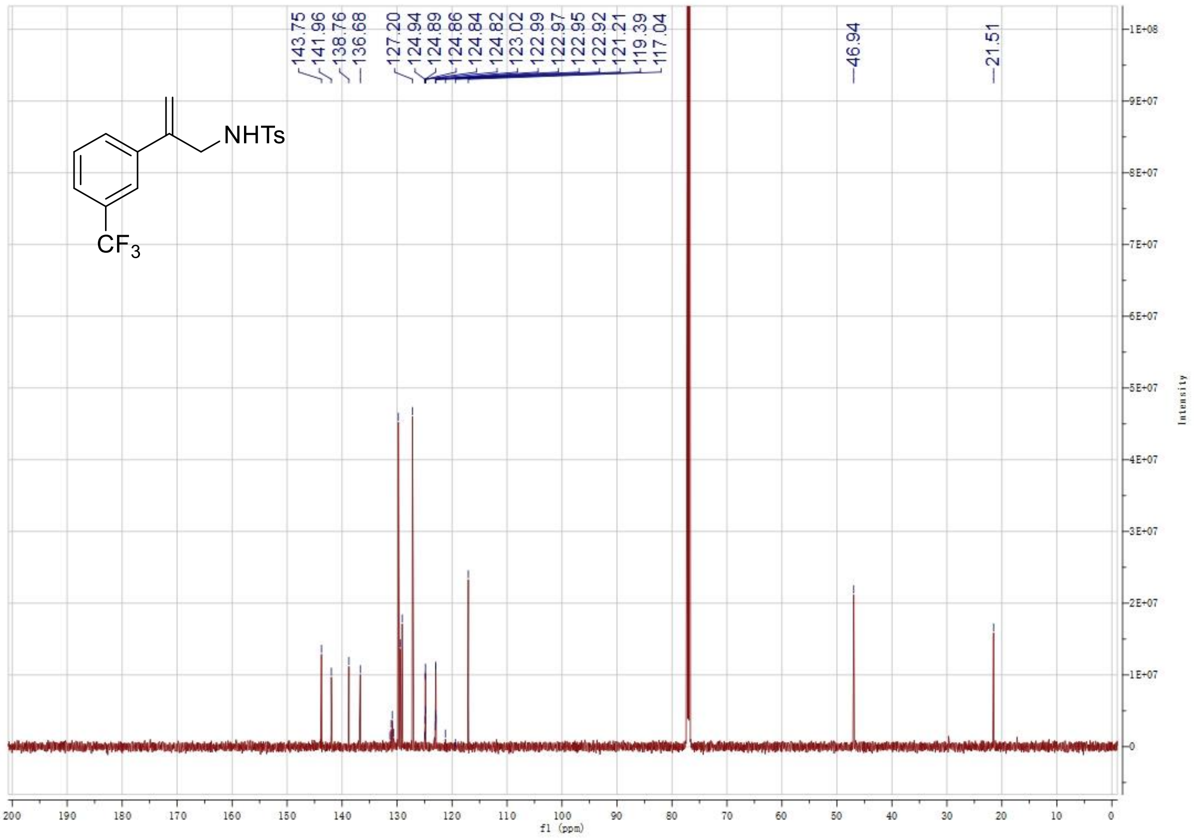

^{19}F NMR (565 MHz, 293 K, $CDCl_3$)

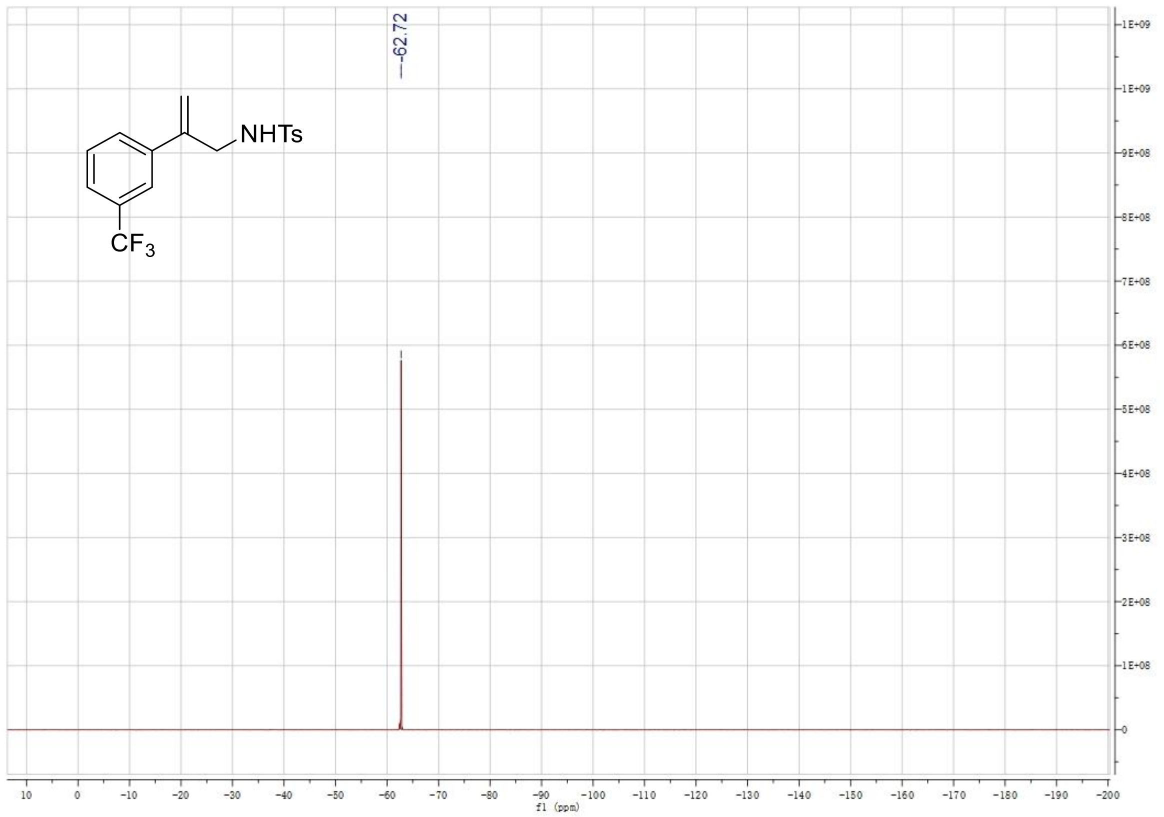

Supplementary Figure 28. *N-(2-(2-Bromophenyl)allyl)-4-methylbenzenesulfonamide (**10m**)*
^{1}H NMR (600 MHz, 293 K, $CDCl_3$)

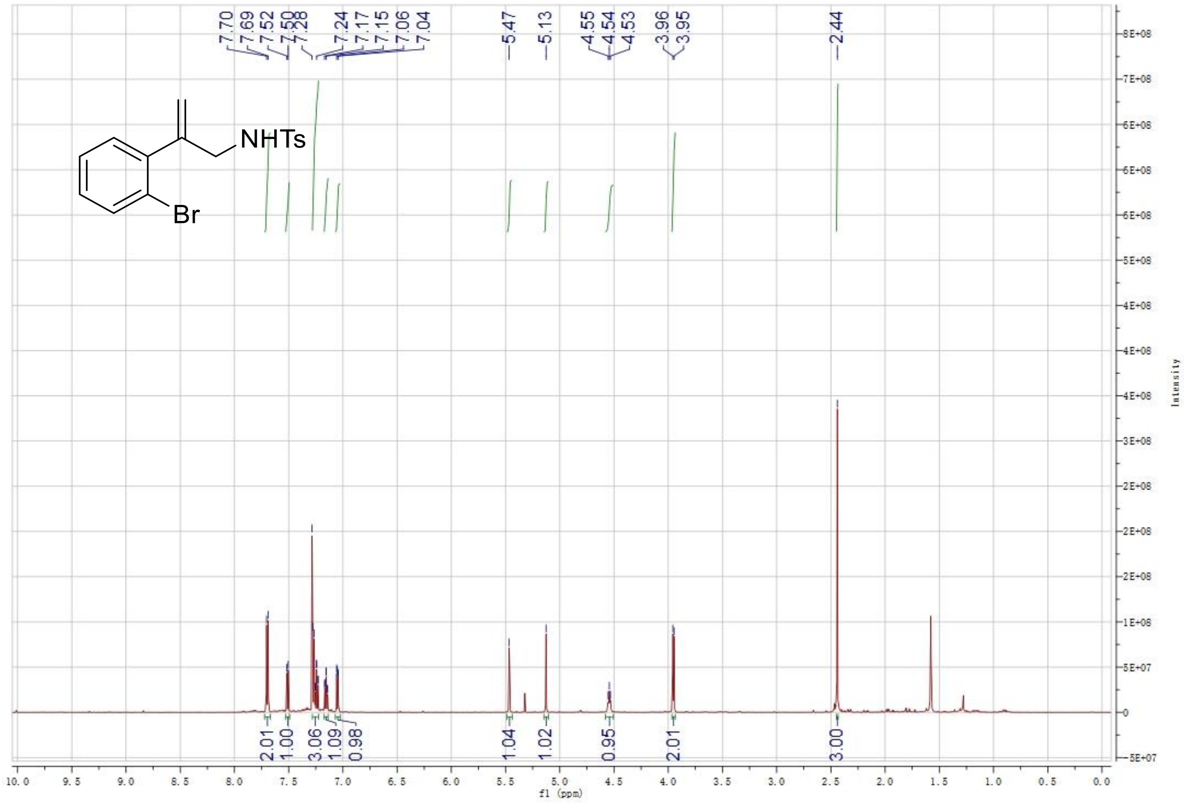

^{13}C NMR (151 MHz, 293 K, $CDCl_3$)

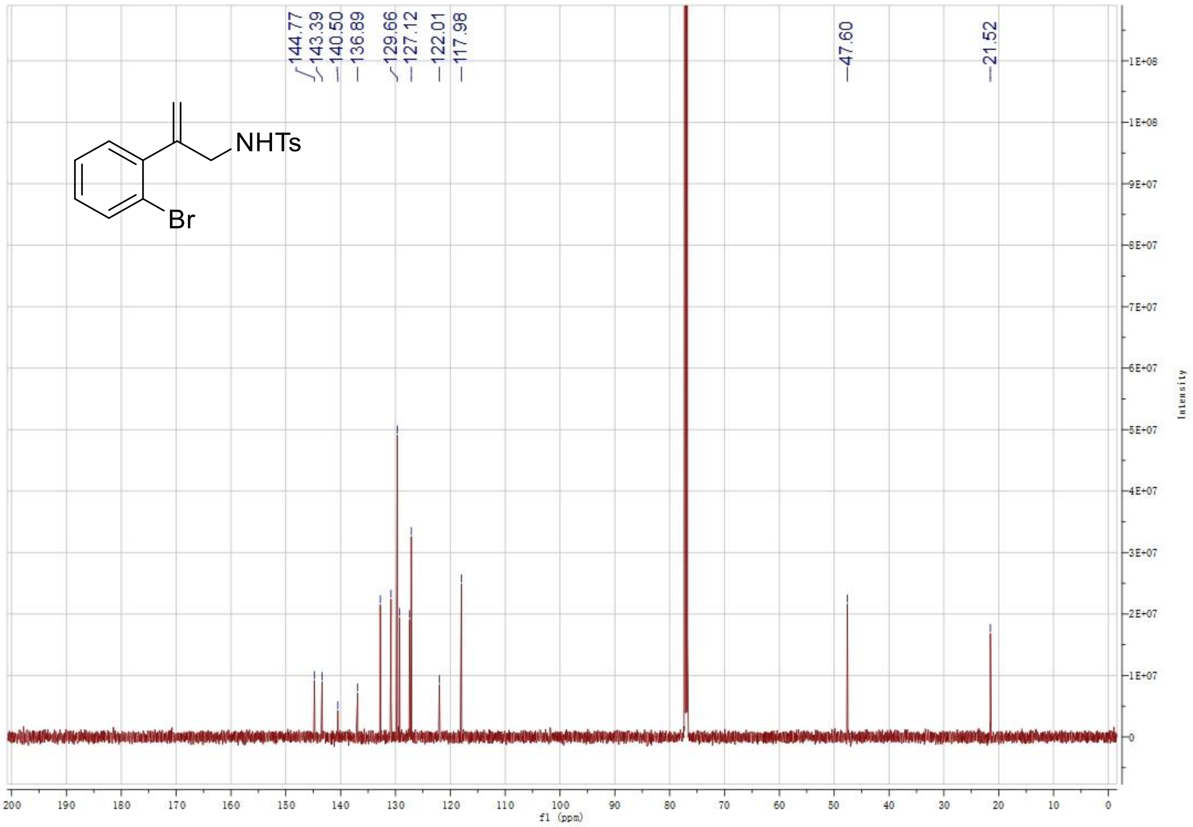

Supplementary Figure 29. *4-Methyl-N-(2-(o-tolyl)allyl)benzenesulfonamide (**10n**)*

^{1}H NMR (600 MHz, 293 K, $CDCl_3$)

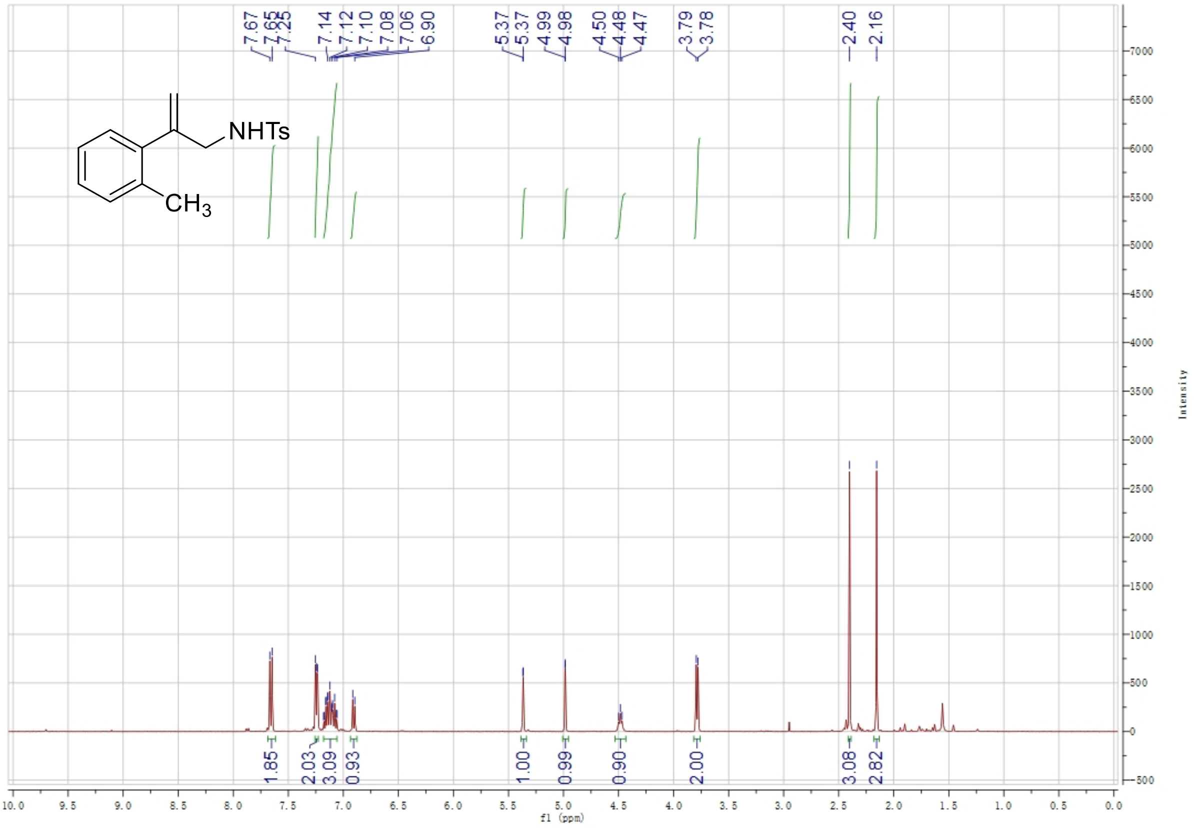

^{13}C NMR (151 MHz, 293 K, $CDCl_3$)

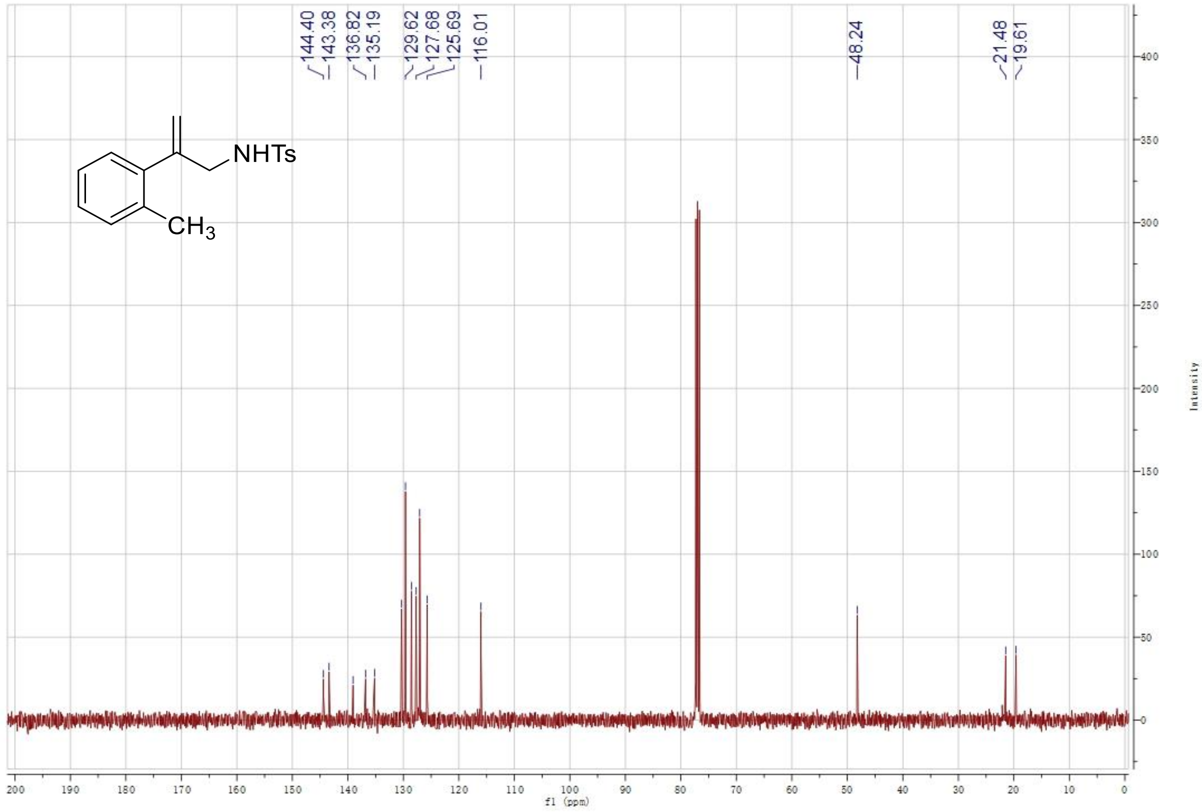

Supplementary Figure 30.

*N-(2-(3,5-Bis(Trifluoromethyl)phenyl)allyl)-4-methylbenzenesulfonamide (**10o**)*

^{1}H NMR (600 MHz, 293 K, $CDCl_3$)

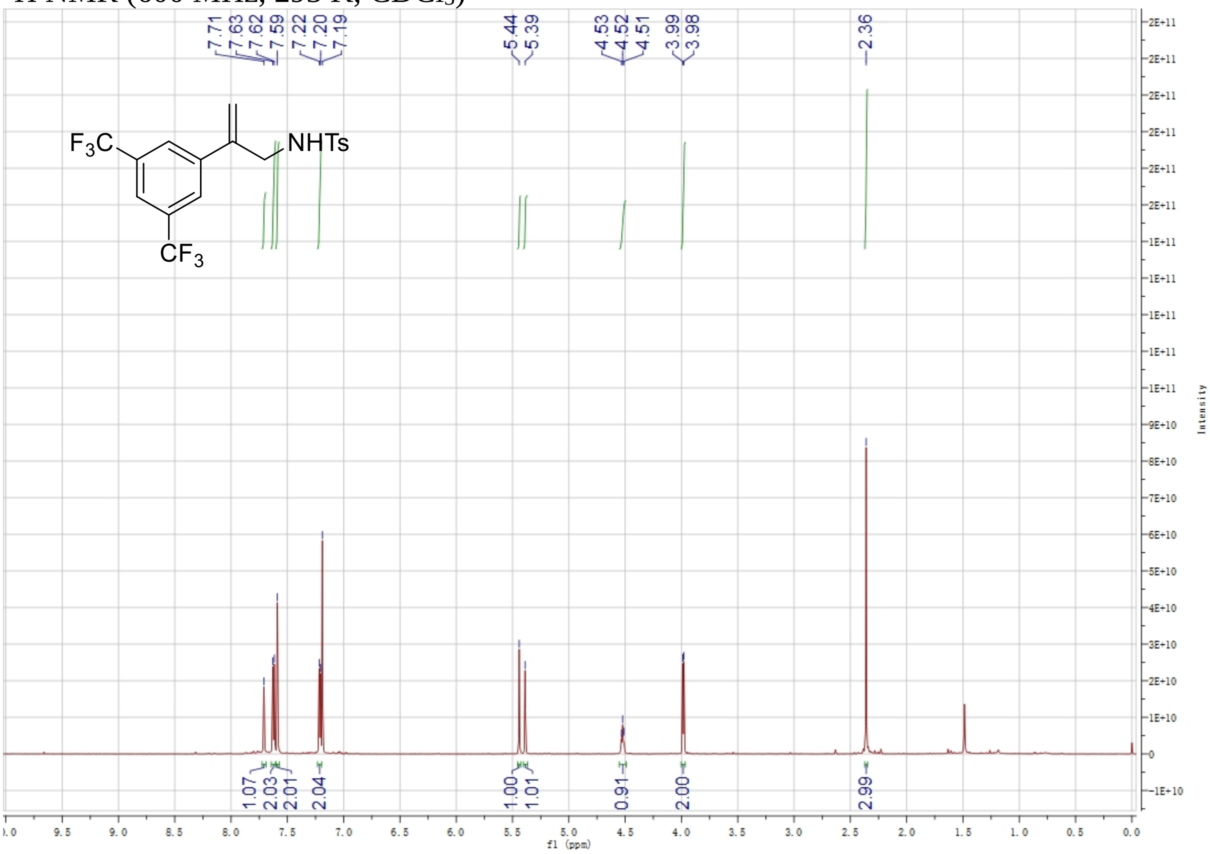

^{13}C NMR (151 MHz, 293 K, $CDCl_3$)

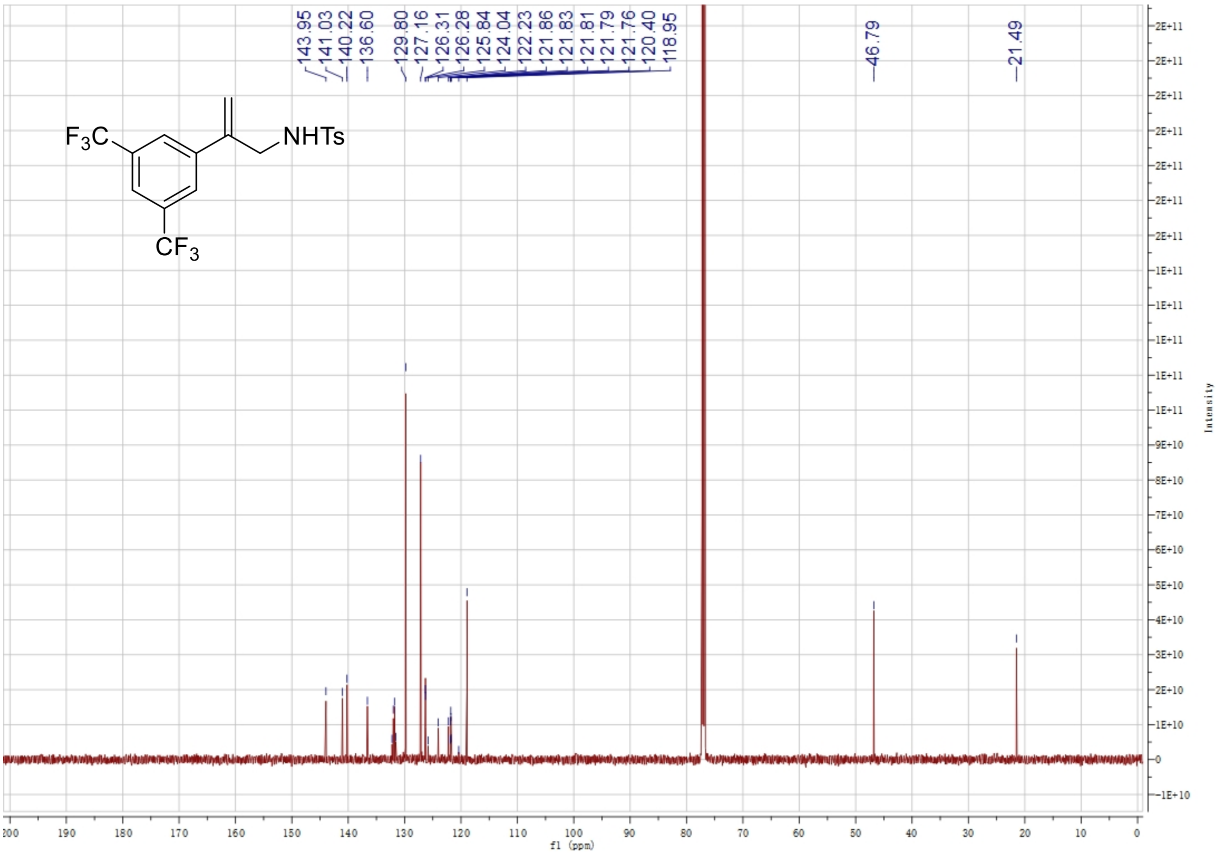

^{19}F NMR (565 MHz, 293 K, $CDCl_3$)

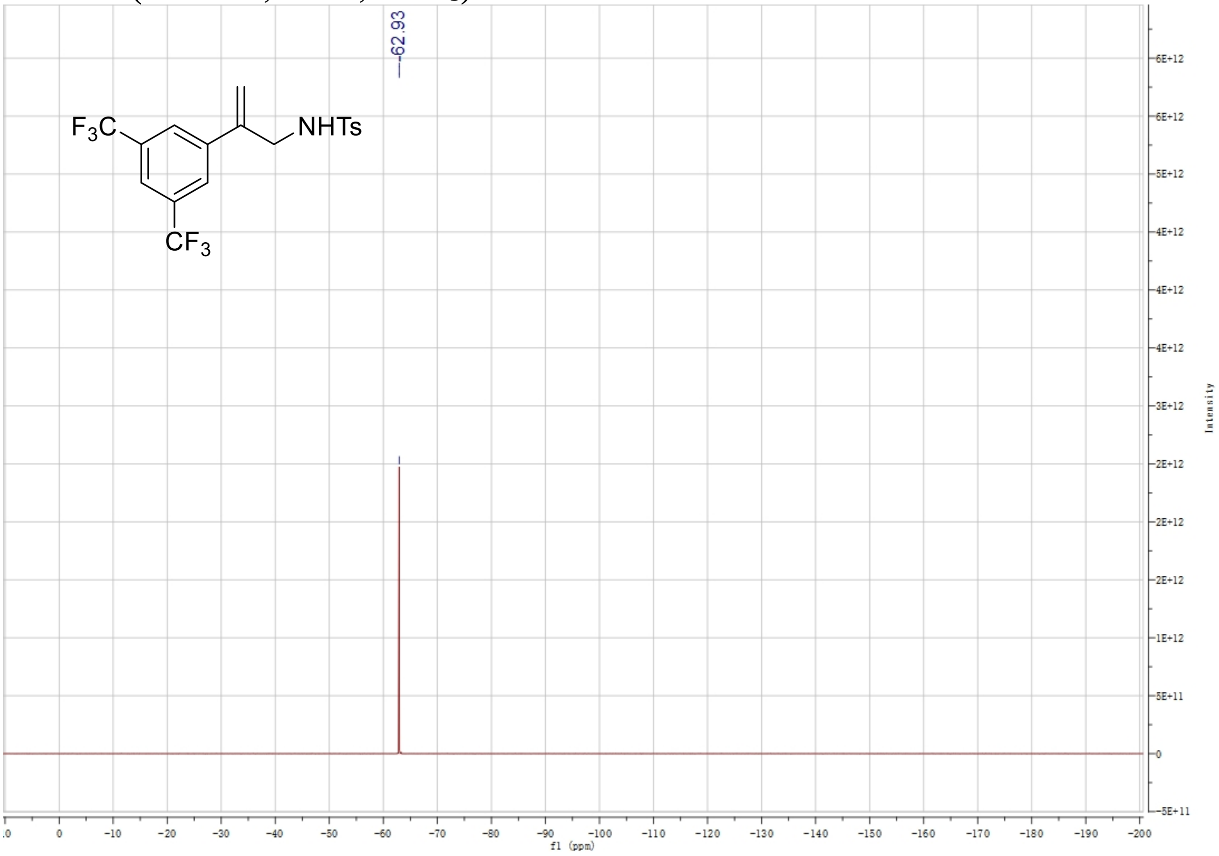

Supplementary Figure 31. *4-Chloro-N-(2-phenylallyl)benzenesulfonamide (**10p**)*

^{1}H NMR (600 MHz, 293 K, $CDCl_3$)

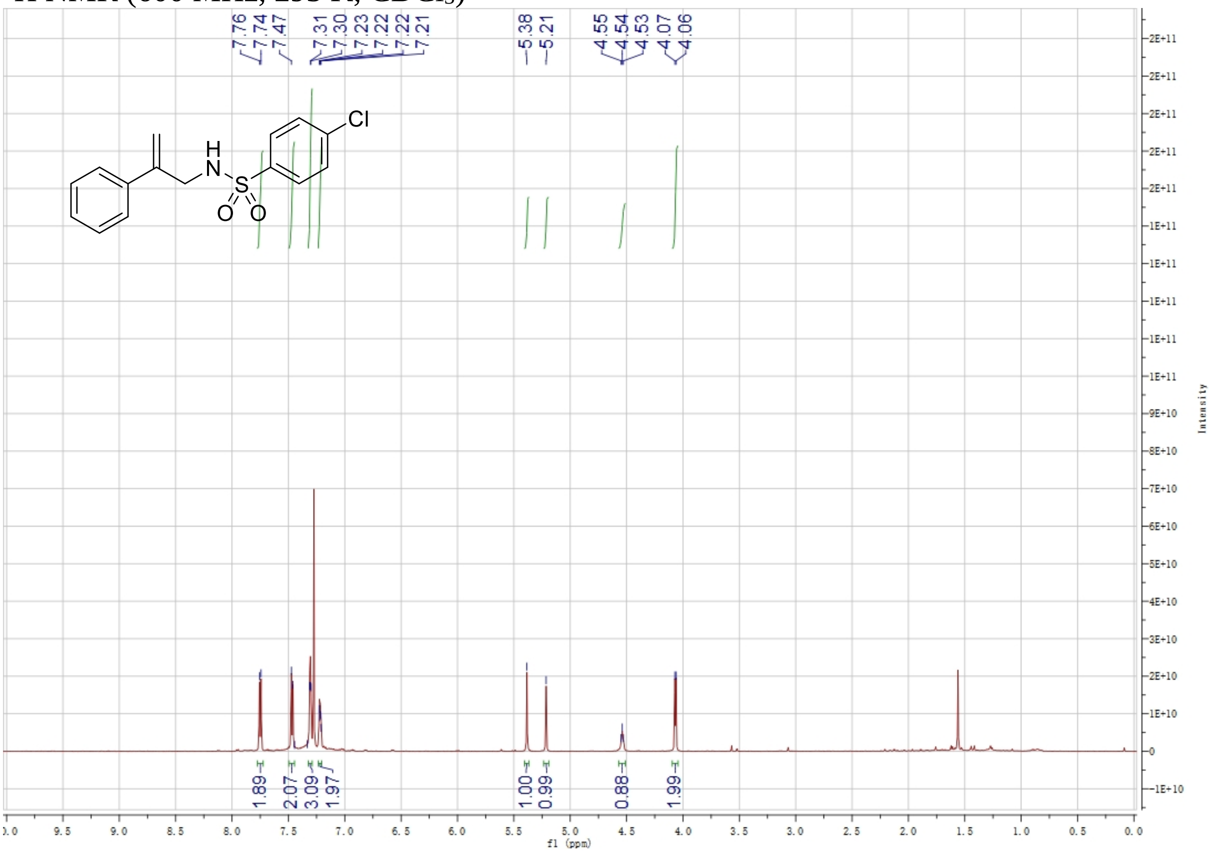

^{13}C NMR (151 MHz, 293 K, $CDCl_3$)

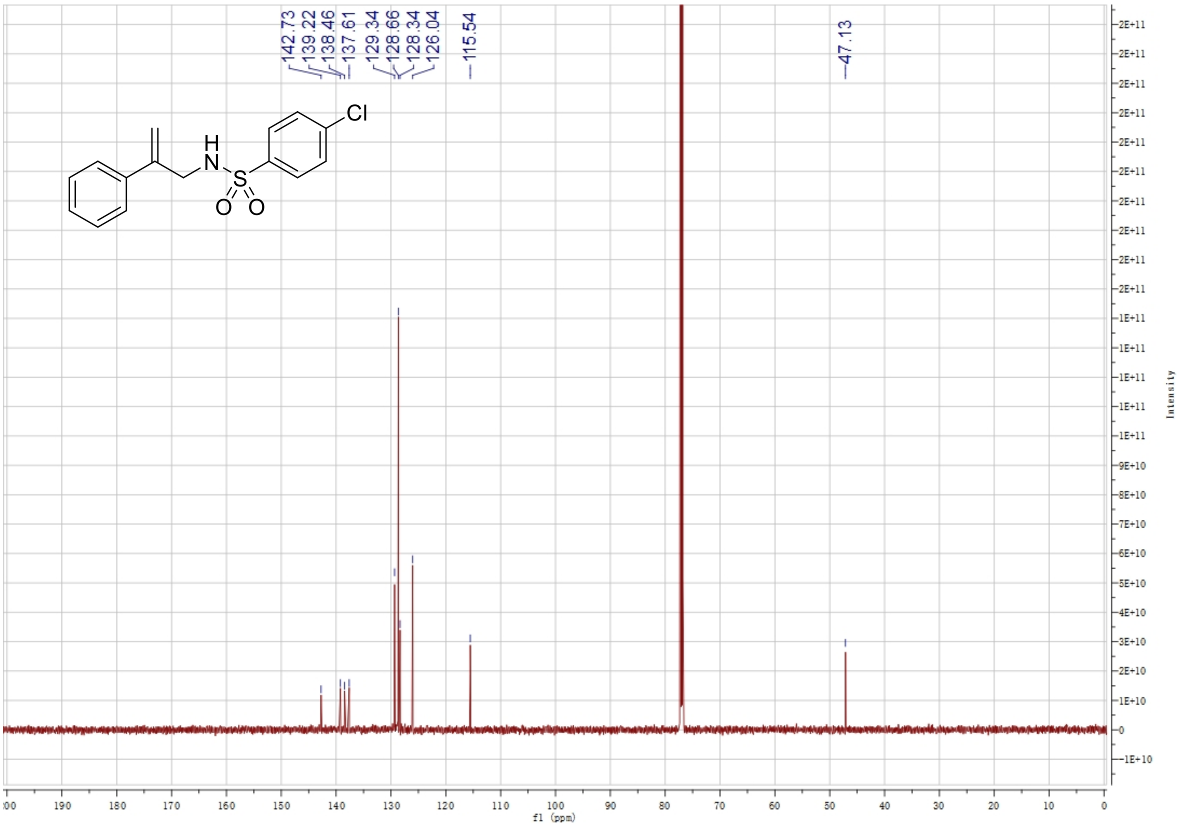

Supplementary Figure 32. *2-Nitro-N-(2-phenylallyl)benzenesulfonamide (**10q**)*

^{1}H NMR (600 MHz, 293 K, $CDCl_3$)

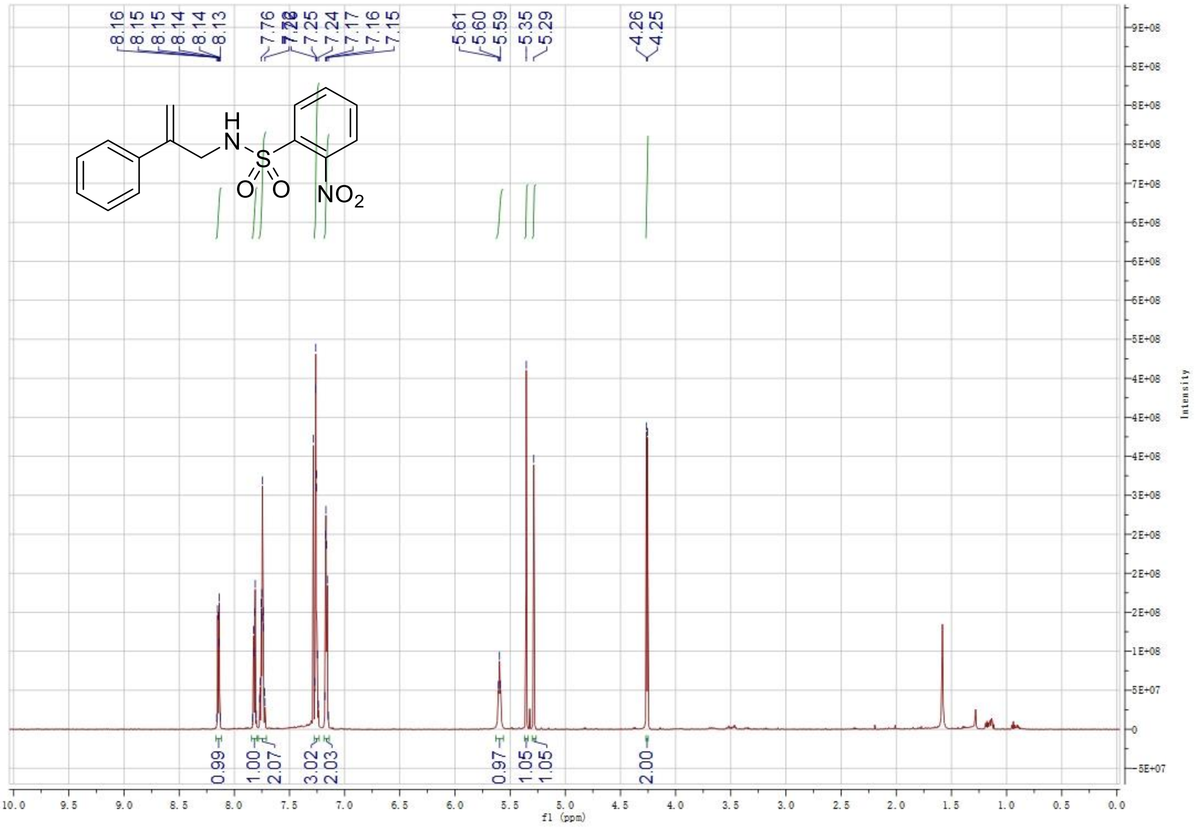

^{13}C NMR (151 MHz, 293 K, $CDCl_3$)

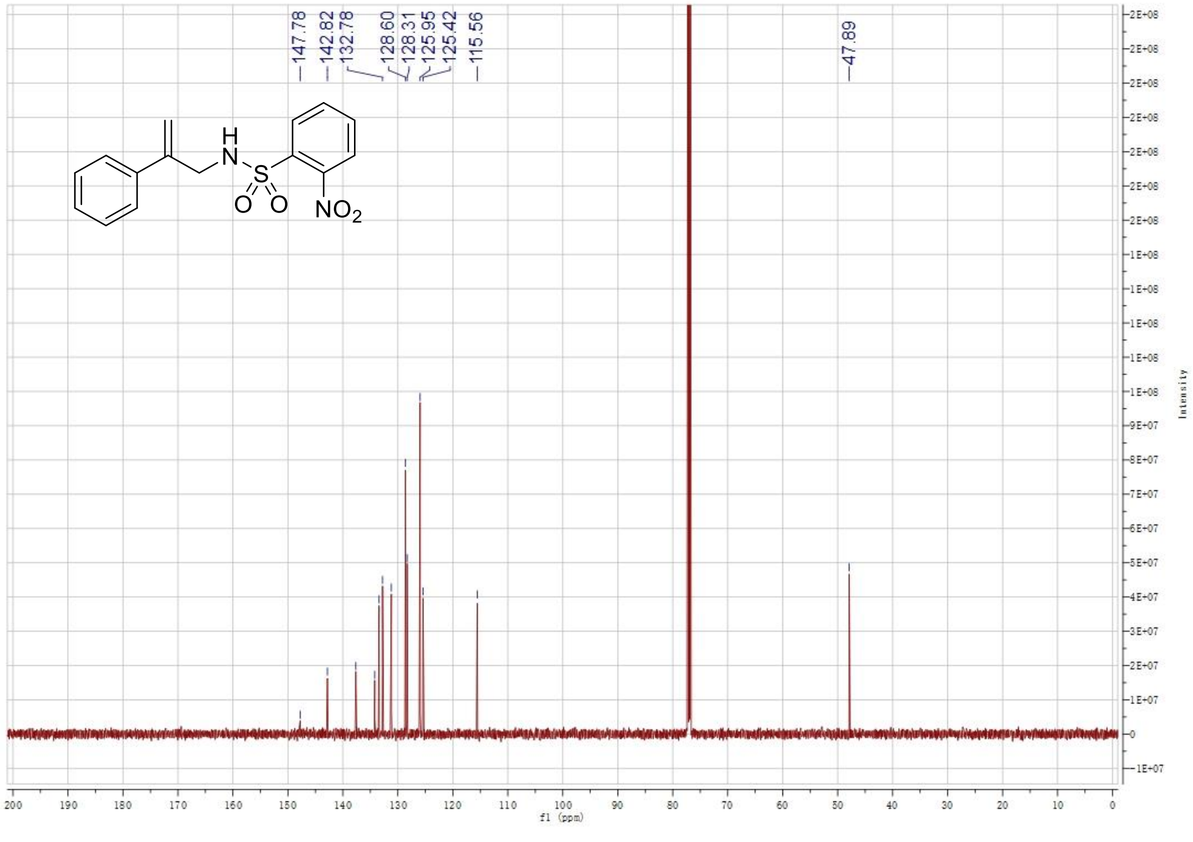

Supplementary Figure 33.

*4-Methyl-N-(2,3,4,5-tetrahydro-[1,1'-biphenyl]-2-yl)benzenesulfonamide (**10r**)*

^{1}H NMR (600 MHz, 293 K, $CDCl_3$)

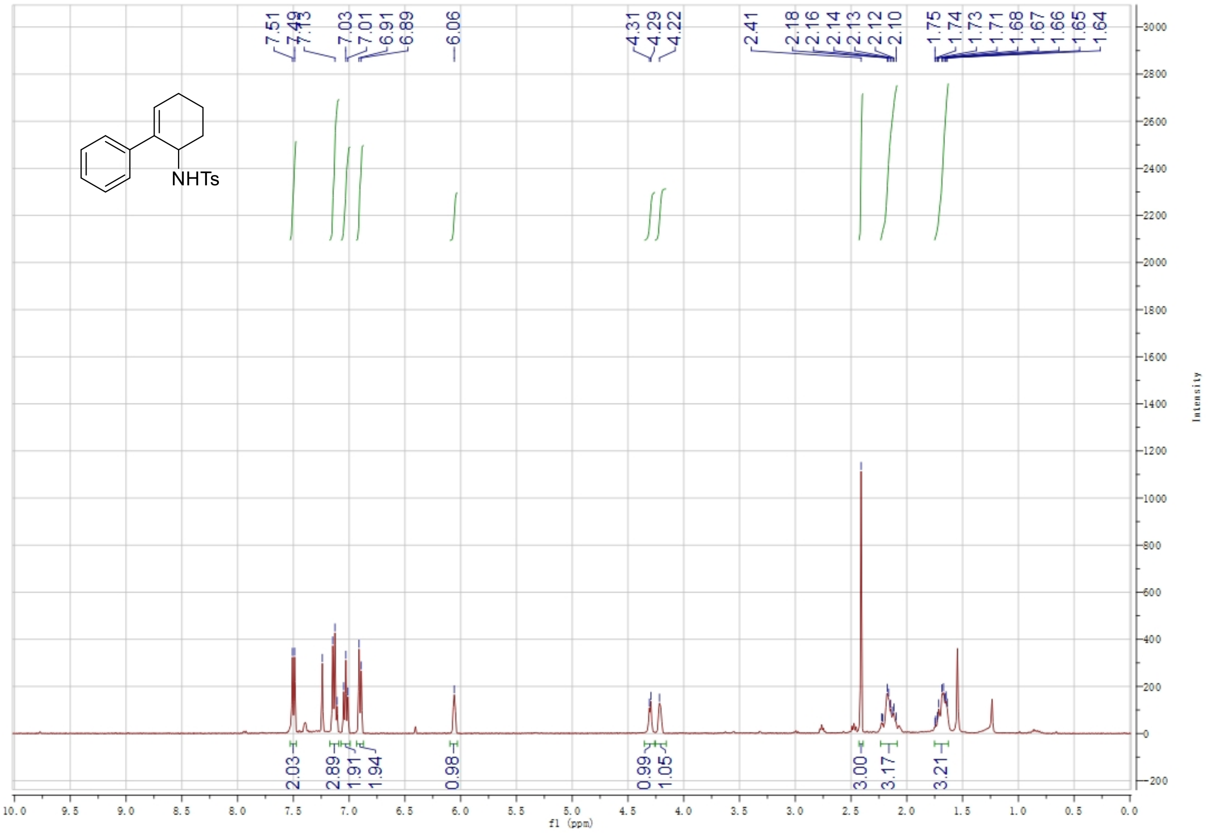

^{13}C NMR (151 MHz, 293 K, $CDCl_3$)

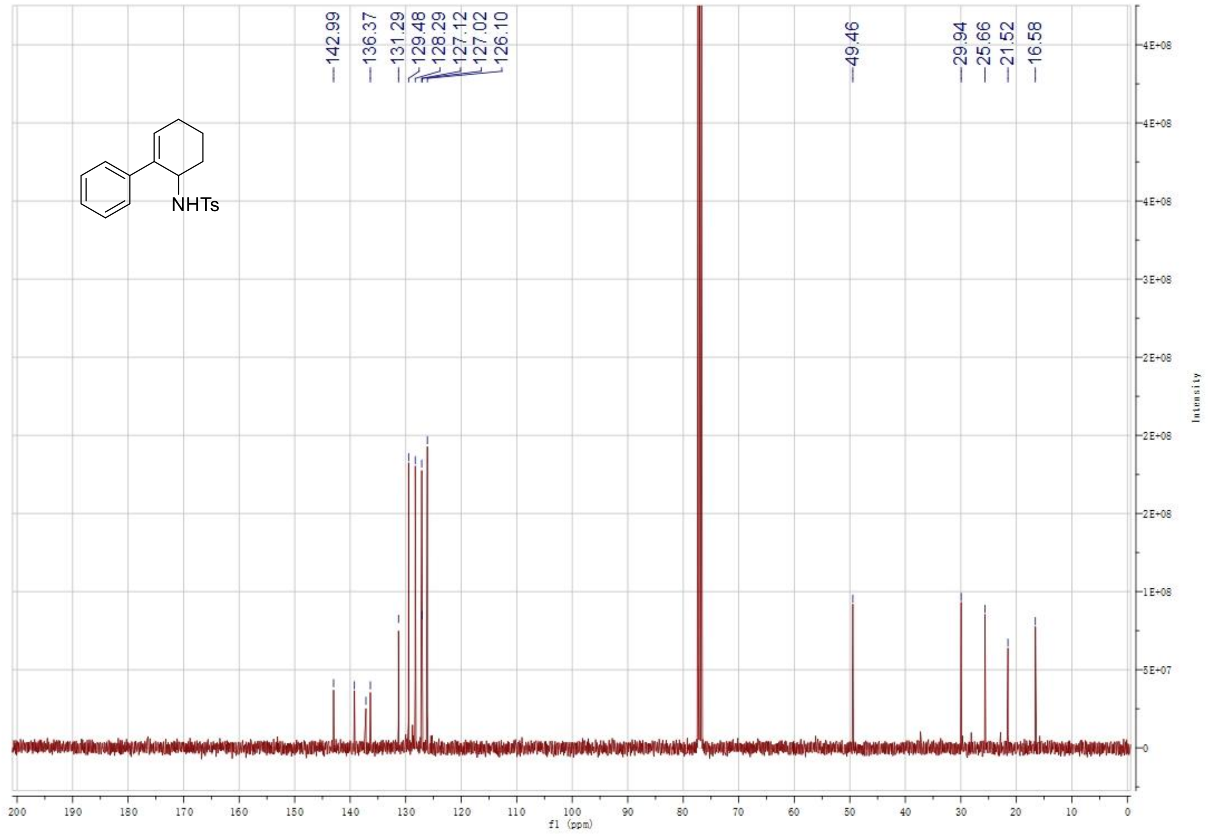

Supplementary Figure 34. *4-Methyl-N-(2-methylcyclopent-2-en-1-yl)benzenesulfonamide (10s)*

^{1}H NMR (600 MHz, 293 K, $CDCl_3$)

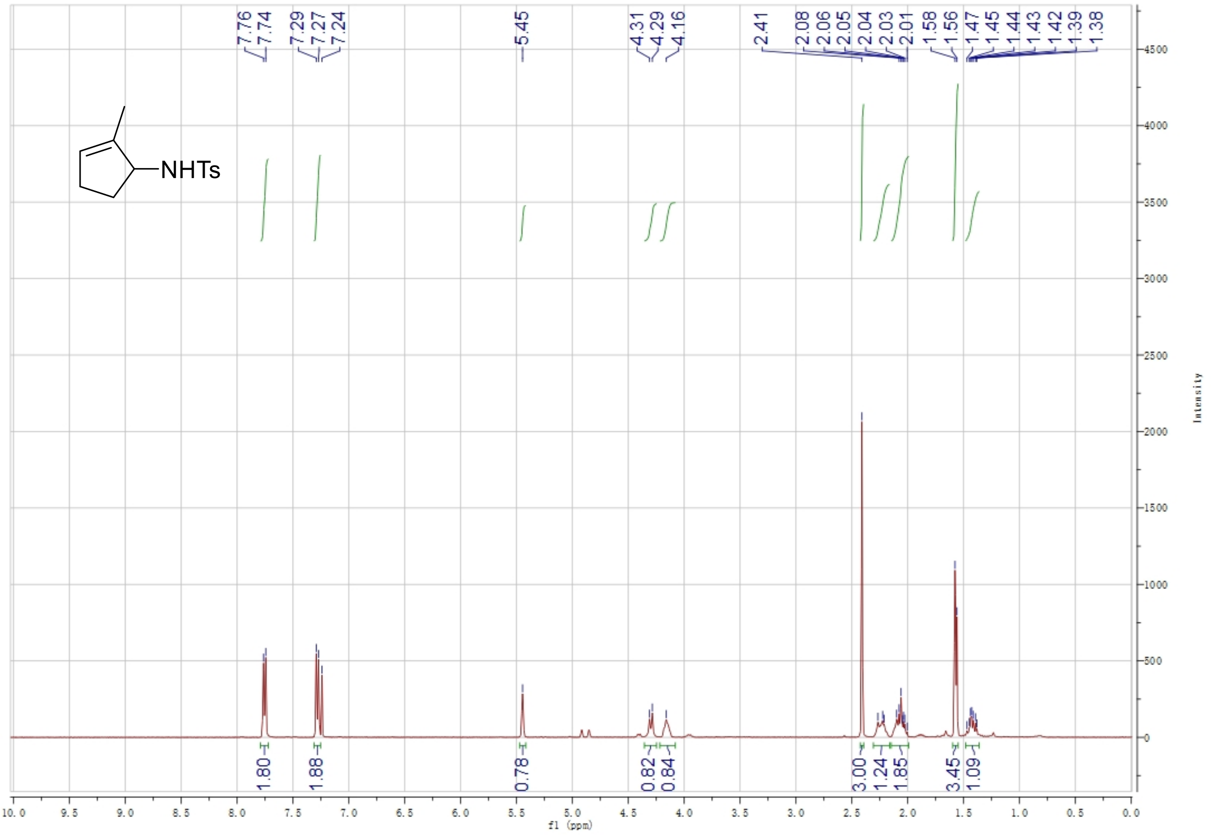

^{13}C NMR (151 MHz, 293 K, $CDCl_3$)

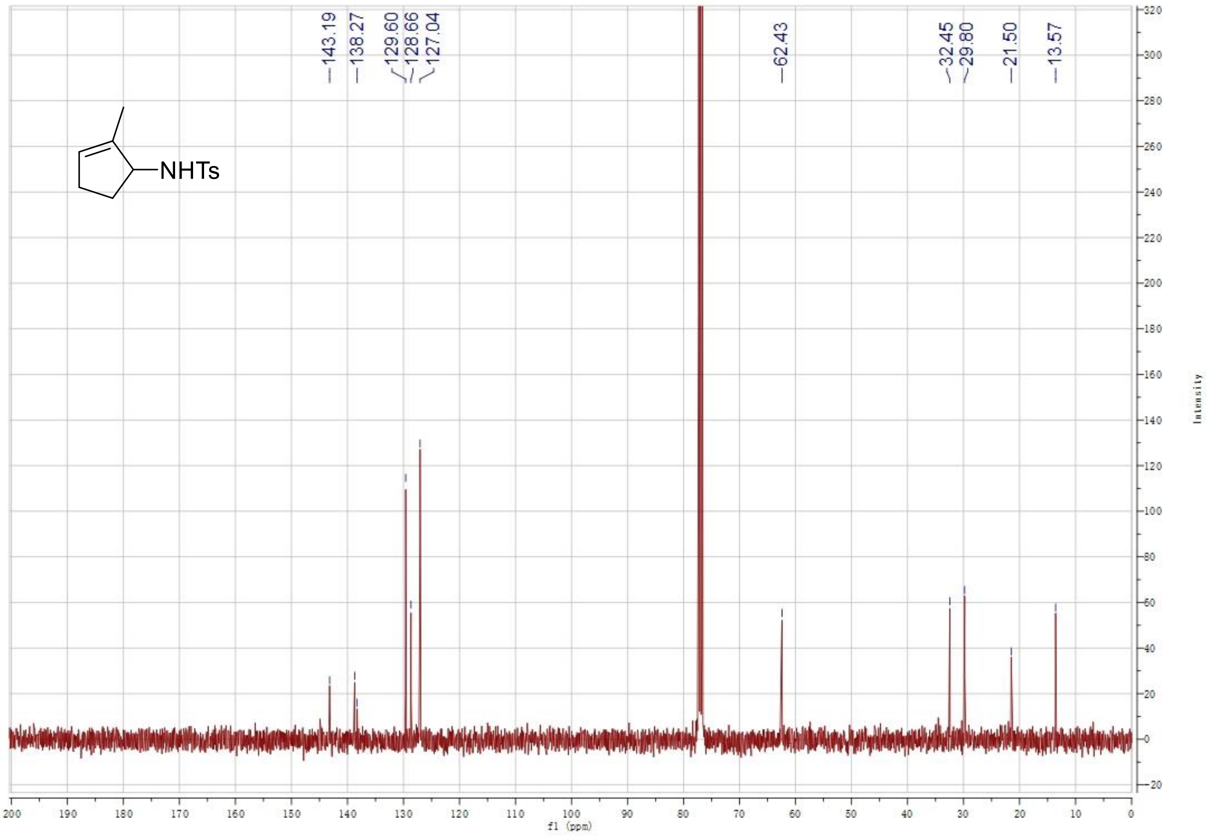

Supplementary Figure 35.

*4-Methyl-N-(2-methylene-4-phenylbut-3-yn-1-yl)benzenesulfonamide (**10t**)*

^{1}H NMR (600 MHz, 293 K, $CDCl_3$)

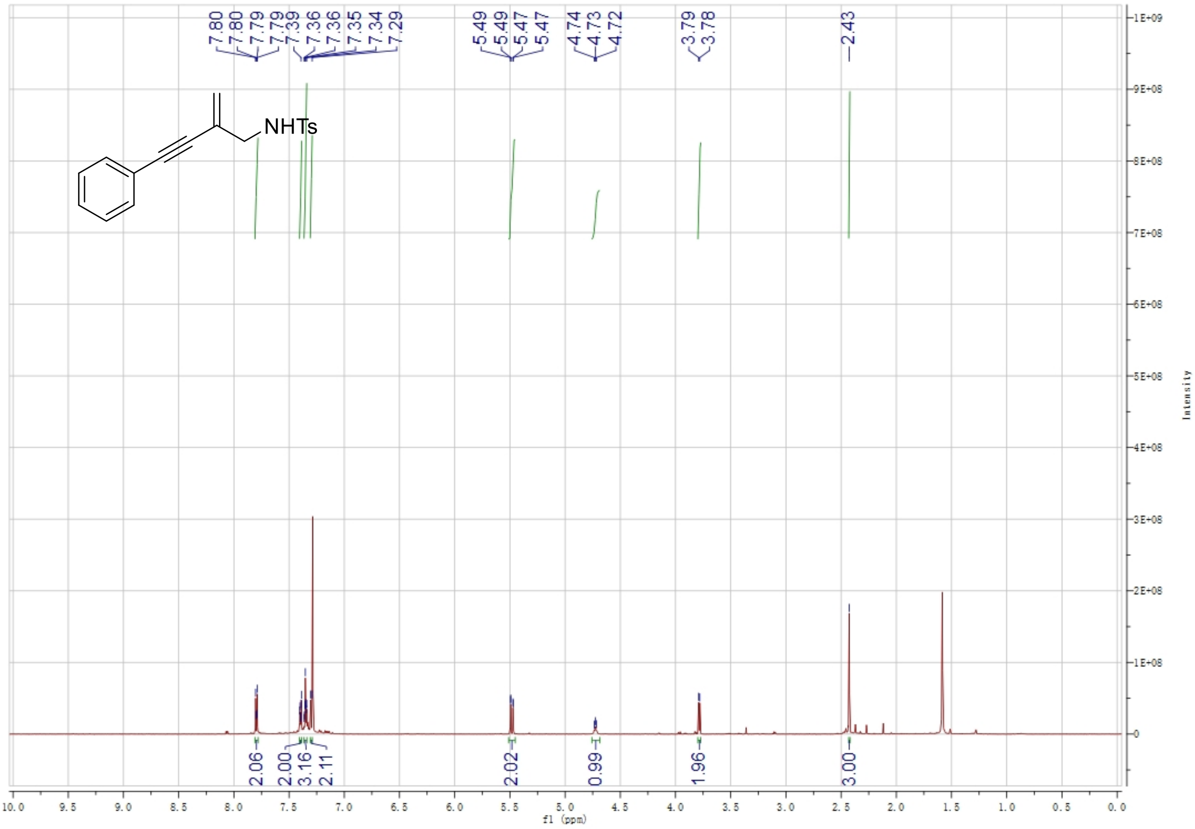

^{13}C NMR (151 MHz, 293 K, $CDCl_3$)

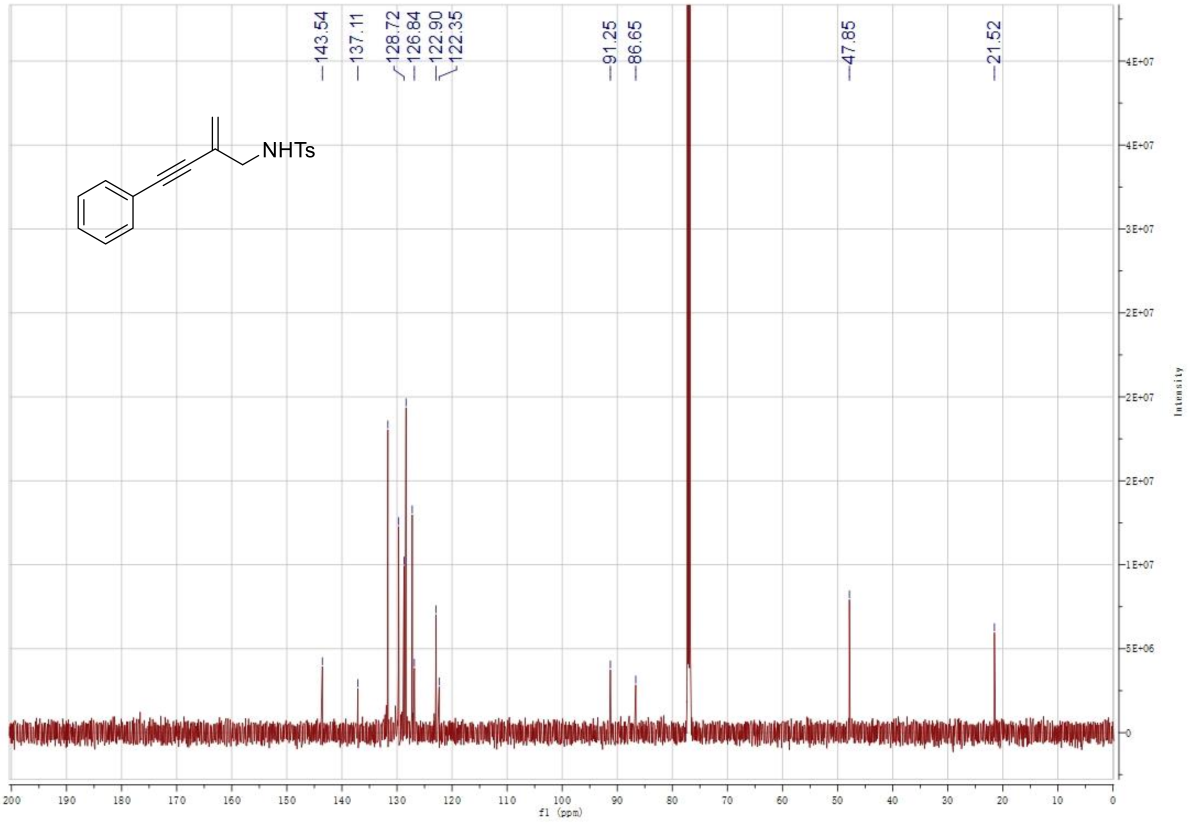

Supplementary Figure 36. *N-(2-Cyclohexylallyl)-4-methylbenzenesulfonamide* (***10u***)

^{1}H NMR (600 MHz, 293 K, $CDCl_3$)

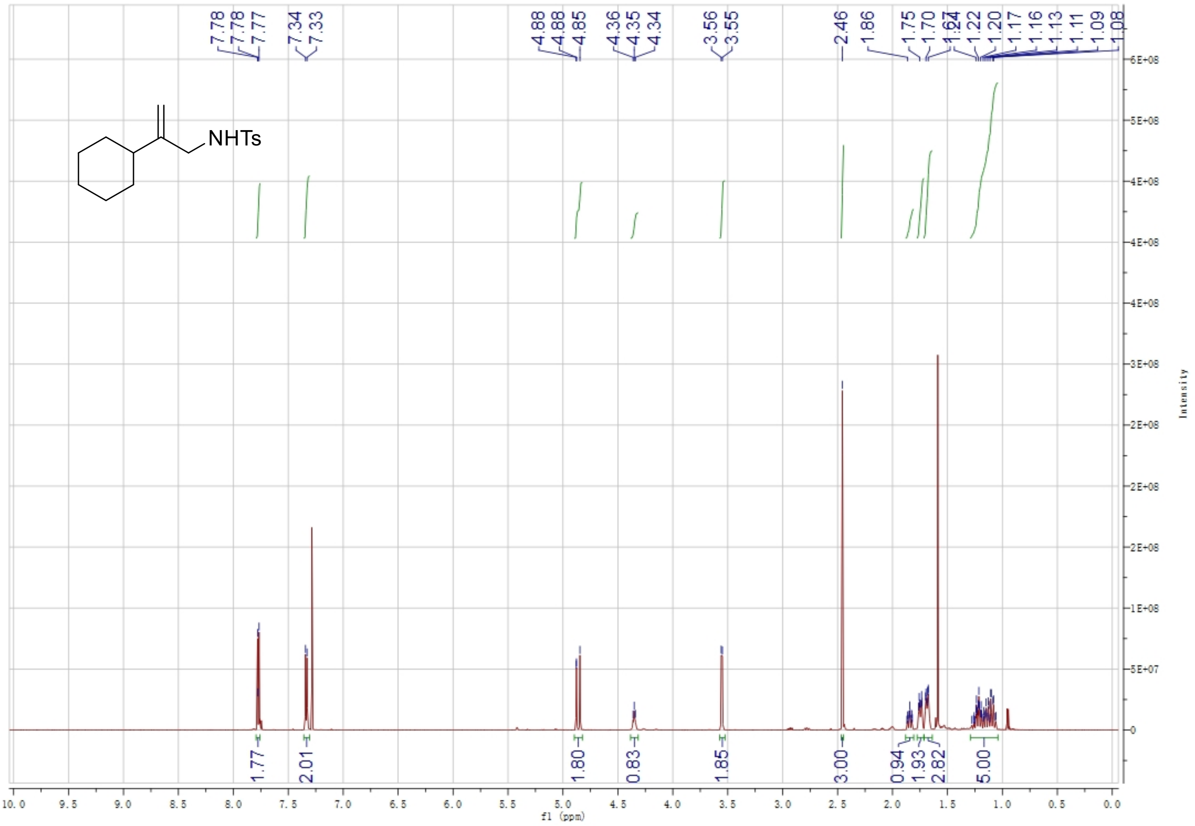

^{13}C NMR (151 MHz, 293 K, $CDCl_3$)

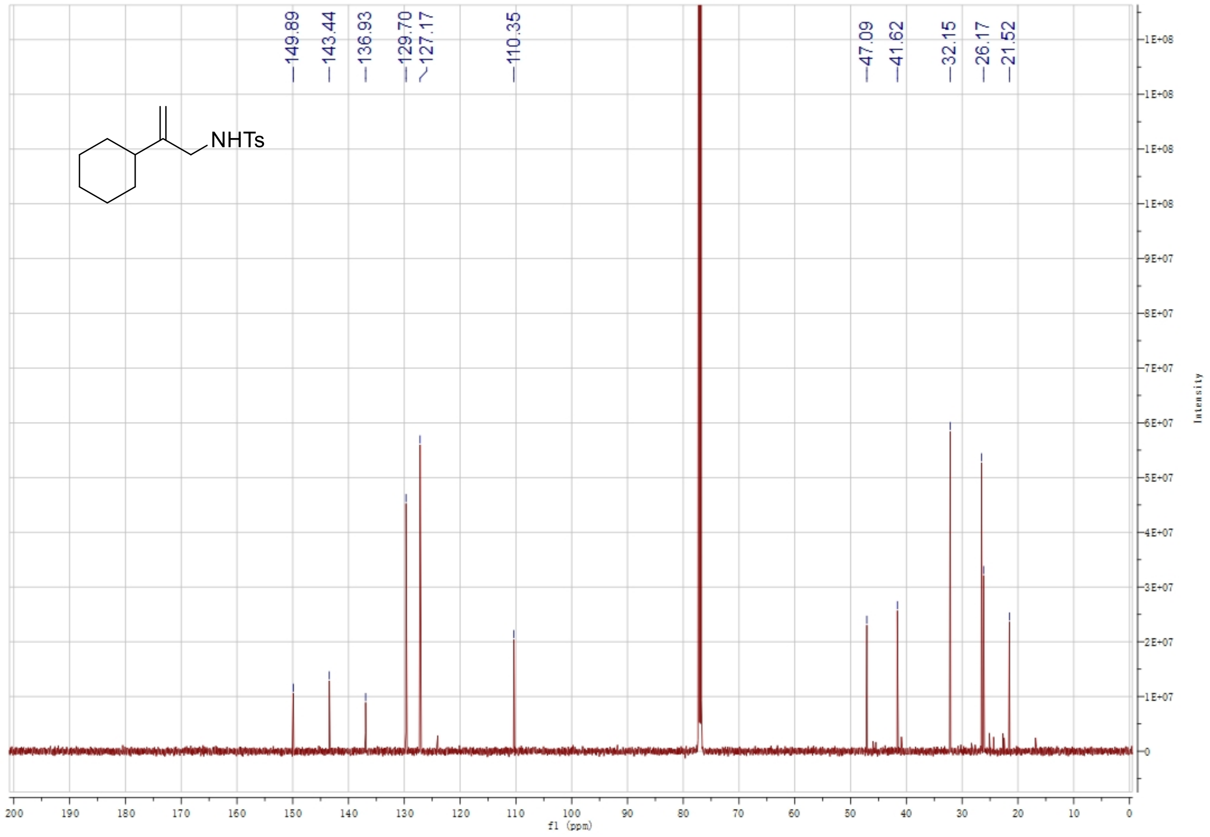

Supplementary Figure 37. *N-(3,3-Dimethyl-2-methylenebutyl)-4-methylbenzenesulfonamide (10v)*

^{1}H NMR (400 MHz, 293 K, $CDCl_3$)

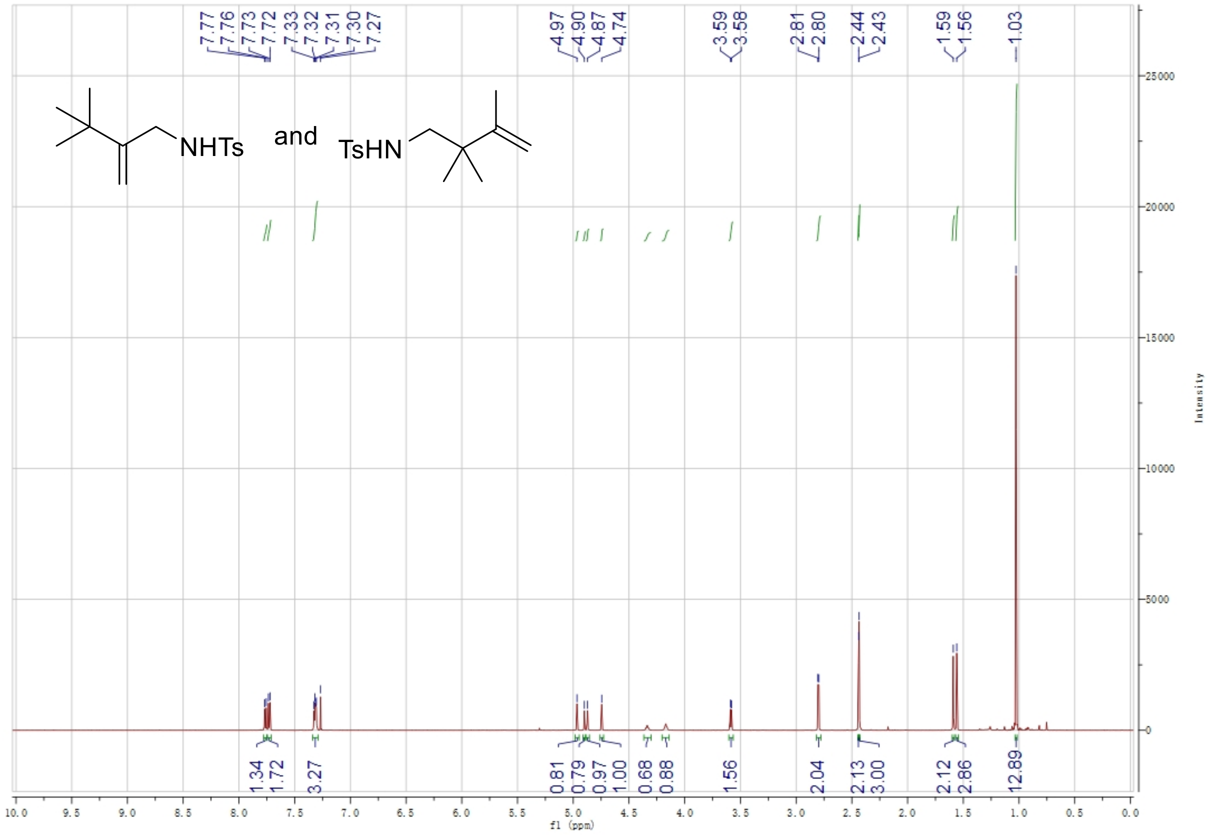

^{13}C NMR (101 MHz, 293 K, $CDCl_3$)

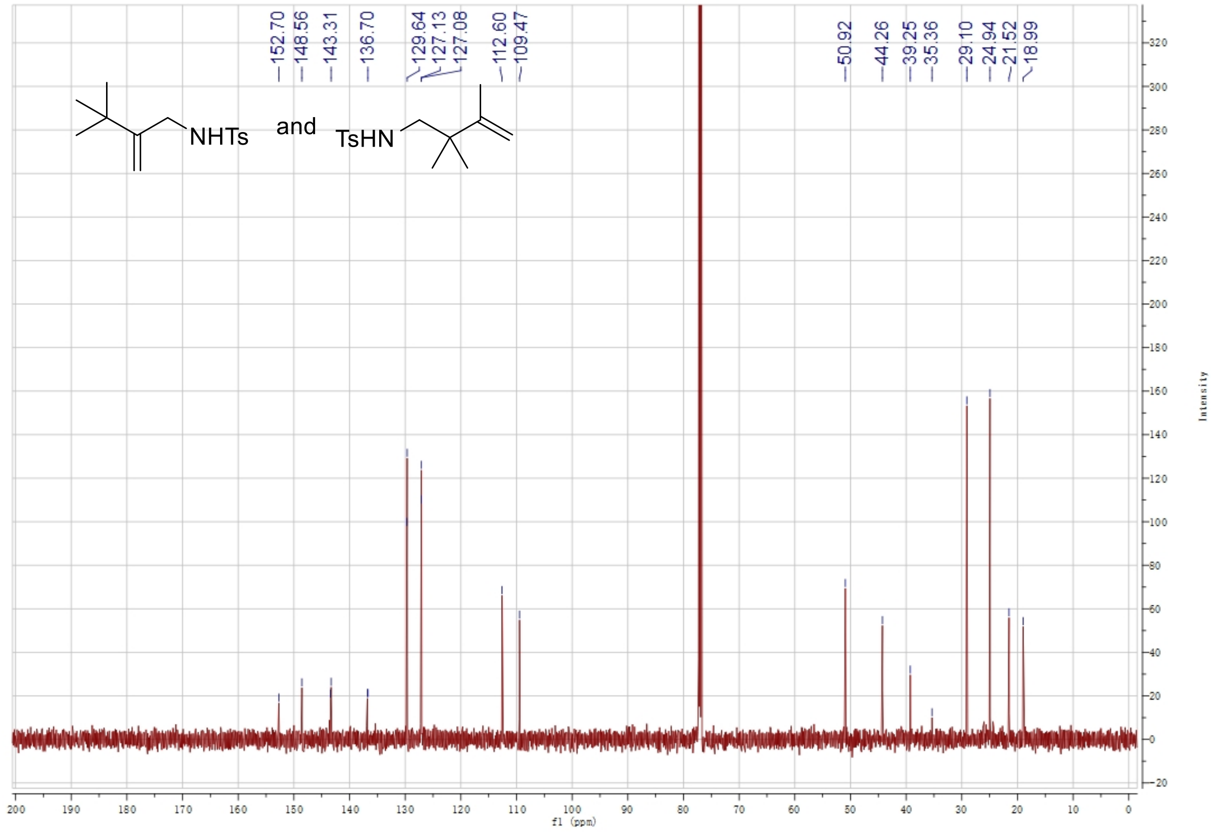

Supplementary Figure 38. *2-Methyl-2-phenyl-1-tosylaziridine (**11a**)*

^{1}H NMR (400 MHz, 293 K, $CDCl_3$)

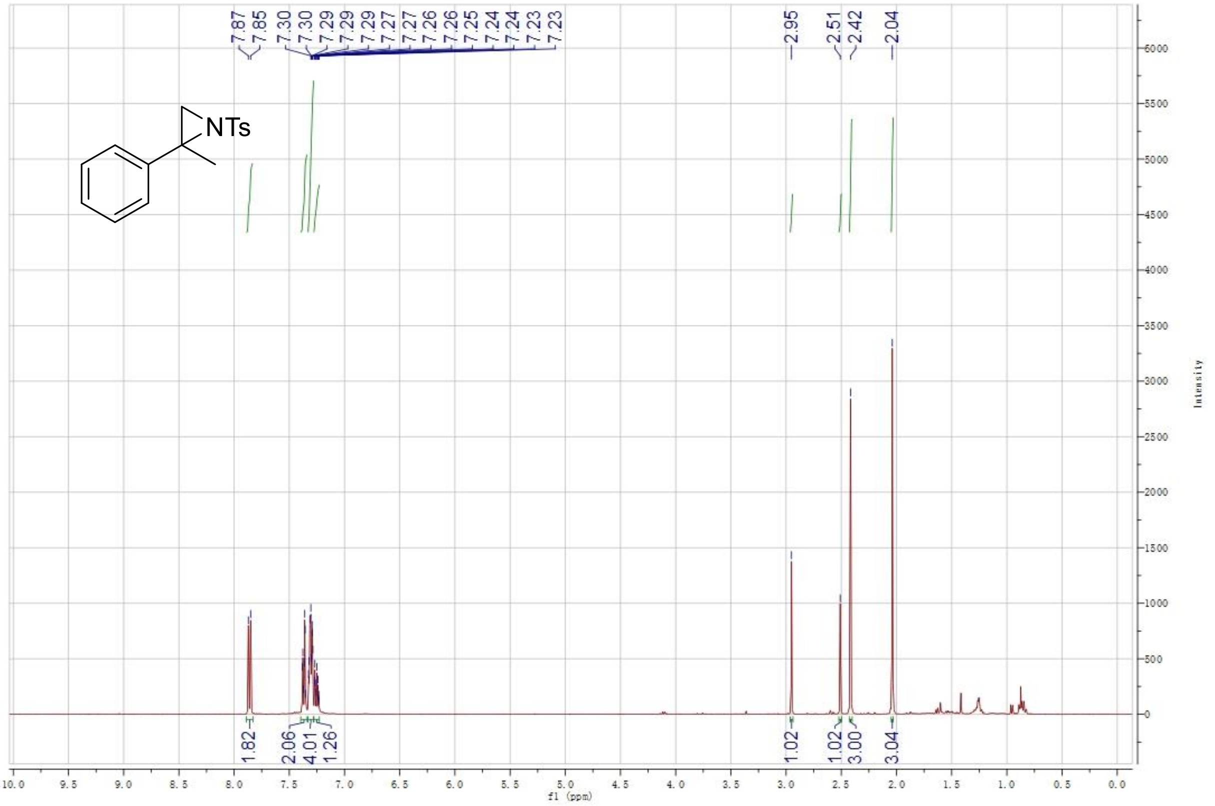

^{13}C NMR (101 MHz, 293 K, $CDCl_3$)

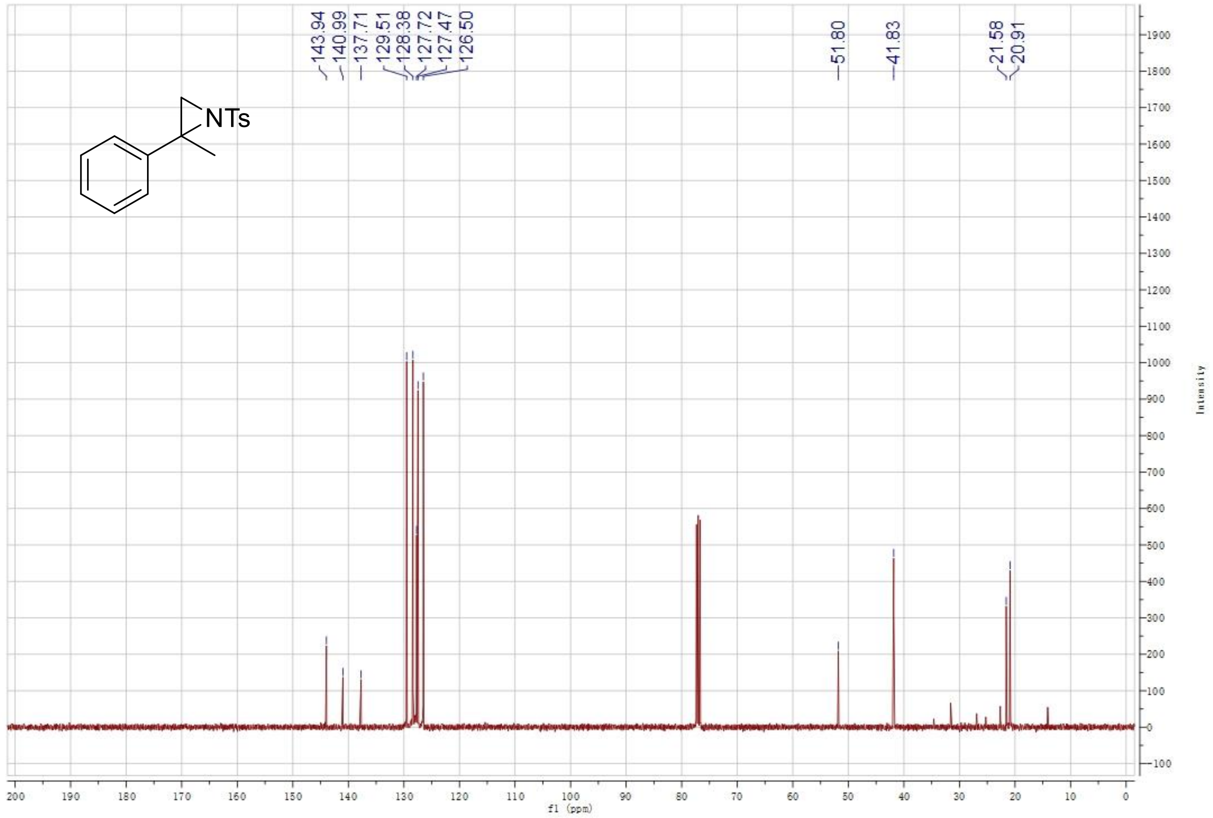

Supplementary Figure 39. *2-(Methyl-d3)-2-phenyl-1-tosylaziridine (**11a-d3**)*

^{1}H NMR (400 MHz, 293 K, $CDCl_3$)

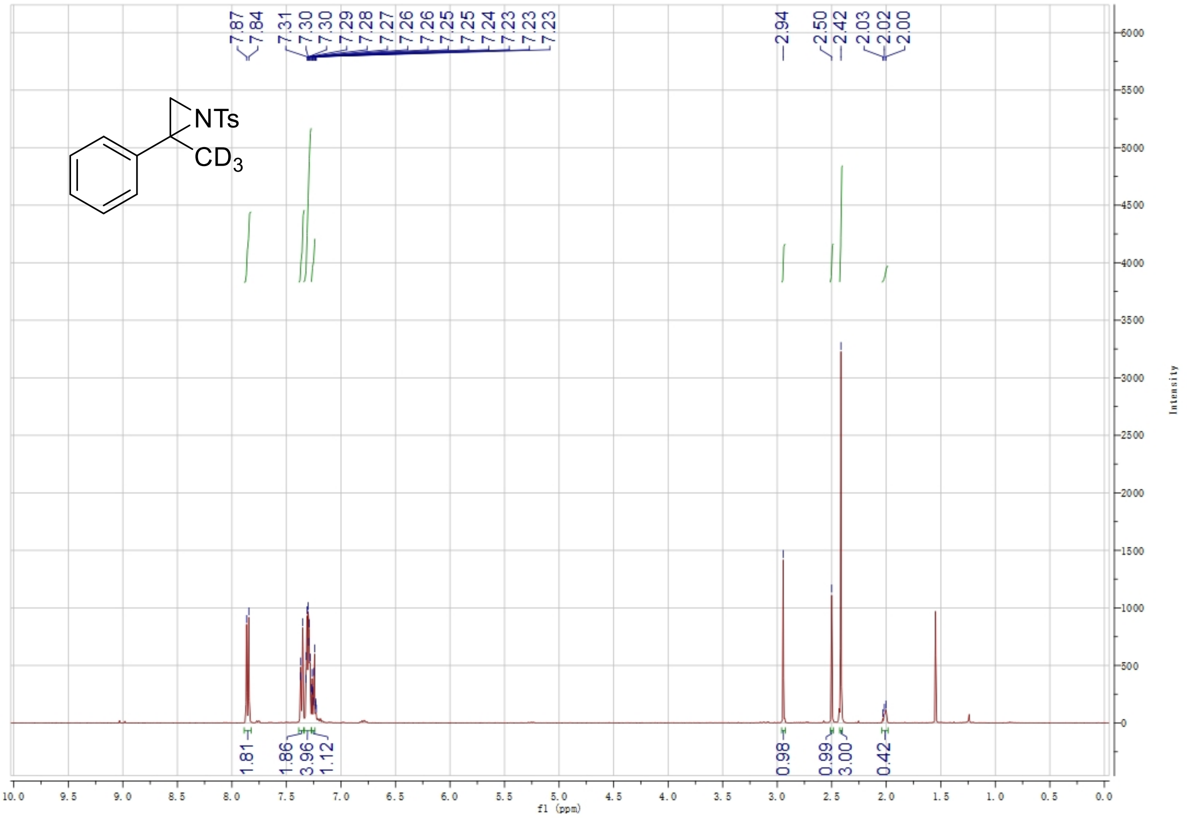

^{13}C NMR (101 MHz, 293 K, $CDCl_3$)

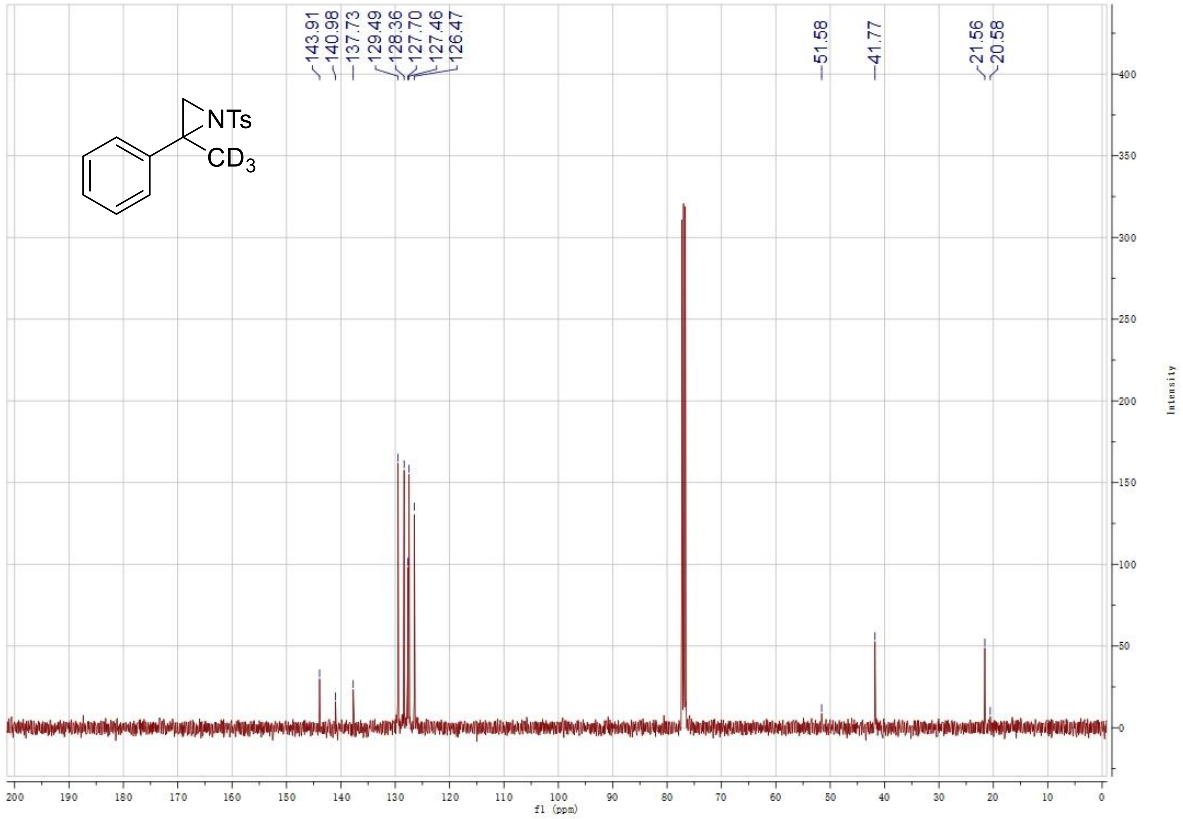

Supplementary Figure 40. *2-(4-Chlorophenyl)-2-methyl-1-tosylaziridine (**11b**)*

^{1}H NMR (400 MHz, 293 K, $CDCl_3$)

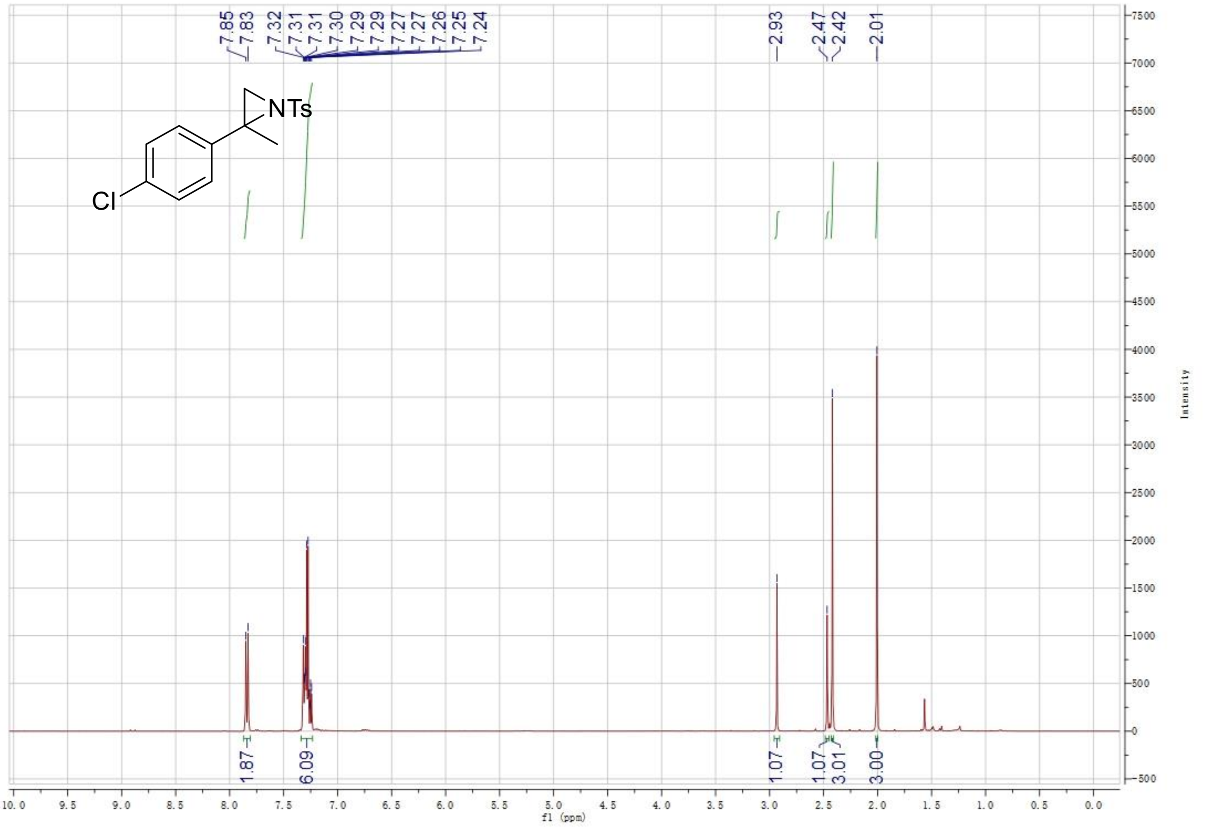

^{13}C NMR (101 MHz, 293 K, $CDCl_3$)

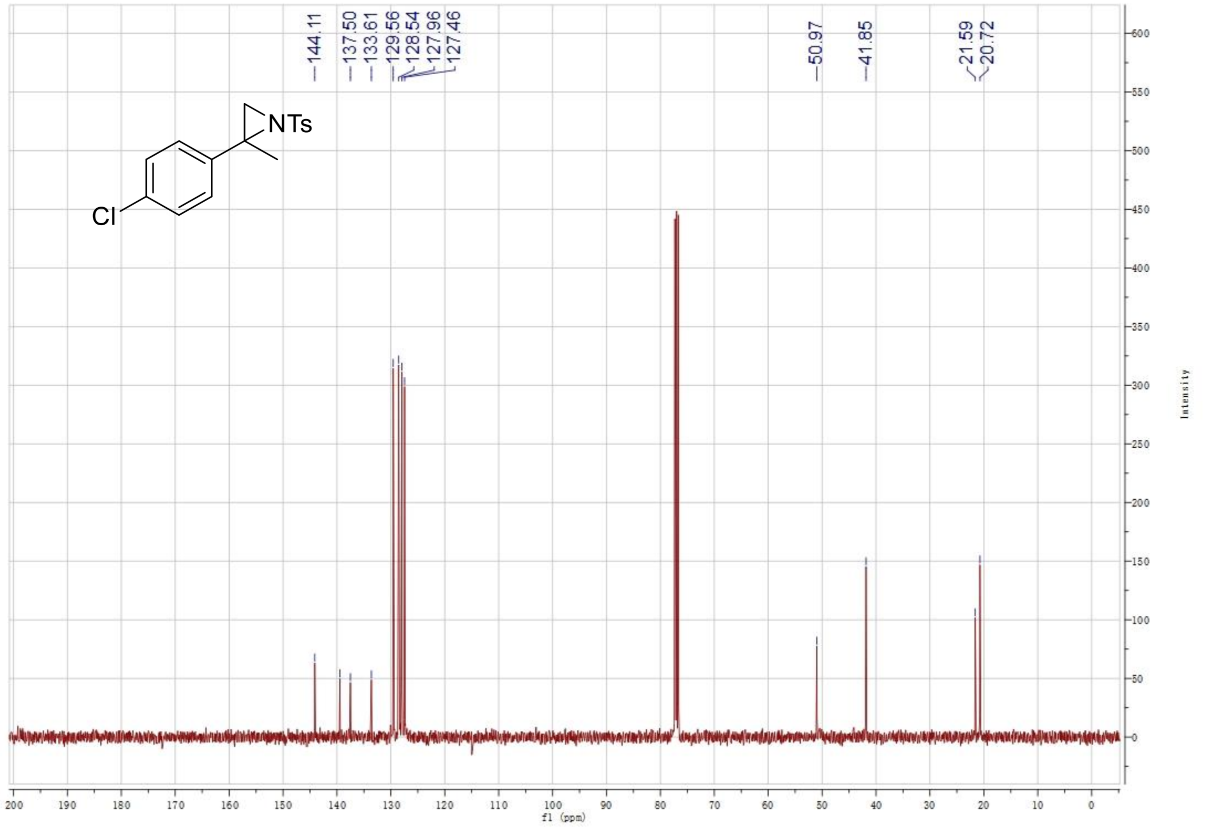

Supplementary Figure 41. *2-(4-Bromophenyl)-2-methyl-1-tosylaziridine (**11c**)*

^{1}H NMR (400 MHz, 293 K, $CDCl_3$)

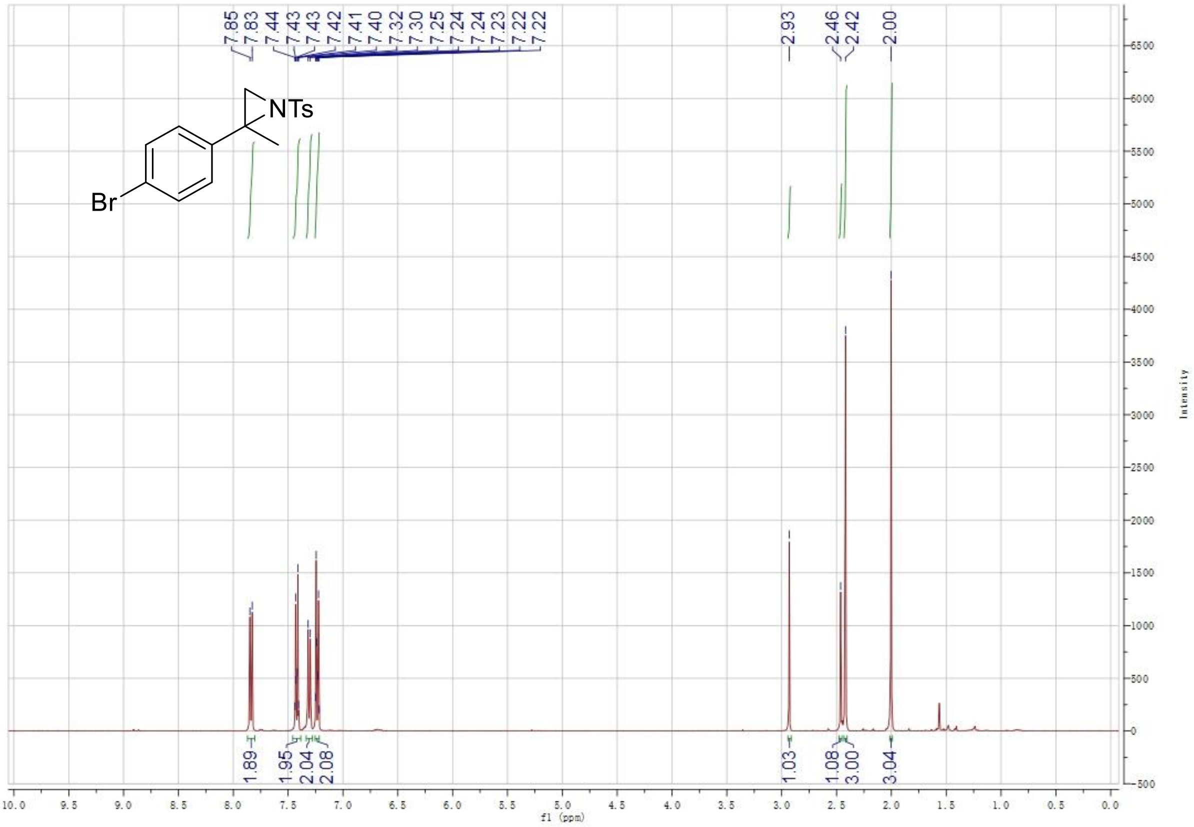

^{13}C NMR (101 MHz, 293 K, $CDCl_3$)

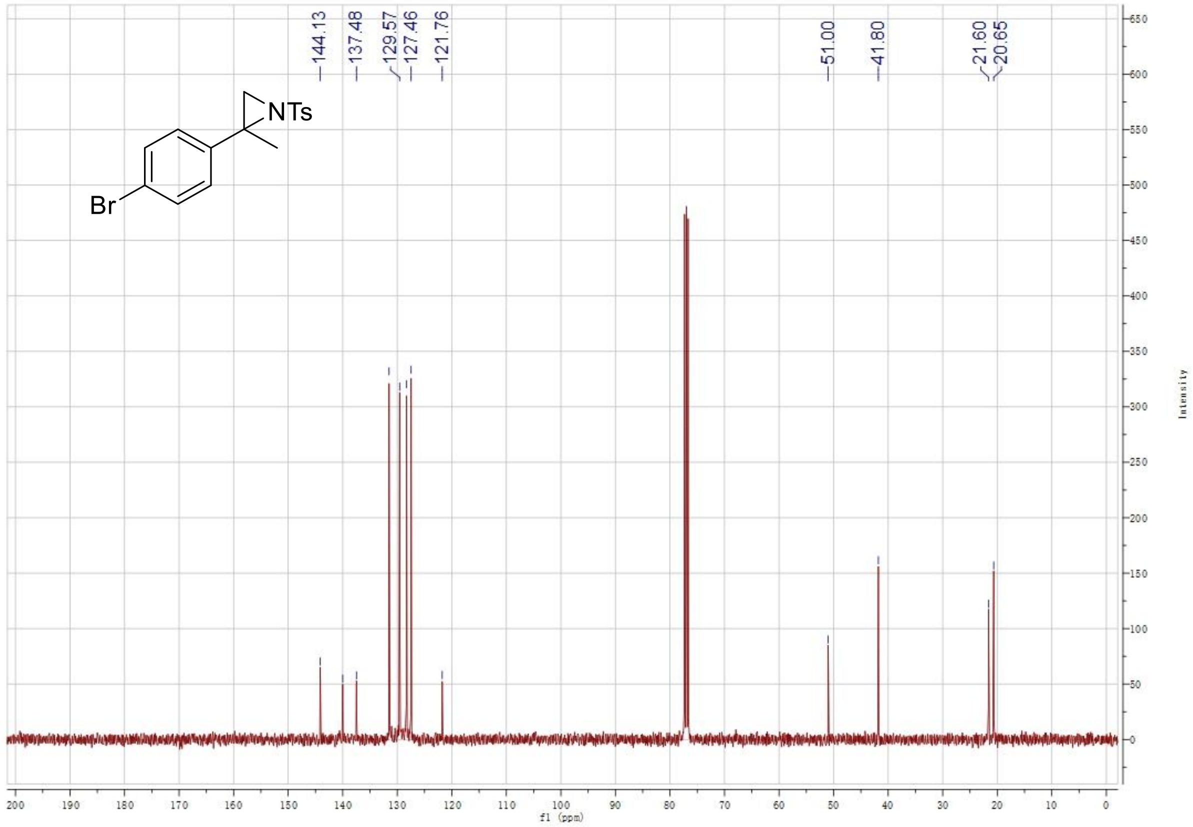

Supplementary Figure 42. *4-(2-Methyl-1-tosylaziridin-2-yl)benzonitrile (**11d**)*

^{1}H NMR (400 MHz, 293 K, $CDCl_3$)

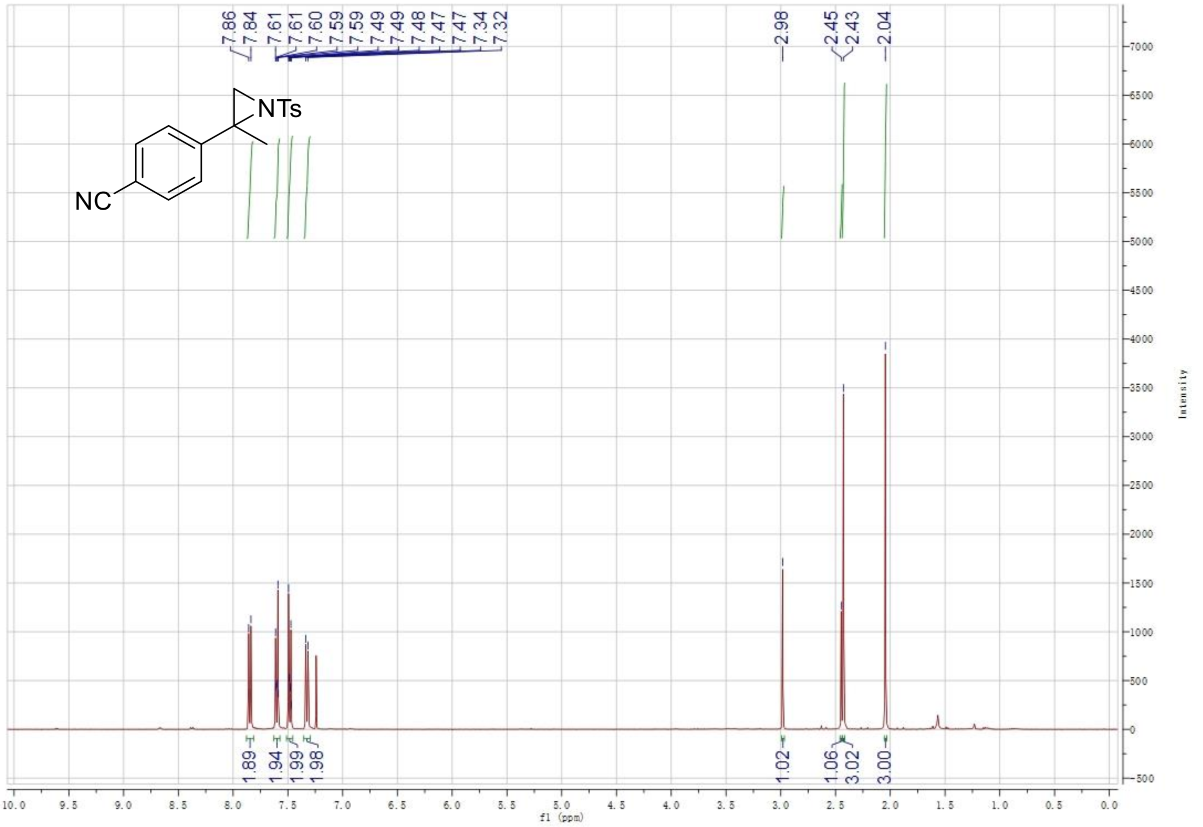

^{13}C NMR (101 MHz, 293 K, $CDCl_3$)

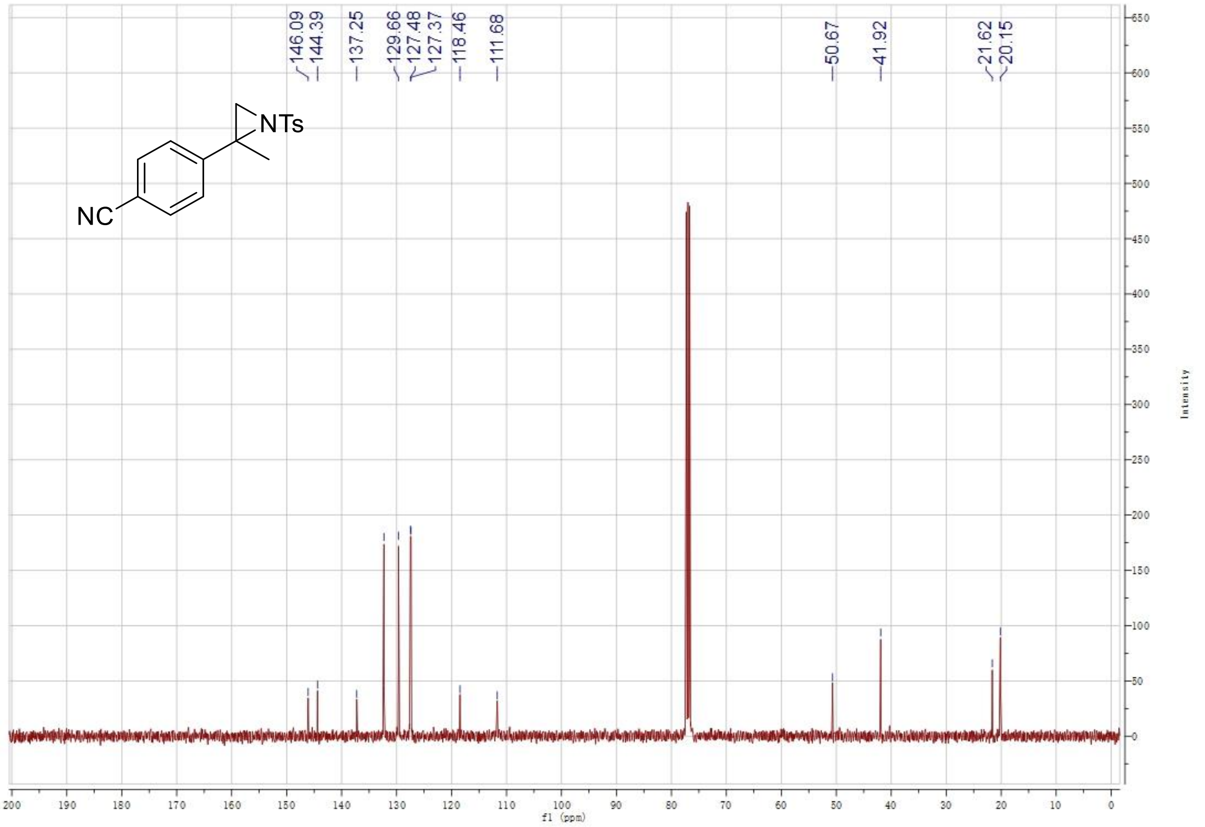

Supplementary Figure 43. *2-(3-Fluorophenyl)-2-methyl-1-tosylaziridine (**11e**)*

^{1}H NMR (400 MHz, 293 K, $CDCl_3$)

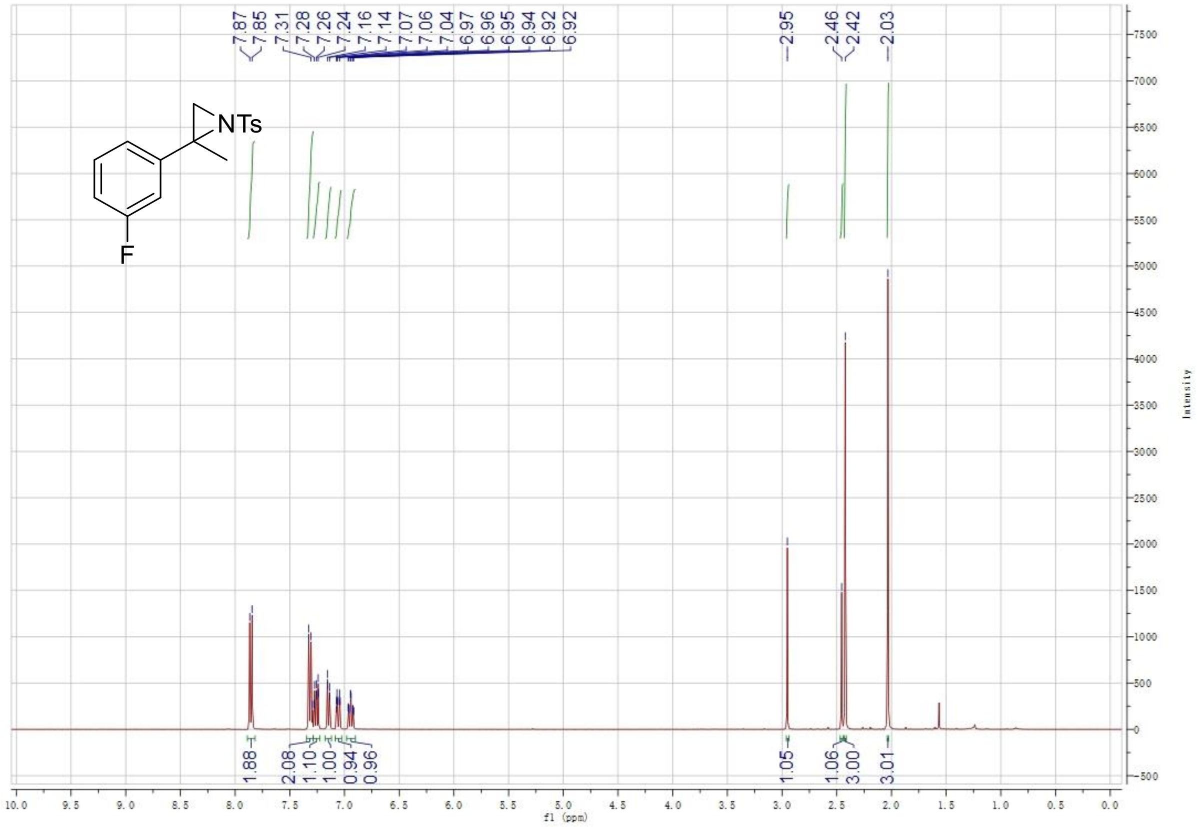

^{13}C NMR (101 MHz, 293 K, $CDCl_3$)

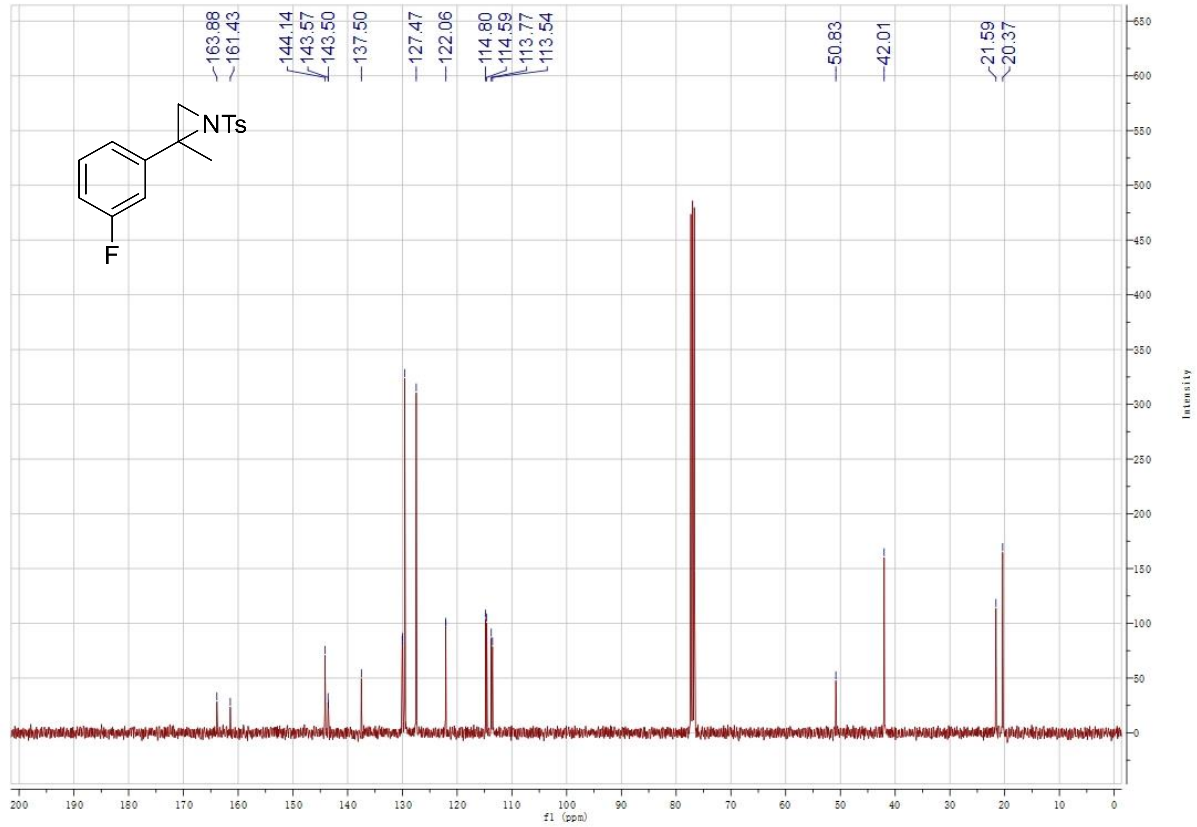

^{19}F NMR (376 MHz, 293 K, $CDCl_3$)

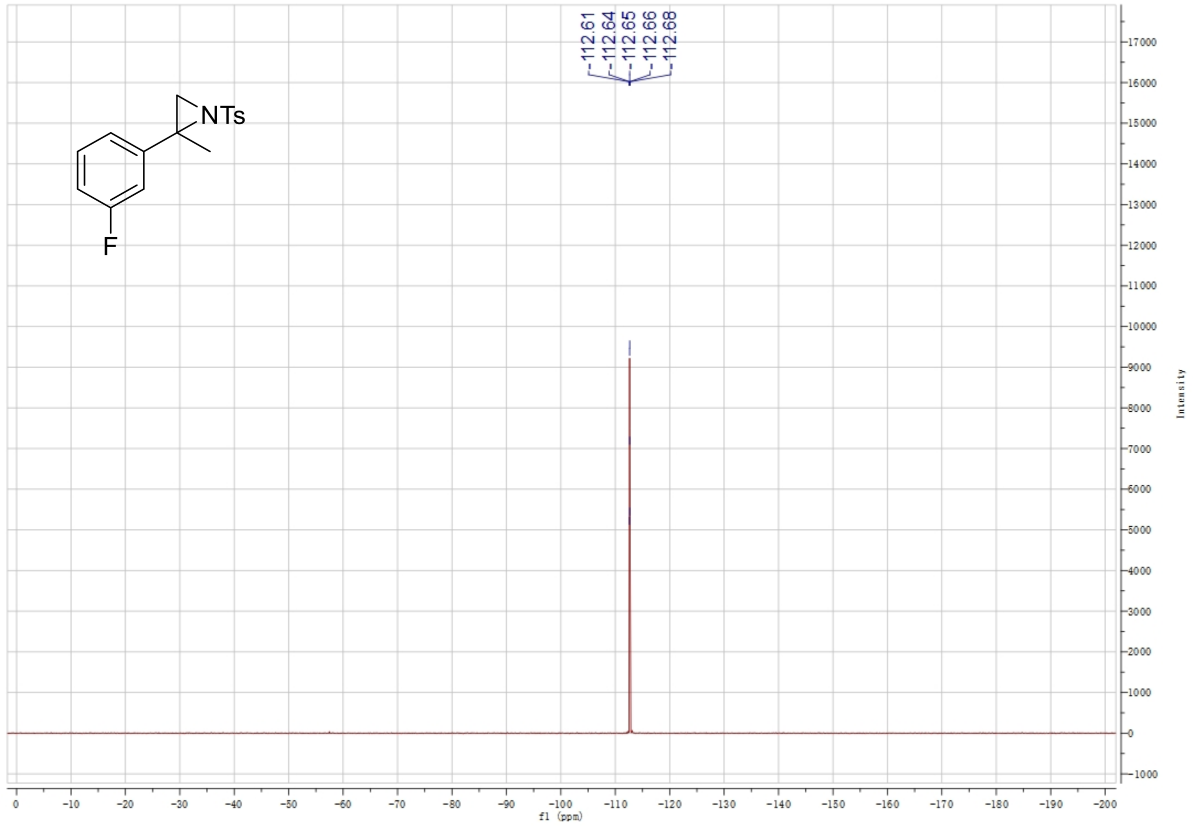

Supplementary Figure 44. *2-Methyl-2-(m-tolyl)-1-tosylaziridine (**11f**)*

^{1}H NMR (400 MHz, 293 K, $CDCl_3$)

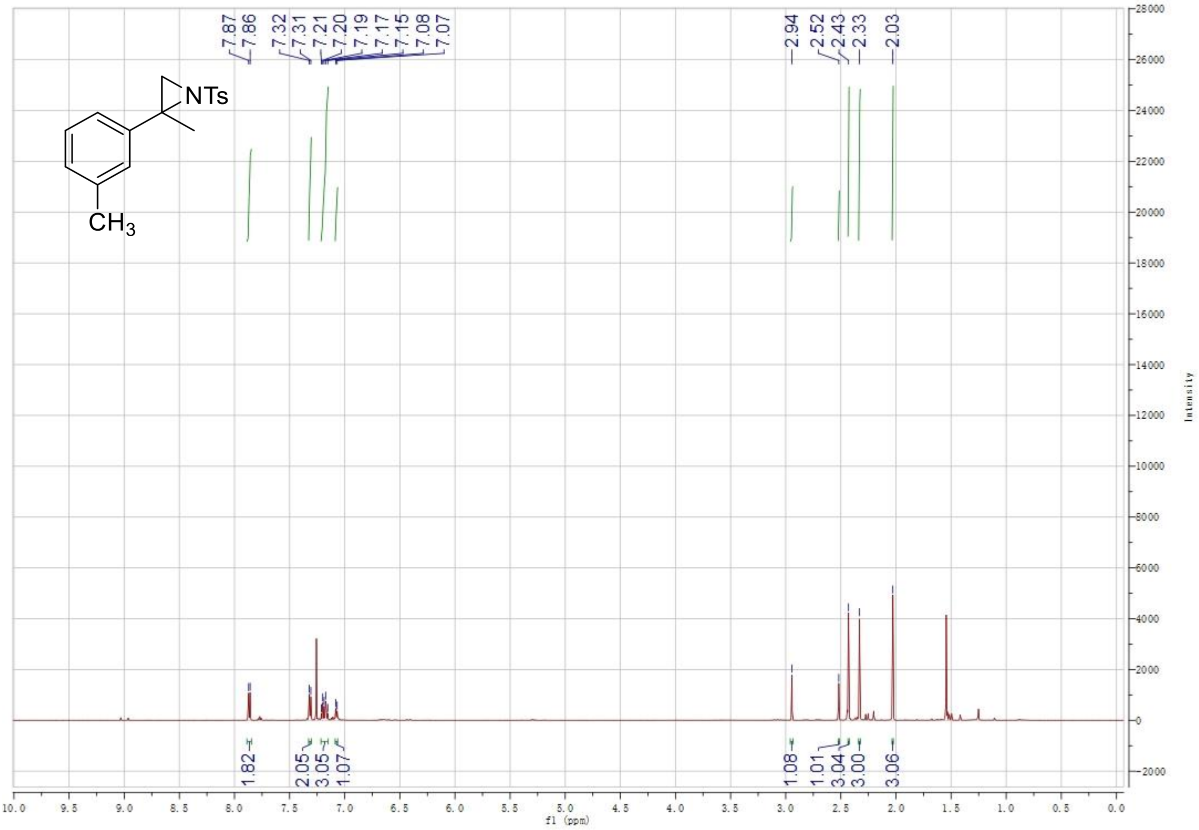

^{13}C NMR (101 MHz, 293 K, $CDCl_3$)

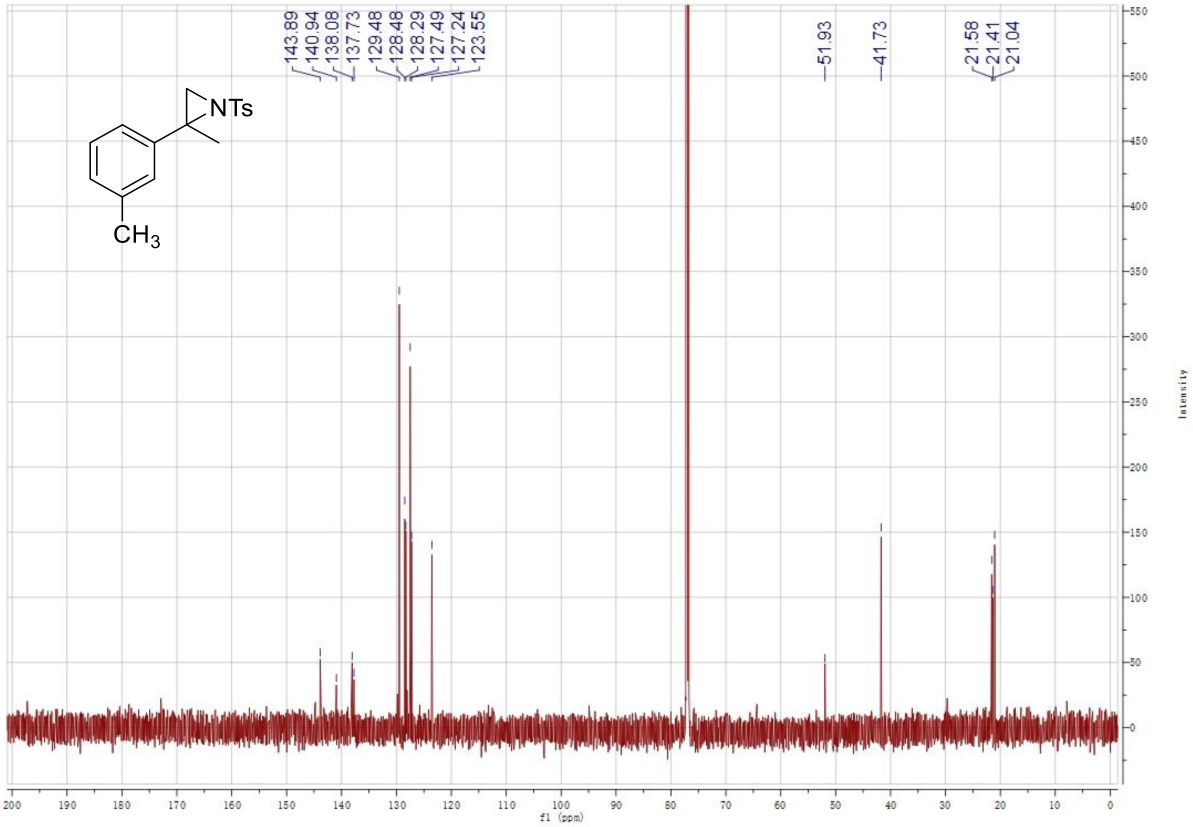

Supplementary Figure 45. *2-(3-Methoxyphenyl)-2-methyl-1-tosylaziridine (**11g**)*

^{1}H NMR (400 MHz, 293 K, $CDCl_3$)

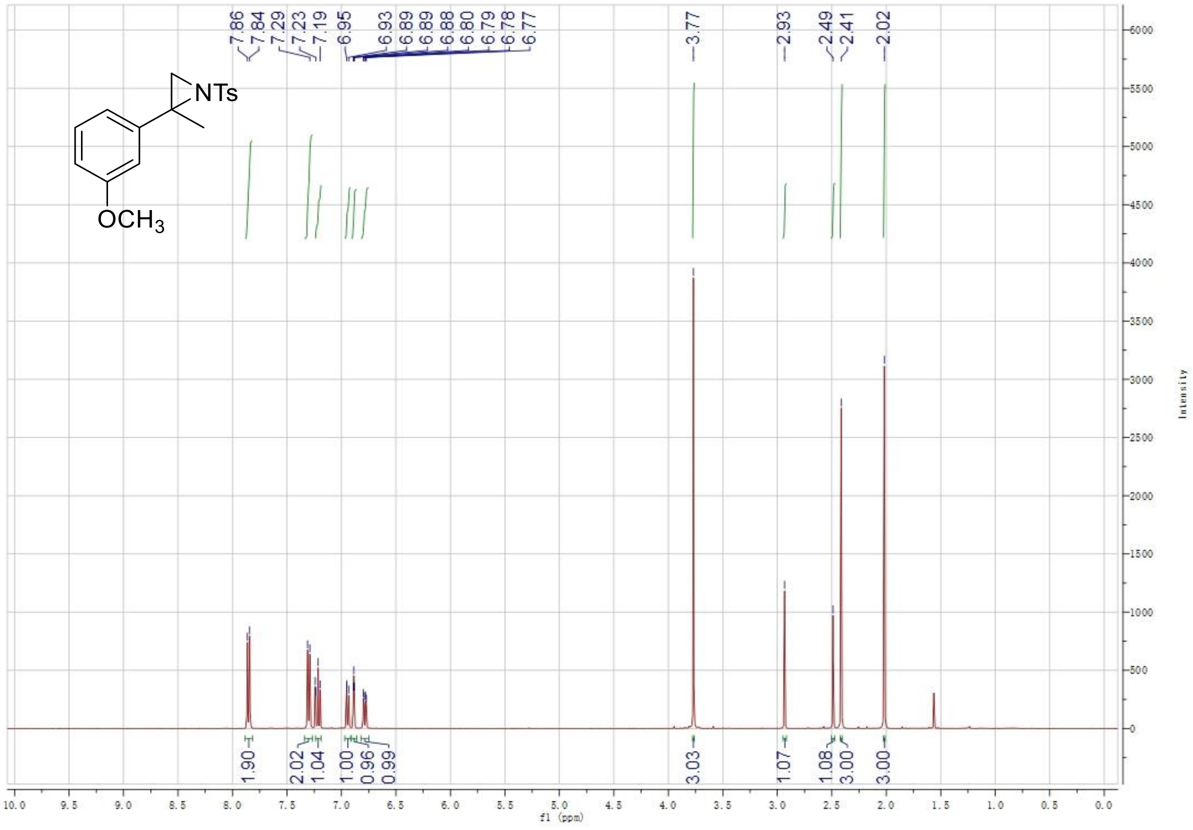

^{13}C NMR (101 MHz, 293 K, $CDCl_3$)

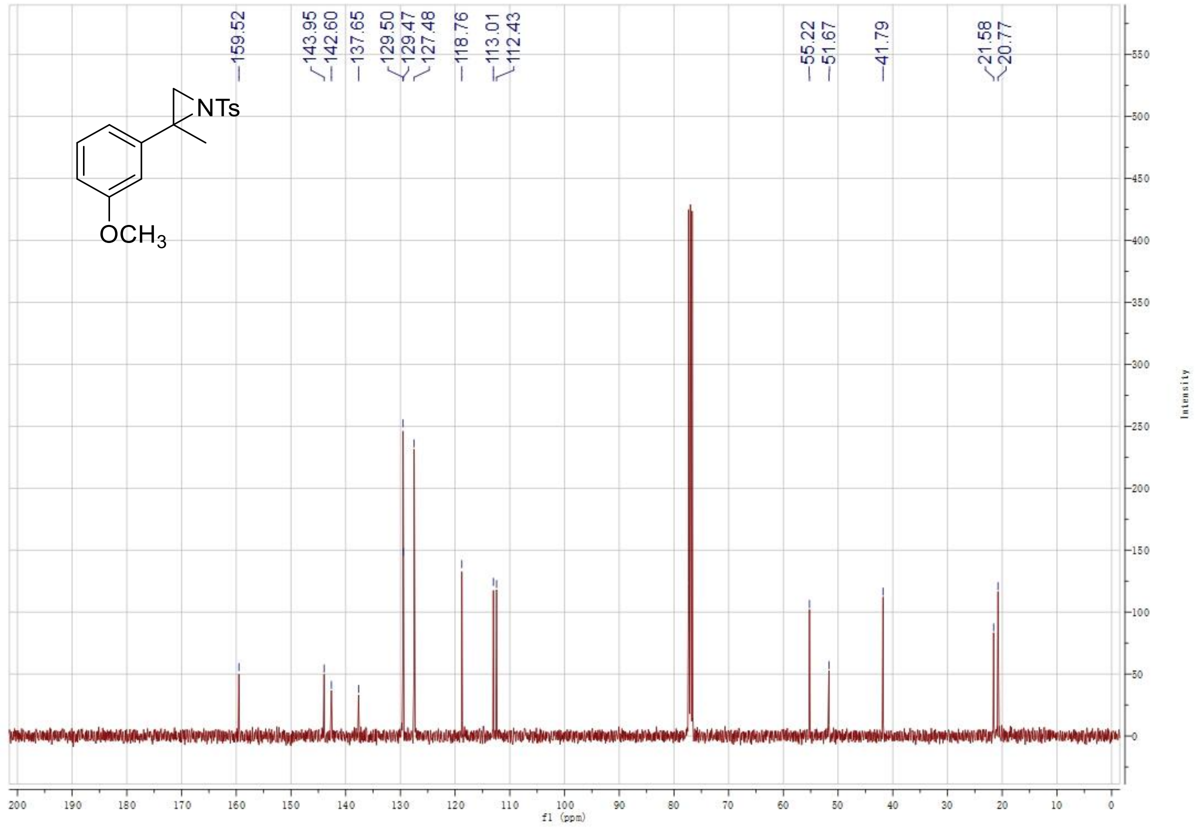

Supplementary Figure 46. *2-Methyl-1-tosyl-2-(3-(trifluoromethyl)phenyl)aziridine (**11h**)*

^{1}H NMR (400 MHz, 293 K, $CDCl_3$)

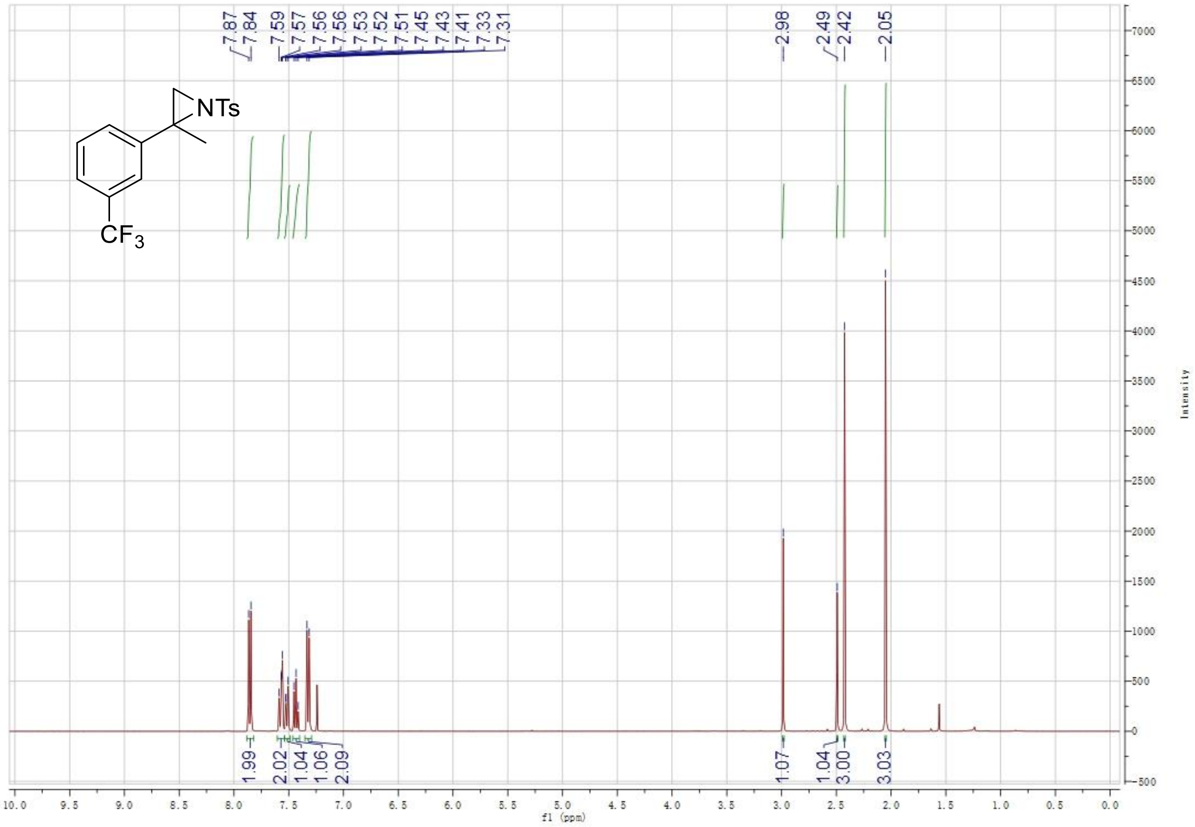

^{13}C NMR (101 MHz, 293 K, $CDCl_3$)

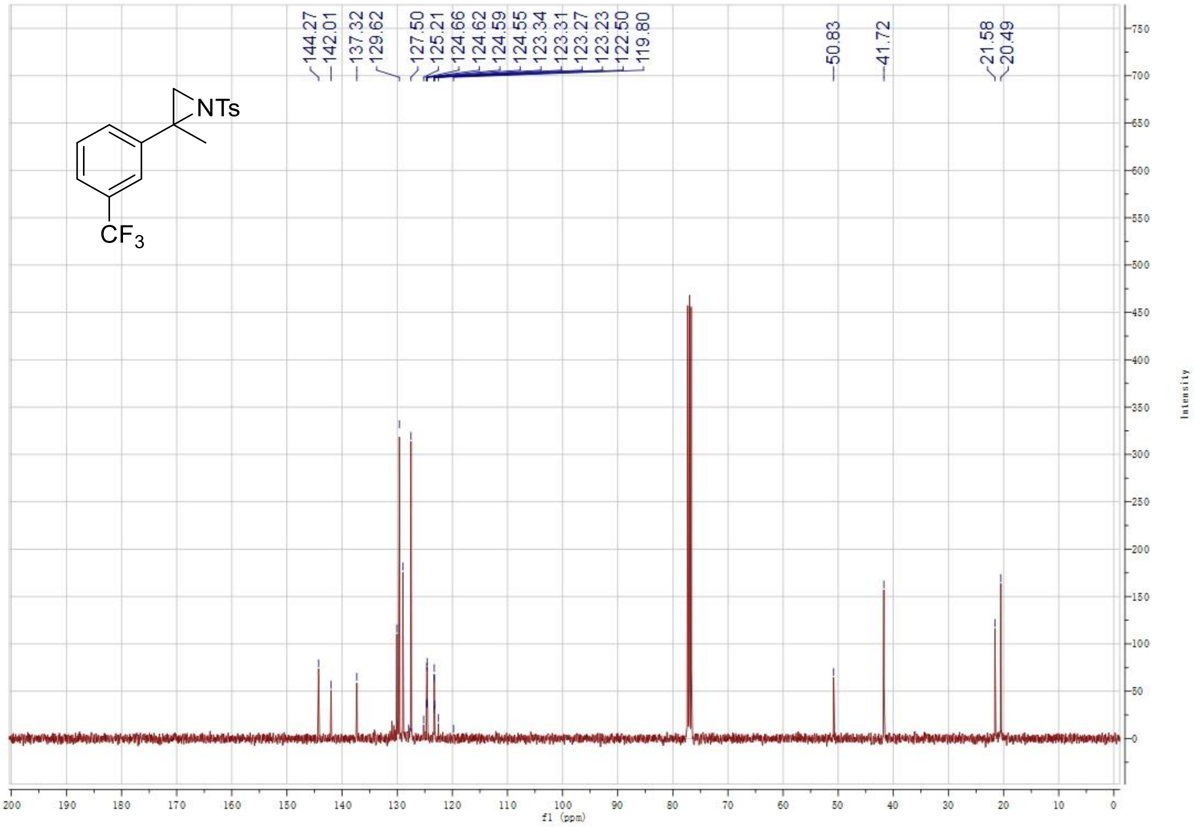

^{19}F NMR (376 MHz, 293 K, $CDCl_3$)

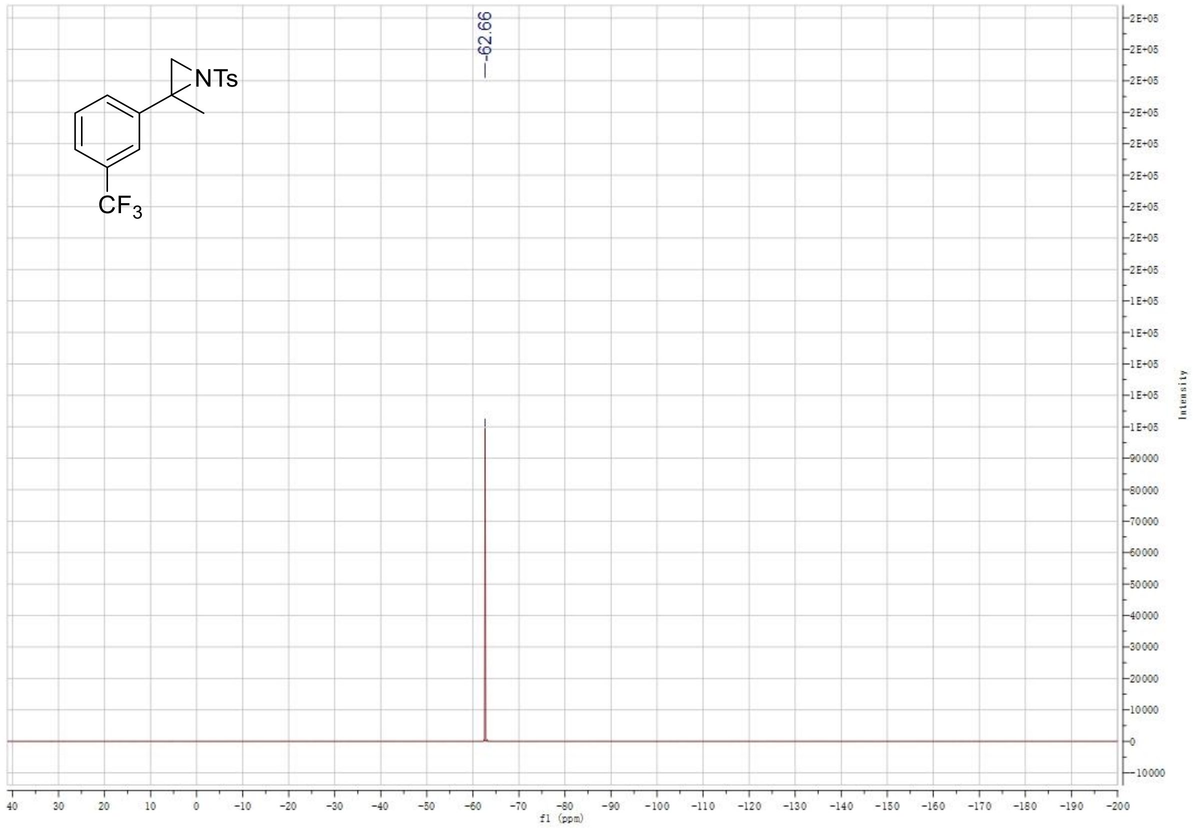

Supplementary Figure 47. *2-(2-Bromophenyl)-2-methyl-1-tosylaziridine (**11i**)*

^{1}H NMR (400 MHz, 293 K, $CDCl_3$)

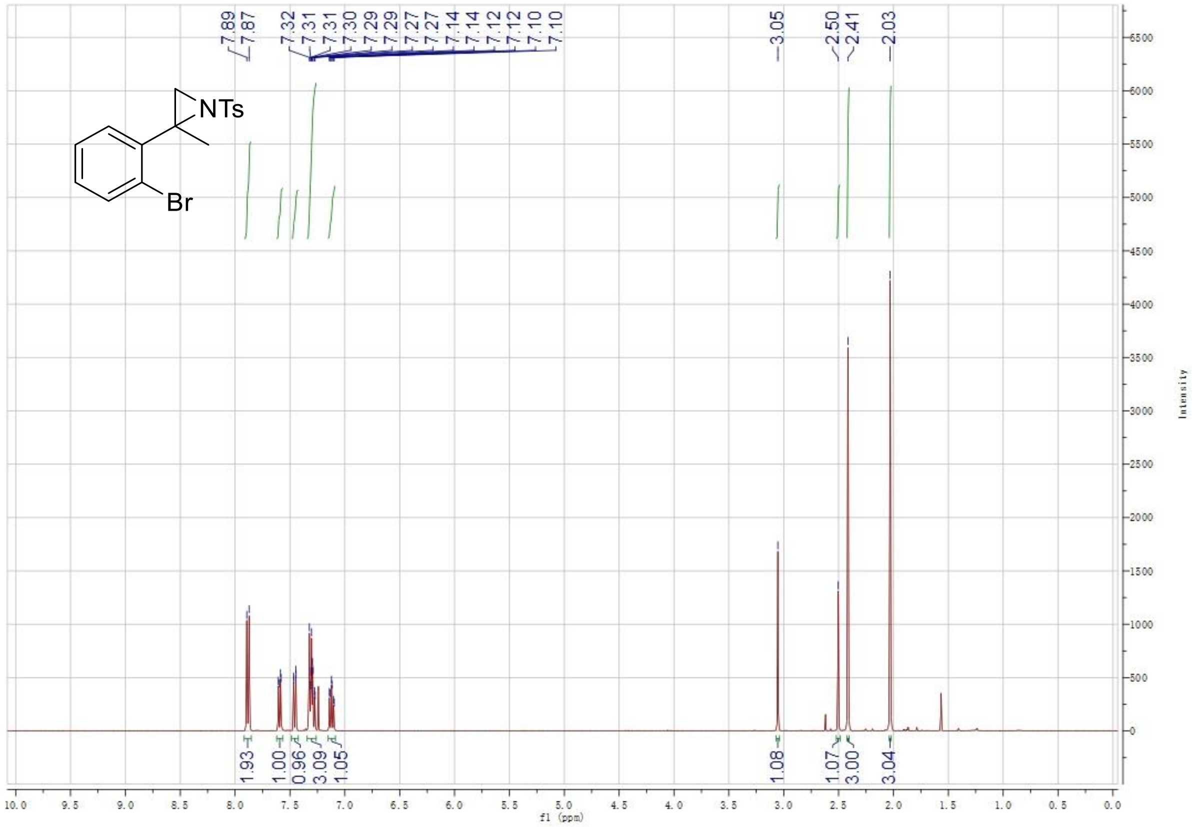

^{13}C NMR (101 MHz, 293 K, $CDCl_3$)

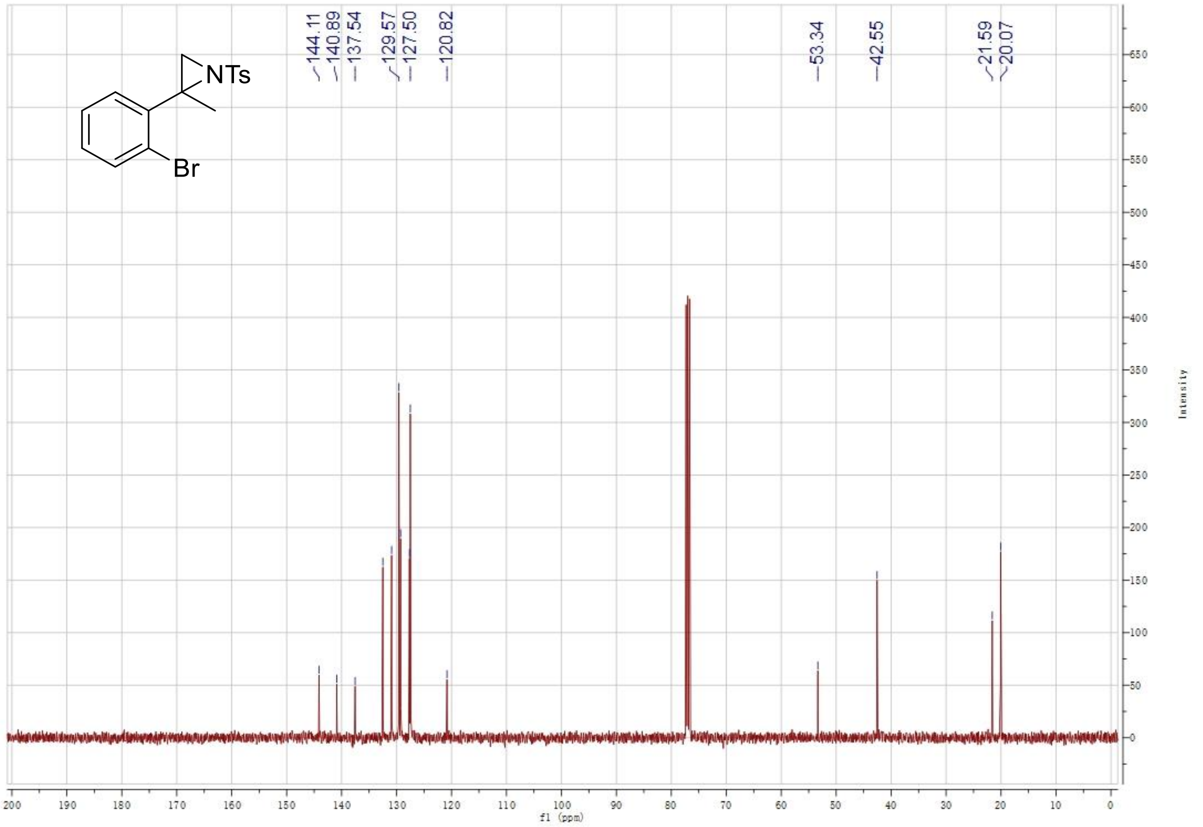

Supplementary Figure 48. *2-Methyl-2-(o-tolyl)-1-tosylaziridine* (***11j***)

^{1}H NMR (400 MHz, 293 K, $CDCl_3$)

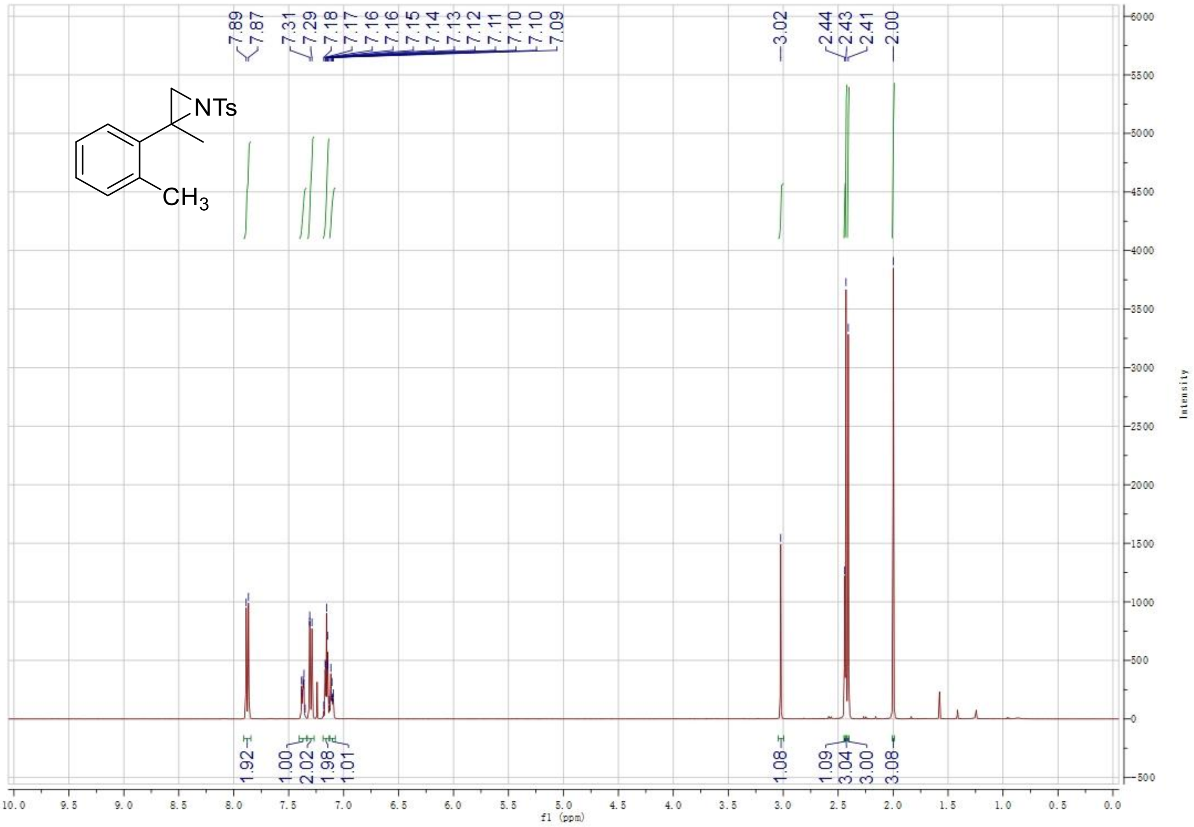

^{13}C NMR (101 MHz, 293 K, $CDCl_3$)

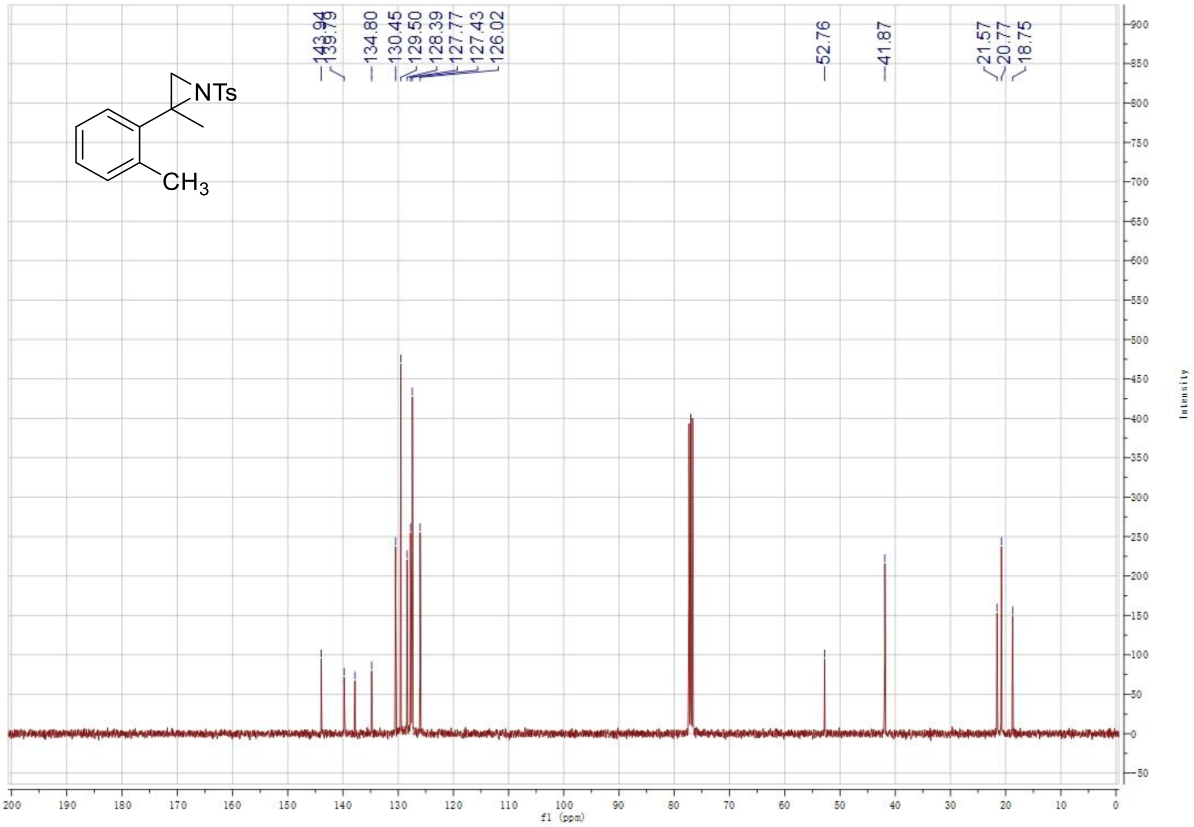

Supplementary Figure 49. *2-(2,4-Dimethylphenyl)-2-methyl-1-tosylaziridine (**11k**)*

^{1}H NMR (400 MHz, 293 K, $CDCl_3$)

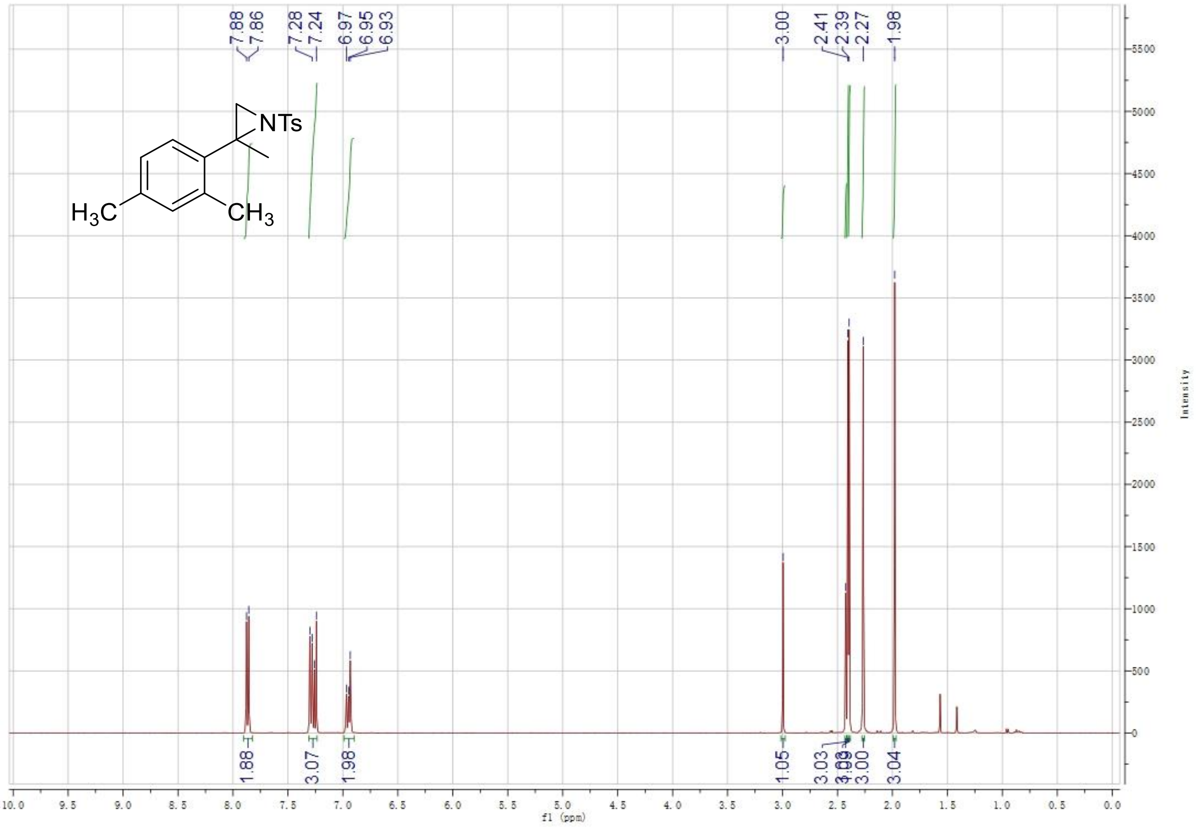

^{13}C NMR (101 MHz, 293 K, $CDCl_3$)

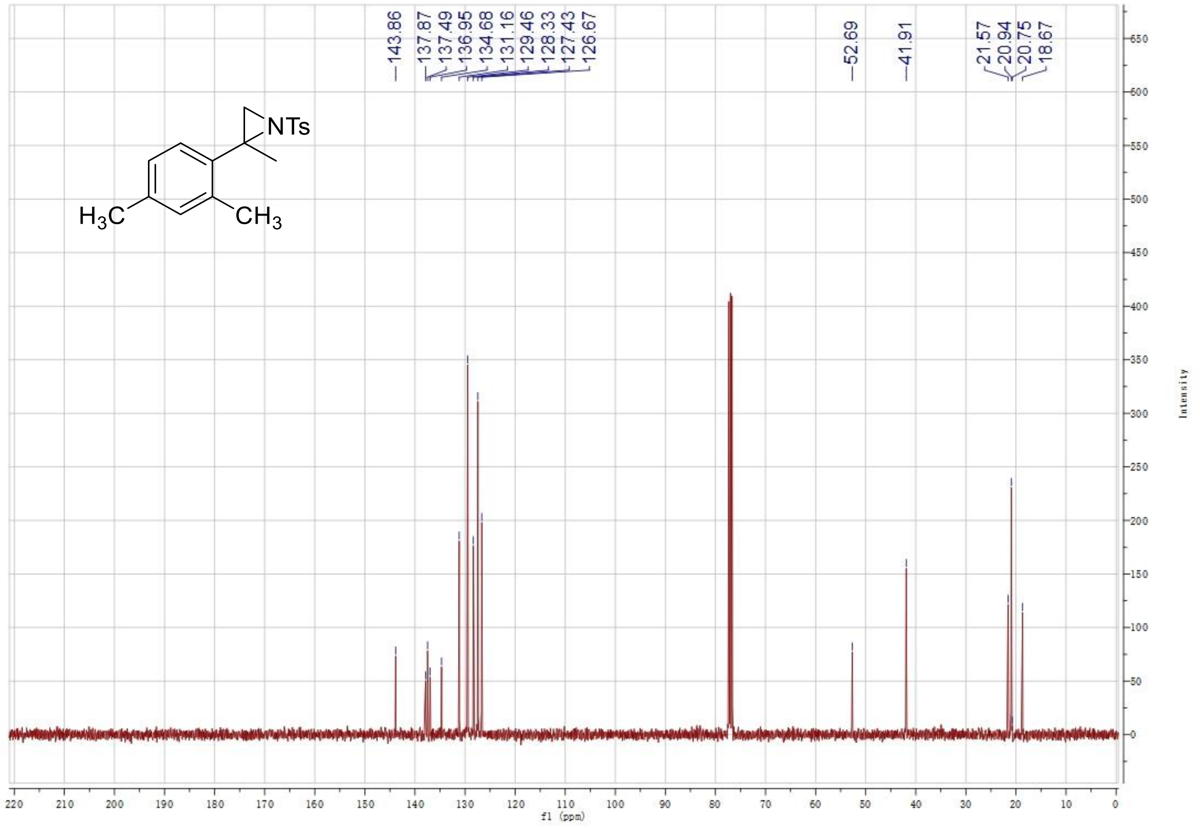

Supplementary Figure 50. *2-Methyl-2-phenyl-1-(phenylsulfonyl)aziridine (**11l**)*

^{1}H NMR (400 MHz, 293 K, $CDCl_3$)

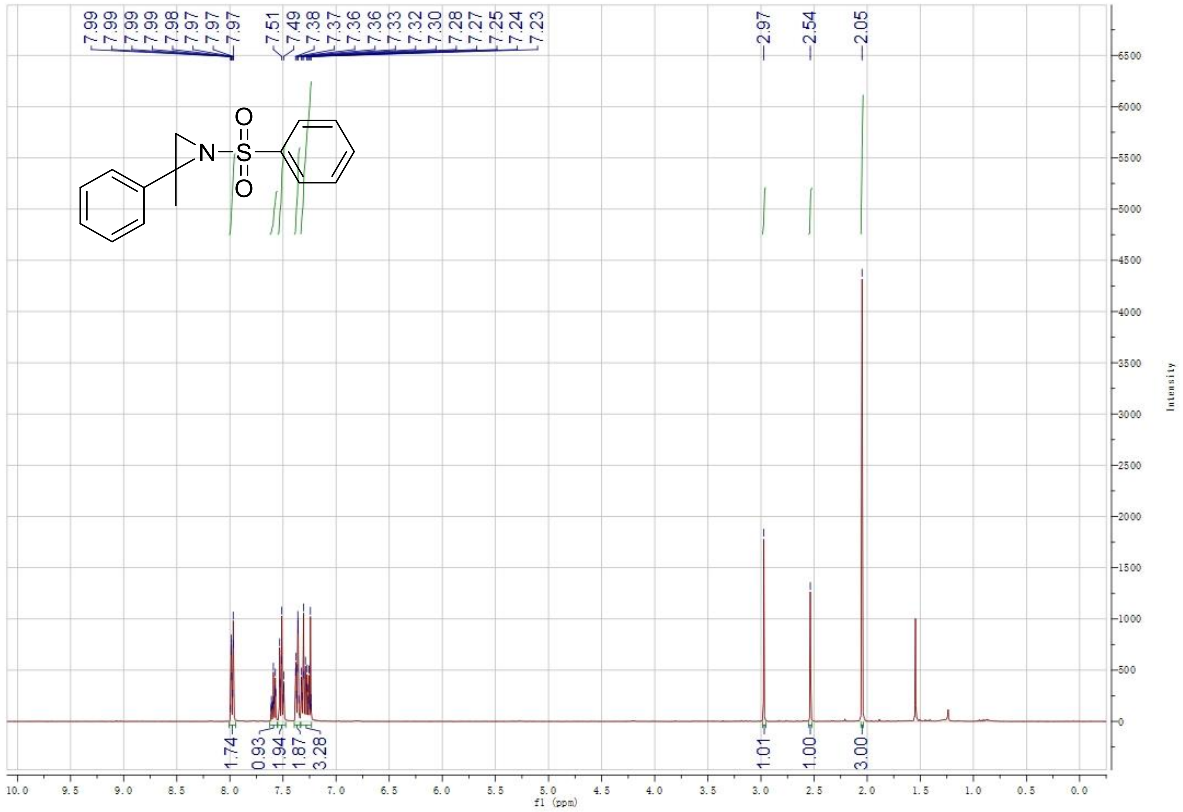

^{13}C NMR (101 MHz, 293 K, $CDCl_3$)

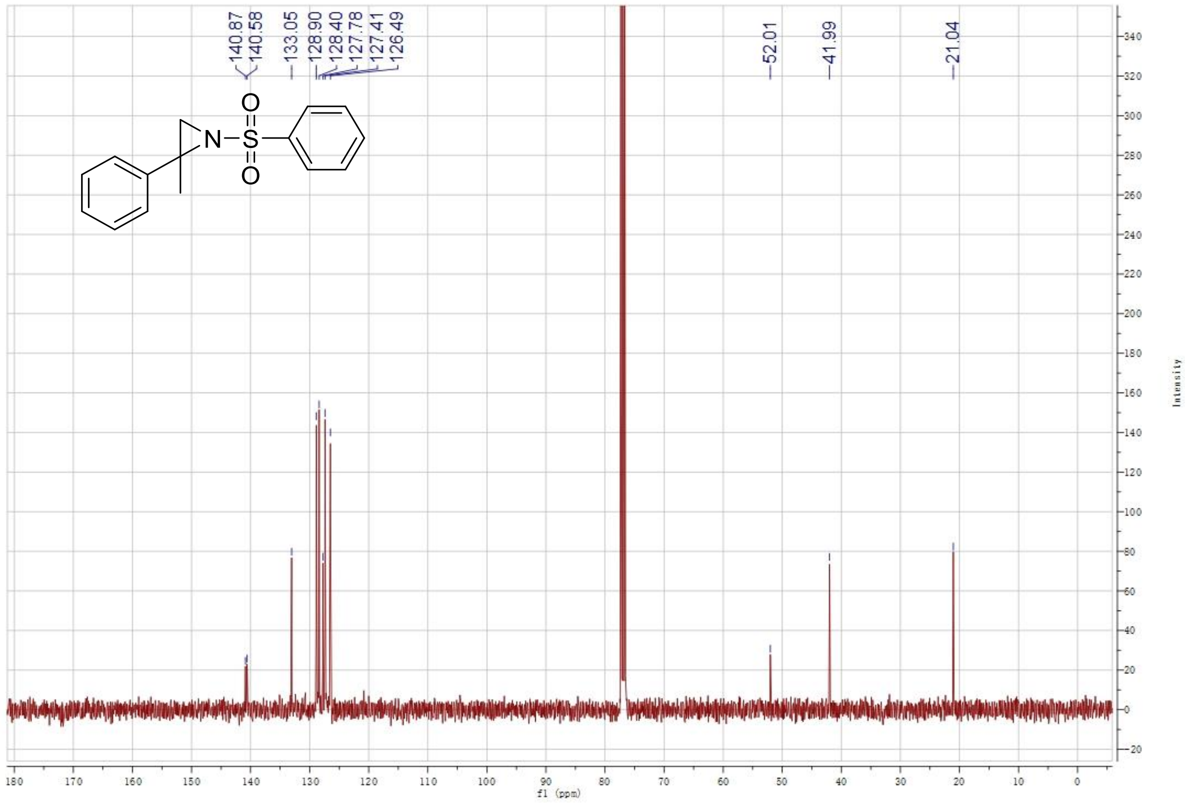

Supplementary Figure 51. *1-((4-Chlorophenyl)sulfonyl)-2-methyl-2-phenylaziridine* (***11m***)

^{1}H NMR (400 MHz, 293 K, $CDCl_3$)

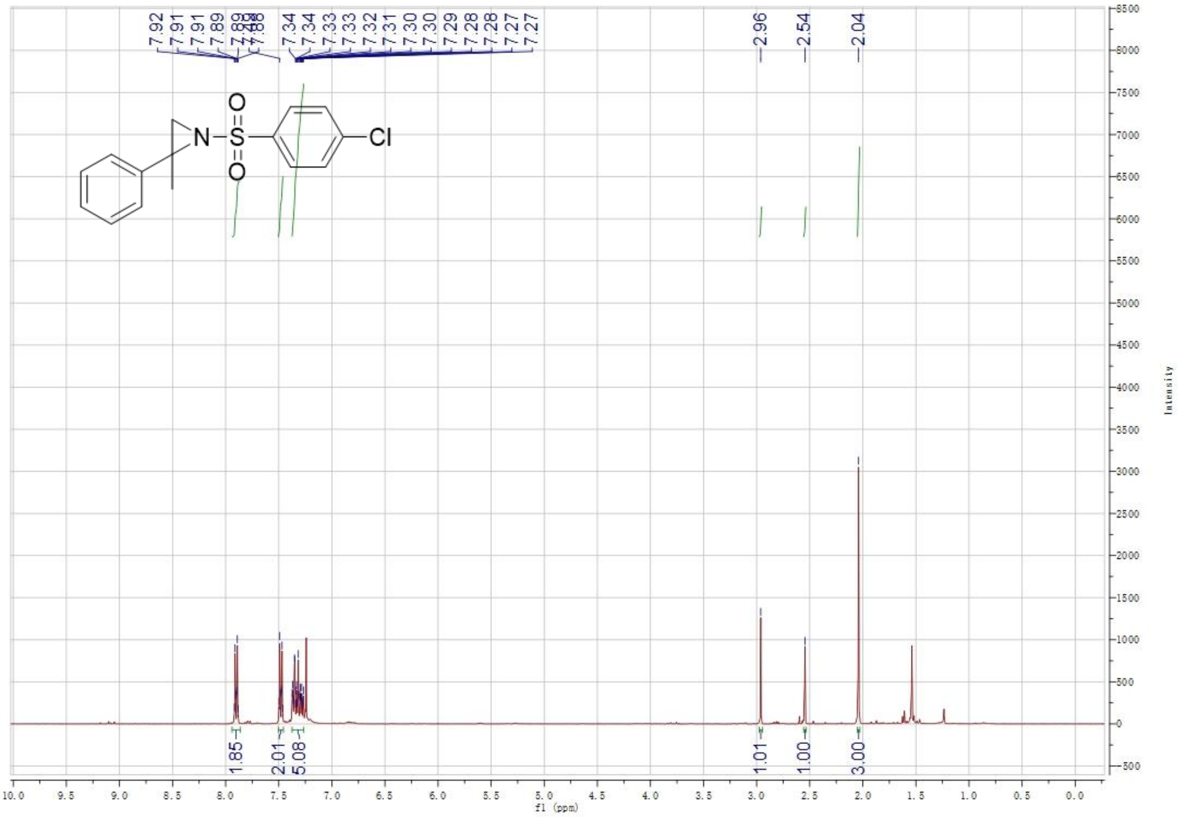

^{13}C NMR (101 MHz, 293 K, $CDCl_3$)

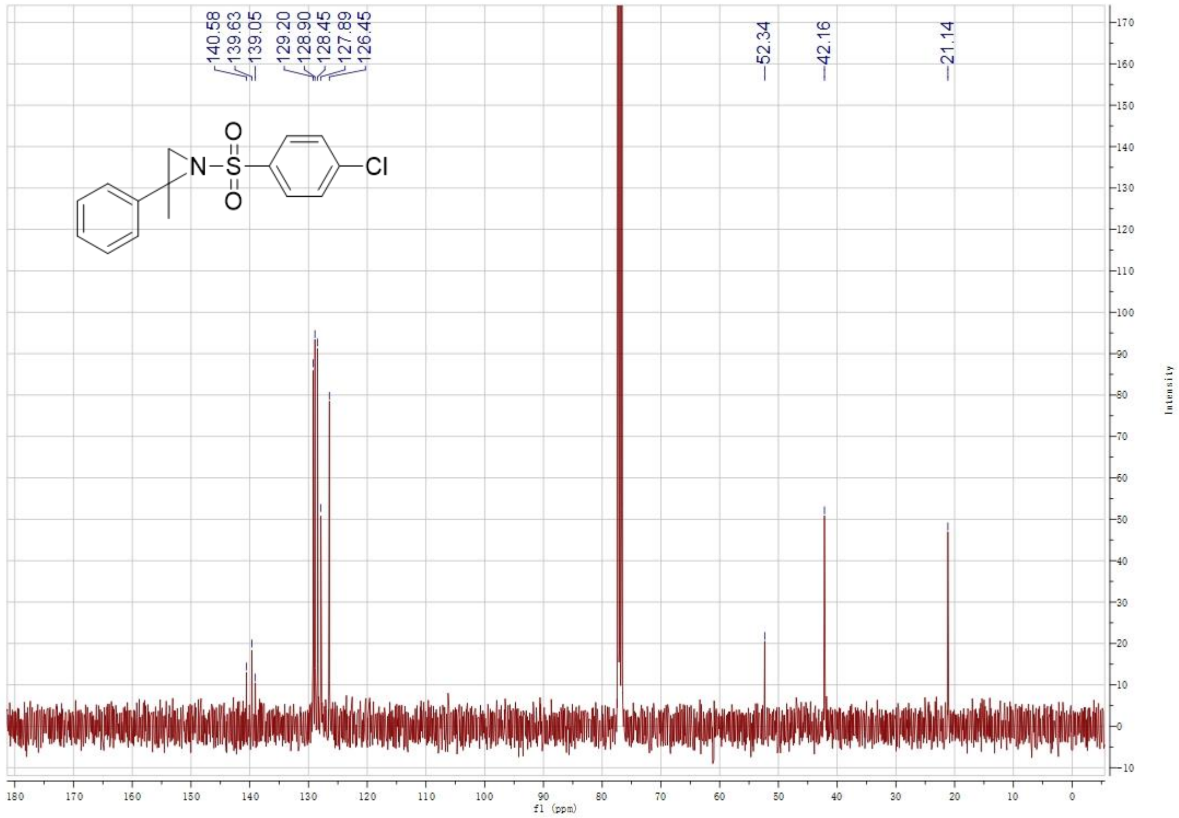

Supplementary Figure 52. *2-Methyl-1-(naphthalen-2-ylsulfonyl)-2-phenylaziridine (**11n**)*

^{1}H NMR (600 MHz, 293 K, $CDCl_3$)

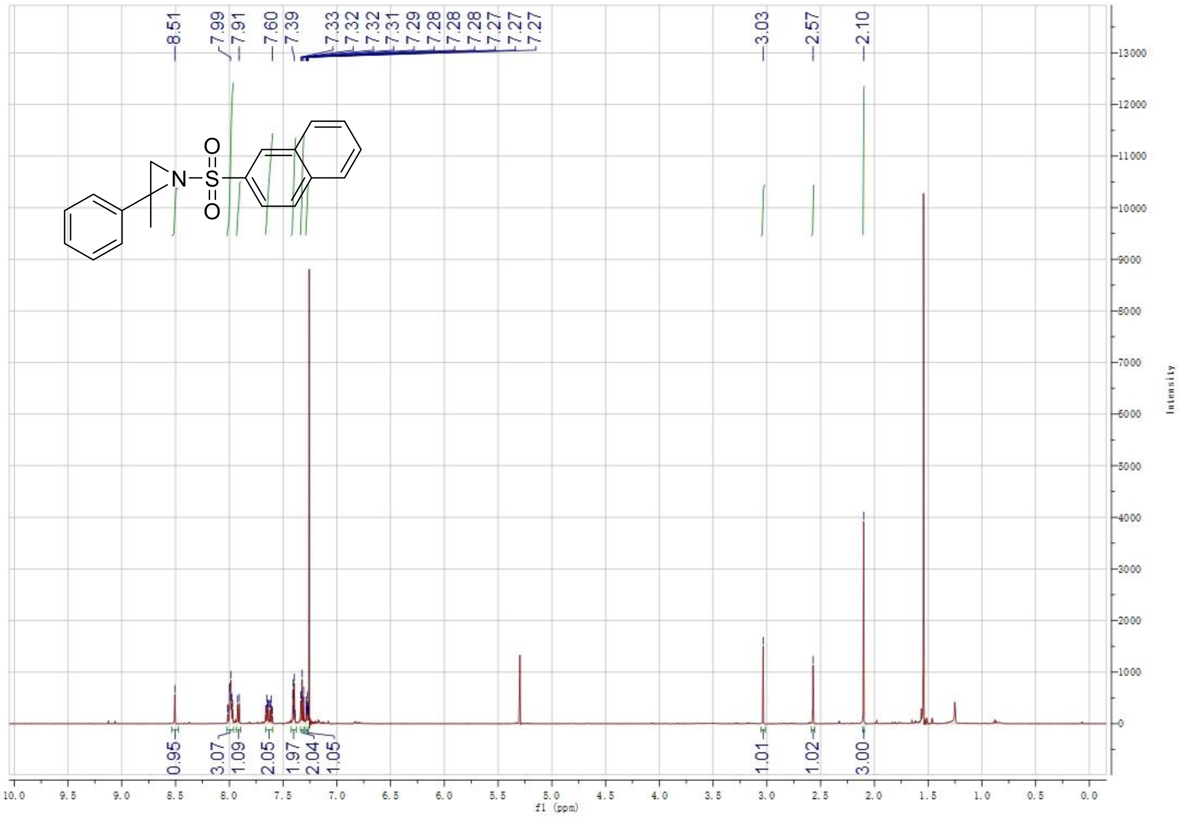

^{13}C NMR (151 MHz, 293 K, $CDCl_3$)

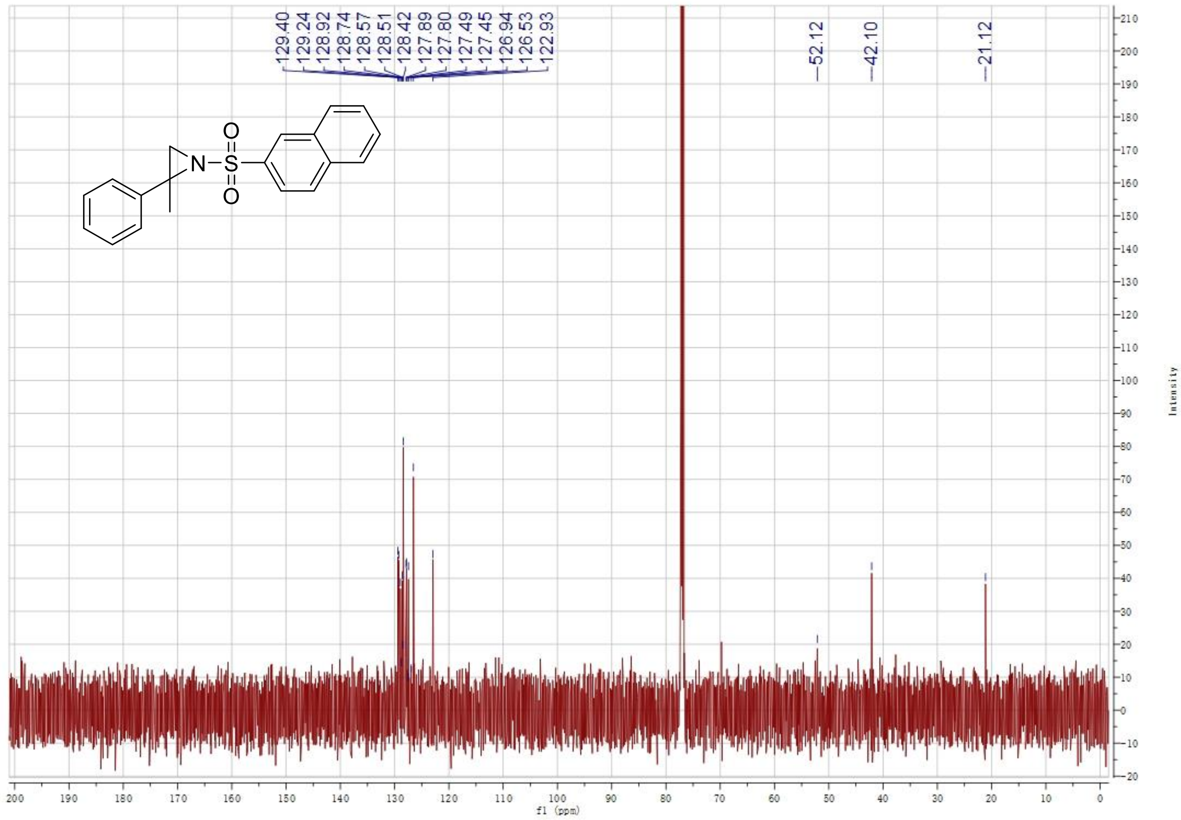

Supplementary Figure 53. *2-Methyl-1-(methylsulfonyl)-2-phenylaziridine (**11o**)*

^{1}H NMR (400 MHz, 293 K, $CDCl_3$)

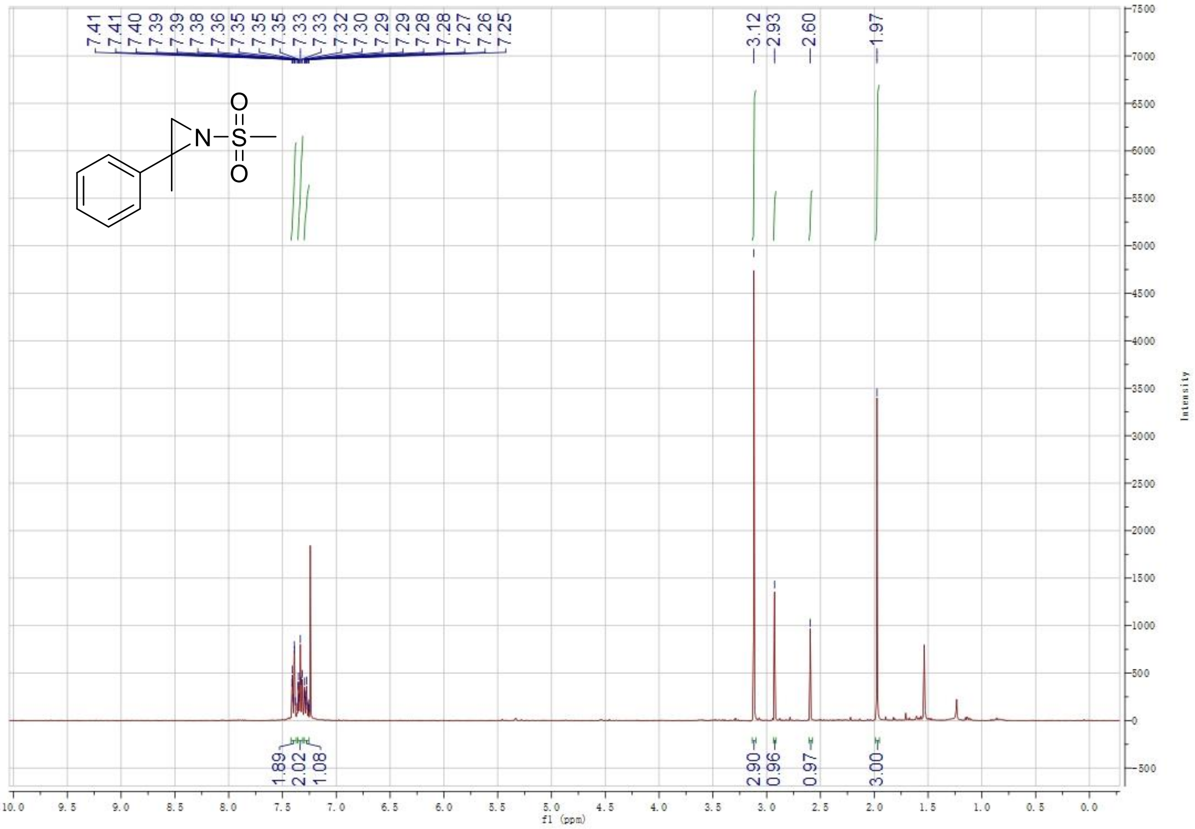

^{13}C NMR (101 MHz, 293 K, $CDCl_3$)

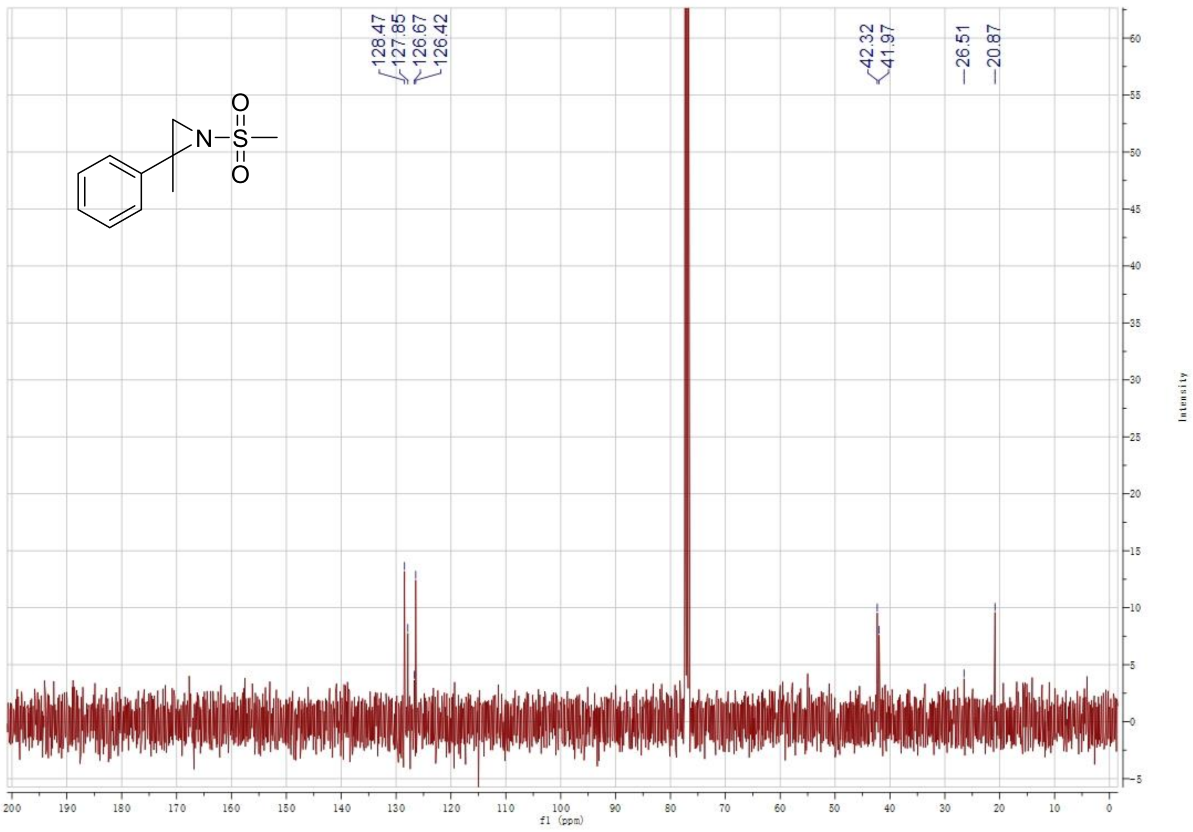

Supplementary Figure 54. *4-(2-Methyl-1-tosylaziridin-2-yl)pyridine (**11p**)*

^{1}H NMR (400 MHz, 293 K, $CDCl_3$)

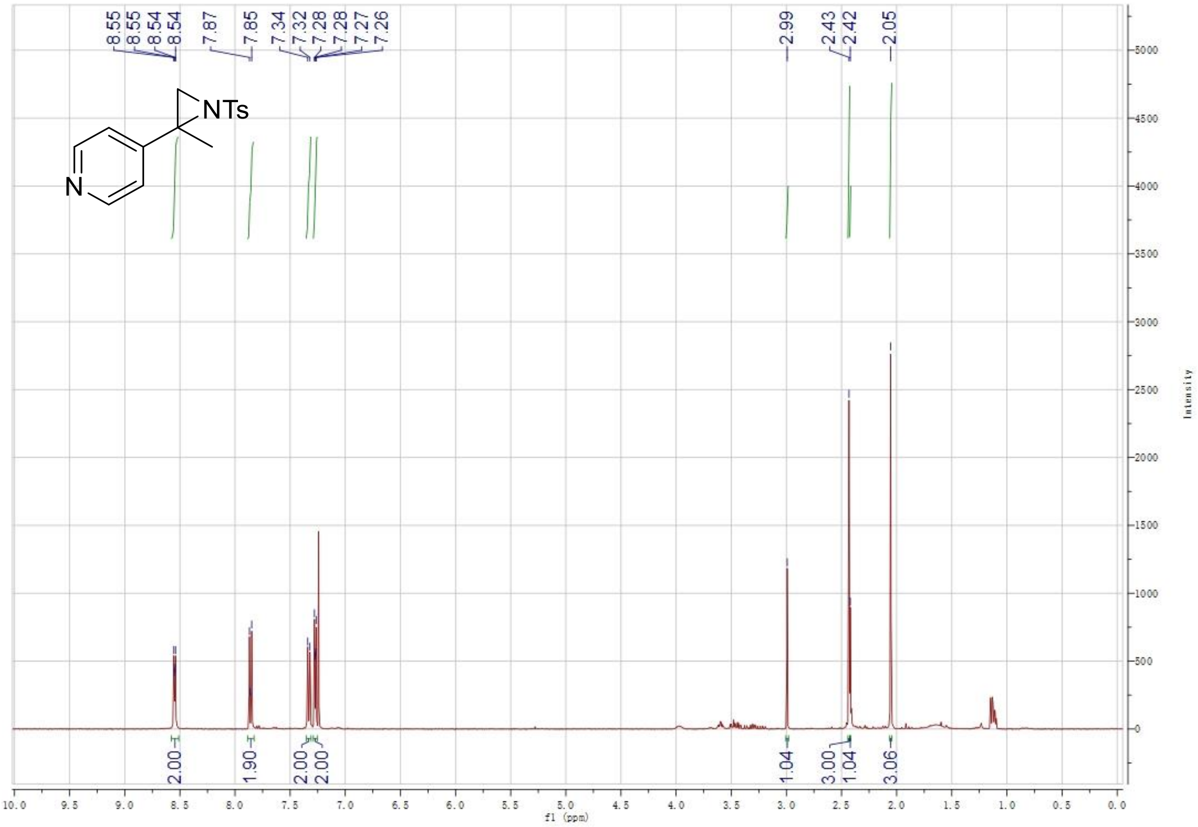

^{13}C NMR (101 MHz, 293 K, $CDCl_3$)

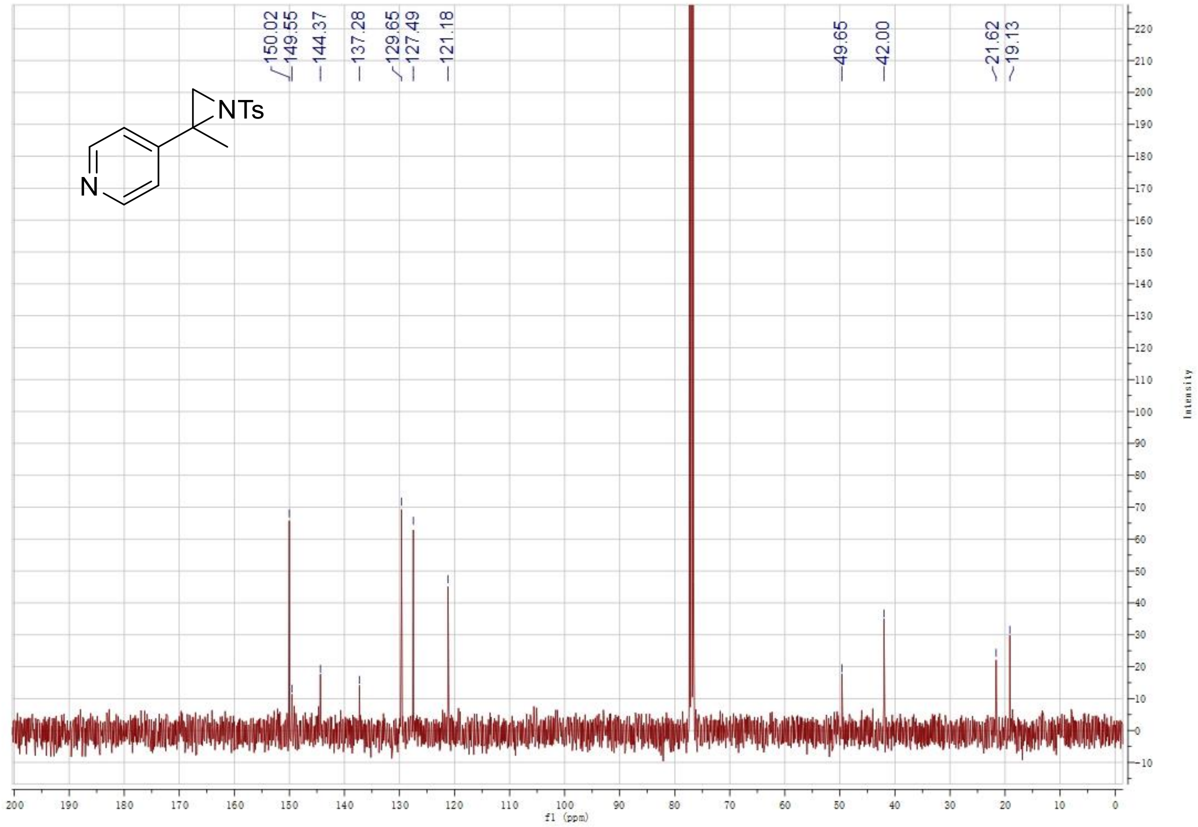

Supplementary Figure 55. *2-(2-Methyl-1-tosylaziridin-2-yl)pyridine (**11q**)*

^{1}H NMR (600 MHz, 293 K, $CDCl_3$)

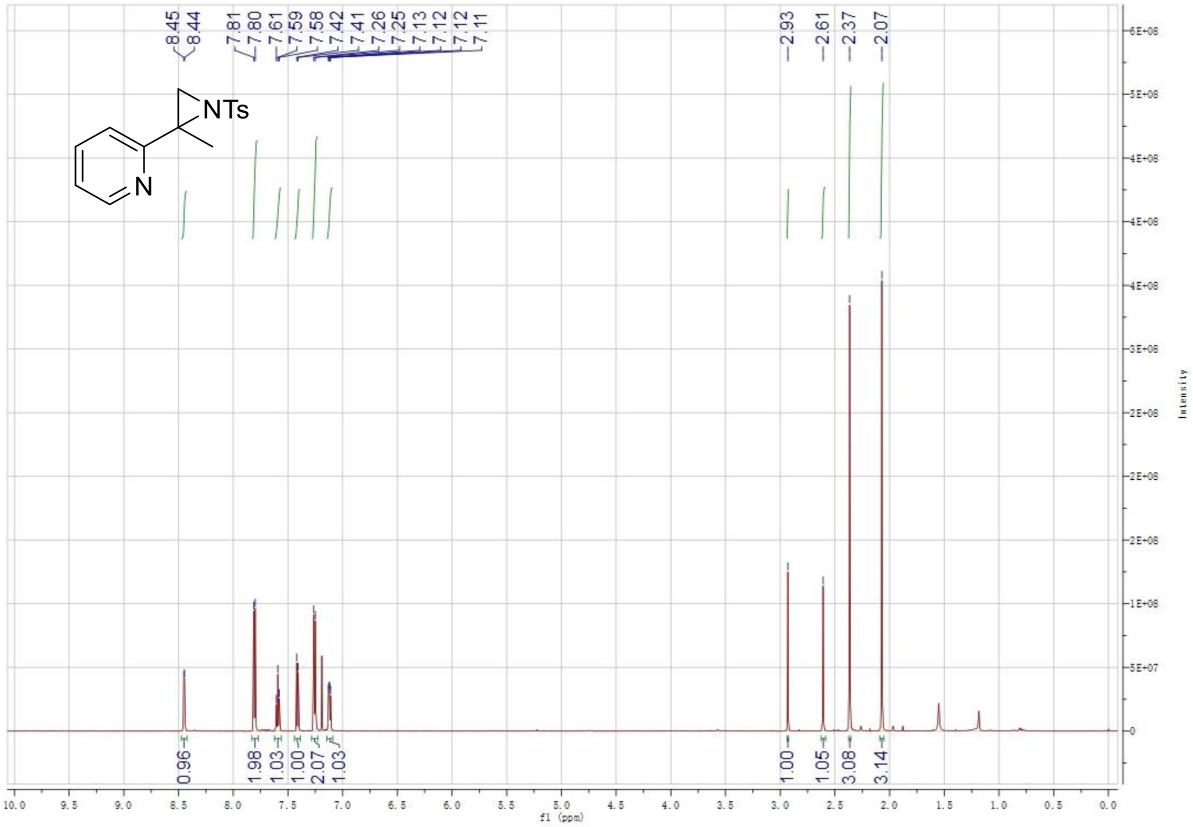

^{13}C NMR (151 MHz, 293 K, $CDCl_3$)

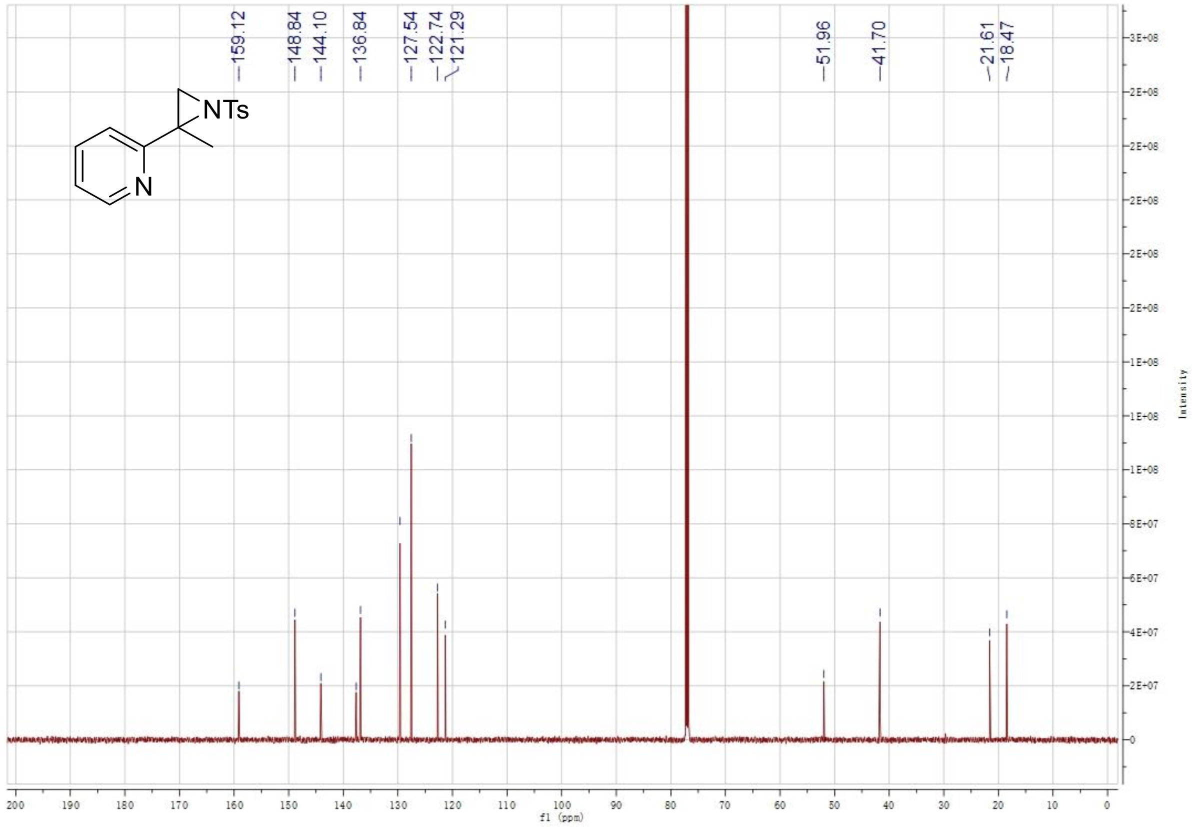

Supplementary Figure 56. *2-(4-Chlorophenyl)-2-ethyl-1-tosylaziridine* (***11r***)

^{1}H NMR (400 MHz, 293 K, $CDCl_3$)

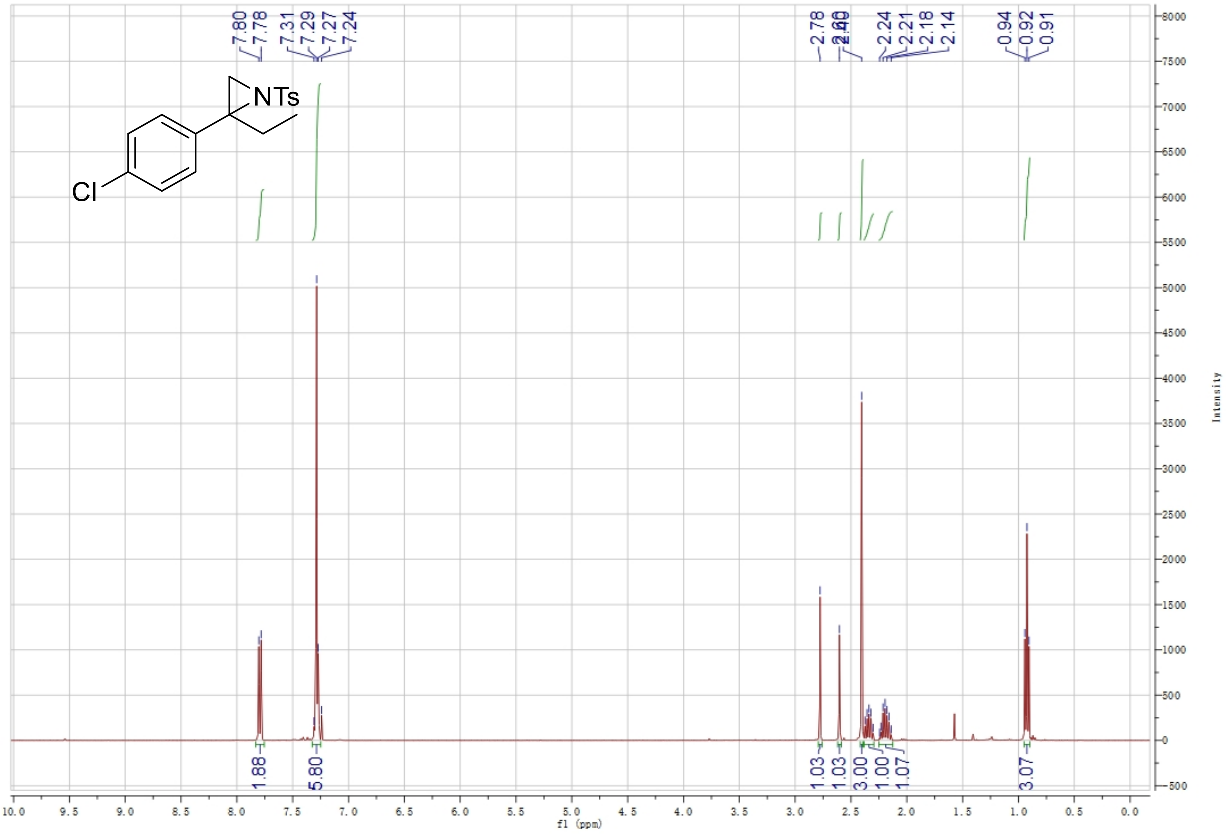

^{13}C NMR (101 MHz, 293 K, $CDCl_3$)

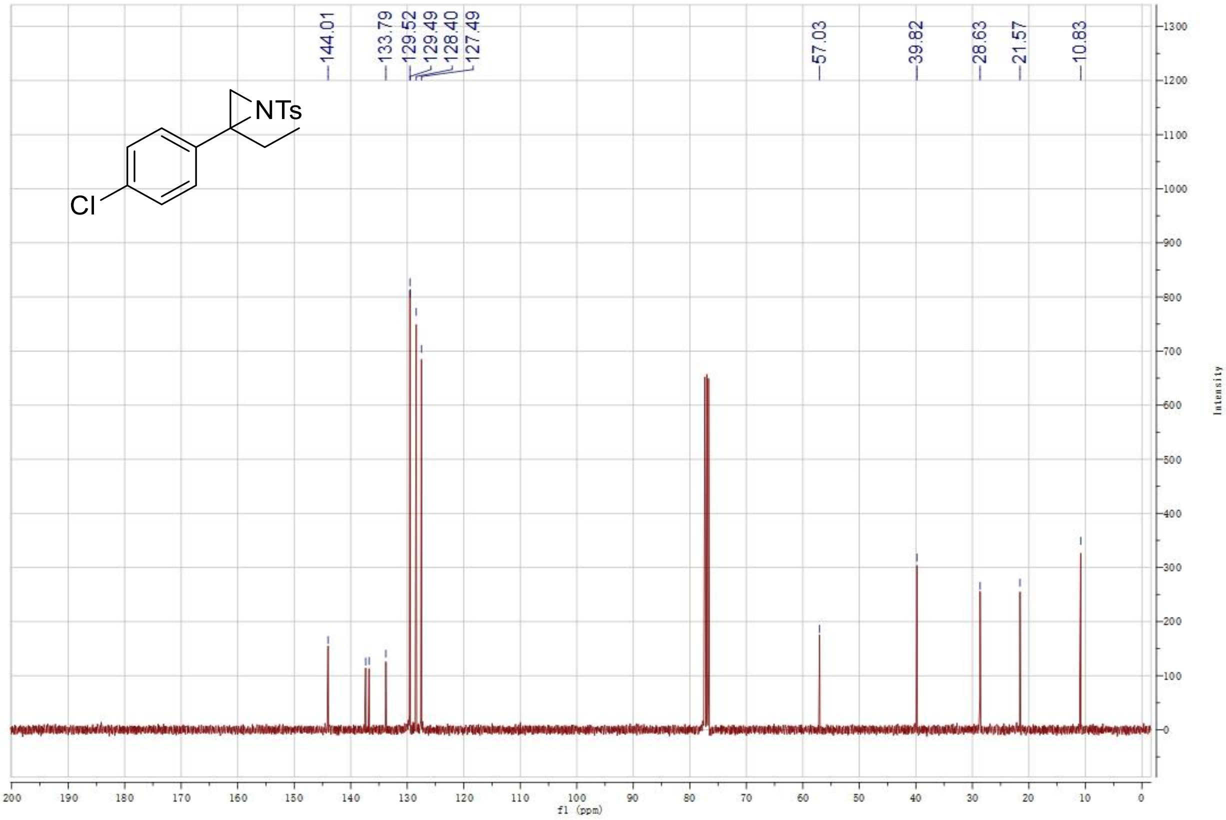

Supplementary Figure 57. *2-(tert-Butyl)-2-phenyl-1-tosylaziridine (**11s**)*

^{1}H NMR (400 MHz, 293 K, $CDCl_3$)

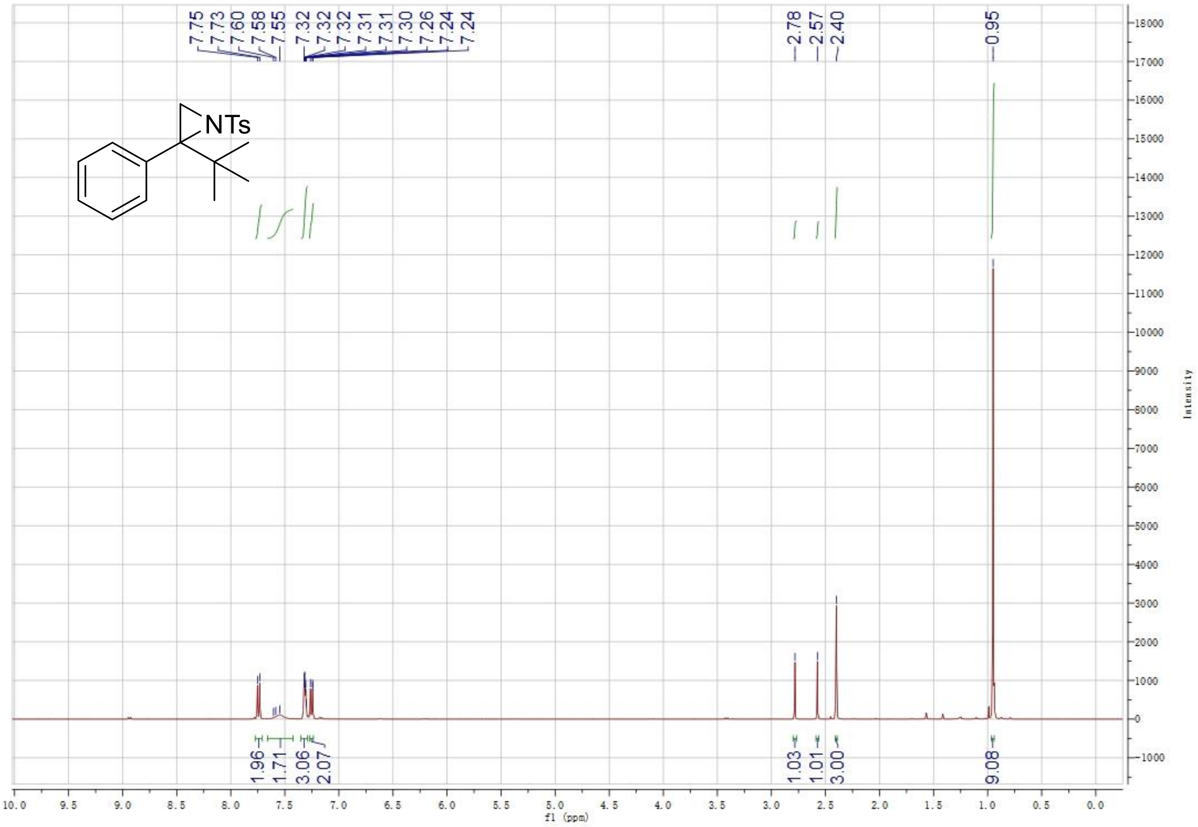

^{13}C NMR (101 MHz, 293 K, $CDCl_3$)

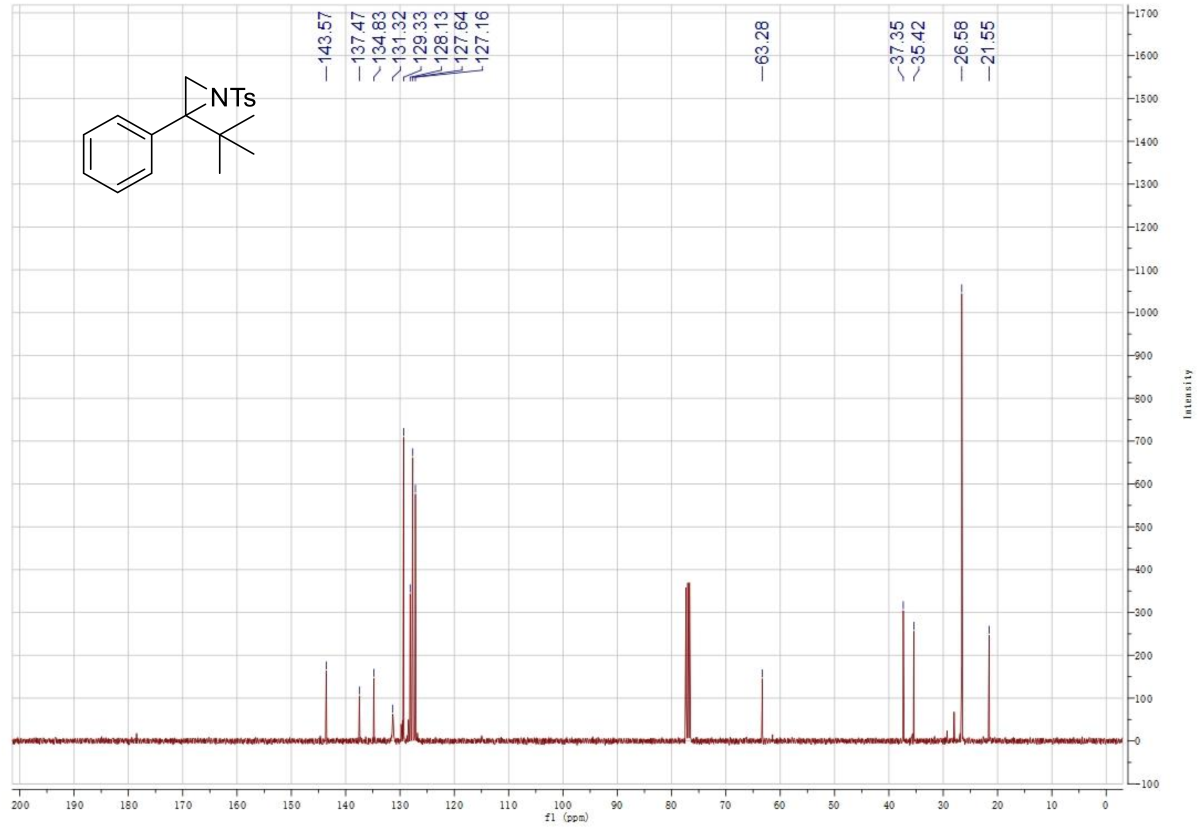

Supplementary Figure 58. *1-Phenyl-7-tosyl-7-azabicyclo[4.1.0]heptane* (***11t***)

^{1}H NMR (600 MHz, 293 K, $CDCl_3$)

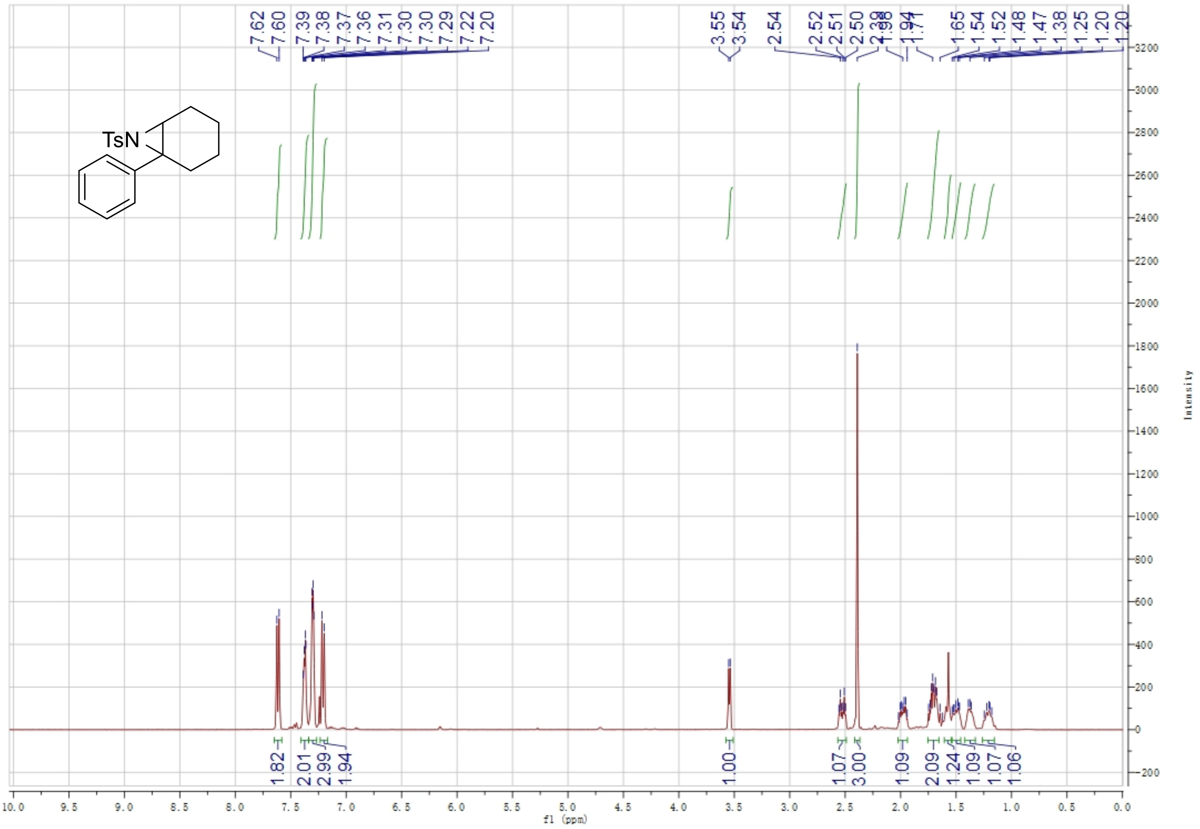

^{13}C NMR (151 MHz, 293 K, $CDCl_3$)

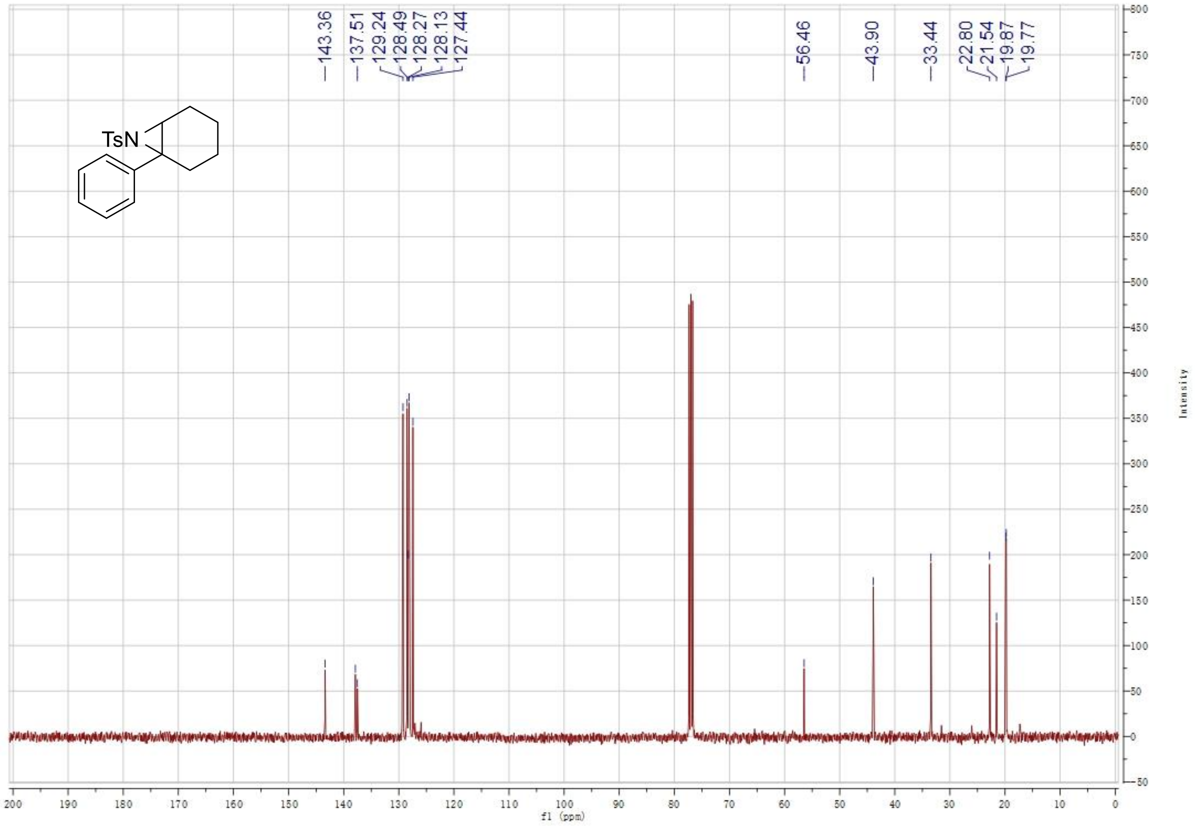

Supplementary Figure 59. *1-Methyl-6-tosyl-6-azabicyclo[3.1.0]hexane* (***11u***)

^{1}H NMR (600 MHz, 293 K, $CDCl_3$)

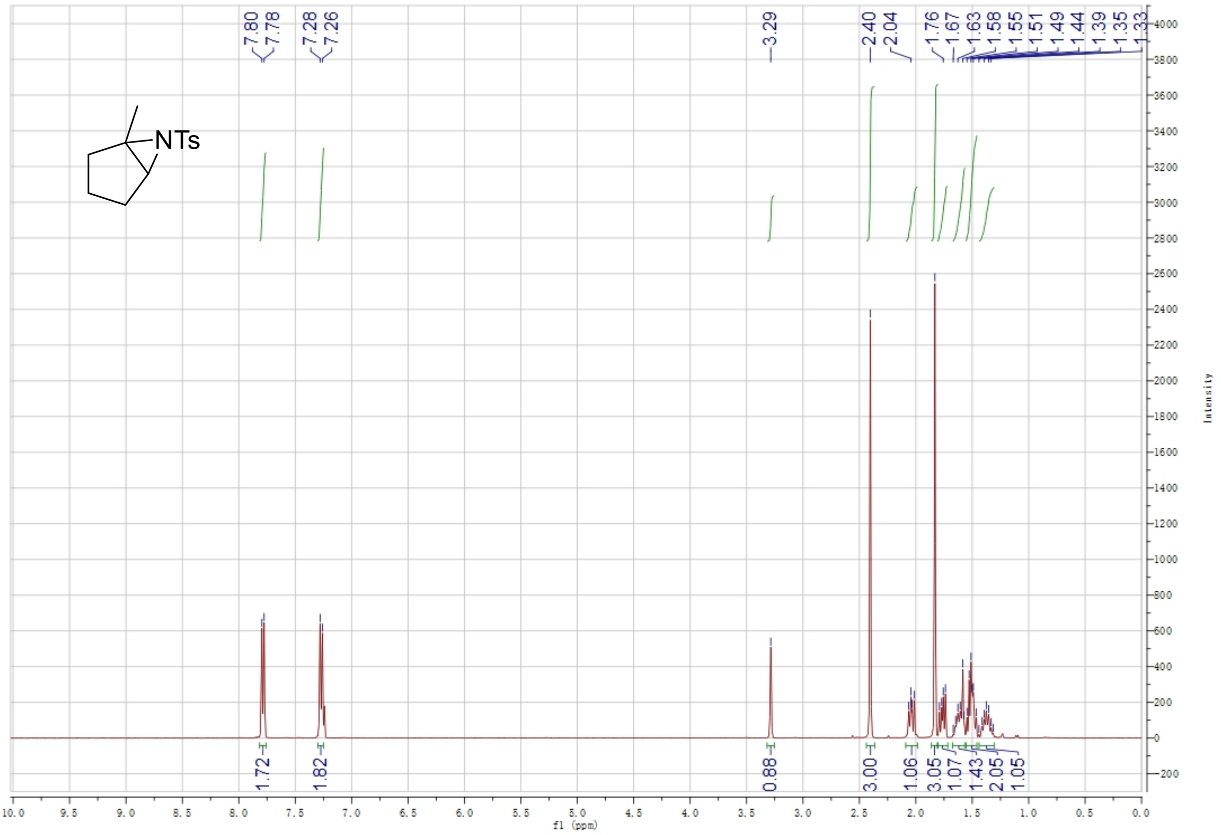

^{13}C NMR (151 MHz, 293 K, $CDCl_3$)

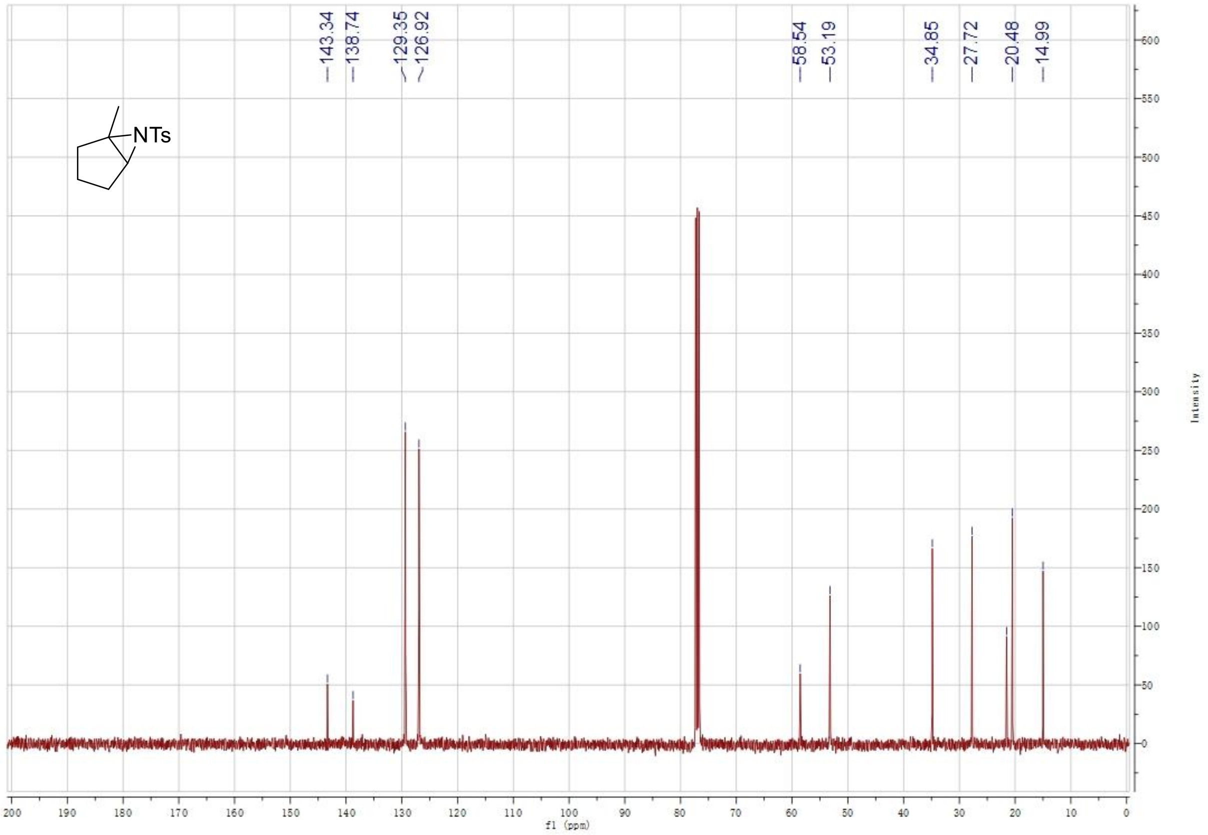

Supplementary Figure 60. *2-Methyl-2-(phenylethynyl)-1-tosylaziridine* (***11v***)

^{1}H NMR (600 MHz, 293 K, $CDCl_3$)

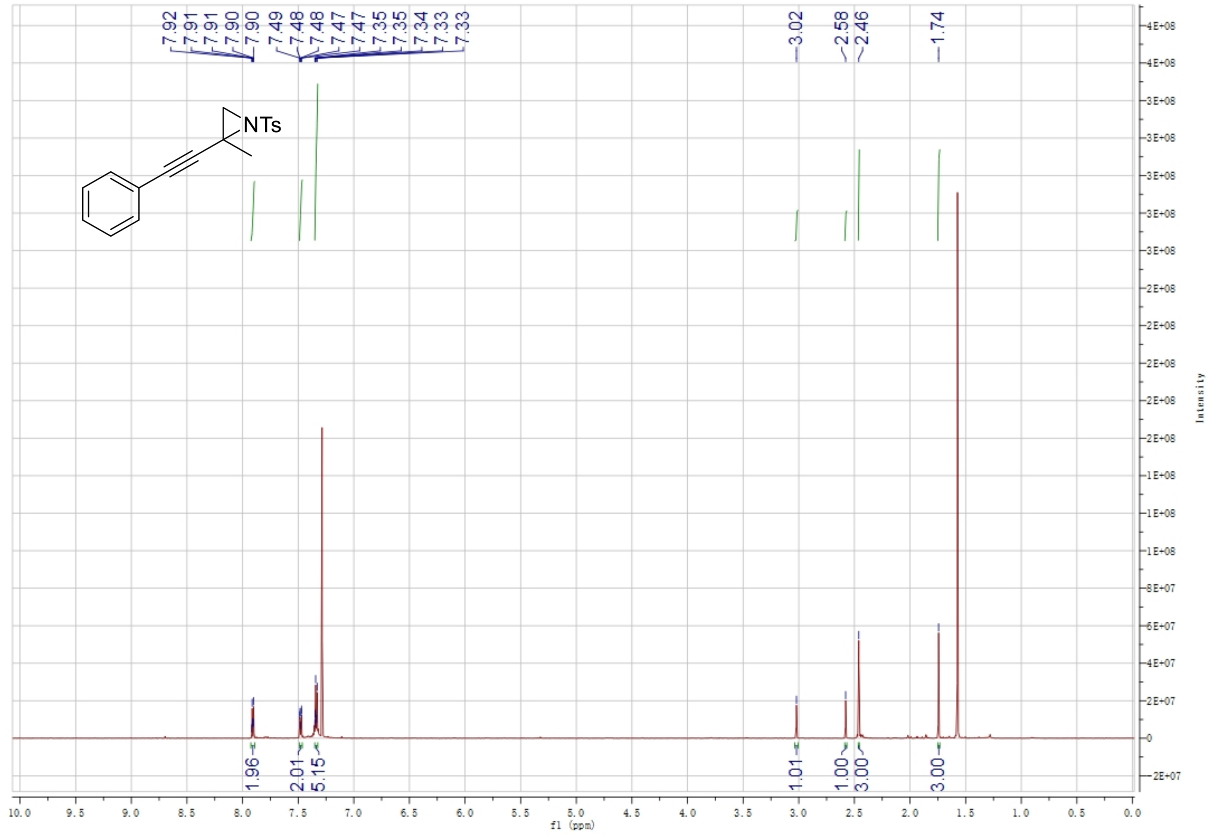

^{13}C NMR (151 MHz, 293 K, $CDCl_3$)

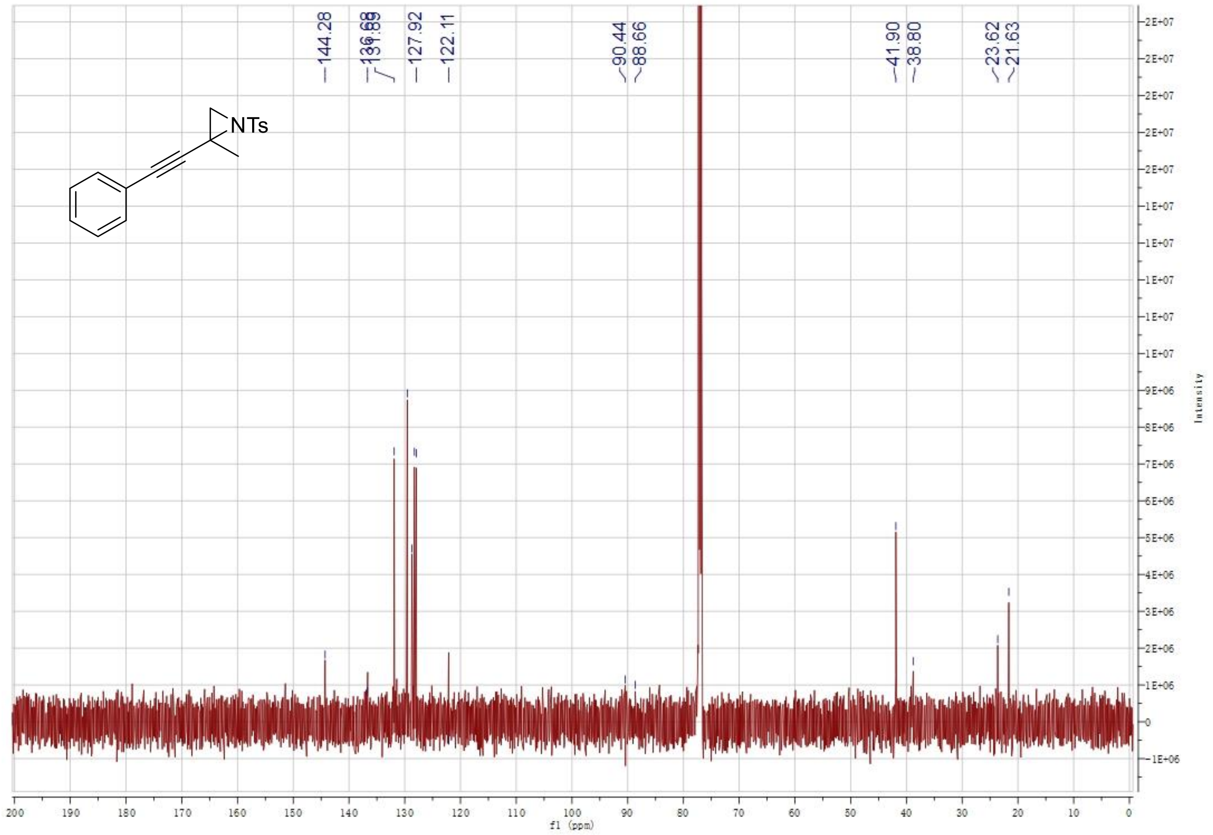

Supplementary Figure 61. *2-Cyclopentyl-2-methyl-1-tosylaziridine* ***(11w)***

^{1}H NMR (600 MHz, 293 K, $CDCl_3$)

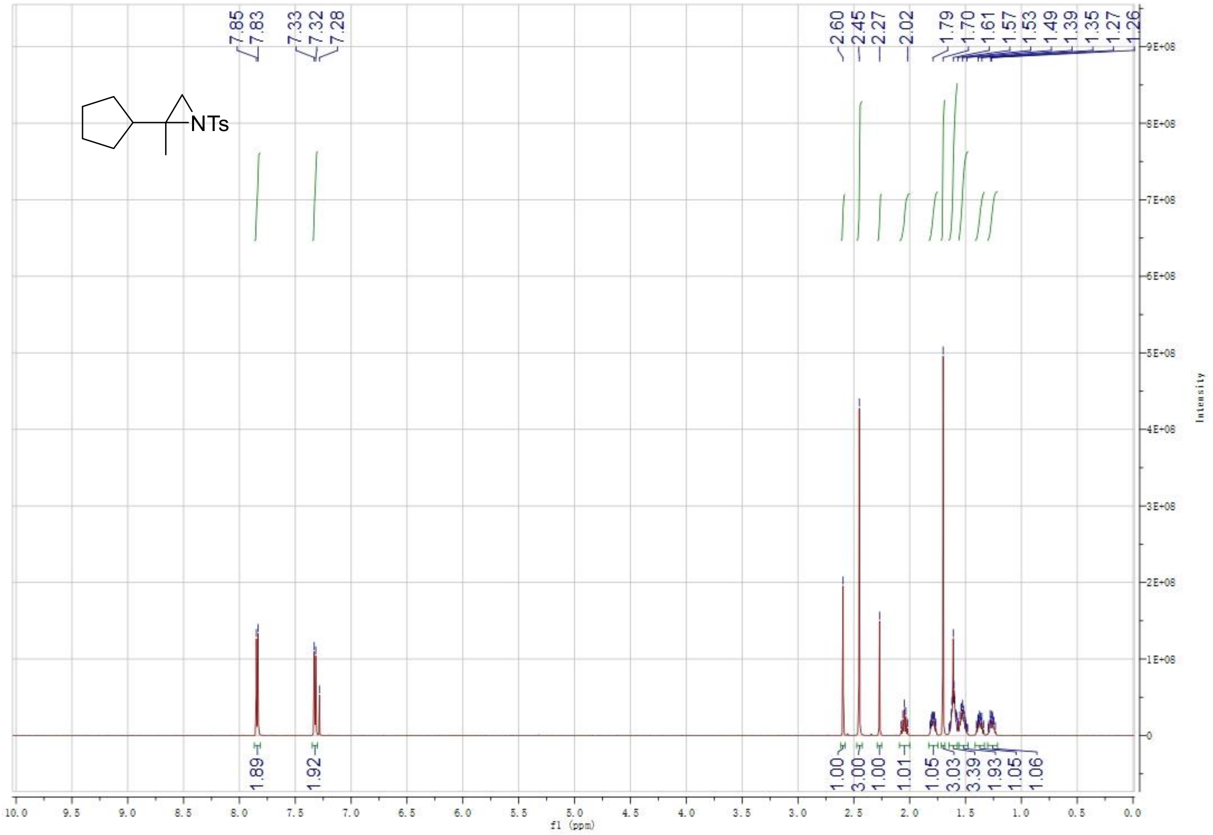

^{13}C NMR (151 MHz, 293 K, $CDCl_3$)

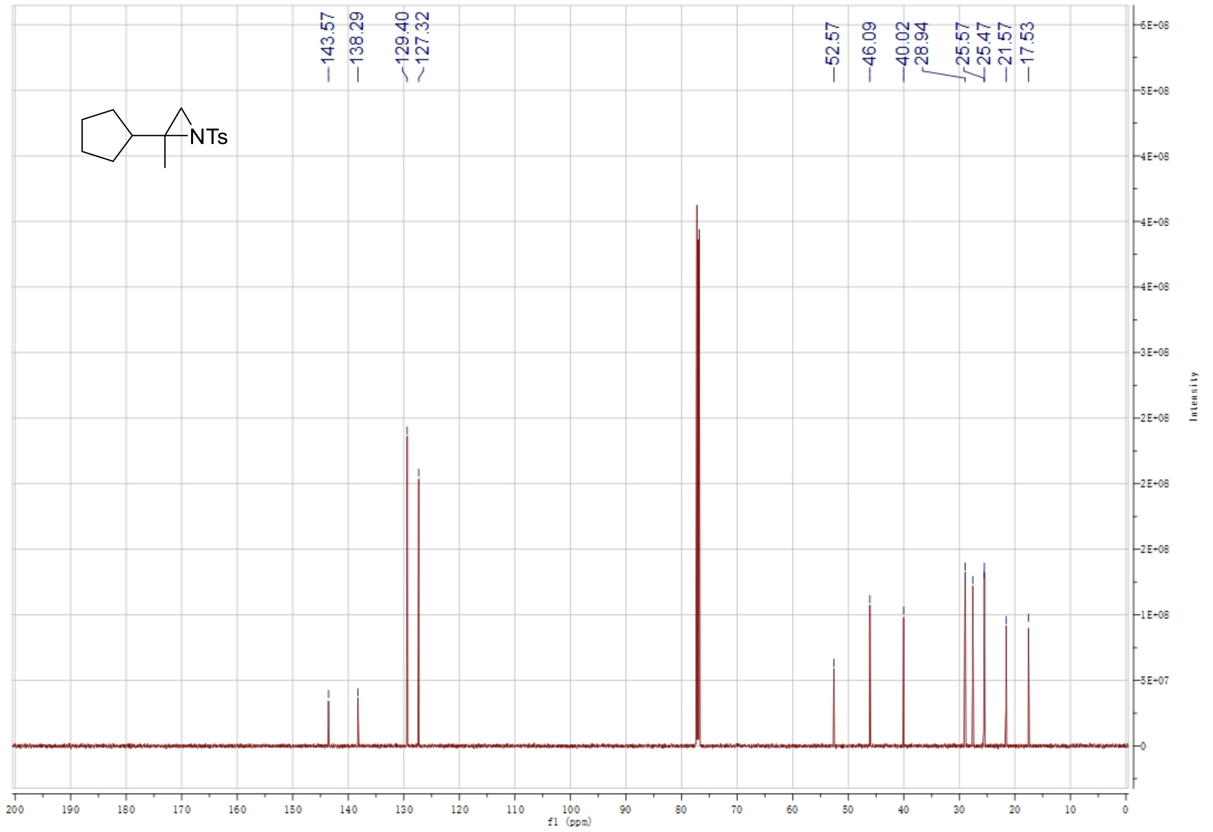

Supplementary Figure 62. *2-Cyclohexyl-2-methyl-1-tosylaziridine (**11x**)*

^{1}H NMR (600 MHz, 293 K, $CDCl_3$)

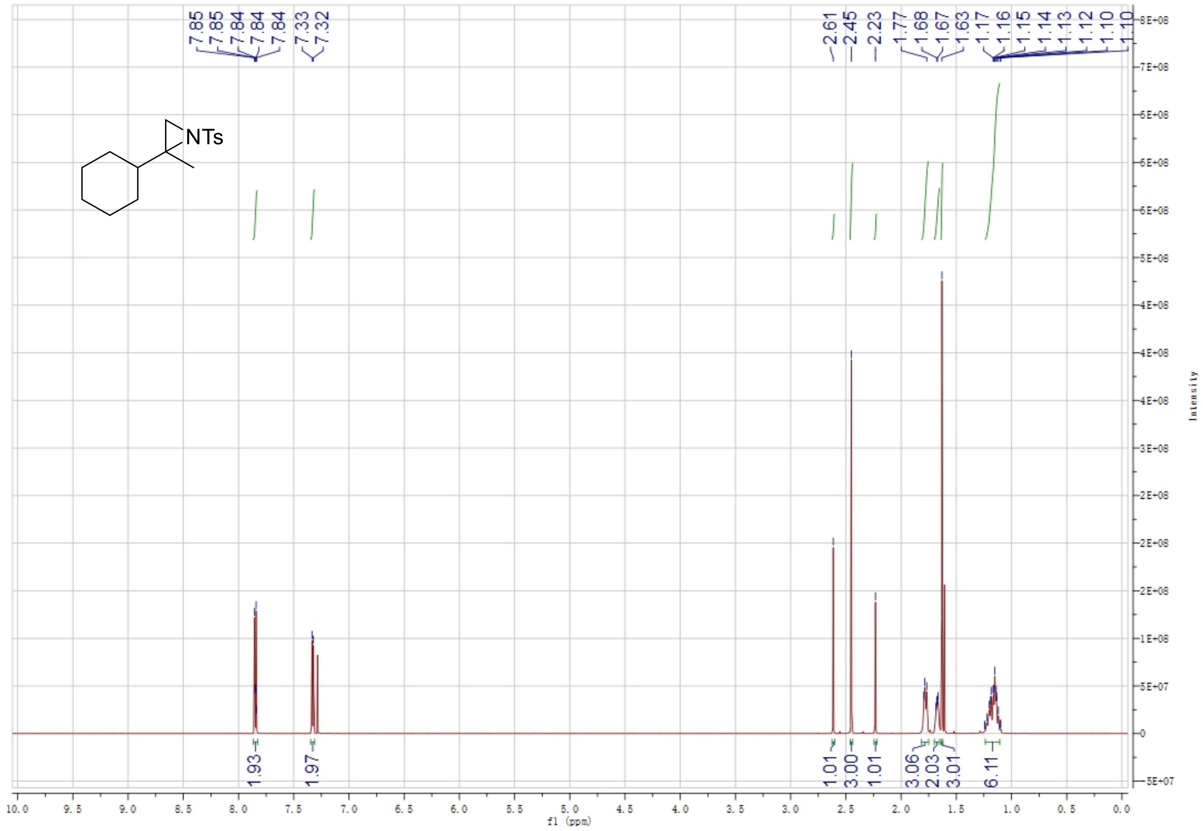

^{13}C NMR (151 MHz, 293 K, $CDCl_3$)

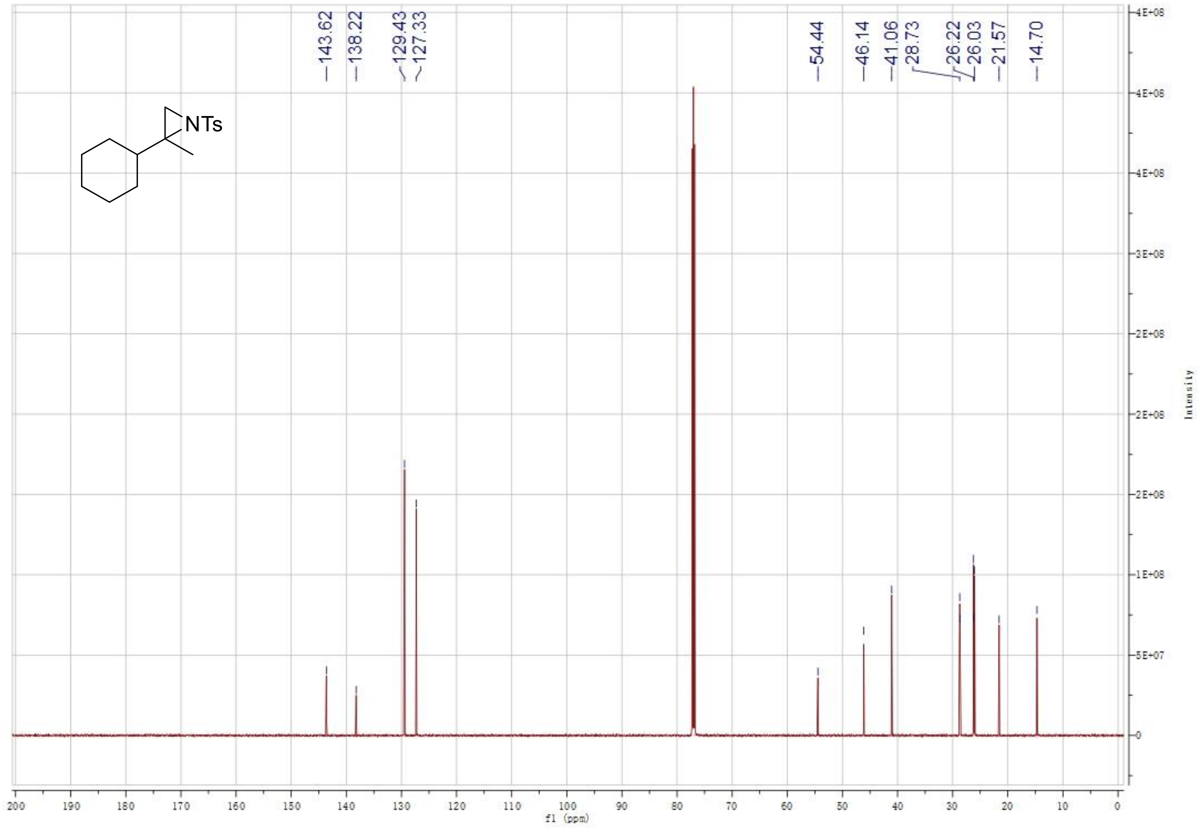

Supplementary Figure 63. *2-Methyl-2-(tetrahydro-2H-pyran-4-yl)-1-tosylaziridine* (***11y***)

^{1}H NMR (600 MHz, 293 K, $CDCl_3$)

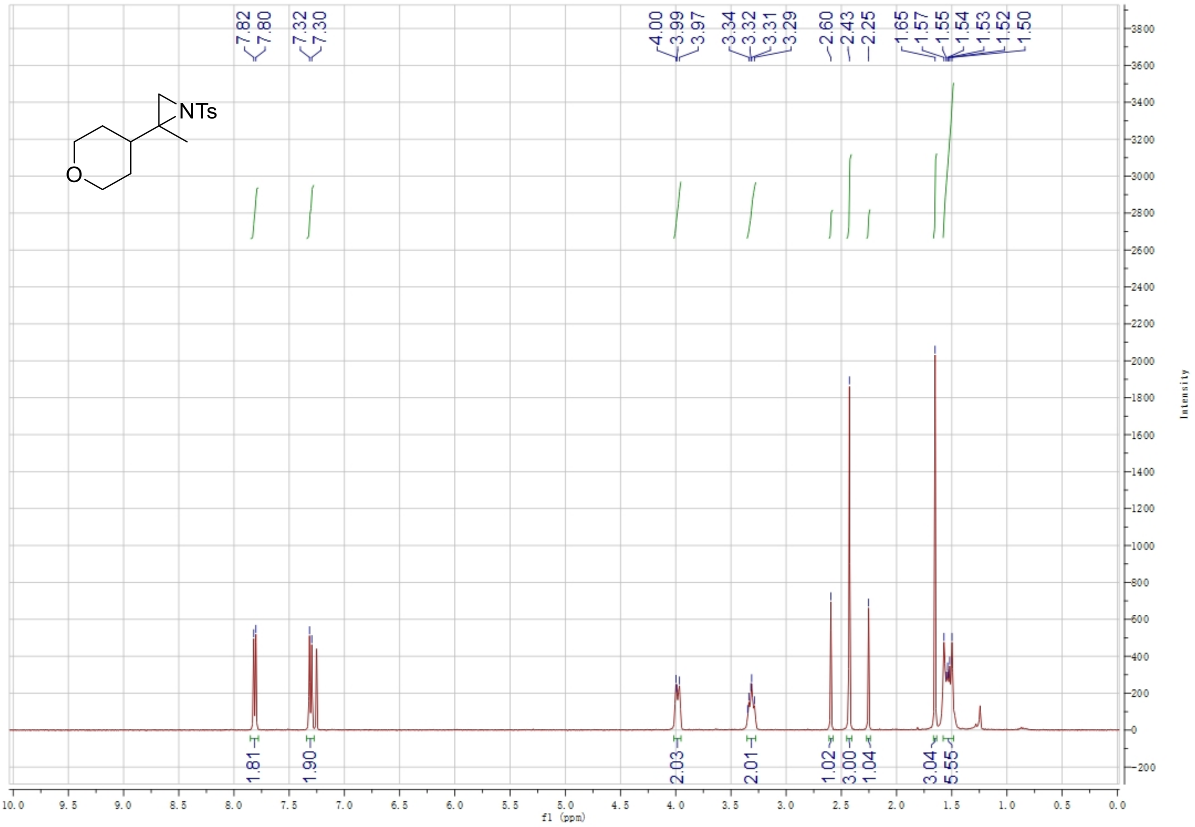

^{13}C NMR (151 MHz, 293 K, $CDCl_3$)

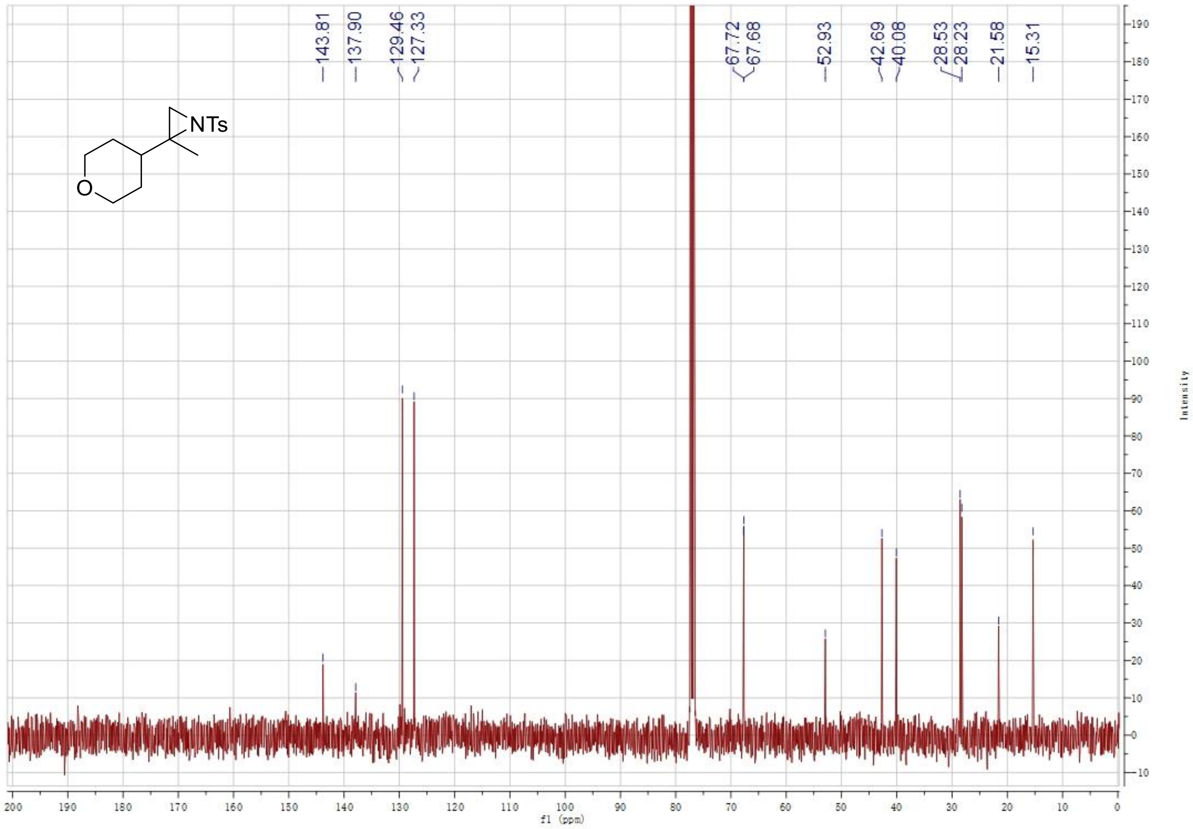

Supplementary Figure 64. *2-(tert-Butyl)-2-methyl-1-tosylaziridine (**11z**)*

^{1}H NMR (600 MHz, 293 K, $CDCl_3$)

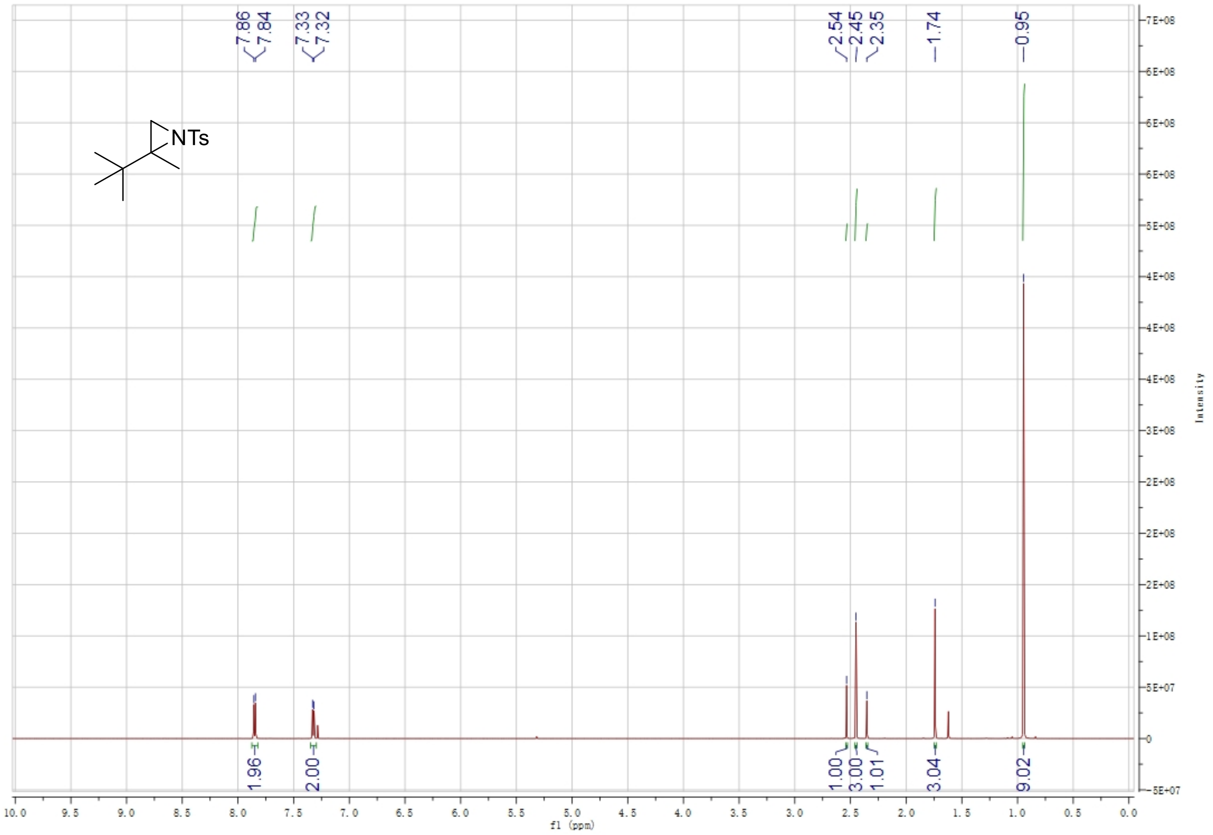

^{13}C NMR (151 MHz, 293 K, $CDCl_3$)

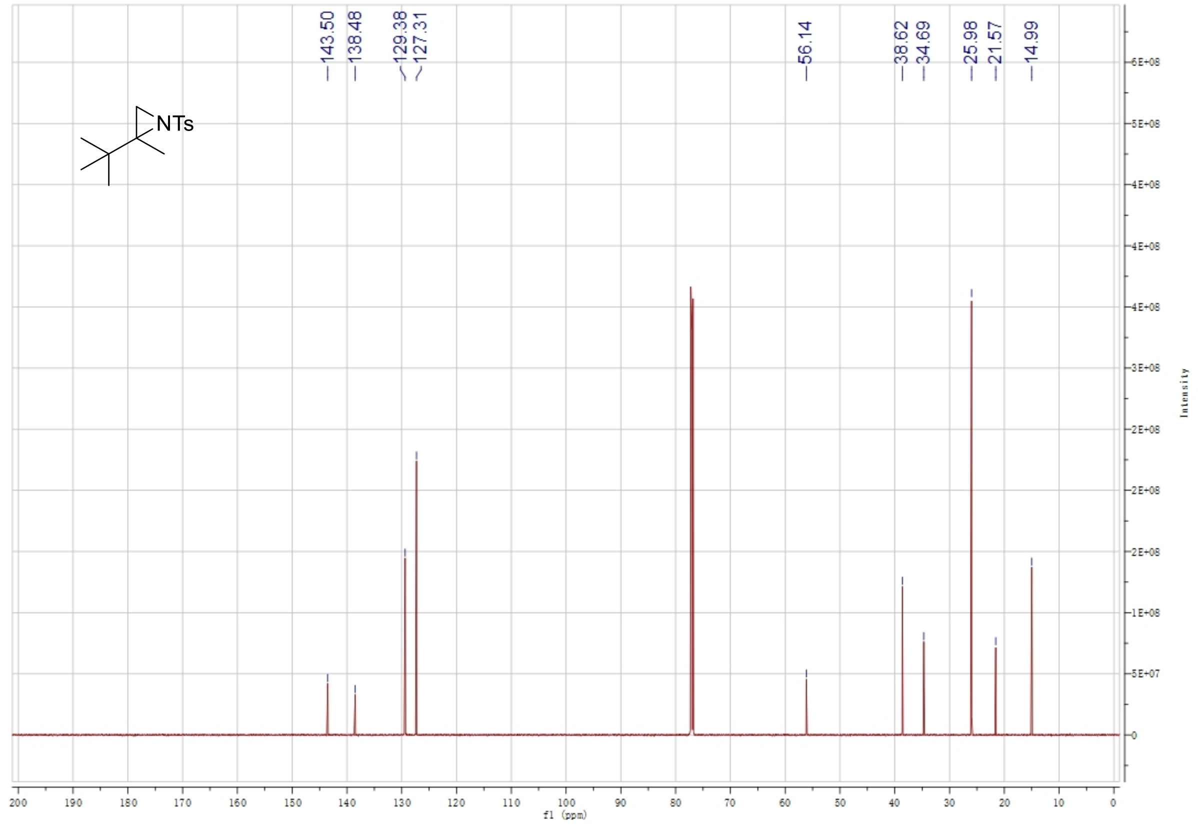

Supplementary Figure 65. *2-Methyl-2-(1-phenylethyl)-1-tosylaziridine (**11aa**)*

^{1}H NMR (600 MHz, 293 K, $CDCl_3$)

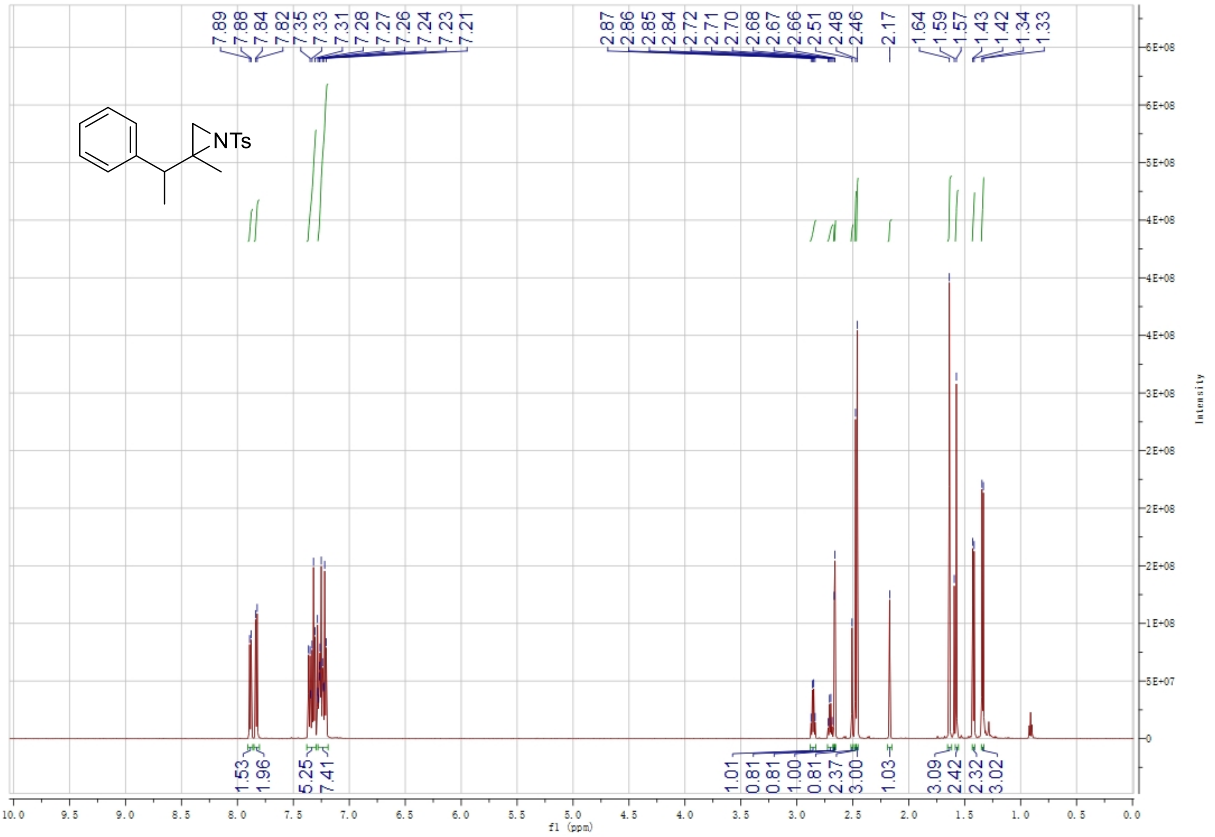

^{13}C NMR (151 MHz, 293 K, $CDCl_3$)

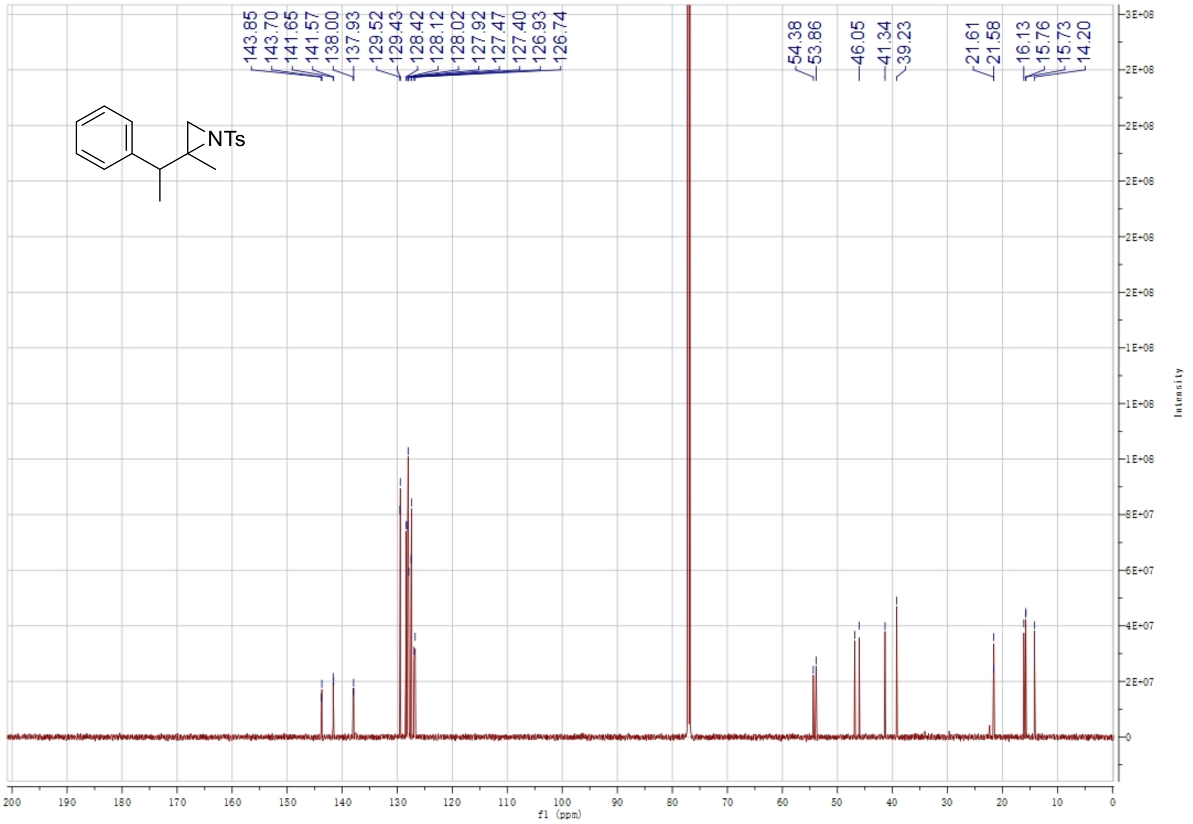

Supplementary Figure 66. *2-Benzyl-2-methyl-1-tosylaziridine (**11ab**)*

^{1}H NMR (600 MHz, 293 K, $CDCl_3$)

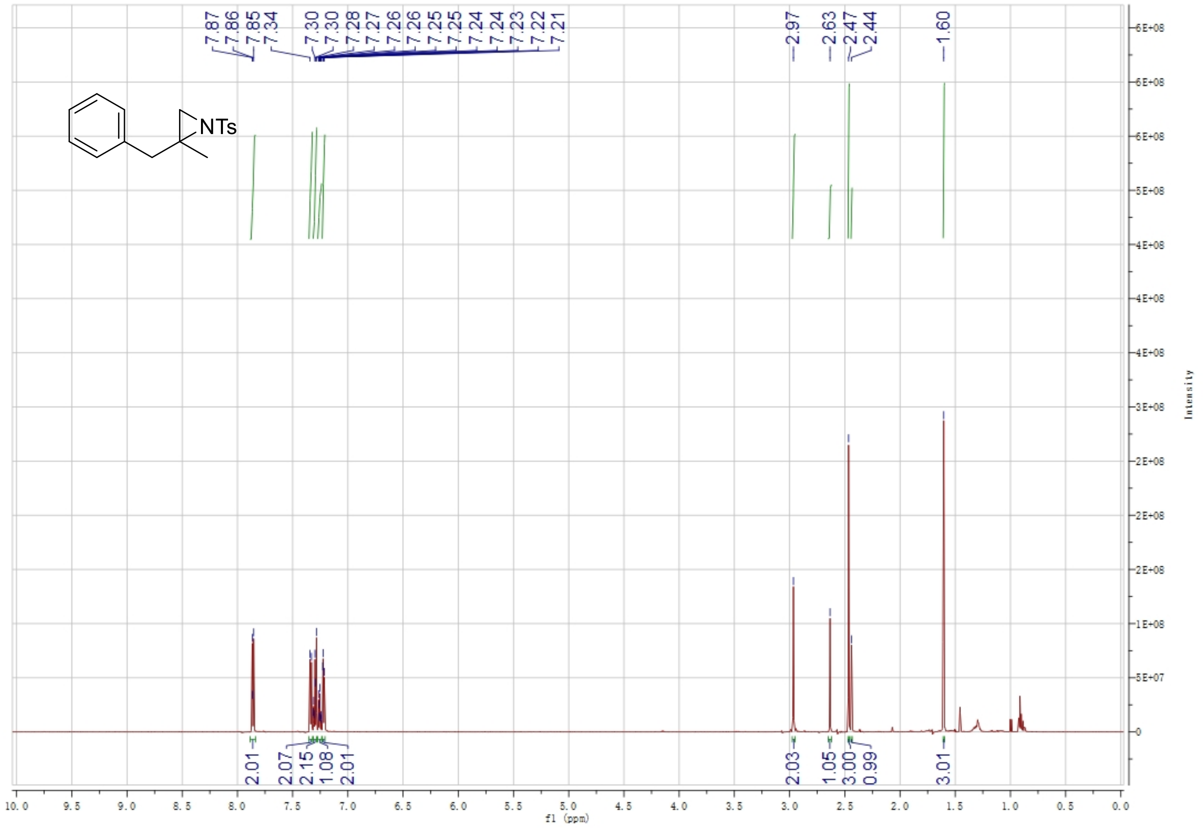

^{13}C NMR (151 MHz, 293 K, $CDCl_3$)

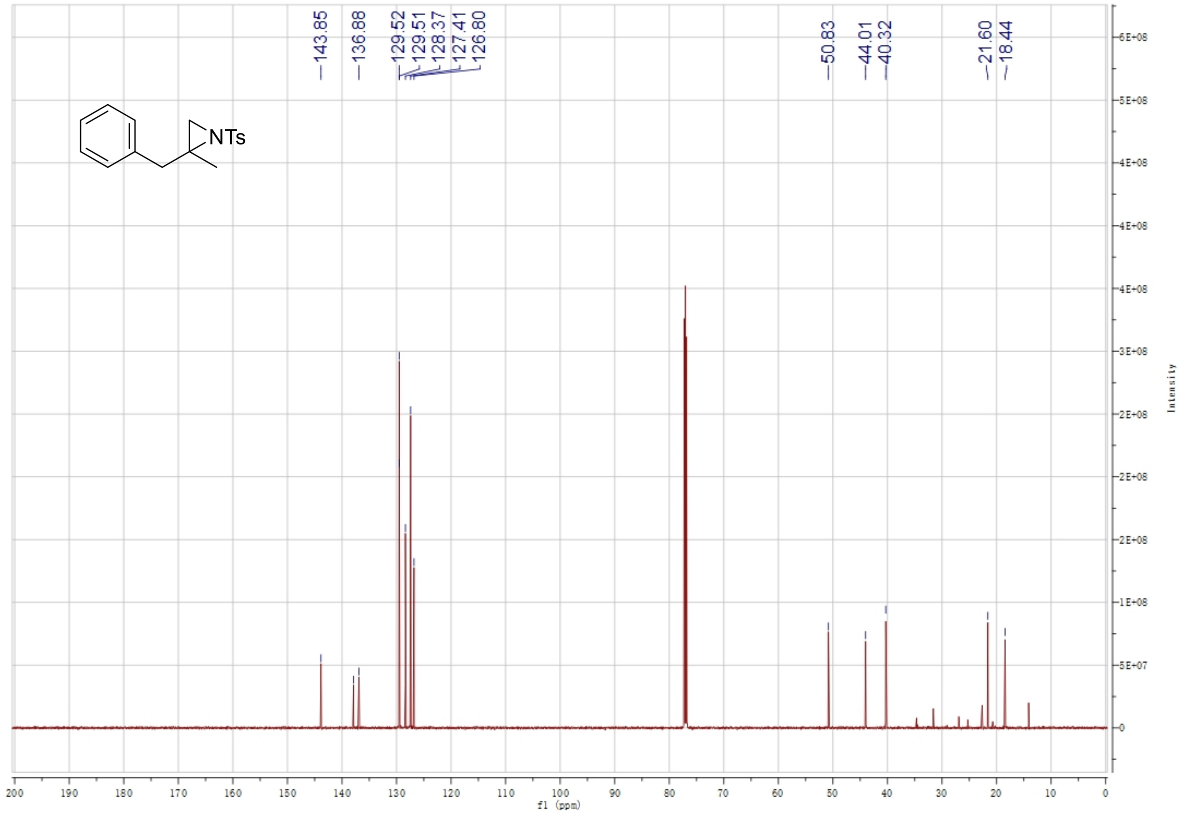

Supplementary Figure 67. *2-Methyl-2-phenethyl-1-tosylaziridine (**11ac**)*

^{1}H NMR (600 MHz, 293 K, $CDCl_3$)

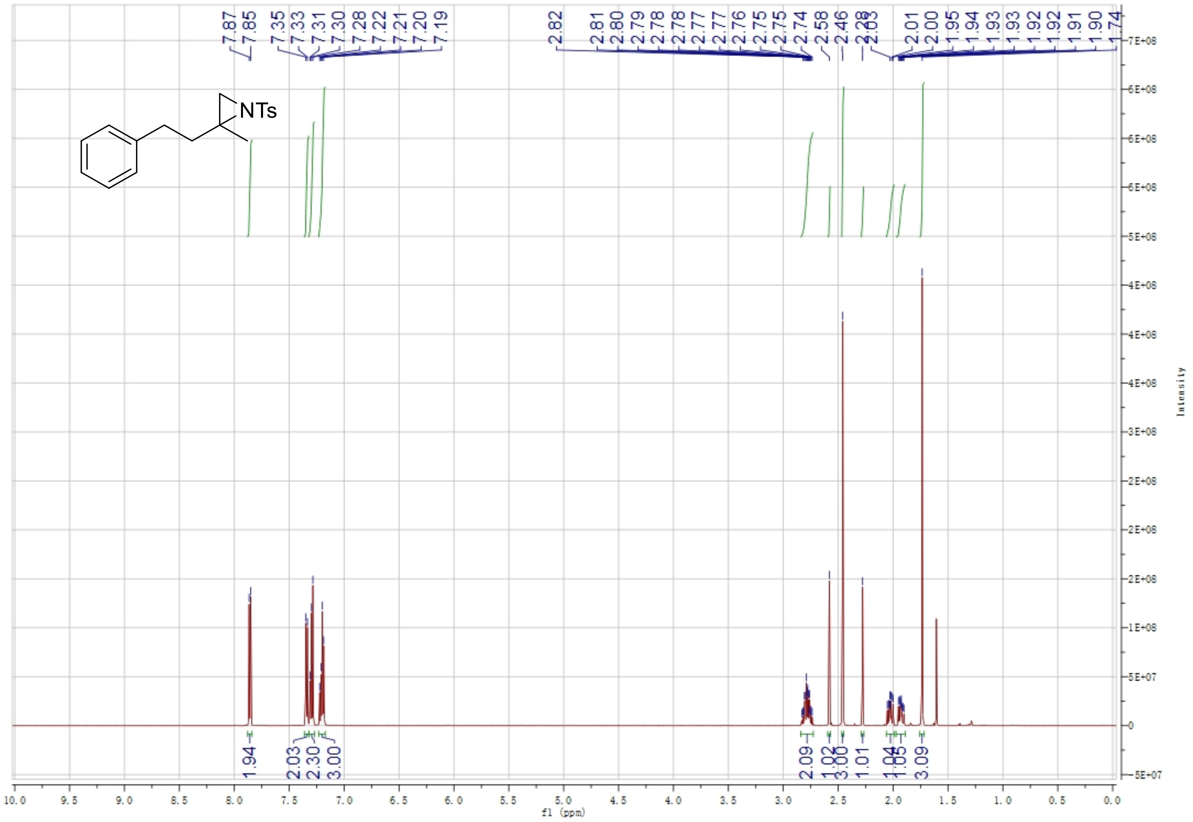

^{13}C NMR (151 MHz, 293 K, $CDCl_3$)

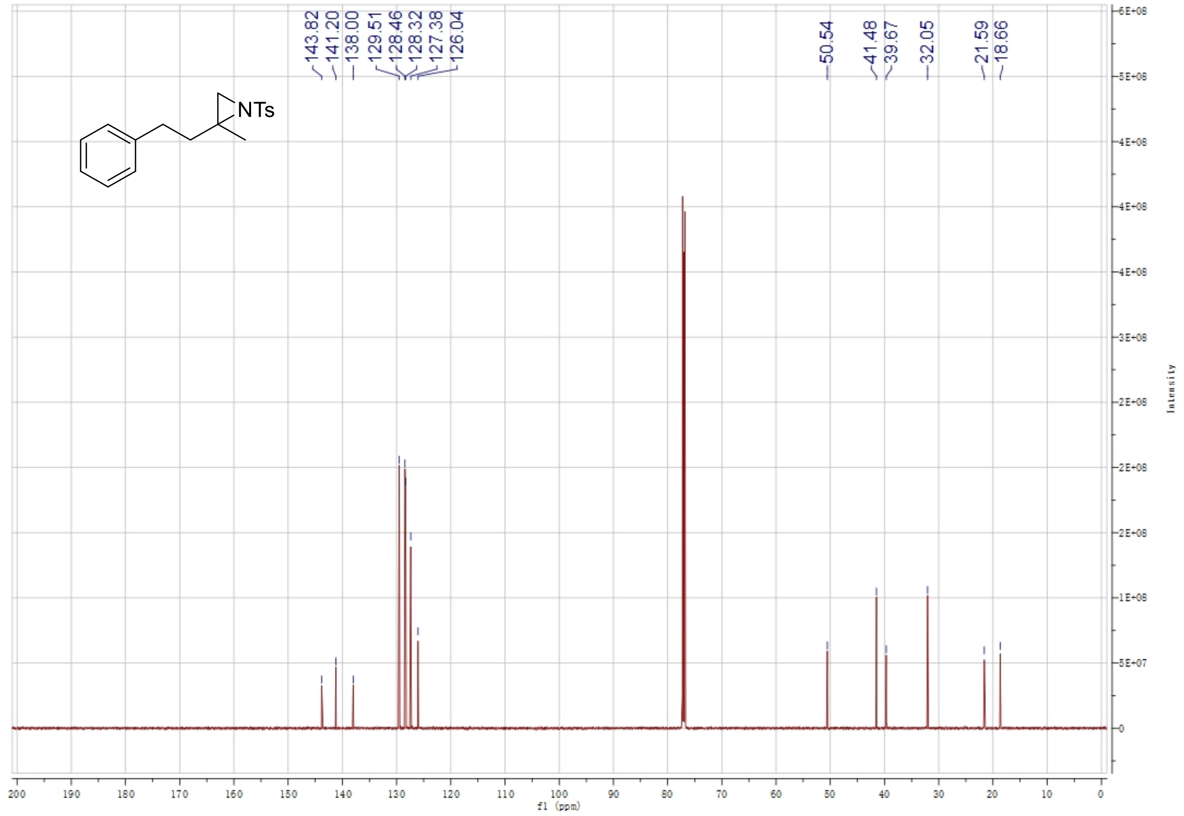

Supplementary Figure 68. *2-Hexyl-2-methyl-1-tosylaziridine* (***11ad***)

^{1}H NMR (600 MHz, 293 K, $CDCl_3$)

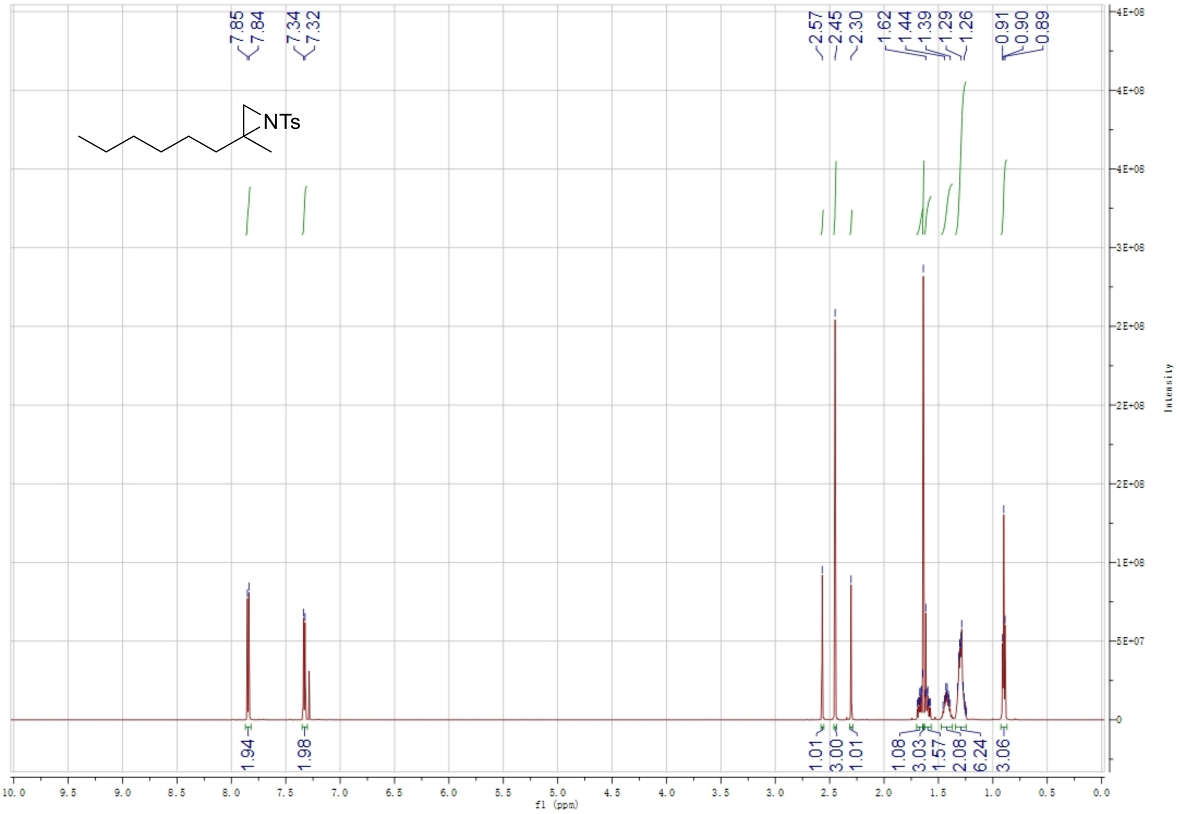

^{13}C NMR (151 MHz, 293 K, $CDCl_3$)

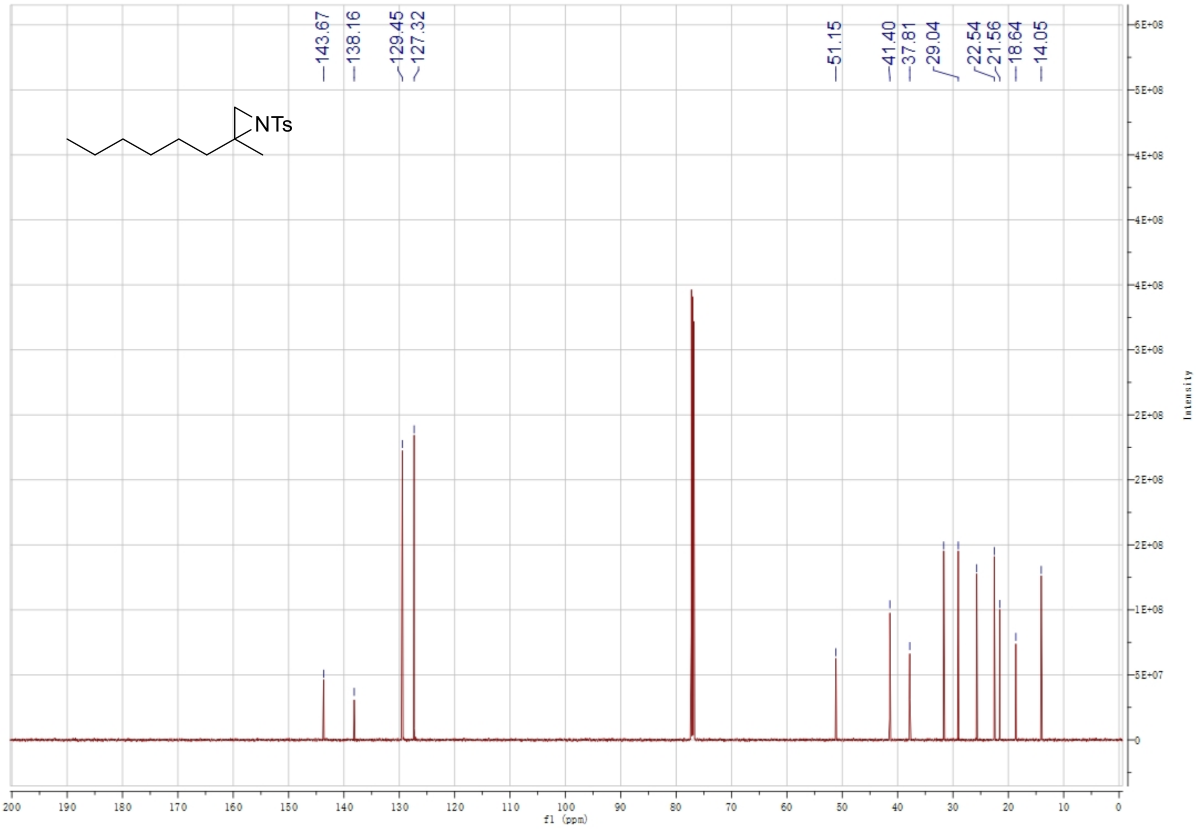

Supplementary Figure 69. *2-Ethyl-2-phenyl-1-tosylaziridine (**14**)*

^{1}H NMR (400 MHz, 293 K, $CDCl_3$)

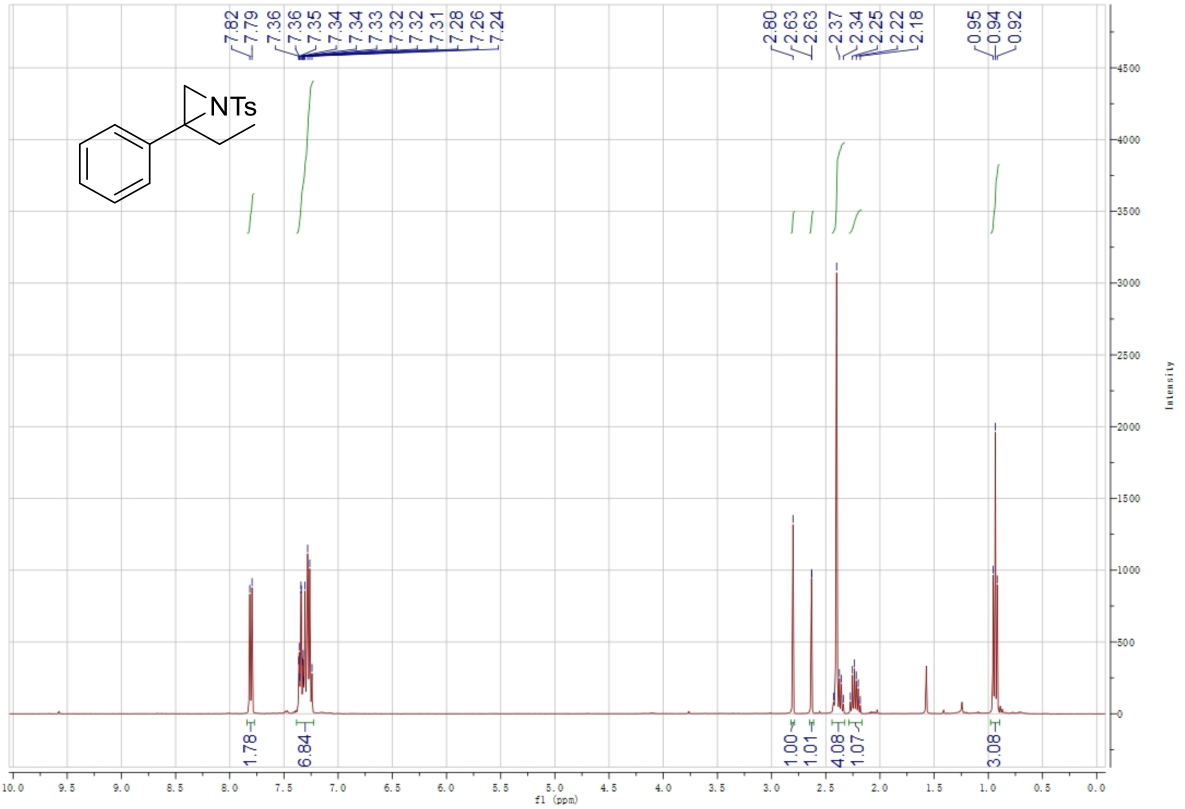

^{13}C NMR (101 MHz, 293 K, $CDCl_3$)

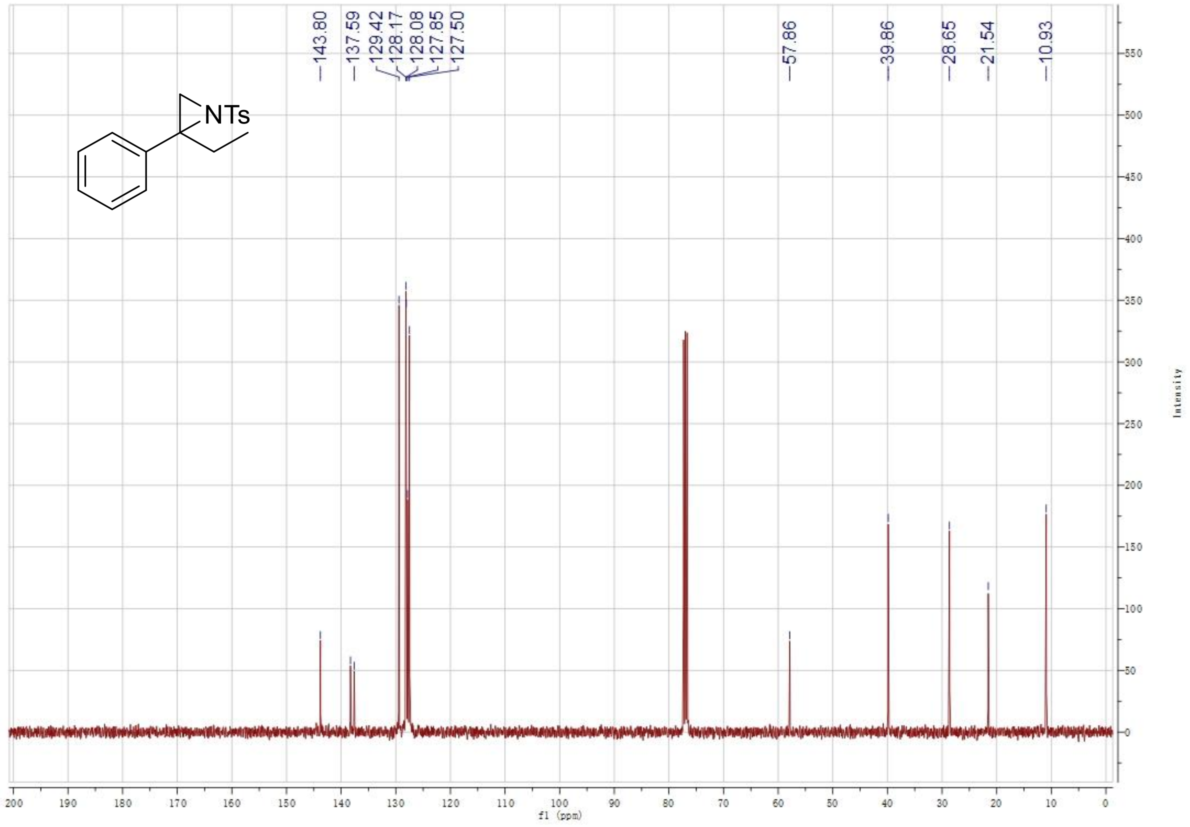

Supplementary Figure 70. *(2R,3R)-2-Methyl-3-phenyl-1-tosylaziridine (**20a**)*
From Z-*β*-methyl styrene
^{1}H NMR (600 MHz, 293 K, $CDCl_3$)

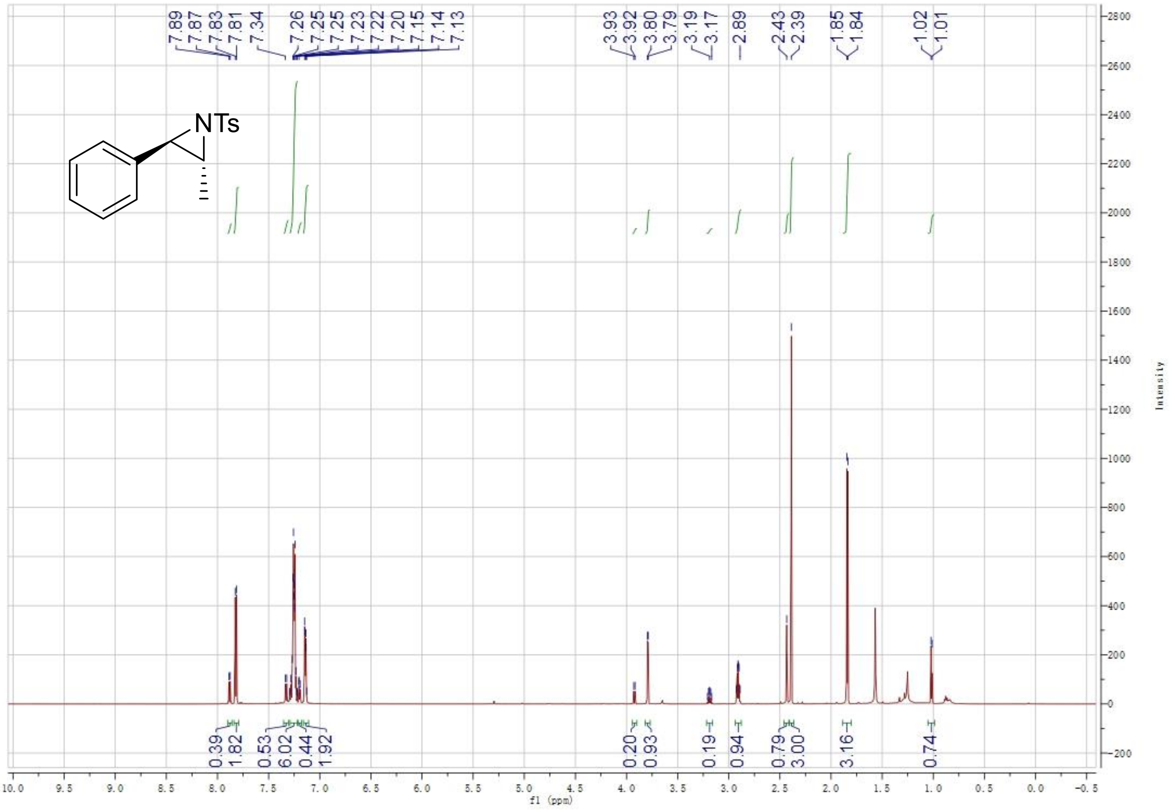

^{13}C NMR (151 MHz, 293 K, $CDCl_3$)

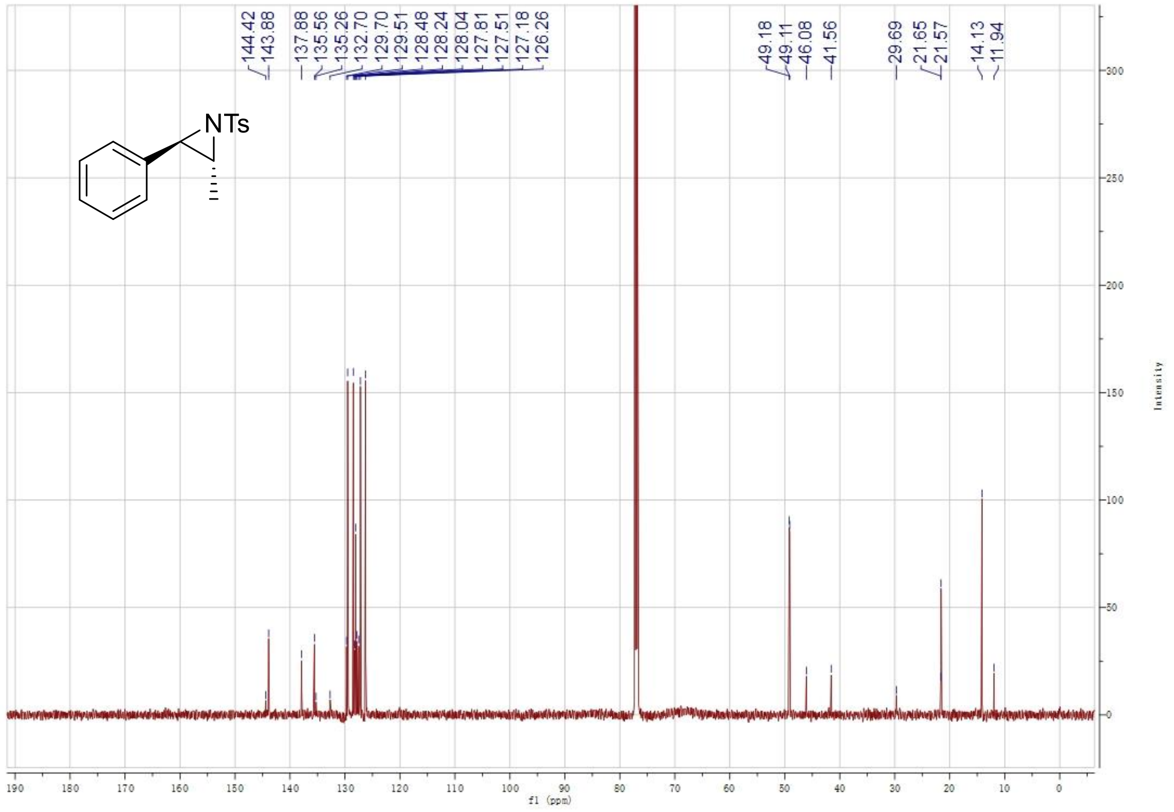

From E-β-methyl styrene

^{1}H NMR (600 MHz, 293 K, $CDCl_3$)

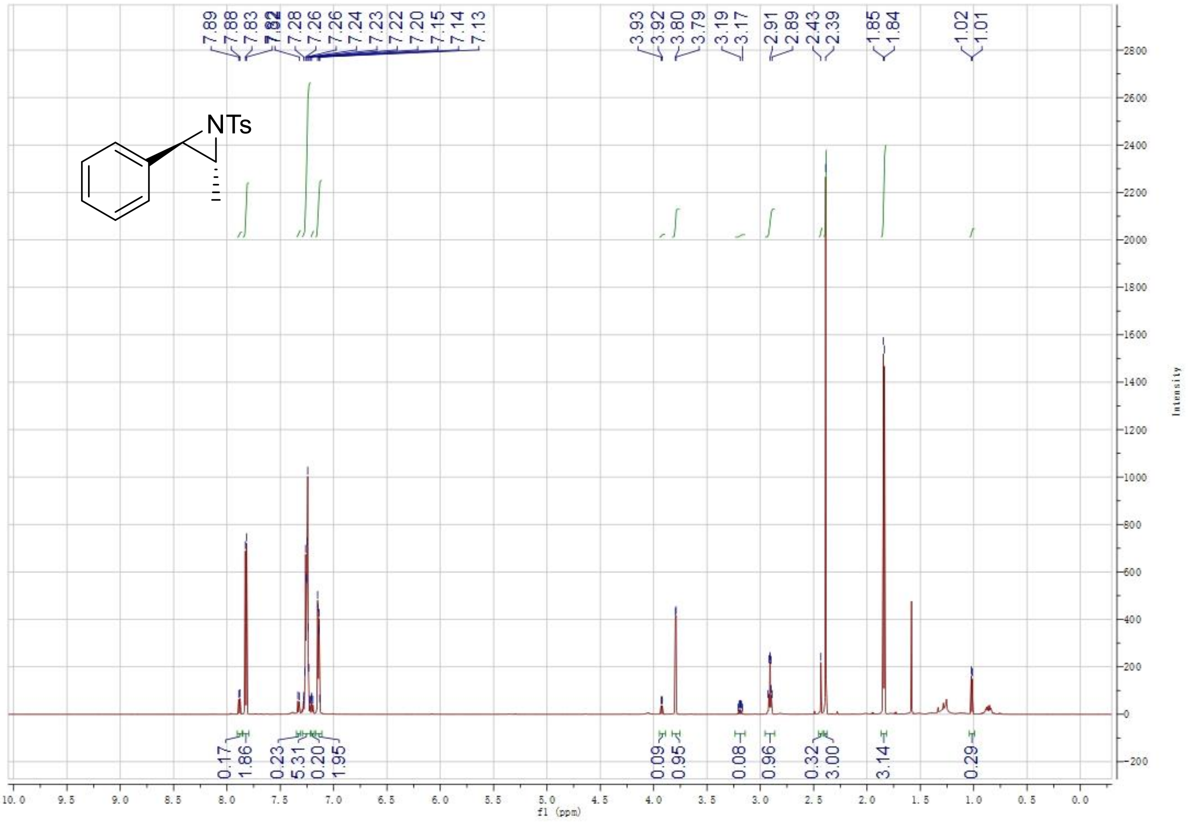

^{13}C NMR (151 MHz, 293 K, $CDCl_3$)

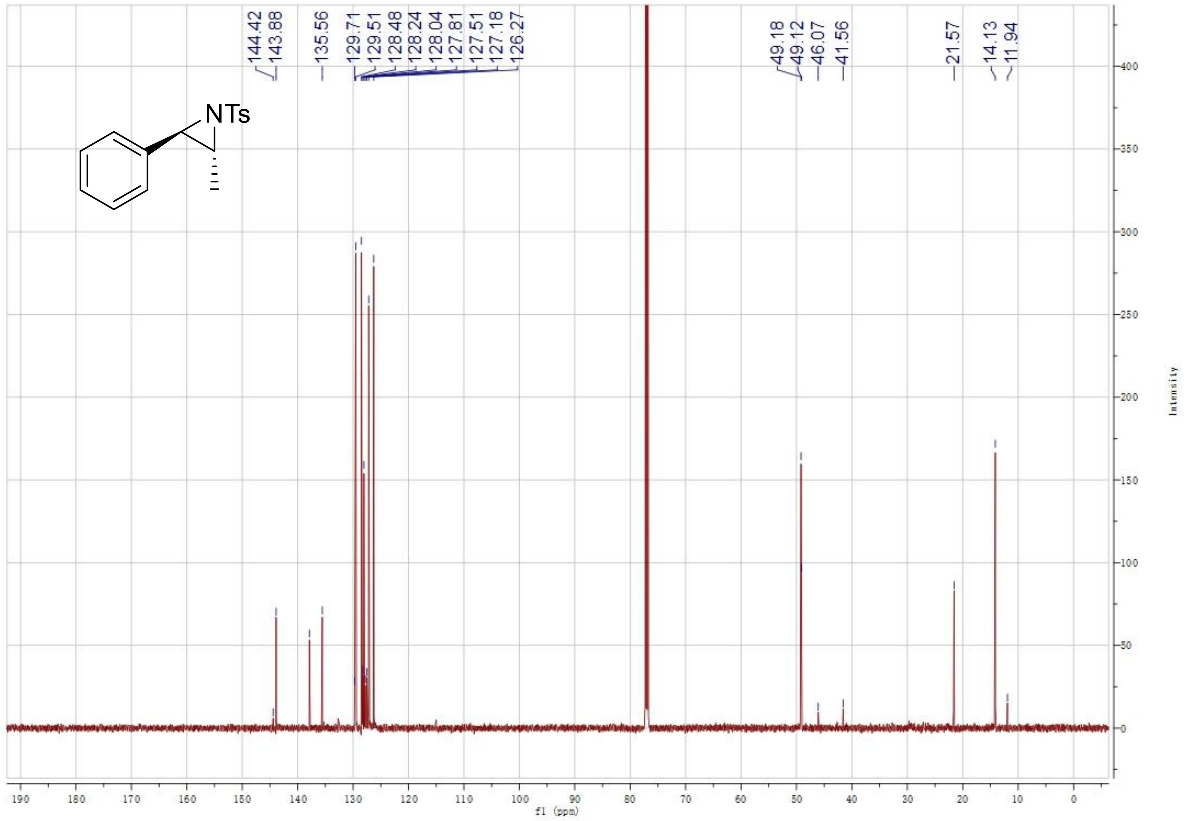

Supplementary Figure 71. *(2R,3R)-2-(4-Bromophenyl)-3-methyl-1-tosylaziridine (**20b**)*

^{1}H NMR (600 MHz, 293 K, $CDCl_3$)

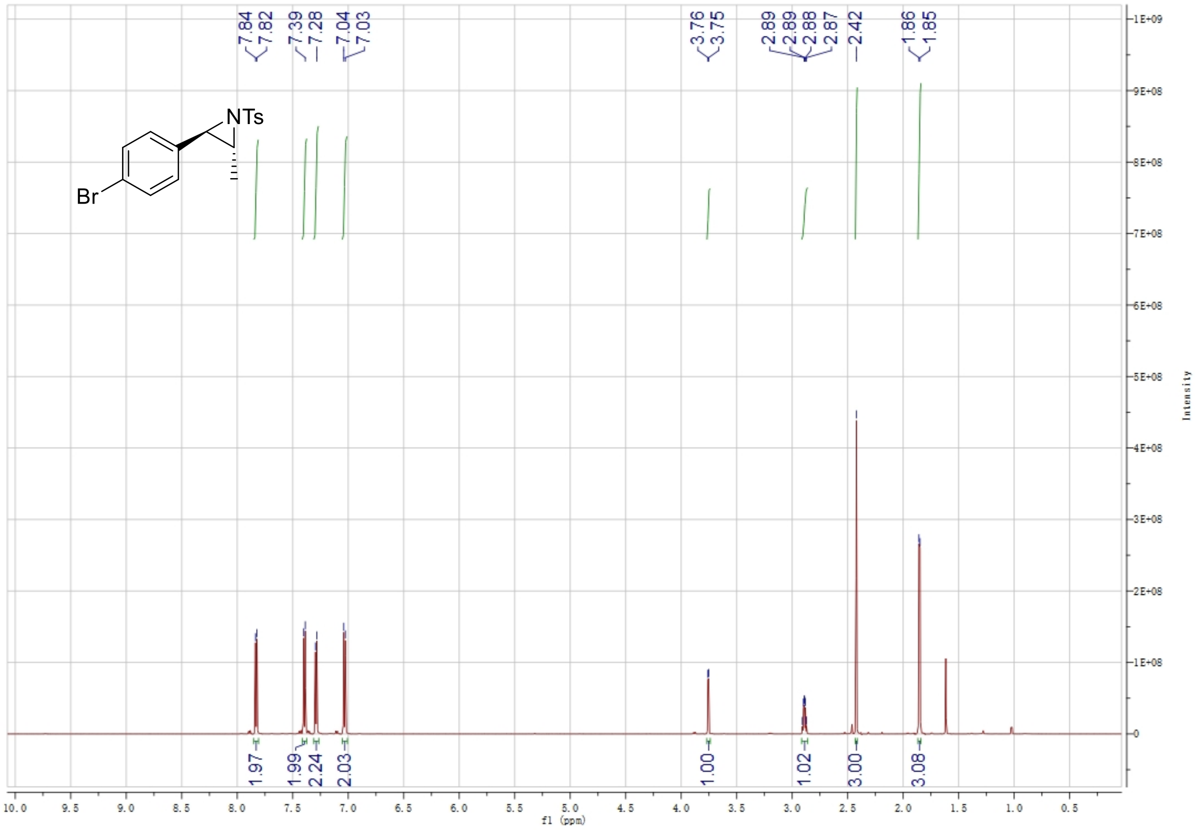

^{13}C NMR (151 MHz, 293 K, $CDCl_3$)

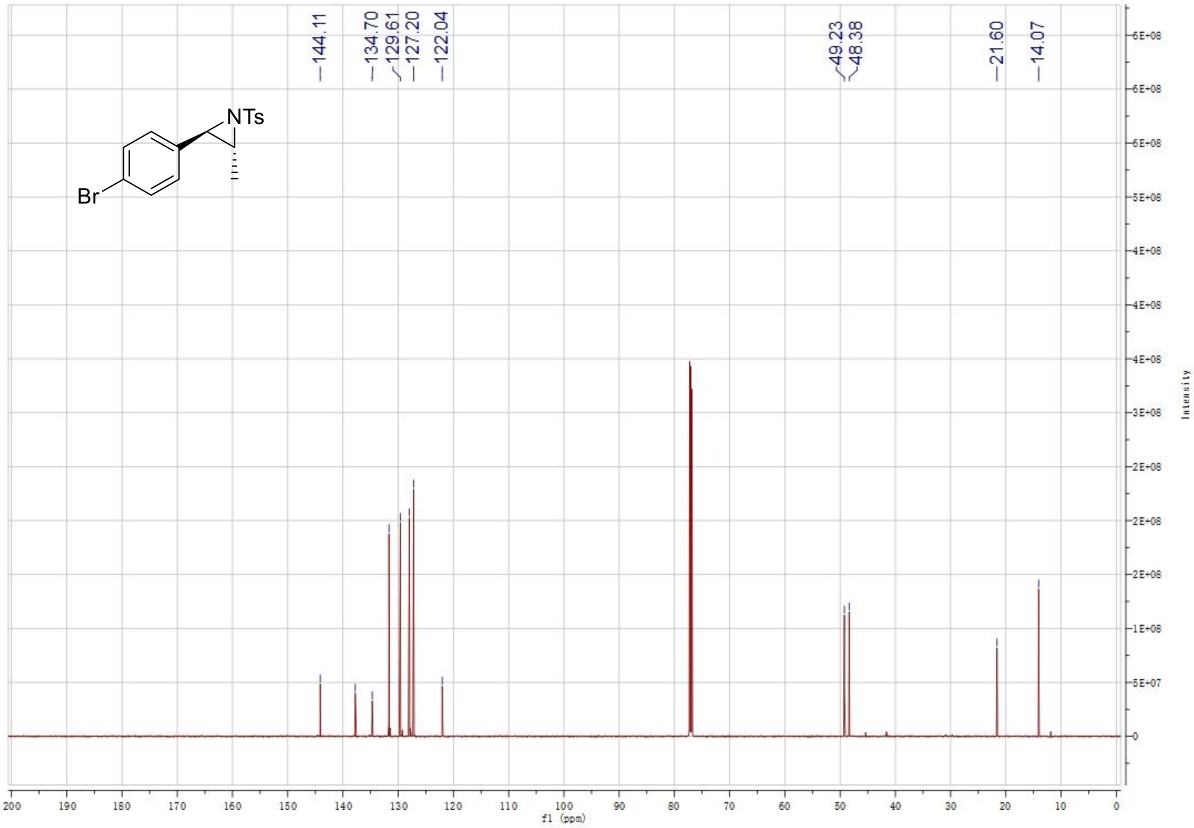

Supplementary Figure 72. *(2R,3R)-2-Methyl-1-tosyl-3-(4-(trifluoromethyl)phenyl)aziridine (**20c**)*

^{1}H NMR (600 MHz, 293 K, $CDCl_3$)

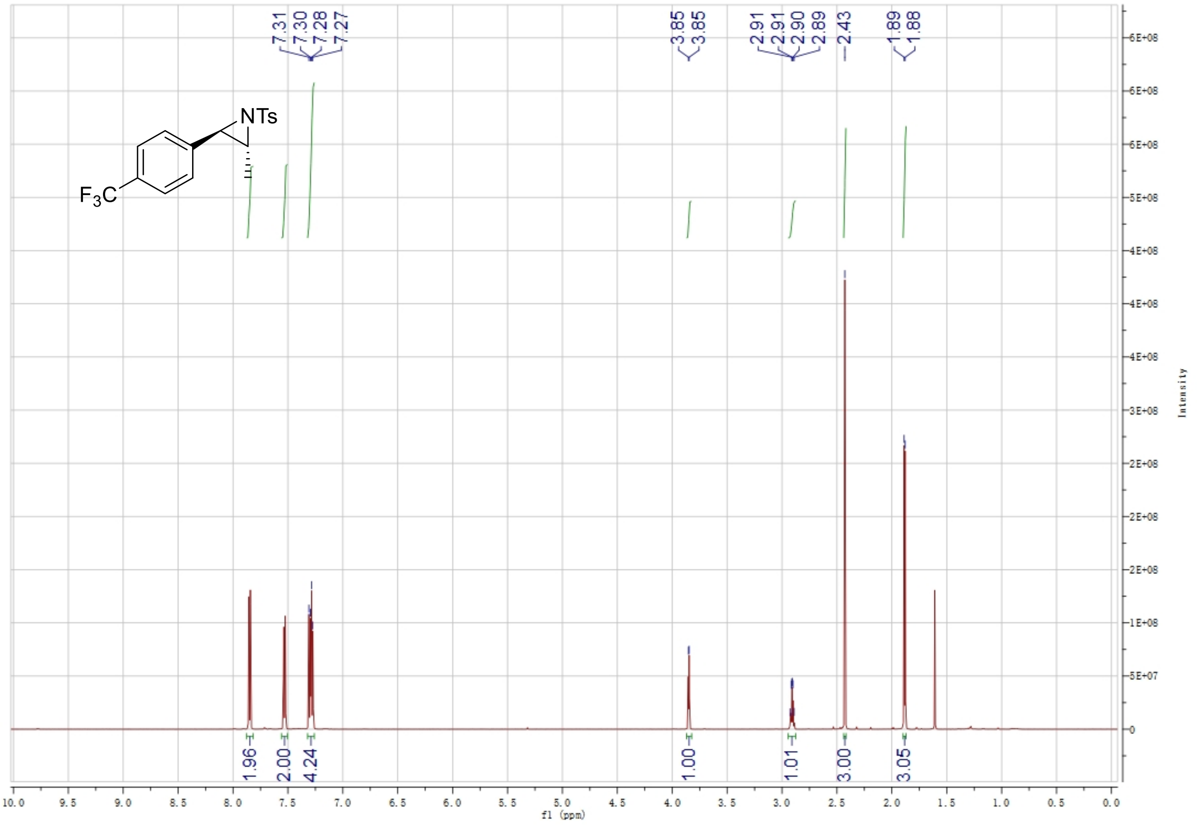

^{13}C NMR (151 MHz, 293 K, $CDCl_3$)

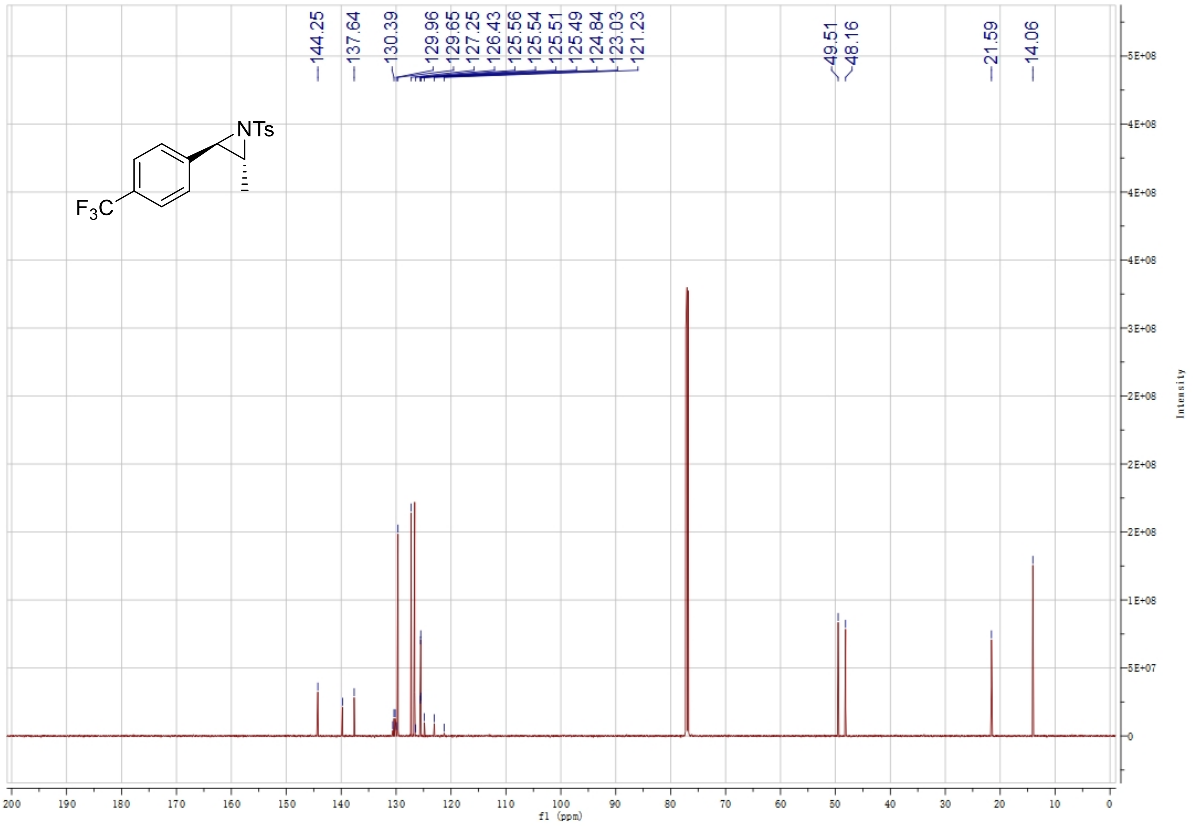

^{19}F NMR (565 MHz, 293 K, $CDCl_3$)

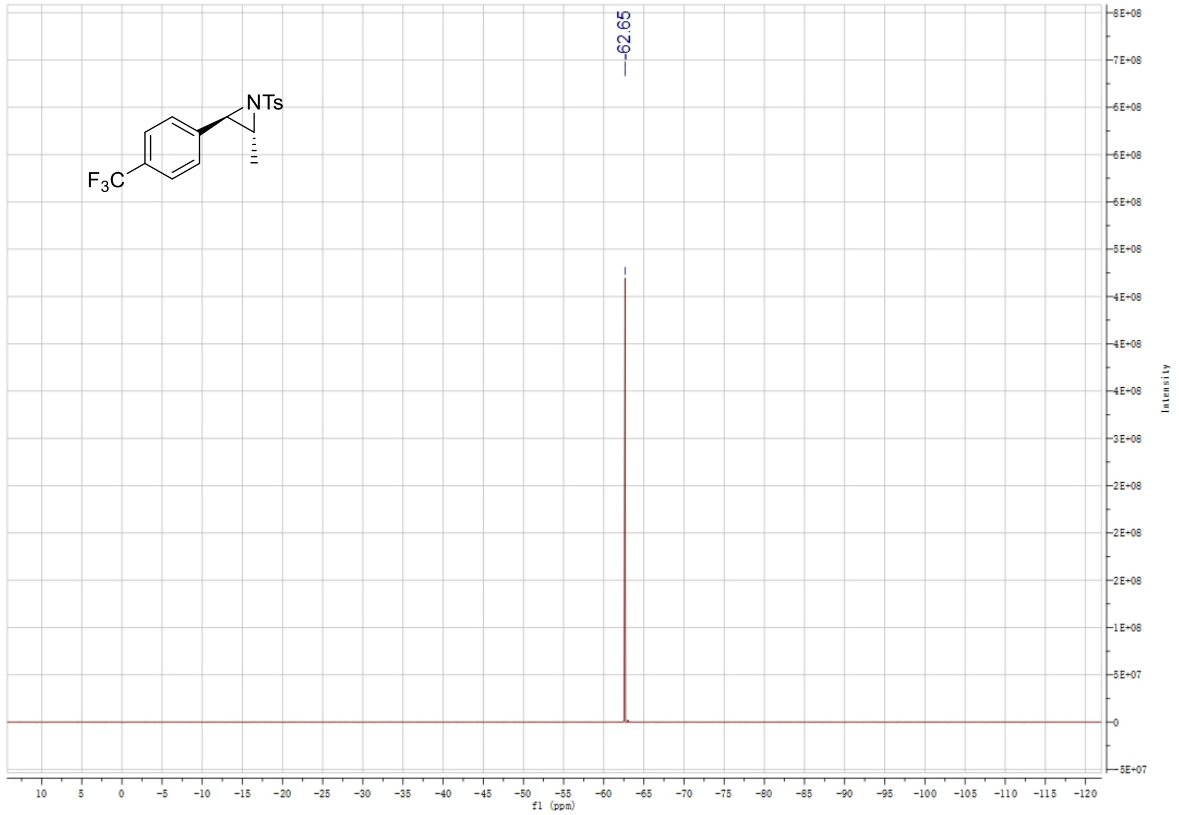

Supplementary Figure 73.

*(2R,3R)-2-Methyl-1-tosyl-3-(4-(trifluoromethoxy)phenyl)aziridine (**20d**)*

^{1}H NMR (600 MHz, 293 K, $CDCl_3$)

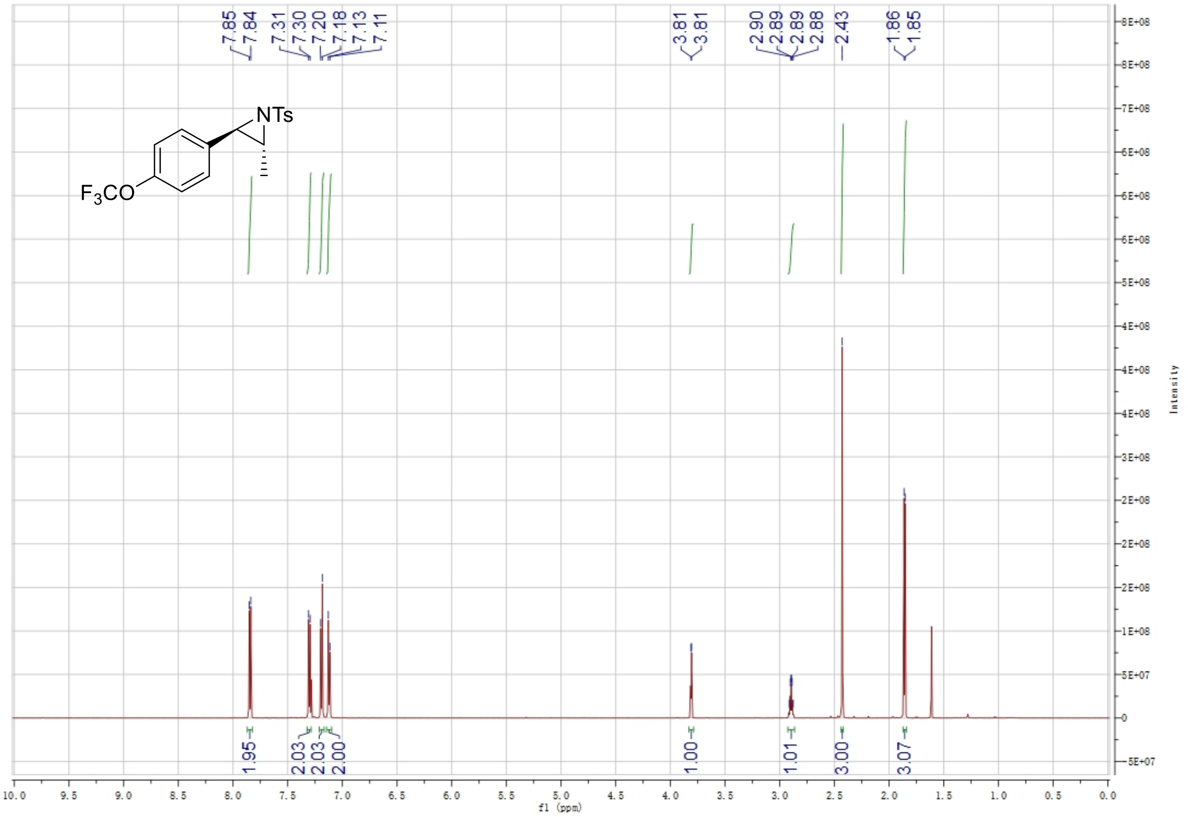

^{13}C NMR (151 MHz, 293 K, $CDCl_3$)

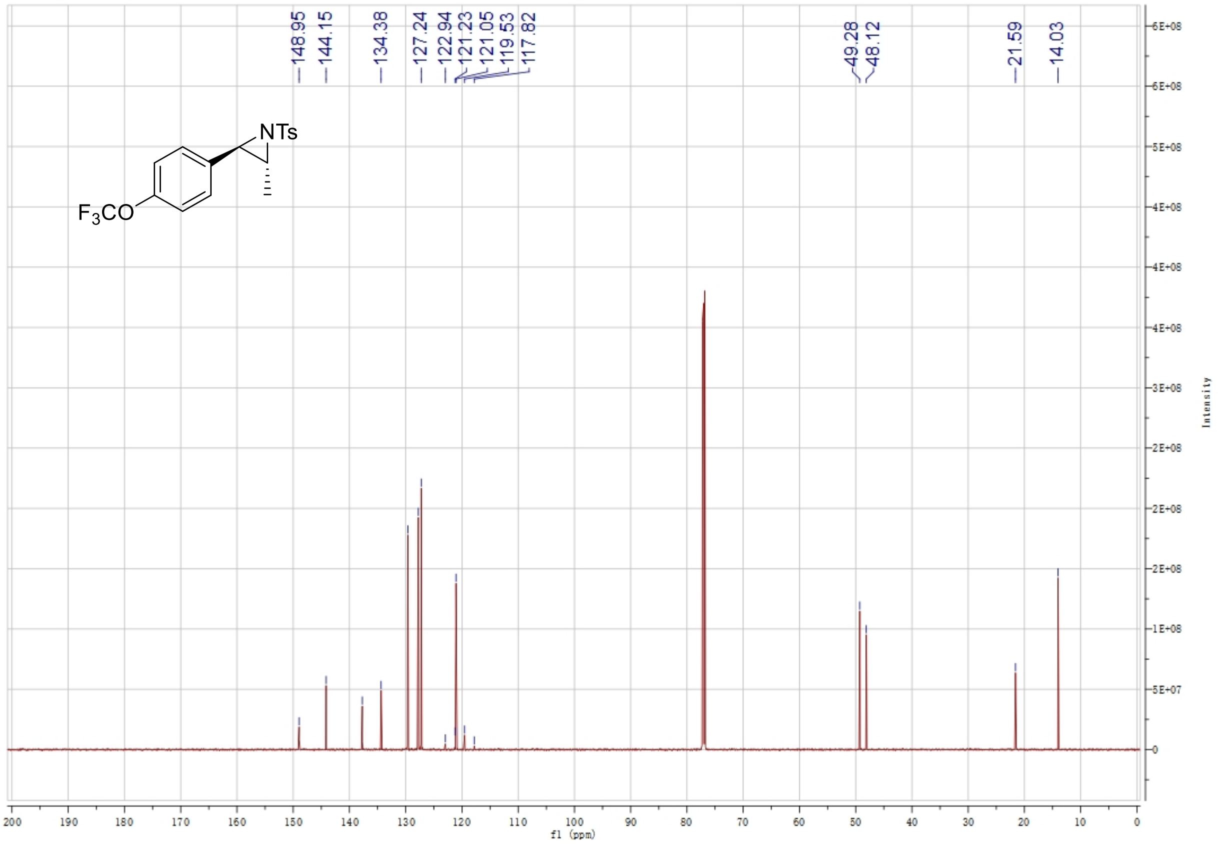

^{19}F NMR (565 MHz, 293 K, $CDCl_3$)

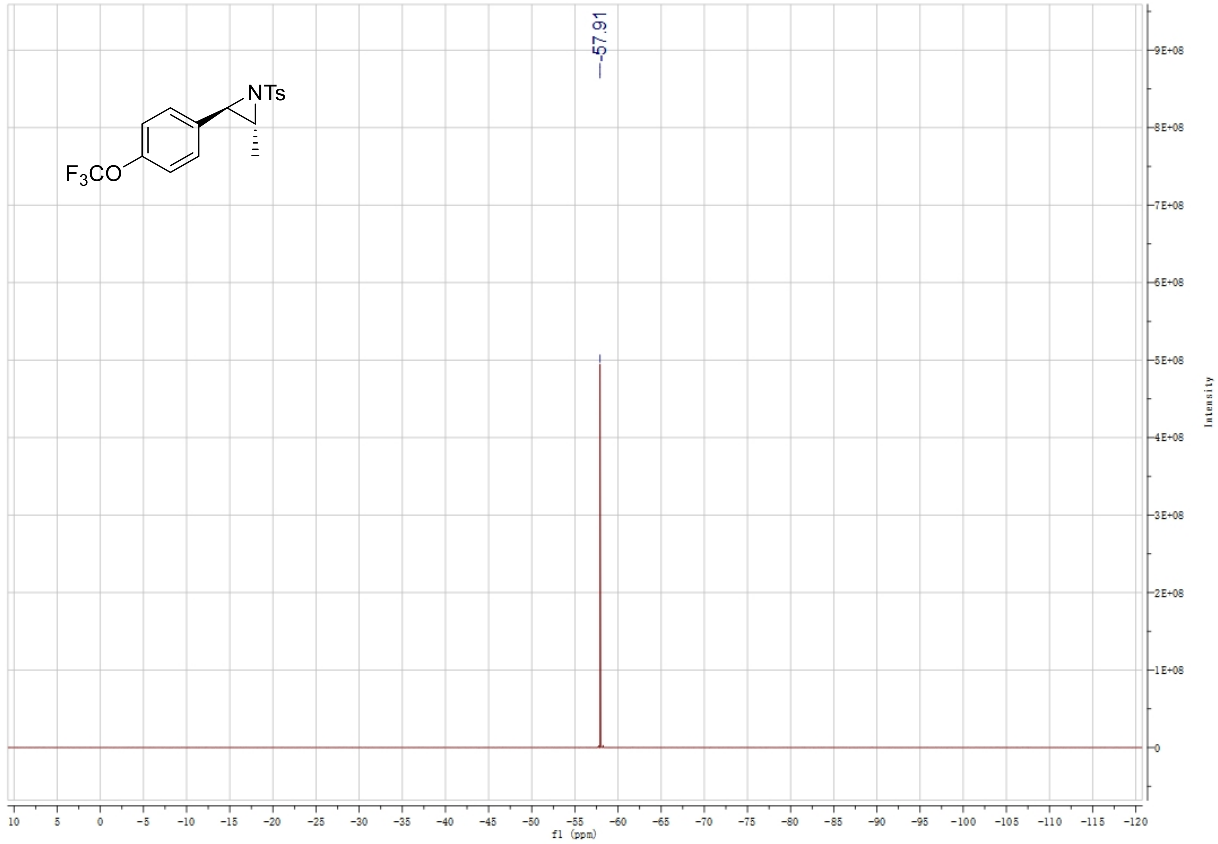

Supplementary Figure 74.
*(2R,3R)-2-Methyl-1-tosyl-3-(3-(trifluoromethoxy)phenyl)aziridine (**20e**)*
^{1}H NMR (600 MHz, 293 K, $CDCl_3$)

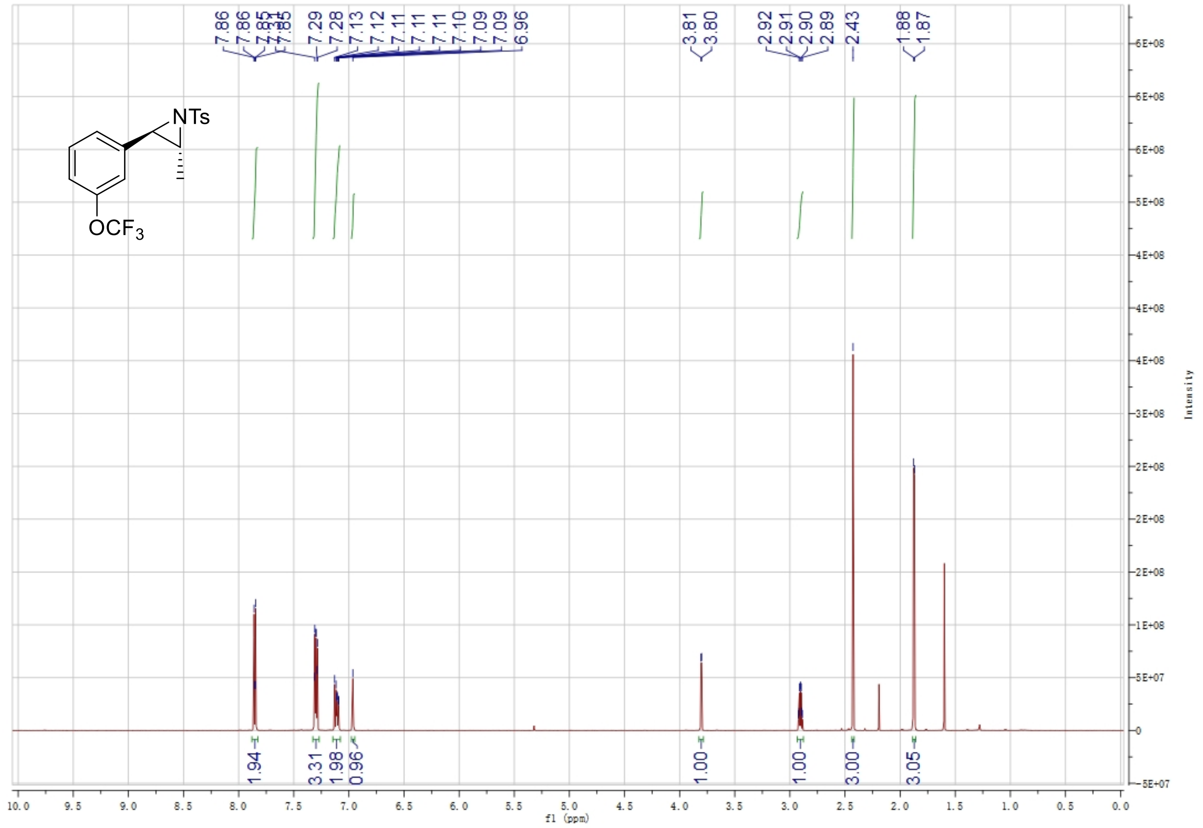

^{13}C NMR (151 MHz, 293 K, $CDCl_3$)

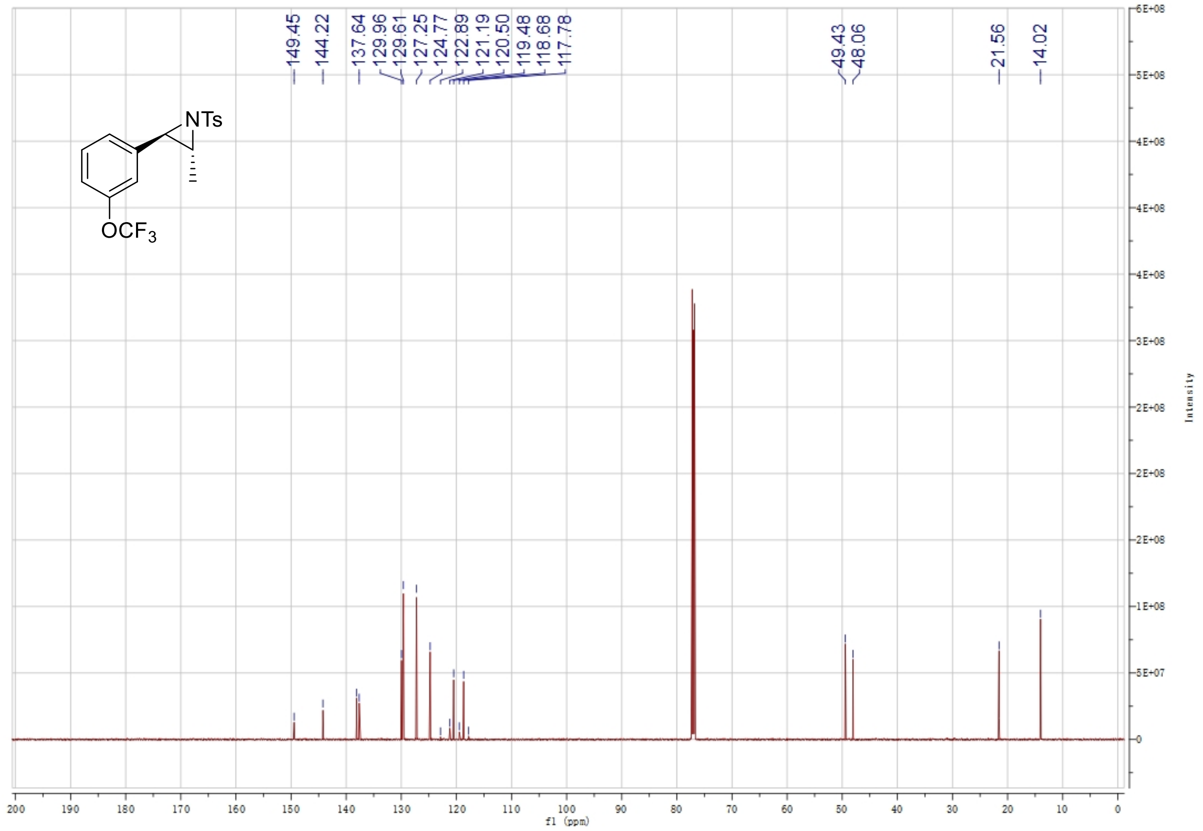

^{19}F NMR (565 MHz, 293 K, $CDCl_3$)

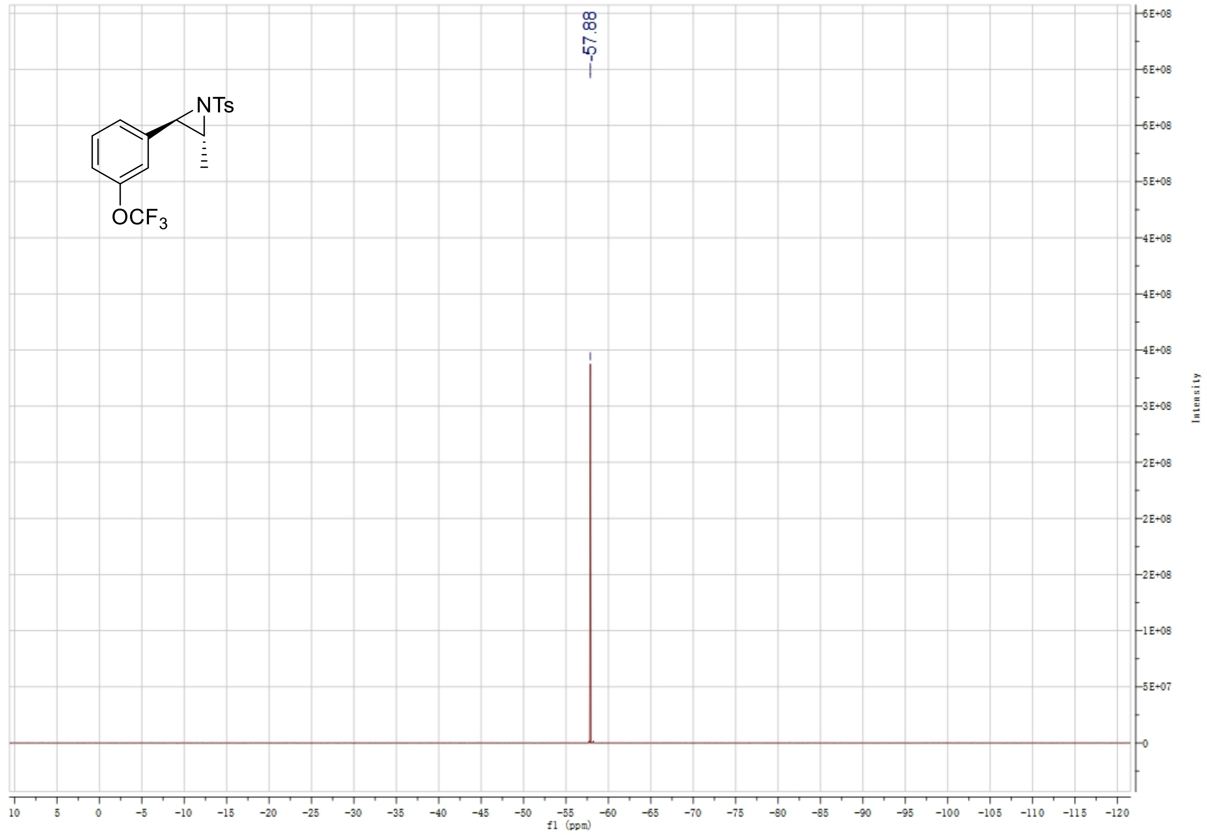

Supplementary Figure 75. *(2R,3R)-2-(2-Bromophenyl)-3-methyl-1-tosylaziridine (**20f**)*
^{1}H NMR (600 MHz, 293 K, $CDCl_3$)

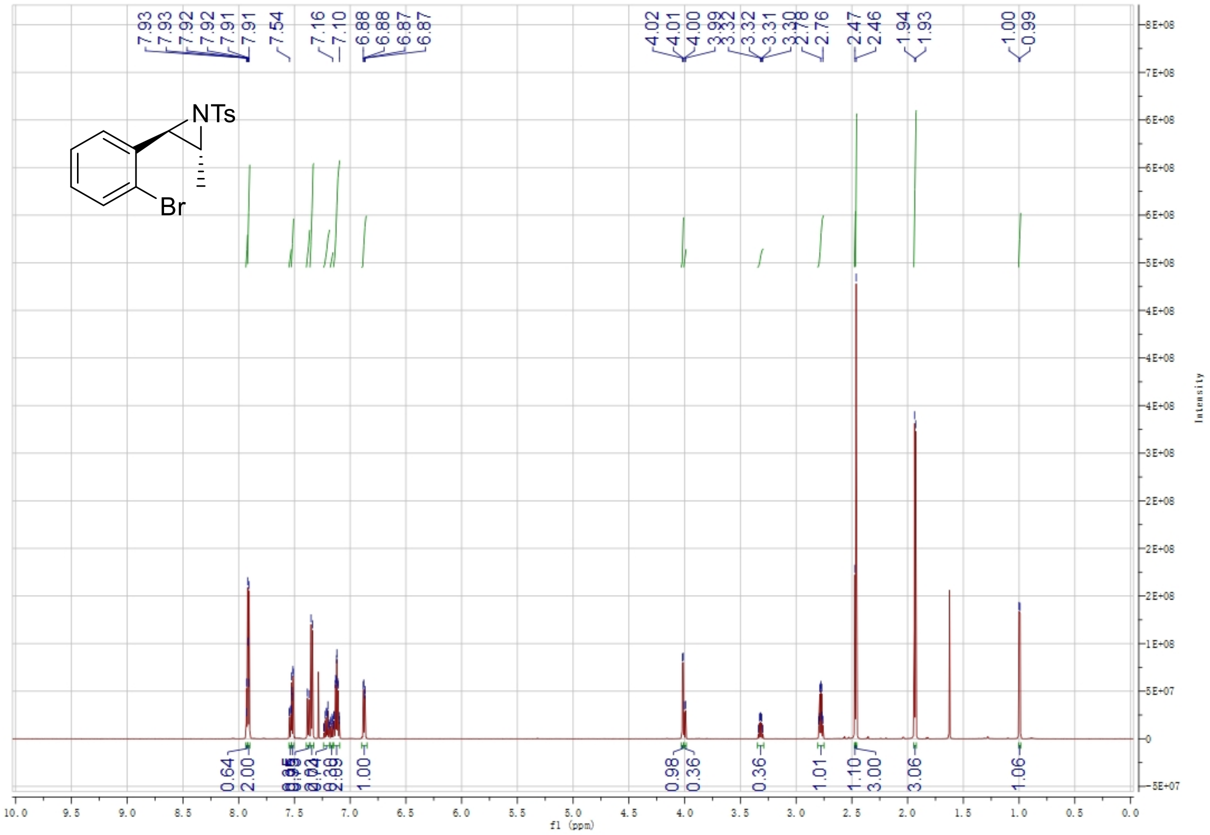

^{13}C NMR (151 MHz, 293 K, $CDCl_3$)

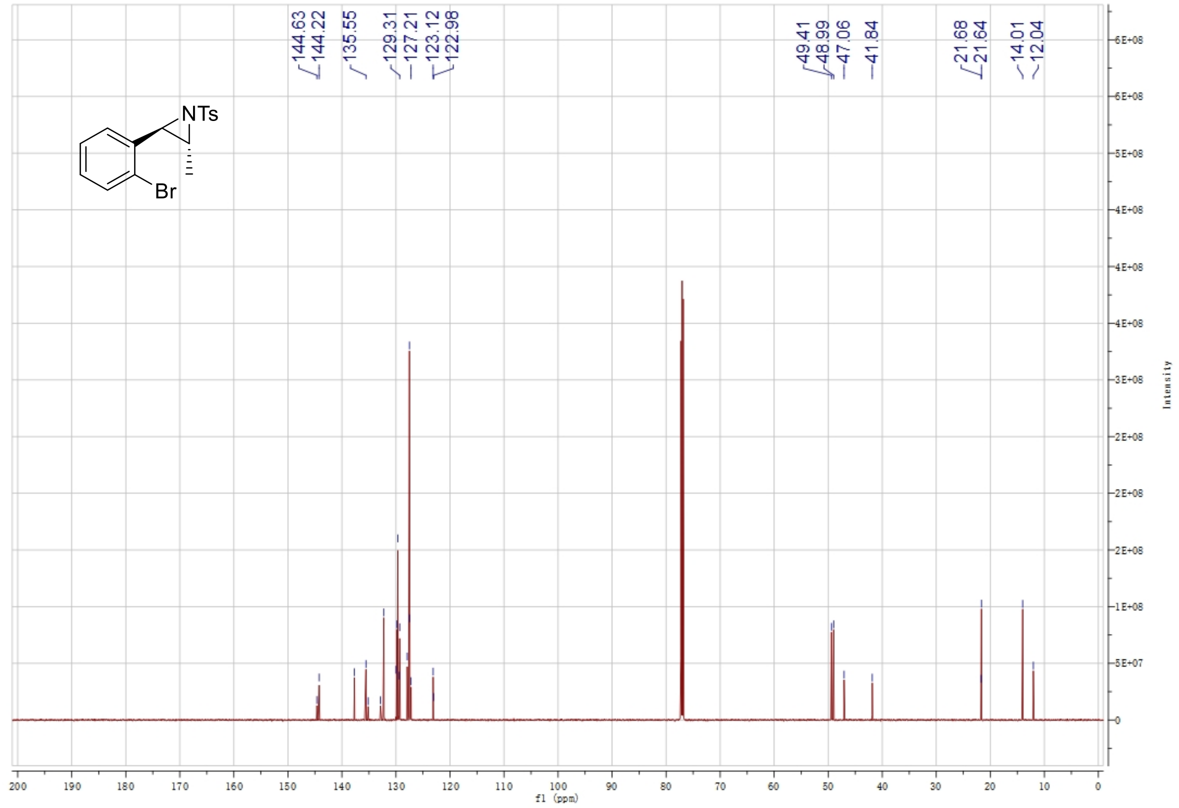

Supplementary Figure 76. *(2R,3R)-2-Ethyl-3-phenyl-1-tosylaziridine (**20g**)*

^{1}H NMR (600 MHz, 293 K, $CDCl_3$)

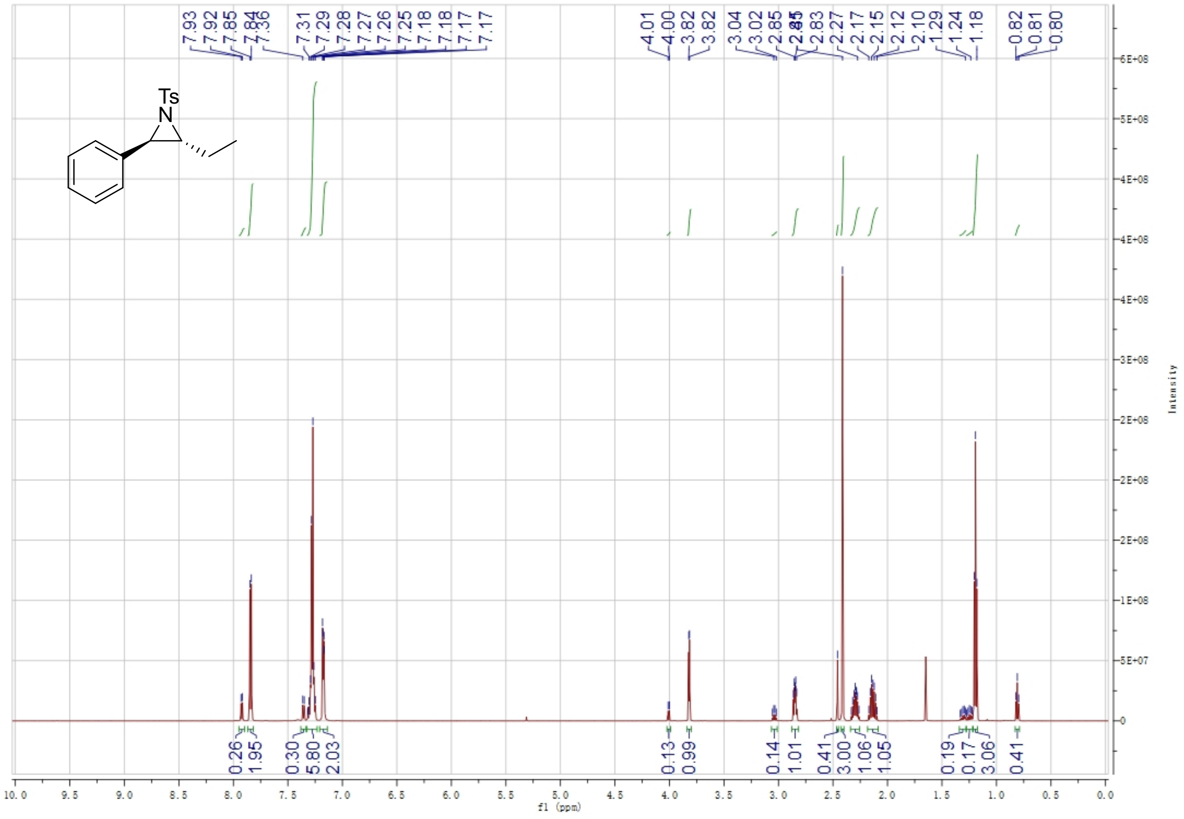

^{13}C NMR (151 MHz, 293 K, $CDCl_3$)

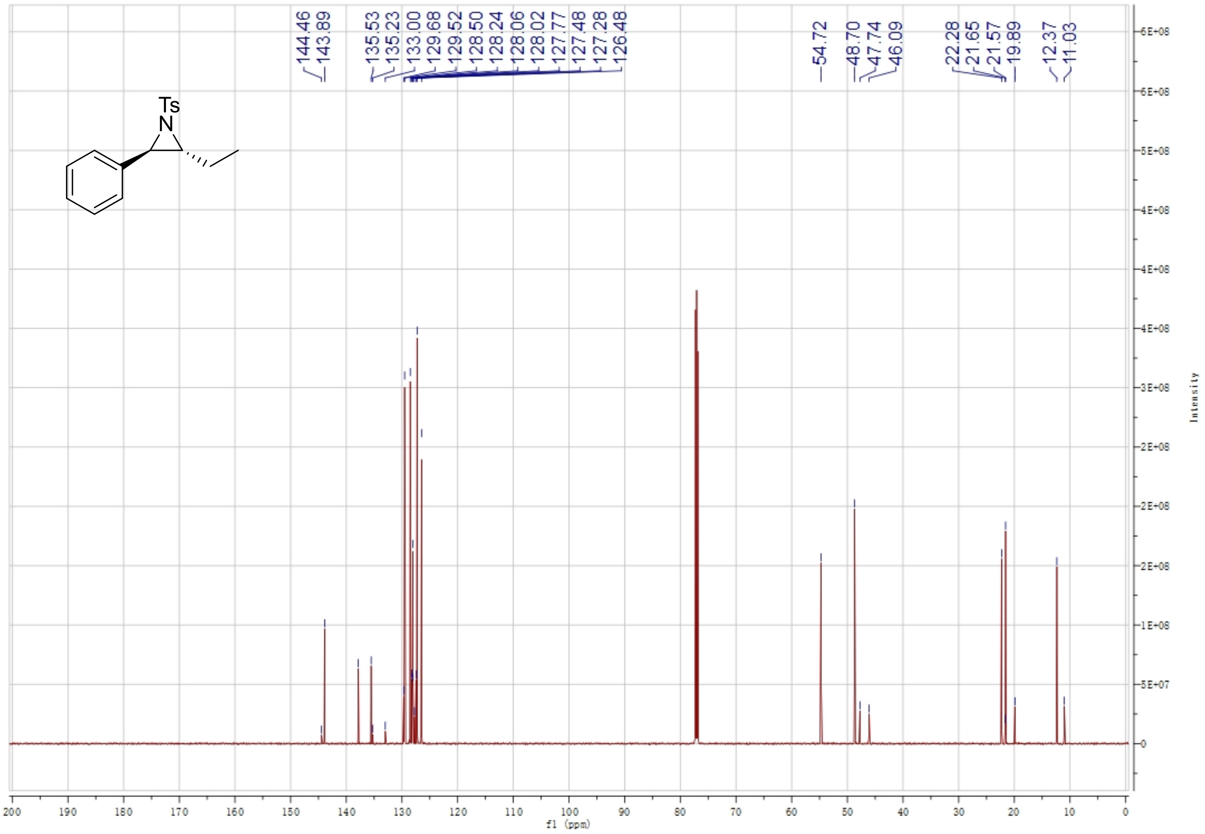

Supplementary Figure 77. *(2S,3S)-2,3-Dibutyl-1-tosylaziridine (**20h**)*

^{1}H NMR (600 MHz, 293 K, $CDCl_3$)

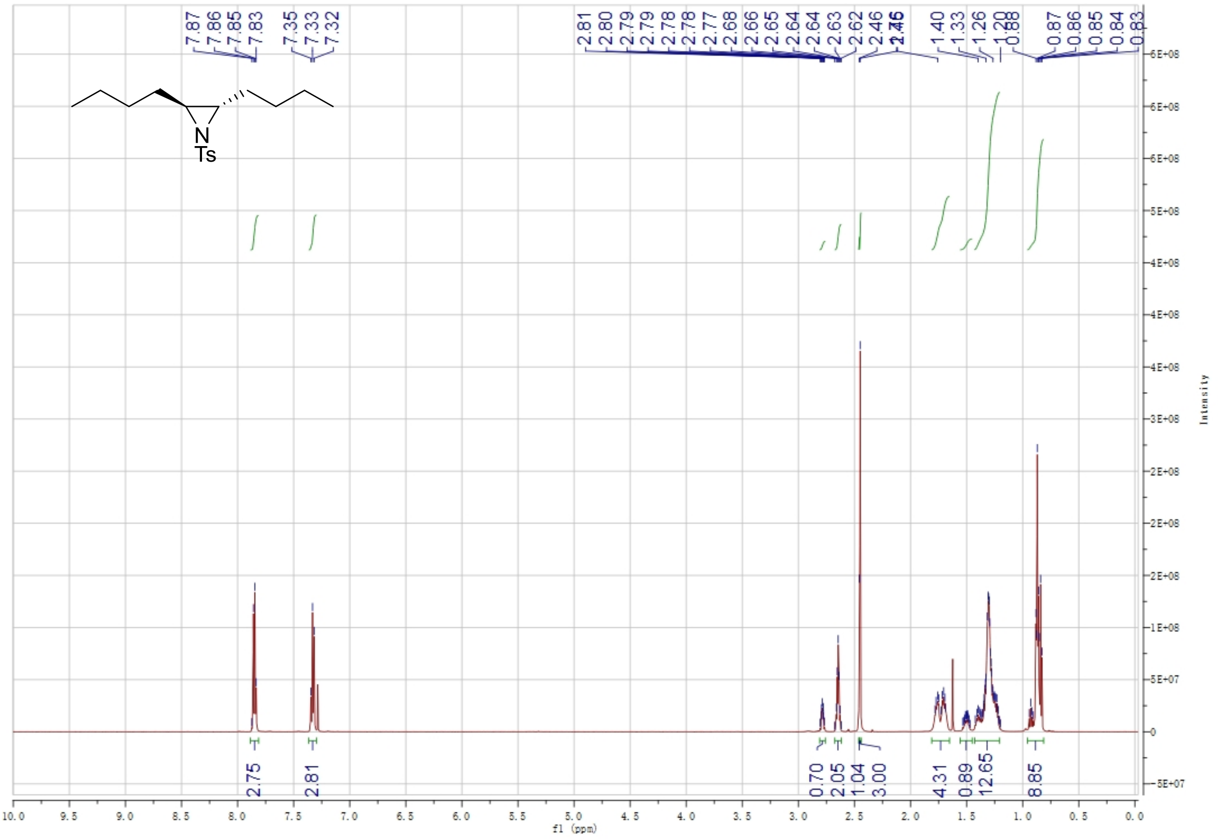

^{13}C NMR (151 MHz, 293 K, $CDCl_3$)

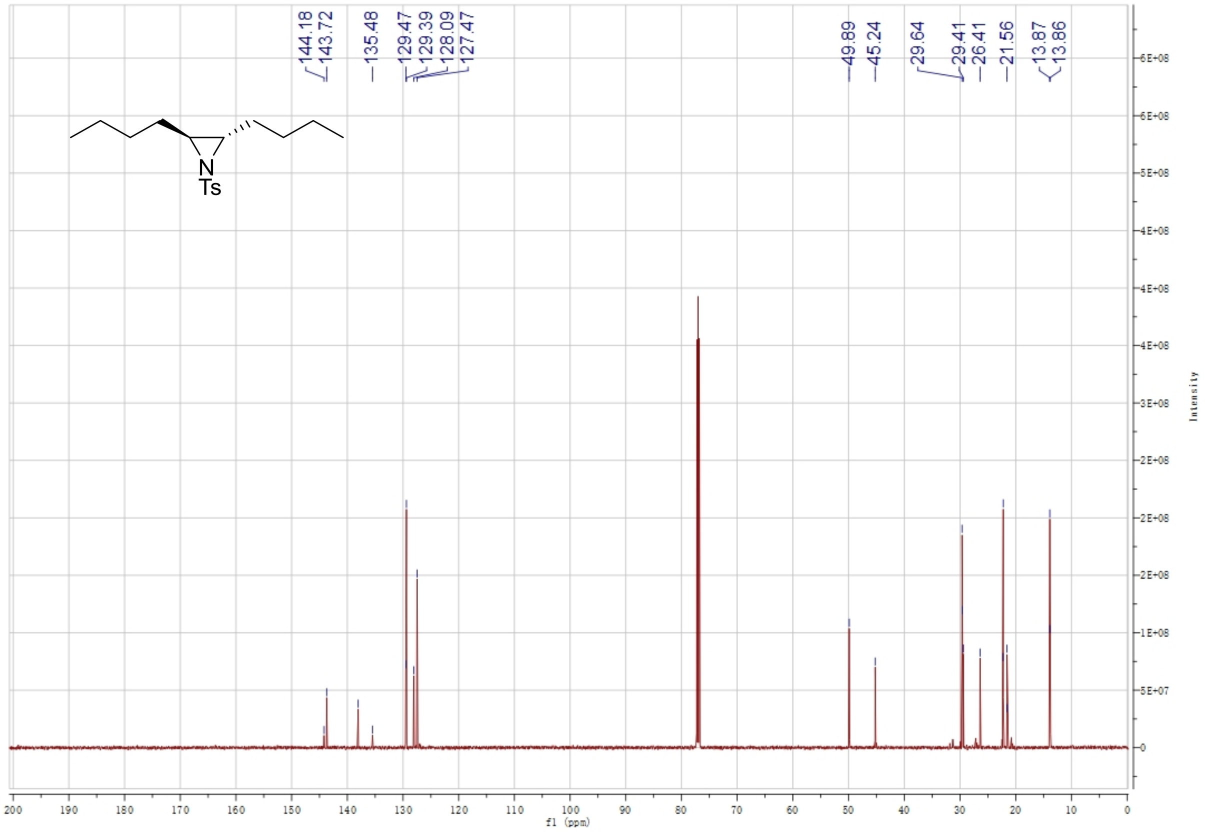

Supplementary Figure 78. *(2S,3S)-2-Isopropyl-3-methyl-1-tosylaziridine (**20i**)*

^{1}H NMR (600 MHz, 293 K, $CDCl_3$)

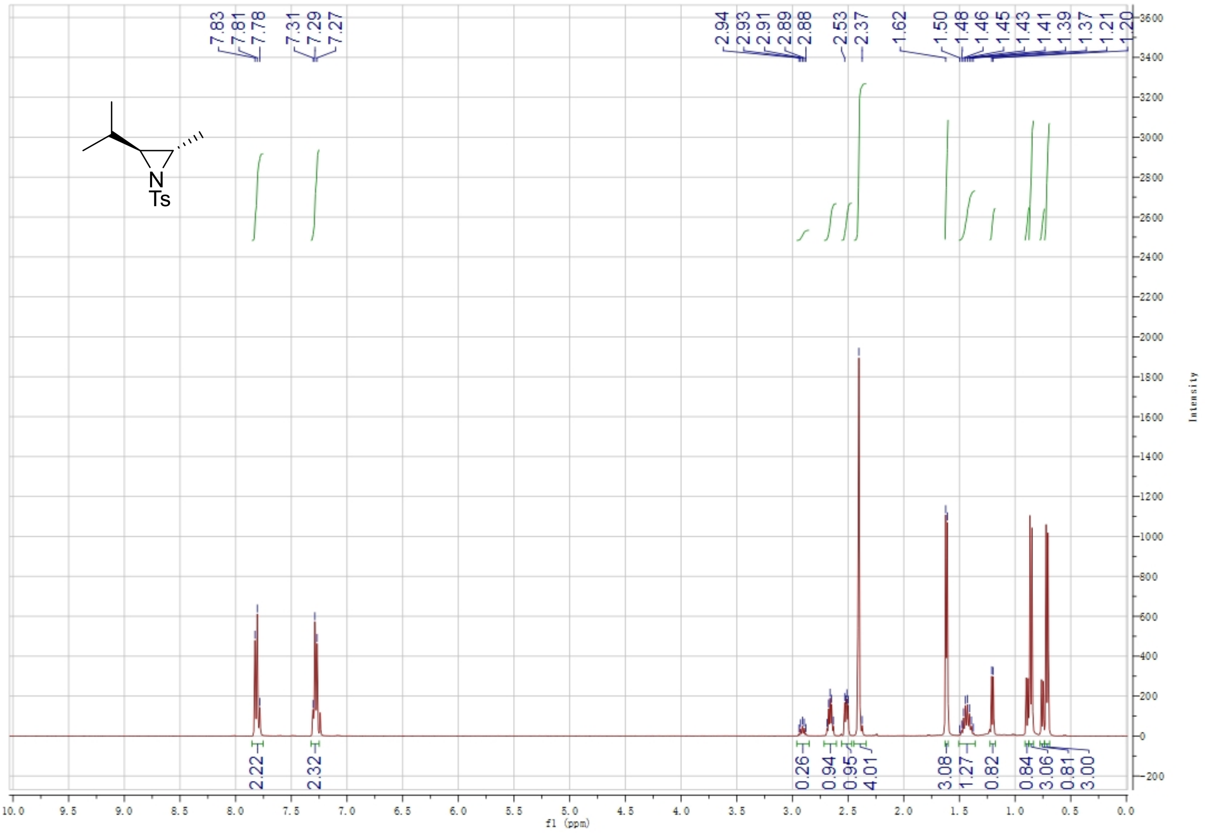

^{13}C NMR (151 MHz, 293 K, $CDCl_3$)

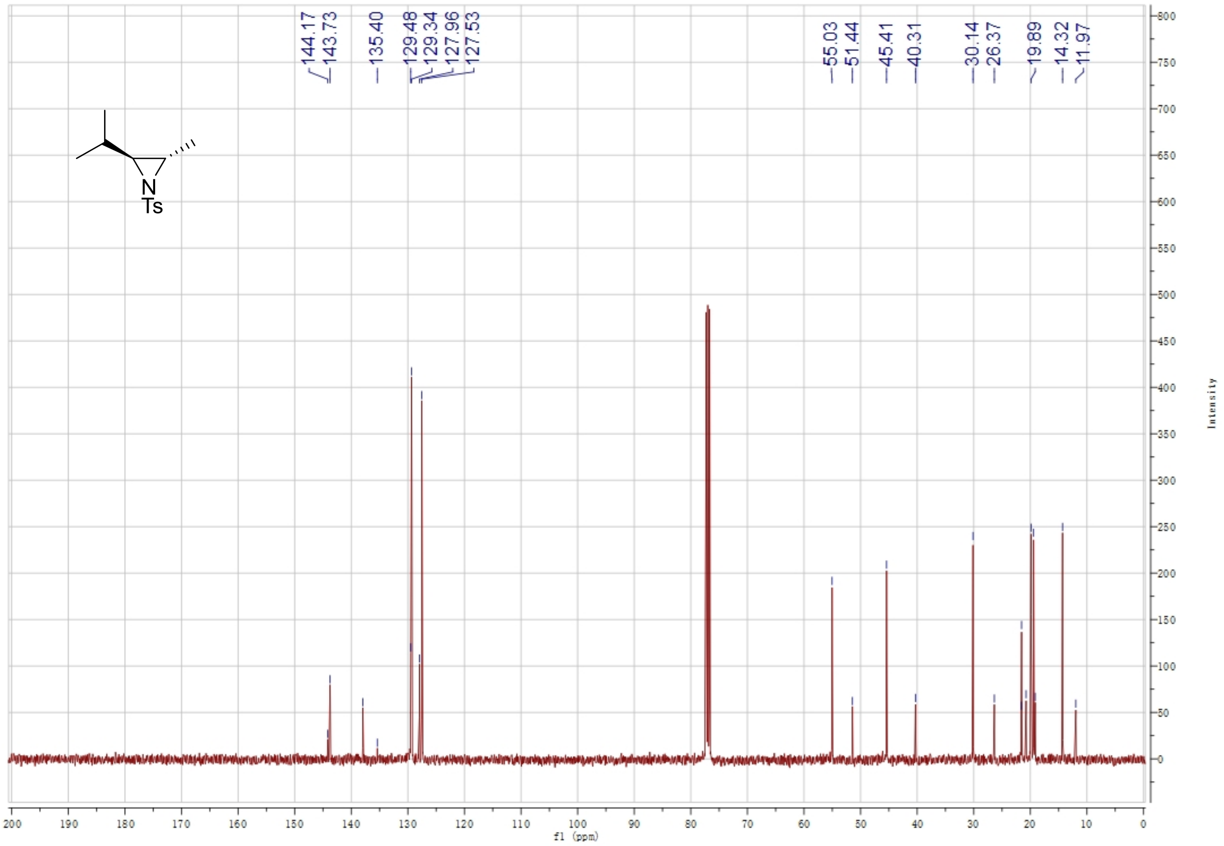

Supplementary Figure 79. *2-Phenyl-1-tosylaziridine (**22a**)*

^{1}H NMR (300 MHz, 293 K, $CDCl_3$)

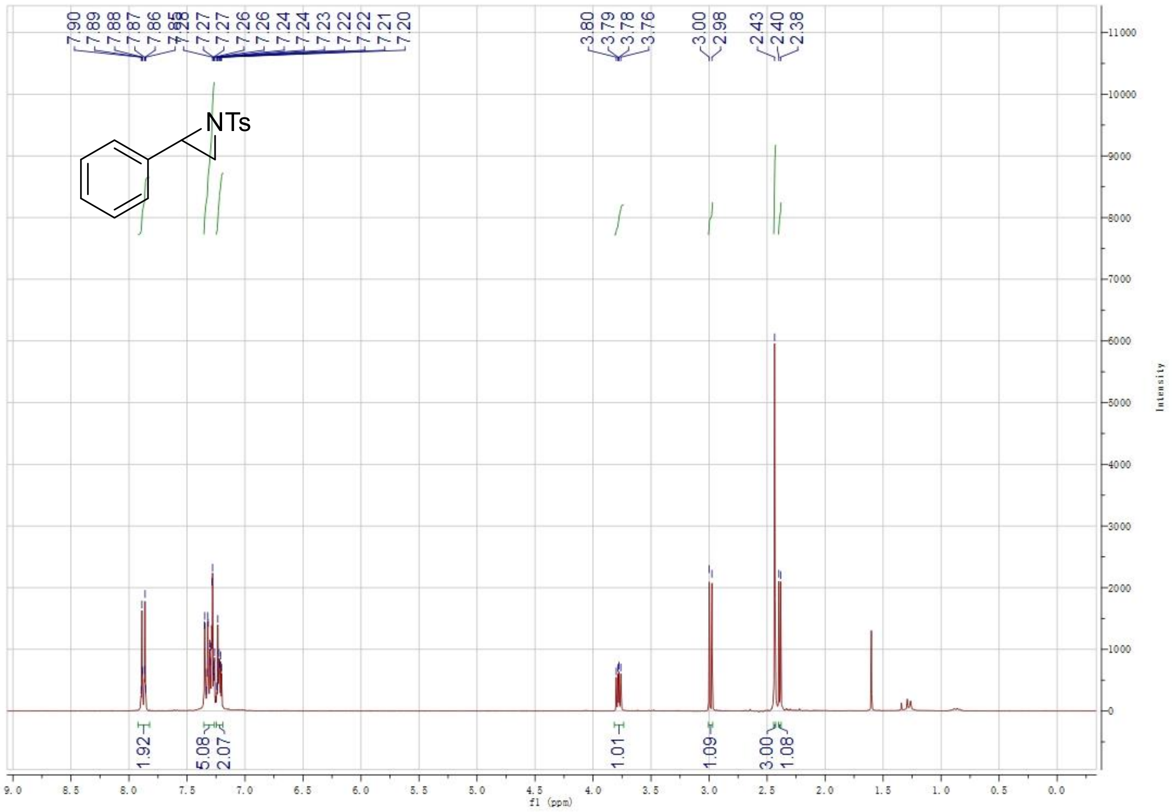

^{13}C NMR (101 MHz, 293 K, $CDCl_3$)

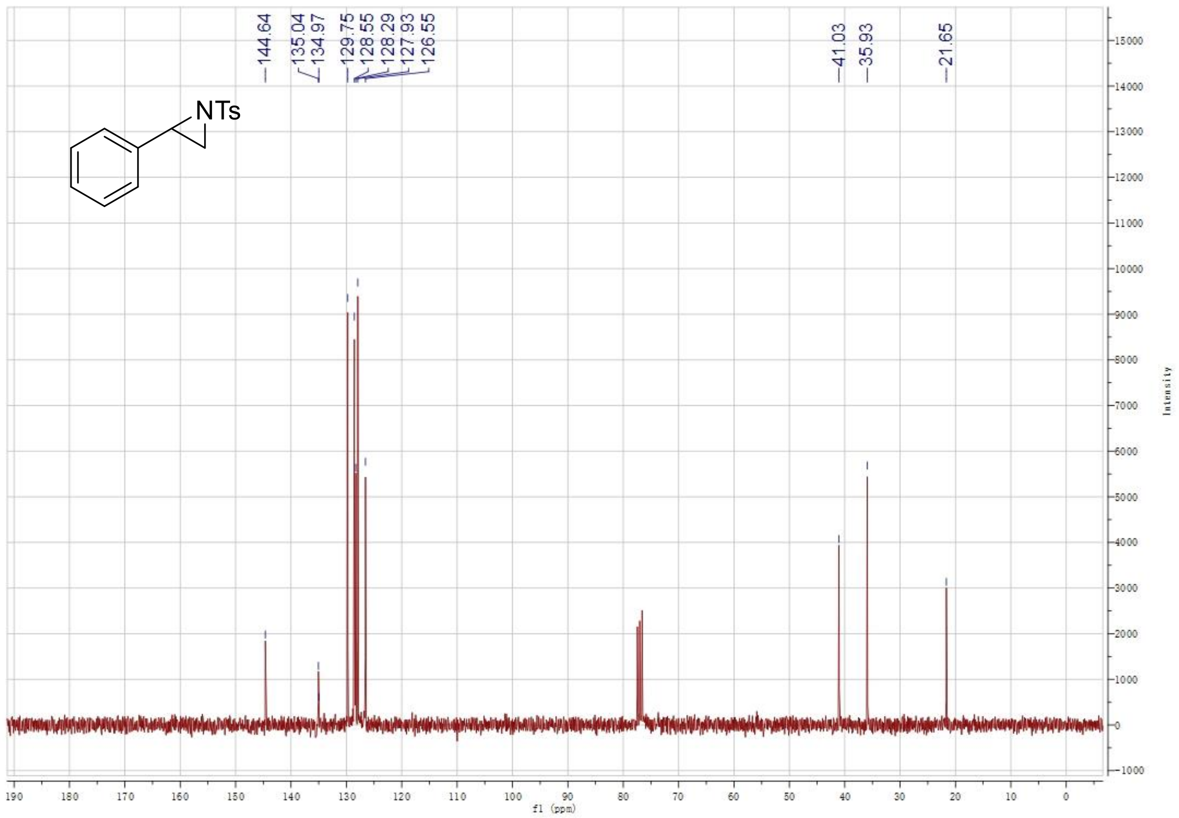

Supplementary Figure 80. *1-((4-Chlorophenyl)sulfonyl)-2-phenylaziridine (**22b**)*
^{1}H NMR (400 MHz, 293 K, $CDCl_3$)

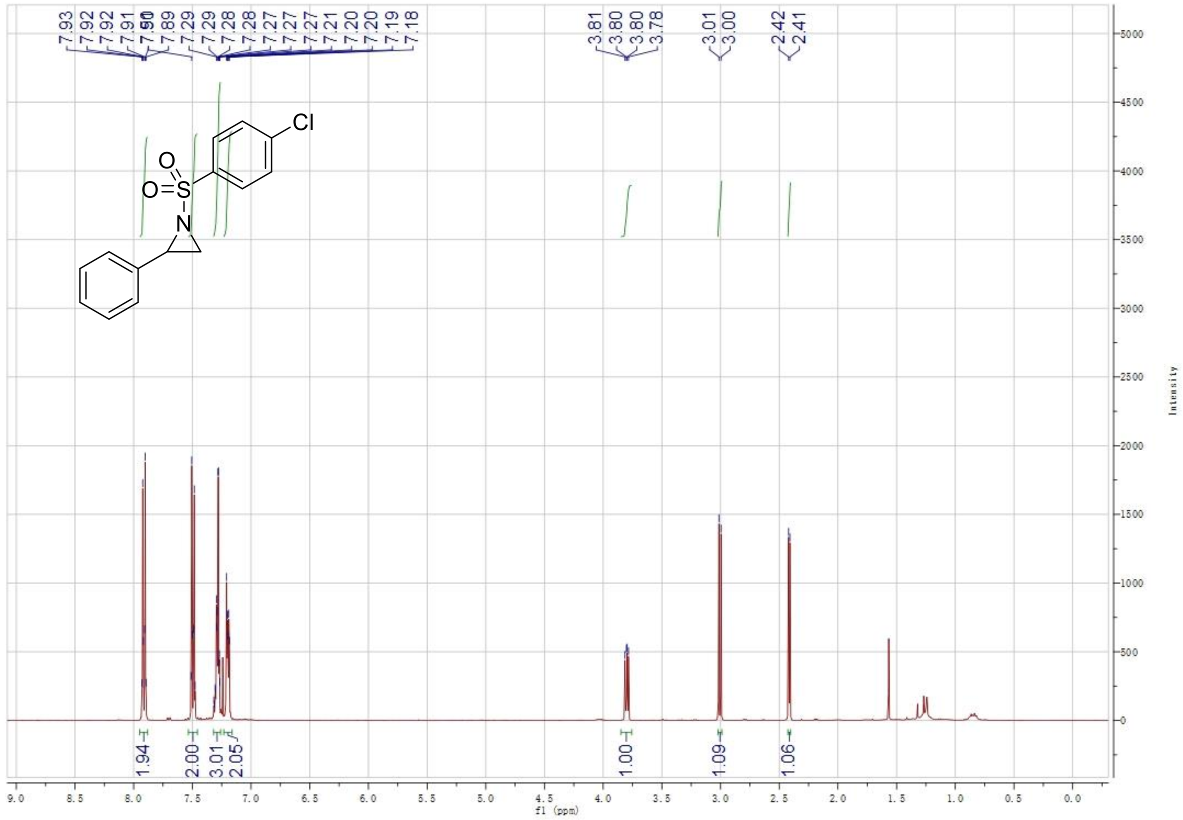

^{13}C NMR (101 MHz, 293 K, $CDCl_3$)

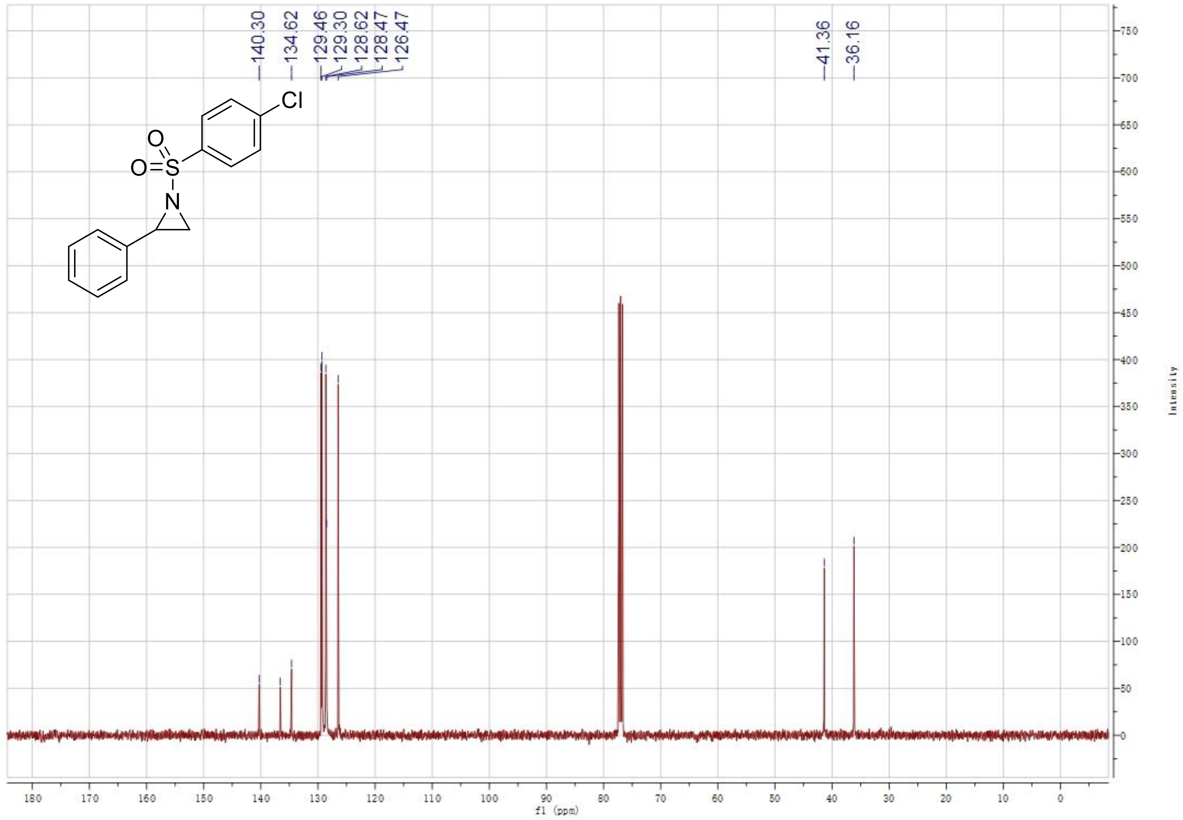

Supplementary Figure 81. *1-((3,5-Bis(Trifluoromethyl)phenyl)sulfonyl)-2-phenylaziridine **(22c)***

^{1}H NMR (600 MHz, 293 K, $CDCl_3$)

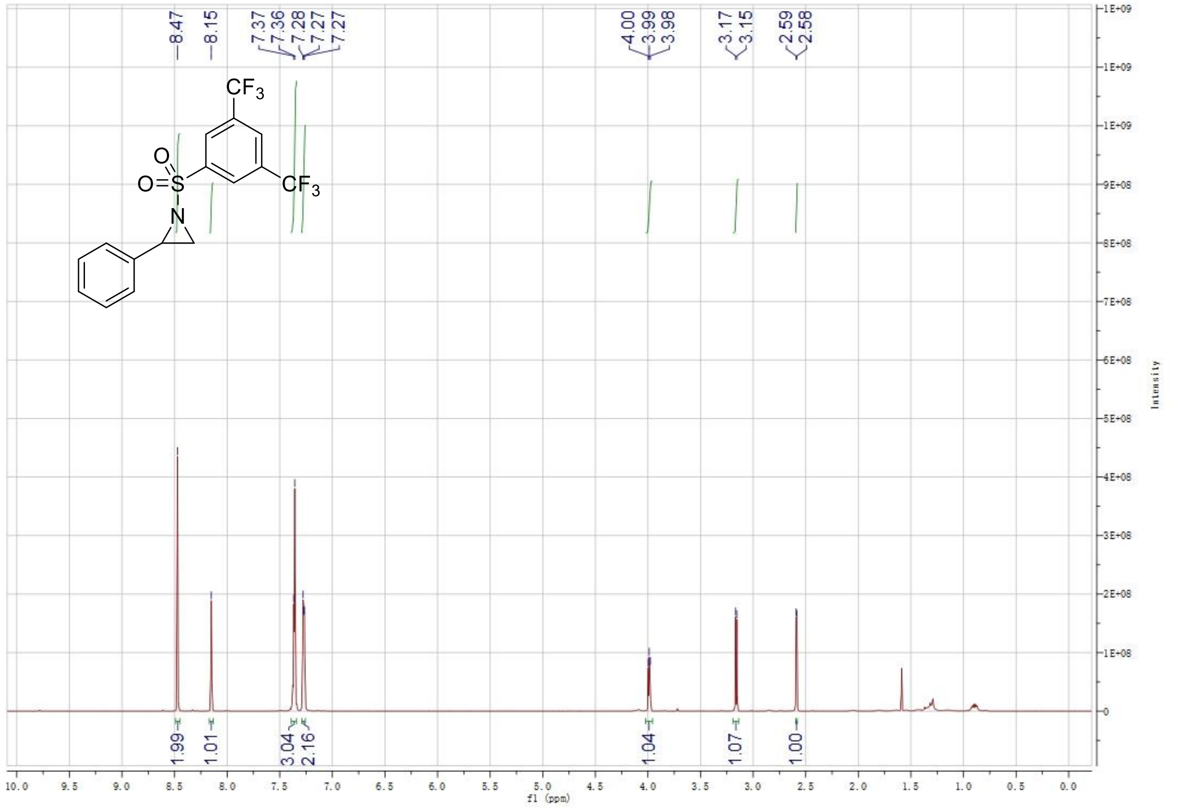

^{13}C NMR (151 MHz, 293 K, $CDCl_3$)

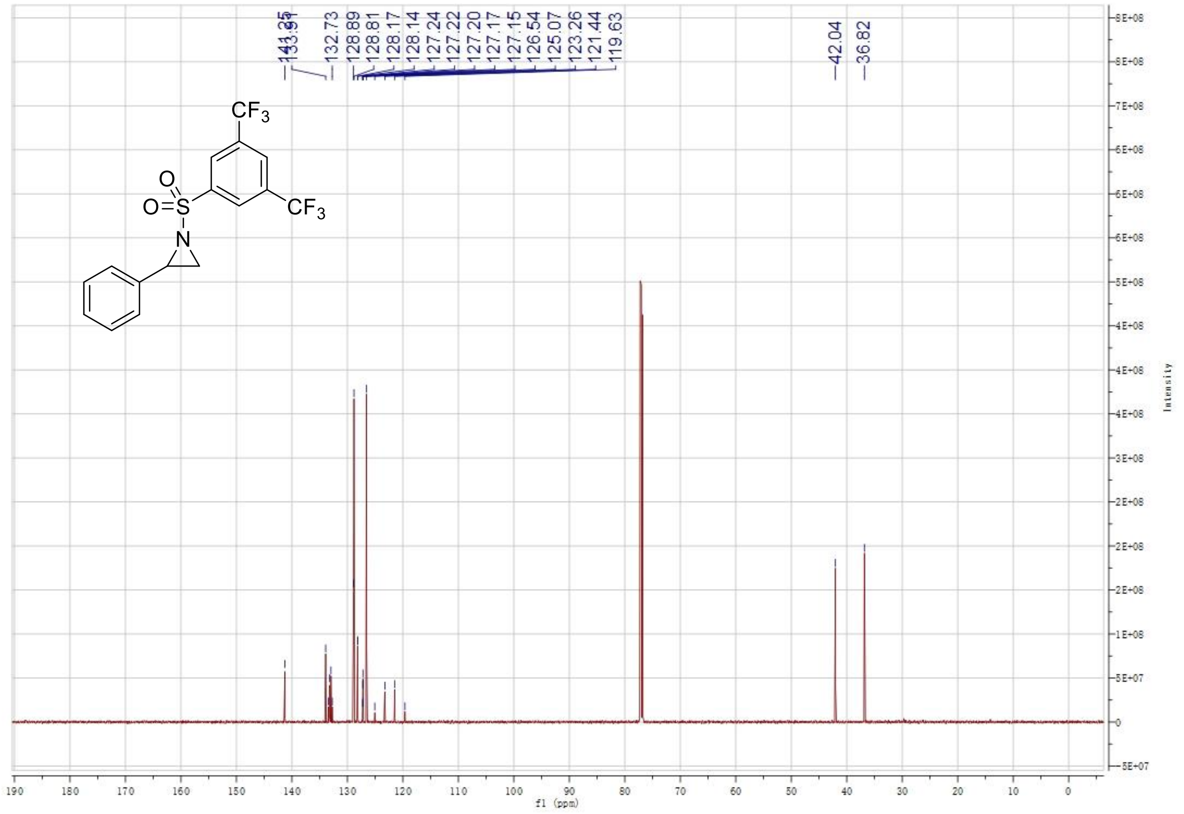

^{19}F NMR (565 MHz, 293 K, $CDCl_3$)

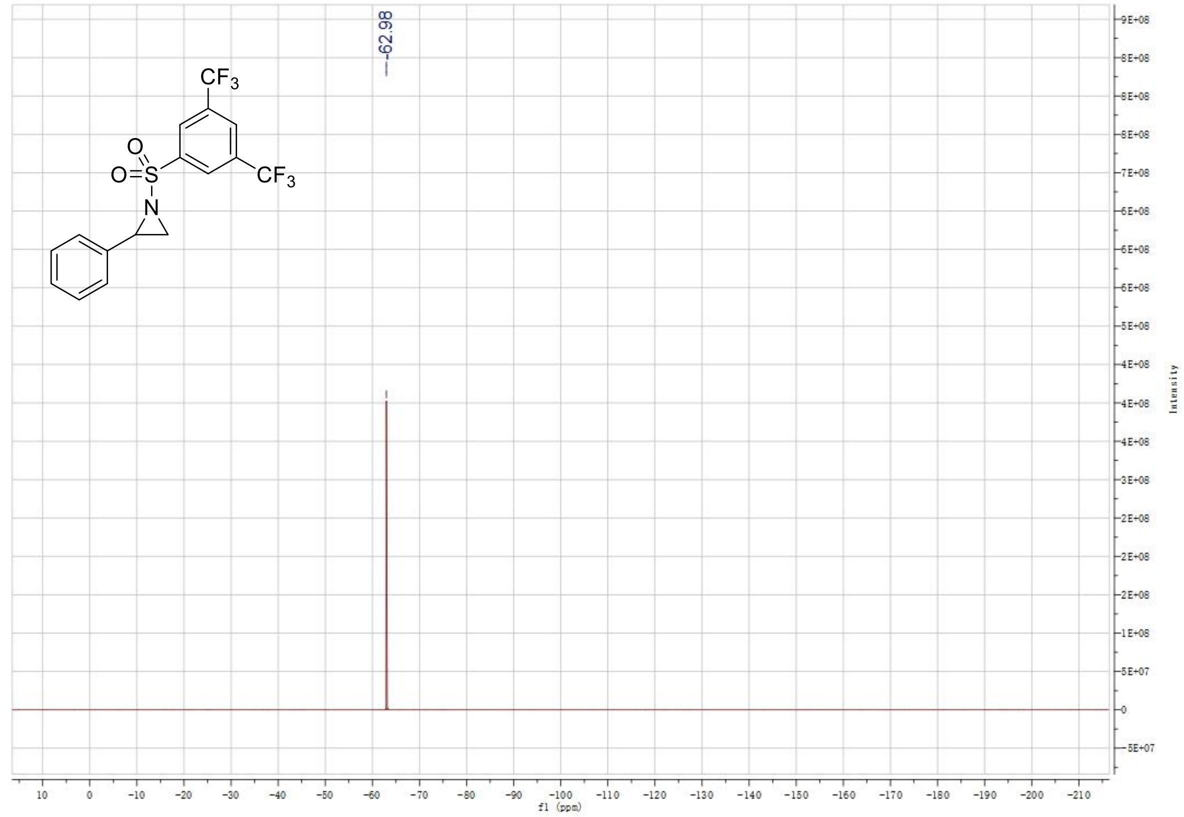

Supplementary Figure 82. *1-((3-Nitrophenyl)sulfonyl)-2-phenylaziridine (**22d**)*

^{1}H NMR (600 MHz, 293 K, $CDCl_3$)

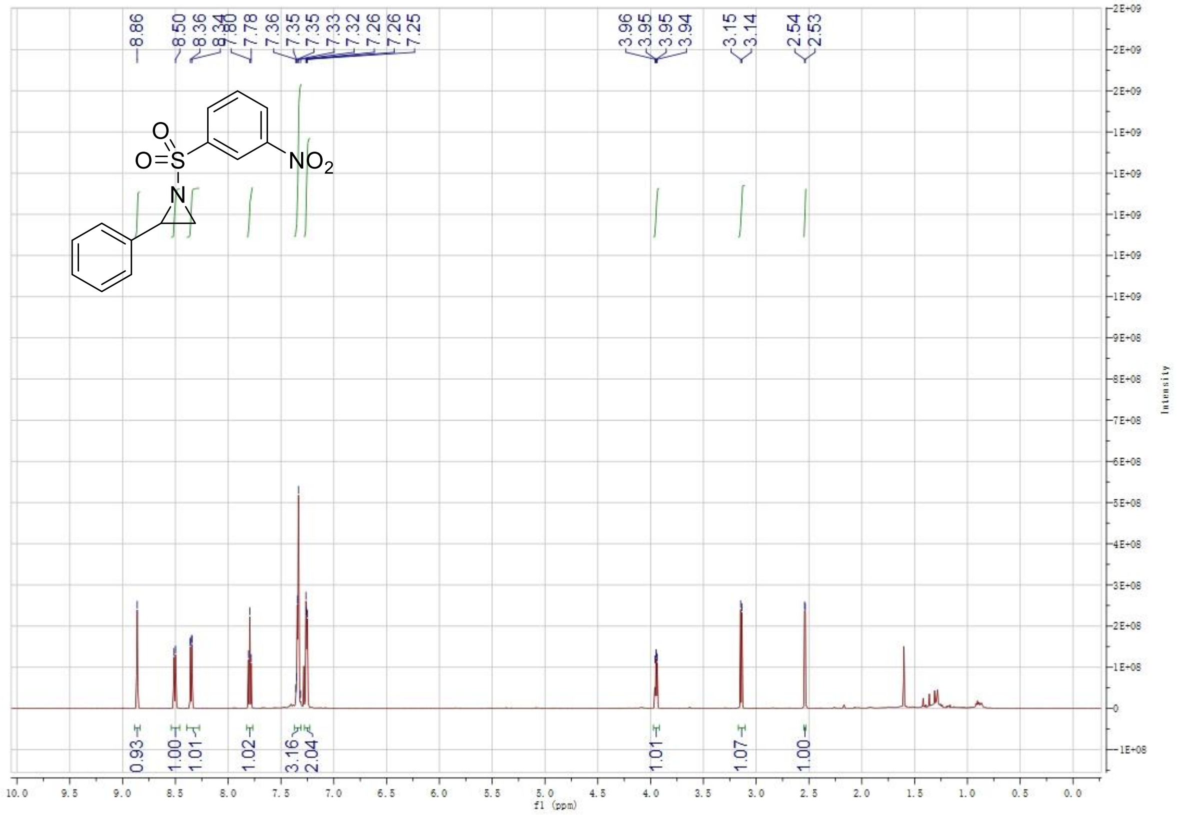

^{13}C NMR (151 MHz, 293 K, $CDCl_3$)

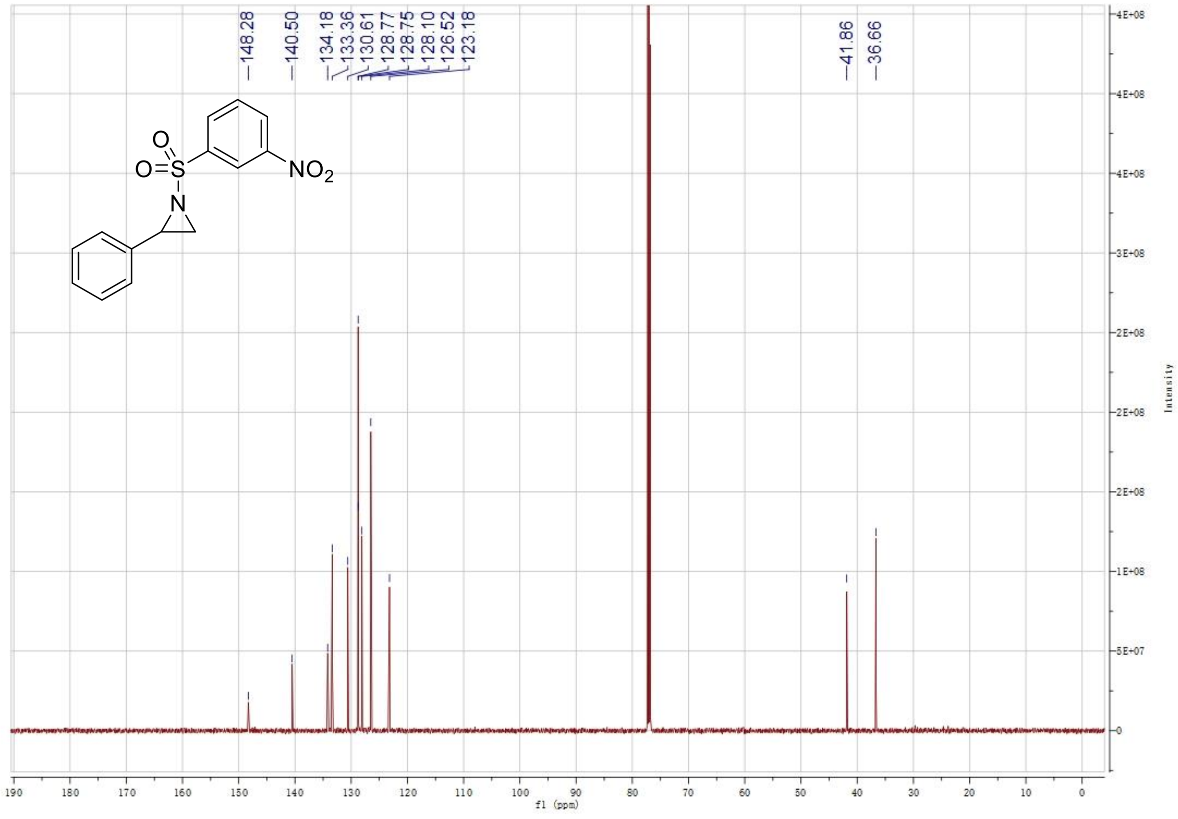

Supplementary Figure 83. *1-((2-Nitrophenyl)sulfonyl)-2-phenylaziridine (**22e**)*

^{1}H NMR (600 MHz, 293 K, $CDCl_3$)

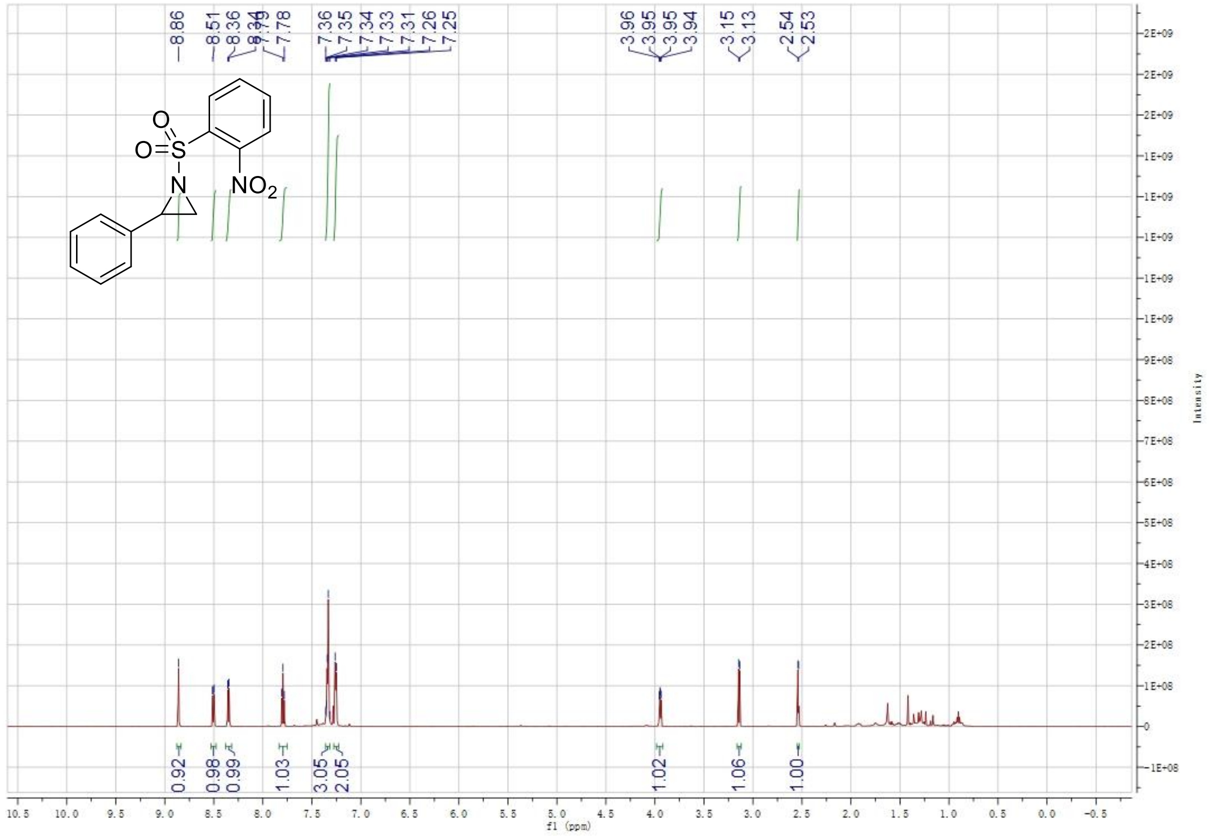

^{13}C NMR (151 MHz, 293 K, $CDCl_3$)

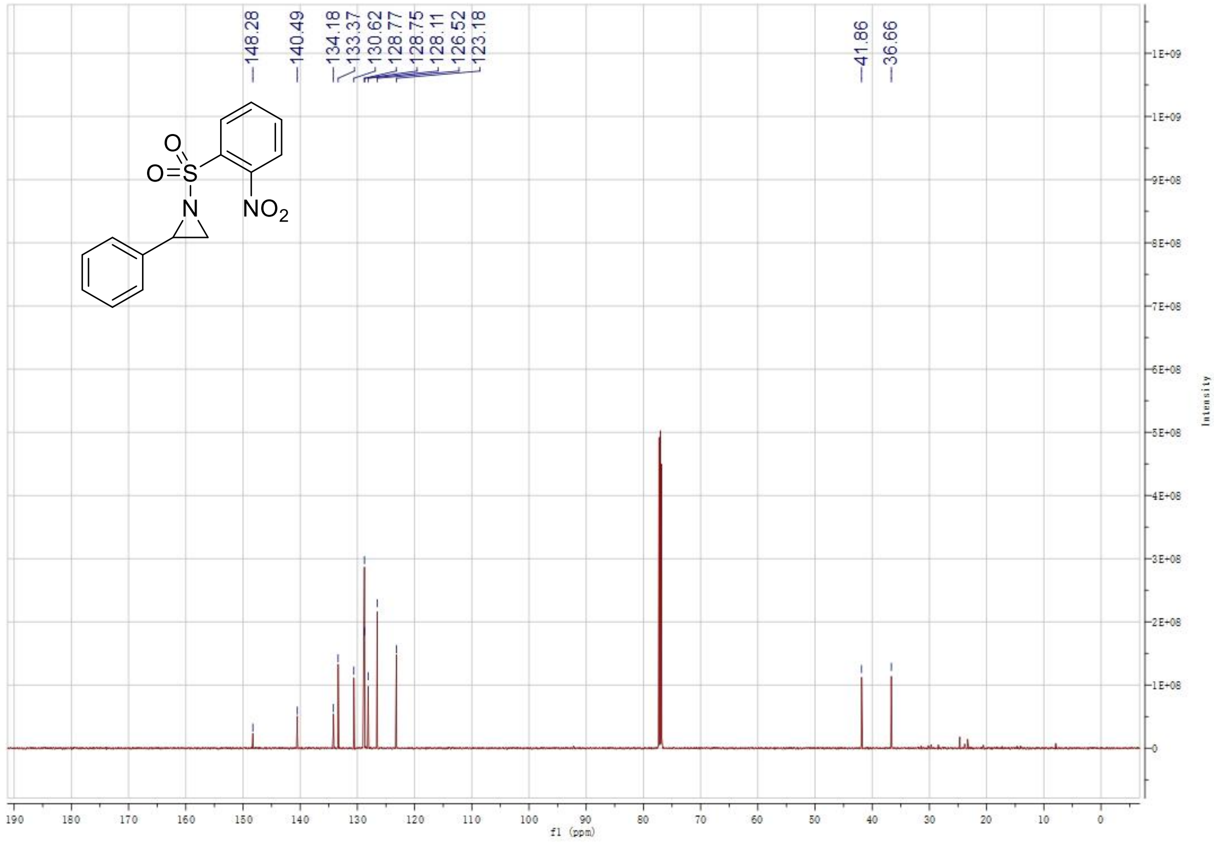

Supplementary Figure 84. *2-Phenyl-1-(o-tolylsulfonyl)aziridine (**22f**))*

^{1}H NMR (600 MHz, 293 K, $CDCl_3$)

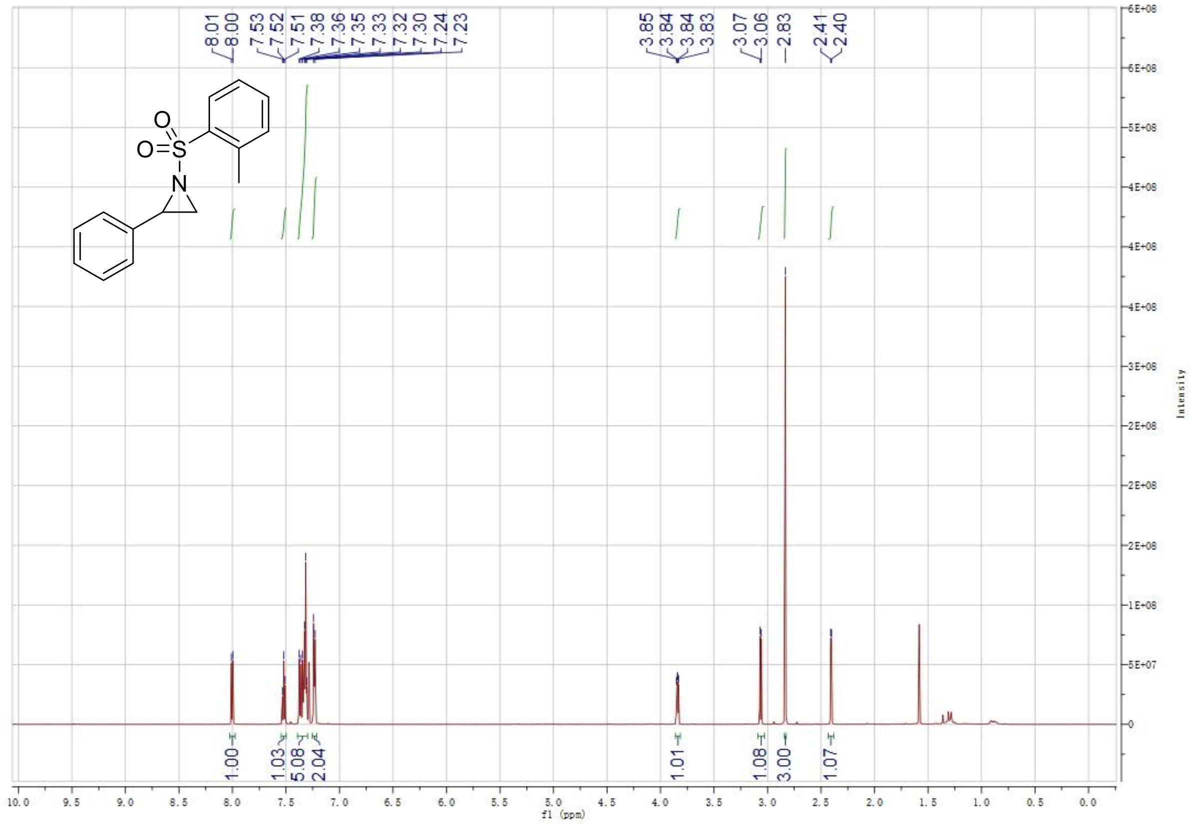

^{13}C NMR (151 MHz, 293 K, $CDCl_3$)

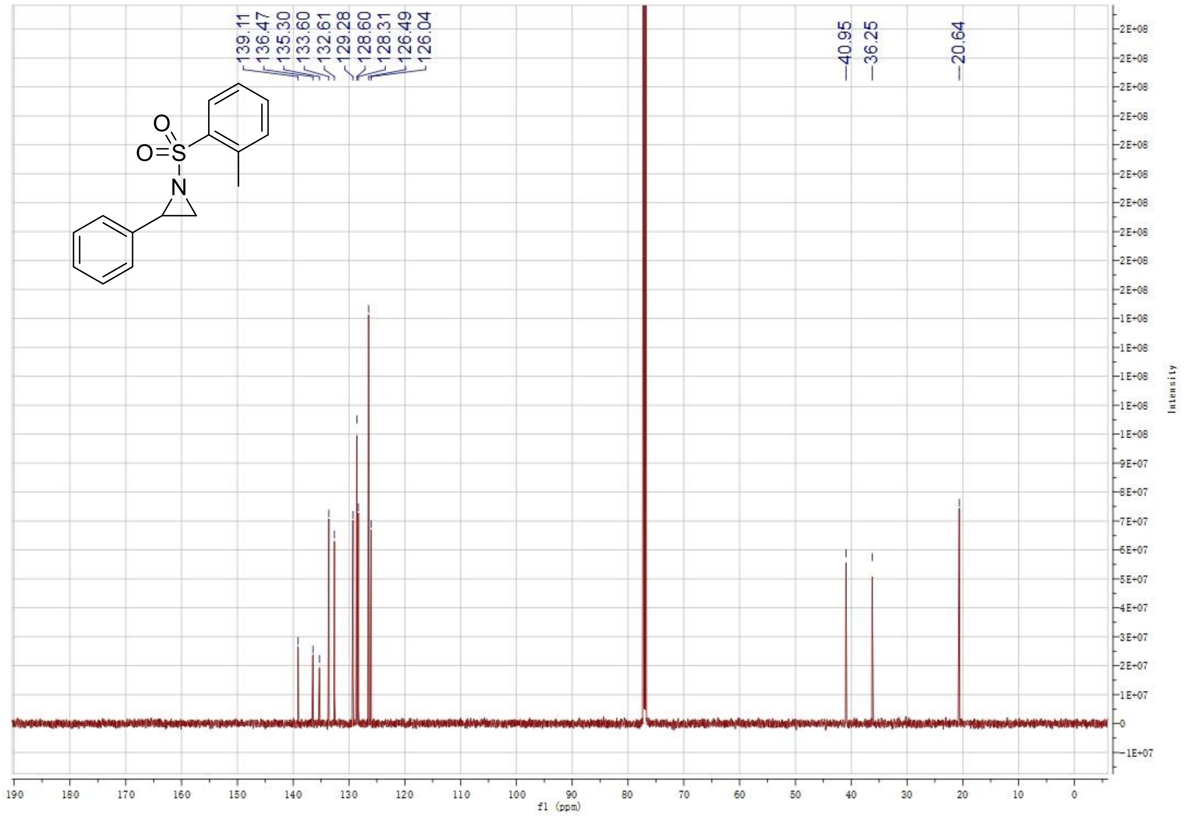

Supplementary Figure 85. *1-([1,1'-Biphenyl]-4-ylsulfonyl)-2-phenylaziridine* (***22g***)

^{1}H NMR (600 MHz, 293 K, $CDCl_3$)

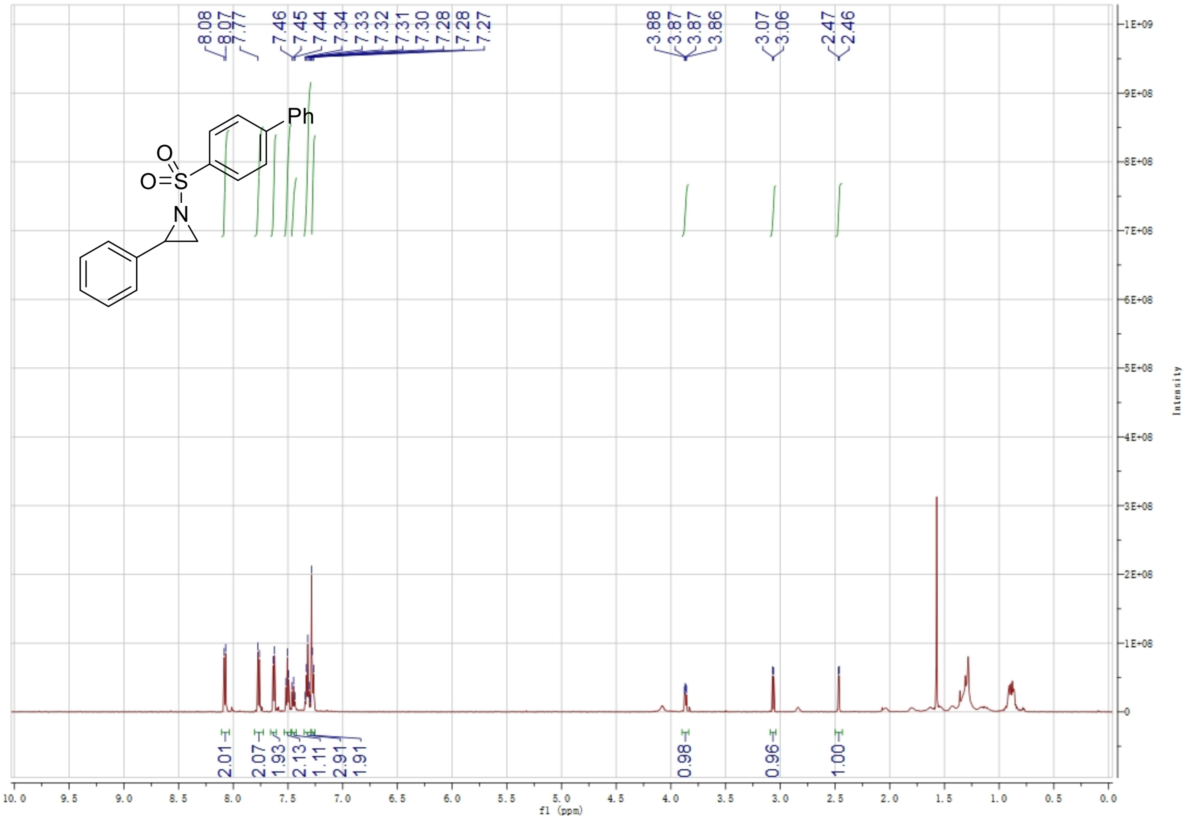

^{13}C NMR (151 MHz, 293 K, $CDCl_3$)

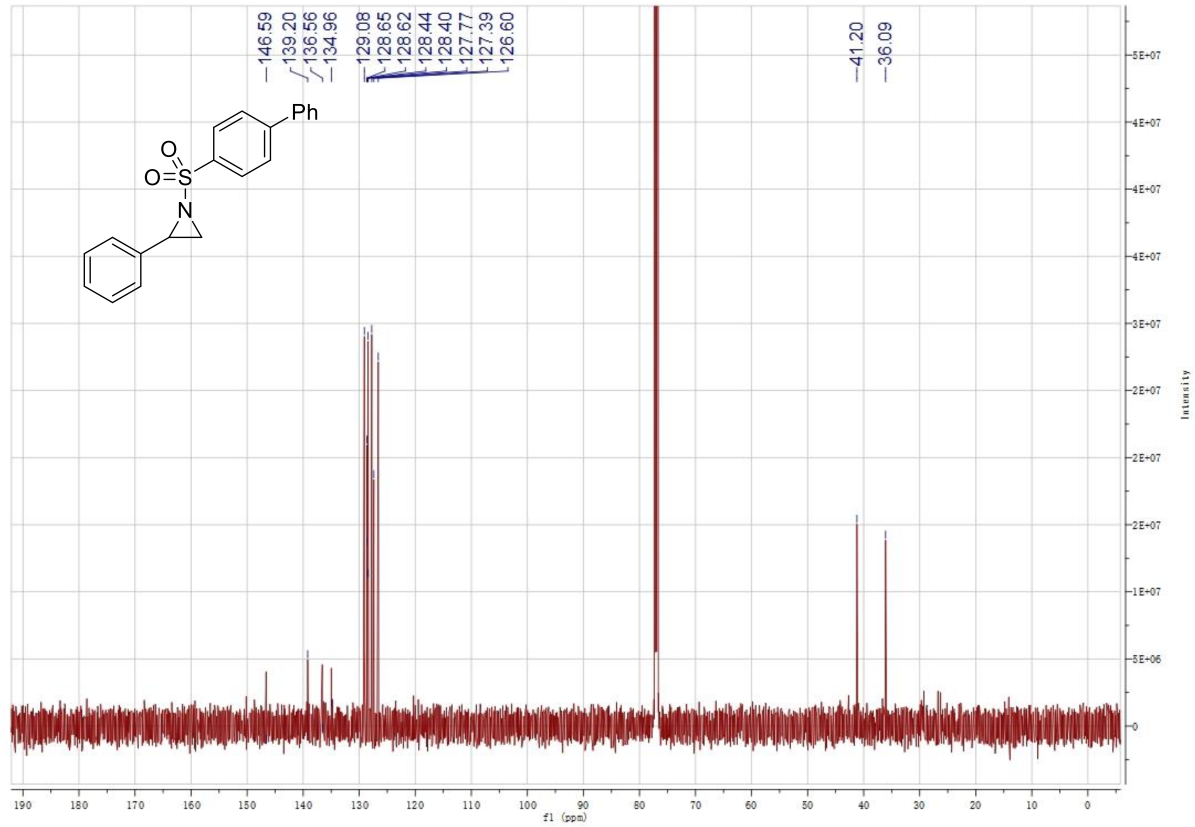

Supplementary Figure 86. *1-(Naphthalen-2-ylsulfonyl)-2-phenylaziridine* (***22h***)

^{1}H NMR (600 MHz, 293 K, $CDCl_3$)

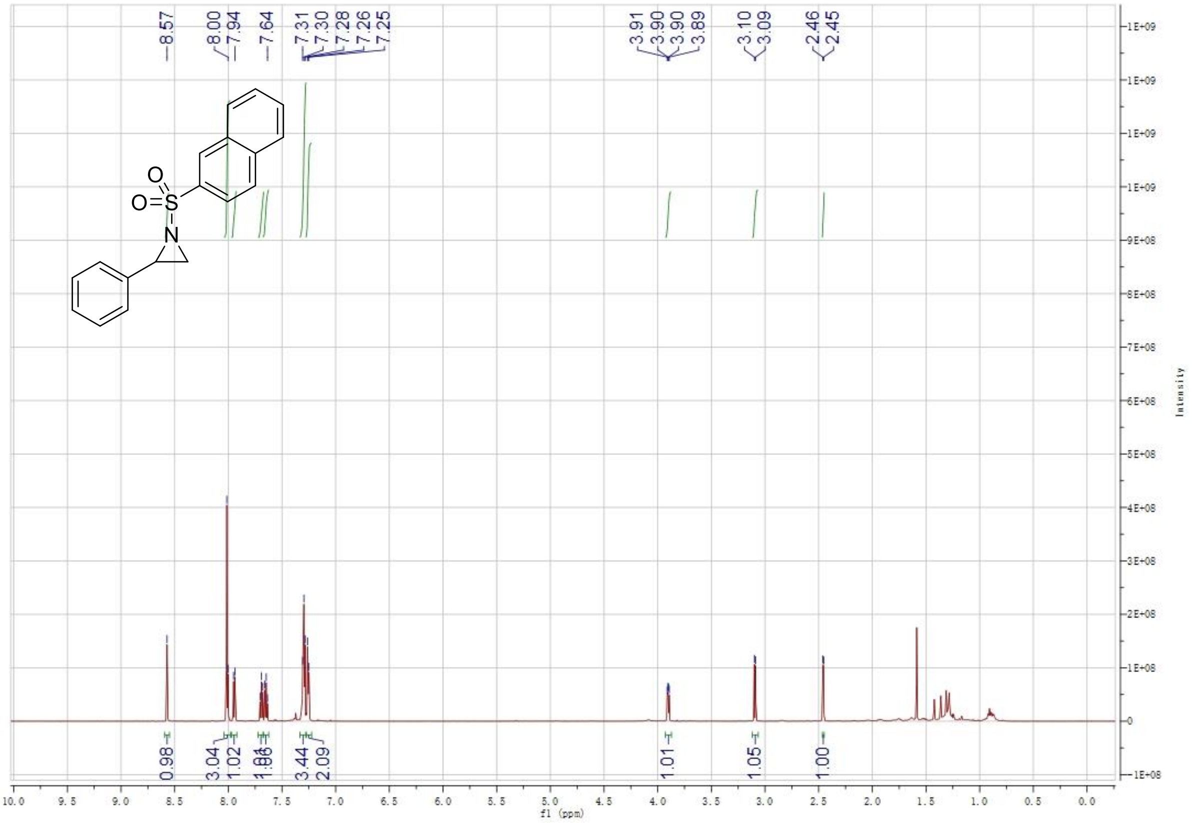

^{13}C NMR (151 MHz, 293 K, $CDCl_3$)

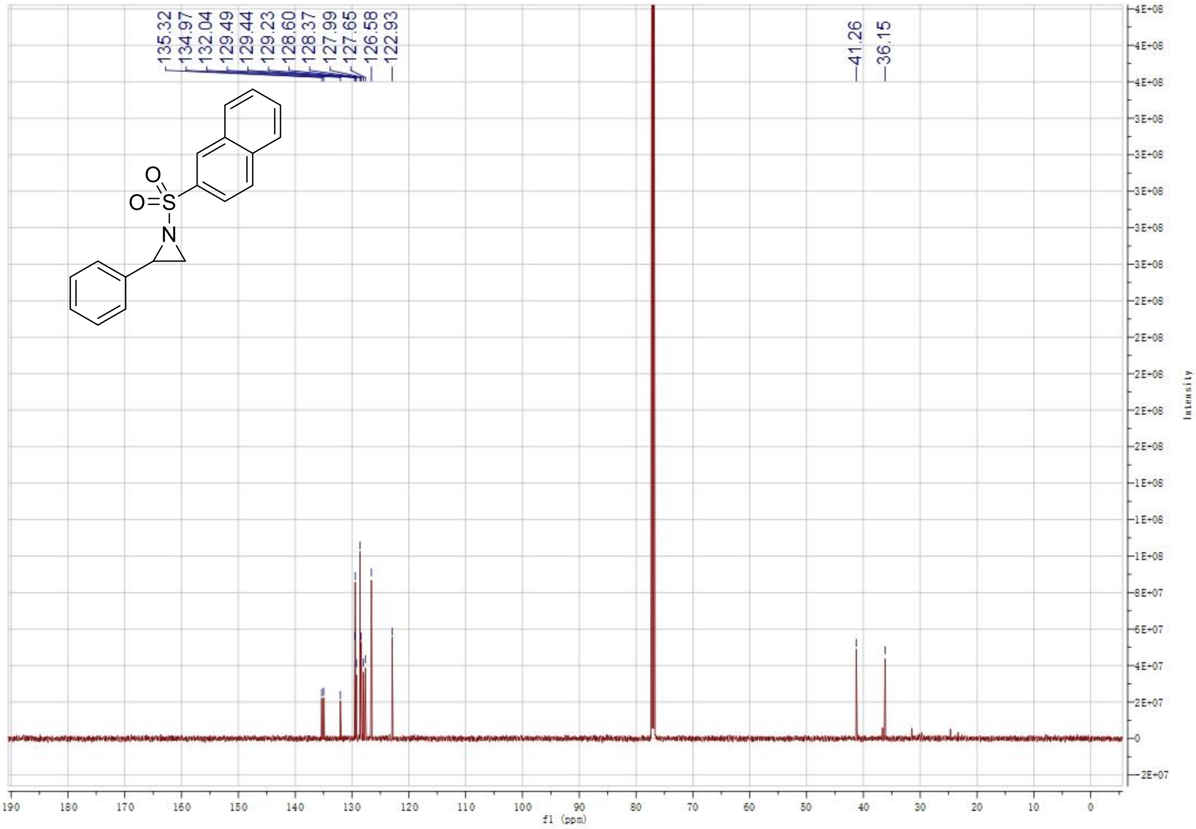

Supplementary Figure 87. *2-(p-Tolyl)-1-tosylaziridine (**22i**)*

^{1}H NMR (400 MHz, 293 K, $CDCl_3$)

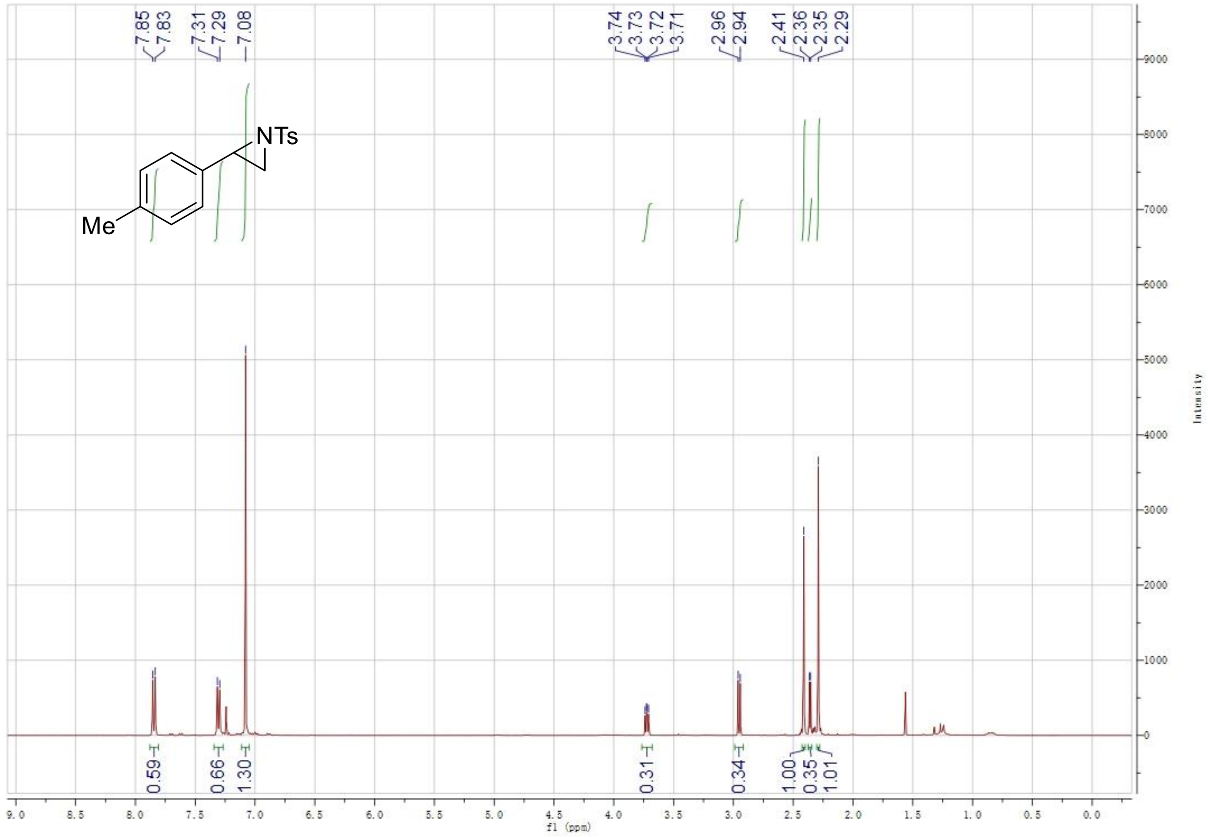

^{13}C NMR (101 MHz, 293 K, $CDCl_3$)

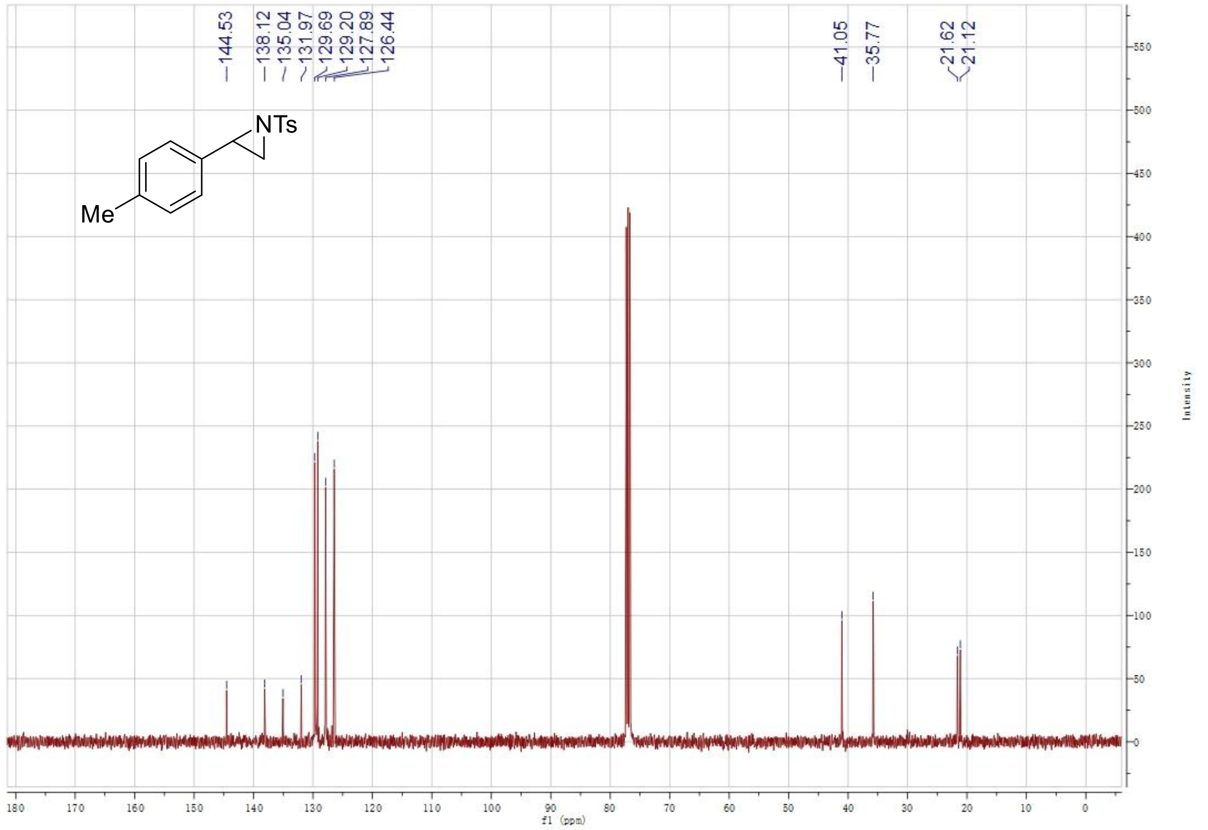

Supplementary Figure 88. *1-(4-(tert-Butyl)phenyl)-1-tosylaziridine (**22j**)*

^{1}H NMR (400 MHz, 293 K, $CDCl_3$)

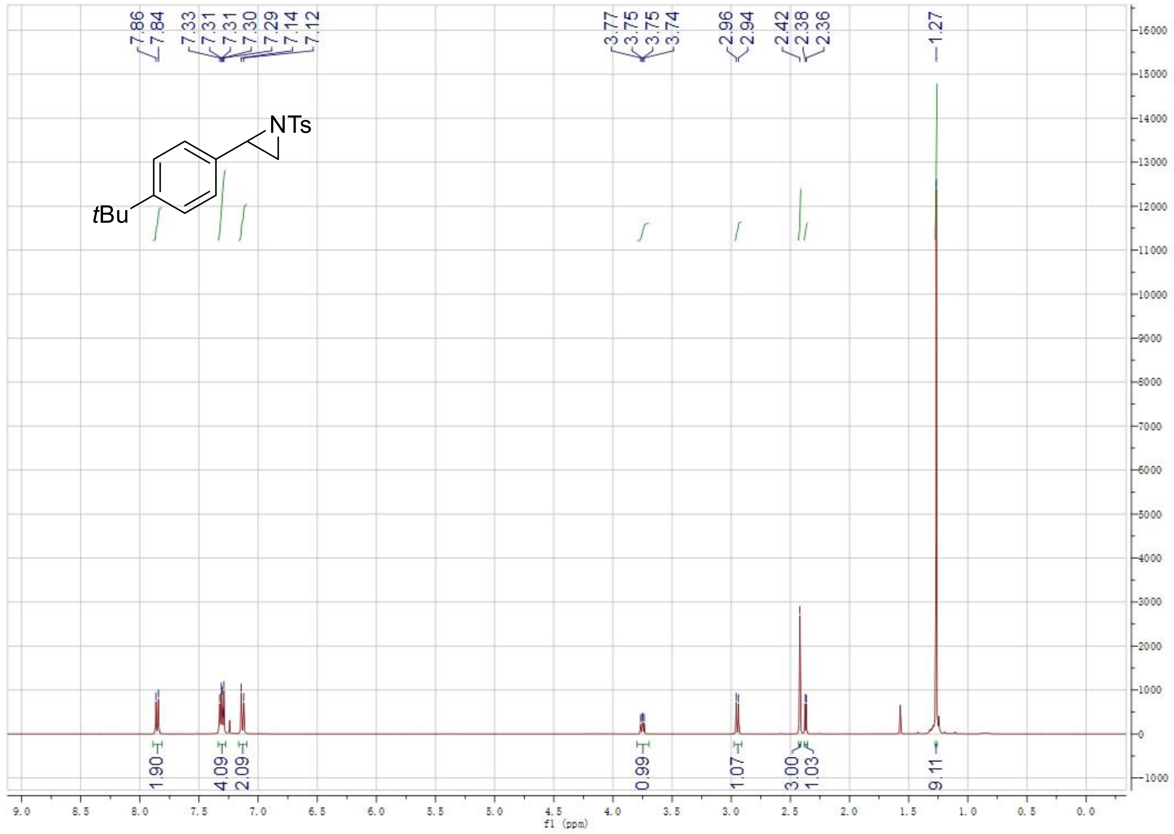

^{13}C NMR (101 MHz, 293 K, $CDCl_3$)

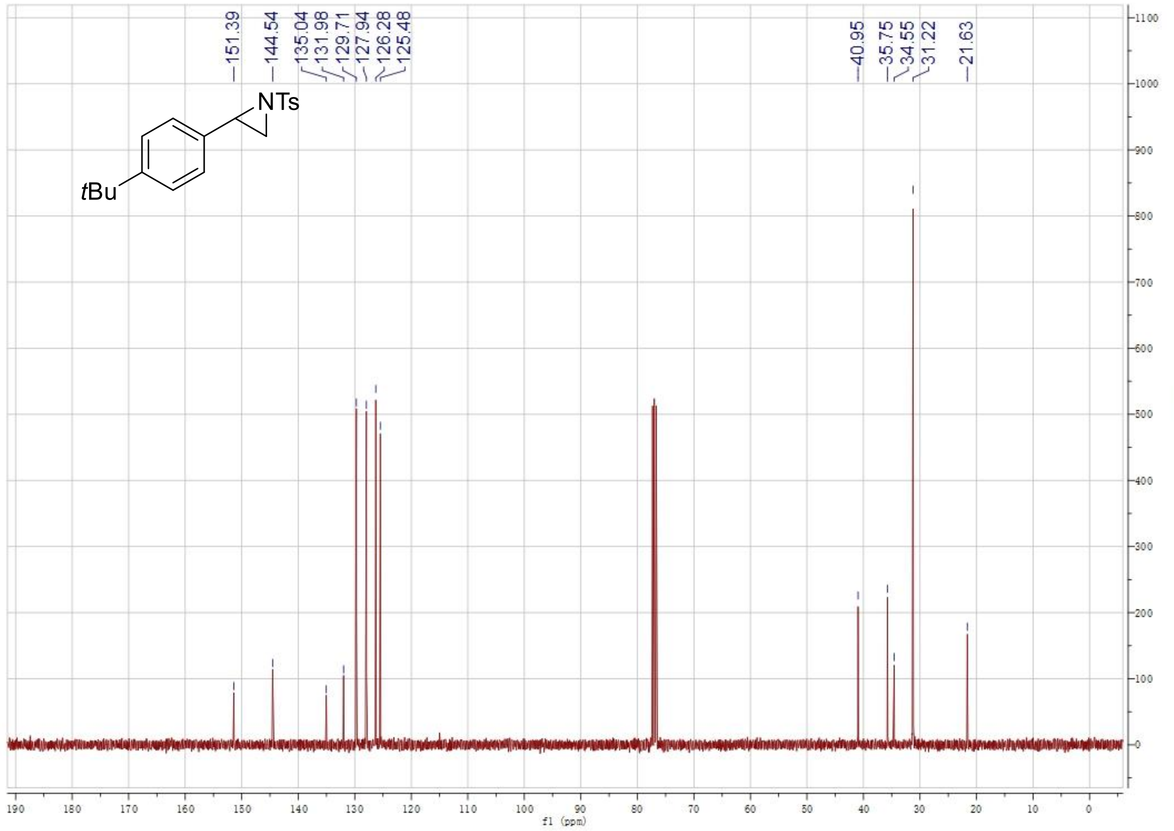

Supplementary Figure 89. *1-Tosyl-2-(4-(trifluoromethyl)phenyl)aziridine* (***22k***)

^{1}H NMR (600 MHz, 293 K, $CDCl_3$)

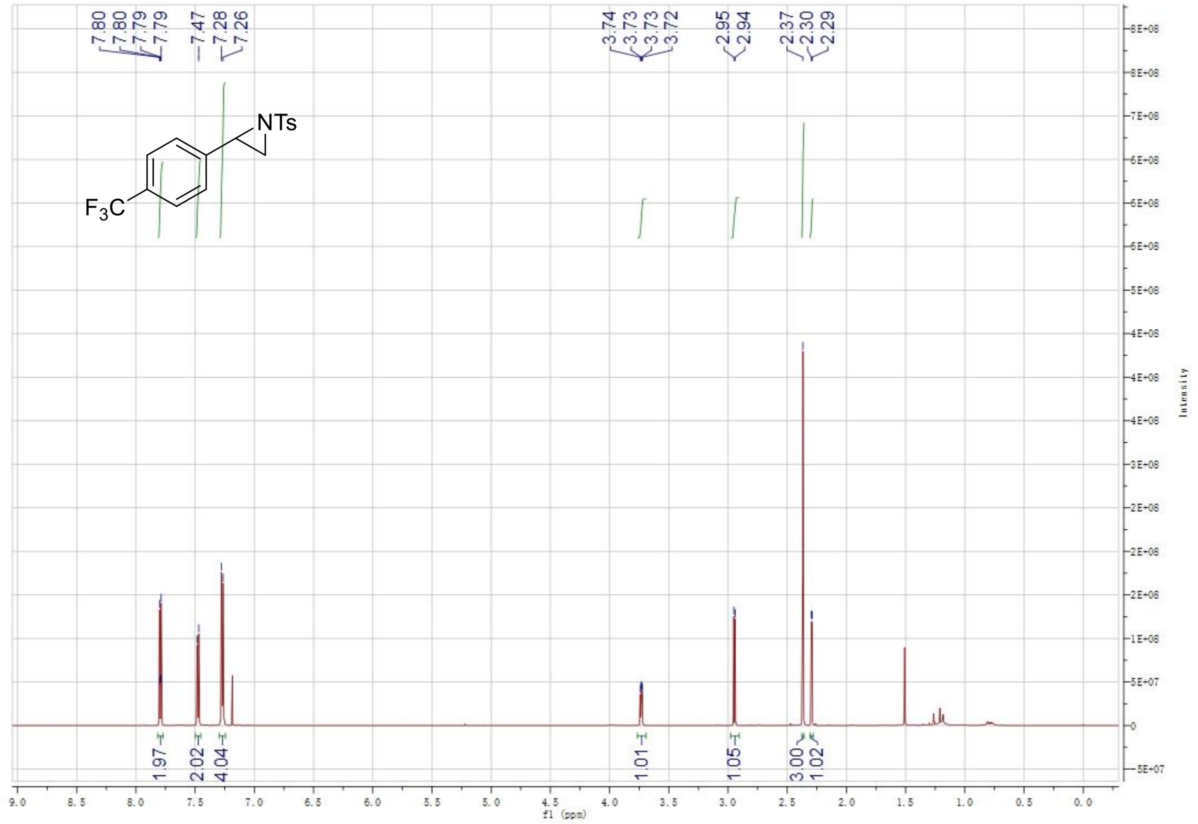

^{13}C NMR (151 MHz, 293 K, $CDCl_3$)

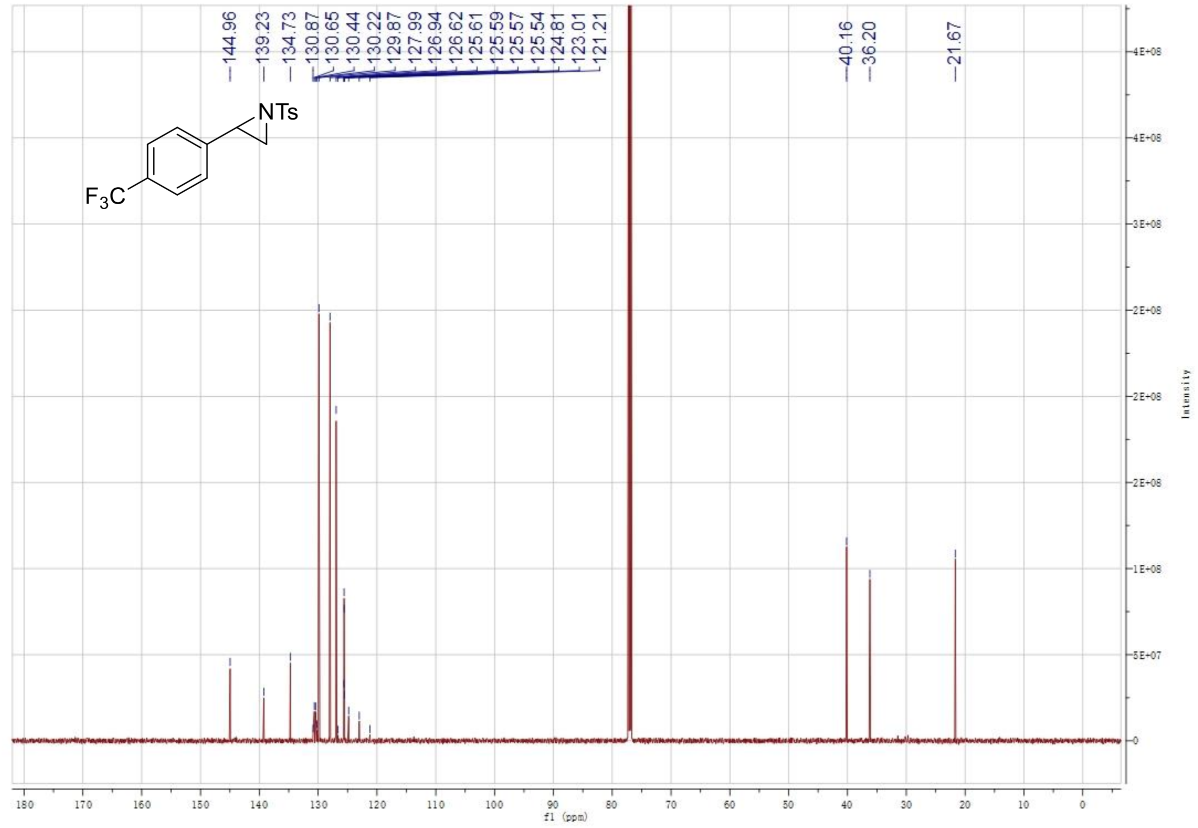

^{19}F NMR (565 MHz, 293 K, $CDCl_3$)

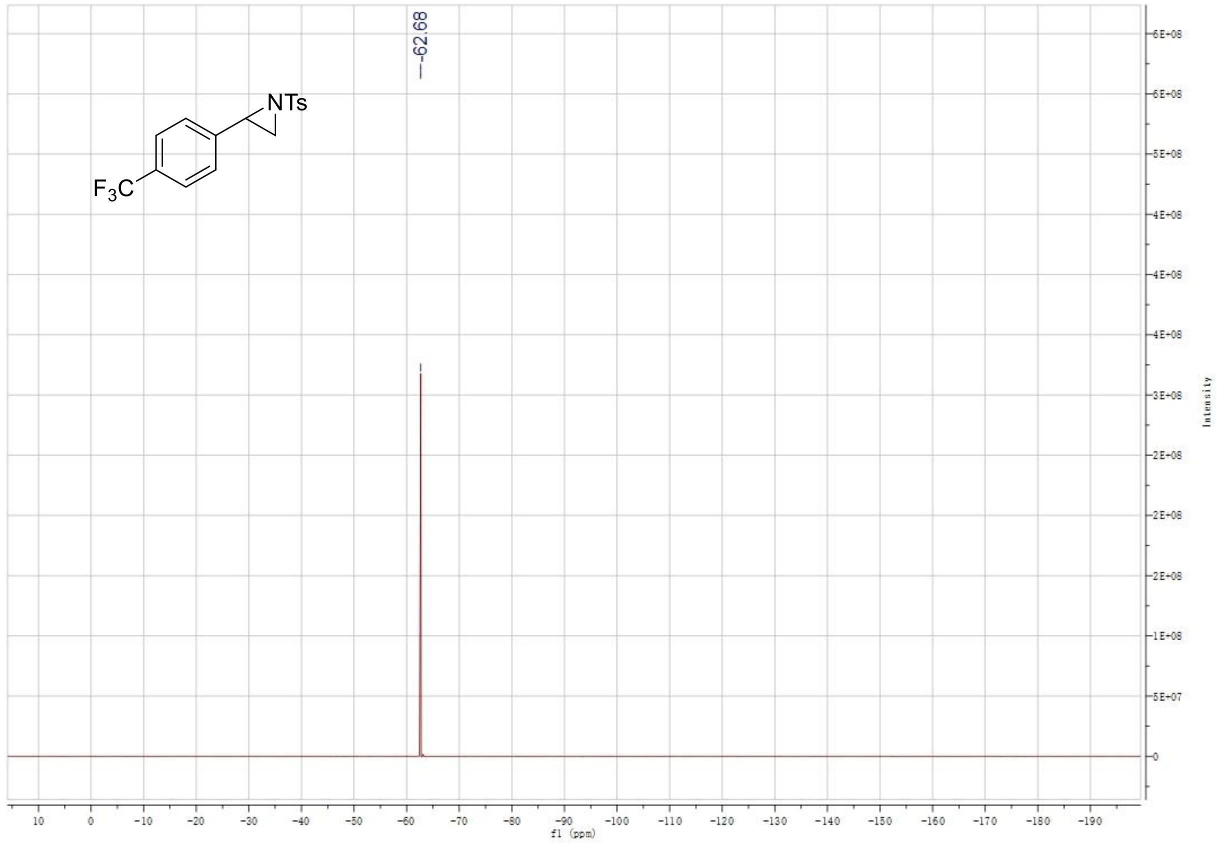

Supplementary Figure 90. *2-(4-Fluorophenyl)-1-tosylaziridine (**22l**)*

^{1}H NMR (400 MHz, 293 K, $CDCl_3$)

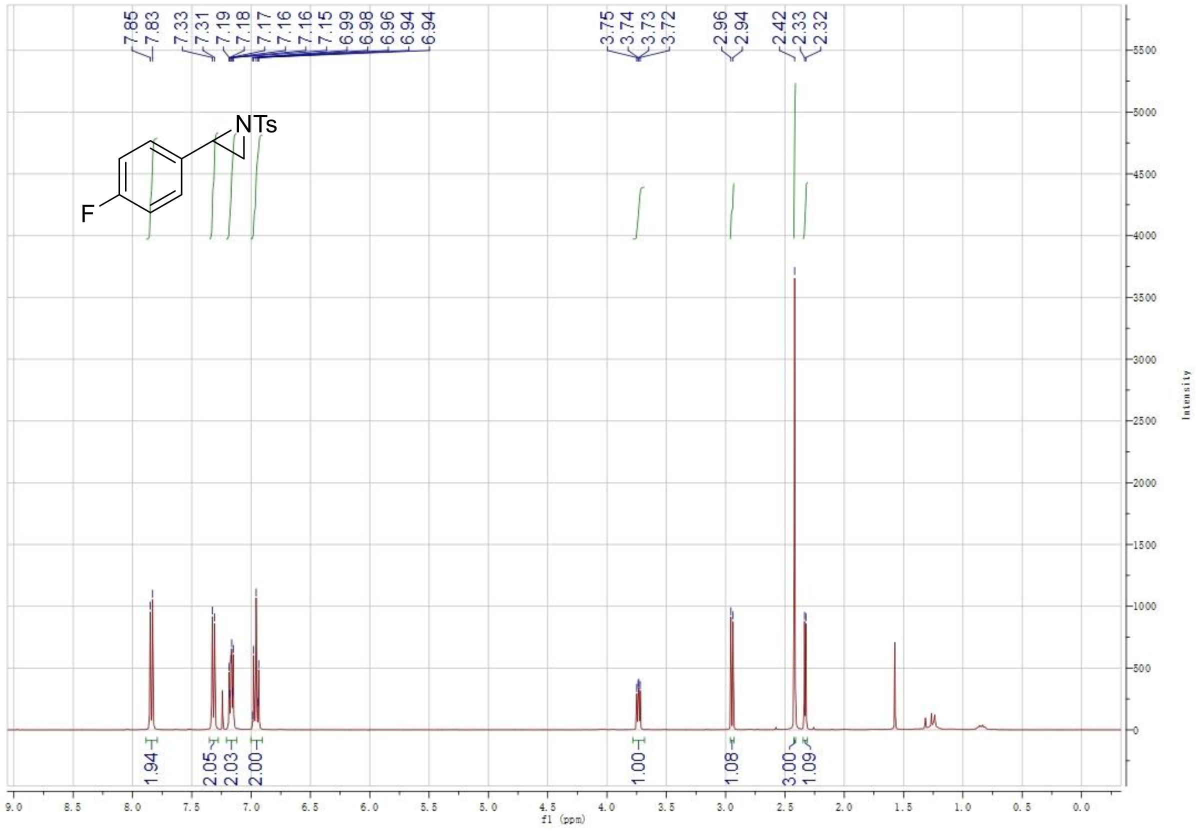

^{13}C NMR (101 MHz, 293 K, CDCl$_3$)

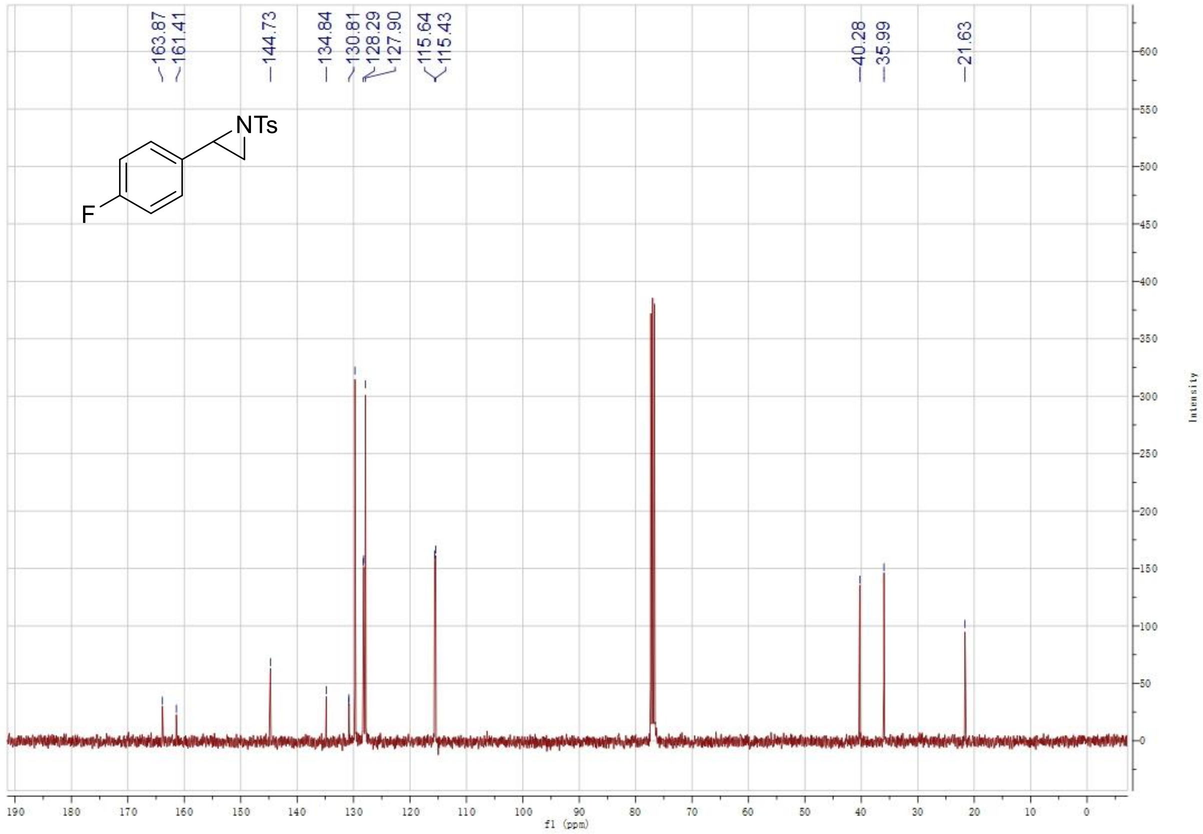

^{19}F NMR (376 MHz, 293 K, CDCl$_3$)

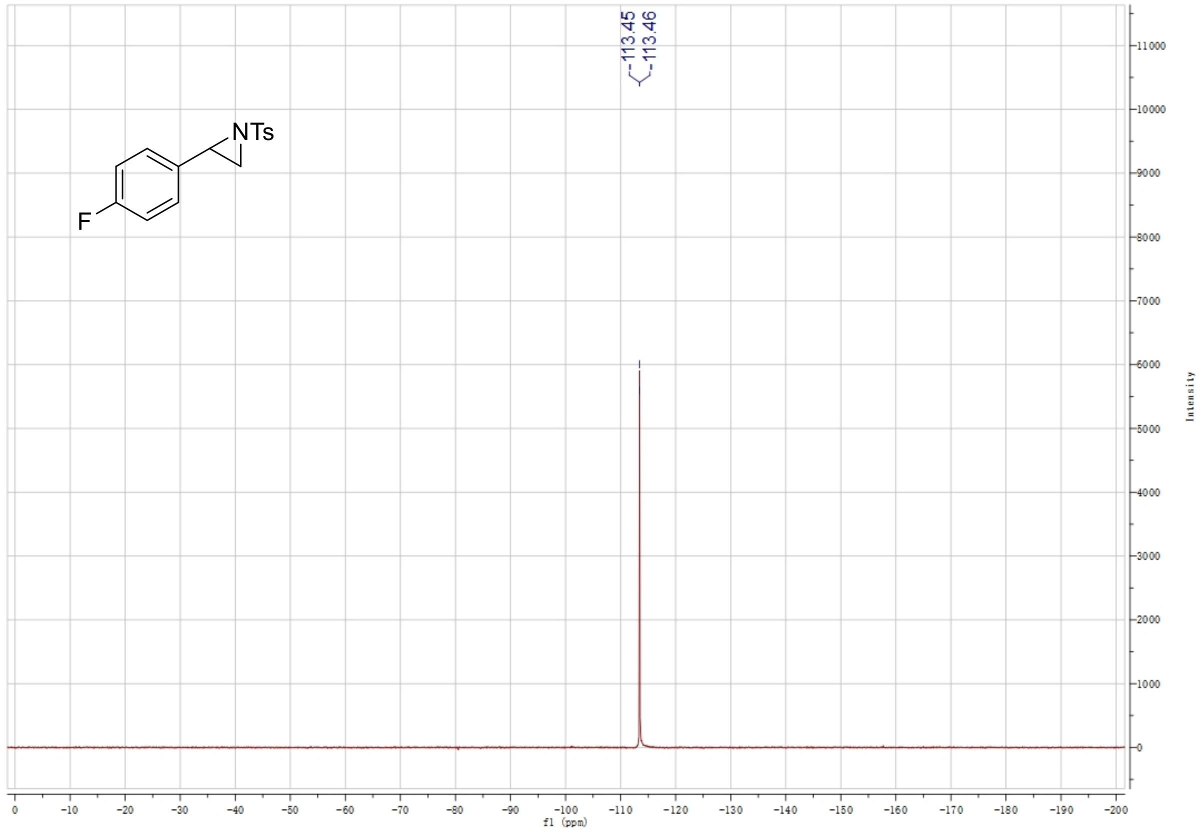

Supplementary Figure 91. *2-(4-Chlorophenyl)-1-tosylaziridine (**22m**)*

^{1}H NMR (400 MHz, 293 K, $CDCl_3$)

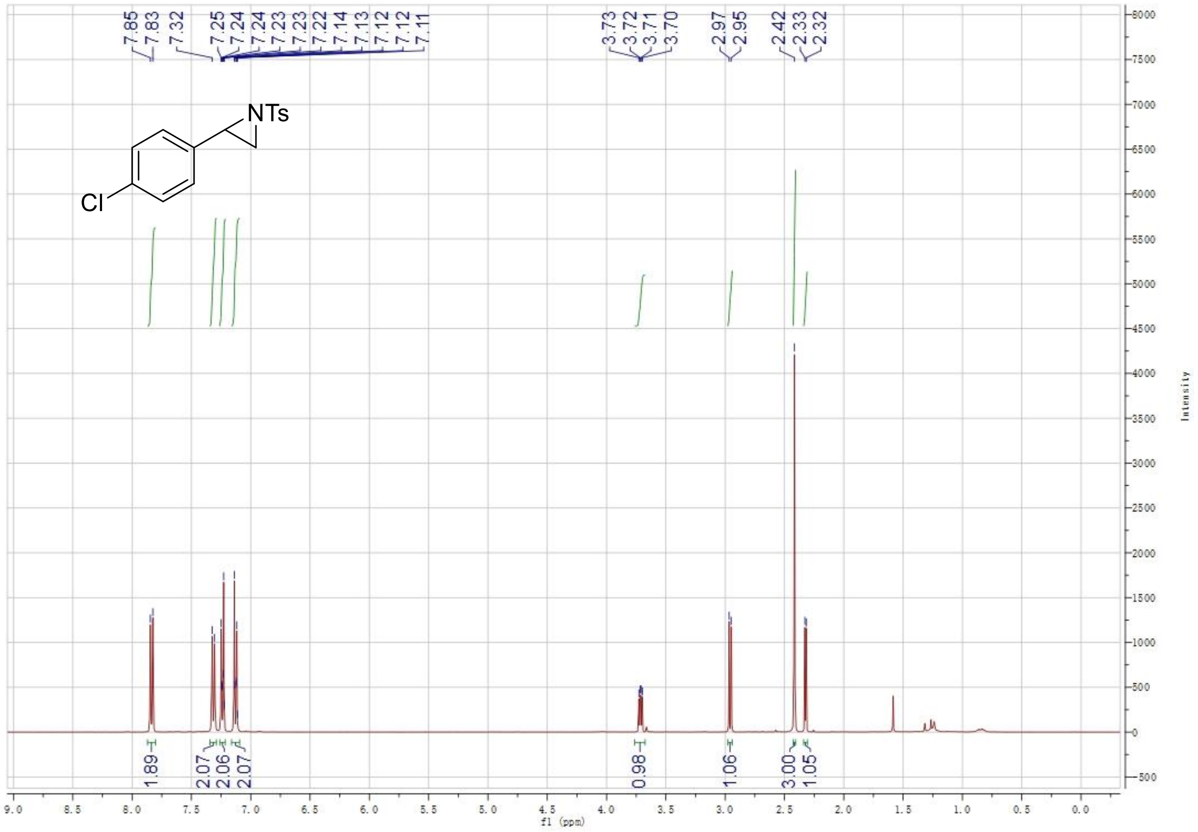

^{13}C NMR (101 MHz, 293 K, $CDCl_3$)

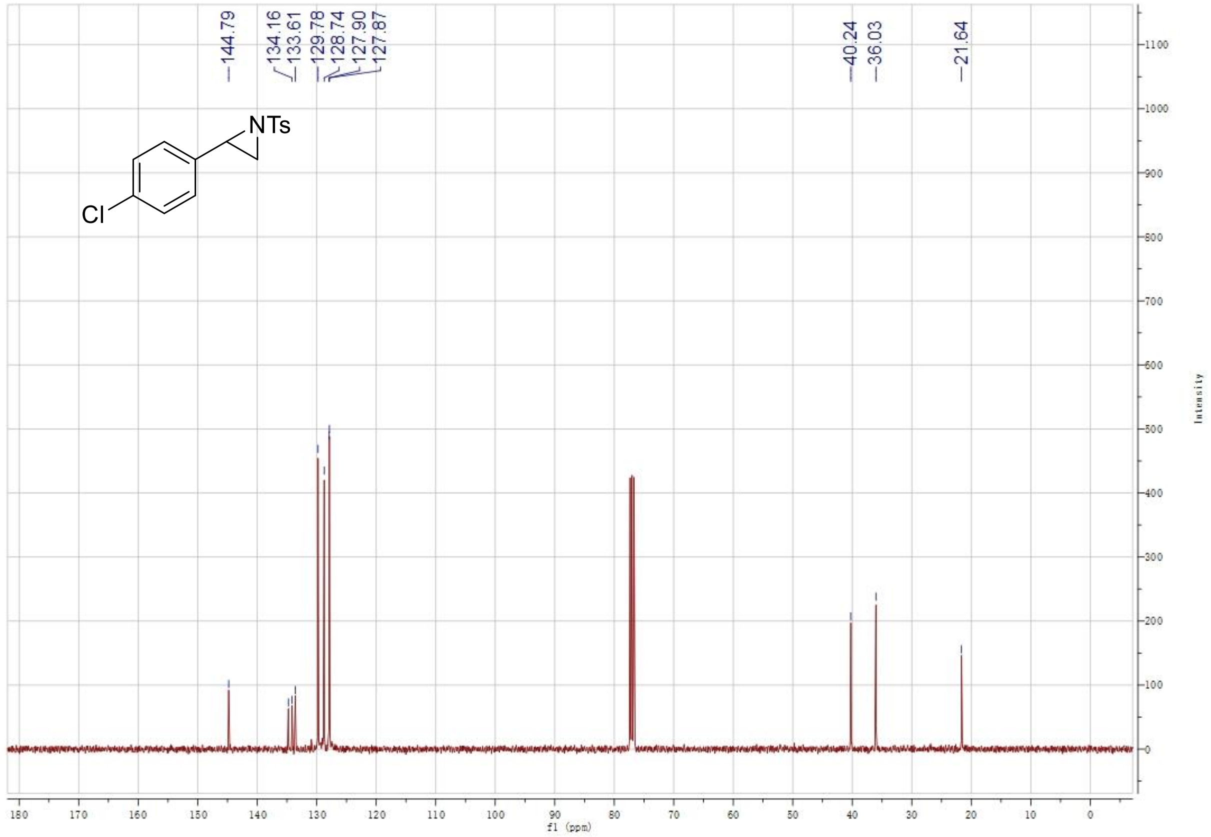

Supplementary Figure 92. *1-(4-Bromophenyl)-1-tosylaziridine (**22n**)*

^{1}H NMR (400 MHz, 293 K, $CDCl_3$)

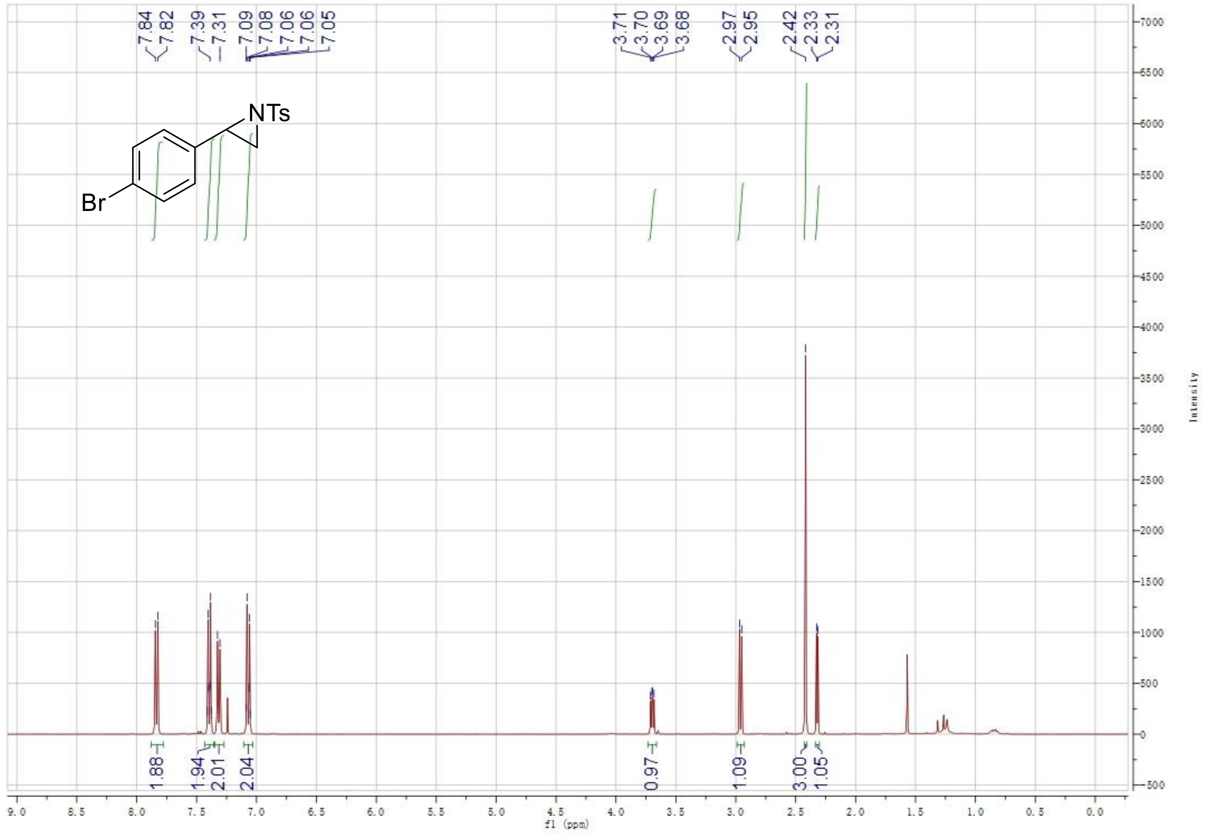

^{13}C NMR (101 MHz, 293 K, $CDCl_3$)

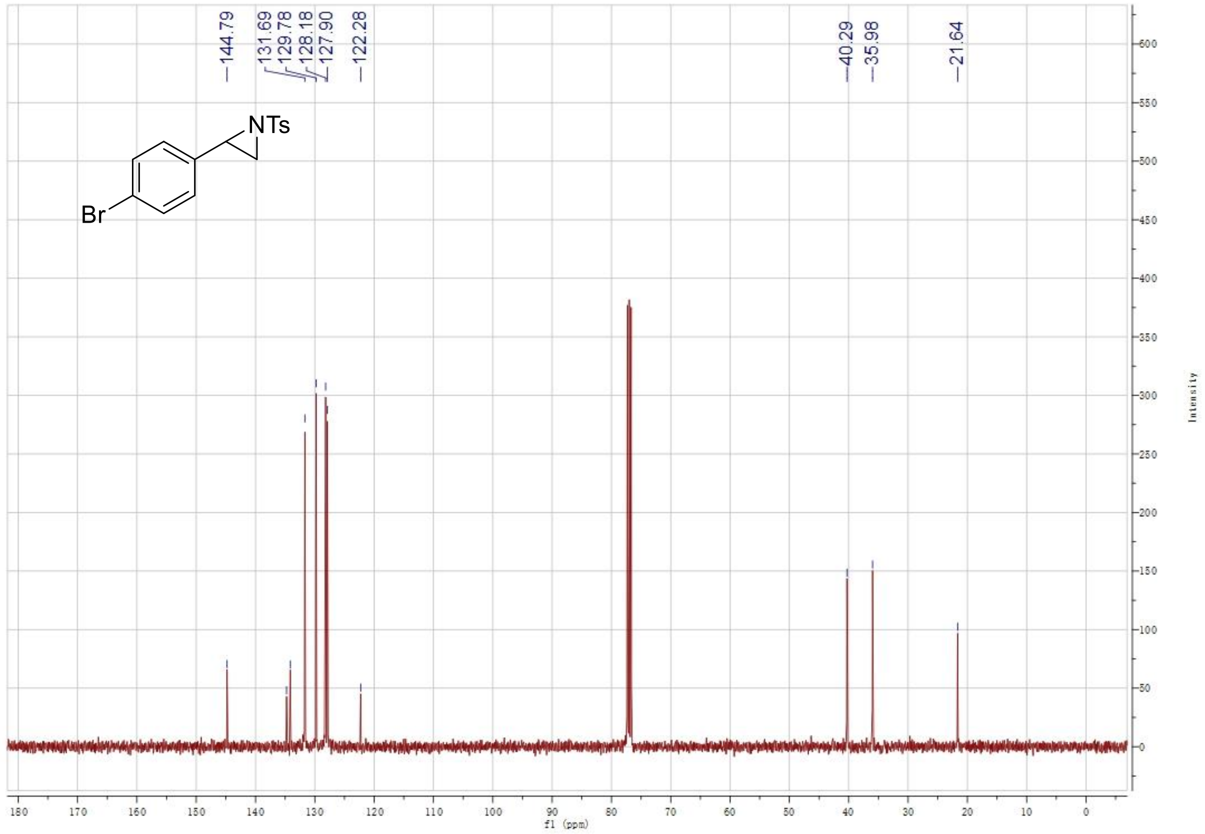

Supplementary Figure 93. *1-(1-Tosylaziridin-2-yl)phenyl acetate (**22o**)*

^{1}H NMR (600 MHz, 293 K, $CDCl_3$)

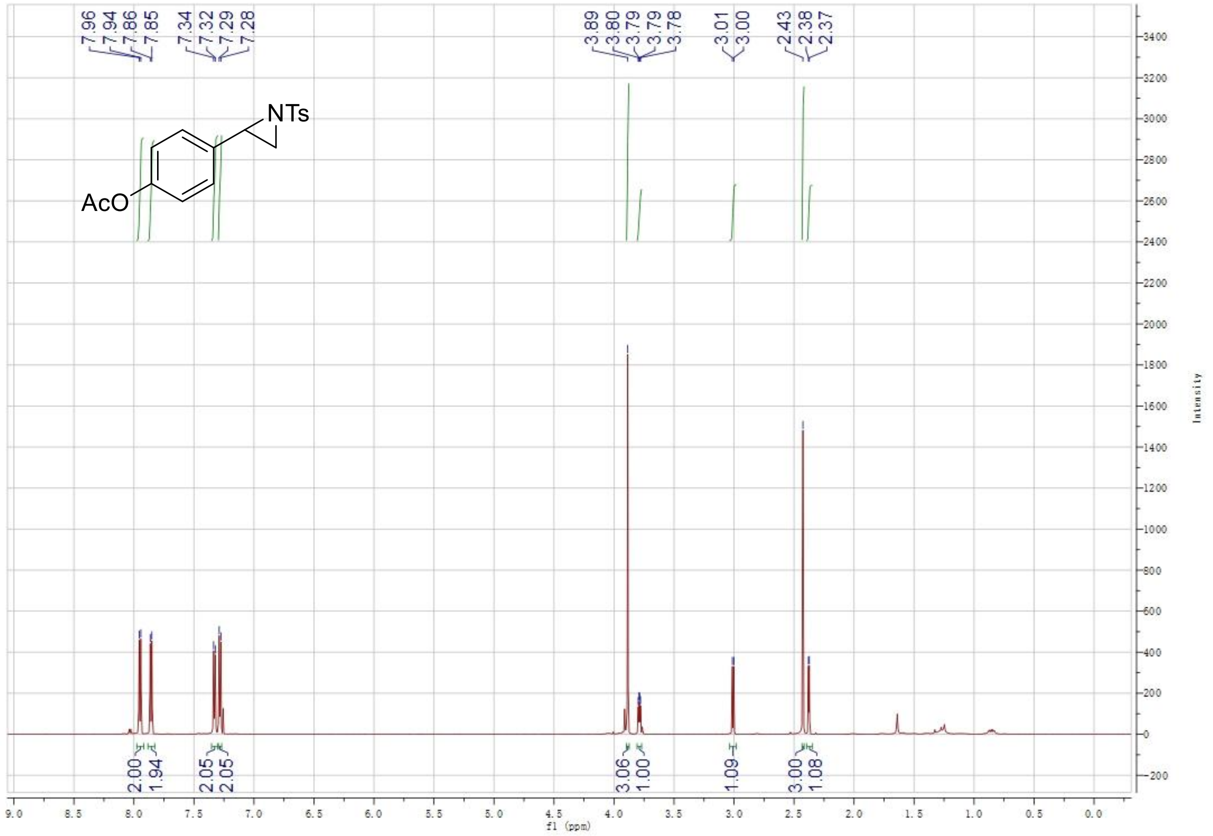

^{13}C NMR (151 MHz, 293 K, $CDCl_3$)

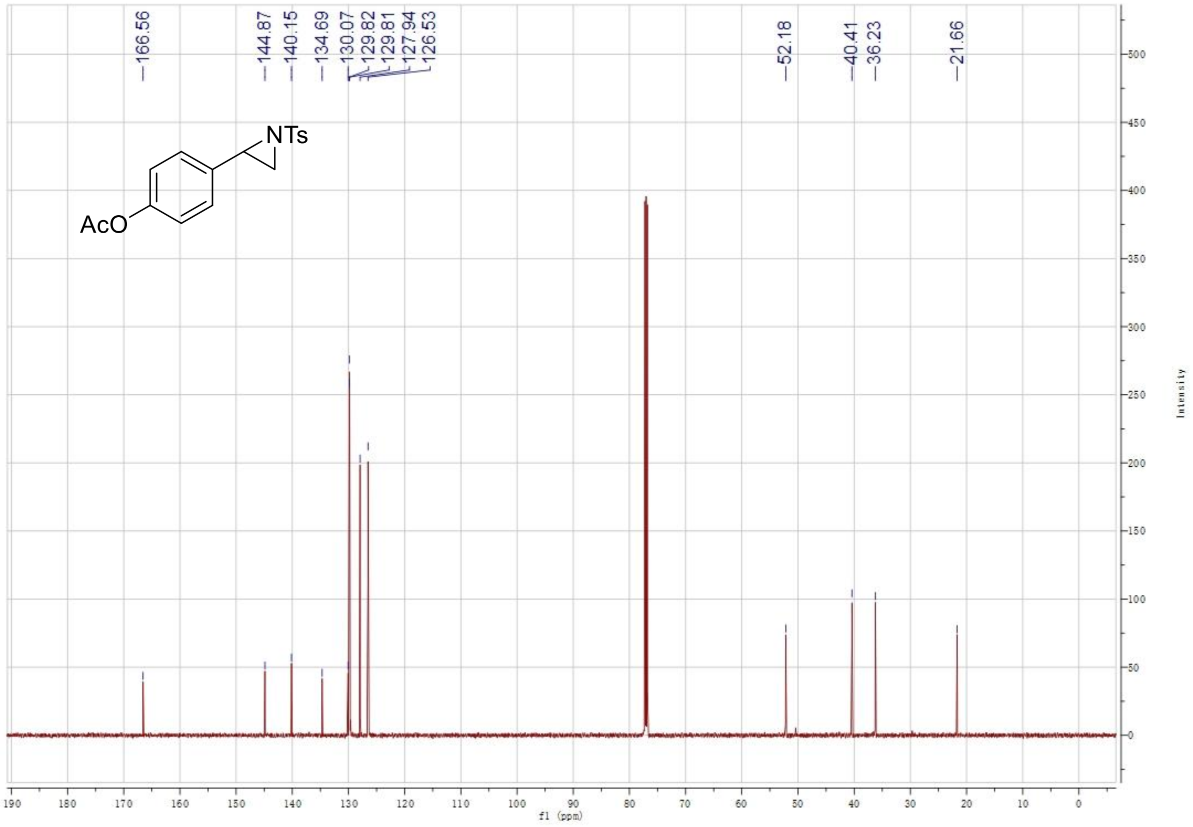

Supplementary Figure 94. *1-(m-Tolyl)-1-tosylaziridine (**22p**)*

^{1}H NMR (400 MHz, 293 K, $CDCl_3$)

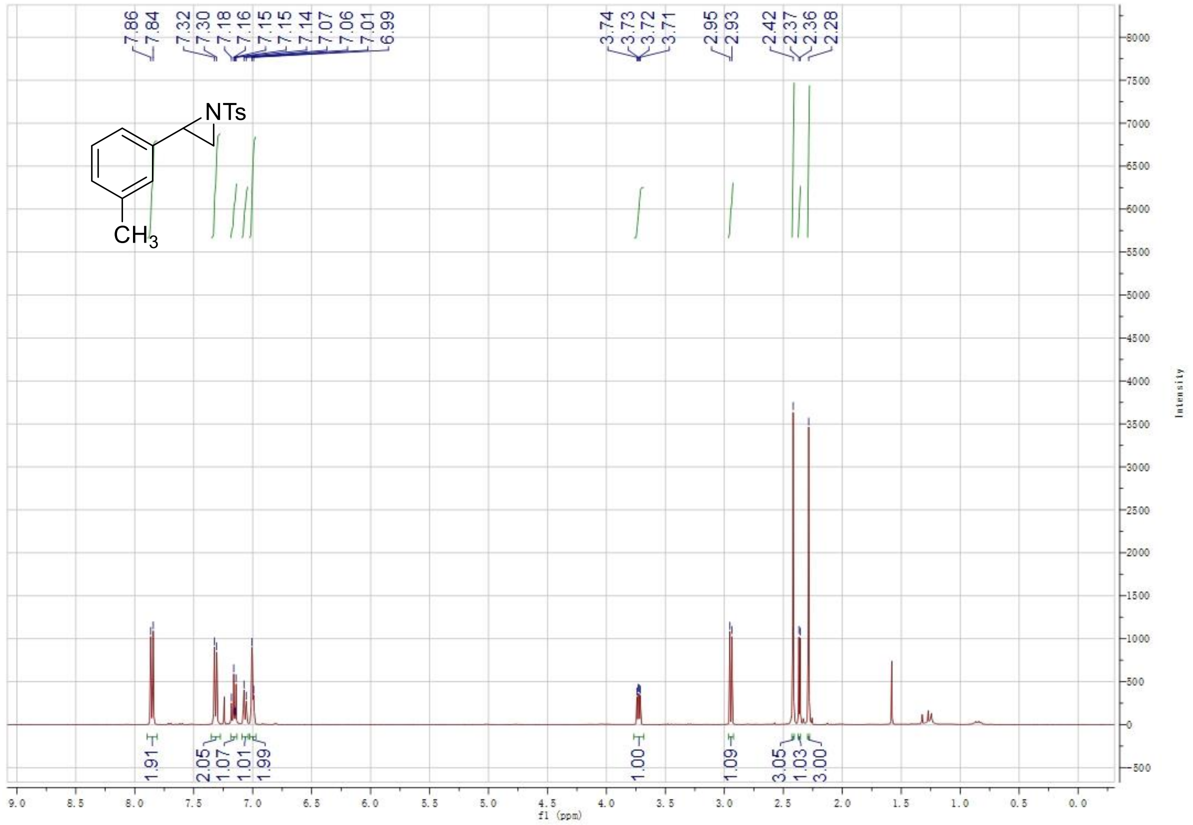

^{13}C NMR (101 MHz, 293 K, $CDCl_3$)

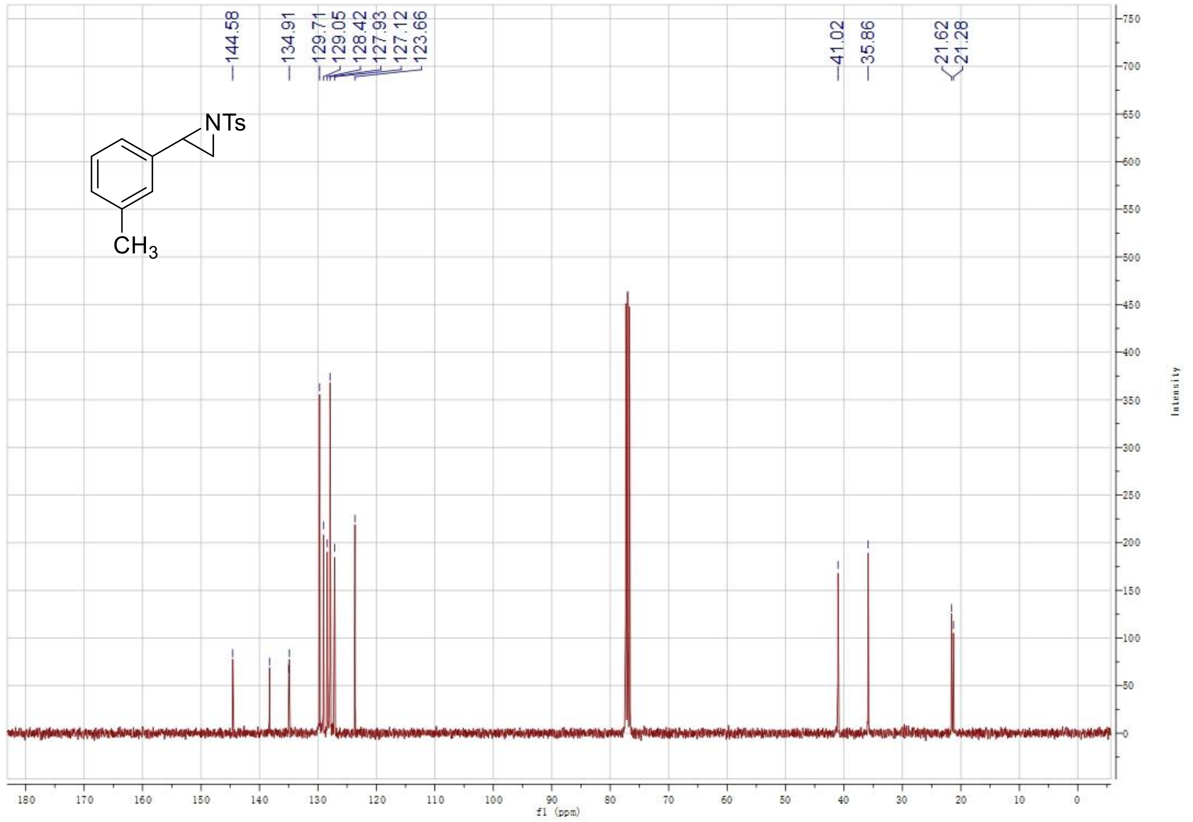

Supplementary Figure 95. *1-Tosyl-2-(3-(trifluoromethyl)phenyl)aziridine* ***(22q)***

^{1}H NMR (600 MHz, 293 K, $CDCl_3$)

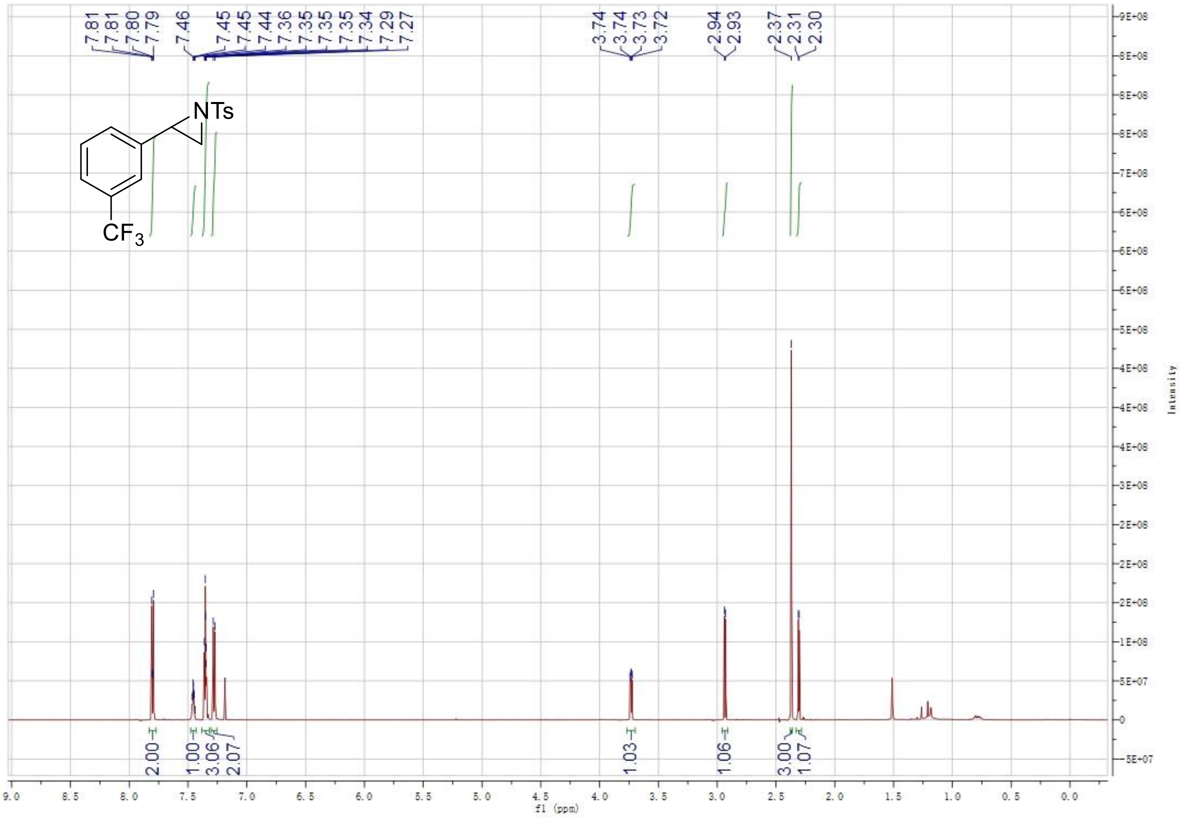

^{13}C NMR (151 MHz, 293 K, $CDCl_3$)

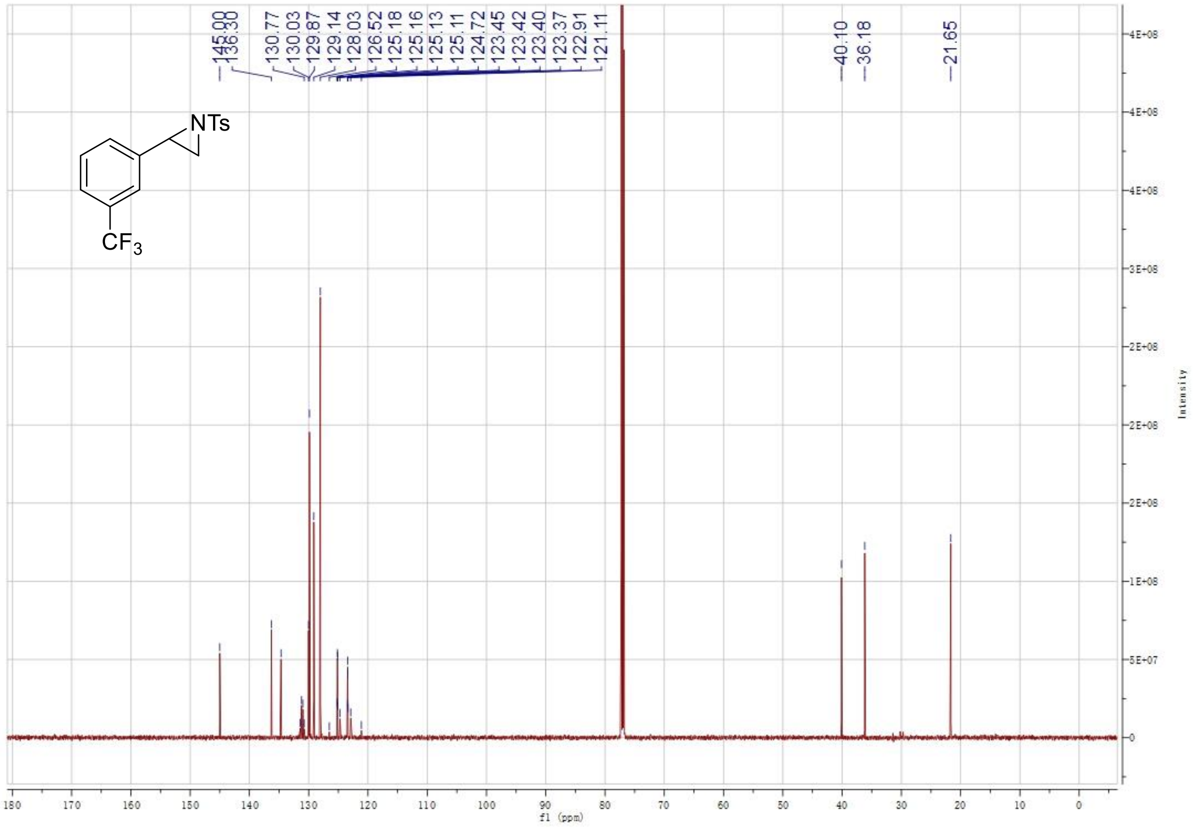

^{19}F NMR (565 MHz, 293 K, $CDCl_3$)

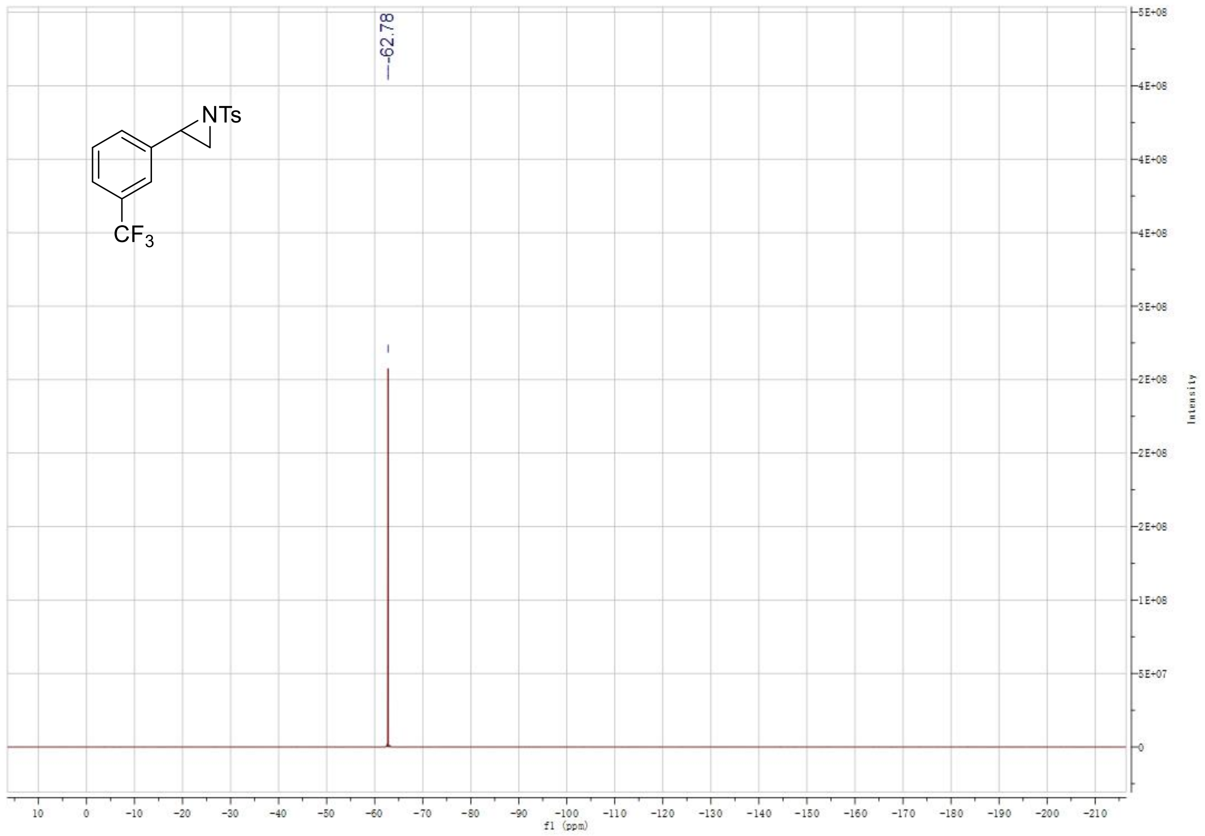

Supplementary Figure 96. *1-(3-Nitrophenyl)-1-tosylaziridine (**22r**)*
^{1}H NMR (600 MHz, 293 K, $CDCl_3$)

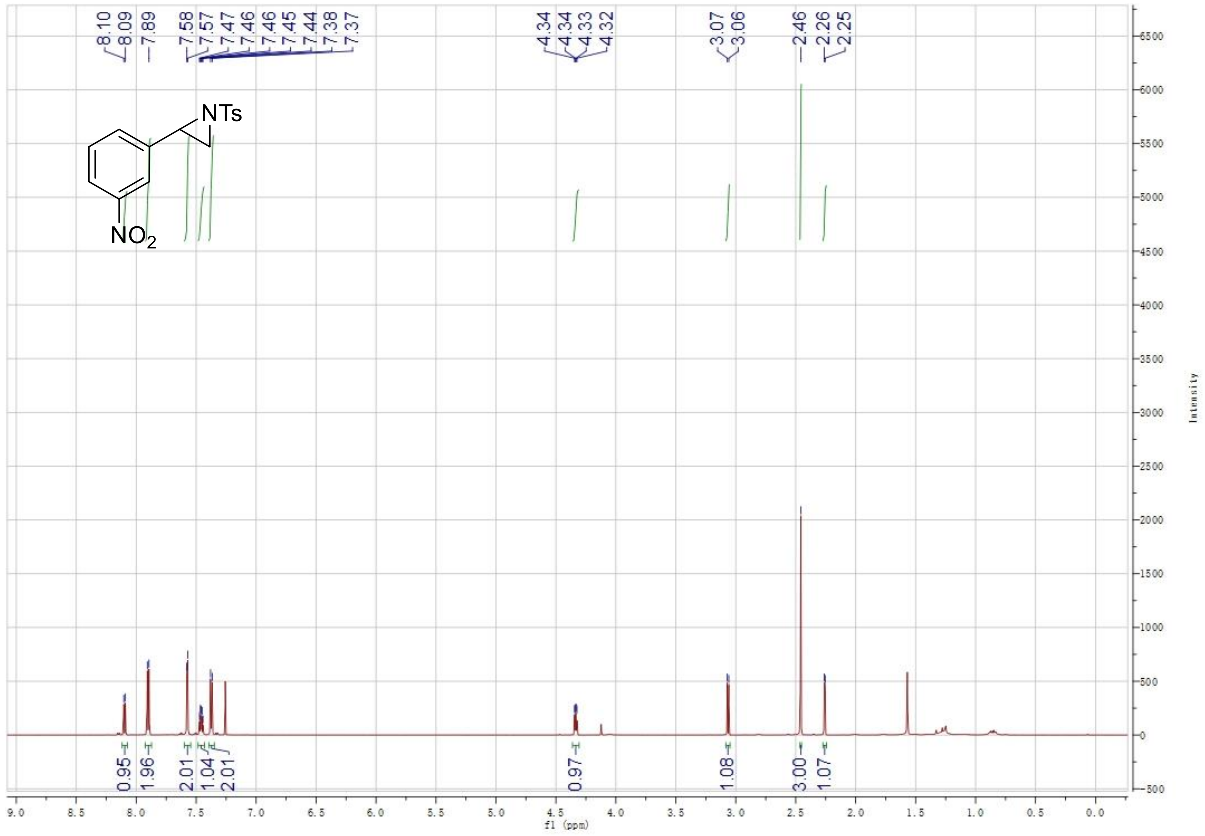

^{13}C NMR (151 MHz, 293 K, CDCl$_3$)

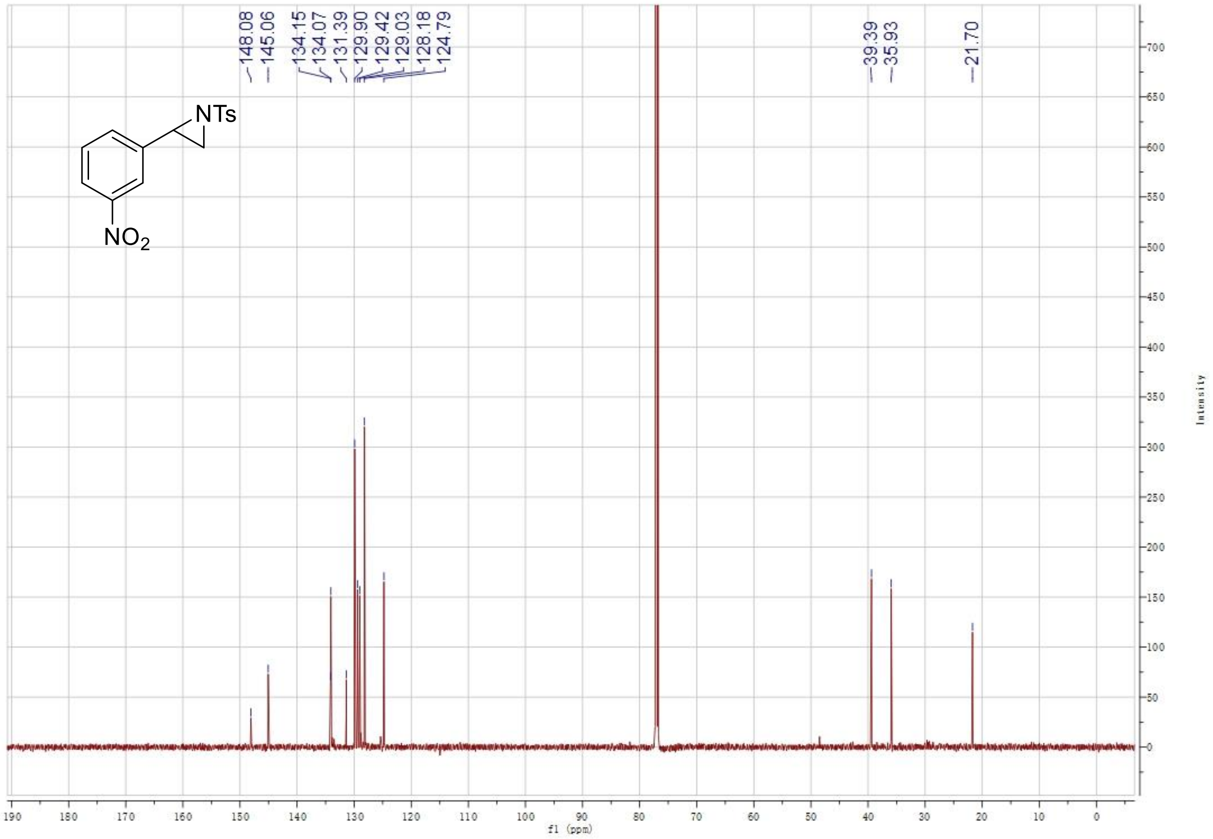

Supplementary Figure 97. *1-(2-Fluorophenyl)-1-tosylaziridine (**22s**)*

^{1}H NMR (400 MHz, 293 K, CDCl$_3$)

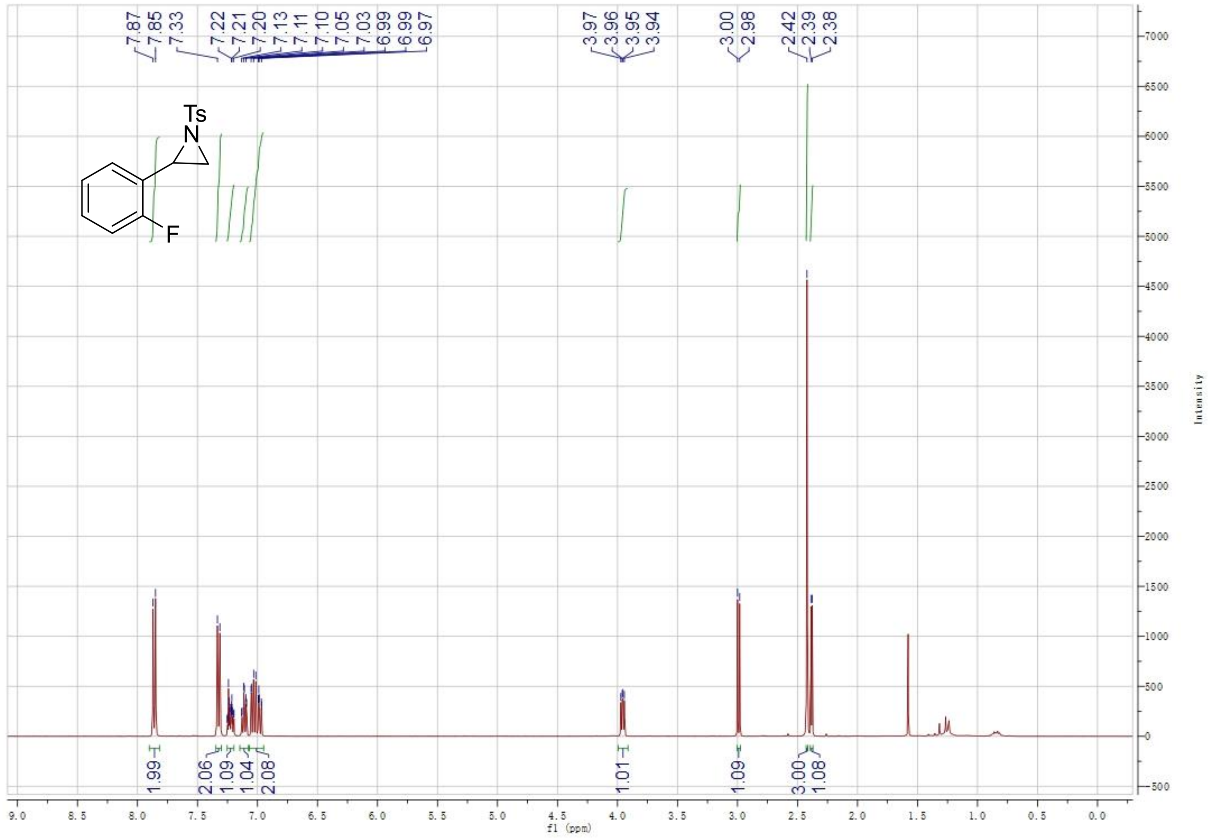

^{13}C NMR (101 MHz, 293 K, $CDCl_3$)

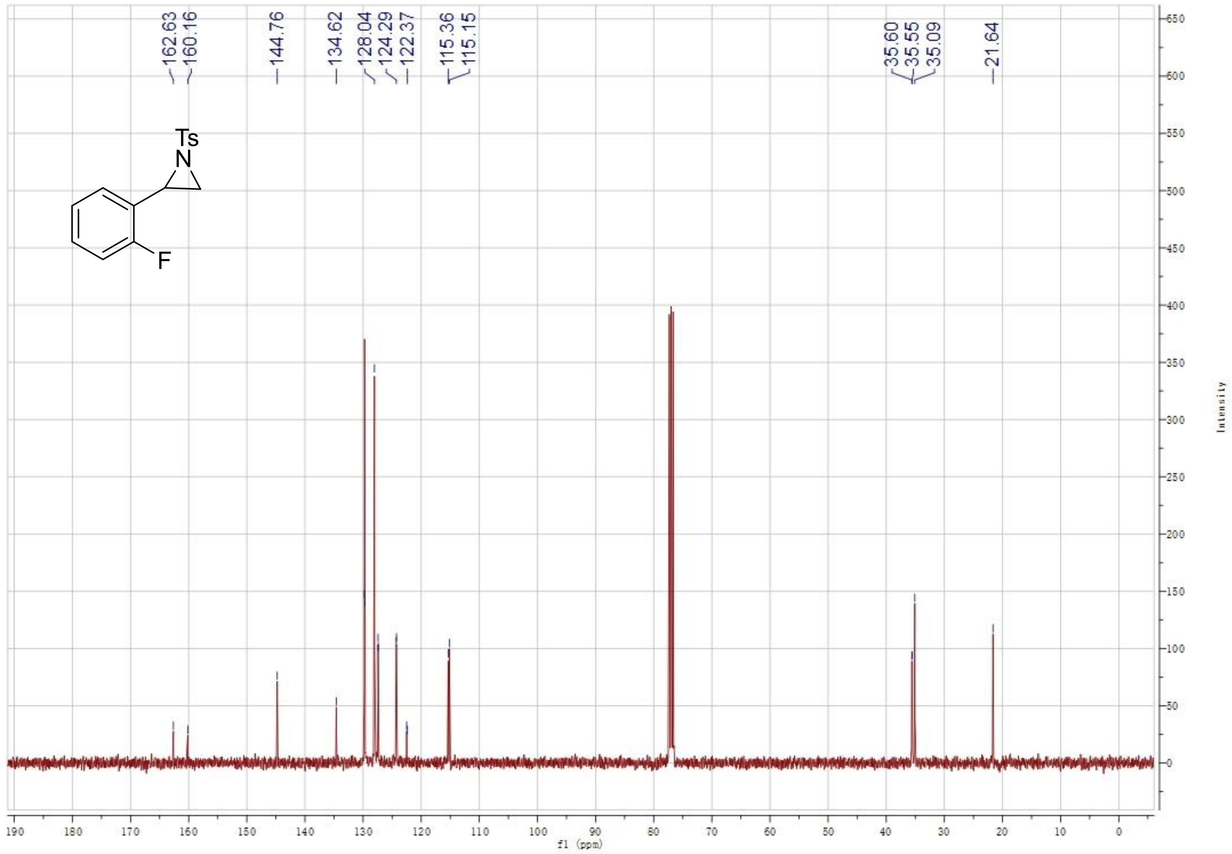

^{19}F NMR (376 MHz, 293 K, $CDCl_3$)

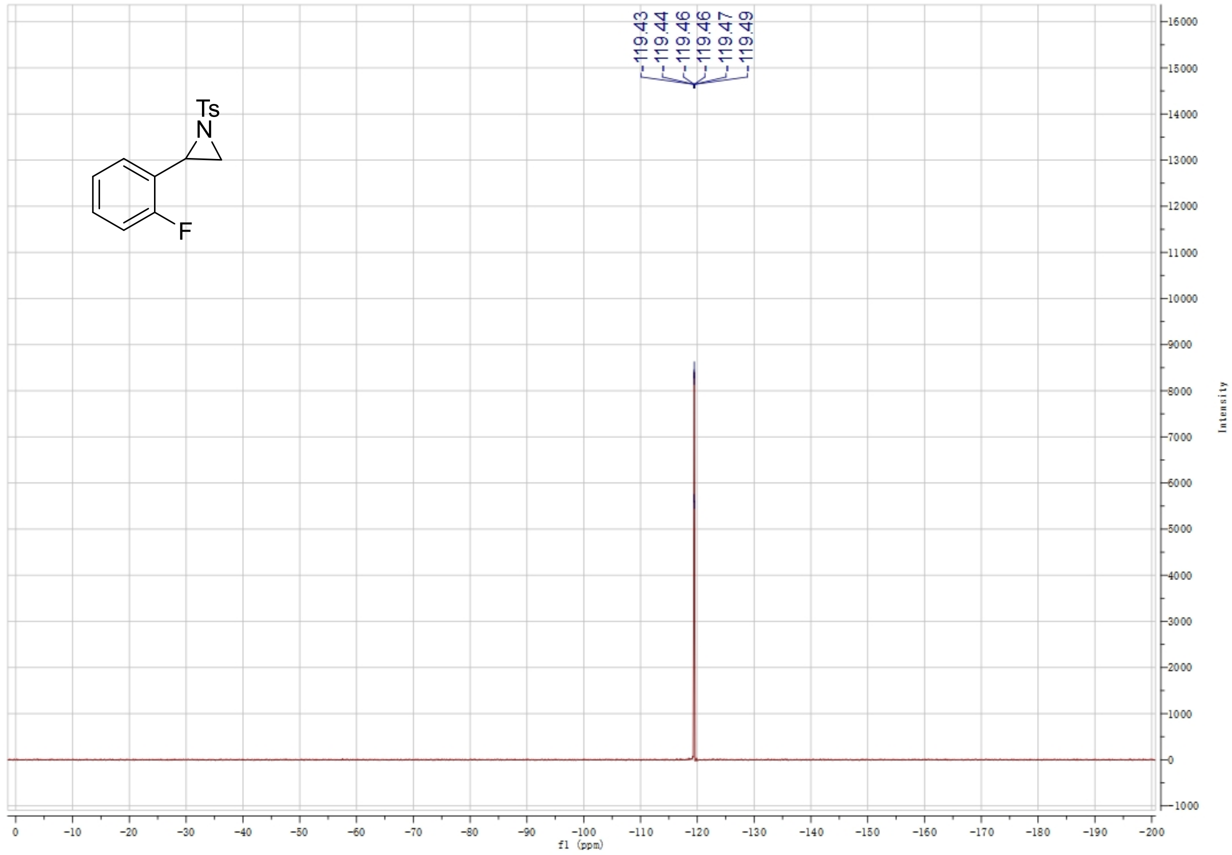

Supplementary Figure 98. *1-(2,6-Dichlorophenyl)-1-tosylaziridine (**22t**)*

^{1}H NMR (600 MHz, 293 K, $CDCl_3$)

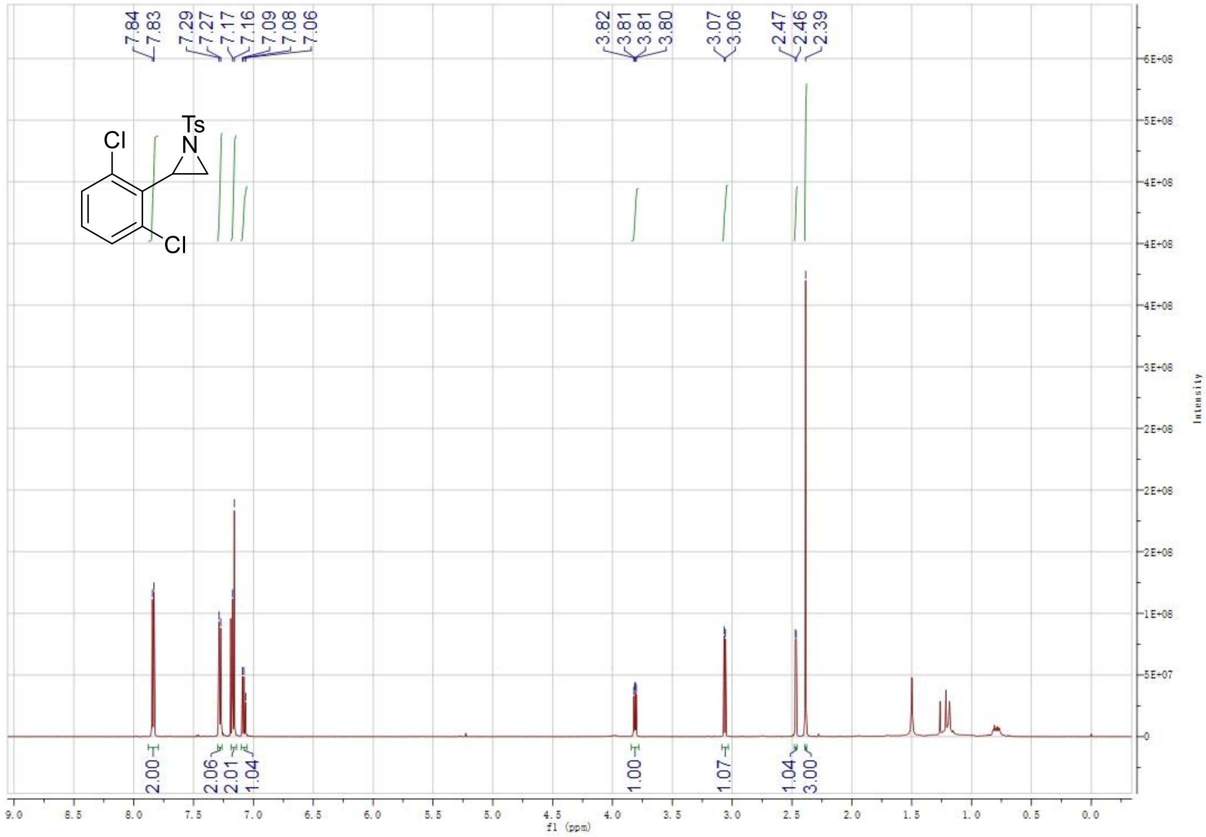

^{13}C NMR (151 MHz, 293 K, $CDCl_3$)

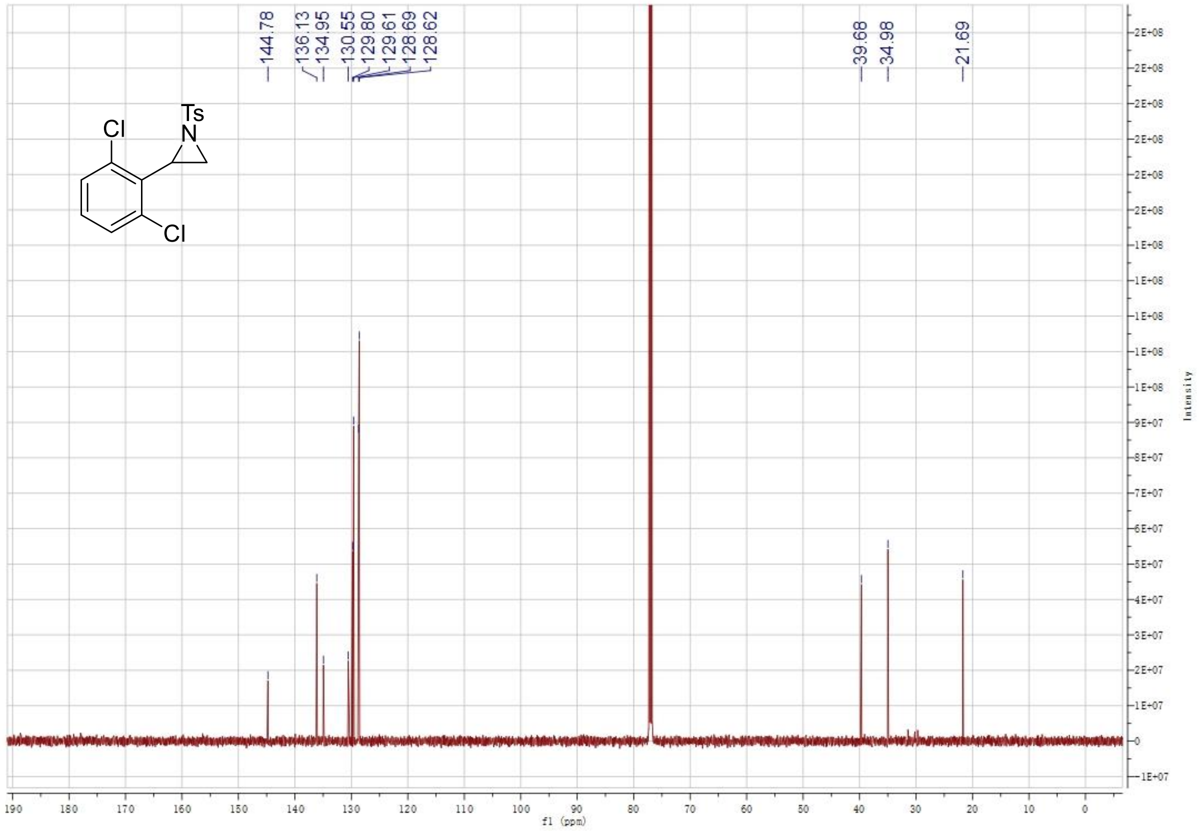

Supplementary Figure 99. *1-Tosyl-1,1a,6,6a-tetrahydroindeno[1,2-b]azirine (**22u**)*

^{1}H NMR (600 MHz, 293 K, $CDCl_3$)

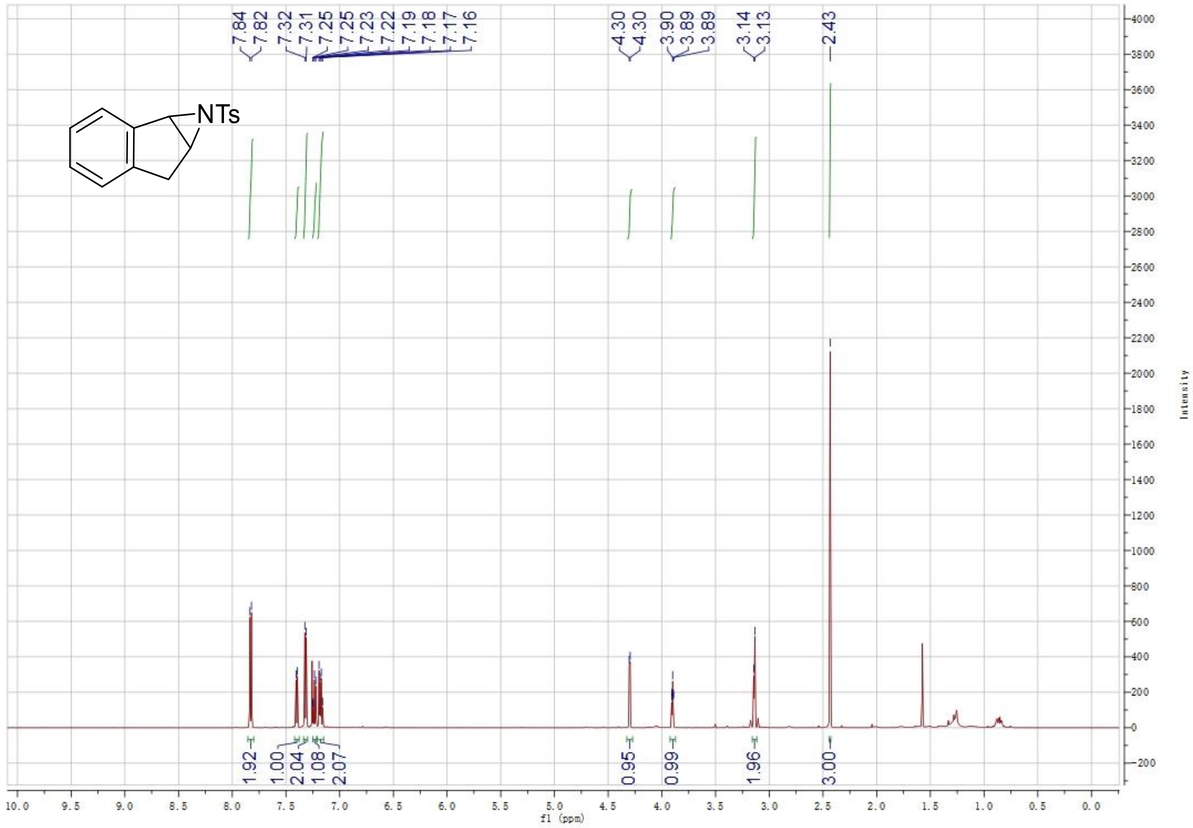

^{13}C NMR (151 MHz, 293 K, $CDCl_3$)

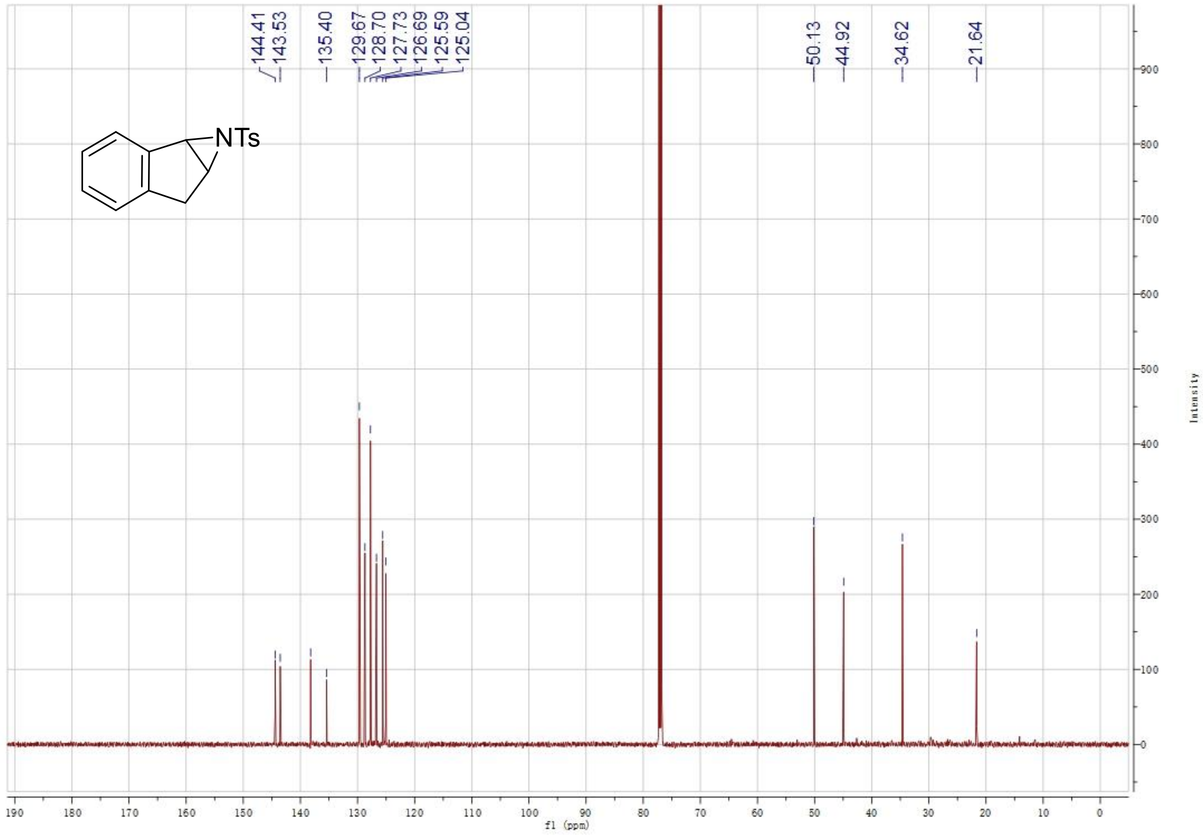

Supplementary Figure 100. *2-Cyclohexyl-1-tosylaziridine (**23a**)*

^{1}H NMR (400 MHz, 293 K, $CDCl_3$)

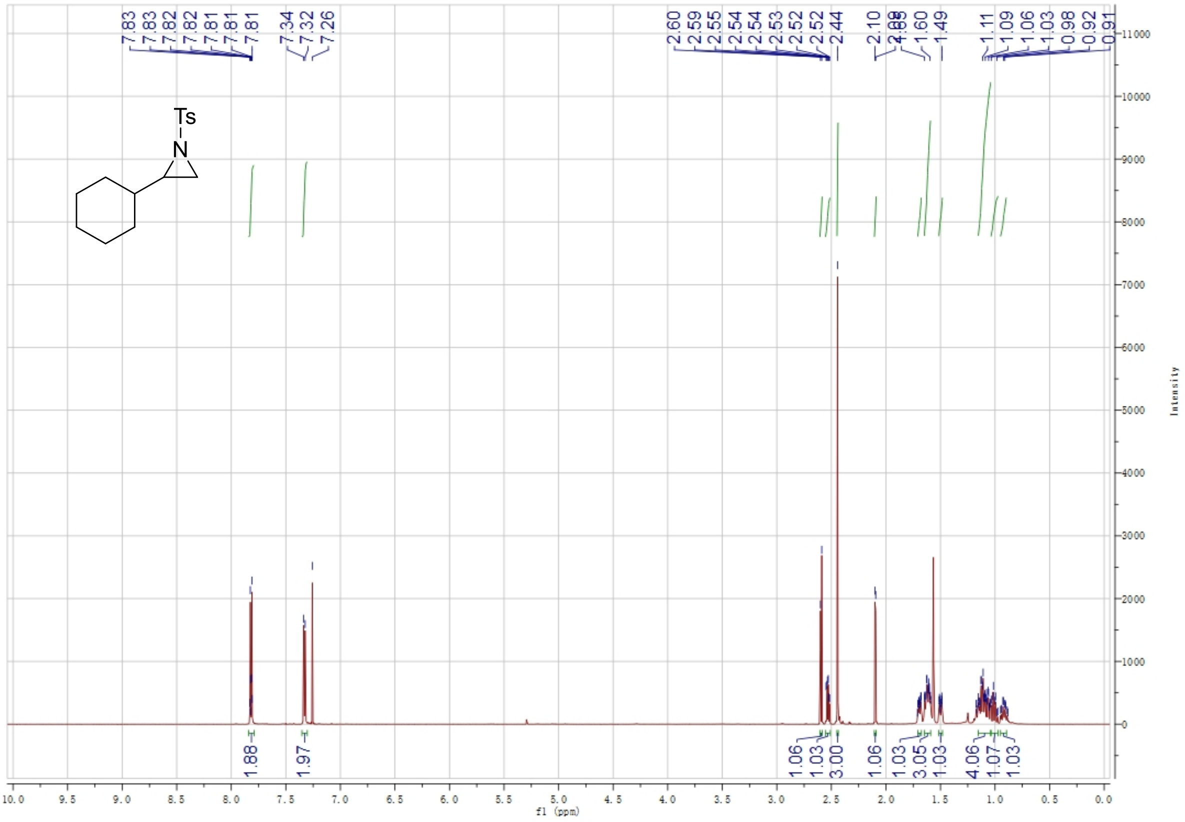

^{13}C NMR (101 MHz, 293 K, $CDCl_3$)

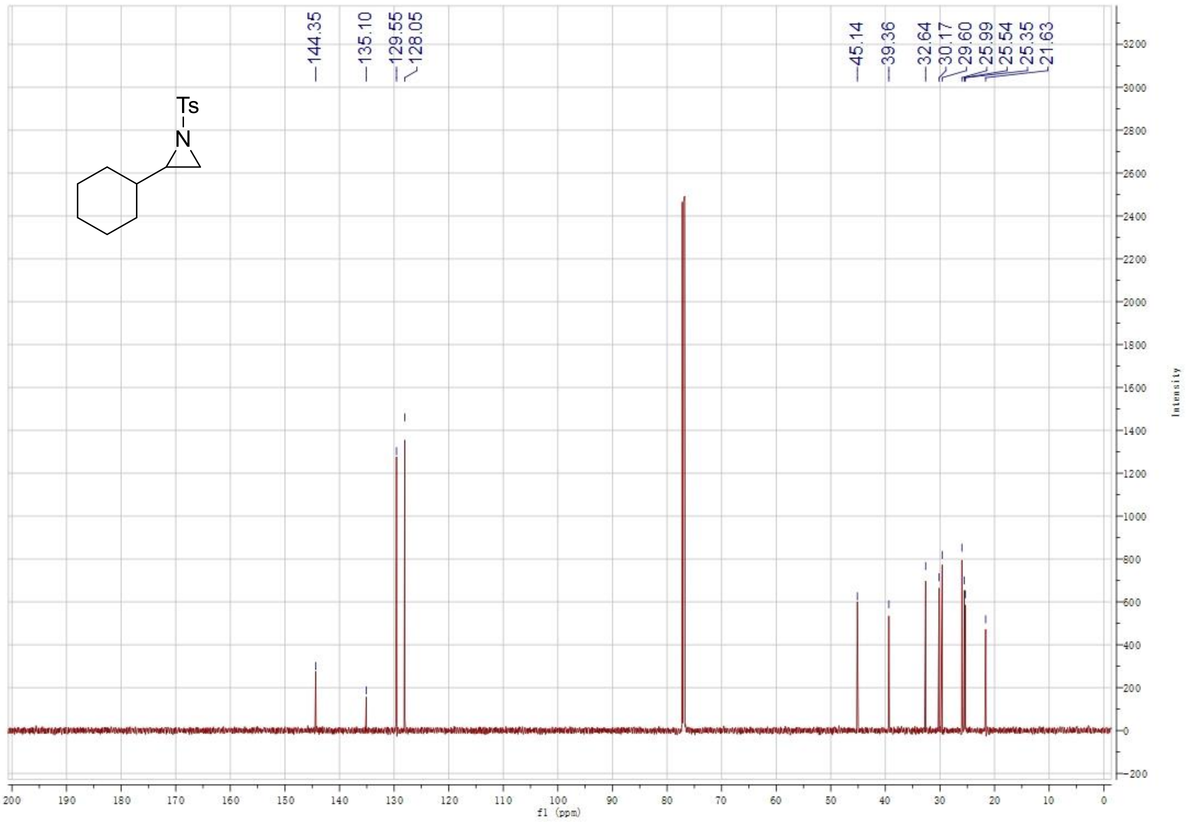

Supplementary Figure 101. *2-Butyl-1-tosylaziridine (**23b**)*

^{1}H NMR (400 MHz, 293 K, $CDCl_3$)

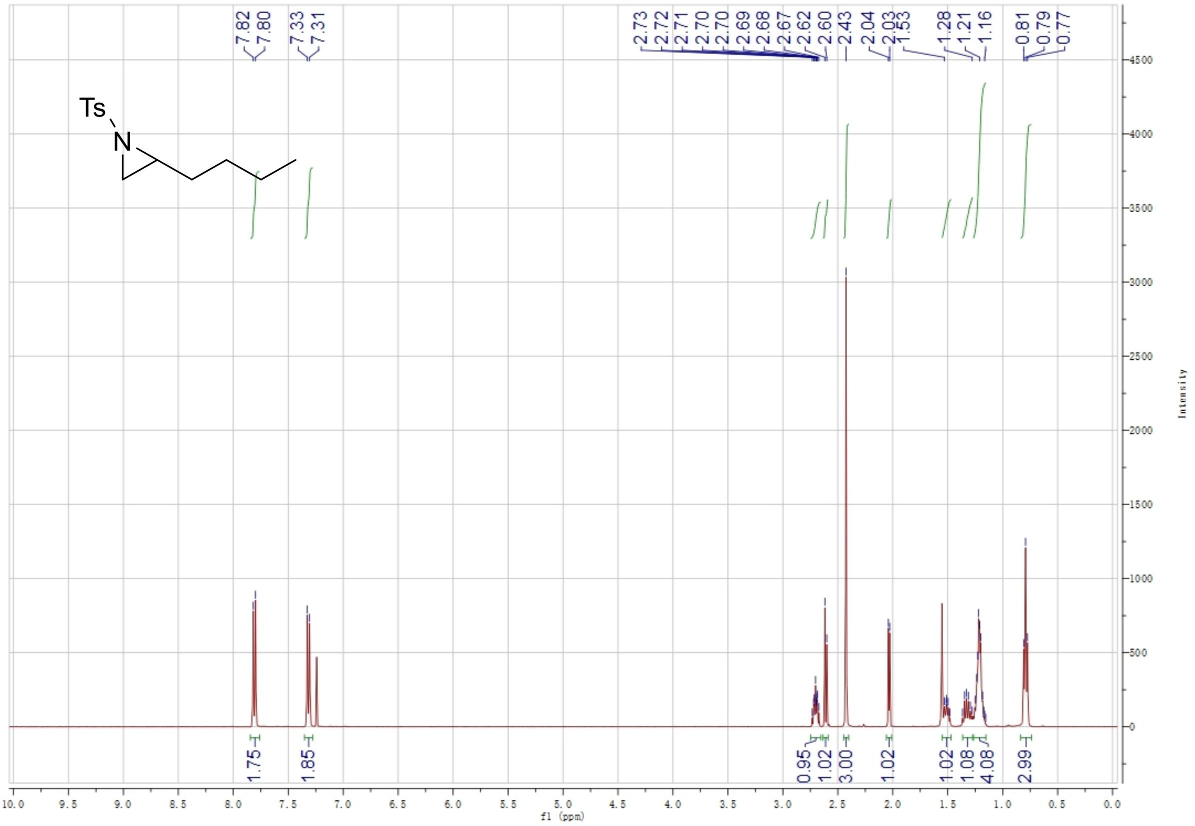

^{13}C NMR (101 MHz, 293 K, $CDCl_3$)

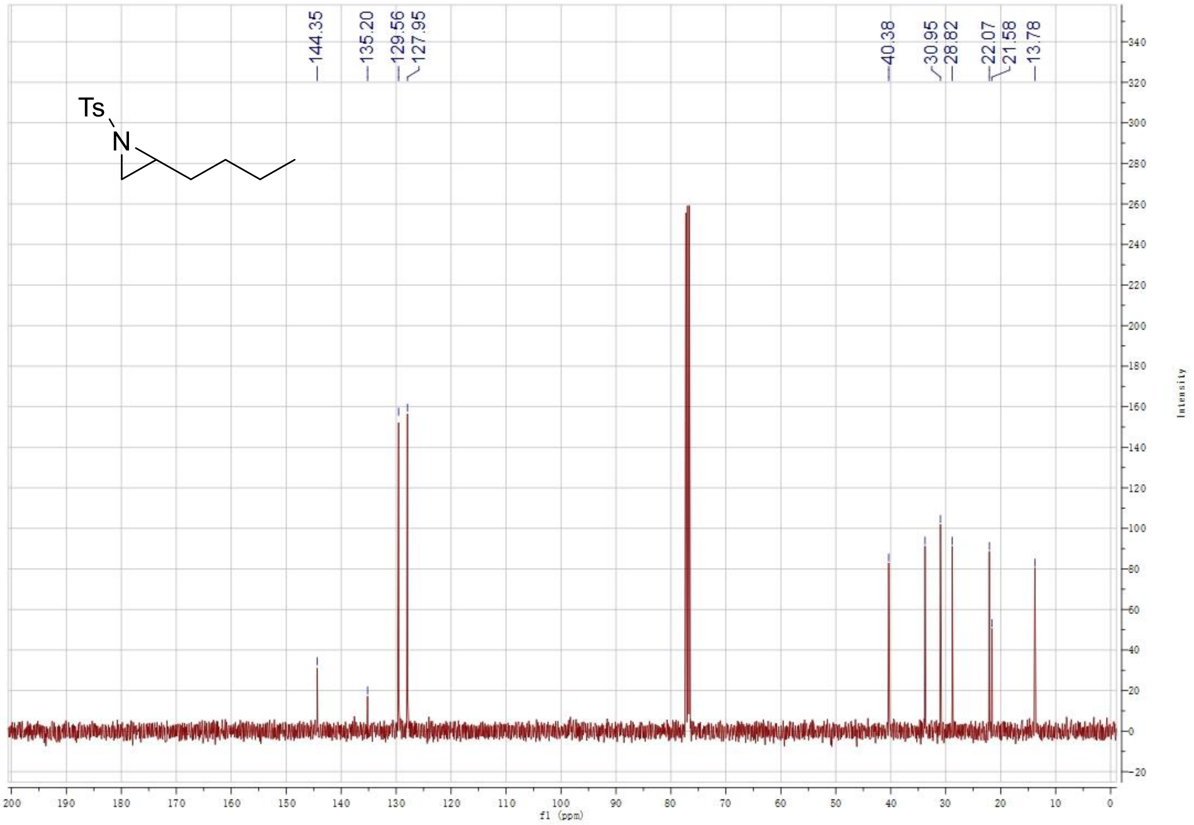

Supplementary Figure 102. *2-Hexyl-1-tosylaziridine* (***23c***)

^{1}H NMR (400 MHz, 293 K, $CDCl_3$)

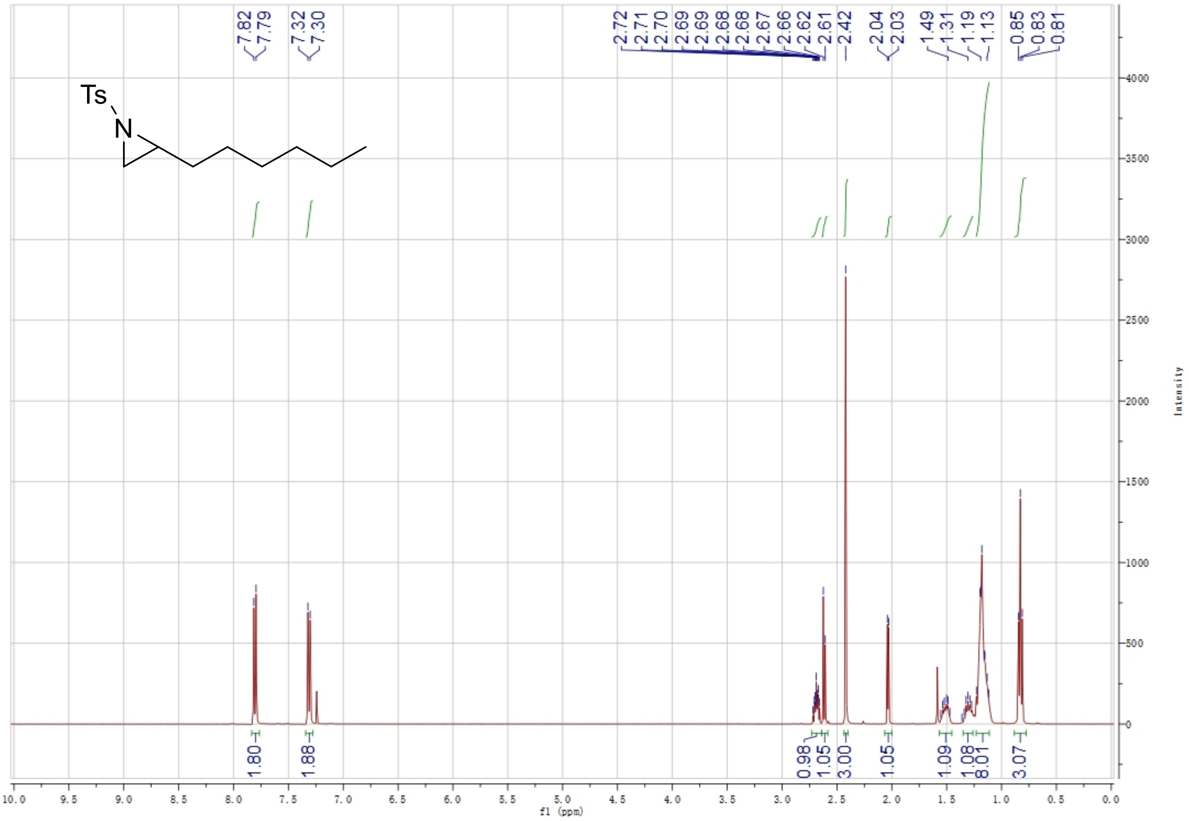

^{13}C NMR (101 MHz, 293 K, $CDCl_3$)

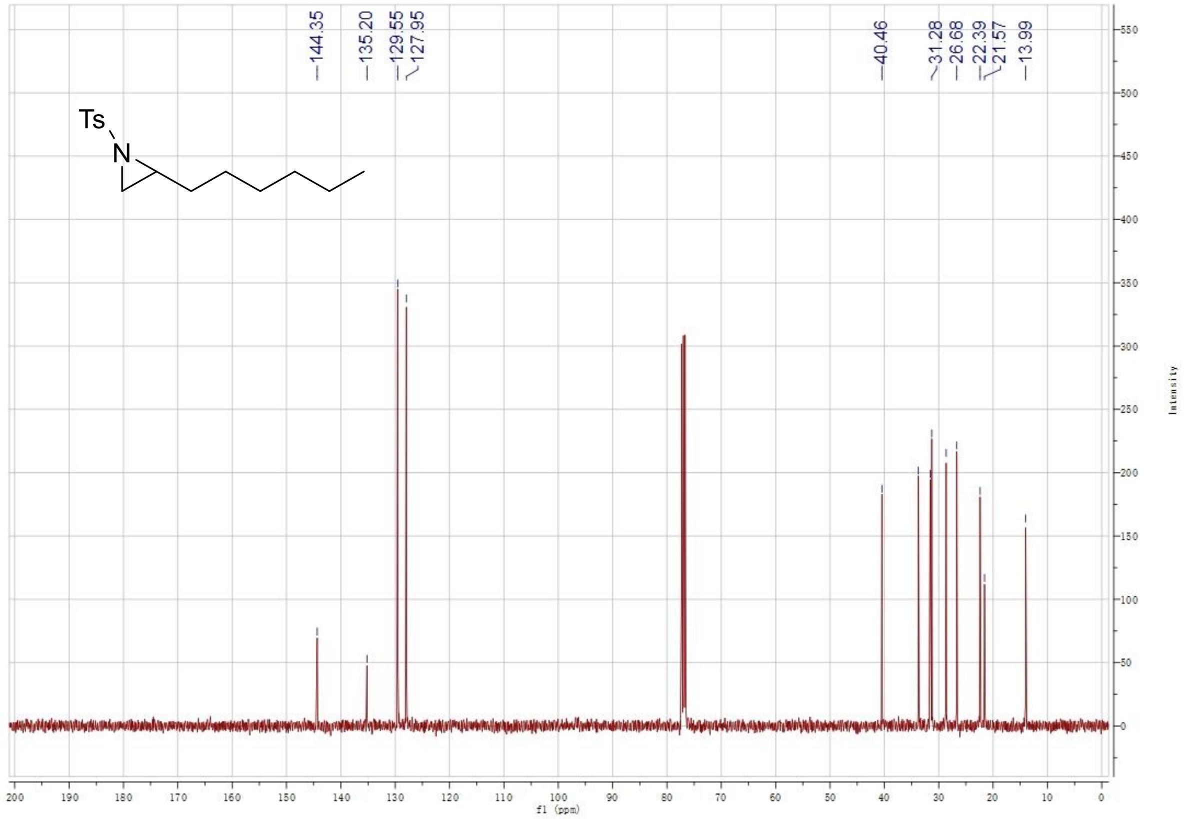

Supplementary Figure 103. *2-Dodecyl-1-tosylaziridine (**23d**)*

^{1}H NMR (400 MHz, 293 K, $CDCl_3$)

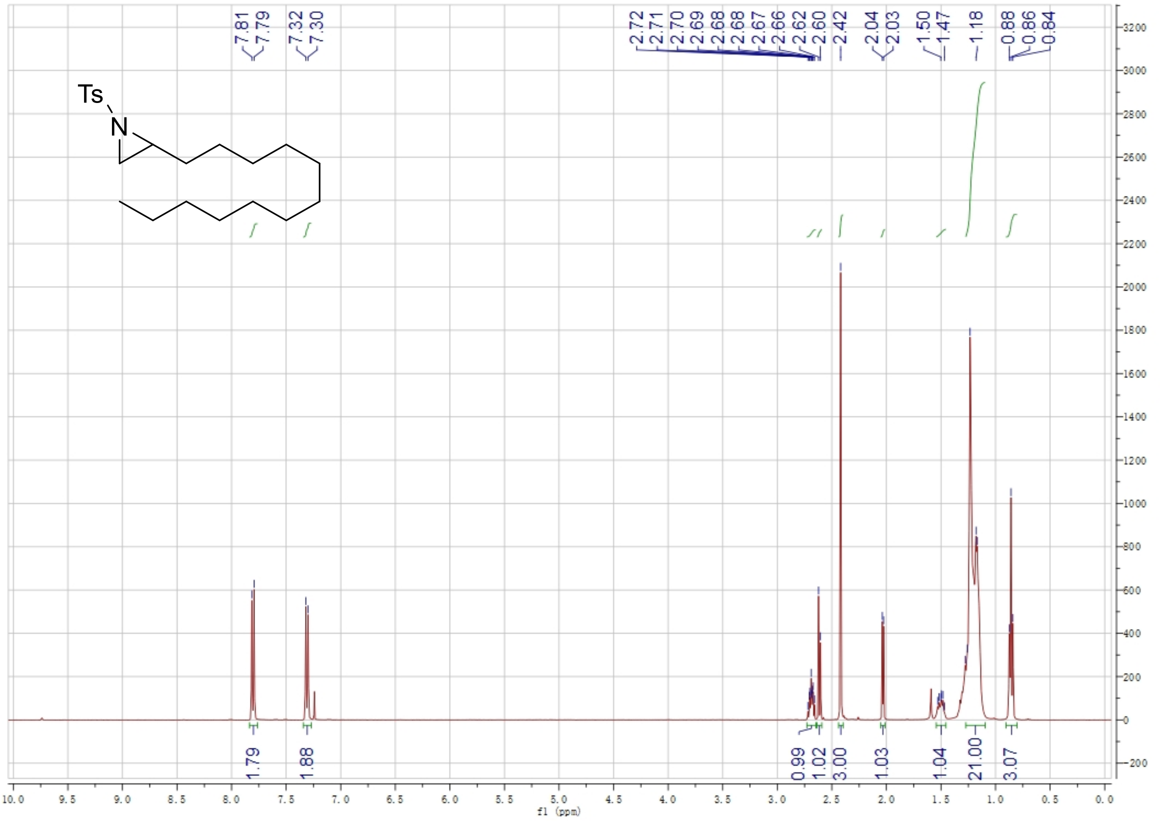

^{13}C NMR (101 MHz, 293 K, $CDCl_3$)

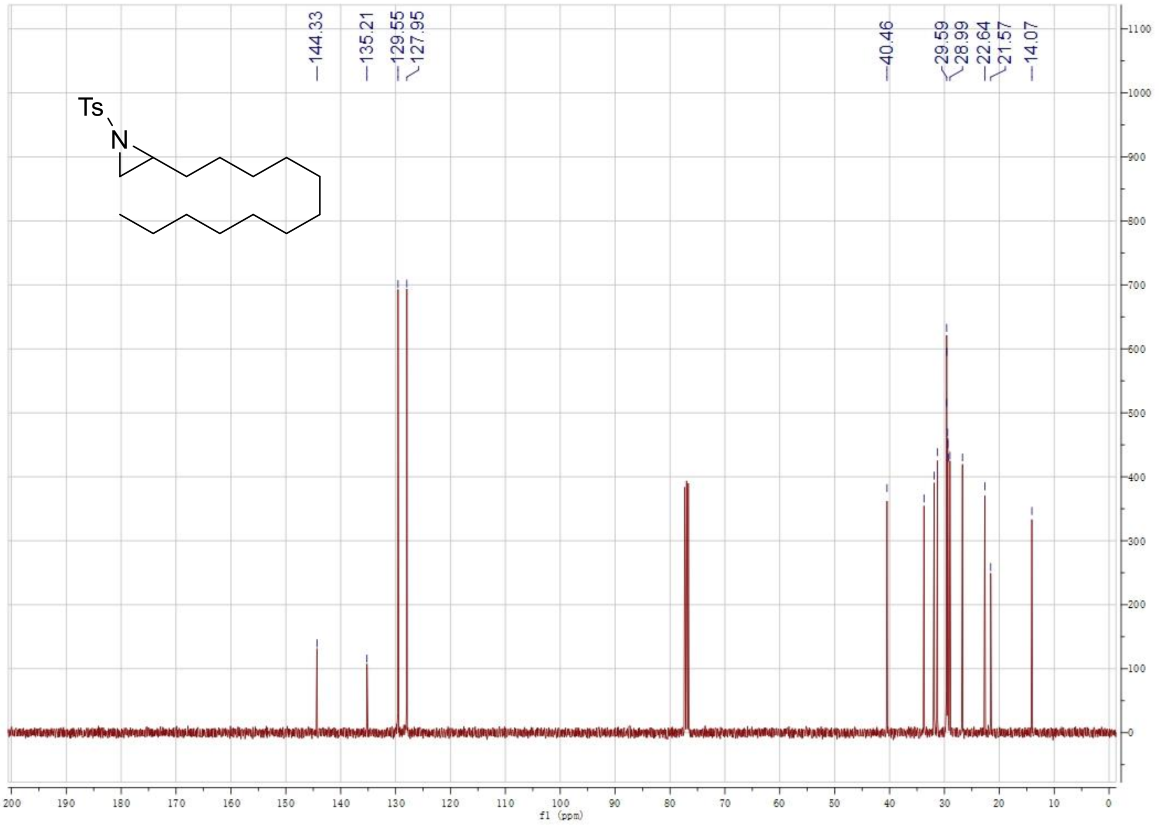

Supplementary Figure 104. *2-(3-Methylbenzyl)-1-tosylaziridine* (***23e***)

^{1}H NMR (400 MHz, 293 K, $CDCl_3$)

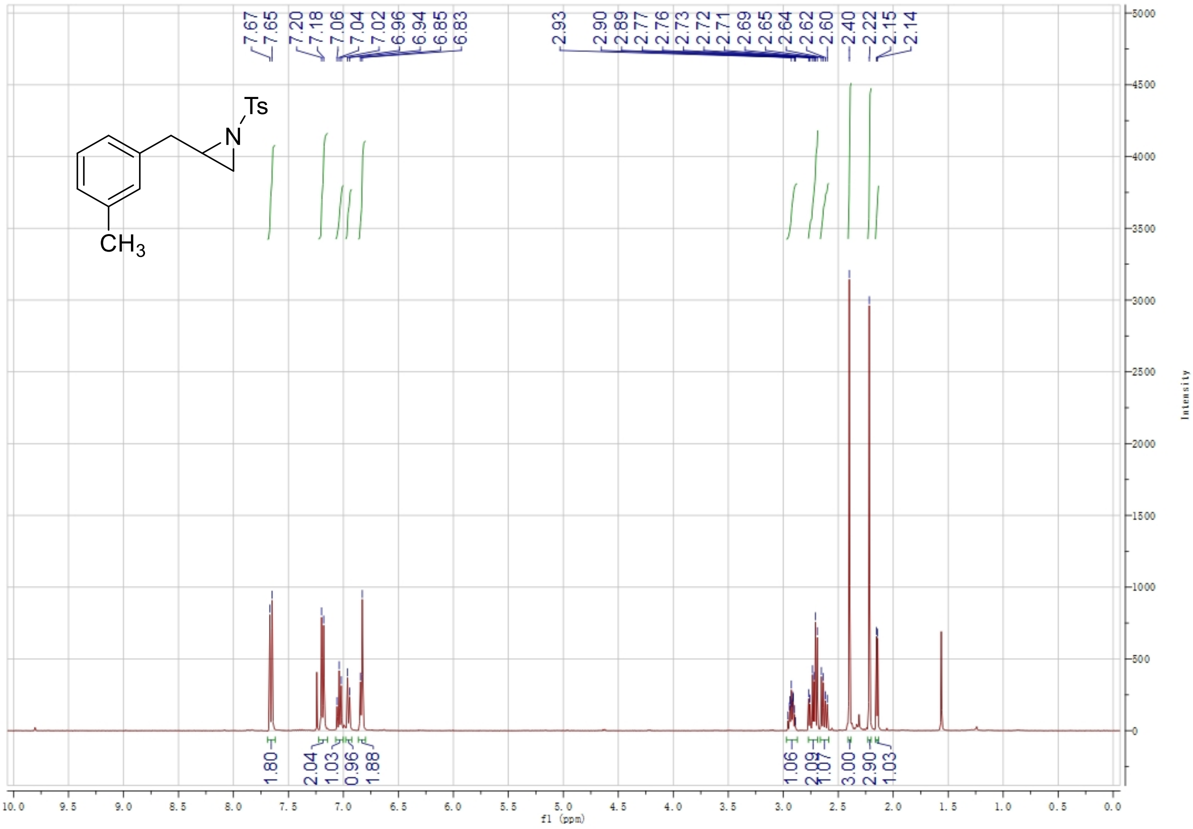

^{13}C NMR (101 MHz, 293 K, $CDCl_3$)

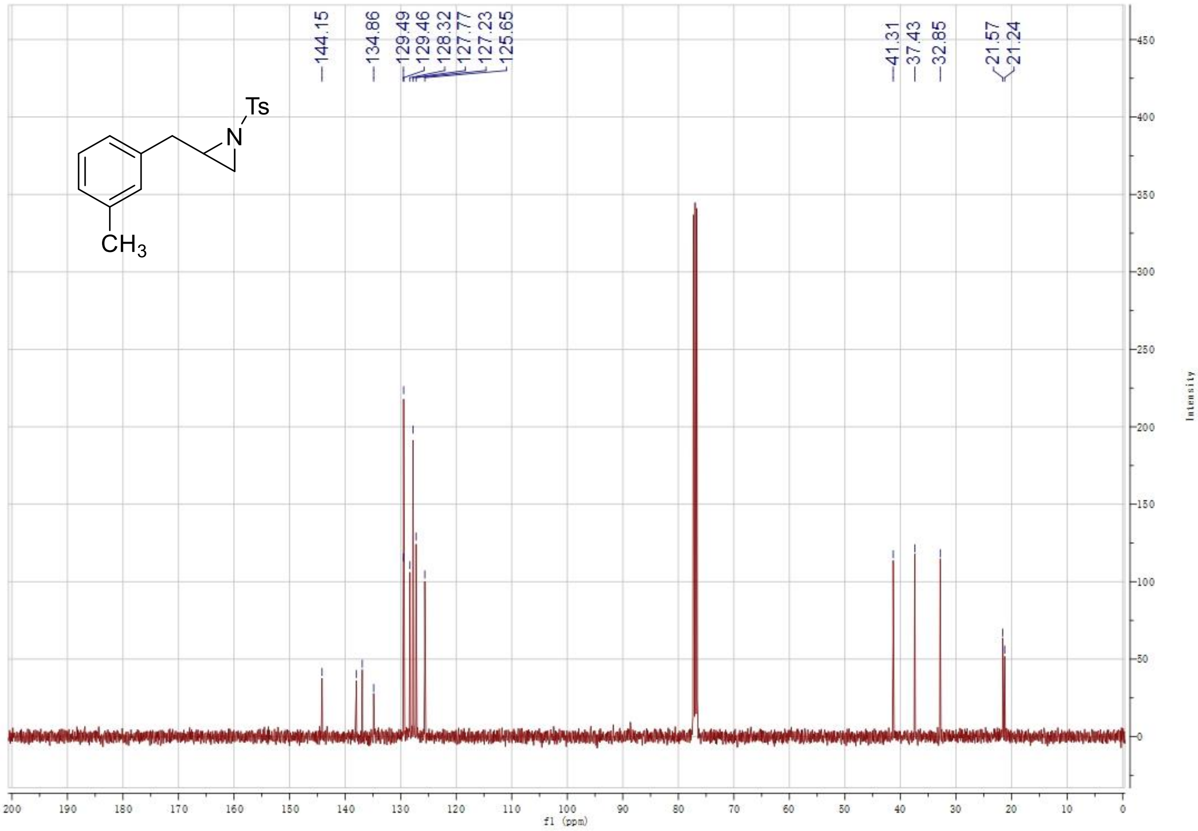

Supplementary Figure 105. *2-Phenethyl-1-tosylaziridine (**23f**)*

^{1}H NMR (400 MHz, 293 K, $CDCl_3$)

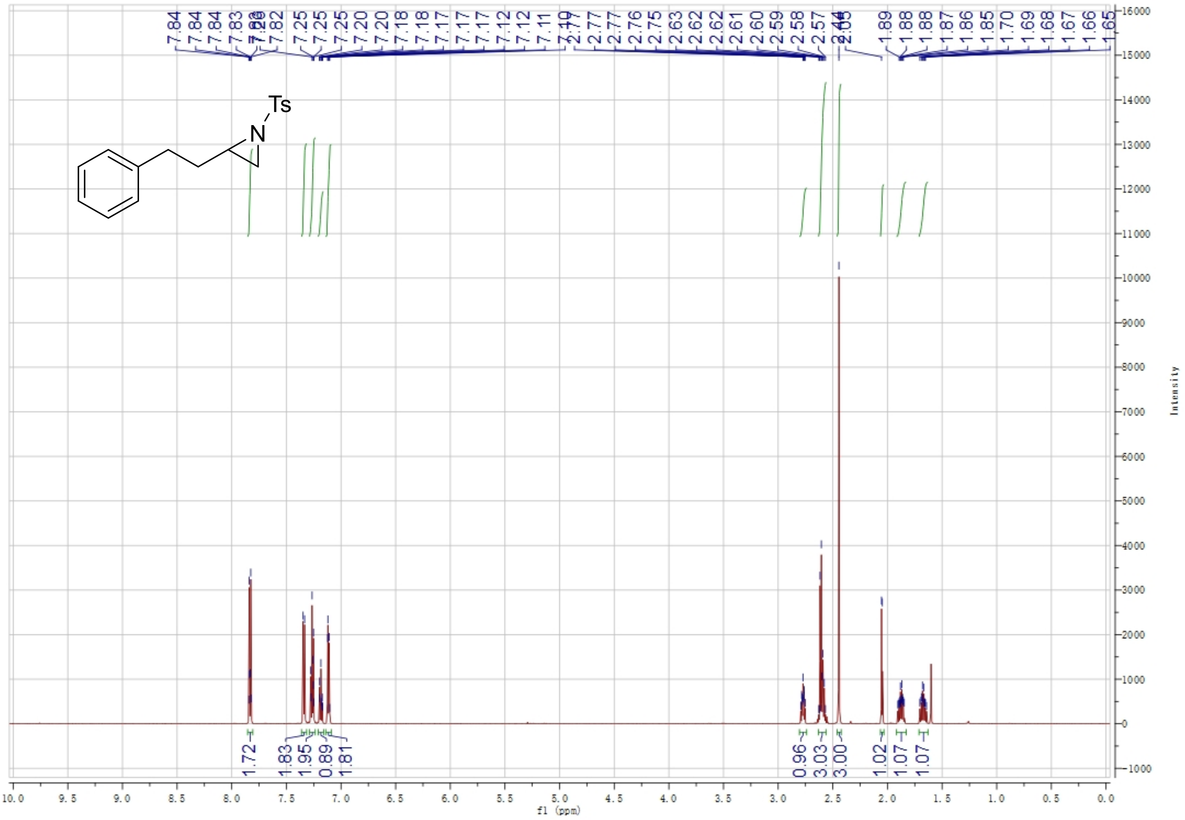

^{13}C NMR (101 MHz, 293 K, $CDCl_3$)

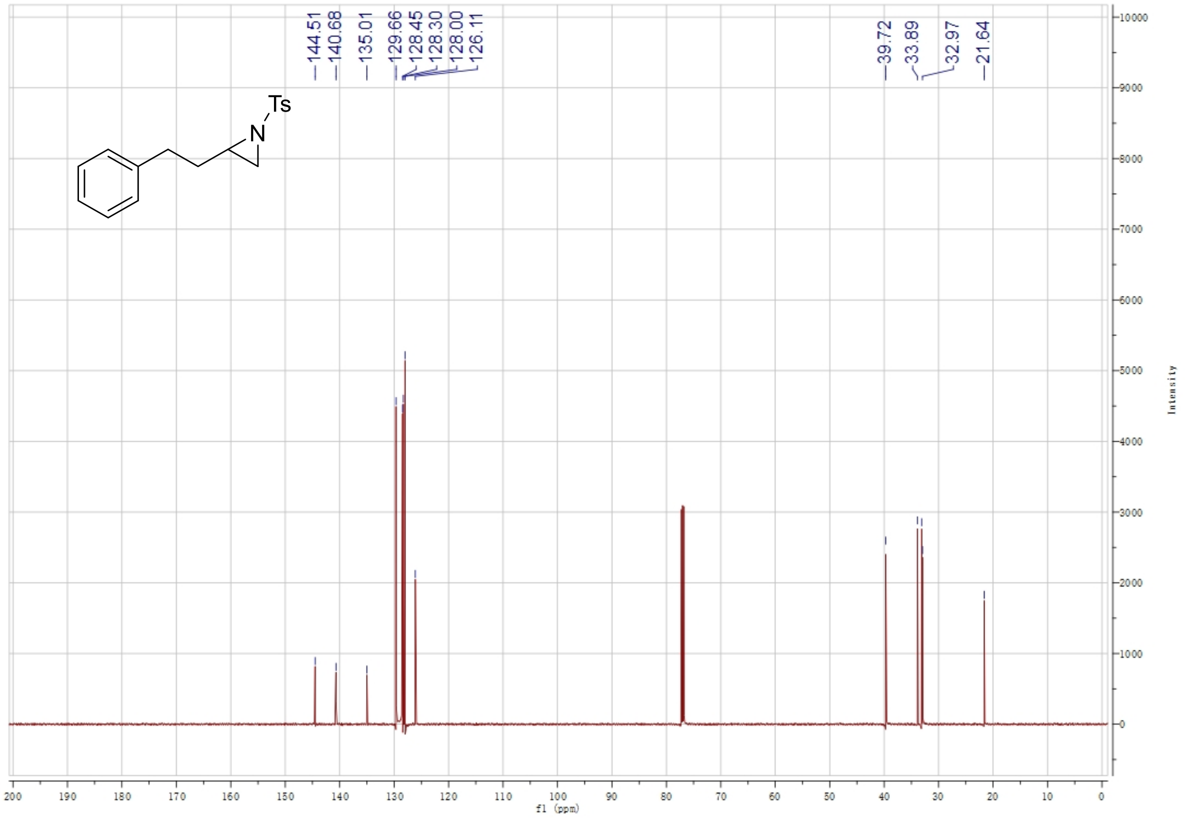

Supplementary Figure 106. *6-Tosyl-6-azabicyclo[3.1.0]hexane (**23g**)*

^{1}H NMR (400 MHz, 293 K, $CDCl_3$)

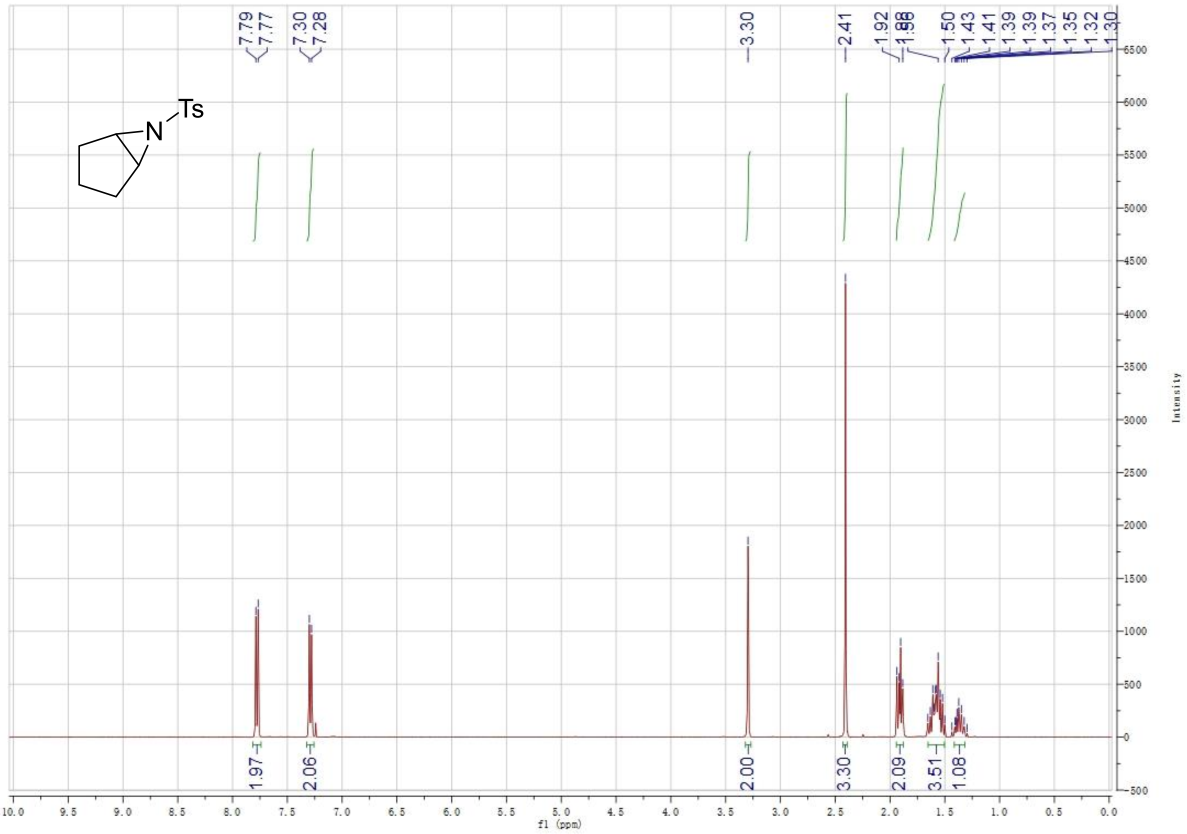

^{13}C NMR (101 MHz, 293 K, $CDCl_3$)

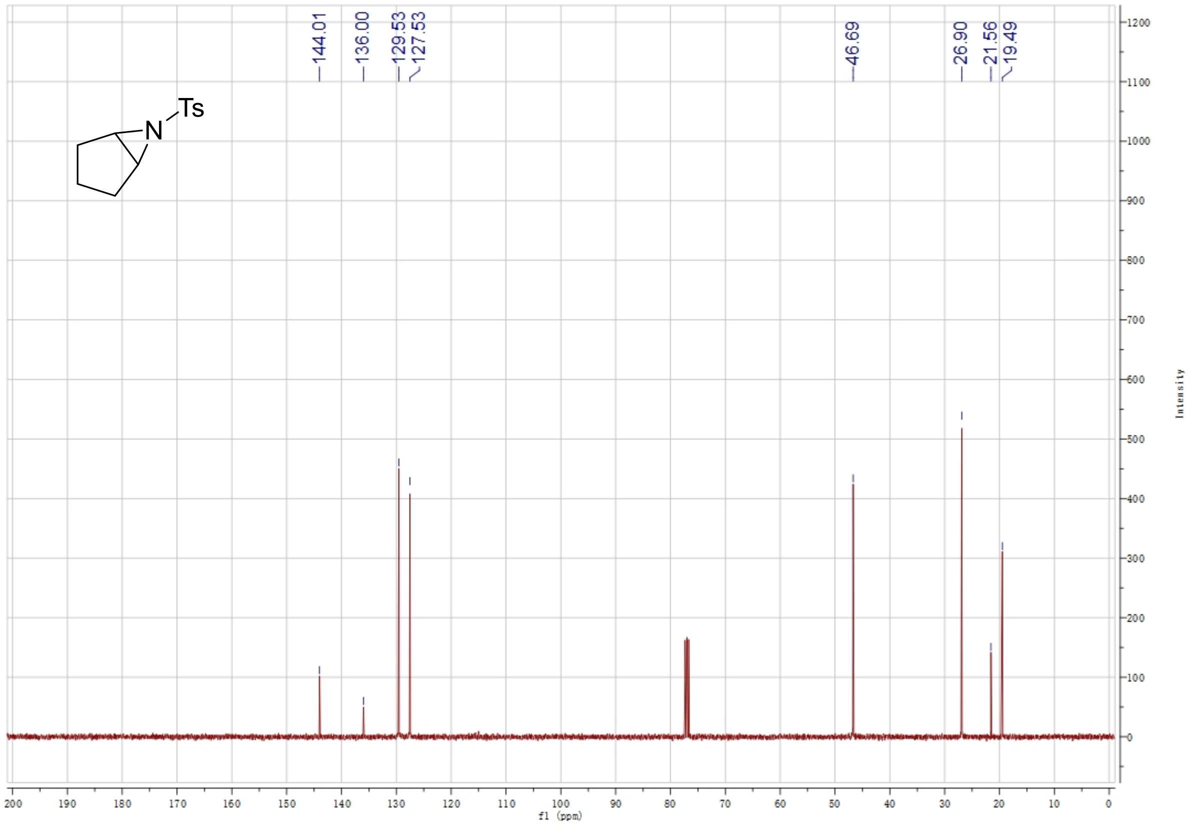

Supplementary Figure 107.

*Methyl (1R,3R,6S)-7-tosyl-7-azabicyclo[4.1.0]heptane-3-carboxylate (**23h**)*

^{1}H NMR (400 MHz, 293 K, $CDCl_3$)

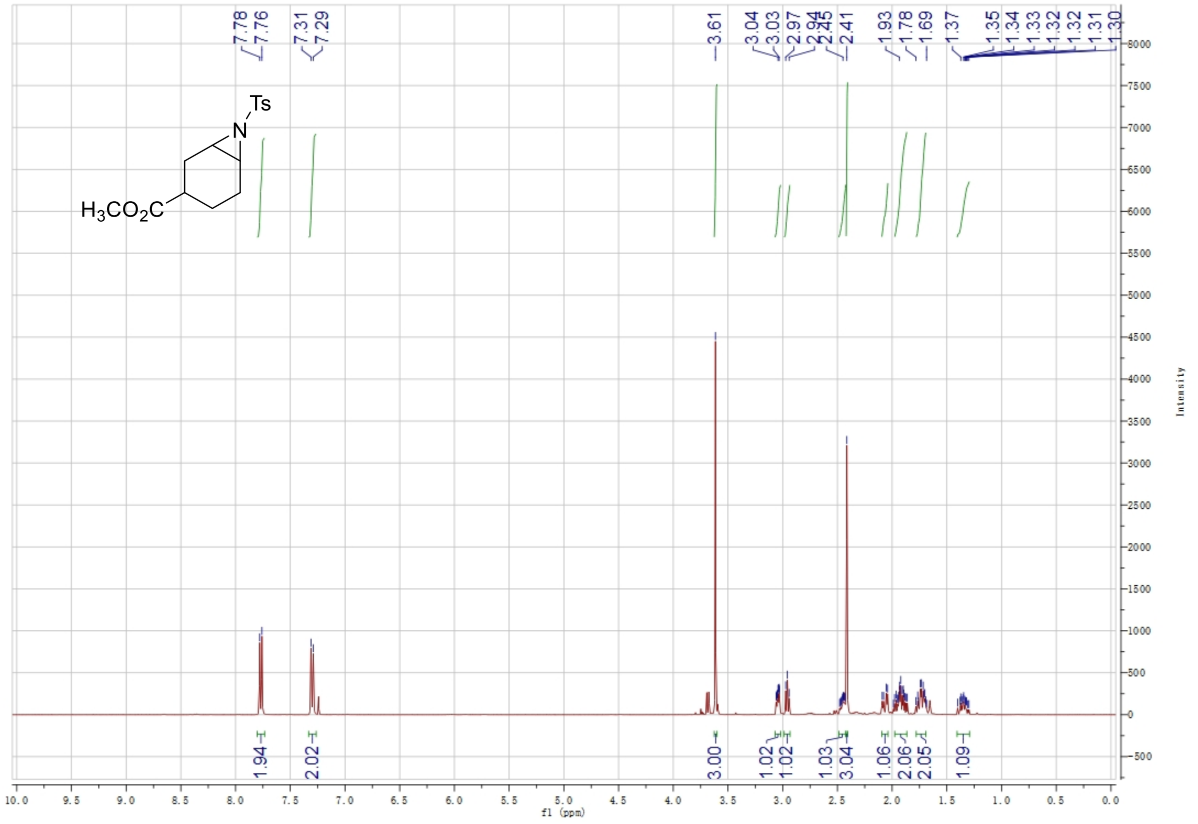

^{13}C NMR (101 MHz, 293 K, $CDCl_3$)

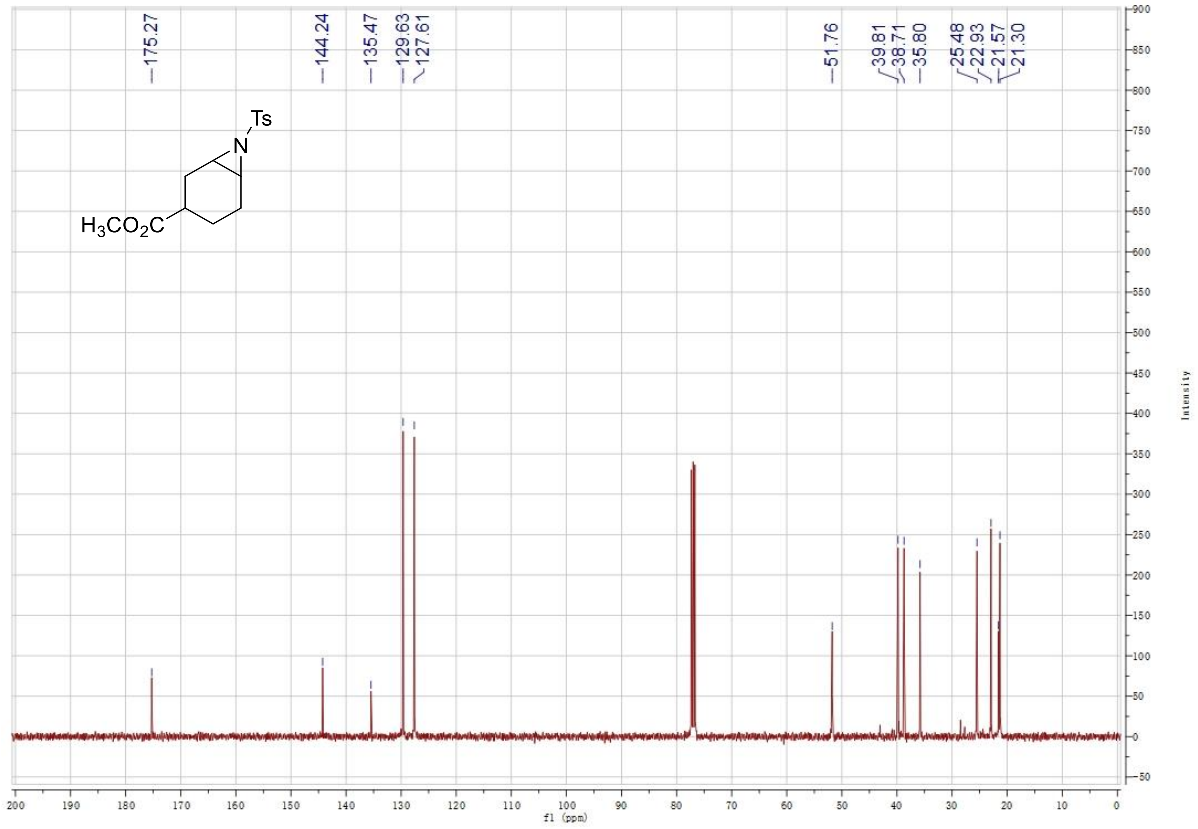

Supplementary Figure 108. *(2Z,4Z,6Z)-9-Tosyl-9-azabicyclo[6.1.0]nona-2,4,6-triene* (**23i**)

^{1}H NMR (400 MHz, 293 K, $CDCl_3$)

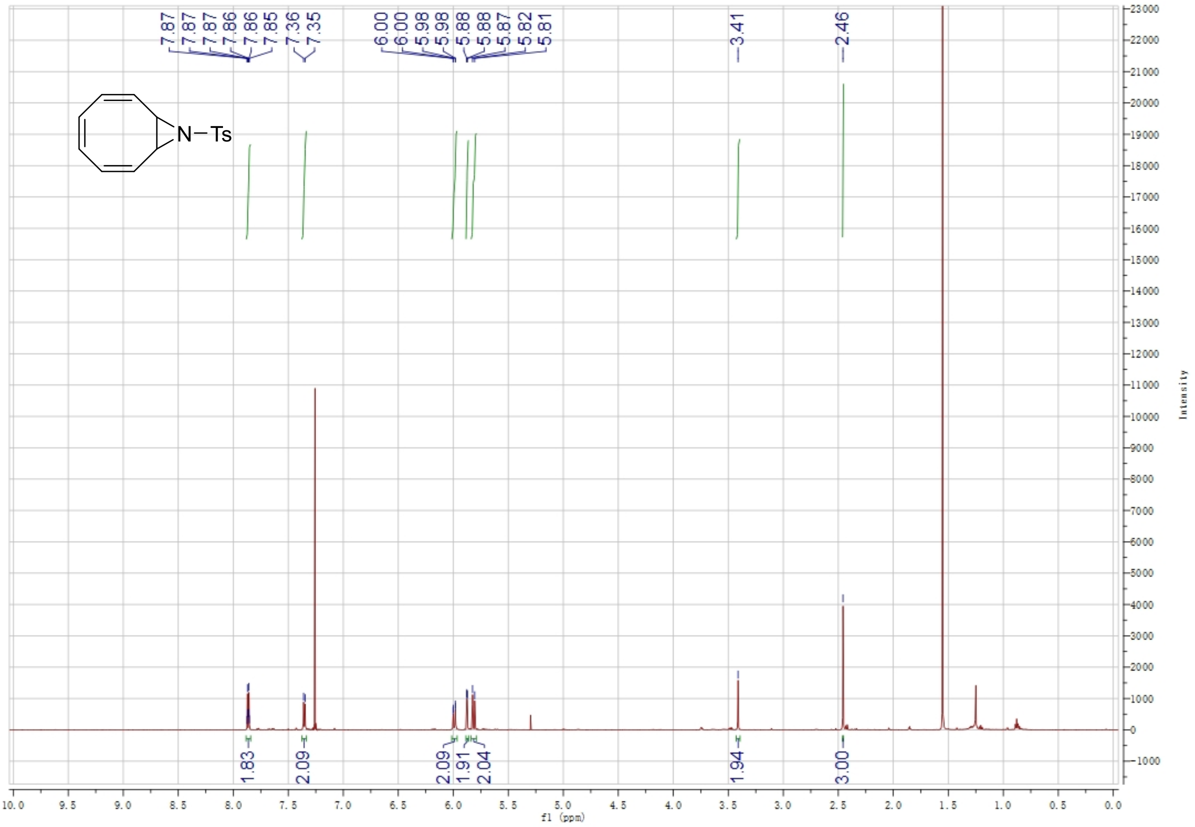

^{13}C NMR (101 MHz, 293 K, $CDCl_3$)

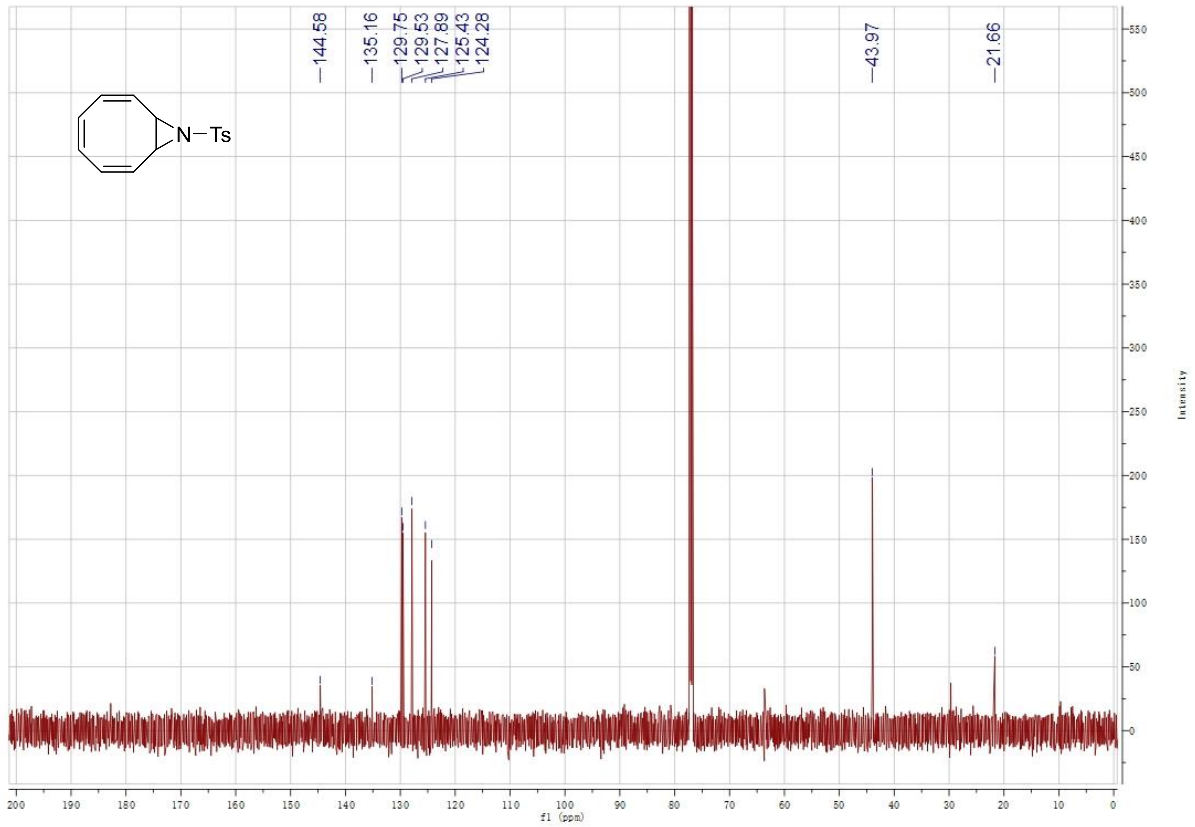

Supplementary References

[1] Yamada, Y.; Yamamoto, T. & Okawara, M. Synthesis and Reaction of New type I－N ylide, N-Tosyliminoiodinane. *Chem. Lett.*, **4**, 361-362 (1975).

[2] Yoshimura, A.; Luedtkeand, M. W. & Zhdankin, V. V. (Tosylimino)phenyl-λ^3-iodane as a Reagent for the Synthesis of Methyl Carbamates via Hofmann Rearrangement of Aromatic and Aliphatic Carboxamides. *J. Org. Chem*. **77**, 2087-2091 (2012).

[3] Coote, S. C.; Moore, S. P.; O'Brien, P.; Whitwood, A. C. & Gilday, J. Organolithium-Mediated Conversion of *β*-Alkoxy Aziridines into Allylic Sulfonamides: Effect of the N-Sulfonyl Group and a Formal Synthesis of (±)-Perhydrohistrionicotoxin. *J. Org. Chem.* **73**, 7852-7855 (2008).

[4] *Gaussian 16*, *Revision C.01*, Frisch, M. J. et al. Gaussian, Inc., Wallingford CT, 2019.

[5] Zhao, Y. & Truhlar, D. G. The M06 suite of density functionals for main group thermochemistry, thermochemical kinetics, noncovalent interactions, excited states, and transition elements: two new functionals and systematic testing of four M06-class functionals and 12 other functionals. *Theor. Chem. Acc.* **120**, 215-241 (2008).

[6] Grimme, S.; Antony, J.; Ehrlich, S. & Krieg, H. A consistent and accurate *ab initio* parametrization of density functional dispersion correction (DFT-D) for the 94 elements H-Pu. *J. Chem. Phys.* **132**, 154104 (2010).

[7] Weigend, F. & Ahlrichs, R. Balanced basis sets of split valence, triple zeta valence and quadruple zeta valence quality for H to Rn: Design and assessment of accuracy. *Phys. Chem. Chem. Phys.* **7**, 3297-3305 (2005).

[8] (a) Fukui, K. Formulation of the Reaction Coordinate. *J. Phys. Chem.* **74**, 4161-4163 (1970). (b) Fukui, K. The Path of Chemical Reactions - the IRC Approach. *Acc. Chem. Res.* **14**, 363-368 (1981).

[9] Marenich, A. V.; Cramer, C. J. & Truhlar, D. G. Universal Solvation Model Based on Solute Electron Density and on a Continuum Model of the Solvent Defined by the Bulk Dielectric Constant and Atomic Surface Tensions. *J. Phys. Chem. B* **113**, 6378-6396 (2009).

[10] CYLview20; Legault, C. Y. Université de Sherbrooke, 2020, (http://www.cylview.org).

[11] Marcus, R. A. On the Theory of Oxidation-Reduction Reactions Involving Electron Transfer. I. *J. Chem. Phys.* **24**, 966-978 (1956).

[12] Lu, T. & Chen, F. Multiwfn: a multifunctional wavefunction analyzer. *J. Comput. Chem.* **33**, 580-592 (2012).
